# Global burden of peripheral artery disease and its risk factors, 1990–2019: a systematic analysis for the Global Burden of Disease Study 2019

**DOI:** 10.1016/S2214-109X(23)00355-8

**Published:** 2023-09-19

**Authors:** GBD 2019 Peripheral Artery Disease Collaborators, Min Seo Kim, Jimin Hwang, Dong Keon Yon, Seung Won Lee, Se Yong Jung, Seoyeon Park, Catherine Owens Johnson, Benjamin A Stark, Christian Razo, Mohammadreza Abbasian, Hedayat Abbastabar, Amir Parsa Abhari, Victor Aboyans, Denberu Eshetie Adane Adane, Oladimeji M Adebayo, Fares Alahdab, Sami Almustanyir, Hany Aly, Edward Kwabena Ameyaw, Jason A Anderson, Catalina Liliana Andrei, Zahra Aryan, Avinash Aujayeb, Sara Bagherieh, Ovidiu Constantin Baltatu, Maciej Banach, Nebiyou Simegnew Bayileyegn, Lindsay M Bearne, Amir Hossein Behnoush, Isabela M Bensenor, Sonu Bhaskar, Ajay Nagesh Bhat, Vivek Bhat, Boris Bikbov, Bagas Suryo Bintoro, Katrin Burkart, Luis Alberto Cámera, Alberico L Catapano, Eeshwar K Chandrasekar, Jaykaran Charan, Vijay Kumar Chattu, Gerald Chi, Isaac Sunday Chukwu, Sheng-Chia Chung, Massimo Cirillo, Kaleb Coberly, Vera Marisa Costa, Omid Dadras, Xiaochen Dai, Thanh Chi Do, Rajkumar Doshi, Michael Ekholuenetale, Islam Y Elgendy, Muhammed Elhadi, Adeniyi Francis Fagbamigbe, Alireza Feizkhah, Ginenus Fekadu, Paramjit Singh Gill, Mohamad Goldust, Mahaveer Golechha, Shi-Yang Guan, Vivek Kumar Gupta, Mostafa Hadei, Najah R Hadi, Ahmad Hammoud, Graeme J Hankey, Netanja I Harlianto, Ahmed I Hasaballah, Shoaib Hassan, Mohammed Bheser Hassen, Golnaz Heidari, Mihaela Hostiuc, Olayinka Stephen Ilesanmi, Masao Iwagami, Mohammad Jokar, Jost B Jonas, Charity Ehimwenma Joshua, Jacek Jerzy Jozwiak, Sina Kazemian, Mohammad Keykhaei, Amirmohammad Khalaji, Moien AB Khan, Sorour Khateri, Biruk Getahun Kibret, Oleksii Korzh, Sindhura Lakshmi Koulmane Laxminarayana, Kewal Krishan, Akshay Kumar, Manoj Kumar, Ambily Kuttikkattu, Tri Laksono, Bagher Larijani, Thao Thi Thu Le, Stephen S Lim, Xuefeng Liu, Stefan Lorkowski, Hassan Magdy Abd El Razek, Kashish Malhotra, Yosef Manla, Andrea Maugeri, Alexios-Fotios A Mentis, Tomislav Mestrovic, Ana Carolina Micheletti Gomide Nogueira de Sá, Andreea Mirica, Erkin M Mirrakhimov, Awoke Misganaw, Manish Mishra, Yousef Mohammad, Ali H Mokdad, Mohammad Ali Moni, Ahmed Al Montasir, Yousef Moradi, Paula Moraga, Negar Morovatdar, Seyed Ali Mousavi-Aghdas, Christopher J L Murray, Mohsen Naghavi, Tapas Sadasivan Nair, Hasan Nassereldine, Zuhair S Natto, Dang H Nguyen, Hien Quang Nguyen, Van Thanh Nguyen, Jean Jacques Noubiap, Bogdan Oancea, Gláucia Maria Moraes Oliveira, Mayowa O Owolabi, Alicia Padron-Monedero, Norberto Perico, Ionela-Roxana Petcu, Amir Radfar, Quinn Rafferty, Mosiur Rahman, Muhammad Aziz Rahman, Pradhum Ram, Sina Rashedi, Ahmed Mustafa Rashid, Salman Rawaf, Giuseppe Remuzzi, Andre M N Renzaho, Malihe Rezaee, Leonardo Roever, Aly M A Saad, Seyedmohammad Saadatagah, Masoumeh Sadeghi, Amirhossein Sahebkar, Mohamed A Saleh, Abdallah M Samy, Milena M Santric-Milicevic, Sadaf G Sepanlou, Allen Seylani, Sadaf Sharfaei, Seyed Afshin Shorofi, Jasvinder A Singh, Paramdeep Singh, Michael Spartalis, Johan Sundström, Ker-Kan Tan, Masayuki Teramoto, Samar Tharwat, Stefanos Tyrovolas, Sahel Valadan Tahbaz, Jef Van den Eynde, Priya Vart, Cong Wang, Fang Wang, Ronny Westerman, Juan Xia, Suowen Xu, Dereje Y Yada, Kazumasa Yamagishi, Naohiro Yonemoto, Mazyar Zahir, Moein Zangiabadian, Armin Zarrintan, Mikhail Sergeevich Zastrozhin, Anasthasia Zastrozhina, Mohammad Zoladl, Simon I Hay, Jae Il Shin, Gregory A Roth

## Abstract

**Background:**

Peripheral artery disease is a growing public health problem. We aimed to estimate the global disease burden of peripheral artery disease, its risk factors, and temporospatial trends to inform policy and public measures.

**Methods:**

Data on peripheral artery disease were modelled using the Global Burden of Disease, Injuries, and Risk Factors Study (GBD) 2019 database. Prevalence, disability-adjusted life years (DALYs), and mortality estimates of peripheral artery disease were extracted from GBD 2019. Total DALYs and age-standardised DALY rate of peripheral artery disease attributed to modifiable risk factors were also assessed.

**Findings:**

In 2019, the number of people aged 40 years and older with peripheral artery disease was 113 million (95% uncertainty interval [UI] 99·2–128·4), with a global prevalence of 1·52% (95% UI 1·33–1·72), of which 42·6% was in countries with low to middle Socio-demographic Index (SDI). The global prevalence of peripheral artery disease was higher in older people, (14·91% [12·41–17·87] in those aged 80–84 years), and was generally higher in females than in males. Globally, the total number of DALYs attributable to modifiable risk factors in 2019 accounted for 69·4% (64·2–74·3) of total peripheral artery disease DALYs. The prevalence of peripheral artery disease was highest in countries with high SDI and lowest in countries with low SDI, whereas DALY and mortality rates showed U-shaped curves, with the highest burden in the high and low SDI quintiles.

**Interpretation:**

The total number of people with peripheral artery disease has increased globally from 1990 to 2019. Despite the lower prevalence of peripheral artery disease in males and low-income countries, these groups showed similar DALY rates to females and higher-income countries, highlighting disproportionate burden in these groups. Modifiable risk factors were responsible for around 70% of the global peripheral artery disease burden. Public measures could mitigate the burden of peripheral artery disease by modifying risk factors.

**Funding:**

Bill & Melinda Gates Foundation.

## Introduction

Lower extremity peripheral arterial disease is an atherosclerotic disease of the peripheral vasculature leading to arterial stenosis or occlusion of the lower limbs, which can manifest as intermittent claudication, ischaemic pain, and functional impairment.^1^, ^2^ Peripheral artery disease is a growing public health problem due to its high and rising prevalence worldwide; however, it often remains unrecognised and undertreated.^3^, ^4^

Although peripheral artery disease has been studied extensively in high-income countries,^5^, ^6^, ^7^ the burden of peripheral artery disease in countries with lower socioeconomic development has been overlooked. However, low-income and middle-income countries are undergoing an epidemiological transition in which the prevalence and burden of cardiovascular diseases have been increasing steeply, probably due to industrialisation, urbanisation, and an increase in metabolic risk factors.^5^, ^8^ Accounting for population size, more than half of individuals with peripheral artery disease globally live in low-income and middle-income countries, despite the higher prevalence of peripheral artery disease reported in high-income countries.^9^, ^10^ It is crucial to understand a comprehensive global picture encompassing all sociodemographic and spatiotemporal trends of peripheral artery disease.

Because peripheral artery disease involves lifelong disability, measuring cross-sectional prevalence alone would not fully capture the disease burden. However, previous systematic reviews that have estimated global burden have accessed only the prevalence of peripheral artery disease,^9^, ^10^ leaving global, regional, and national disability caused by peripheral artery disease unaddressed. The present study aimed to provide disability estimates (ie, disability-adjusted life years [DALYs]), as well as prevalence estimates, for the global distribution of peripheral artery disease burden by analysing data from the Global Burden of Disease, Injuries, and Risk Factors Study (GBD) 2019.

**Research in context**

**Evidence before this study**

We searched PubMed on December 23, 2022, for articles in English using the search terms (“peripheral artery disease*”[tiab] OR “peripheral vascular disease*”[tiab] OR “peripheral artery occlusive disease*”[tiab] OR “peripheral obliterative arteriopathy”[tiab]) AND (“prevalence*”[tiab] OR “incidence*”[tiab] OR “mortality”[tiab] OR “DALY*”[tiab]) AND (“systematic review*”[tiab] OR “meta-analysis”[tiab]). No other restrictions were applied to the search. Of 298 published articles, two systematic reviews and meta-analyses reported on global and regional prevalence for peripheral artery disease, three reported on national or regional specific estimates, six reported on the prevalence of specific conditions in patients with peripheral artery disease, and two reported on peripheral artery disease prevalence in specific populations. The Global Peripheral Artery Disease Study has estimated the global and regional prevalence of peripheral artery disease in 2010 (Fowkes and colleagues; analysis of 34 studies) and 2015 (Song and colleagues; 119 studies). However, they have not assessed death or disability attributed to peripheral artery disease. We also curated one Global Burden of Disease, Injuries, and Risk Factors Study (GBD) 2019 study on peripheral artery disease, but it did not address disability-adjusted life-years (DALYs) associated with the disease.

**Added value of this study**

The present study aimed to provide prevalence, incidence, death, and disability estimates (ie, DALYs) to map the global distribution of burden due to peripheral artery disease by analysing data from GBD 2019. Because peripheral artery disease involves lifelong disability, measuring cross-sectional prevalence alone would not fully capture the disease burden of peripheral artery disease. As part of GBD 2019, this study provides estimates for the burden of peripheral artery disease in 21 GBD regions and 204 countries and territories from 1990 to 2019 by age, sex, and country sociodemographic level. Peripheral artery disease burden was measured by prevalence, incidence, DALYs, and mortality, as well as the peripheral artery disease-related DALYs attributable to modifiable risk factors. The global prevalence of peripheral artery disease was higher in older individuals and, consistent with the findings of Fowkes and colleagues and Song and colleagues, prevalence was higher in females than males. However, the overall disease burden measured by DALYs was similar in both sexes, suggesting a disproportionate burden among males and indicating the need for equivalent attention and care for peripheral artery disease in both sexes. Peripheral artery disease prevalence was higher in countries with high Socio-demographic Index (SDI) and income and lower in countries with low SDI and income, but DALY rates in countries with low SDI and income were disproportionate to prevalence, suggesting that peripheral artery disease has not been adequately managed in these countries. Lastly, in 2019, almost 70% of total peripheral artery disease DALYs globally were attributable to risk factors.

**Implications of all the available evidence**

Despite the lower prevalence of peripheral artery disease in males and low-income countries, these groups showed similar DALY rates to females and higher-income countries, highlighting a disproportionate burden that demands improved action and care. Modifiable risk factors were responsible for around 70% of the peripheral artery disease burden, and therefore implementing interventions aimed at decreasing exposure to risk factors will be crucial to mitigating the global burden of peripheral artery disease. This GBD study can help policy makers direct evidence-based health-care planning, prevention, and resource allocation for those with peripheral artery disease who are at increased risk of disability due to pain and cardiovascular complications. Based on the burden estimations, public health and research priorities should include: developing prevention strategies for peripheral artery disease risk factors; providing care and attention to vulnerable groups; and enriching screening and prevention of peripheral artery disease in lower-income countries.

## Methods

### Overview

Methods relevant to peripheral artery disease GBD are described briefly in this section and in detail in the appendix (pp 2–11, 331–335). For this study, we obtained estimates of incidence, prevalence, cause-specific mortality, years of life lost (YLLs), years lived with disability (YLDs), and DALYs for peripheral artery disease from GBD 2019, as described in the original GBD 2019 report.^11^, ^12^, ^13^, ^14^, ^15^

### Mortality estimates

Detailed methodology for cause-specific mortality estimation is described in the appendix (pp 51–91) and in previous publications.^11^ Cause-specific mortality for peripheral artery disease was estimated using the Cause of Death Ensemble model (CODEm) software with vital registration records as input data.^11^ International Classification Disease (ICD) codes in vital registration records were mapped to the GBD cause list (ICD 10: I70.2–I70.8, I73–I73.9, and ICD 9: 440.2, 440.4, 443.0–443.9). Non-specific, intermediate, or implausible causes of death (eg, “heart disease, unspecified”, “heart failure”, “senility”, or “hypertension”) were reassigned to correct underlying causes of death, including peripheral artery disease, via a set of redistribution algorithms developed for GBD 2019.^11^ The garbage code redistribution algorithm is described in detail in the appendix (pp 63–69). Country-level covariates associated with peripheral artery disease were included to inform the model.

CODEm produces estimates of cause-specific mortality by age, sex, and location for each year with use of an ensemble of modelling methods with varying choices of covariates determined by model performance in out-of-sample predictive validity testing.^11^ Possible covariates were selected based on a priori knowledge of the association between the covariate and peripheral artery disease; this list is in the appendix (pp 2–3). Covariates and combinations of covariates were tested for statistical significance and plausibility (the coefficient must be in the expected direction). Covariates meeting these criteria are retained in the final model.^11^ Detailed methods describing the covariate selection process used in CODEm are in the appendix (pp 80–82) and elsewhere.^16^ The results obtained with the Ensemble models were then adjusted by scaling them within the fraction of deaths due to all cardiovascular diseases and all-cause mortality. The 2·5th and 97·5th percentiles of the posterior distribution were used to determine uncertainty intervals (UIs).

### Morbidity estimates

The studies eligible for assessing peripheral artery disease prevalence were those that included an ankle brachial index (ABI) measurement and defined peripheral artery disease as ABI less than or equal to 0**·**90. We excluded literature with different ABI cutoffs to minimise inconsistency. In addition to published studies, we also included health system administrative data, including outpatient claims data for prevalence assessment. We adjusted administrative health-care data using literature data reporting directly measured ABI values as reference data according to the standard adjustment procedure outlined in the appendix (pp 93–116). Details of the search strategy and a full list of the input data sources used in the morbidity analysis are in the appendix (pp 5–9). When calculating YLDs, we only took into account burden from peripheral artery disease with intermittent claudication. Intermittent claudication was defined clinically,^17^ as leg pain on exertion in those with an ABI less than or equal to 0·90. We used DisMod-MR to model the proportion of peripheral artery disease with intermittent claudication and used the proportion of intermittent claudication to split the overall prevalence of peripheral artery disease into symptomatic and asymptomatic peripheral artery disease. This approach has been used in previous GBD papers to split prevalence of disease by stage,^18^ symptom,^19^ and severity.^20^ The list of studies we used to calculate the proportion of claudication and further details are provided in the appendix (pp 6–11).

Estimates of peripheral artery disease prevalence and the proportion of peripheral artery disease cases with intermittent claudication were calculated using two separate DisMod-MR 2.1 models.^11^ DisMod-MR is a Bayesian geospatial disease modelling approach that uses different disease parameters (eg, prevalence, incidence, remission, and mortality), epidemiological relationships between these parameters, and geospatial patterns to generate disease estimates. The model ensures consistency among all disease parameters by employing differential equations with suitable boundary conditions. The tool incorporates an offset log-normal model with fixed effects for location-specific covariates and random effects for locations. The covariates included in the models are listed in the appendix (pp 101–124). Estimates were made for seven super-regions, 21 world regions, and 204 countries and territories with use of a geographic cascade, as described in the appendix (pp 116–117). Disease distributions from higher geographical levels were used as priors to information for the next levels. The peripheral artery disease DisMod-MR models were evaluated based on comparisons with estimates from previous iterations of GBD and expert review via the GBD collaborator network.^11^

### Summary burden measures

To compare disease burden across locations, GBD 2019 computed three summary measures. YLLs were calculated as the difference between the age of death for peripheral artery disease and the maximum life expectancy across all locations observed in GBD 2019. YLDs were calculated as the product of the disability weights^21^ for symptomatic and asymptomatic peripheral artery disease and the corresponding prevalence; information on disability weights for these two health states is in the appendix (p 10). DALYs are calculated as the sum of YLLs and YLDs to provide a comprehensive picture of the disease burden due to each cause. Age-standardised rates per 100 000 population were computed by the direct method to the GBD population standard.

### Risk factors

The GBD comparative risk assessment framework was used to estimate the burden of peripheral artery disease attributable to six risk factors: smoking, high fasting plasma glucose, high blood pressure, kidney dysfunction, high sodium intake, and lead exposure. These risk factors were selected based on the following criteria: sufficient evidence for causation for each risk-outcome pair using the Bradford Hill criteria; availability of risk exposure data; and potential for risk modification and policy relevance. Peripheral artery disease-related attributable burden was estimated by age, sex, country, and year. Further information about the methodology is in the appendix (pp 12–50) and in previous GBD publications.^11^

### Role of the funding source

The funder of this study had no role in study design, data collection, data analysis, data interpretation, or writing of the report.

## Results

### Global burden of peripheral artery disease

**Figure 1:**
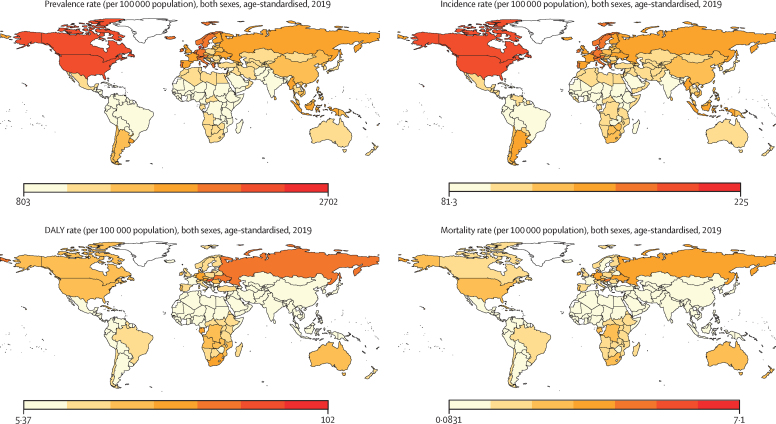
Geographical distribution of age-standardised rates of peripheral artery disease in 2019 DALY=disability-adjusted life-year.

**Figure 2:**
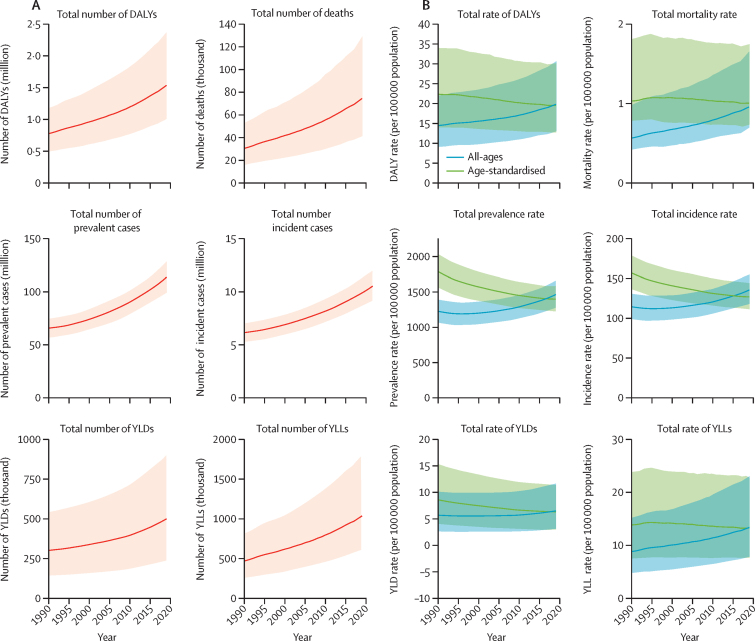
Total numbers and rates of peripheral artery disease, 1990–2019 (A) Total number of DALYs, deaths, prevalent cases, incident cases, YLDs, and YLLs of peripheral artery disease at the global level. (B) Rates (per 100 000 population) of age-standardised and all-age DALYs, deaths, prevalent cases, incident cases, YLDs, and YLLs of peripheral artery disease at the global level. Shaded regions indicate 95% uncertainty intervals. DALY=disability-adjusted life-year. YLD=year lived with disability. YLL=year of life lost.

The age-standardised DALYs, mortality, prevalence, and incidence rates of peripheral artery disease at the country level are shown in figure 1. In 2019, the global prevalence of peripheral artery disease was 1·52% (95% UI 1·33–1·72), of which 42·61% was in countries with low to middle Socio-demographic Index (SDI); the global prevalence of peripheral artery disease was substantially higher in females (2·03% [1·77–2·30]) than in males (1·01% [0·88–1·16]) from 1990 to 2019 (table). The global prevalence of peripheral artery disease was higher in older people (14·91% [12·41–17·87] in those aged 80–84 years), and the prevalence diverged by sex (18·03% [15·01–21·63] in females and 10·56% [8·78–12·76] in males). Globally, the total number of patients with peripheral artery disease almost doubled from 65·8 million (95% UI 57·2–74·5) in 1990 to 113 million (99·2–128·4) in 2019 (appendix pp 140–151, figure 2A). However, global age-standardised prevalence rates decreased during the study period, from 1790 per 100 000 population (95% UI 1564–2033) in 1990 to 1402 per 100 000 population (1229–1589) in 2019, a 21·7% (95% UI 20·5–22·8) decrease (appendix pp 140–151, figure 2B). Similarly, the total number of DALYs increased two-fold from 0·776 million (95% UI 0·488–1·178) in 1990 to 1·536 million (1·007–2·370) in 2019, whereas age-standardised DALY rates decreased from 22·4 per 100 000 population (95% UI 14·1–34·1) in 1990 to 19·6 per 100 000 population (12·9–30·2) in 2019 (appendix pp 152–163, figure 2).

**Table:** Estimated age-specific prevalence of peripheral artery disease, by sex and overall, in HICs, LMICs, and globally, 2019

	**Prevalence in females, % (95% UI)**			**Prevalence in males, % (95% UI)**			**Overall prevalence, % (95% UI)**		
	HICs	LMICs	Global	HICs	LMICs	Global	HICs	LMICs	Global
All ages (≥40 years)	4·48% (3·94–5·03)	1·01% (0·87–1·15)	2·03% (1·77–2·30)	2·73% (2·39–3·08)	0·57% (0·49–0·65)	1·01% (0·88–1·16)	3·62% (3·18–4·07)	0·79% (0·68–0·90)	1·52% (1·33–1·72)
40–44 years	1·23% (0·95–1·55)	0·43% (0·32–0·57)	0·62% (0·47–0·81)	0·49% (0·37–0·63)	0·30% (0·22–0·40)	0·34% (0·25–0·45)	0·85% (0·66–1·09)	0·37% (0·27–0·49)	0·48% (0·36–0·63)
45–49 years	2·08% (1·69–2·49)	0·99% (0·76–1·25)	1·36% (1·08–1·68)	1·01% (0·81–1·24)	0·66% (0·51–0·83)	0·73% (0·57–0·91)	1·54% (1·26–1·85)	0·82% (0·64–1·03)	1·05% (0·82–1·29)
50–54 years	2·98% (2·40–3·64)	1·84% (1·40–2·37)	2·45% (1·89–3·11)	1·83% (1·45–2·26)	1·19% (0·91–1·52)	1·32% (1·02–1·67)	2·41% (1·93–2·95)	1·51% (1·16–1·95)	1·89% (1·46–2·39)
55–59 years	4·11% (3·36–4·87)	2·95% (2·31–3·58)	3·75% (2·97–4·52)	3·11% (2·54–3·67)	1·88% (1·48–2·28)	2·14% (1·71–2·58)	3·62% (2·97–4·26)	2·42% (1·90–2·94)	2·96% (2·35–3·56)
60–64 years	5·75% (4·71–6·87)	4·29% (3·37–5·27)	5·38% (4·26–6·52)	4·82% (3·92–5·82)	2·68% (2·12–3·30)	3·17% (2·54–3·87)	5·30% (4·35–6·35)	3·50% (2·76–4·31)	4·30% (3·44–5·22)
65–69 years	8·99% (7·47–10·62)	6·27% (5·09–7·66)	8·02% (6·59–9·65)	7·3% (6·05–8·66)	3·81% (3·1–4·65)	4·64% (3·81–5·62)	8·19% (6·8–9·73)	5·08% (4·13–6·19)	6·4% (5·24–7·72)
70–74 years	13·38% (10·70–16·30)	8·77% (6·82–10·89)	11·32% (8·95–13·89)	10·28% (8·26–12·49)	5·23% (4·08–6·49)	6·59% (5·23–8·06)	11·93% (9·56–14·54)	7·07% (5·50–8·78)	9·09% (7·21–11·15)
75–79 years	17·64% (14·58–21·1)	11·38% (9·14–13·79)	14·64% (11·88–17·66)	12·96% (10·67–15·41)	6·77% (5·47–8·21)	8·58% (6·98–10·29)	15·55% (12·84–18·60)	9·27% (7·45–11·27)	11·91% (9·67–14·37)
80–84 years	21·55% (18·08–25·55)	14·01% (11·48–17·02)	18·03% (15·01–21·63)	15·38% (12·96–18·30)	8·37% (6·84–10·27)	10·56% (8·78–12·76)	19·00% (16·03–22·55)	11·59% (9·51–14·10)	14·91% (12·41–17·87)
85–89 years	24·60% (20·81–29·02)	15·82% (13·08–18·84)	20·83% (17·44–24·73)	17·32% (14·67–20·36)	9·84% (8·13–11·77)	12·72% (10·69–15·05)	21·93% (18·56–25·80)	13·29% (11·02–15·85)	17·79% (14·90–21·12)
90–94 years	26·80% (22·94–31·05)	17·38% (14·58–20·58)	23·24% (19·78–27·13)	18·91% (16·21–21·95)	11·21% (9·21–13·40)	15·16% (12·80–17·76)	24·36% (20·89–28·19)	14·84% (12·36–17·60)	20·70% (17·58–24·18)

UI=uncertainty interval. HIC=high-income country. LMIC=lower-middle-income country.

**Figure 3:**
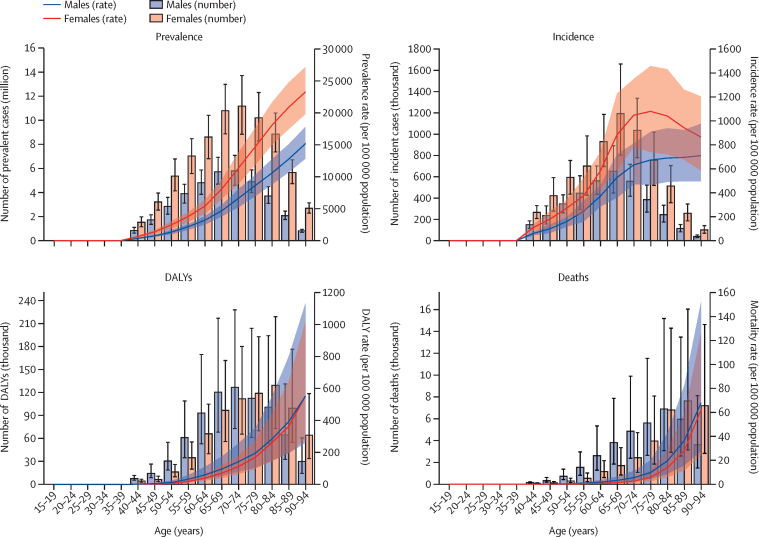
Numbers and age-standardised rates (per 100 000 population) of prevalence, incidence, DALYs, and deaths from peripheral artery disease at the global level by age group and sex, 2019 Error bars and shaded regions indicate 95% uncertainty intervals. DALY=disability-adjusted life-year.

Age-specific prevalence peaked at age 70–74 years and age-specific incidence peaked at age 65-69 years (figure 3). The highest number of DALYs occurred at age 70–74 years for males, whereas the distribution was skewed towards older age for females, with the highest number of DALYs at age 80–84 years (appendix pp 336–37).

### Burden of peripheral artery disease according to SDI

The disease burden of peripheral artery disease was associated with SDI level (appendix p 135). The age-standardised prevalence rates of peripheral artery disease increased with increasing SDI quintile, with the high SDI quintile having the highest prevalence rate (1794 per 100 000 population [95% UI 1585–2006]) and the low SDI quintile having the lowest prevalence rate (938·6 per 100 000 population [815·0–1074·6]) in 2019 (appendix pp 140–151). Furthermore, from 1990 to 2019, higher SDI quintiles underwent a steep decrease in peripheral artery disease age-standardised prevalence rates, with a decrease of 34·0% (95% UI 32·0–35·8) in the high SDI quintile and of 15·4% (14·2–16·4) in the high-middle SDI quintiles from 1990 to 2019, whereas in lower SDI quintiles, the rates remained stable (appendix p 135).

**Figure 4:**
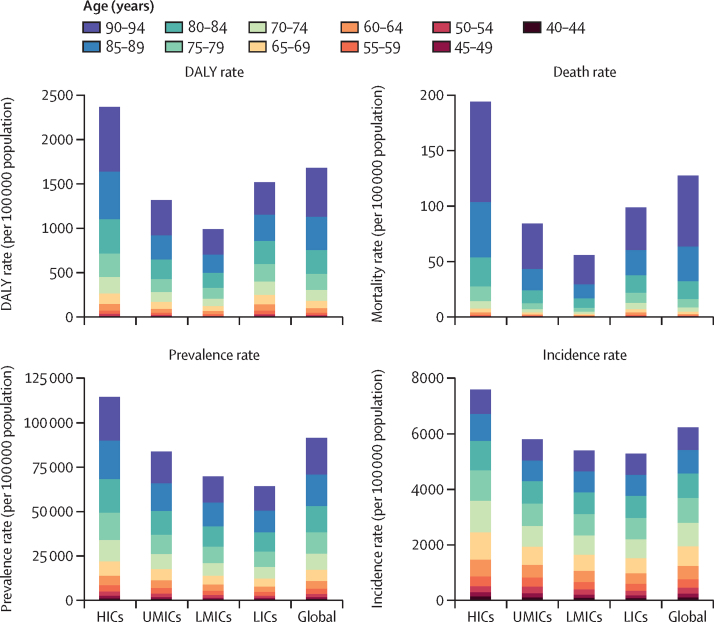
DALYs, mortality, prevalence, and incidence rates (per 100 000 persons) of peripheral artery disease for both sexes at the World Bank income level, by age group, 2019 DALY=disability-adjusted life-year. HIC=high-income country. UMIC=upper-middle-income country. LMIC=lower-middle-income country. LIC=low-income country.

Higher SDI quintiles tended to have higher DALY and mortality rates, with the high and high-middle quintiles having the highest DALY and mortality rates across the study period (1·5 peripheral artery disease deaths per 100 000 population [95% UI 0·7–2·8] in the high SDI quintile and 1·4 per 100 000 population [0·7–2·4] in the high-middle SDI quintile in 2019) and the middle and low-middle SDI quintiles having the lowest rates (0·4 peripheral artery disease deaths per 100 000 population [0·3–0·5] for both middle and low-middle quintiles in 2019). However, the low SDI quintile was in the middle, with DALY and mortality rates higher than the low-middle and middle quintiles and lower than the high and high-middle quintiles (0·7 peripheral artery disease deaths per 100 000 population [0·4–1·0] in 2019; appendix pp 164–175). This trend was replicated with the analysis using the World Bank income level; prevalence and incidence rates of peripheral artery disease increased stepwise with increasing income level, whereas DALY and mortality rates of peripheral artery disease were U-shaped by income level (figure 4).

### Risk factors

**Figure 5:**
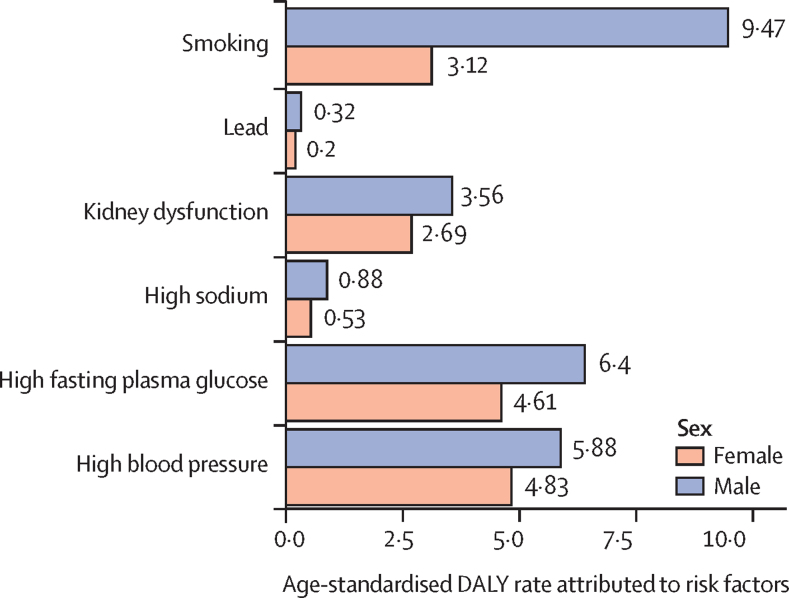
Age-standardised DALYs rate (per 100 000 persons) of peripheral artery disease attributed to risk factors in 2019

The total number of peripheral artery disease DALYs globally attributable to all estimated risk factors in 2019 was 1·066 million (95% UI 0·690–1·646) for both sexes combined, which accounted for 69·4% (95% UI 64·2–74·3) of all peripheral artery disease DALYs. Males were estimated to have 0·589 million (0·333–1·072) peripheral artery disease DALYs attributable to risk factors, or 76·9% (72·7–80·4) of all peripheral artery disease DALYs in males, whereas females were estimated to have 0·477 million (0·285–0·760) peripheral artery disease DALYs attributable to risk factors, or 62·0% (57·0–67·0) of all peripheral artery disease DALYs in females. In males, the age-standardised DALY rate for peripheral artery disease attributed to smoking was 9·5 (5·1–17·2), to high fasting plasma glucose was 6·4 (3·5–12·0), to high blood pressure was 5·9 (3·2–10·8), to kidney dysfunction was 3·6 (1·9–6·8), to high sodium was 0·9 (0·2–2·4), and to lead was 0·3 (0·2–0·6; figure 5; appendix p 218). In females, the age-standardised DALY rate for peripheral artery disease attributed to smoking was 3·1 (1·7–5·4), to high fasting plasma glucose was 4·6 (2·7–7·6), to high blood pressure was 4·8 (2·7–8·3), to kidney dysfunction was 2·7 (1·5–4·4), to high sodium was 0·5 (0·1–1·5), and to lead was 0·2 (0·1–0·4; figure 5).

## Discussion

We assessed the global burden of peripheral artery disease using estimates from GBD 2019. The total number of people with peripheral artery disease has increased from 1990 to 2019, which is likely to be due to population growth and ageing, considering that age-standardised rates have not increased. Indeed, age-standardised estimates for peripheral artery disease were stable or decreased, depending on summary measures, possibly indicating better prevention and management of peripheral artery disease over time. Modifiable risk factors were responsible for around 70% of the global peripheral artery disease burden, which highlights the extent to which public health measures could mitigate the burden of peripheral artery disease by modifying risk factors. Increased SDI was associated with an increased burden of peripheral artery disease, probably due to greater metabolic pressures (ie, high blood pressure and plasma glucose) in high-income nations (appendix p 139).

In 2019, the global prevalence of peripheral artery disease was 14·91% (95% UI 12·41–17·87) in those aged 80–84 years, and the prevalence diverged by sex (18·03% [15·01–21·63] in females and 10·56% [8·78–12·76] in males). Our estimated prevalence of peripheral artery disease in this age group was similar to the latest systematic reviews by the Global Peripheral Artery Disease Study,^10^ in which they reported a global peripheral artery disease prevalence of 15·69% at age 80–84 years. Moreover, we replicated the findings of previous studies^9^, ^10^ in that the prevalence of peripheral artery disease was higher in females than males, particularly in low-income and middle-income regions. Low pain threshold, more common leg symptoms,^22^, ^23^ and a greater tendency to seek medical care in women^24^ compared with men might have facilitated a higher prevalence in women. Sex hormone mechanisms in cardiovascular diseases and atherosclerosis could be another reason.^25^, ^26^, ^27^ Menopause is associated with an increased risk of peripheral artery disease,^28^ and it could partially explain a gradual increase in the difference in prevalence between sex after menopausal age. The paradoxical situation of peripheral artery disease being more prevalent in females, despite the higher prevalence of major risk factors (ie, smoking, hypertension, diabetes) in males, has been a longstanding question.^10^, ^29^ Our study showed that despite the higher prevalence in females, the overall peripheral artery disease burden measured by DALYs was similar in both sexes, and the total DALYs attributable to modifiable risk factors was actually higher in males than females (76·9% [95% UI 72·7–80·4] *vs* 62·0% [57·0–67·0]). These data suggest that males experience more disability due to peripheral artery disease than females, which could be attributed to diagnosis of peripheral artery disease at more advanced stages or greater risk of complications. These observations indicate that a disproportionate burden is placed on males and equivalent attention and gender-specific care for peripheral artery disease is warranted.

Atherosclerotic diseases have been traditionally thought to be diseases of wealthy countries, consistent with the concept of epidemiological transition; however, the burden of cardiovascular disease has been rapidly increasing in many low-income and middle-income countries, which can be explained in part by changes in lifestyle and environment due to development and urbanisation.^8^, ^30^, ^31^ In our study, age-standardised prevalence rates of peripheral artery disease were increased stepwise with higher SDI and World Bank income levels. By contrast, DALY and mortality rates of peripheral artery disease showed U-shape patterns, wherein countries with the highest SDI and income levels reported the highest DALY and mortality rates, countries with the middle SDI and income levels reported the lowest DALY and mortality rates, and the countries with the lowest SDI and income level had moderate values. This pattern might suggest that countries with low SDI and income had a disease burden that was disproportionate to prevalence, implying that peripheral artery disease burden has not been adequately managed in these countries. Considering the lower prevalence of peripheral artery disease in low-SDI and low-income countries, mild or asymptomatic peripheral artery disease might simply be underdiagnosed due to lack of resources;^32^ furthermore, factors associated with low socio-developmental status, such as insufficient access to care, lack of quality in care, and conditions that are suboptimal for lifestyle modifications, might have facilitated worse disease burden and mortality. Therefore, prevention, early diagnosis, and apt management of peripheral artery disease in low-resource settings must be highlighted in the global community, and system-level disparities in managing cardiovascular risk factors should be explored.

Several strengths and novel aspects of this study should be highlighted. First, this study provided estimates of peripheral artery disease from diverse health metrics. Previous global estimate studies by Fowkes and colleagues^9^ and Song and colleagues^10^ assessed only the global prevalence of peripheral artery disease, whereas this study presented prevalence, cause-specific mortality, and DALYs. Because peripheral artery disease can result in lifelong disability, cross-sectional prevalence might not provide a complete picture of the disease burden. DALYs are a more appropriate health measure for peripheral artery disease, because they express the number of years lost due to ill health, disability, or early death, and are commonly used to quantify the impact of diseases or health conditions on a population.^11^ Second, by integrating the DALY metric, we were able to estimate the disease burden of peripheral artery disease attributable to risk factors using an approach demonstrated previously.^33^ Our analysis showed that approximately 69% of the peripheral artery disease burden could be avoided by making modifications to these risk factors. This approach offered a unique perspective on associations between risk factors and peripheral artery disease from previous global peripheral artery disease studies^9^, ^10^ that have provided only odds ratios for the risk of peripheral artery disease with a given risk factor. Absolute risk more often represents the actual burden and enriches decision-making compared with relative risk.^34^ Third, we used search strategies to obtain as much prevalence data of peripheral artery disease as possible, while ensuring data quality and comparability. To this end, we included only studies that confirmed the presence of peripheral artery disease based on an ABI value of less than or equal to 0·9, regardless of intermittent claudication, complying with the strategies used in previous studies.^9^, ^10^ From clinical and public-health perspectives, this approach is meaningful because asymptomatic peripheral artery disease is also associated with an increased risk of cardiovascular morbidity and mortality,^10^, ^35^ and such potential burdens deserve attention and action. Of note, ABI could be falsely elevated in a subset of patients, such as those with diabetes, and the values should be interpreted with caution.^36^

Although it benefits from a global dataset with country-level and region-level data, the present study has several limitations. First, this study includes the inherent limitations of the GBD dataset, mainly the data missingness and low availability and quality of data in some regions. For these data-sparse regions, descriptive statistics had to rely on predictive modelling using covariates, and inferences might not be as accurate as for other regions.^11^ Second, this was an ecological study based on country-level aggregate data; associations with SDI and income might not reflect individual patient-level associations. Third, this analysis could have underestimated the prevalence of peripheral artery disease burden due to under-ascertainment because some proportion of the population might not have sought medical care or could have escaped the epidemiological assessment, especially in low-resource settings. Fourth, our analysis incorporated six specific risk factors and could not account for some emerging risk factors, such as lipoprotein levels. This limitation arose primarily from insufficient risk exposure data to generate accurate estimates. However, the risk-outcome pair matrix is constantly being updated to reflect changes in the available scientific evidence, and our future iterations of the peripheral artery disease GBD study will include more risk factors. The interactions between risk factors and their impact on peripheral artery disease burden should also be explored in future iterations. Fifth, our estimates for the burden of peripheral artery disease could be underestimated because we might not have fully captured the burden of acute limb ischaemia and chronic limb-threatening ischaemia.^37^ Nevertheless, the global estimate of 113 million peripheral artery disease cases is already a large burden, and understanding its contribution at global, regional, and national levels is a current research priority.^11^ Future studies would benefit from including acute limb ischaemia and chronic limb-threatening ischaemia for a more comprehensive landscape of global peripheral artery disease burden. Lastly, although ethnicity could be an important factor to consider, we did not include it because the main goal of the GBD study is to provide descriptive estimates by geographic location.

Total prevalence, mortality, and DALYs of peripheral artery disease have increased worldwide from 1990 to 2019 while their age-standardised rates have decreased, which is likely to reflect a transition in population structure (ie, population growth and ageing). The evidence from this GBD study indicates that a substantial proportion of the global peripheral artery disease burden could be prevented through collective efforts to address modifiable risk factors.

### Data sharing

This paper summarises key findings from our analysis of GBD 2019 estimates. All estimates are publicly available in our online tools (http://ghdx.healthdata.org/gbd-2019). Citations for the data used in this study can be accessed from the Global Health Data Exchange data input sources tool (http://ghdx.healthdata.org/gbd-2019/data-input-sources). Files containing all GBD 2019 estimates are available on the Global Health Data Exchange website (http://ghdx.healthdata.org/gbd-2019) and can also be downloaded from the Global Health Data Exchange results tool (http://healthdata.org/gbd-results-tool).

## Declaration of interests

S W Lee reports support for the present manuscript from a National Research Foundation grant funded by the Ministry of Education, South Korea (NRF2021R1I1A2059735). S Bhaskar reports leadership or fiduciary roles in board, society, committee, or advocacy groups, paid or unpaid with Rotary District 9675 as a chair of Diversity, Equity, and Inclusion; the Rotary Club of Sydney as a board director and chair of Youth, Global Health and Migration; and the Global Health Hub Germany as a founding member and chair; all outside the submitted work. K Krishan reports other non-financial support from the UGC Centre of Advanced Study, CAS II, Department of Anthropology, Panjab University, Chandigarh, India; all outside the submitted work. S Lorkowski reports grants or contracts from Akcea Therapeutics Germany; consulting fees from Danone, Novartis Pharma, Swedish Orphan Biovitrum, and Upfield; payment or honoraria for lectures, presentations, speakers bureaus, manuscript writing, or educational events from Akcea Therapeutics Germany, AMARIN Germany, Amedes Holding, Amgen, Berlin-Chemie, Boehringer Ingelheim Pharma, Daiichi Sankyo Deutschland, Danone, Hubert Burda Media Holding, Janssen-Cilag, Lilly Deutschland, Novartis Pharma, Novo Nordisk Pharma, Roche Pharma, Sanofi-Aventis, SYNLAB Holding Deutschland, and SYNLAB Akademie; support for attending meetings or travel from Amgen; participation on a data safety monitoring board or advisory board with Akcea Therapeutics Germany, Amgen, Daiichi Sankyo Deutschland, Novartis Pharma, and Sanofi-Aventis; all outside the submitted work. A-F A Mentis reports grants or contracts from MilkSafe (a novel pipeline to enrich formula milk using omics technologies), research co-financed by the European Regional Development Fund of the EU and Greek national funds through the Operational Program Competitiveness, Entrepreneurship and Innovation, under the call RESEARCH–CREATE–INNOVATE (project code T2EDK-02222), as well as from ELIDEK (Hellenic Foundation for Research and Innovation, MIMS-860); payment for expert testimony from serving as an external peer reviewer for Fondazione Cariplo, Italy; leadership or fiduciary roles in board, society, committee, or advocacy groups, paid or unpaid as an editorial board member for *Systemic Reviews* and *Annals of Epidemiology*, and as associate editor for *Translational Psychiatry*; stocks in a family winery; other financial or non-financial support from the BGI group as a scientific officer; outside the submitted work. A Radfar reports support for the present manuscript from Avicenna Medical and Clinical Research Institute. J A Singh reports consulting fees from Crealta/Horizon, Medisys, Fidia, PK Med, Two Labs, Adept Field Solutions, Clinical Care Options, Clearview Healthcare Partners, Putnam Associates, Focus Forward, Navigant Consulting, Spherix, MedIQ, Jupiter Life Science, UBM, Trio Health, Medscape, WebMD, and Practice Point Communications, and the National Institutes of Health and the American College of Rheumatology; payment or honoraria for lectures, presentations, speakers bureaus, manuscript writing, or educational events from the speakers bureau of Simply Speaking; support for attending meetings or travel from the steering committee of OMERACT; participation on a data safety monitoring board or advisory board as a member of the FDA Arthritis Advisory Committee; leadership or fiduciary roles in board, society, committee, or advocacy groups, paid or unpaid as a steering committee member of OMERACT, with the Veteran Affairs Rheumatology Field Advisory Committee as a chair, and with the UAB Cochrane Musculoskeletal Group Satellite Center on Network Meta-analysis as an editor; stock or stock options in Atai Life Sciences, Kintara Therapeutics, Intelligent Biosolutions, Acumen Pharmaceutical, TPT Global Tech, Vaxart Pharmaceuticals, Atyu Biopharma, Adaptimmune Therapeutics, GeoVax Labs, Pieris Pharmaceuticals, Enzolytics, Seres Therapeutics, Tonix Pharmaceuticals and Charlotte's Web, and previously owned stock options in Amarin, Viking, and Moderna Pharmaceuticals; all outside the submitted work. J Sundström reports stock or stock options as a shareholder in Anagram Kommunication and Symptoms Europe, outside the submitted work. All other authors declare no competing interests.

## References

[1] Gerhard-Herman MD, Gornik HL, Barrett C (2017). 2016 AHA/ACC guideline on the management of patients with lower extremity peripheral artery disease: a report of the American College of Cardiology/American Heart Association Task Force on Clinical Practice Guidelines. J Am Coll Cardiol.

[2] Kullo IJ, Rooke TW (2016). Clinical practice. Peripheral artery disease. N Engl J Med.

[3] Creager MA (2020). A bon VOYAGER for peripheral artery disease. N Engl J Med.

[4] Berger JS, Ladapo JA (2017). Underuse of prevention and lifestyle counseling in patients with peripheral artery disease. J Am Coll Cardiol.

[5] Fowkes FGR, Aboyans V, Fowkes FJ, McDermott MM, Sampson UK, Criqui MH (2017). Peripheral artery disease: epidemiology and global perspectives. Nat Rev Cardiol.

[6] Criqui MH, Denenberg JO, Langer RD, Fronek A (1997). The epidemiology of peripheral arterial disease: importance of identifying the population at risk. Vasc Med.

[7] Fowkes FG (1988). Epidemiology of atherosclerotic arterial disease in the lower limbs. Eur J Vasc Surg.

[8] Yusuf S, Reddy S, Ôunpuu S, Anand S (2001). Global burden of cardiovascular diseases: part I: general considerations, the epidemiologic transition, risk factors, and impact of urbanization. Circulation.

[9] Fowkes FGR, Rudan D, Rudan I (2013). Comparison of global estimates of prevalence and risk factors for peripheral artery disease in 2000 and 2010: a systematic review and analysis. Lancet.

[10] Song P, Rudan D, Zhu Y (2019). Global, regional, and national prevalence and risk factors for peripheral artery disease in 2015: an updated systematic review and analysis. Lancet Glob Health.

[11] Vos T, Lim SS, Abbafati C (2020). Global burden of 369 diseases and injuries in 204 countries and territories, 1990–2019: a systematic analysis for the Global Burden of Disease Study 2019. Lancet.

[12] Dicker D, Nguyen G, Abate D (2018). Global, regional, and national age-sex-specific mortality and life expectancy, 1950–2017: a systematic analysis for the Global Burden of Disease Study 2017. Lancet.

[13] Afshin A, Sur PJ, Fay KA (2019). Health effects of dietary risks in 195 countries, 1990–2017: a systematic analysis for the Global Burden of Disease Study 2017. Lancet.

[14] James SL, Abate D, Abate KH (2018). Global, regional, and national incidence, prevalence, and years lived with disability for 354 diseases and injuries for 195 countries and territories, 1990–2017: a systematic analysis for the Global Burden of Disease Study 2017. Lancet.

[15] Roth GA, Abate D, Abate KH (2018). Global, regional, and national age-sex-specific mortality for 282 causes of death in 195 countries and territories, 1980–2017: a systematic analysis for the Global Burden of Disease Study 2017. Lancet.

[16] Foreman KJ, Lozano R, Lopez AD, Murray CJ (2012). Modeling causes of death: an integrated approach using CODEm. Popul Health Metr.

[17] Patel SK, Surowiec SM (2023). StatPearls.

[18] Bikbov B, Purcell CA, Levey AS (2020). Global, regional, and national burden of chronic kidney disease, 1990–2017: a systematic analysis for the Global Burden of Disease Study 2017. Lancet.

[19] Haile LM, Kamenov K, Briant PS (2021). Hearing loss prevalence and years lived with disability, 1990–2019: findings from the Global Burden of Disease Study 2019. Lancet.

[20] Degenhardt L, Charlson F, Ferrari A (2018). The global burden of disease attributable to alcohol and drug use in 195 countries and territories, 1990–2016: a systematic analysis for the Global Burden of Disease Study 2016. Lancet Psychiatry.

[21] Salomon JA, Haagsma JA, Davis A (2015). Disability weights for the Global Burden of Disease 2013 study. Lancet Glob Health.

[22] Schramm K, Rochon PJ (2018). Gender differences in peripheral vascular disease. Semin Intervent Radiol.

[23] McDermott MM, Greenland P, Liu K (2003). Sex differences in peripheral arterial disease: leg symptoms and physical functioning. J Am Geriatr Soc.

[24] Thompson AE, Anisimowicz Y, Miedema B, Hogg W, Wodchis WP, Aubrey-Bassler K (2016). The influence of gender and other patient characteristics on health care-seeking behaviour: a QUALICOPC study. BMC Fam Pract.

[25] Vogel B, Acevedo M, Appelman Y (2021). The *Lancet* women and cardiovascular disease Commission: reducing the global burden by 2030. Lancet.

[26] Zhao D, Guallar E, Ouyang P (2018). Endogenous sex hormones and incident cardiovascular disease in post-menopausal women. J Am Coll Cardiol.

[27] Man JJ, Beckman JA, Jaffe IZ (2020). Sex as a biological variable in atherosclerosis. Circ Res.

[28] Honigberg MC, Zekavat SM, Aragam K (2019). Association of premature natural and surgical menopause with incident cardiovascular disease. JAMA.

[29] Murray CJ, Aravkin AY, Zheng P (2020). Global burden of 87 risk factors in 204 countries and territories, 1990–2019: a systematic analysis for the Global Burden of Disease Study 2019. Lancet.

[30] Omran AR (2001). The epidemiologic transition. A theory of the epidemiology of population change. 1971. Bull World Health Organ.

[31] Gaziano TA, Bitton A, Anand S, Abrahams-Gessel S, Murphy A (2010). Growing epidemic of coronary heart disease in low- and middle-income countries. Curr Probl Cardiol.

[32] Kengne AP, Echouffo-Tcheugui JB (2019). Differential burden of peripheral artery disease. Lancet Glob Health.

[33] Tran KB, Lang JJ, Compton K (2022). The global burden of cancer attributable to risk factors, 2010–19: a systematic analysis for the Global Burden of Disease Study 2019. Lancet.

[34] Noordzij M, van Diepen M, Caskey FC, Jager KJ (2017). Relative risk versus absolute risk: one cannot be interpreted without the other. Nephrol Dial Transplant.

[35] Golomb BA, Dang TT, Criqui MH (2006). Peripheral arterial disease: morbidity and mortality implications. Circulation.

[36] Potier L, Abi Khalil C, Mohammedi K, Roussel R (2011). Use and utility of ankle brachial index in patients with diabetes. Eur J Vasc Endovasc Surg.

[37] Hess CN, Huang Z, Patel MR (2019). Acute limb ischemia in peripheral artery disease. Circulation.

