# Supplementary material for: Global burden of peripheral artery disease and its risk factors, 1990–2019: a systematic analysis for the Global Burden of Disease Study 2019
Source: Lancet Glob Health. 2023 Sep 19;11(10):e1553–65. doi: 10.1016/S2214-109X(23)00355-8 (PMC10522777; doi:10.1016/S2214-109X(23)00355-8)
Supplement: Supplementary appendix [file mmc1.pdf]

THE LANCET
Global Health

Supplementary appendix

This appendix formed part of the original submission and has been peer reviewed. We post it as supplied by the authors.

Supplement to: GBD 2019 Peripheral Artery Disease Collaborators. Global burden of peripheral artery disease and its risk factors, 1990–2019: a systematic analysis for the Global Burden of Disease Study 2019. *Lancet Glob Health* 2023; **11:** e1553–65.

Supplementary Appendix

Global burden of peripheral artery diseases and its risk factors, 1990-2019: a systematic analysis for the Global Burden of Disease Study 2019

This appendix provides further methodological detail for "Global burden of peripheral artery diseases and its risk factors, 1990-2019: a systematic analysis for the Global Burden of Disease Study 2019".

Portions of this appendix methods 1–5 have been reproduced or adapted from Roth et al.,[1] James et al.,[2] Kyu et al.,[3] Stanaway et al.,[4] and GBD 2019 capstone papers. References are provided for reproduced sections.

Contents

I. Supplementary GBD methodology pp 2-133

II. Supplementary figures and tables pp 134-330:
a. Figure S1. Age-standardised DALYs, deaths, prevalent cases, incident cases, YLDs, YLLs rates (per 100 000 persons) of PAD at the SDI region level.
b. Figure S2. Age-standardised rate (per 100 000 persons) of DALYs, mortality, prevalence, and incidence of PAD at the SDI region level.
c. Figure S3. Correlation between SDI and age-standardised DALYs (per 100 000 persons) of PAD at the country level, 2019. The solid line denotes the linear trend between the two variables. Outlier countries are not shown.
d. Figure S4. Numbers and age-standardized rates (per 100 000 persons) of PAD prevalence, incidence, DALYs, and deaths at the global level, 1990 to 2019. Error bars and shaded regions indicate 95% uncertainty intervals.
e. Figure S5. Age-standardised DALY rates (per 100 000 persons) of PAD attributable to risk factors by SDI regions and sex.
f. Table S1: PAD prevalence in 1990 and 2019 for both sexes and percentage change in age-standardised rates by location.
g. Table S2. DALYs due to PAD in 1990 and 2019 for both sexes and percentage change in age-standardised rates by location.
h. Table S3: Deaths due to PAD in 1990 and 2019 for both sexes and percentage change in age-standardised rates by location.
i. Table S4: Incidence rates and total incident cases of PAD in 1990 and 2019 for both sexes and percentage change in age-standardised rates by location.
j. Table S5: YLDs of PAD in 1990 and 2019 for both sexes and percentage change in age-standardised rates by location.
k. Table S6: YLLs of PAD in 1990 and 2019 for both sexes and percentage change in age-standardised rates by location.
l. Table S7. DALYs rate (per 100 000 persons) of male and female PAD patient at world bank income level in 2019 by age group.
m. Table S8. Mortality rate (per 100 000 persons) of male and female PAD patient at world bank income level in 2019 by age group.
n. Table S9. Prevalence rate (per 100 000 persons) of male and female PAD patient at world bank income level in 2019 by age group.
o. Table S10. Incidence rate (per 100 000 persons) of male and female PAD patient at world bank income level in 2019 by age group.
p. Table S11. Age-standardised incidence rate due to PAD in 2019 for both sexes by SDI group.
q. Table S12. Age-standardised DALYs rate (per 100 000 persons) of PAD attributed to risk factors for males, females, and sex combined in 2019.
r. Table S13. Estimated age-specific prevalence (%) of women and men living with peripheral artery disease in 204 countries and territories in 2019.

III. Supplementary methods and results pp 331-338

IV. Authors' contributions pp 339-341

Section 1: Peripheral Artery Disease

Cause of death estimation for peripheral arterial disease

Flowchart

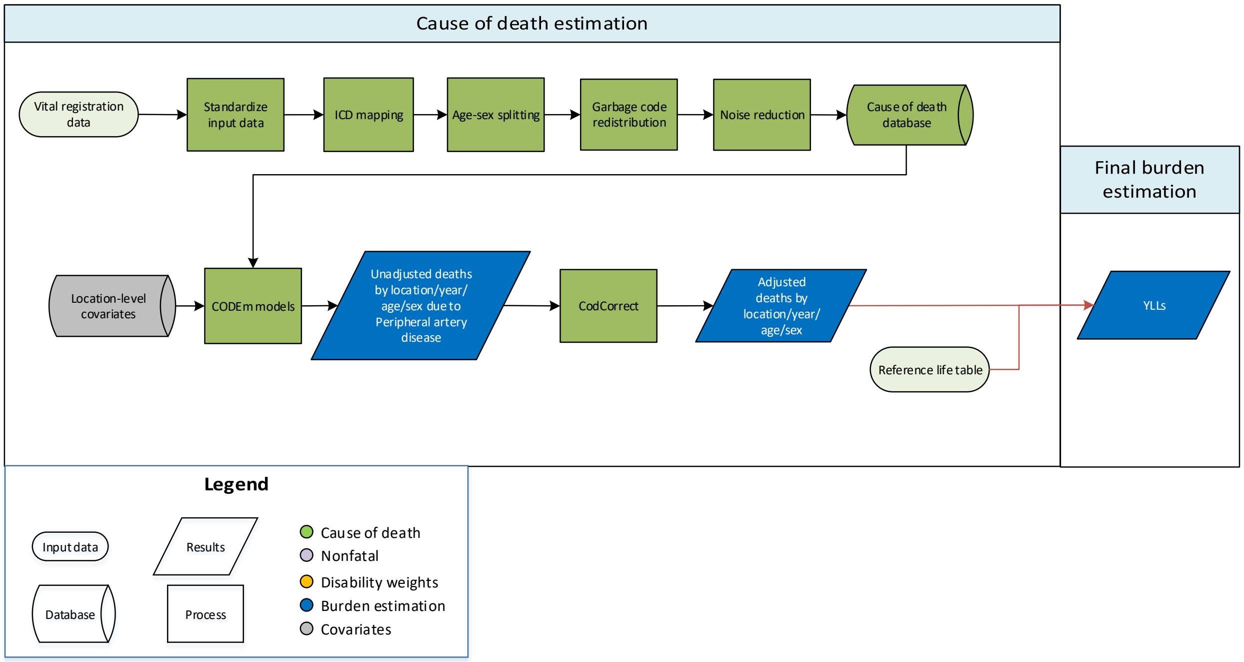

Input Data and Methodological Summary

Vital registration data were used to model peripheral artery disease. We outliered all datapoints with less than 1 death in Egypt per expert review.

Modelling strategy

We utilized the standard Cause of Death Ensemble Modeling (CODEm) approach to model deaths caused by peripheral artery disease. The covariates considered in the ensemble modeling process are enumerated in the table below. There have been no significant alterations made to the methodology utilized in GBD 2017. Further statistical details regarding CODEm can be found in a separate section of this appendix.

Table 1. Covariates used in peripheral artery disease mortality modeling.

Level	Covariate	Direction
1	Summary exposure variable, PAD	1
	Systolic blood pressure (mmHg)	1
	Cholesterol (total, mean per capita)	1
	Smoking prevalence	1
2	Mean body mass index (kg/m^2)	-1
	Healthcare access and quality index	1
	Diabetes fasting plasma glucose (mmol/L)	-1
3	Lag distributed income per capita (I$)	-1
	Socio-demographic Index	1
	Summary exposure value, omega-3	1
	Summary exposure value, fruits	1
	Summary exposure value, vegetables	1
	Summary exposure value, nuts and seeds	1

	Pulses/legumes (kcal/capita, unadjusted)	-1
	Summary exposure value, polyunsaturated fatty acids	1
	Alcohol (litres per capita)	1

Non-fatal estimation for peripheral arterial disease

Flowchart

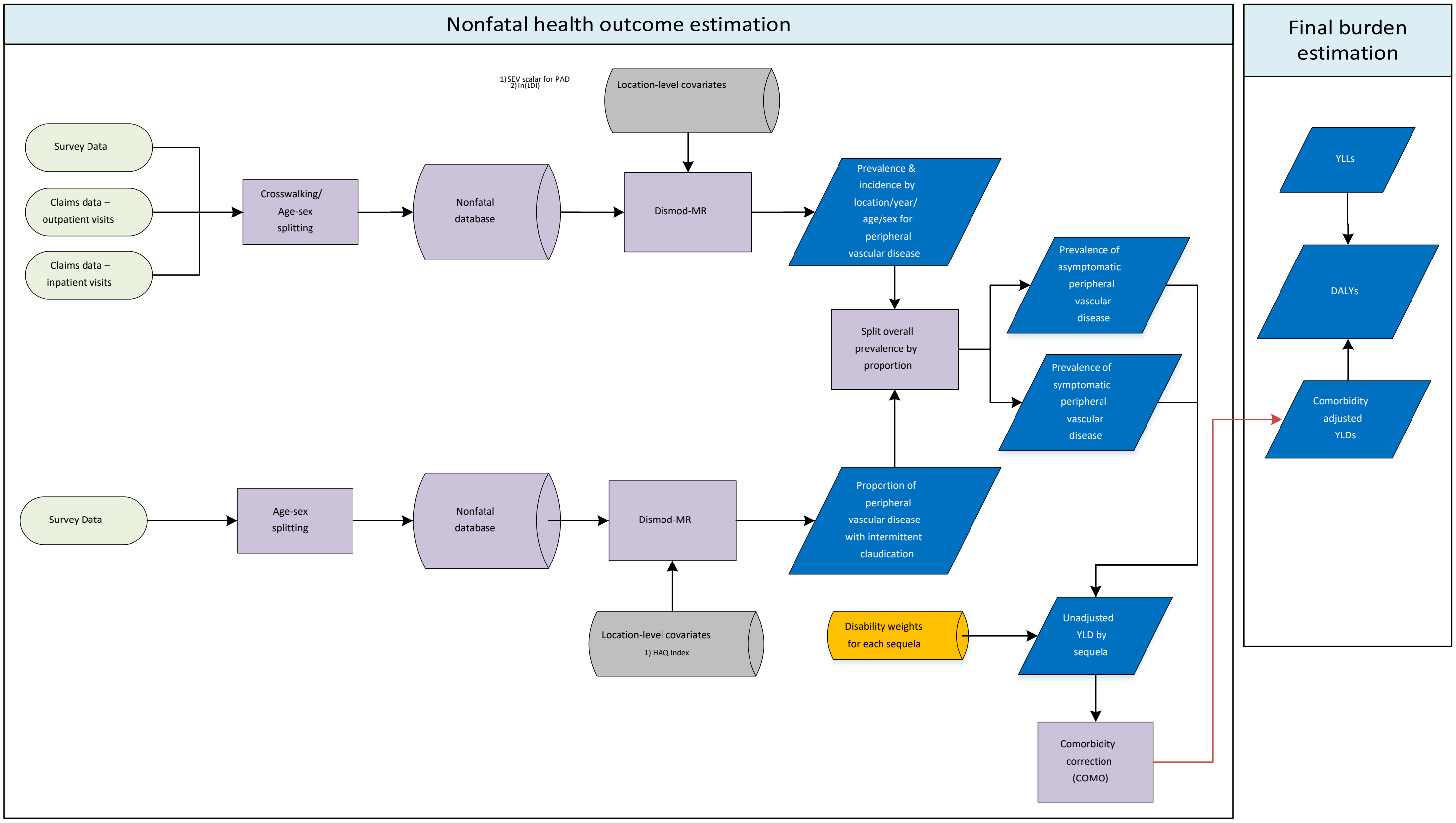

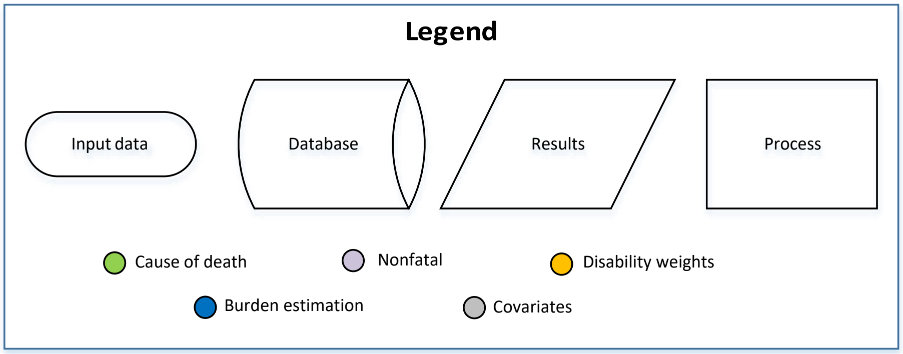

Input Data and Methodological Summary

Case definition

For GBD 2019, peripheral arterial disease was defined as having an ankle-brachial index (ABI) ≤ 0.9. Intermittent claudication was defined clinically as leg pain on exertion among those with an ABI below that threshold.

Table 1: Reference and alternate definitions of peripheral arterial disease

Quantity of interest	Reference or alternate	Definition
Prevalence of peripheral arterial disease	Reference	Persons with an ankle brachial index (ABI) ≤ 0.9. ABI is the ratio of systolic blood pressure measured at the ankle and the arm
Prevalence of peripheral arterial disease	Alternate	Peripheral arterial disease as identified in administrative claims, outpatient, or primary care data
Proportion of patients with peripheral arterial disease and intermittent claudication	Reference	Persons with an ankle brachial index (ABI) ≤ 0.9 who report pain due to claudication

Table 2: ICD codes for claims data included in GBD 2019 mapped to peripheral arterial disease

ICD Code	ICD Cause name
440.20, 440.21, 440.22, 440.23, 440.24, 440.29, 440.4, 440.8, 440.9	Atherosclerosis of native arteries of the extremities
443<br>443.1, 443.2, 443.8, 443.81, 443.82, 443.89,443.9	Other peripheral vascular disease
I70.2<br>I70.20, I70.201, I70.202, I70.203, I70.208, I70.209<br>I70.21, I70.211, I70.212, I70.213, I70.218, I70.219<br>I70.22, I70.221, I70.222,	Atherosclerosis of native arteries of the extremities<br>Unspecified atherosclerosis of native arteries of extremities<br>Atherosclerosis of native arteries of extremities with intermittent claudication

I70.223, I70.228, I70.229	Atherosclerosis of native arteries of extremities with rest pain
I70.23, I70.231, I70.232, I70.233, I70.234, I70.235, I70.238, I70.239	Atherosclerosis of native arteries of right leg with ulceration
I70.24, I70.241, I70.242, I70.243, I70.244, I70.245, I70.248, I70.249	Atherosclerosis of native arteries of left leg with ulceration
I70.25	Atherosclerosis of native arteries of other extremities with ulceration
I70.26, 70.261, I70.262, I70.263, I70.268, I70.269	Atherosclerosis of native arteries of extremities with gangrene
I70.29, I70.291, I70.292, I70.293, I70.298, I70.299	Other atherosclerosis of native arteries of extremities
I73, I73.1, I73.8, I73.81, I73.89, I73.9	Other peripheral vascular diseases

Input data

Table 3 shows the source counts for peripheral arterial disease modeling. We did not perform a systematic review for GBD 2019. A systematic review was performed for peripheral arterial disease and intermittent claudication for GBD 2015. The search terms were: ('peripheral vascular disease'[TIAB] AND 'epidemiology'[Subheading]) OR ('peripheral arterial disease'[TIAB] AND 'epidemiology'[Subheading]) OR ('peripheral artery disease'[TIAB] AND 'epidemiology'[Subheading]) OR ('intermittent claudication'[TIAB] AND 'epidemiology'[Subheading]) OR ('ankle-brachial index'[TIAB] AND 'epidemiology'[Subheading]) OR ('ankle brachial index'[TIAB] AND 'epidemiology'[Subheading]) OR ('peripheral artery occlusive disease'[TIAB] AND 'epidemiology'[Subheading]) OR ('peripheral obliterative arteriopathy'[TIAB] AND 'epidemiology'[Subheading]) OR ('peripheral vascular disease'[TIAB] AND 'prevalence'[MeSH Terms]) OR ('peripheral vascular disease'[TIAB] AND 'incidence'[MeSH Terms]) OR ('peripheral vascular disease'[TIAB] AND 'case fatality'[All Fields]) OR ('symptomatic claudication'[TIAB] AND (proportion[All Fields] OR percent[All Fields]))

The search was conducted from 1/1/2013 to 3/16/2015. 1,658 results were returned, of which six were extracted.

A systematic review was also performed for peripheral arterial disease and intermittent claudication for GBD 2013. Search terms can be provided upon request.

Table 4 presents a comprehensive list of the citations for the data sources incorporated in the non-fatal models utilized for modeling peripheral artery disease, as well as the proportion of cases with intermittent claudication.

Apart from the claims data from the United States, we did not include any non-literature-based data types. We did not use inpatient hospital data, as peripheral arterial disease is expected to be rare in inpatient data but common in outpatient data as it is a condition usually managed on an outpatient basis, except for specific surgical interventions. This discrepancy leads to implausible correction factors based on inpatient/outpatient information from claims data (~150X); thus, adjusted data cannot be used. Including uncorrected data in the model is likely to lead to incorrect estimates as hospitalization and procedure rates are likely to vary between geographies based on access to and patterns of care.

Table 3: Source counts for peripheral arterial disease (all data sources including outliers)

Measure	Total sources
All measures	45
Incidence	0
Prevalence	37
Proportion	11
Cause of death	2591

Table 4. List of Data Sources for Non-Fatal Models of Peripheral Artery Disease

nid	measure	citation	location
336847	prevalence	Truven Health Analytics. United States MarketScan Claims and Medicare Data 2015. Ann Arbor, United States: Truven Health Analytics.	United States of America
408680	prevalence	Truven Health Analytics. United States MarketScan Claims and Medicare Data 2016. Ann Arbor, United States: Truven Health Analytics.	United States of America
217161	prevalence	Wang Y, Xu Y, Li J, Wei Y, Zhao D, Hou L, Hasimu B, Yang J, Yuan H, Hu D. Characteristics of prevalence in peripheral arterial disease and correlative risk factors and comorbidities among female natural population in China. VASA. 2010; 39(4): 305–11.	China
120234	prevalence	Eldrup N, Sillesen H, Prescott E, Nordestgaard BG. Ankle brachial index, C-reactive protein, and central augmentation index to identify individuals with severe atherosclerosis. Eur Heart J. 2006; 27(3): 316 - 322.	Denmark
140193	prevalence	Ramos R, Quesada M, Solanas P, Subirana I, Sala J, Vila J, Masiá R, Cerezo C, Elosua R, Grau M, Cordón F, Juvinyà D, Fitó M, Isabel Covas M, Clarà A, Angel Muñoz M, Marrugat J, REGICOR Investigators. Prevalence of symptomatic and asymptomatic peripheral arterial disease and the value of the ankle-brachial index to stratify	Spain

		cardiovascular risk. Eur J Vasc Endovasc Surg. 2009; 38(3): 305-11.	
137300	prevalence	Carbayo JA, Divisón JA, Escribano J, López-Abril J, López de Coca E, Artigao LM, Martínez E, Sanchis C, Massó J, Carrión L, Grupo de Enfermedades Vasculares de Albacete (GEVA). Using ankle-brachial index to detect peripheral arterial disease: prevalence and associated risk factors in a random population sample. Nutr Metab Cardiovasc Dis. 2007; 17(1): 41-9.	Spain
137300	prevalence	Carbayo JA, Divisón JA, Escribano J, López-Abril J, López de Coca E, Artigao LM, Martínez E, Sanchis C, Massó J, Carrión L, Grupo de Enfermedades Vasculares de Albacete (GEVA). Using ankle-brachial index to detect peripheral arterial disease: prevalence and associated risk factors in a random population sample. Nutr Metab Cardiovasc Dis. 2007; 17(1): 41-9.	Spain
49502	prevalence	National Center for Health Statistics (NCHS), Centers for Disease Control and Prevention (CDC). United States National Health and Nutrition Examination Survey 1976-1980. Hyattsville, United States: National Center for Health Statistics (NCHS), Centers for Disease Control and Prevention (CDC).	United States of America
49205	prevalence	National Center for Health Statistics (NCHS), Centers for Disease Control and Prevention (CDC). United States National Health and Nutrition Examination Survey 2001-2002. Hyattsville, United States: National Center for Health Statistics (NCHS), Centers for Disease Control and Prevention (CDC).	United States of America
47962	prevalence	National Center for Health Statistics (NCHS), Centers for Disease Control and Prevention (CDC). United States National Health and Nutrition Examination Survey 2003-2004. Hyattsville, United States: National Center for Health Statistics (NCHS), Centers for Disease Control and Prevention (CDC).	United States of America
322188	prevalence	Urbano L, Portilla E, Munoz W, Hofman A, Sierra-Torres CH. Prevalence and risk factors associated to peripheral arterial disease in an adult population from Colombia. Arch Cardiol Mex. 2017.	Colombia
324267	prevalence	Buitrón-Granados LV, Martínez-López C, Escobedo-de la Peña J. Prevalence of peripheral arterial disease and related risk factors in an urban Mexican population. Angiology. 2004; 55(1): 43–51.	Mexico
140190	prevalence	Sodhi HS, Shrestha SK, Rauniyar R, Rawat B. Prevalence of peripheral arterial disease by ankle-brachial index and its correlation with carotid intimal thickness and coronary risk factors in Nepalese population over the age of forty years. Kathmandu Univ Med J (KUMJ). 2007; 5(1): 12-5.	Nepal
140187	prevalence	Chuang S-Y, Chen C-H, Cheng C-M, Chou P. Combined use of brachial-ankle pulse wave velocity and ankle-brachial index for fast assessment of arteriosclerosis and atherosclerosis in a community. Int J Cardiol. 2005; 98(1): 99-105.	China
120233	prevalence	Murabito JM, Evans JC, Nieto K, Larson MG, Levy D, Wilson PWF. Prevalence and clinical correlates of peripheral arterial disease in the Framingham Offspring Study. Am Heart J. 2002; 143(6): 961-5.	United States of America
140188	prevalence	Kweon S-S, Shin M-H, Park K-S, Nam H-S, Jeong S-K, Ryu S-Y, Chung E-K, Choi J-S. Distribution of the ankle-brachial index and associated cardiovascular risk factors in a population of middle-aged and elderly	Republic of Korea

		koreans. J Korean Med Sci. 2005; 20(3): 373-8.	
140191	prevalence	Kröger K, Stang A, Kondratieva J, Moebus S, Beck E, Schmermund A, Möhlenkamp S, Dragano N, Siegrist J, Jöckel K-H, Erbel R, Heinz Nixdorf Recall Study Group. Prevalence of peripheral arterial disease - results of the Heinz Nixdorf recall study. Eur J Epidemiol. 2006; 21(4): 279-85.	Germany
322227	prevalence	Zheng Z-J, Sharrett AR, Chambless LE, Rosamond WD, Nieto FJ, Sheps DS, Dobs A, Evans GW, Heiss G. Associations of ankle-brachial index with clinical coronary heart disease, stroke and preclinical carotid and popliteal atherosclerosis:: the Atherosclerosis Risk in Communities (ARIC) Study. Atherosclerosis. 1997; 131(1): 115–25.	United States of America
336203	prevalence	Ministry of Health and Welfare (Taiwan). Taiwan National Health Insurance Claims Data 2016.	Taiwan (Province of China)
244370	prevalence	Truven Health Analytics. United States MarketScan Claims and Medicare Data - 2010. Ann Arbor, United States: Truven Health Analytics.	United States of America
336850	prevalence	Truven Health Analytics. United States MarketScan Claims and Medicare Data 2011. Ann Arbor, United States: Truven Health Analytics.	United States of America
244371	prevalence	Truven Health Analytics. United States MarketScan Claims and Medicare Data - 2012. Ann Arbor, United States: Truven Health Analytics.	United States of America
336849	prevalence	Truven Health Analytics. United States MarketScan Claims and Medicare Data 2013. Ann Arbor, United States: Truven Health Analytics.	United States of America
336848	prevalence	Truven Health Analytics. United States MarketScan Claims and Medicare Data 2014. Ann Arbor, United States: Truven Health Analytics.	United States of America
295762	prevalence	Meijer WT, Hoes AW, Rutgers D, Bots ML, Hofman A, Grobbee DE. Peripheral arterial disease in the elderly: The Rotterdam Study. Arterioscler Thromb Vasc Biol. 1998; 18(2): 185–92.	The Netherlands
322206	prevalence	Blanes JI, Cairols MA, Marrugat J, ESTIME. Prevalence of peripheral artery disease and its associated risk factors in Spain: The ESTIME Study. Int Angiol. 2009; 28(1): 20-5.	Spain
119831	prevalence	Norman PE, Flicker L, Almeida OP, Hankey GJ, Hyde Z, Jamrozik K. Cohort Profile: The Health In Men Study (HIMS). Int J Epidemiol. 2009; 38(1): 48-52.	Australia
295757	prevalence	Diehm C, Schuster A, Allenberg JR, Darius H, Haberl R, Lange S, Pittrow D, von Stritzky B, Tepohl G, Trampisch H-J. High prevalence of peripheral arterial disease and co-morbidity in 6880 primary care patients: cross-sectional study. Atherosclerosis. 2004; 172(1): 95–105.	United States of America
229971	prevalence	Newman AB, Shemanski L, Manolio TA, Cushman M, Mittelmark M, Polak JF, Powe NR, Siscovick D. Ankle-arm index as a predictor of cardiovascular disease and mortality in the Cardiovascular Health Study. The Cardiovascular Health Study Group. Arterioscler Thromb Vasc Biol. 1999; 19(3): 538–45.	United States of America
140179	prevalence	Guerchet M, Aboyans V, Mbelesso P, Mouanga AM, Salazar J, Bandzouzi B, Tabo A, Clément JP, Preux PM, Lacroix P. Epidemiology of Peripheral Artery Disease in Elder General Population of Two Cities	Central African Republic and Congo

		of Central Africa: Bangui and Brazzaville. Eur J Vasc Endovasc Surg. 2012; 44(2): 164-9.	
140192	prevalence	Wong SYS, Kwok T, Woo J, Lynn H, Griffith JF, Leung J, Tang YYN, Leung PC. Bone mineral density and the risk of peripheral arterial disease in men and women: results from Mr. and Ms Os, Hong Kong. Osteoporos Int. 2005; 16(12): 1933-8.	Hong Kong Special Administrative Region of China
217151	proportion	Félix-Redondo FJ, Fernández-Bergés D, Grau M, Baena-Diez JM, Mostaza JM, Vila J. Prevalence and Clinical Characteristics of Peripheral Arterial Disease in the Study Population Hermex. Rev Esp Cardiol (Engl Ed). 2012; 65(8): 726–33.	Spain
115580	proportion	Alzamora MT, Forés R, Baena-Díez JM, Pera G, Toran P, Sorribes M, Vicheto M, Reina MD, Sancho A, Albaladejo C, Llussà J, PERART/ARTPER study group. The peripheral arterial disease study (PERART/ARTPER): prevalence and risk factors in the general population. BMC Public Health. 2010; 10: 38.	Spain
115581	proportion	Merino J, Planas A, Elosua R, de Moner A, Gasol A, Contreras C, Vidal-Barraquer F, Clarà A. Incidence and risk factors of peripheral arterial occlusive disease in a prospective cohort of 700 adult elderly men followed for 5 years. World J Surg. 2010; 34(8): 1975-9.	Spain
120235	proportion	Murabito JM, Evans JC, Larson MG, Nieto K, Levy D, Wilson PWF. The Ankle-Brachial Index in the Elderly and Risk of Stroke, Coronary Disease, and Death: The Framingham Study. Arch Intern Med. 2003; 163(16): 1939-42.	United States of America
120236	proportion	Ostchega Y, Paulose-Ram R, Dillon CF, Gu Q, Hughes JP. Prevalence of peripheral arterial disease and risk factors in persons aged 60 and older: data from the National Health and Nutrition Examination Survey 1999-2004. J Am Geriatr Soc. 2007; 55(4): 583-9.	United States of America
120233	proportion	Murabito JM, Evans JC, Nieto K, Larson MG, Levy D, Wilson PWF. Prevalence and clinical correlates of peripheral arterial disease in the Framingham Offspring Study. Am Heart J. 2002; 143(6): 961-5.	Massachusetts
120239	proportion	He Y, Jiang Y, Wang J, Fan L, Li X, Hu FB. Prevalence of peripheral arterial disease and its association with smoking in a population-based study in Beijing, China. J Vasc Surg. 2006; 44(2): 333-8.	China
120243	proportion	Sigvant B, Wiberg-Hedman K, Bergqvist D, Rolandsson O, Andersson B, Persson E, Wahlberg E. A population-based study of peripheral arterial disease prevalence with special focus on critical limb ischemia and sex differences. J Vasc Surg. 2007; 45(6): 1185-91.	Sweden
129401	proportion	National Center for Health Statistics (NCHS), Centers for Disease Control and Prevention (CDC). United States National Health and Nutrition Examination Survey 1999-2004. Hyattsville, United States: National Center for Health Statistics (NCHS), Centers for Disease Control and Prevention (CDC).	United States of America
120233	proportion	Murabito JM, Evans JC, Nieto K, Larson MG, Levy D, Wilson PWF. Prevalence and clinical correlates of peripheral arterial disease in the Framingham Offspring Study. Am Heart J. 2002; 143(6): 961-5.	United States of America

Data sources for cause of death can be found in https://ghdx.healthdata.org/gbd-2019/data-input-sources

For GBD 2019 we adjusted prevalence data from claims using the MR-BRT data adjustment procedure described elsewhere the appendix. Our reference data was from literature in which the prevalence of PAD was based on directly measured ABI values. The coefficients in Table 5 below can be used to calculate adjustment factors for alternative definitions. The formula for computing adjustment factors for prevalence is given in equation 1 below. We also included a standardized age variable (age scaled) and a sex variable to the crosswalking procedure to adjust for the possibly of bias. Proportion data was not adjusted.

Equation 1: Calculation of adjustment factors:

$$Estimated\ Reference\ Def = invlogit(logit(Alternative\ Def) - Beta_{Alternative\ Def} - Beta_{Sex} * Sex - Beta_{Age_{scaled}} * Age\ Scaled)$$

Table 5: MR-BRT Crosswalk Adjustment Factors for Peripheral Arterial Disease

Data input	Measure	Reference or alternative case definition	Gamma	Beta Coefficient,Logit (95% UI)*	Adjustment factor**
Measured ABI less than or equal to 0.90	Prevalence	Ref	0	---	
Claims data	Prevalence	Alt		-1.87 (-1.92 to -1.82)	0.15 (0.14 to 0.16)
Age scaled	Prevalence	Alt		0.27 (0.23 to 0.31)	1.30 (1.25 to 1.36)
Sex (male)	Prevalence	Alt		0.29 (0.22 to 0.36)	1.33 (1.25 to 1.43)

**MR-BRT crosswalk adjustments can be interpreted as the factor the alternative case definition is adjusted by to reflect what it would have been had it been measured using the reference case definition. If the log/logit beta coefficient is negative, then the alternative is adjusted up to the reference. If the log/logit beta coefficient is positive, then the alternative is adjusted down to the reference.*
***The adjustment factor column is the exponentiated beta coefficient. For log beta coefficients, this is the relative rate between the two case definitions. For logit beta coefficients, this is the relative odds between the two case definitions.*

Severity splits and disability weights

We used the proportion of intermittent claudication to split the overall prevalence of peripheral arterial disease into symptomatic and asymptomatic peripheral vascular disease. The table below illustrates these values:

Table 6: Severity distribution, details on the severity levels for Peripheral Arterial Disease and the associated disability weight (DW) with that severity.

Severity level	Lay description	DW (95% CI)
Asymptomatic	No symptoms	No DW assigned
Symptomatic	Has cramping pains in the legs after walking a medium distance. The pain goes away after a short rest.	0.014 (0.007–0.025)

Modelling strategy

Prevalence of peripheral arterial disease

For GBD 2019, we used DisMod MR 2.1 to model the overall prevalence of peripheral arterial disease using prevalence dat a from literature studies and and crosswalked claims data. Further statistical details regarding DisMod MR 2.1 can be foun d in a separate section of this appendix.

We included the log-transformed, age-standardized SEV scalar for PAD and log-transformed LDI as fixed-effect, country-level covariates. We set value priors of 0 for incidence from ages 0 to 30. We also set a value prior of 0 for remission for

all ages. Additionally, we set a value prior of 0 for excess mortality in between ages 0 and 30 as well as a value prior between 0 and 0.05 for excess mortality in between ages 30 and 100.

The table below illustrate the beta values and exponentiated beta values for the covariates chosen for the overall peripheral vascular disease model.

Table 7: Summary of covariates used in the Peripheral Arterial Disease DisMod-MR meta-regression model

Covariate	Parameter	Beta	Exponentiated beta
Log-transformed age-standardised SEV scalar: PAD	Prevalence	1.24 (1.22 to 1.25)	3.46 (3.39 to 3.49)
LDI (I$ per capita)	Excess mortality rate	-0.3 (-0.5 to -0.1)	0.74 (0.61 to 0.90)

Proportion of peripheral arterial disease with intermittent claudication
We used DisMod MR to model the proportion of peripheral vascular disease with intermittent claudication. We set a value prior of 0 for proportion for ages 0 to 40. We included the Health Access and Quality Index score as a country-level covariate for excess mortality.

The table below illustrate the study covariates, parameters, beta, and exponentiated beta values for the proportion model for intermittent claudication.

Table 8: Summary of covariates used in the Intermittent Claudication DisMod-MR meta-regression model

Covariate	Parameter	Beta	Exponentiated beta
Healthcare Access and Quality index	Proportion	-.0064(-.014 to -.00066)	0.99 (.99 to 1.00)

Estimation of asymptomatic and symptomatic sequelae
To obtain final estimates for the sequelae of interest, we multiplied the prevalence model by the proportion model at the draw level to generate the prevalence of symptomatic and asymptomatic peripheral vascular disease.

Models were evaluated based on expert review, comparisons with estimates from prior rounds of GBD, and assessing model fit.

There have been no substantive changes from GBD 2017 in terms of modelling strategy for peripheral arterial disease.

Analyses were completed with Python version 3.6.2, Stata version 13, and R version 3.5.0. Statistical code used for GBD estimation is publicly available online (https://ghdx.healthdata.org/gbd-2019/code).

Attributable burden estimation for peripheral arterial disease

Lead exposure

Flowchart

Lead Exposure

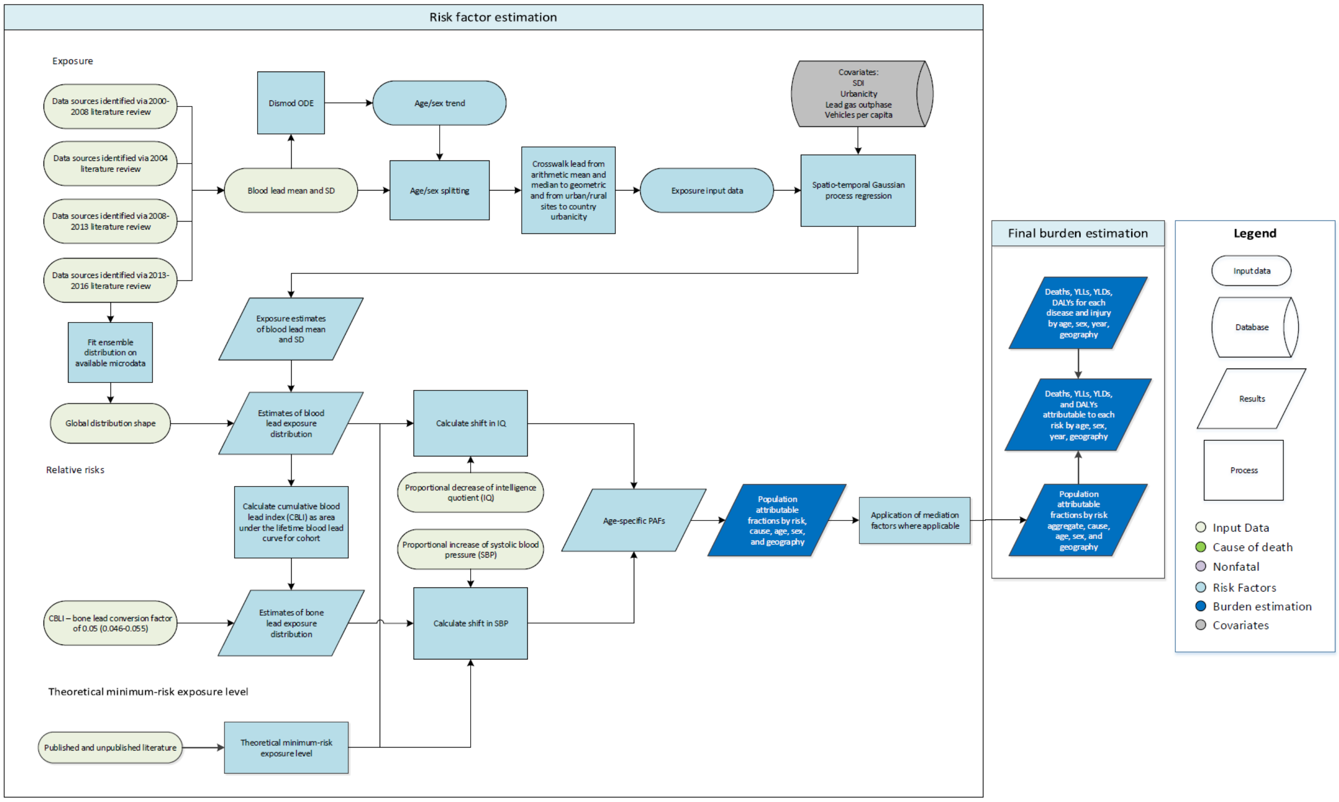

Definitions

Exposure to lead is defined in two different ways according to the currently known pathways of attributable health loss. Acute lead exposure, measured as micrograms of lead per deciliter of blood (µg/dL), is associated with IQ loss in children. Chronic lead exposure, measured as micrograms of lead per gram of bone (µg/g), is associated with increased systolic blood pressure and cardiovascular diseases.

Input data

The input data for lead exposure is primarily extracted from literature reports of blood lead levels, in addition to a few blood lead surveys. Blood lead values are derived from studies that take blood samples and analyse them using various techniques to determine the level of lead present. Our literature review, which was last updated in GBD 2017, resulted in 3183 usable datapoints from 554 different studies, which span the years 1970 to 2017. The second pathway of burden, bone lead exposure, was estimated by calculating a cumulative blood lead index for cohorts using estimated blood lead exposure over their lifetime. The cumulative blood lead index is then used to estimate bone lead using a scalar defined in literature.[1] Table 1 provides a summary of the exposure input data used.

Table 1: Data inputs for exposure

Input data	Exposure
Source count (total)	552

Number of countries with data	84

Data processing

In GBD 2019, we used MR-BRT to crosswalk our data. Blood lead exposure data are reported in the literature as either an arithmetic mean, a geometric mean, or a median. To standardise the data, we adjusted all values reported as a geometric mean or median to reflect what they would have been had the study reported the arithmetic mean. Additionally, the data come from locations of varying urbanicity (proportion of individuals in a given location living in an urban area). Because we expected the urbanicity of a location to affect our estimates, we adjusted our data so that they were equivalent to the average urbanicity of the country from which the data were collected. Tables 2 and 3 show the MR-BRT crosswalk adjustment factors.

Table 2: MR-BRT crosswalk adjustment factors for lead exposure (mean)

**Reference or alternative case definition**	**Gamma**	**Beta coefficient, log (95% CI)**	**Adjustment factor***
Reference (data reported as arithmetic mean)	0.25	---	---
Alternative (data reported as geometric mean)		-0.178 (-0.667 to 0.311)	0.837 (0.513 to 1.365)
Alternative (data reported as median)		-0.157 (-0.646 to 0.333)	0.855 (0.524 to 1.395)

Table 3: MR-BRT crosswalk adjustment factors for lead exposure (urbanicity)

**Reference or alternative case definition**	**Gamma**	**Beta coefficient, log (95% CI)**	**Adjustment factor***
Reference (study urbanicity equals national average urbanicity)	0.32	---	---
Alternative (study urbanicity does not equal national average urbanicity)		0.222 (-0.411 to 0.855)	1.248 (0.663 to 2.351)

**Adjustment factor is the transformed beta coefficient in normal space and can be interpreted as the factor by which the alternative case definition is adjusted to reflect what it would have been if measured as the reference.*

As an example of how the crosswalking works, a datapoint of 4.85 reported as a geometric mean was multiplied by the adjustment factor of 0.837 to get an estimated arithmetic mean of 4.06. The estimated arithmetic mean value was then used as the final datapoint in our modelling process.

Exposure modelling

The methodology to estimate lead exposure last underwent significant change in GBD 2013. Global exposure had been previously modelled using age-integrating Bayesian hierarchal modelling (DisMod-MR). The modelling process was updated for GBD 2013 by shifting to a spatiotemporal Gaussian process regression methodology (ST-GPR). This allowed for estimates of all country-age-sex-year groups for single years instead of five-year periods. This approach improved the granularity of estimates for bone lead, which requires back-estimation of previous blood lead to calculate a cumulative blood lead index.

For GBD 2019, the spatiotemporal Gaussian process regression modelling methodology was updated as detailed in the appendix specific to this analytical technique, which is common to a variety of risk factors. In order to predict blood lead in country-years with insufficient data, covariates that have been produced across time and space relevant to this analysis were used. For blood lead exposure, the covariates determined to have predictive ability were the Socio-demographic Index (SDI), urbanicity, the combined number of two- and four-wheeled vehicles per capita, and a covariate indicating whether leaded gasoline had been phased out in a given country-year (smoothed over the first five years of phase-out to reflect its gradual implementation). ST-GPR was used to produce estimates of mean and standard deviation of blood lead for all age groups, for both sexes, and for all GBD locations from 1970 to 2019. The linear regression equation is shown below.

$$log(data) \sim sdi + urbanicity + (leaded\ gas\ outphase * vehicles\ per\ capita) + (1|level\_1)$$

SDI = Socio-demographic Index
Urbanicity = proportion of population living in urban areas
Leaded gas outphase = whether or not a country has banned use of leaded gasoline
Vehicles per capita = number of 2- and 4-wheeled vehicles per capita
(1|level_1) = super-region-level random effects

In earlier iterations of GBD, the distribution of lead exposure was assumed to be log-normal. Since GBD 2016, ensemble modelling techniques were used to find an optimal global distribution by fitting a variety of distributions to the available blood lead microdata. This was a common update for all continuous risk factors. The ST-GPR mean and standard deviation estimates for blood lead were used with the global distribution shape to determine distributions for blood lead exposure. The distribution ultimately included 11 different probability distributions: exponential, gamma, inverse-gamma, mirrored gamma, log-logistic, Gumbel, mirrored Gumbel, Weibull, log-normal, normal, and beta. A little over 80% of the final distribution was log-logistic (35%), inverse-gamma (18%), log-normal (16%), or mirrored Gumbel (12%), with the seven other distributions comprising the remaining 20%.

To calculate blood lead over the lifetime of a given cohort, blood lead was assumed to grow linearly from 2.0 µg/dL in 1920 (see section *Theoretical minimum-risk exposure level*) to the value for that cohort in 1970. Using the exposure distributions of blood lead over time and space, cohorts were constructed such that lifetime blood lead could be expressed as a curve over each year of life. The area under this curve was the cumulative blood lead index, which was used to estimate bone lead in a given year with the aforementioned scalar.

Estimating attributable burden

Assessment of risk-outcome pairs

We included outcomes based on the strength of available evidence supporting a causal relationship. Blood lead level (a measure of acute lead exposure) is paired with idiopathic developmental intellectual disability as modelled through the impact of blood lead levels on IQ in children. Bone lead level (a measure of chronic lead exposure) is paired with systolic blood pressure, and subsequently to all cardiovascular outcomes to which systolic blood pressure is paired, which include the following: rheumatic heart disease, ischaemic heart disease, ischaemic stroke, intracerebral haemorrhage,

hypertensive heart disease, other cardiomyopathy, atrial fibrillation and flutter, aortic aneurysm, peripheral artery disease, endocarditis, other cardiovascular and circulatory diseases, chronic kidney disease due to hypertension, chronic kidney disease due to glomerulonephritis, and chronic kidney disease due to other and unspecified causes.

Theoretical minimum-risk exposure level

In previous iterations of GBD, the TMREL was estimated at 2.0 µg/dL. This level was based upon ambient sources of lead that would be impossible to eliminate[2] and a review of the literature indicating no consistent statistically significant estimates of increased relative risks at lower levels of blood lead. We have continued to use a TMREL of 2.0 µg/dL for GBD 2019. While the majority of global exposure is estimated to be well above this level, average blood lead exposures in a number of countries have fallen below 2.0 µg/dL in recent years (including, for example, the United States, where the average adult BLL was 1.2 µg/dL in 2009-2010).[3] This is consistent with estimates of pre-industrial blood lead in humans, which are as low as 0.018 µg/dL.[4] This suggests that the TMREL ought to be lowered, and this change will be evaluated for the GBD 2020 cycle.

Relative risks

Because the relative risk of IQ loss from lead exposure is specific to children, in previous iterations of GBD, no burden of lead via IQ loss was estimated in the population aged 15 and above. To better account for the continued burden of past lead exposure on IQ in older age groups, since GBD 2016 we have constructed cohorts from the entire population. Estimates of a cohort's lead exposure in early childhood (at 24 months of age) were used to determine past IQ loss, and thus calculate burden via the impact on concurrent IQ in the older population.

Blood lead relative risks were previously taken from a 2005 pooled analysis that was first incorporated in GBD 2010.[5] Those relative risks were then updated for GBD 2017 using a 2013 re-analysis of the findings of that 2005 paper, providing slightly adjusted relative risk estimates specific to exposure at 24 months of age.[6] The bone lead relative risks were taken from a 2008 meta-analysis that showed a 0.26 mmHg increase in systolic blood pressure (SBP) per 10 µg/g increase in bone lead (95% CI: 0.02 to 0.50).[7] Table 4 shows a summary of this information. Because bone lead is associated with increases in SBP, all of the health burden attributable to exposure to bone lead is mediated through SBP. As such, the relative risks for bone lead exposure are all the same as the relative risks that SBP has for its outcomes. Table 5 shows the relative risks for exposure to blood lead, and Table 6 shows a snapshot of the relative risks for bone lead (a full table can be found in Appendix Table 4a).

Table 4: Data inputs for relative risks

Input data	Relative risk
Source count (total)	2

Table 5: Relative risks for exposure to blood lead

Exposure level	IQ shift
2 µg/dL	0.0 (0.0 to 0.0)
4 µg/dL	3.146 (1.154 to 5.139)
6 µg/dL	3.804 (1.395 to 6.213)
8 µg/dL	4.296 (1.575 to 7.016)
10 µg/dL	4.688 (1.719 to 7.656)

12 µg/dL	5.014 (1.839 to 8.19)
15 µg/dL	5.42 (1.988 to 8.853)
20 µg/dL	5.952 (2.183 to 9.721)
25 µg/dL	6.37 (2.336 to 10.403)
30 µg/dL	6.713 (2.462 to 10.965)
35 µg/dL	7.006 (2.569 to 11.442)
40 µg/dL	7.26 (2.662 to 11.857)

Table 6: Relative risks for exposure to bone lead (snapshot)

Exposure level	Outcome	25-29 years	60-64 years	95+ years
10 µg/g	Ischaemic heart disease	1.042 (1.022 to 1.06)	1.021 (1.018 to 1.025)	1.014 (1.008 to 1.022)
10 µg/g	Ischaemic stroke	1.038 (1.021 to 1.06)	1.021 (1.016 to 1.026)	1.011 (1.006 to 1.019)
10 µg/g	Haemorrhagic stroke	1.047 (1.021 to 1.068)	1.024 (1.018 to 1.03)	1.015 (1.007 to 1.026)
10 µg/g	Hypertensive heart disease	1.066 (1.038 to 1.09)	1.04 (1.023 to 1.073)	1.033 (1.006 to 1.075)
10 µg/g	Non-rheumatic calcific aortic valve disease	1.035 (1.015 to 1.055)	1.014 (1.01 to 1.016)	1.007 (1.004 to 1.013)
10 µg/g	Atrial fibrillation and flutter	1.035 (1.018 to 1.056)	1.016 (1.014 to 1.018)	1.008 (1.005 to 1.01)
10 µg/g	Aortic aneurysm	1.027 (1.014 to 1.048)	1.014 (1.011 to 1.016)	1.007 (1.004 to 1.01)
10 µg/g	Peripheral vascular disease	1.034 (1.011 to 1.056)	1.009 (1.006 to 1.011)	1.006 (1.003 to 1.009)
10 µg/g	Endocarditis	1.035 (1.015 to 1.055)	1.014 (1.01 to 1.016)	1.007 (1.004 to 1.013)

Population attributable fraction

We used the standard GBD population attributable fraction (PAF) equation to calculate PAFs for bone lead exposure and each of its paired outcomes using exposure estimates and relative risks. We used a similar approach for estimating PAFs for the burden of intellectual disability attributable to blood lead, which uses the estimated distribution of intellectual disability and the modeled shifts in IQ due to blood lead levels to determine the PAF.

References

1. Hu H, Shih R, Rothenberg S, Schwartz BS. The epidemiology of lead toxicity in adults: measuring dose and consideration of other methodologic issues. Environ Health Perspect. 2007;115(3):455-62.
2. Pruss-Astun A, Fewtrell L, Landrigan PJ, Ayuso-Mateos JL. Lead Exposure. In: Ezzati M, Lopez AD, Rodgers A, Murray CJ, eds. Comparative quantifications of health risks: Global and regional burden of disease attrituable to selected major risk factors. Geneva, World Health Organization, 2004: 1496-542

3. CDC - Adult Blood Lead Epidemiology and Surveillance (ABLES): Program Description: NIOSH Workplace Safety and Health Topic." Centers for Disease Control and Prevention, 11 May 2018, www.cdc.gov/niosh/topics/ables/description.html.
4. Flegal AR, Smith DR. Lead levels in preindustrial humans. N Engl J Med. 1992;326(19):1293-4.
5. Lanphear BP, Hornung R, Khoury J, et al. Low-level environmental lead exposure and children's intellectual function: an international pooled analysis. Environ Health Perspect. 2005;113(7):894-9.
6. Crump K, Van Landingham C, Bowers T, Cahoy D, Chandalia J. A statistical reevaluation of the data used in the Lanphear et al. (2005) pooled-analysis that related low levels of blood lead to intellectual deficits in children. Critical Reviews in Toxicology. 2013;43(9):785-799.
7. Navas-Acien A, Schwartz BS, Rothenberg SJ, Hu H, Silbergeld EK, Guallar E. Bone lead levels and blood pressure endpoints: a meta-analysis. Epidemiology. 2008;19(3):496-504.

Smoking

Flowchart

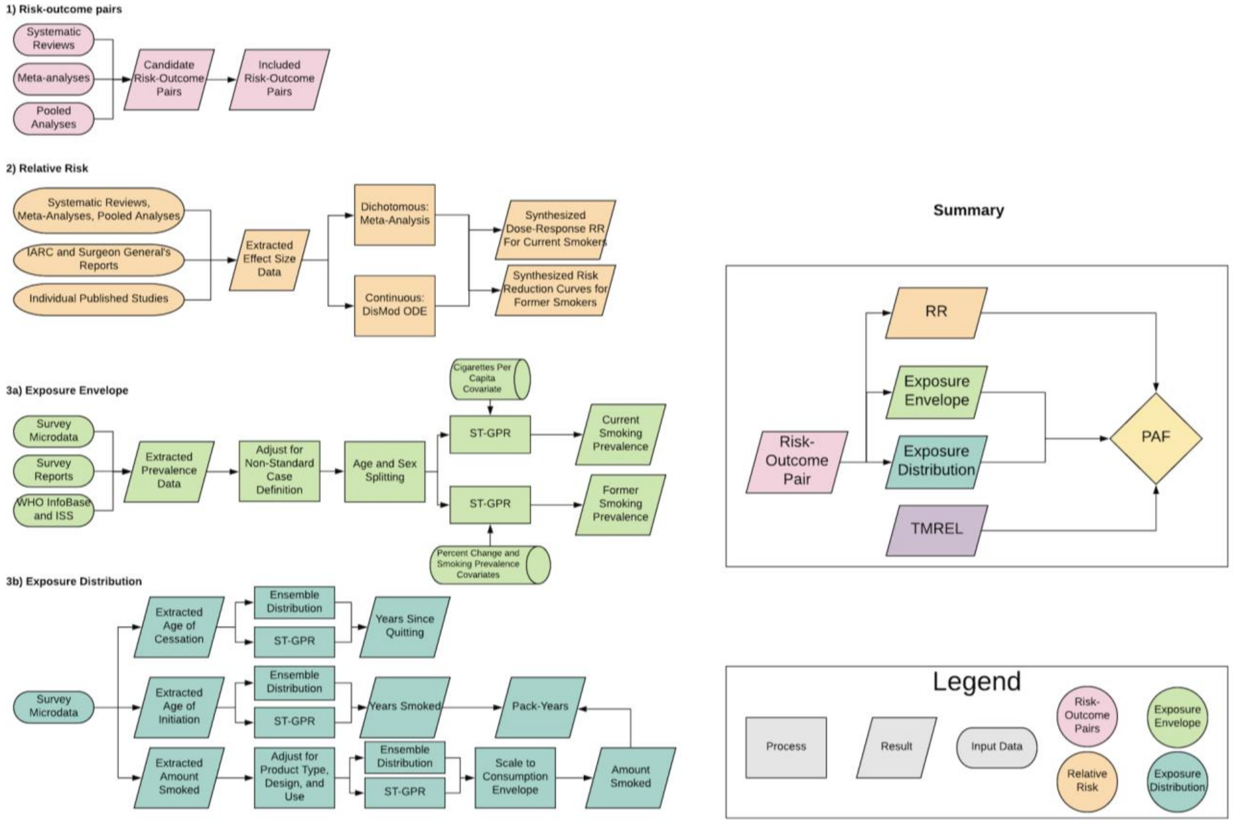

Input data and methodological summary

Definition

Exposure

As in GBD 2017, we estimated the prevalence of current smoking and the prevalence of former smoking using data from cross-sectional nationally representative household surveys. We defined current smokers as individuals who currently use any smoked tobacco product on a daily or occasional basis. We defined former smokers as individuals who quit

using all smoked tobacco products for at least six months, where possible, or according to the definition used by the survey.

Input data

Our extraction method has not changed from GBD 2017. We extracted primary data from individual-level microdata and survey report tabulations. We extracted data on current, former, and/or ever smoked tobacco use reported as any combination of frequency of use (daily, occasional, and unspecified, which includes both daily and occasional smokers) and type of smoked tobacco used (all smoked tobacco, cigarettes, hookah, and other smoked tobacco products such as cigars or pipes), resulting in 36 possible combinations. Other variants of tobacco products, for example hand-rolled cigarettes, were grouped into the four type categories listed above based on product similarities.

For microdata, we extracted relevant demographic information, including age, sex, location, and year, as well as survey metadata, including survey weights, primary sampling units, and strata. This information allowed us to tabulate individual-level data in the standard GBD five-year age-sex groups and produce accurate estimates of uncertainty. For survey report tabulations, we extracted data at the most granular age-sex group provided.

Table 1: Data inputs for exposure for smoking.

Input data	Exposure
Source count (total)	3439
Number of countries with data	201

Table 2: Data inputs for relative risks for smoking.

Input data	Relative risk
Source count (total)	673
Number of countries with data	16

Crosswalk

Our GBD smoking case definitions were current smoking of any tobacco product and former smoking of any tobacco product. All other data points were adjusted to be consistent with either of these definitions. Some sources contained information on more than one case definition and these sources were used to develop the adjustment coefficient to transform alternative case definitions to the GBD case definition. The adjustment coefficient was the beta value derived from a linear model with one predictor and no intercept. We used the same crosswalk adjustment coefficients as in GBD 2017, and thus we have not included a methods explanation in this appendix, as it has been detailed previously.

Age and sex splitting

As in GBD 2017, we split data reported in broader age groups than the GBD 5-year age groups or as both sexes combined by adapting the method reported in Ng et al[1] to split using a sex- geography- time-specific reference age pattern. We separated the data into two sets: a training dataset, with data already falling into GBD sex-specific 5-year age groups, and a split dataset, which reported data in aggregated age or sex groups. We then used spatiotemporal Gaussian process regression (ST-GPR) to estimate sex-geography-time-specific age patterns using data in the training dataset. The estimated age patterns were used to split each source in the split dataset.

The ST-GPR model used to estimate the age patterns for age-sex splitting used an age weight parameter value that minimises the effect of any age smoothing. This parameter choice allowed the estimated age pattern to be driven by data, rather than being enforced by any smoothing parameters of the model. Because these age-sex split data points were to be incorporated in the final ST-GPR exposure model, we did not want to doubly enforce a modelled age pattern for a given sex-location-year on a given aggregate data point.

Modelling strategy

Smoking prevalence modelling

We used ST-GPR to model current and former smoking prevalence. The model is nearly identical to that in GBD 2017. Full details on the ST-GPR method are reported elsewhere in the appendix. Briefly, the mean function input to GPR is a complete time series of estimates generated from a mixed effects hierarchical linear model plus weighted residuals smoothed across time, space, and age. The linear model formula for current smoking, fit separately by sex using restricted maximum likelihood in R, is:

$$logit(p_{g,a,t}) = \beta_0 + \beta_1 CPC_{g,t} + \sum_{k=2}^{19} \beta_k I_{A[a]} + \alpha_s + \alpha_r + \alpha_g + \epsilon_{g,a,t}$$

Where $CPC_{g,t}$ is the tobacco consumption covariate by geography g and time t, described above, $I_{A[a]}$ is a dummy variable indicating specific age group A that the prevalence point $p_{g,a,t}$ captures, and α_s, α_r, and α_g are super-region, region, and geography random intercepts, respectively. Random effects were used in model fitting but not in prediction.

The linear model formula for former smoking is:

$$logit(p_{g,a,t}) = \beta_0 + \beta_1 PctChange_{A[a],g,t} + \beta_3 CSP_{A[a],g,t} + \sum_{k=3}^{20} \beta_k I_{A[a]} + \alpha_s + \alpha_r + \alpha_g + \epsilon_{g,a,t}$$

Where $PctChange_{A[a],g,t}$ is the percentage change in current smoking prevalence from the previous year, and $CSP_{A[a],g,t}$ is the current smoking prevalence by specific age group A, geography g, and time t that point $p_{g,a,t}$ captures, both derived from the current smoking ST-GPR model defined above.

Supply-side estimation

The methods for modelling supply-side-level data were changed substantially from those used in GBD 2017. The raw data were domestic supply (USDA Global Surveillance Database and UN FAO) and retail supply (Euromonitor) of tobacco. Domestic supply was calculated as production + imports - exports. The data went through three rounds of outliering. First, they were age-sex split using daily smoking prevalence to generate number of cigarettes per smoker per day for a given location-age-sex-year. If more than 12 points for a particular source-location-year (equal to over 1/3 of the split points) were above the given thresholds, that source-location-year was outliered. A point would not be outliered if it was (in cigarettes per smoker): under five (10–14 year olds); under 20 (males, 15–19 year olds); under 18 (females, 15–19 year olds); under 38/35 and over three (males/females, 20+ year olds). These thresholds were chosen by visualising histograms of the data for each age-sex, as well as with expert knowledge about reasonable consumption levels. In the second round of outliering, the mean tobacco per capita value over a 10-year window was calculated. If a point was over 70% of that mean value away from the mean value, it was outliered. The 70% limit was chosen using histograms of these distances. Additionally, some manual outliering was performed to account for edge cases. Finally, data smoothing was performed by taking a three-year rolling mean over each location-year.

Next, a simple imputation to fill in missing years was performed for all series to remove compositional bias from our

final estimates. Since the data from our main sources covered different time periods, by imputing a complete time series for each data series, we reduced the probability that compositional bias of the sources was leading to biased final estimates. To impute the missing years for each series, we modelled the log ratio of each pair of sources as a function of an intercept and nested random effects on super-region, region, and location. The appropriate predicted ratio was multiplied by each source that we did have, and then the predictions were averaged to get the final imputed value. For example, if source A was missing for a particular location-year, but sources B and C were present, then we predicted A twice: once from the modelled ratio of A to B, and again from the modelled ratio of A to C. These two predictions were then averaged. For some locations where there was limited overlap between series, the predicted ratio did not make sense, and a regional ratio was used.

Finally, variance was calculated both across series (within a location-year) as well as across years (within a location-source). Additionally, if a location-year had one imputed point was, the variance was multiplied by 2. If a location-year had two imputed points, the variance was multiplied by 4. The average estimates in each location-year were the input to an ST-GPR model. For this, we used a simple mixed effects model, which was modelled in log space with nested location random effects. Subnational estimates were then further modelled by splitting the country-level estimates using current smoking prevalence.

Theoretical minimum-risk exposure level

The theoretical minimum-risk exposure level is 0.

Exposure among current and former smokers

Identical to GBD 2017, we estimated exposure among current smokers for two continuous indicators: cigarettes per smoker per day and pack-years. Pack-years incorporates aspects of both duration and amount. One pack-year represents the equivalent of smoking one pack of cigarettes (assuming a 20-cigarette pack) per day for one year. Since the pack-years indicator collapses duration and intensity into a single dimension, one pack-year of exposure can reflect smoking 40 cigarettes per day for six months or smoking 10 cigarettes per day for two years.

To produce these indicators, we simulated individual smoking histories based on distributions of age of initiation and amount smoked. We informed the simulation with cross-sectional survey data capturing these indicators, modelled at the mean level for all locations, years, ages, and sexes using ST-GPR. We rescaled estimates of cigarettes per smoker per day to an envelope of cigarette consumption based on supply-side data. We estimated pack-years of exposure by summing samples from age- and time-specific distributions of cigarettes per smoker for a birth cohort in order to capture both age trends and time trends and avoid the common assumption that the amount someone currently smokes is the amount they have smoked since they began smoking. All distributions were age-, sex-, and region- specific ensemble distributions, which were found to outperform any single distribution.

We estimated exposure among former smokers using years since cessation. We utilised ST-GPR to model mean age of cessation using cross-sectional survey data capturing age of cessation. Using these estimates, we generated ensemble distributions of years since cessation for every location, year, age group, and sex.

Relative risk

The same risk-outcome pairs from GBD 2017 were used: tuberculosis, lower respiratory tract infections, oesophageal cancer, stomach cancer, bladder cancer, liver cancer, laryngeal cancer, lung cancer, breast cancer, cervical cancer, colorectal cancer, lip and oral cancer, nasopharyngeal cancer, other pharyngeal cancer, pancreatic cancer, kidney cancer, leukaemia, ischaemic heart disease, ischaemic stroke, haemorrhagic stroke, subarachnoid haemorrhage, atrial fibrillation and flutter, aortic aneurysm, peripheral arterial disease, chronic obstructive pulmonary disease, other chronic respiratory diseases, asthma, peptic ulcer disease, gallbladder and biliary tract diseases, Alzheimer disease and other dementias, Parkinson disease (protective), multiple sclerosis, type-II diabetes, rheumatoid arthritis, low back pain, cataracts, macular degeneration, and fracture.

Dose-response risk curves

Input data for relative risks were nearly the same as in GBD 2017. The only addition was for chronic obstructive pulmonary disease, for which a few additional studies were included. We synthesised effect sizes by cigarettes per smoker per day, pack-years, and years since quitting from cohort and case-control studies to produce nonlinear dose-response curves using a Bayesian meta-regression model. For outcomes with significant differences in effect size by sex or age, we produced sex- or age-specific risk curves.

We estimated risk curves of former smokers compared to never smokers taking into account the rate of risk reduction among former smokers seen in the cohort and case-control studies, and the cumulative exposure among former smokers within each age, sex, location, and year group.

Population attributable fraction (PAF)

As in GBD 2017, we estimated PAFs based on the following equation:

$$PAF = \frac{p(n) + p(f)\int \exp(x) * rr(x) + p(c)\int \exp(y) * rr(y) - 1}{p(n) + p(f)\int \exp(x) * rr(x) + p(c)\int \exp(y) * rr(y)}$$

where $p(n)$ is the prevalence of never smokers, $p(f)$ is the prevalence of former smokers, $p(c)$ is the prevalence of current smokers, $\exp(x)$ is a distribution of years since quitting among former smokers, $rr(x)$ is the relative risk for years since quitting, $\exp(y)$ is a distribution of cigarettes per smoker per day or pack-years, and $rr(y)$ is the relative risk for cigarettes per smoker per day or pack-years.

We used pack-years as the exposure definition for cancers and chronic respiratory diseases, and cigarettes per smoker per day for cardiovascular diseases and all other health outcomes.

References

1. Ng M, Freeman MK, Fleming TD, Robinson M, Dwyer-Lindgren L, Thomson B, et al. Smoking Prevalence and Cigarette Consumption in 187 Countries, 1980–2012. *JAMA*. 2014 Jan 8;311(2):183–92.

High fasting plasma glucose

Flowchart

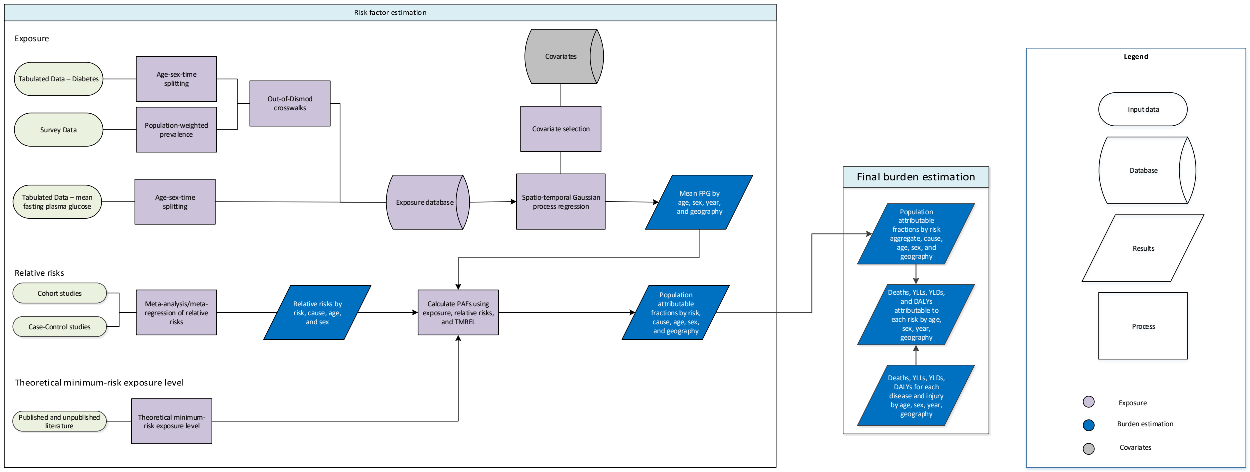

Case definition

High fasting plasma glucose (FPG) is measured as the mean FPG in a population, where FPG is a continuous exposure in units of mmol/L. Since FPG is along a continuum, we define high FPG as any level above the TMREL, which is 4.8-5.4 mmol/L.

Data seeking

Exposure

We conducted a systematic review for FPG and diabetes in GBD 2019. We use all available sources on FPG and prevalence of diabetes in the FPG model.

1. Search terms:

Diabetes Mellitus search string: (diabetes[TI] AND (prevalence[TIAB] OR incidence[TIAB])) OR ('Diabetes Mellitus'[MeSH Terms] AND 'epidemiology'[MeSH Terms]) OR (diabetes[TI] AND 'epidemiology'[MeSH Terms]) NOT gestational[All Fields] NOT ('neoplasms'[MeSH Terms] OR 'neoplasms'[All Fields] OR 'cancer'[All Fields]) NOT ('mice'[MeSH Terms] OR 'mice'[All Fields]) NOT ('schizophrenia'[MeSH Terms] OR 'schizophrenia'[All Fields]) NOT ('emigrants and immigrants'[MeSH Terms] OR ('emigrants'[All Fields] AND 'immigrants'[All Fields]) OR 'emigrants and immigrants'[All Fields] OR 'immigrants'[All Fields]) NOT ('pregnancy'[MeSH Terms] OR 'pregnancy'[All Fields] OR 'gestation'[All Fields]) NOT ('rats'[MeSH Terms] OR 'rats'[All Fields] OR 'rat'[All Fields]) NOT ('kidney'[MeSH Terms] OR 'kidney'[All Fields]) NOT renal[All Fields] NOT ('vitamins'[Pharmacological Action] OR 'vitamins'[MeSH Terms] OR 'vitamins'[All Fields] OR 'vitamin'[All Fields])

And

FPG search string: ((“glucose”[Mesh] OR “hyperglycemia”[Mesh] OR “prediabetic state”[Mesh]) AND "Geographic Locations"[Mesh] NOT "United States"[Mesh]) AND ("humans"[Mesh] AND "adult"[MeSH]) AND ("Data Collection"[Mesh] OR "Health Services Research"[Mesh] OR "Population Surveillance"[Mesh] OR "Vital statistics"[Mesh] OR "Population"[Mesh] OR "Epidemiology"[Mesh] OR surve*[TiAb]) NOT Comment[ptyp] NOT Case Reports[ptyp]) NOT "hospital"[TiAb]

Search date: October 17, 2018. The search took place for the following dates: 10/15/2017-10/16/2018. The number of studies returned was 717, and the number of studies extracted was 36.

Figure 1: PRISMA diagram of data sources used in GBD 2019 high fasting plasma glucose model

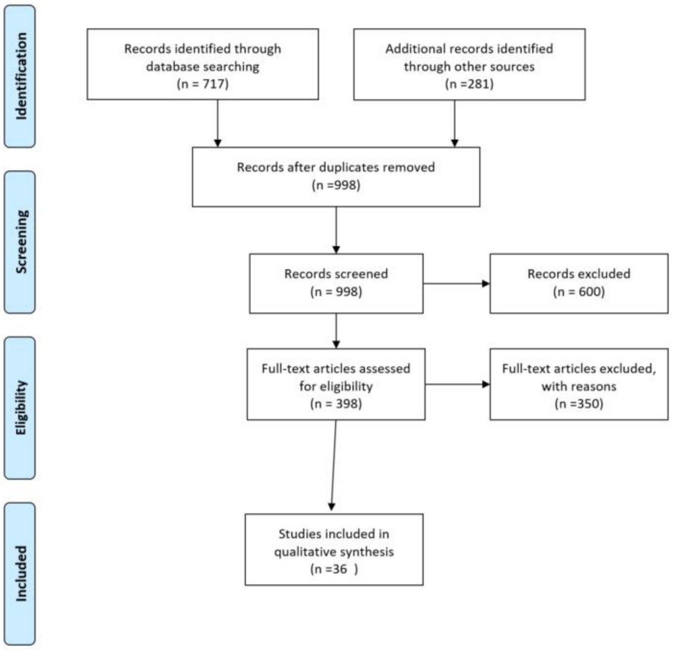

Data inputs

Data inputs come from 3 sources:

- Estimates of mean FPG in a representative population
- Individual-level data of fasting plasma glucose measured from surveys
- Estimates of diabetes prevalence in a representative population

Data sources that did not report mean FPG or prevalence of diabetes are excluded from analysis. When a study reported both mean fasting plasma glucose (FPG) and prevalence of diabetes, we use the mean FPG for exposure estimates. Where possible, individual-level data supersede any data described in a study. Individual-level data are aggregated to produce estimates for each 5-year age group, sex, location, and year of a survey.

Table 1: Number of sources used in exposure and relative risk models in GBD 2019

Measure	Total sources	Countries with data
Total	549	127
Relative risk	20	-
Exposure	529	127

Data processing

We perform several processing steps to the data in order to address sampling and measurement inconsistencies that will ensure the data are comparable.

1. *Small sample size*
   Estimates in a sex and age group with a sample size <30 persons is considered a small sample size. In order to avoid small sample size problems that may bias estimates, data are collapsed into the next age group in the same study till the sample size reach at least 30 persons. The intent of collapsing the data is to preserve as much granularity between age groups as possible. If the entire study sample consists of <30 persons and did not include a population-weight, the study is excluded from the modelling process.

2. *Crosswalks*
   We predicted mean FPG from diabetes prevalence using an ensemble distribution. We characterized the

distribution of FPG using individual-level data. Details on the ensemble distribution can be found elsewhere in the Appendix. Before predicting mean FPG from prevalence of diabetes, we ensured that the prevalence of diabetes was based on the reference case definition: fasting plasma glucose (FPG) >126 mg/dL (7 mmol/L) or on treatment. For more details on how the case-definition crosswalk is conducted, please see the diabetes mellitus appendix in *Global, regional, and national incidence, prevalence, and years lived with disability for 354 diseases and injuries for 195 countries, 1990–2019: a systematic analysis for the Global Burden of Disease Study 2019*.

Exposure modelling

Exposure estimates are produced for every year between 1980 to 2019 for each national and subnational location, sex, and for each 5-year age group starting from 25 years. As in previous rounds of GBD, we used a Spatio-Temporal Gaussian Process Regression (ST-GPR) framework to model the mean fasting plasma glucose at the location-, year-, age-, and sex- level. Updates to the ST-GR modelling framework for GBD 2019 are detailed elsewhere in the Appendix.

Fasting plasma glucose is frequently tested or reported in surveys aiming at assessing the prevalence of diabetes mellitus. In these surveys, the case definition of diabetes may include both a glucose test and questions about treatment for diabetes. People with positive history of diabetes treatment may be excluded from the FPG test. Thus, the mean FPG in these surveys would not represent the mean FPG in the entire population. In this event, we estimated the prevalence of diabetes assuming a definition of FPG>126 mg/dL (7mmol/L), then crosswalked it to our reference case definition, and then predicted mean FPG.

To inform our estimates in data-sparse countries, we systematically tested a range of covariates and selected age specific prevalence of obesity as a covariate based on direction of the coefficient and significance level.

Mean FPG is estimated using a mixed-effects linear regression, run separately by sex:

$$\mathrm{logit}(\mathrm{FPG}_{c,a,t}) = \beta_0 + \beta_1 p_{\mathrm{overweight}_{c,a,t}} + \sum_{k=2}^{16} \beta_k I_{A[a]} + \alpha_s + \alpha_r + \alpha_c + \epsilon_{c,a,t}$$

where $p_{\mathrm{overweight}_{c,a,t}}$ is the prevalence of overweight, $I_{A[a]}$ is an indicator variable for a fixed effect on a given 5-year age group, and α_s α_r α_c are random effects at the super-region, region, and country level, respectively. The estimates were then propagated through the ST-GPR framework to obtain 1000 draws for each location, year, age, and sex.

Theoretical minimum-risk exposure level

The theoretical minimum-risk exposure level (TMREL) for FPG is 4.8-5.4 mmol/L. This was calculated by taking the person-year weighted average of the levels of FPG that were associated with the lowest risk of mortality in the pooled analyses of prospective cohort studies.[1]

Relative risks

We estimate 15 outcomes due to high fasting plasma glucose (continuous risk) or diabetes (categorical risk).

**Risk**	**Outcome**

Fasting plasma glucose	Ischemic heart disease
Fasting plasma glucose	Ischemic stroke
Fasting plasma glucose	Subarachnoid hemorrhage
Fasting plasma glucose	Intracerebral hemorrhage
Fasting plasma glucose	Peripheral vascular disease
Fasting plasma glucose	Type 1 diabetes
Fasting plasma glucose	Type 2 diabetes
Fasting plasma glucose	Chronic kidney disease due to Type 1 diabetes
Fasting plasma glucose	Chronic kidney disease due to Type 2 diabetes
Diabetes mellitus	Drug-resistant tuberculosis
Diabetes mellitus	Drug-susceptible tuberculosis
Diabetes mellitus	Multidrug-resistant tuberculosis without extensive drug resistance
Diabetes mellitus	Extensively drug-resistant tuberculosis
Diabetes mellitus	Liver cancer due to NASH
Diabetes mellitus	Liver cancer due to other causes
Diabetes mellitus	Pancreatic cancer
Diabetes mellitus	Ovarian cancer
Diabetes mellitus	Colorectal cancer
Diabetes mellitus	Bladder cancer
Diabetes mellitus	Lung cancer
Diabetes mellitus	Breast cancer
Diabetes mellitus	Glaucoma
Diabetes mellitus	Cataracts
Diabetes mellitus	Dementia

Relative risks for High Fasting Plasma Glucose (continuous risk)

After a review of the chronic kidney disease literature, we determined that there is only an attributable risk of chronic

kidney disease due to diabetes type 1 and chronic kidney disease due to diabetes type 2 to FPG. Thus, in GBD 2019 we removed chronic kidney disease due to glomerulonephritis, chronic kidney disease due to hypertension, chronic kidney disease due to other causes as an outcome.

Relative risks (RR) were obtained from dose-response meta-analysis of prospective cohort studies. Please see the citation list for a full list of studies that are utilized. For cardiovascular outcomes, we estimated age-specific RRs using DisMod-MR 2.1 with log (RR) as the dependent variable and median age at event as the independent variable with an intercept at age 110. Morbidity and mortality directly caused by diabetes type 1 and diabetes type 2 is considered directly attributable to FPG.

Relative risks for Diabetes mellitus (Categorical risk)
Relative risks were obtained from meta-analysis of cohort studies. Please see the citation list for a full list of studies that are utilized.

References

1 . Singh GM, Danaei G, Farzadfar F, *et al.* The age-specific quantitative effects of metabolic risk factors on cardiovascular diseases and diabetes: a pooled analysis. *PloS One* 2013; **8**: e65174.

High systolic blood pressure

Flowchart

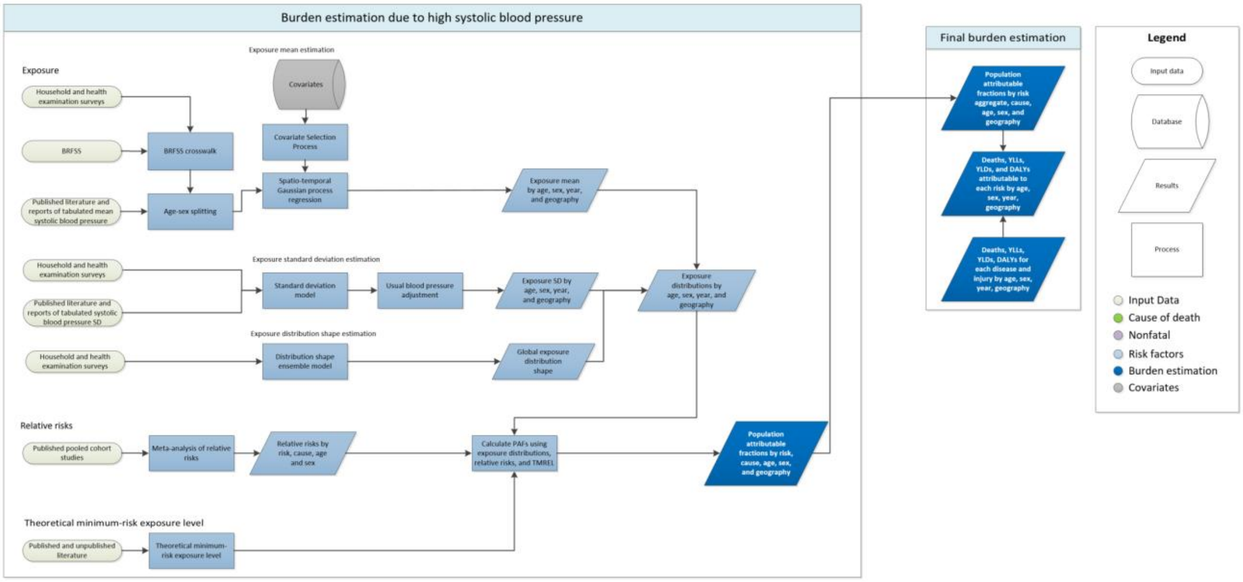

Input data and methodological summary

Exposure

Case definition

Brachial systolic blood pressure in mmHg.

Input data

We utilised data on mean systolic blood pressure from literature and from household survey microdata and reports (e.g. STEPS, NHANES). For GBD 2019, we did not carry out a systematic review of the literature for new data. Counts of the data inputs used for GBD 2019 are show in Tables 1 and 2 below. Details of inclusion and exclusion criteria and data processing steps follow.

Table 1: Data inputs for exposure for systolic blood pressure.

Input data	Exposure
Total sources	1112
Number of countries with data	166

Table 2: Data inputs for relative risks for systolic blood pressure.

Input data	Relative risk
Source count (total)	3

Inclusion criteria

Studies were included if they were population-based and directly measured systolic blood pressure using a sphygmomanometer. We assumed the data were representative if the geography or the population were not selected because it was related to hypertension or hypertensive outcomes.

Outliers

Data were utilised in the modelling process unless an assessment strongly suggested that the source was biased. A candidate source was excluded if the quality of study did not warrant a valid estimate because of selection (non-representative populations) or if the study did not provide methodological details for evaluation. In a small number of cases, a data point was considered to be an outlier candidate if the level was implausibly low or high based on expert judgement and data from other country data.

Data extraction

Where possible, individual-level data on blood pressure estimates were extracted from survey microdata. These data points were collapsed across demographic groupings to produce mean estimates in the standard GBD five-year age-sex groups. If microdata were unavailable, information from survey reports or from literature were extracted along with any available measure of uncertainty including standard error, uncertainty interval, and sample size. Standard deviations were also extracted. Where mean systolic blood pressure was reported split out by groups other than age, sex, location, and year (e.g. by hypertensive status), a weighted mean was calculated.

Incorporating United States prevalence data

Survey reports and literature often report information only about the prevalence, but not the level, of hypertension in the population studied. These sources were not used to model systolic blood pressure, with the exception of data from the Behavioral Risk Factors Surveillance System (BRFSS) because of the availability of a similarly structured exam survey that is representative of the same population (NHANES). BRFSS is a telephone survey conducted in the United States for all US counties. It collects self-reported diagnosis of hypertension. These self-reported values of prevalence of raised blood pressure were adjusted for self-report bias and tabulated by age group, sex, US state, and year. These prevalence values were used to predict a mean systolic blood pressure for the same strata with a regression using data from the National Health and Nutrition Examination Survey, a nationally representative health examination survey of the US adult population. The regression was run separately by sex, and was specified as:

$$\mathrm{SBP}_{l,a,t,s} = \beta_0 + \beta_1 \mathrm{prev}_{l,a,t,s}$$

where $\mathrm{SBP}_{\mathrm{l,a,t,s}}$ is the location, age, time, and sex specific mean systolic blood pressure and $\mathrm{prev}_{\mathrm{l,a,t,s}}$ is the location, age, time, and sex specific prevalence of raised blood pressure. The coefficients for both models are reported in Table 3.

Table 3. Coefficients in the sex-specific US states blood pressure prediction models

Term	Male model	Female model
Intercept (β_0)	114.65	108.28
Prevalence (β_1)	51.86	68.87

Out of sample RMSE was used to quantify the predictive validity of the model. The regression was repeated 10 times for each sex, each time randomly holding out 20% of the data. The RMSEs from each holdout analysis were averaged to get the average out of sample RMSE. The results of this holdout analysis are reported in Table 4.

Table 4. Out of sample RMSEs of the sex-specific US states blood pressure prediction models

	Male model	Female model
Out of sample RMSE	2.37 mmHg	3.27 mmHg

Age and sex splitting

Prior to modelling, data provided in age groups wider than the GBD five-year age groups were processed using the approach outlined in Ng and colleagues.[2] Briefly, age-sex patterns was identified using 115 sources of microdata with multiple age-sex groups, and these patterns were applied to estimate age-sex-specific levels of mean systolic blood pressure from aggregated results reported in published literature or survey reports. In order to incorporate uncertainty into this process and borrow strength across age groups when constructing the age-sex pattern, we used a model with auto-regression on the change in mean SBP over age groups:

$$\mu_a = \mu_{a-1} + \omega_a$$
$$\omega_a \sim N(\omega_{a-1}, \tau)$$

Where μ_a is the mean predicted value for age group *a*, μ_{a-1} is the mean predicted value for the age group previous to age group *a*, ω_a is the difference in mean between age group *a* and age group *a-1*, ω_{a-1} is the difference between age group *a-1* and age group *a-2*, and τ is a user-input prior on how quickly the mean SBP changes for each unit increase in age. We used a τ of 1.5 mmHg for this model. Draws of the age-sex pattern were combined with draws of the input data needing to be split in order to calculate the new variance of age-sex split data points.

Modelling

Exposure estimates were produced from 1980 to 2019 for each national and subnational location, sex, and for each five-year age group starting from 25+. As in GBD 2017, we used a spatiotemporal Gaussian process regression (ST-GPR) framework to model the mean systolic blood pressure at the location-, year-, age-, sex- level. Details of the ST-GPR method used in GBD 2019 can be found elsewhere in the appendix.

Covariate selection

The first step of the ST-GPR framework requires the creation of a linear model for predicting SBP at the location-, year-, age-, sex- level. Covariates for this model were selected in two stages. First a list of variables with an expected causal relationship with SBP was created based on significant association found within high-quality prospective cohort studies reported in the published scientific literature. The second stage in covariate selection was to test the predictive validity of every possible combination of covariates in the linear model, given the covariates selected above. This was done separately for each sex. Predictive validity was measured with out of sample root-mean-squared error.

In GBD 2016, the linear model with the lowest root-mean-squared error for each sex was then used in the ST-GPR model. Beginning in GBD 2017, we used an ensemble model of the 50 models with the lowest root-mean-squared error for each sex. This allows us to utilise covariate information from many plausible linear mixed-effects models. The 50 models were each used to predict the mean SBP for every age, sex, location, and year, and the inverse-RMSE-weighted average of this set of 50 predictions was used as the linear prior. The relative weight contributed by each covariate is plotted by sex in Figure 1.

Figure 1. Results of the ensemble linear model covariate selection

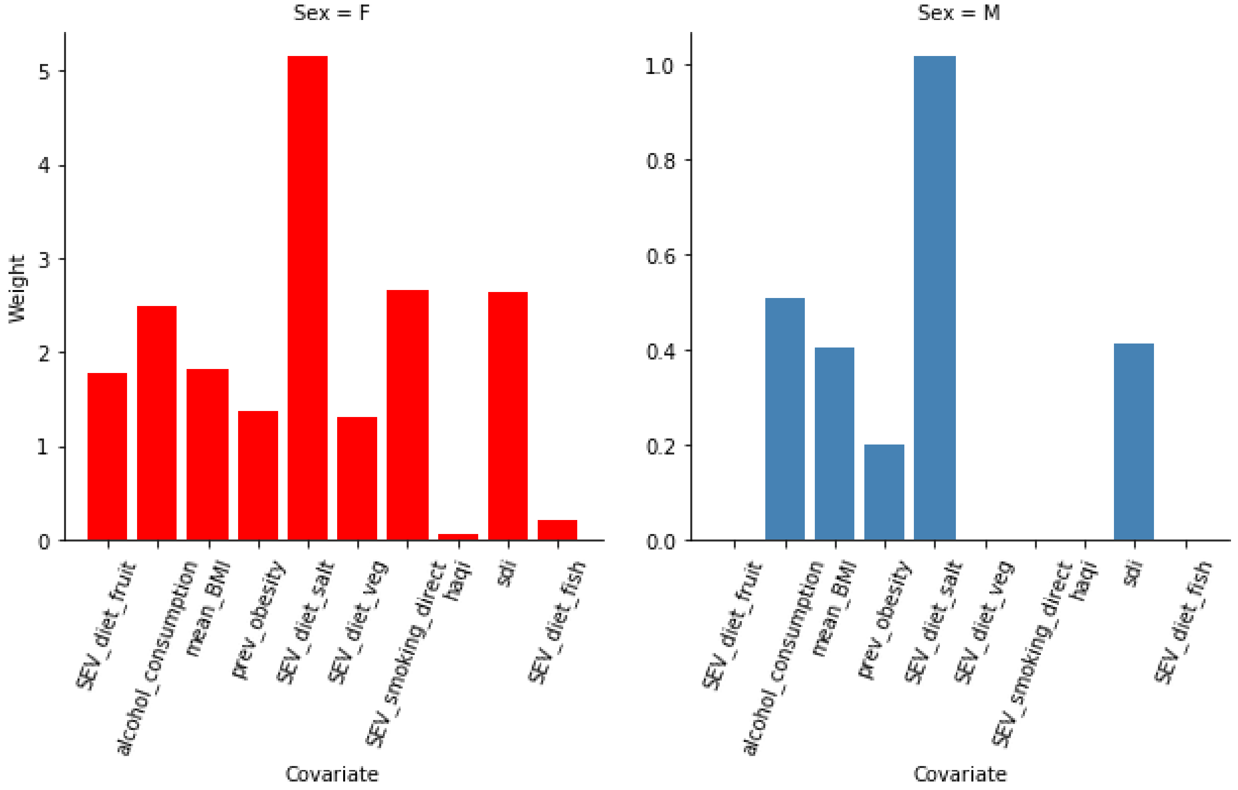

The results of the ensemble linear model were used for the first stage in an ST-GPR model. The result of the ST-GPR model are estimates of the mean SBP for each age, sex, location, and year.

Estimate of standard deviation

Currently, the ST-GPR model only produces an estimate of mean exposure level without standard deviation. Therefore, the standard deviation of systolic blood pressure within a population was estimated for each national and subnational location, sex, and five-year age group starting from age 25 using the standard deviation from person-level and some tabulated data sources. Person-level microdata accounted for 10 375 of the total 12 570 rows of data on standard deviation. The remaining 2195 rows came from tabulated data. Tabulated data were only used to model standard deviation if it was sex-specific and five-year-age-group-specific and reported a population standard deviation of systolic blood pressure. The systolic blood pressure standard deviation function was estimated using a linear regression:

$$\log(SD_{l,a,t,s}) = \beta_0 + \beta_1 \log(mean\_SBP_{l,a,t,s}) + \beta_4 sex + \sum_{k=2}^{16} \beta_k I_A$$

where $mean\_SBP_{l,a,t,s}$ is the location-, age-, time-, and sex-specific mean SBP estimate from ST-GPR, and I_A is a dummy variable for a fixed effect on a given five-year age group.

Adjustment for usual levels of blood pressure

To account for in-person variation in systolic blood pressure, a “usual blood pressure” adjustment was done. The need

for this adjustment has been described elsewhere.[5] Briefly, measurements of a risk factor taken at a single time point may not accurately capture an individual's true long-term exposure to that risk. Blood pressure readings are highly variable over time due to measurement error as well as diurnal, seasonal, or biological variation. These sources of variation result in an overestimation of the variation in cross-sectional studies of the distribution of SBP.

To adjust for this overestimation, we applied a correction factor to each location-, age-, time-, and sex-specific standard deviation. These correction factors were age-specific and represented the proportion of the variation in blood pressure within a population that would be observed if there were no within-person variation across time. Four longitudinal surveys were used to estimate these factors: the China Health and Retirement Longitudinal Survey (CHRLS), the Indonesia Family Life Survey (IFLS), the National Health and Nutrition Examination Survey I Epidemiological Follow-up Study (NHANES I/EFS), and the South Africa National Income Dynamics Survey (NIDS). The sample size and number of blood pressure measurements at each measurement period for each survey is reported in Table 5.

Table 1. Characteristics of longitudinal surveys used for the usual blood pressure adjustment

Source	Measurement periods	Number of measurements	Sample size
CHRLS	2008	3	1967
	2012	3	1419
IFLS	1997	1	19 418
	2000	1	16 626
	2007	3	14 136
NIDS	1997	2	14 084
	2000	2	9612
	2007	2	9098
NHANES I/EFS	1971–1976	2	20 716
	1982–1984	3	9932

For each survey, the following regression was created for each age group:

$$\mathrm{SBP}_{i,a} = \beta_0 + \beta_1 \mathrm{sex} + \beta_3 \mathrm{age} + +\upsilon_i$$

where $\mathrm{SBP}_{i,a}$ is the systolic blood pressure of an individual i at age a, sex is a dummy variable for the sex of an individual, age is a continuous variable for the age of an individual, and υ_i is a random intercept for each individual. Then, a blood pressure value $\widehat{\mathrm{SBP}}_{i,b}$ was predicted for each individual i for his/her age at baseline b. The correction factor cf for each age group within each survey was calculated as variation in these predicted blood pressures was divided by the variation in the observed blood pressures at baseline, $\mathrm{SBP}_{i,b}$:

$$\mathrm{cf} = \sqrt{\frac{\mathrm{var}(\widehat{\mathrm{SBP}}_b)}{\mathrm{var}(\mathrm{SBP}_b)}}$$

The average of the correction factors was taken over the three surveys to get one set of age-specific correction factors, which were then multiplied by the square of the modelled standard deviations to estimate standard deviation of the "usual blood pressure" of each age, sex, location, and year. Because of low sample sizes, the correction factors for the 75–79 age group was used for all terminal age groups. The final correction factors for each age group are reported in

Table 6. Figure 2 shows the correction factors by survey and age group ID.

Table 2. Age-specific usual blood pressure correction factors

Age group	Correction factor
25–29	0.665
30–34	0.713
35–39	0.737
40–44	0.733
45–49	0.798
50–54	0.771
55–59	0.764
60–64	0.753
65–69	0.719
70–74	0.689
75+	0.678

Figure 2: Correction factor by survey and age group id. The correction factor is equal to the variance of the predictions divided by the variance of the raw dataset. In pink is the average correction factor for each age group, summarised in Table 6.

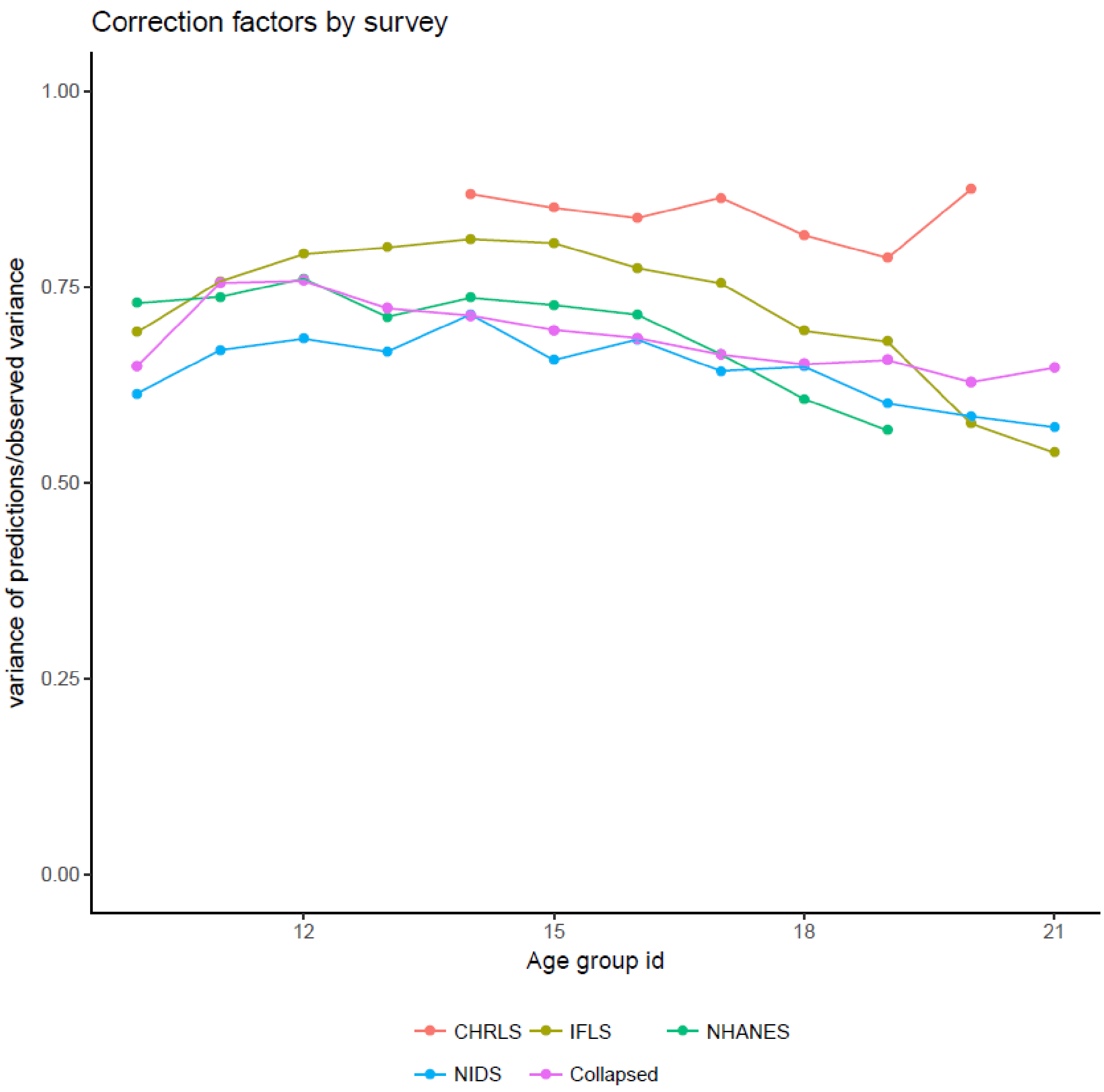

A visualisation of how the uncorrected blood pressure measurements overestimate the “usual” blood pressure variation is shown in Figure 3. This image shows the density of the distribution of the observed blood pressure values $\mathrm{SBP}_{\mathrm{i,b}}$ in participants in the Indonesian Family Life Study survey in red, and the density of the predicted blood pressure values $\widehat{\mathrm{SBP}}_{\mathrm{i,b}}$ in blue. The ratio of the variance of the blue distribution to the variance of the red distribution is an example of the scalar adjustment factor being applied to the modelled standard deviations.

Figure 3: Raw and predicted distributions of blood pressure in the Indonesia Family Life Survey

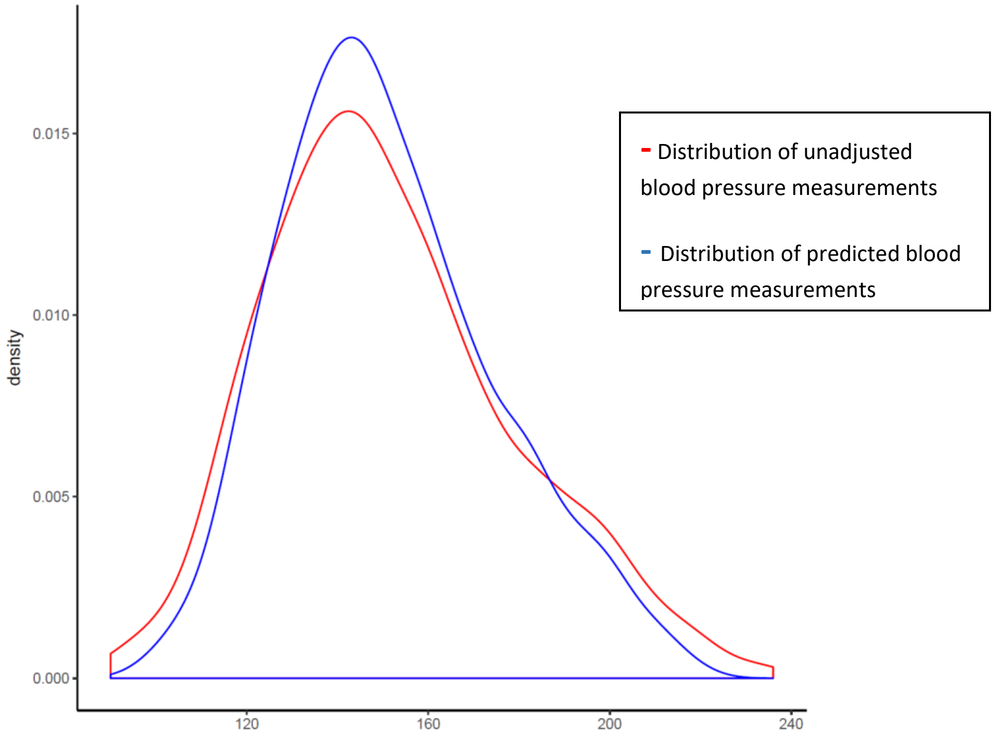

Estimating the exposure distribution shape

The shape of the distribution of systolic blood pressure was estimated using all available person-level microdata sources, which was a subset of the input data into the modelling process. The distribution shape modelling framework for GBD 2019 is detailed in the elsewhere in the appendix. Briefly, an ensemble distribution created from a weighted average of distribution families was fit for each individual microdata source, separately by sex. The weights for the distribution families for each individual source were then averaged and weighted to create a global ensemble distribution for each sex.

Theoretical minimum-risk exposure level

No changes have been made to the TMREL used for systolic blood pressure since GBD 2015. We estimated that the TMREL of SBP ranges from 110 to 115 mmHg based on pooled prospective cohort studies that show risk of mortality increases for SBP above that level.[3,4] Our selection of a TMREL of 110–115 mmHg is consistent with the GBD study approach of estimating all attributable health loss that could be prevented even if current interventions do not exist that can achieve such a change in exposure level, for example a tobacco smoking prevalence of zero percent. To include the uncertainty in the TMREL, we took a random draw from the uniform distribution of the interval between 110 mmHg and 115 mmHg each time the population attributable burden was calculated.

Relative risks

No changes have been made to the relative risk estimates for blood pressure outcomes used since GBD 2016. RRs for chronic kidney disease are from the Renal Risk Collaboration meta-analysis of 2.7 million individuals in 106 cohorts. For other outcomes, we used data from two pooled epidemiological studies: the Asia Pacific Cohort Studies Collaboration (APCSC) and the Prospective Studies Collaboration (PSC).[4,5] Additional estimates of RR for cardiovascular outcomes were used from the CALIBER study, a health-record linkage cohort study from the UK.[6]

For cardiovascular disease, epidemiological studies have shown that the RR associated with SBP declines with age, with the log (RR) having an approximately linear relationship with age and reaching a value of 1 between the ages of 100 and

120. RRs were reported per 10 mmHg increase in SBP above the TMREL value (115 mmHg), calculated as in the equation below:

$$RR(x) = RR_0^{\frac{(x-TMREL)}{10\ mmHg}}$$

Where $RR(x)$ is the RR at exposure level x and RR_0 is the increase in RR for each 10 mmHg above the TMREL. We used DisMod-MR 2.1 to pool effect sizes from included studies and generate a dose-response curve for each of the outcomes associated with high SBP. The tool enabled us to incorporate random effects across studies and include data with different age ranges. RRs were used universally for all countries and the meta-regression only helped to pool the three major sources and produce RRs with uncertainty and covariance across ages taking into account the uncertainty of the data points.

References

1 Bangalore S, Gong Y, Cooper-DeHoff RM, Pepine CJ, Messerli FH. 2014 Eighth Joint National Committee panel recommendation for blood pressure targets revisited: results from the INVEST study. *J Am Coll Cardiol* 2014; **64**: 784–93.

2 Ng M, Fleming T, Robinson M, *et al.* Global, regional, and national prevalence of overweight and obesity in children and adults during 1980–2013: a systematic analysis for the Global Burden of Disease Study 2013. *The Lancet* 2014; **384**: 766–81.

3 Singh GM, Danaei G, Farzadfar F, *et al.* The age-specific quantitative effects of metabolic risk factors on cardiovascular diseases and diabetes: a pooled analysis. *PloS One* 2013; **8**: e65174.

4 Collaboration APCS, others. Blood pressure and cardiovascular disease in the Asia Pacific region. *J Hypertens* 2003; **21**: 707–16.

5 Prospective Studies Collaboration. Age-specific relevance of usual blood pressure to vascular mortality: a meta-analysis of individual data for one million adults in 61 prospective studies. *The Lancet* 2002; **360**: 1903–13.

6 Rapsomaniki E, Timmis A, George J, *et al.* Blood pressure and incidence of twelve cardiovascular diseases: lifetime risks, healthy life-years lost, and age-specific associations in 1·25 million people. *Lancet Lond Engl* 2014; **383**: 1899–911.

Kidney dysfunction

Flowchart

Kidney dysfunction

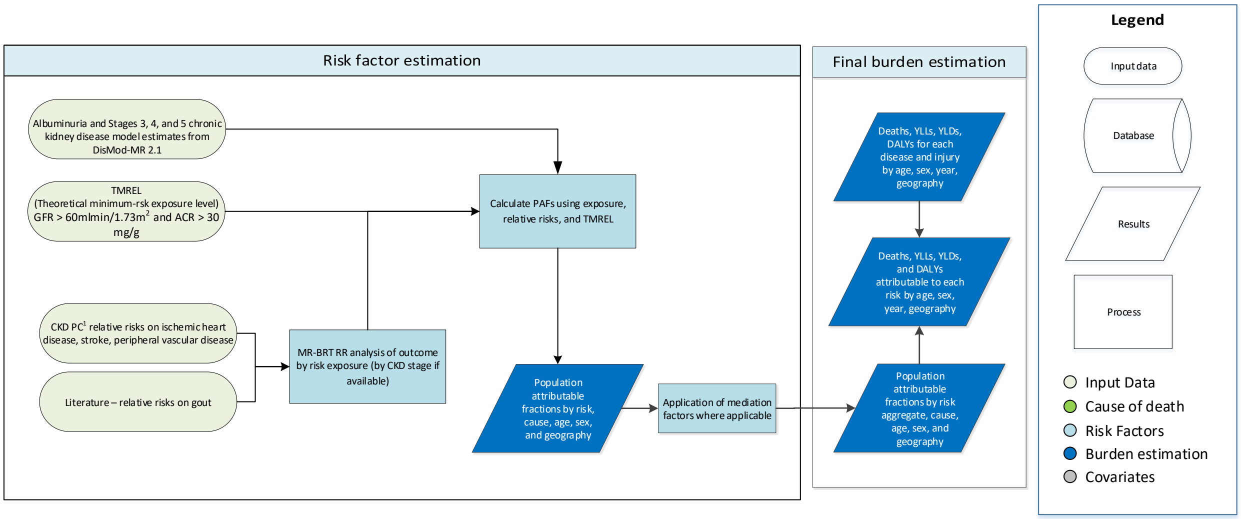

1. The Chronic Kidney Disease Prognosis Consortium is a research group composed of investigators representing cohorts from around the world. Investigators share data for the purpose of collaborative meta-analyses to study prognosis in CKD.

Input data and methodological summary

Exposure

Case definition

The kidney dysfunction risk factor exposure is divided into four categories of renal function defined by urinary albumin to creatinine ratio (ACR) and estimated glomerular filtration rate (eGFR):

- Albuminuria with preserved eGFR (ACR >30 mg/g & eGFR >=60 ml/min/1.73m^2); this corresponds to stages 1 and 2 chronic kidney disease (CKD) in the Kidney Disease Improving Global Outcomes (KDIGO) classification
- CKD stage 3 (eGFR of 30-59 ml/min/1.73m^2);
- CKD stage 4 (eGFR of 15-29 ml/min/1.73m^2); and
- CKD stage 5 (eGFR <15ml/min/1.73m^2, not (yet) on renal replacement therapy).

The modelling of renal function prevalence estimates is described in detail in the CKD section of the appendix to the GBD 2019 disease and injury paper.

Theoretical minimum-risk exposure level

The theoretical minimum-risk exposure level is ACR 30 mg/g or less and eGFR greater than 60ml/min/1.73m^2. An ACR above 30 mg/g and eGFR below 60ml/min/1.73m^2 have been demonstrated in the literature to be the thresholds at which increased cardiovascular and gout events occur secondary to kidney dysfunction.(1-10)

Input data

The last systematic review of prevalence of low glomerular filtration rate was conducted for GBD 2016, updating searches done in GBD 2015, GBD 2013, and GBD 2010. Exclusion criteria included surveys that were not population-representative and studies not reporting on CKD by stage.

Data sources for kidney dysfunction:

Input data	Exposure
Source count (total)	98

Number of countries with data	35

Input data	Relative risk
Source count (total)	9

Modelling strategy

We model the proportion of cardiovascular and musculoskeletal diseases attributable to kidney dysfunction. This is performed by 1) running DisMod-MR 2.1 models to estimate the prevalence of albuminuria, stage 3 CKD, stage 4 CKD, and stage 5 CKD; 2) estimate relative risks from available data on cardiovascular outcomes and gout; 3) calculate the population attributable fraction of those outcomes to IKF.

The prevalence of exposure to albuminuria and CKD were obtained from the GBD 2019 non-fatal burden of disease analysis.

Data on relative risks were contributed by the Chronic Kidney Disease Prognosis Consortium (CKD-PC). The Chronic Kidney Disease Prognosis Consortium is a research group composed of investigators representing cohorts from around the world. Investigators share data for the purpose of collaborative meta-analyses to study prognosis in CKD.

Relative risks

We estimate burden attributable to kidney dysfunction for cardiovascular diseases, chronic kidney diseases, and gout.

In GBD 2017, we relied on a pooled cohort analysis of six cohort studies from the CKD-PC. For GBD 2019, in collaboration with CKD-PC, we got data on 38 new cohorts and continued to use the original from the previous analysis. We ran these new data through MR-BRT meta-regression to determine the relationship between age and outcomes based on exposure to IKF. Estimates were nested within cohorts. A three-degree spline was placed on age with decreasing monotonicity. All relative risk estimates for stroke and ischaemic heart disease above age 85 were set equal to the risk at age 85 to control for lack of data in older age groups. Gout currently uses GBD 2017 estimations of relative risk.

We ran some sensitivity analyses with and without controlling for blood pressure. This is because IKF increases the risk of cardiovascular diseases directly, as well as through blood pressure. We wanted to understand how estimates of risk would differ. Generally, the relative risk of cardiovascular disease was lower when controlling for blood pressure. We decided to go with this lower risk that controlled for hypertension for a more conservative estimate.

Relative risk plots

The following plot shows the relative risks for heart disease and stroke by each stage of CKD. As expected, stage 5 and stage 4 CKD have higher risks overall. Risks is also higher at younger ages and lower at the oldest age, likely reflecting competing risk factors. While the risks themselves dip below zero at the oldest age, we believe this is merely a function of lack of data above age 85. Because of this, our estimates for relative risk above age 85 take the estimate at age 85.

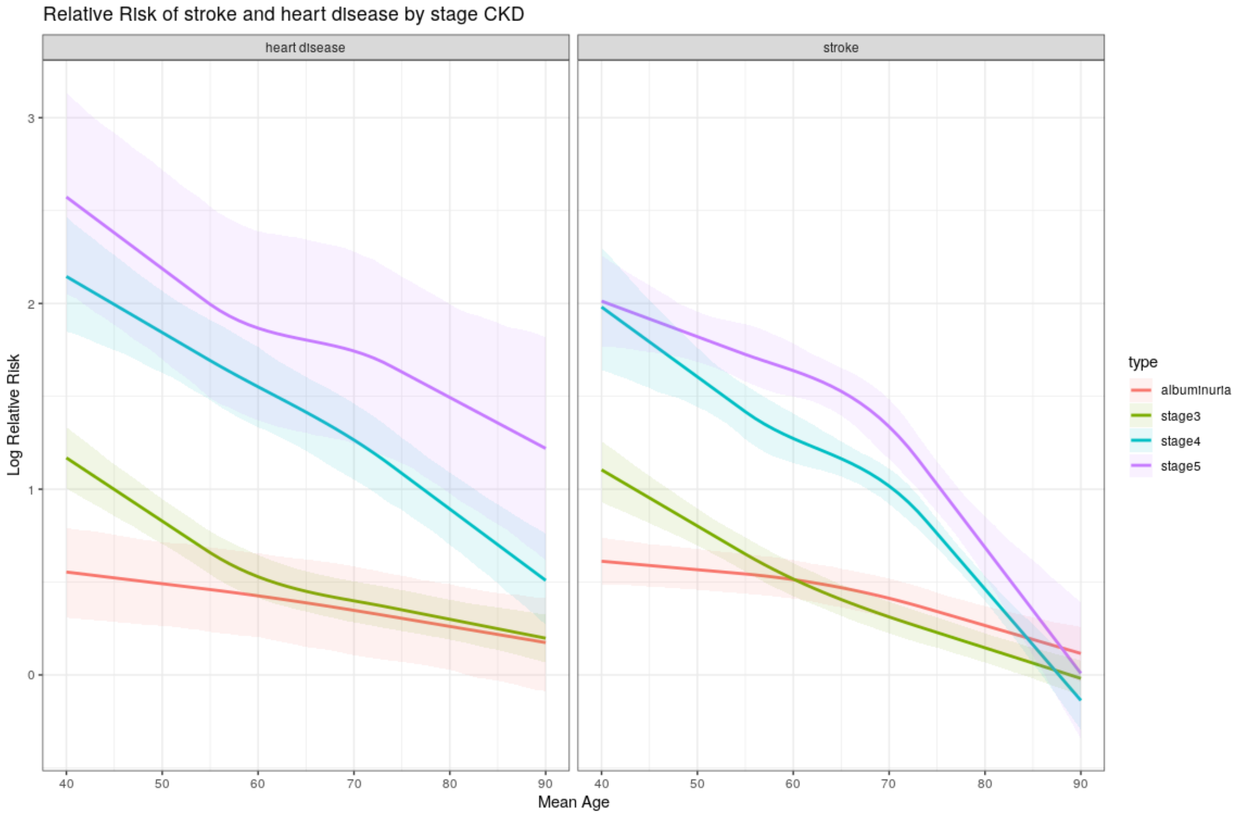

We also include two forest plots to show the distribution of risk estimates for heart disease and stroke across our studies. In general, we see an expected pattern, with earlier stages of CKD with lower risks.

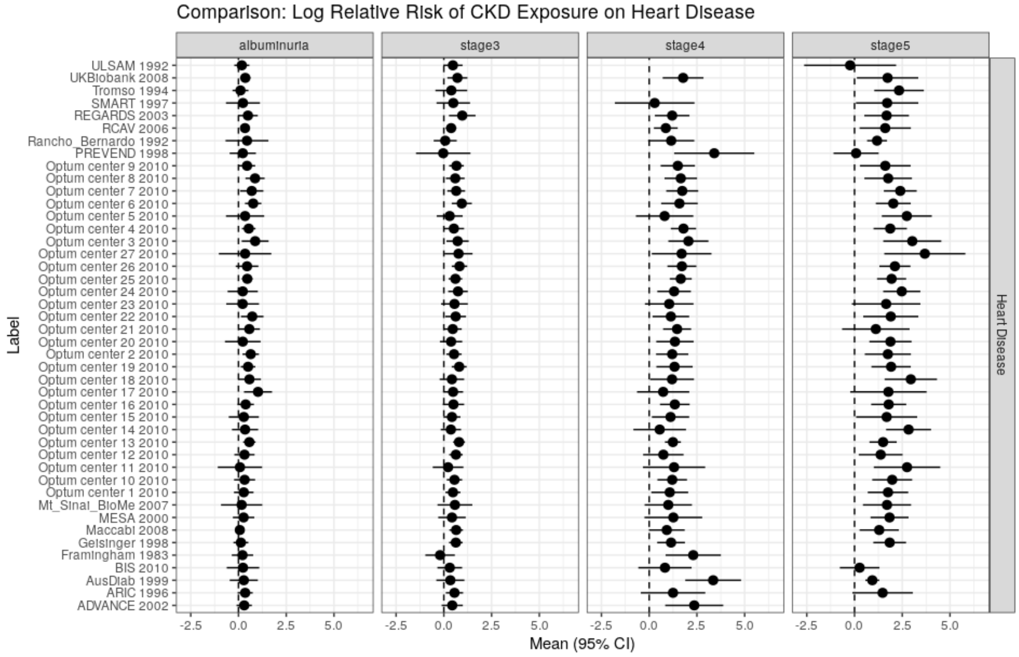

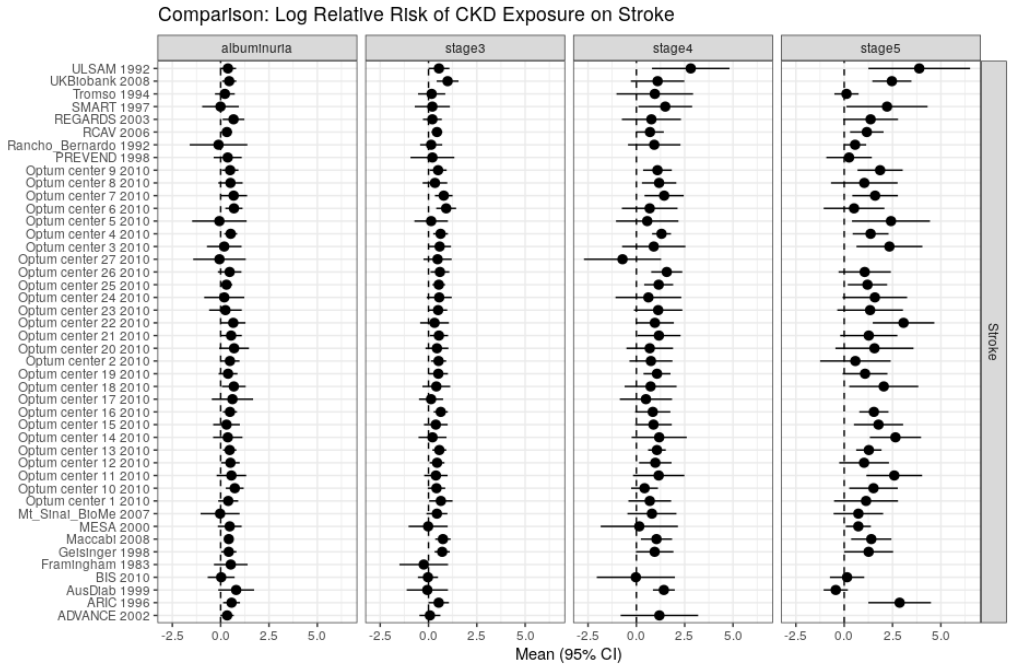

Population attributable fraction

We calculated the cardiovascular and gout fatal and non-fatal burden attributable to the categorical exposure to kidney dysfunction using the following equation:

$$PAF = \frac{\sum_{i=1}^{n} P_i(RR_i - 1)}{\sum_{i=1}^{n} P_i(RR_i - 1) + 1}$$

Equation 1. PAF based on categorical exposure

where RR_i is the relative risk for exposure level *i*, P_i is the proportion of the population in that exposure category, and *n* is the number of exposure categories.(11)

Primary changes between GBD 2017 and GBD 2019

The following are the main changes in the GBD 2019 modelling strategy compared to GBD 2017:

1. In GBD 2019, we used MR-BRT to run a nested meta-regression analysis on the within-study sex ratios to estimate a pooled sex ratio with 95% confidence intervals. In GBD 2017, this was estimated in DisMod-MR 2.1.
2. In GBD 2019, we used MR-BRT to make bias adjustments for data with alternative case definitions. CKD uses CKD-Epi as the reference definition. Alternative equations include the Cockcroft-Gualt and Modification of Diet in Renal Disease equations. MR-BRT models have larger confidence intervals due to taking into account study variance across all input data. In GBD 2017, these adjustments were made in DisMod-MR 2.1. The values of these adjustments are in the table below:

MR-BRT bias adjustment factors

Data input	Status	*Gamma*	*Beta coefficient, logit (95% CI)*	*Adjustment factor**
CKD-EPI	Ref	---	---	---

Stage III CG	Alt	*0.25*	0.24<br>(-0.28 to 0.76)	0.56<br>(0.43–0.68)
Stage III MDRD	Alt	*0.03*	0.49<br>(0.34–0.64)	0.62<br>(0.58–0.66)
Stage IV CG	Alt	*0*	0.09<br>(-0.05 to 0.24)	0.52<br>(0.49–0.56)
Stage IV MDRD	Alt	*0*	-0.07<br>(-0.19 to 0.04)	0.48<br>(0.45–0.51)
Stage V CG	Alt	*0*	-0.18<br>(-0.45 to 0.09)	0.45<br>(0.39–0.52)
Stage V MDRD	Alt	*0*	-0.06<br>(-0.28 to 0.18)	0.49<br>(0.43–0.54)
Stage III-V CG	Alt	*0.26*	0.23<br>(-0.29 to 0.75)	0.56<br>(0.43–0.68)
Stage III-V MDRD	Alt	*0.03*	0.47<br>(0.32–0.62)	0.62<br>(0.58–0.65)

3. In GBD 2017, the RRs were estimated via a pooled cohort meta-regression conducted in R using the metafor package. In GBD 2019, we made use of MR-BRT to run a nested meta-regression analysis that allowed more flexibility in the estimation process.

References

1. Go AS, Chertow GM, Fan D, McCulloch CE, Hsu CY. Chronic kidney disease and the risks of death, cardiovascular events, and hospitalization. N Engl J Med. 2004;351(13):1296-305.
2. Ninomiya T, Kiyohara Y, Kubo M, Tanizaki Y, Doi Y, Okubo K, et al. Chronic kidney disease and cardiovascular disease in a general Japanese population: the Hisayama Study. Kidney international. 2005;68(1):228-36.
3. Shara NM, Wang H, Mete M, Al-Balha YR, Azalddin N, Lee ET, et al. Estimated GFR and incident cardiovascular disease events in American Indians: the Strong Heart Study. American journal of kidney diseases : the official journal of the National Kidney Foundation. 2012;60(5):795-803.
4. Mann JF, Gerstein HC, Pogue J, Bosch J, Yusuf S. Renal insufficiency as a predictor of cardiovascular outcomes and the impact of ramipril: the HOPE randomized trial. Annals of internal medicine. 2001;134(8):629-36.
5. Chronic Kidney Disease Prognosis C, Matsushita K, van der Velde M, Astor BC, Woodward M, Levey AS, et al. Association of estimated glomerular filtration rate and albuminuria with all-cause and cardiovascular mortality in general population cohorts: a collaborative meta-analysis. Lancet. 2010;375(9731):2073-81.
6. De Graauw J, Chonchol M, Poppert H, Etgen T, Sander D. Relationship between kidney function and risk of asymptomatic peripheral arterial disease in elderly subjects. Nephrology, dialysis, transplantation : official publication of the European Dialysis and Transplant Association - European Renal Association. 2011;26(3):927-32.
7. Wattanakit K, Folsom AR, Selvin E, Coresh J, Hirsch AT, Weatherley BD. Kidney function and risk of peripheral arterial disease: results from the Atherosclerosis Risk in Communities (ARIC) Study. Journal of the American Society of Nephrology : JASN. 2007;18(2):629-36.
8. O'Hare AM, Vittinghoff E, Hsia J, Shlipak MG. Renal insufficiency and the risk of lower extremity peripheral arterial disease: results from the Heart and Estrogen/Progestin Replacement Study (HERS). Journal of the American Society of Nephrology : JASN. 2004;15(4):1046-51.
9. Manjunath G, Tighiouart H, Coresh J, Macleod B, Salem DN, Griffith JL, et al. Level of kidney function as a risk factor for cardiovascular outcomes in the elderly. Kidney international. 2003;63(3):1121-9.

10. Manjunath G, Tighiouart H, Ibrahim H, MacLeod B, Salem DN, Griffith JL, et al. Level of kidney function as a risk factor for atherosclerotic cardiovascular outcomes in the community. Journal of the American College of Cardiology. 2003;41(1):47-55.
11. Miettinen OS. Proportion of disease caused or prevented by a given exposure, trait or intervention. American journal of epidemiology. 1974;99(5):325-32.

Dietary risks

Flowchart

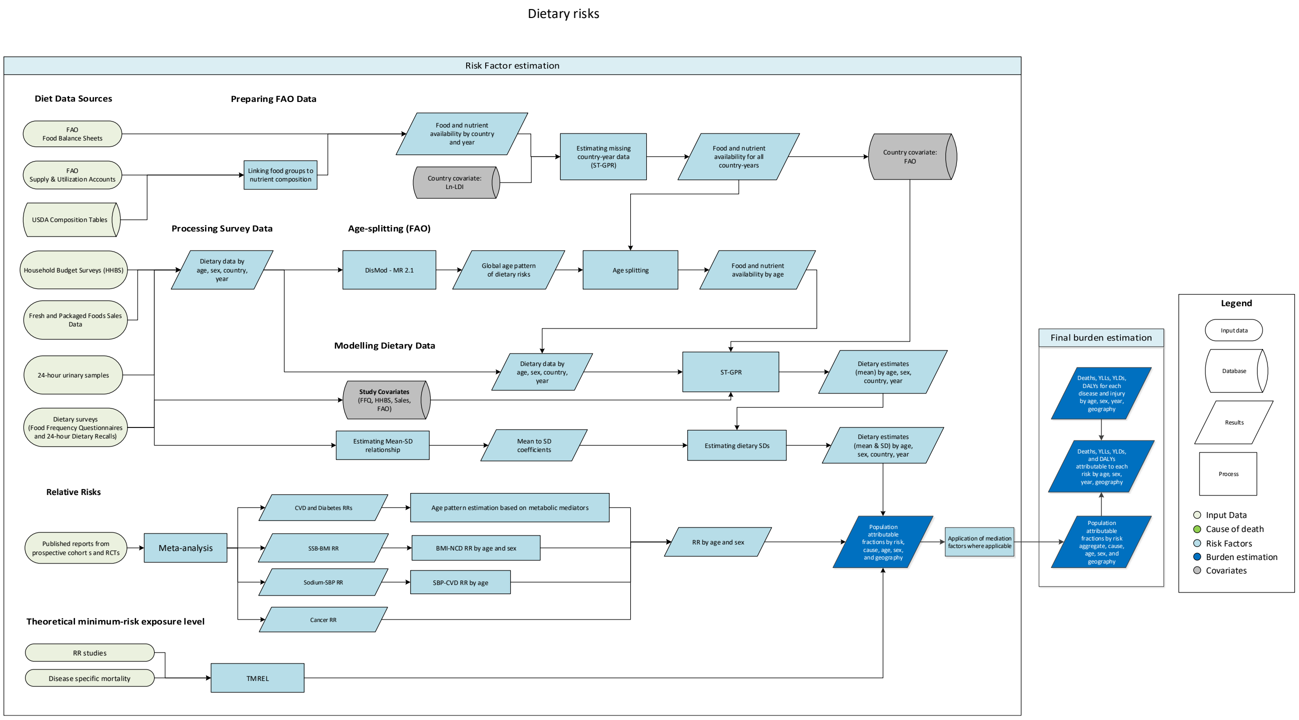

Input data and methodological summary

Definition

Exposure

Risk	Definition
**Diet low in fruit**	Average daily consumption (in grams per day) of less than 310-340 grams of fruit including fresh, frozen, cooked, canned, or dried fruit, excluding fruit juices and salted or pickled fruits
**Diet low in vegetables**	Average daily consumption (in grams per day) of less than 280-320 grams of vegetables, including fresh, frozen, cooked, canned, or dried vegetables and excluding legumes and salted or pickled vegetables, juices, nuts and seeds, and starchy vegetables such as potatoes or corn

**Diet low in whole grains**	Average daily consumption (in grams per day) of less than 140-160 grams of whole grains (bran, germ, and endosperm in their natural proportion) from breakfast cereals, bread, rice, pasta, biscuits, muffins, tortillas, pancakes, and other sources
**Diet low in nuts and seeds**	Average daily consumption (in grams per day) of less than 10-19 grams of nuts and seeds, including tree nuts and seeds and peanuts
**Diet low in fibre**	Average daily consumption (in grams per day) of less than 21-22 grams of fibre from all sources including fruits, vegetables, grains, legumes, and pulses
**Diet low in omega-3 fatty acids**	Average daily consumption (in milligrams per day) of less than 430-470 milligrams of eicosapentaenoic acid (EPA) and docosahexaenoic acid (DHA)
**Diet low in polyunsaturated fatty acids (PUFA)**	Average daily consumption (in % daily energy) of less than 7-9% total energy intake from polyunsaturated fatty acids
**Diet low in calcium**	Average daily consumption (in grams per day) of less than 1.06-1.1 grams of calcium from all sources, including milk, yogurt, and cheese
**Diet low in milk**	Average daily consumption (in grams per day) of less than 360-500 grams of milk including non-fat, low-fat, and full-fat milk, excluding soy milk and other plant derivatives
**Diet low in legumes**	Average daily consumption (in grams per day) of less than of 90-100 grams of legumes and pulses, including fresh, frozen, cooked, canned, or dried legumes
**Diet high in red meat**	Any intake (in grams per day) of red meat including beef, pork, lamb, and goat but excluding poultry, fish, eggs, and all processed meats
**Diet high in processed meat**	Any intake (in grams per day) of meat preserved by smoking, curing, salting, or addition of chemical preservatives
**Diet high in sugar-sweetened beverages (SSBs)**	Any intake (in grams per day) of beverages with ≥50 kcal per 226.8 gram serving, including carbonated beverages, sodas, energy drinks, fruit drinks, but excluding 100% fruit and vegetable juices
**Diet high in trans fatty acids**	Any intake (in percent daily energy) of trans fat from all sources, mainly partially hydrogenated vegetable oils and ruminant products
**Diet high in sodium**	Average 24-hour urinary sodium excretion (in grams per day) greater than 1-5 grams

Input data

In GBD 2019, we included new dietary recall sources from a literature search of PubMed and new sources from the IHME GHDx yearly known survey series updates in our models. We also conducted a new systematic review for sodium (Figure 1). As in GBD 2017, the dietary data that we use in the models comes from multiple sources, including nationally

and subnationally representative nutrition surveys, household budget surveys, accounts of national sales from the Euromonitor, and availability data from the United Nations FAO Supply and Utilization Accounts (SUA). Table 1 below provides a summary of data inputs used for dietary risk modeling in GBD 2019.

Figure 1: PRISMA diagram for sodium intake data systematic review

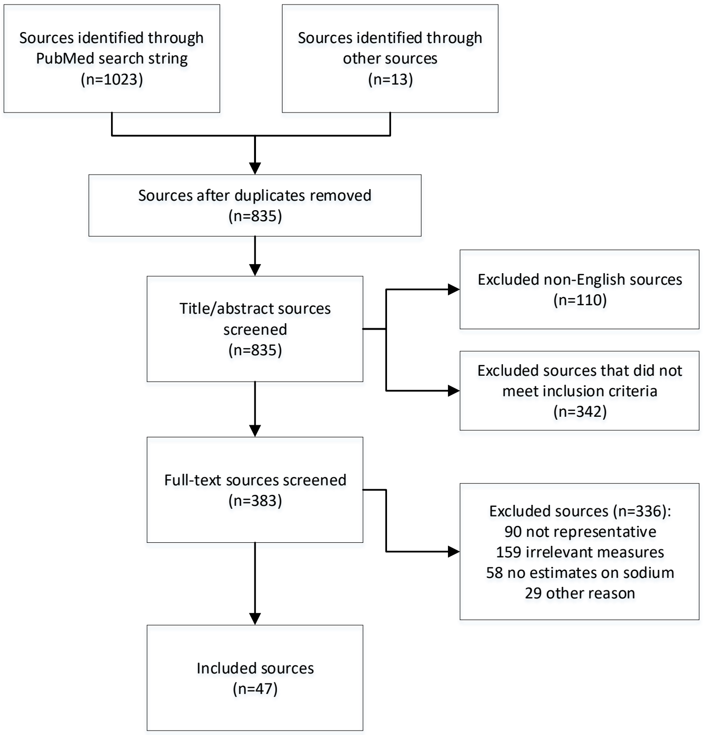

Table 1a: Data inputs for exposure for dietary risk factors.

Dietary risk factor	Total exposure sources	Countries with data
All dietary risks	1461	195
Calcium	160	178
Fiber	155	180
Fruit	869	180
Legumes	683	169
Milk	1148	177
Nuts and seeds	100	158
Omega 3	20	178
Processed meat	737	66

PUFA	70	180
Red meat	760	178
Sodium	92	53
SSBs	720	66
Trans fat	924	72
Vegetables	871	180
Whole grains	52	188

Table 1b: Data inputs for risk analysis for dietary risk factors.

Dietary risk factor	Total relative risk sources	Countries with data
Calcium	37	9
Fiber	64	16
Fruit	116	23
Legumes	10	5
Milk	12	8
Nuts and seeds	23	9
Omega 3	50	16
Processed meat	41	11
PUFA	18	8
Red meat	92	20
Sodium	21	6
SSBs	15	5
Transfat	10	5
Vegetable	39	11
Whole grains	37	9

The availability data for food groups in GBD were previously based on the FAO Food Balance Sheets (FBS), which provide tabulated and processed data of national food supply. In GBD 2019, to more accurately characterise the national availability of various food groups, we used more disaggregated data on food commodities that were included in FAO SUA and recreated the national availability of each food group based on the GBD definition of the food group. We

modelled missing country-year data from FAO using a spatiotemporal Gaussian process regression and lag-distributed country income as the covariate. For nutrient availability, we continued to use data from Global Nutrient Database.[1]

For each dietary factor, we estimated the global age pattern of consumption based on nutrition surveys (ie, 24-hour diet recall) and applied that age pattern to the all-age data (availability, sales and household budget surveys) before the data source bias adjustment.

Our gold-standard data source for all dietary risks (except sodium) is 24-hour dietary recall surveys where food and nutrient intake are reported or convertible to grams per person per day; the gold-standard data source for sodium is 24-hour urinary sodium. The other data sources we use – household budget surveys, food frequency questionnaires, sales, and availability – are treated as alternate definitions for dietary intake and crosswalked to the gold-standard definition. In GBD 2016 and GBD 2017, we determined the bias adjustment factors from a mixed effects linear regression. In GBD 2019, we used MR-BRT (a network meta-regression) to determine the adjustment factors for non-gold-standard datapoints. Coefficients for these models can be found in Table 3.

Table 2. Types of data sources (other than 24-hour dietary recall) and covariates used in modelling of each dietary factor.

	Data sources				Country-level covariate
	Sales	FFQ[1]	HBS[2]	FAO	
Diet low in fruits	■	■	■	■	Lag distributed income
Diet low in vegetables	■	■	■	■	Energy availability (kcal)
Diet low in whole grains	-	■	-	■	Energy availability (kcal)
Diet low in nuts and seeds	-	-	■	■	Energy availability (kcal)
Diet low in milk	■	■	■	■	Energy availability (kcal)
Diet high in red meat	■	■	■	■	Energy availability (kcal)
Diet high in processed meat	■	■	■	-	Energy availability (kcal), pigs per capita
Diet low in legumes	■	■	-	■	Energy availability (kcal)
Diet high in sugar-sweetened beverages	■	■	■	-	Energy availability (kcal), availability of sugar
Diet low in fibre	-	■	-	■	Energy availability (kcal)
Diet suboptimal in calcium	-	■	-	■	Energy availability (kcal)
Diet low in seafood omega-3 fatty acids	-	-	-	■	Lag distributed income, proportion landlocked area
Diet low in polyunsaturated fatty acids	-	■	-	■	Lag distributed income
Diet high in trans fatty acids	■	■	-	-	
Diet high in sodium[3]	-	-	-	-	

[1] Food Frequency Questionnaire
[2] Household Budge Survey
[3] For sodium, we used data from the 24-hour urinary sodium and 24-hour dietary recall.

Table 3: MR-BRT crosswalk adjustment factors for all dietary risks

Dietary risk	Sex	Data input	Reference or alternative case definition	Gamma	Beta coefficient, log (95% CI)	Adjustment factor*
Calcium	---	DR	Ref	0.24	---	---
Calcium	Female	FAO	Alt		0.04 (0.04, 0.5)	0.96 (0.64, 1.65)
Calcium	Female	FFQ	Alt		-0.04 (-0.04, 0.43)	1.04 (0.59, 1.53)
Calcium	Male	FAO	Alt		0.17 (0.17, 0.63)	0.84 (0.73, 1.88)
Calcium	Male	FFQ	Alt		0.09 (0.09, 0.55)	0.91 (0.67, 1.74)
Fibre	---	DR	Ref	0.33	---	---
Fibre	Female	FAO	Alt		0.56 (0.56, 1.17)	0.57 (0.93, 3.23)
Fibre	Female	FFQ	Alt		0.27 (0.27, 0.88)	0.76 (0.69, 2.41)
Fibre	Male	FAO	Alt		0.55 (0.55, 1.17)	0.57 (0.92, 3.22)
Fibre	Male	FFQ	Alt		0.26 (0.26, 0.88)	0.77 (0.69, 2.4)
Fruit	---	DR	Ref	0.76	---	---
Fruit	Female	FAO	Alt		0.36 (0.36, 1.83)	0.7 (0.31, 6.21)
Fruit	Female	Sales	Alt		0.73 (0.73, 2.19)	0.48 (0.45, 8.98)
Fruit	Female	FFQ	Alt		-0.15 (-0.15, 1.32)	1.17 (0.19, 3.73)
Fruit	Female	HHBS	Alt		0.23 (0.23, 1.71)	0.79 (0.27, 5.5)
Fruit	Male	FAO	Alt		0.32 (0.32, 1.79)	0.73 (0.3, 5.97)
Fruit	Male	Sales	Alt		0.69 (0.69, 2.16)	0.5 (0.43, 8.64)
Fruit	Male	FFQ	Alt		-0.19 (-0.19, 1.28)	1.21 (0.18, 3.58)
Fruit	Male	HHBS	Alt		0.19 (0.19, 1.66)	0.83 (0.26, 5.27)
Legumes	---	DR	Ref	0.74	---	---
Legumes	Female	FAO	Alt		-0.08 (-1.49,1.39)	1.08 (0.22,4)
Legumes	Female	Sales	Alt		-0.9 (-2.31,0.56)	2.47 (0.1,1.75)
Legumes	Female	FFQ	Alt		-0.53 (-1.94,0.95)	1.7 (0.14,2.58)

Legumes	Male	FAO	Alt		0.06 (-1.35,1.53)	0.94 (0.26,4.61)
Legumes	Male	Sales	Alt		-0.76 (-2.16,0.7)	2.14 (0.12,2.01)
Legumes	Male	FFQ	Alt		-0.39 (-1.79,1.09)	1.47 (0.17,2.98)
Milk	---	DR	Ref		---	---
Milk	Female	FAO	Alt		0.27 (0.27, 2.57)	0.76 (0.16, 13.01)
Milk	Female	Sales	Alt		0.01 (0.01, 2.31)	0.99 (0.13, 10.11)
Milk	Female	FFQ	Alt		0.46 (0.46, 2.78)	0.63 (0.18, 16.2)
Milk	Female	HHBS	Alt	1.06	-0.61 (-0.61, 1.69)	1.84 (0.07, 5.4)
Milk	Male	FAO	Alt		0.28 (0.28, 2.58)	0.75 (0.17, 13.17)
Milk	Male	Sales	Alt		0.03 (0.03, 2.33)	0.97 (0.13, 10.23)
Milk	Male	FFQ	Alt		0.48 (0.48, 2.8)	0.62 (0.18, 16.43)
Milk	Male	HHBS	Alt		-0.59 (-0.59, 1.7)	1.81 (0.07, 5.48)
Nuts	---	DR	Ref		---	---
Nuts	Female	FAO	Alt		0.49 (0.49, 3.63)	0.62 (0.06, 37.68)
Nuts	Female	FFQ	Alt		-0.34 (-0.34, 2.76)	1.41 (0.02, 15.75)
Nuts	Female	HHBS	Alt	1.58	-0.72 (-0.72, 2.42)	2.06 (0.02, 11.27)
Nuts	Male	FAO	Alt		0.6 (0.6, 3.73)	0.55 (0.07, 41.65)
Nuts	Male	FFQ	Alt		-0.23 (-0.23, 2.87)	1.26 (0.03, 17.58)
Nuts	Male	HHBS	Alt		-0.62 (-0.62, 2.54)	1.85 (0.02, 12.66)
Omega-3	---	DR	Ref		---	---
Omega-3	Male	FAO	Alt	0.12	-1.15 (-1.15, -0.92)	3.16 (0.25, 0.4)
Omega-3	Female	FAO	Alt		-1.01 (-1.01, -0.78)	2.75 (0.29, 0.46)
Proc. meat	---	DR	Ref		---	---
Proc. meat	Female	Sales	Alt		0.79 (0.79, 3.14)	0.46 (0.19, 23.07)
Proc. meat	Female	FFQ	Alt		-0.3 (-0.3, 2.25)	1.35 (0.05, 9.49)
Proc. meat	Female	HHBS	Alt	1.21	-0.46 (-0.46, 1.89)	1.59 (0.05, 6.63)
Proc. meat	Male	Sales	Alt		0.95 (0.95, 3.3)	0.39 (0.22, 27.03)
Proc. meat	Male	FFQ	Alt		-0.13 (-0.13, 2.42)	1.14 (0.06, 11.2)
Proc. meat	Male	HHBS	Alt		-0.3 (-0.3, 2.06)	1.35 (0.06, 7.82)

PUFA	---	DR	Ref	0.14	---	---
PUFA	Female	FAO	Alt		-0.14 (-0.14, 0.14)	1.15 (0.65, 1.15)
PUFA	Female	FFQ	Alt		1.05 (1.05, 1.43)	0.35 (1.96, 4.18)
PUFA	Male	FAO	Alt		-0.18 (-0.18, 0.1)	1.2 (0.62, 1.1)
PUFA	Male	FFQ	Alt		1 (1, 1.38)	0.37 (1.87, 3.98)
Red meat	---	DR	Ref	0.83	---	---
Red meat	Female	FAO	Alt		0.89 (0.89, 2.54)	0.41 (0.45, 12.69)
Red meat	Female	Sales	Alt		1.09 (1.09, 2.74)	0.34 (0.54, 15.49)
Red meat	Female	FFQ	Alt		-0.34 (-0.34, 1.6)	1.4 (0.11, 4.95)
Red meat	Female	HHBS	Alt		0.45 (0.45, 2.1)	0.64 (0.29, 8.18)
Red meat	Male	FAO	Alt		0.89 (0.89, 2.54)	0.41 (0.45, 12.66)
Red meat	Male	Sales	Alt		1.09 (1.09, 2.74)	0.34 (0.54, 15.43)
Red meat	Male	FFQ	Alt		-0.34 (-0.34, 1.6)	1.4 (0.11, 4.94)
Red meat	Male	HHBS	Alt		0.45 (0.45, 2.1)	0.64 (0.29, 8.15)
Sodium	---	Urinary sodium	Ref	0.39	---	---
Sodium	Female	DR	Alt		-0.02 (-0.02, 0.85)	1.02 (0.38, 2.34)
Sodium	Female	FFQ	Alt		0.47 (0.47, 1.29)	0.63 (0.69, 3.64)
Sodium	Male	DR	Alt		-0.06 (-0.06, 0.8)	1.06 (0.38, 2.23)
Sodium	Male	FFQ	Alt		0.43 (0.43, 1.26)	0.65 (0.67, 3.52)
SSBs	---	DR	Ref	0.61	---	---
SSBs	Female	Sales	Alt		0.15 (0.15, 1.43)	0.86 (0.37, 4.17)
SSBs	Female	FFQ	Alt		-0.01 (-0.01, 1.32)	1.01 (0.3, 3.75)
SSBs	Female	HHBS	Alt		-0.59 (-0.59, 0.68)	1.8 (0.18, 1.98)
SSBs	Male	Sales	Alt		0.35 (0.35, 1.63)	0.7 (0.45, 5.1)
SSBs	Male	FFQ	Alt		0.19 (0.19, 1.53)	0.83 (0.37, 4.6)
SSBs	Male	HHBS	Alt		-0.39 (-0.39, 0.89)	1.48 (0.22, 2.43)
Trans fat	---	DR	Ref	0.22	---	---
Trans fat	Male	Sales	Alt		-0.23 (-1.27,0.94)	1.25 (0.28, 2.55)

Trans fat	Female	Sales	Alt		-0.23 (-1.27,0.94)	1.25 (0.28, 2.55)
Trans fat	Male	FFQ	Alt		0.59 (-2.72,4.23)	0.56 (0.07,68.72)
Trans fat	Female	FFQ	Alt		0.86 (-2.63,4.9)	0.42 (0.07,134.0)
Vegetables	---	DR	Ref	0.64	---	---
Vegetables	Female	FAO	Alt		0.12 (0.12, 1.33)	0.89 (0.31, 3.78)
Vegetables	Female	Sales	Alt		0.62 (0.62, 1.83)	0.54 (0.51, 6.21)
Vegetables	Female	FFQ	Alt		-0.05 (-0.05, 1.16)	1.05 (0.26, 3.18)
Vegetables	Female	HHBS	Alt		0.1 (0.1, 1.31)	0.91 (0.3, 3.69)
Vegetables	Male	FAO	Alt		0.16 (0.16, 1.37)	0.85 (0.32, 3.94)
Vegetables	Male	Sales	Alt		0.66 (0.66, 1.87)	0.52 (0.53, 6.49)
Vegetables	Male	FFQ	Alt		-0.01 (-0.01, 1.2)	1.01 (0.27, 3.32)
Vegetables	Male	HHBS	Alt		0.14 (0.14, 1.35)	0.87 (0.32, 3.85)
Whole grains	---	DR	Ref	0.69	---	---
Whole grains	Female	FAO	Alt		1.94 (1.94, 3.37)	0.14 (1.82, 29.05)
Whole grains	Female	FFQ	Alt		-0.35 (-0.35, 1.37)	1.42 (0.13, 3.94)
Whole grains	Male	FAO	Alt		2.09 (2.09, 3.52)	0.12 (2.12, 33.76)
Whole grains	Male	FFQ	Alt		-0.2 (-0.2, 1.52)	1.22 (0.15, 4.58)

**Adjustment factor is the transformed beta coefficient in normal space and can be interpreted as the factor by which the alternative case definition is adjusted to reflect what it would have been if measured as the reference.*

Modelling strategy

Exposure model

We use a spatiotemporal Gaussian process regression (ST-GPR) framework to estimate the mean intake of each dietary factor by age, sex, country, and year. In GBD 2019, we removed lag-distributed income as a covariate from most of our models and added country-level energy availability (Table 2).

To characterise the distribution of each dietary factor at the population level, we use an ensemble approach that separately fit 12 distributions for individual-level microdata to specific to each data source's sampled population. The respective goodness of fit of each family was assessed, and a weighting scheme was determined to optimise overall fit to the unique distribution of each risk factor. A global mean of the weights for each risk factor's data sources was created. We then determined the standard deviation of each population's consumption through a linear regression that captured the relationship between the standard deviation and mean of intake in nationally representative nutrition surveys using 24-hour diet recalls:

$$ln\ (Standard\ deviation) = \beta_0 + \beta_1 \times ln\ (Mean_i)$$

Then we applied the coefficients of this regression to the outputs of our ST-GPR model to calculate the standard deviation of intake by age, sex, year, and country. We also quantified the within-person variation in consumption of each dietary component and adjusted the standard deviations accordingly.

Theoretical minimum-risk exposure level

The dietary TRMELs were updated for GBD 2019. For harmful dietary risks other than sodium, TMREL was set to zero. For protective dietary risk factors, we first calculated the level of intake associated with the lowest risk of mortality from each disease endpoint based on the 85th percentile of intake across all epidemiological studies included in the meta-analysis of the risk-outcome pair. Then we calculated the TMREL as the weighted average of these numbers using the global number of deaths from each outcome as the weight.

Table 4**. Theoretical minimum-risk exposure level for dietary factors, GBD 2017 and GBD 2019**

**Dietary factor**	**GBD 2017**	**GBD 2019**
Fruits	200-300 g/day	310-340 g/day
Vegetables	290-430 g/day	280-320 g/day
Whole grains	100-150 g/day	140-160 g/day
Nuts	16-25 g/day	10-19 g/day
Red meats	18-27 g/day	0 g/day
Processed meats	0-4 g/day	0 g/day
Milk	350-520 g/day	360-500 g/day
Legumes	50-70 g/day	90-100 g/day
Sugar sweetened beverages	0-5 g/day	0 g/day
Polyunsaturated fatty acids	9-13% of total daily energy	7-9% of total daily energy
Seafood omega-3 fatty acids	200-300 mg/day	430-470 mg/day
Trans fatty acids	0-1% of total daily energy	0% of total daily energy
Dietary fibre	19-28 g/day	21-22 g/day
Dietary calcium	1.0-1.3 g/day	1.06 – 1.1 g/day
Dietary sodium	1-5 g/day	1-5 g/day

Relative risks

For GBD 2019, we performed systematic reviews for each dietary risk and its related outcomes. Using the sources identified during these searches, we incorporated the most recent epidemiological evidence assessing the relationship between each GBD dietary risk factor and related outcomes in our relative risk analysis. After evaluating all available evidence, we found sufficient evidence on the casual relationship for 8 new R-O pairs and insufficient evidence for 5 old R-O pairs. Based on these results, we updated the R-O pairs used the GBD dietary risk factor analysis in the following ways:

Removed:

Diet low in fruit and nasopharynx cancer
Diet low in fruit and other pharynx cancer
Diet low in fruit and oesophageal cancer
Diet low in fruit and larynx cancer
Diet low in whole grains and haemorrhagic stroke

Added:

Diet low in whole grains and colon and rectum cancer
Diet high in red meat and breast cancer
Diet high in red meat and ischaemic heart disease
Diet high in red meat and haemorrhagic stroke
Diet high in red meat and ischaemic stroke
Diet low in fibre and ischaemic stroke
Diet low in fibre and haemorrhagic stroke
Diet low in fibre and diabetes mellitus

Additionally, based on the most recent epidemiological evidence and GBD 2019 newly developed methods for characterising the risk curve, we updated the dose-response curve of relative risks for all dietary risks. For sodium, we continued to estimate its effect on cardiovascular disease based on the effect of sodium on systolic blood pressure.

There is a well-documented attenuation of the risk for cardiovascular disease due to metabolic risks factors throughout one's life. To incorporate this age trend in the relative risks, we first identified the median age-at-event across all cohorts and considered that as the reference age group. We then assigned our newly estimated risk curves to this reference age group. Then, we derived the percentage change in relative risks between each age group and the reference age group by averaging percentage changes in relative risks of all metabolic mediators. The three cardiovascular disease outcomes for dietary risks are haemorrhagic stroke (including intracerebral hemmorhage and subarachnoid hemmorhage), ischaemic stroke, and ischaemic heart disease, and the effects of dietary risks on them are mediated through high systolic blood pressure, cholesterol (not included for haemorrhagic stroke), and fasting plasma glucose. Since the effect of diet is estimated independently of body-mass index (BMI) in the GBD, BMI was not included as a mediator in the RR age trend analysis.

Citations

1. Schmidhuber, Josef, et al. The Global Nutrient Database: Availability of Macronutrients and Micronutrients in 195 Countries from 1980 to 2013. *The Lancet Planetary Health*, vol. 2, no. 8, 2018, doi:10.1016/s2542-5196(18)30170-0.

Cause of death estimation for peripheral arterial disease

Estimation process for DALY

Computing DALYs

To estimate DALYs for GBD 2019, we started by estimating cause-specific mortality and non-fatal health loss. For each year for which YLDs have been estimated, we computed DALYs by adding YLLs and YLDs for each age-sex-location. Uncertainty in YLLs was assumed to be independent of uncertainty in YLDs. We calculated 1000 draws for DALYs by summing the first draw of the 1000 draws for YLLs and YLDs and then repeating for each subsequent draw. 95% UIs were computed by using the 25th and 975th ordered draw of the DALY uncertainty distribution. We calculated DALYs as the sum of YLLs and YLDs for each cause, location, age group, sex, and year. For more information, please refer to the following figure A.

Figure A. DALY burden estimation for GBD 2019

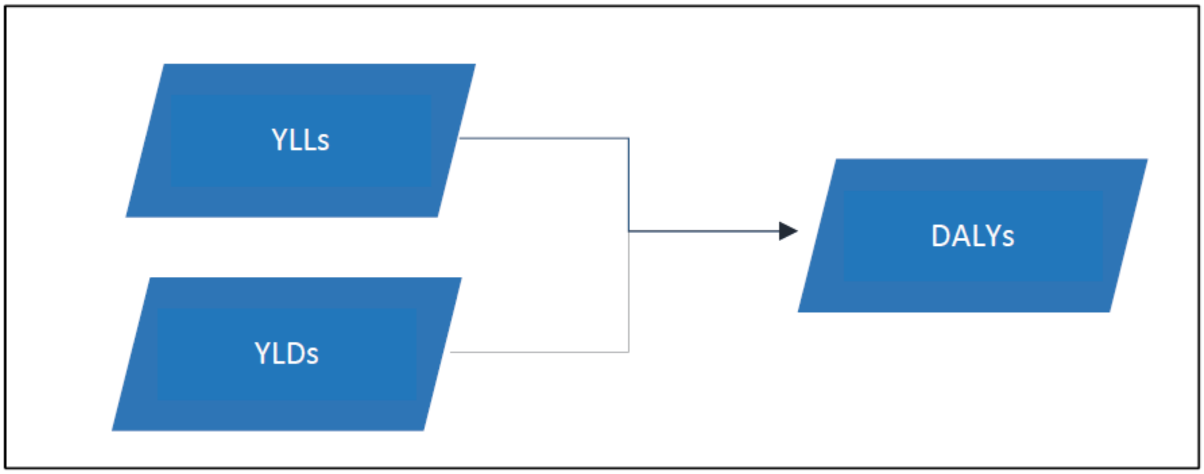

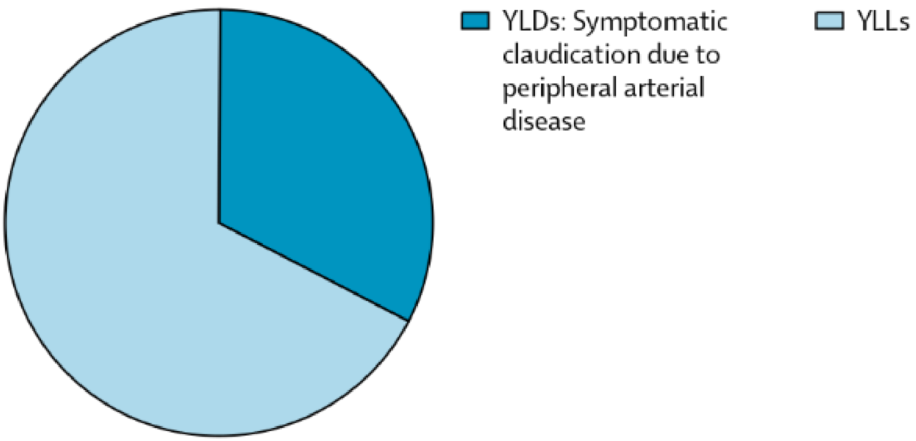

Section 2: GBD 2019 Causes of Death database

Given that various aspects of the GBD model, such as the estimation of Causes of Death (CoD) and non-fatal outcome, are interlinked, the modeling process for PAD is not entirely standalone from other GBD disease models. Consequently, we provide a detailed outline of the general steps and procedures used for handling data for CoD and non-fatal outcome estimates.

Section 2.1: CoD data identification[1]

Section 2.1.1: Overview of data types

The CoD database contains seven types of data sources (table S4): vital registration (VR), verbal autopsy (VA), cancer registry, police records, sibling history, surveillance, survey/census, and minimally invasive tissue sample (MITS) diagnoses. In countries with complete VR systems, there is no need to use any other data source. Less than half the world's population has deaths captured in a VR system, therefore, for countries with incomplete VR systems, vital statistics for causes of death may be supplemented with other data types (appendix figure 3).

Section 2.1.2: ICD-detail

A majority of the CoD data is VR data obtained from the World Health Organization (WHO) Mortality Database, a compilation of data submitted to the WHO by individual countries. VR is also obtained from country-specific mortality databases operated by official offices. Each cause is coded directly to the most detailed CoD when possible, whereas cause codes in data tabulated by International Classification of Disease (ICD-) are coded to aggregated cause groups. The CoD database contains 2,525 country-years of detailed data from 1980 to 2018, which includes underlying CoD coded with 3–5 digit codes, by country, year, sex, and age groups. Detailed causes are coded to one of the following ICD-detail coding systems: ICD-8, ICD-9, or ICD-10 (table S5). Each coding system has a similar cause hierarchy and cause list that has continually developed over time. ICD-10 is the current standard and the most exhaustive cause list. Within the cause lists, 5-digit codes are truncated to 4-digit codes to condense the lists. Updates to ICD-detail occur biannually as WHO releases new versions or as country collaborators provide additional data. Updates to data from WHO increasingly include ICD-10 CoD data as it is the most current classification of CoD, while updates to ICD-8 and ICD-9 detailed lists are less common. In the case of overlapping data, preference is given to data from pre-determined country collaborations, which are updated annually.

Section 2.1.3: ICD-tabulations list

The ICD tabulation lists include the ICD-8 List A (ICD-8A), ICD-9 Basic Tabulation List (BTL), ICD-10 Mortality Tabulation, Russia Tabulation, and India Medical Certification of Cause of Death (MCCD). These data sources make up 1096 country-years from 1980 to 2016 in the CoD database. All are condensed versions of the ICD-8, ICD-9 and ICD-10 detail lists with some differences in the format of cause lists depending on the data source. ICD-8A, ICD-9 BTL, and ICD-10 Mortality Tabulation CoD are assigned to subtotal groups (referred to as chapters) and cause groups respective to ICD-detail groups. Additionally, ICD-9 BTL includes ICD-9 detail codes for some cancers and a custom tabulation scheme for the former Union of Soviet Socialist Republics (USSR) countries. The Russia Tabulation lists and India MCCD cause lists each have custom nomenclatures based on ICD-detail cause codes.

Two of the drawbacks in using tabulation lists are discrepancies in the accuracy of death counts and lack of detail due to aggregated cause groups. There are instances where the sum of deaths in chapter subtotals are not equal to the sum of cause groups within the chapter. To account for any missing or duplicate deaths reported within the cause groupings, death counts are systematically adjusted by calculating the differences between subtotals and sub-causes within the cause groups. Any differences are assigned to a remainder cause group. To account for the lack of cause code detail, select cause groups are disaggregated (Step 1.1) to create a complete cause list. Updates to ICD tabulation lists obtained from WHO occur less frequently compared to ICD-detailed lists as more countries are reporting deaths in ICD-detail. In instances of overlapping data, preference is given first to detailed collaborator data, followed by detailed WHO data, then tabulated collaborator data, and finally tabulated WHO data.

Section 2.1.4: China Disease Surveillance Points /China Center for Disease Control and Prevention

The two primary sources of data for China are surveillance data from the China Disease Surveillance Points (DSP) system and VR data collected by the Chinese Center for Disease Control and Prevention (CDC). In the China DSP data, deaths were reported across 145 disease surveillance points used from 1991 to 2003, 161 disease surveillance points from 2004 to 2012, and 605 disease surveillance points from 2013 to 2017. While China DSP with ICD-10 coding is considered sample VR data, it provides national coverage and cause detail. Thus, it receives similar processing and treatment to the China CDC VR from 2008 to 2016. From 2008 to 2017, all of the deaths and CoD information from the DSP system and other system points throughout China were collected and reported via the Mortality Registration and Reporting System, an online reporting system of the Chinese CDC. The deaths in these data are reported at the strata level, a metric that is specific to China. Counties are stratified by urban and rural classification, but definitions of urbanity vary across counties. In Step 7, we use a method developed to scale up deaths from strata level to the province level.

Section 2.1.5: Sample registration system

Sample registration systems are expanding in several countries, and are key sources of data in Indonesia and India. The Sample Registration System (SRS) is a dual-record system wherein a resident part-time enumerator continuously records births and deaths in each household within the sample unit every month. A full-time SRS supervisor thereafter independently collects the vital events along with other related details for each of the preceding six month periods during the calendar year.

Section 2.1.6: India Medical Certification of Cause of Death

The India MCCD has data for the urban parts of the majority of the states and union territories beginning in 1980. Deaths reported in this data source have been medically certified and are considered VR data. The CoD are reported in a tabulation list with a unique numbering scheme that conforms to ICD-9 and ICD-10 detail codes, which must be disaggregated. MCCD is state-split to fill in data gaps (Step 1.2 State Splitting) prior to age-sex splitting. Because SRS is widely considered a more credible assessment of CoD in India, we chose to use MCCD data only in certain cases for modelling with cause of death ensemble modelling (CODEm). We preserved MCCD data in the database for two primary reasons. First, where the three midpoint years of SRS data resulted in the loss of a clear time trend, as was the case for maternal mortality, we chose to preserve MCCD in addition to SRS. Second, MCCD has an advantage over SRS in cases where VA is not a valid instrument for ascertaining CoD, like encephalitis and dengue fever. In these cases, we kept MCCD over SRS.

Section 2.2: Verbal autopsy[1]

Section 2.2.1: Verbal autopsy coded to ICD-10 and other lists

In countries without VR systems, VA studies are a viable data source to inform CoD. Data are obtained by trained interviewers who use a standardised questionnaire to ask relatives about the signs, symptoms, and demographic characteristics of recently deceased family members. CoD is assigned based on the answers to the questionnaires.

VA data are highly heterogeneous: studies use different instruments, different cause lists (from single causes to full ICD cause lists), different methods for assigning CoD, different recall periods, and different age groups. Cultural differences may also affect the interpretation of specific questions. CoD validity must be considered when mapping to a GBD cause. VAs are likely accurate in assigning CoD to road injury or homicide but less accurate for causes requiring medical certification, such as cardiovascular causes. Studies may also occur as stand-alone assessments or as part of an extended network, such as The International Network for the Demographic Evaluation of Populations and their Health (INDEPTH) Network[6]— a continuous surveillance source with several Demographic Surveillance Systems sites that collect data coded to ICD-detail causes.

Section 2.2.2: InterVA-modelled verbal autopsy

InterVA (Interpreting Verbal Autopsy), a set of computer models intended to facilitate interpreting VAs, was found to be non-credible by the Population Health Metrics Research Consortium (PHMRC).[7] As a result, InterVA-modelled VAs are typically excluded from our analysis because of low validations, except for injuries and maternal causes, used to fill gaps and stabilise patterns.

Section 2.2.3: Other data types

Section: 2.2.3.1 Maternal mortality data

In locations with low-quality, or no VR, maternal mortality metrics can be found in surveillance, surveys, census, and sibling history data sources. The best data have death counts due to maternal causes and the total number of deaths for women within the reproductive ages of 10–54 by year. If a data source is missing these components, creating a complete cause list is necessary by using live births and all-cause mortality deaths.[8] Though death counts are the preferred metric, maternal mortality is often measured by using the maternal mortality ratio (MMR), which is easily converted to deaths by using live births. The China Maternal and Child Surveillance data is adjusted by scaling data from the strata to the province level (Step 7).

Section: 2.2.3.2 Surveys and censuses reporting fraction of deaths due to selected injuries

Surveys and censuses are often used in countries with less developed VR systems; in countries with adequate VR, surveys and censuses are supplementary. Much like VAs, the CoD validity is a concern because of lack of medical certification at the time of death. For these data sources, we keep only causes related to maternal mortality and injuries. The remaining causes are accounted for as a remainder of total deaths in the sample size.

Section 2.2.4: Police records

In most countries, police and crime reports are an important source of information for some types of injury deaths, notably road injuries and interpersonal violence. Our police data come from reports on road traffic and crime trends. The police reports used in this analysis were obtained from published studies, national agencies, and institutional surveys such as the United Nations (UN) Crime Trends survey and the UN Office on Drugs and Crime Global Study on Homicides. We assessed whether police reports were likely to be complete and to cover the entire country by comparing police trends with those seen in VR. Data are excluded in instances where police data for road traffic injuries are significantly lower than the VR. Police data that meet our inclusion criteria and provide complete coverage are uploaded to the database for use in road injuries and interpersonal violence deaths estimation.

Section 2.2.5: Population-based cancer registries

Section 2.2.5.1 Cancer registries with incidence

Data on cancer incidence were sought from individual population-based cancer registries as well as from databases that include multiple registries, including Cancer Incidence in Five Continents, NORDCAN, and EUREG. Cancer registries were identified through the membership list of the International Association of Cancer Registries, through the GBD collaborator network, through publications, or through the GHDx. Registries were excluded if they were not representative of the coverage population, if the data were limited to years prior to 1980, if the source did not provide details on the population covered, or if the list of cancer types included was not comprehensive for the age group covered. Beginning in GBD 2019, childhood cancer-specific population-based cancer registry data were sought and included.

Section 2.2.5.2 Cancer registries with incidence and high-quality mortality data

In addition to incidence, some high-quality cancer registries also report cancer mortality data. These data were also extracted and used as inputs to the mortality-to-incidence model.

Section 2.3: Standardise input data (step 1)[1]

The input data to the CoD database are received in various formats and must be standardised to run through central CoD machinery to then upload to the database. Raw data inputs come from data sources such as mortality databases, literature reviews, or reports. Usable data sources must have a clear sample size of the number of deaths in the population and exhaustive cause lists. The complexity of the data cleaning process varies drastically across data sources. For VR microdata with the location, age, sex, year, and ICD-coded cause of every death, very little effort is necessary to standardise it into a consistent structure. Other sources may require weeks of careful review to accurately extract scans of hardcover CoD reports into spreadsheets that can be transformed and standardised.

At this point, data are assigned source identifiers so that they can be linked to the GHDx and cited appropriately. Any aggregate age and sex categories are flagged for age-sex splitting. The methods of cause-of-death assignment and data collection are reviewed to determine which source type to assign; for example, we distinguish sibling history data from surveys with a VA module. Only data at the most detailed level of the GBD location hierarchy are used. Documentation from the source is reviewed to determine if the population is representative of the location or only a subset of the population in that location. Data sources representing a subset of the population are flagged as non-representative; this flag is used by Cause of Death Ensemble modelling (CODEm) to increase the variance associated with such data points.

Finally, diagnostics are reviewed at this stage to avoid sending cleaning errors downstream. We review cause-specific deaths for each demographic group to ensure the data are reasonable. For example, it is unlikely that male breast cancer deaths are higher than female breast cancer deaths or deaths from neonatal causes occur in age groups over one year. All death totals are compared with the sum of cause-specific deaths to ensure the observed deaths are accounted for and sample size is complete.

Section 2.3.1: Disaggregation (step 1.1)

CoD in tabulated VR data are condensed into aggregated groups, some of which can be mapped directly to GBD causes, while other aggregated cause groups are not informative and cannot be mapped to them. To correct for this, aggregated causes were mapped and split onto multiple ICD-8, ICD-9, and ICD-10 detail causes, or targets, based on the ICD groupings within the aggregated causes. ICD-8, ICD-9, and ICD-10 detail codes serve as targets because they are the highest-quality VR data and enable the calculation of proportions used to split the aggregated cause data into detailed causes. The proportions of deaths from nearby countries within the super-region were used to fill in data gaps as they were likely to have similar CoD trends.

We determined the targets based on detail causes missing from the tabulated cause list. For example, in ICD-9 BTL, the tabulated cause list includes a viral diseases group. In the hierarchy of causes, this group is comprised of “measles”, “yellow fever”, “encephalitis”, “hepatitis”, “rabies”, “other infectious diseases”, “garbage code”, and “remainder of viral diseases”. We did not consider this list to be an

exhaustive list of viral diseases based on the range of ICD-detail codes given in the ICD-9 BTL documentation. To make the cause list exhaustive and inclusive of other viral diseases, we split the remainder of the viral diseases group into “other meningitis”, “other infectious diseases”, “herpes”, “dengue”, “other neglected tropical diseases”, and “garbage code”. After a list of targets was determined, the aggregated deaths were disaggregated to the target causes by using ICD-8, ICD-9, and ICD-10 detail proportions generated at the super-region level for the corresponding sex and age groups across all years in the time series. For example, in ICD-9 detail data, 54.8% of deaths in males in Latin America and the Caribbean within the target group for the BTL “remainder of viral diseases” group were designated to “other meningitis.” Thus, 54.8% of deaths in the tabulated group “remainder of viral diseases” were assigned to “other meningitis” for any country within that particular super-region. For any cause and demographic group for which we lacked ICD-detail, global proportions were used.

Section 2.3.2: State splitting (step 1.2)

Two sources for CoD estimation in India are the MCCD report, which reports medically certified deaths from health facilities in mostly urban areas[9], and the SRS, which collects information via VA about one-half of 1% of the total population in India, including both urban and rural areas, from 8853 sampling units as of 2014.[10] For MCCD, missing data impedes estimation of trends at the state level. We used a first-order, log-linear model of the four-way contingency table of deaths by sex, age, state, and year to estimate the missing state-years. We fit the model to all available data for MCCD separately for each cause, including state-specific all-age measurements and age-specific national measurements. From this, we produced estimates for each combination of sex, age, state, and year. We then used these estimates wherever the raw data did not include sex-specific, age-specific, and state-specific death counts.

For MCCD, the model was fit separately for ICD-10-based and ICD-9-based reports by using the tabulated cause list present in the data.

Section 2.3.3: Calculate non-maternal deaths (step 1.3)

In cases when maternal mortality metrics do not include both deaths due to maternal causes and deaths due to non-maternal causes for women of reproductive age, live births and all-cause mortality estimates can be used to calculate deaths. Many studies report maternal deaths as the MMR. MMR is the number of maternal deaths per 100,000 live births and can be used to calculate deaths when it has been derived from primary data and not estimated. Maternal deaths were calculated by using MMR and live births; if live births were missing we substituted live birth estimates and used the following equation:

$$Maternal\ deaths\ = \frac{MMR}{100{,}000} \times Live\ births$$

If a study was non-representative, we extracted sample size and live births from that study. After maternal deaths were calculated, we used the difference from all-cause mortality estimates to determine non-maternal deaths.

A more accurate and data-inclusive method of calculating maternal and non-maternal deaths incorporates coverage and splits deaths for a range of years into individual years. If there were live births in the study, we adjusted the coverage.

$$Coverage = \frac{Live\ births}{GBD\ estimated\ live\ births}$$

After coverage was calculated, totals deaths were scaled to be more representative. This gives a more accurate death count since the envelope assumes representative coverage. We then calculated non-maternal deaths by using all-cause mortality as an all-cause total.

$$Maternal\ envelope\ with\ coverage\ = Maternal\ envelope \times Coverage$$

An additional adjustment can be applied to maternal data spanning over a range of consecutive years, which allows for more data inclusion. The years within specified year ranges are separated into individual years, and total deaths within the year range were split between each individual year by using the fixed proportions of maternal deaths from VR in that particular country. We used only VR data to inform the proportions because it was both high-quality and representative.

Section 2.4: Map to GBD cause list (step 2)[1]

In GBD 2019, we used 439 maps to translate causes found in the input data to the GBD 2019 cause list. This included 31 maps for VR data, 314 for VA data sources, and 98 for other data types. The largest, and most universal, maps used were those for ICD-9 and ICD-10 VR data. The input data causes varied from 3–4 digit ICD codes to custom cause lists with cause names such as "cholera" or "hepatitis". Our mapping process enabled us to compare these various data sources across demographic groups.

A crucial aspect of enhancing the comparability of data for cause of death is to deal with uninformative, so-called garbage codes. Garbage codes are codes to which deaths were assigned that cannot or should not be considered as the underlying cause of death, for example: heart failure, ill-defined cancer site, senility, ill-defined external causes of injuries, and septicaemia. In GBD 2019, we developed additional maps to translate ICD- codes found in the input data that are non-underlying causes to appropriate target codes based on the levels of the GBD cause list. These garbage codes were mapped to Levels 1–4 of the GBD cause list according to the following criteria:

1. **Level 1** includes all garbage codes for which a Level 1 GBD cause cannot be directly assigned. For example, the underlying causes of "sepsis" or "peritonitis", if not specified in the data, could be an injury, a non-communicable disease, or a type of communicable disease. In these cases, deaths will be redistributed across all three of these Level 1 causes. In addition, deaths coded to impossible or ill-defined causes of death (including "senility" and "unspecified causes") fall into this category, as they will be redistributed onto all causes.

2. **Level 2** includes all garbage codes that can be assigned to Level 1 causes in the GBD cause list. This would include deaths coded to "unspecified injuries" (X59), which are redistributed onto all injuries.

3. **Level 3** includes all garbage codes for which we know the Level 2 CoD and can redistribute onto Level 3 causes. This includes deaths coded to causes such as “unspecified cardiovascular disease”, which falls within the Level 2 cause “cardiovascular diseases”, as well as those coded to “unspecified cancer site”, which falls within the Level 2 cause “neoplasms”.

4. **Level 4** includes all garbage codes for underlying causes of death that can be redistributed within a Level 3 cause. This includes garbage codes such as “unspecified stroke” or “unspecified road injuries.”

Section 2.5: Age-sex splitting (step 3)[1]

Different sources, particularly VA studies, report deaths for a wide range of age groups with varying intervals. For the analysis of CoD, we mapped these different age intervals to the GBD standard set of age groups. The approach to undertake this mapping was the same as in the prior GBD studies (GBD 2017, GBD 2016, GBD 2015, GBD 2013, and GBD 2010).

In the process of assembling a consolidated demographic database, we found that the aggregation of age groups is perhaps the strongest source of inconsistency. By convention, such data are reported in broad age groupings such as 0–4, 5–14, and 15–49, or with both sexes together. The issue of comparability between age-sex groups arose when assembling the GBD CoD database. We developed a tool called age-sex splitting that takes aggregated age groupings and the “both sexes combined” grouping and divides them into what their constituent age groups would likely have been if respective cause-specific and country-specific age distributions had been used. The analytical framework for GBD includes three infant age categories: early neonatal (0–6 days), late neonatal (7–27 days), and post-neonatal (28–364 days), and 20 non-infant age categories: 1–4 years, 5–9 years, and so forth proceeding in five-year age groups until the terminal age group of 95 years and older. We treat unknown ages and sexes in the same manner we treated the “all ages combined” age category and “both sexes combined” sex group. Through this process, we were able to directly compare all data sources on even terms.

The approach to age splitting is based on the following formula. The key assumption underlying this formula is that the relative risk of death by age group compared to a reference age group is invariant across populations. Although this assumption is likely violated in specific cases, a strong biologically based pattern of the relative risk of death for a cause by age is observed for most causes. The basic formula is as follows:

$$D_a = R_a N_a \left(\frac{D_a^{a+x}}{\sum_a^{a+x}(R_a N_a)}\right)$$

Where:

D_a = the number of deaths from a cause in age group *a*

R_a = global cause-specific mortality rate of age group *a*

N_a = the country-year-sex-specific population in age group *a*

D_a^{a+x}= the number of deaths in the age group *a* to *a+x*

With the assumption of invariant relative risks of death by age with respect to a reference age group, this equation can be used, along with population distribution by age, to split an aggregate number of deaths for the age groups *a* to *a+x* into specific deaths for each age group within the aggregate interval.

$$D_{as} = R_{as}N_{as}(\frac{D_{as}^{a+x,s}}{\sum_a^{a+x}(R_{as}N_{as})})$$

Where:

D_{as}= the number of deaths from a cause in age group *a,* sex *s*

R_{as}= global cause-specific mortality rate of age group *a,* sex *s*

N_{as}= the country-year-sex-specific population in age group *a* for sex *s*

$D_{a,s}^{a+x,s}$= the number of deaths in the age group *a* to *a+x* for sex *s*

In some cases, deaths are reported for an aggregate age group for both sexes combined. The task in this case is more complicated, but the same principle can be applied. In this case we assumed that the relative risks of death by age and sex are constant.

This equation can be used to split data aggregated by age and sex. The assumption, however, of invariant relative risks across age and sex is a stronger assumption. Fortunately, data pooled across sexes are less common in the published or unpublished CoD data.

The relative risk of death in a particular age group for a given sex is derived from the global distribution of cause-specific mortality rates found in available VR data. Location-years from the following code systems are used, provided they report the requisite age-detail and sex-detail: ICD-7, ICD-8, ICD-9 BTL, ICD-10 tabulated, ICD-9, and ICD-10. Upon compiling these data, we mapped them to GBD causes and aggregated up to cause Level 3. This is the level at which a particular cause is split—that is, any child cause of a Level 3 parent is split by using the age distribution of that parent (so, chronic kidney disease due to diabetes would be split by using the age pattern of chronic kidney disease).

We next adjusted separately for estimated adult and child VR completeness. Location-year-age-sex-cause specific deaths and population were then aggregated across all location-years, to produce cause-specific mortality rates by age and sex. These were used to determine the risk of death at any age relative to any reference age group, as shown in the above equations.

Section 2.5.1: Correct age-sex violations

Occasionally, data sources include deaths by a cause for which medical consensus exists that death is impossible for the sex and age. For example, some number of deaths may be attributed to cervical cancer in males, or to maternal causes in children younger than 10 years. We have constructed a conservative list of age-sex restrictions. When deaths violate these restrictions, we redistribute them

proportionally onto all causes. All restrictions are included in table S5, Restrictions on age and sex by cause for GBD 2019.

Section 2.6: Correction for miscoding of Alzheimer's and other dementias, Parkinson's disease, and atrial fibrillation and flutter (step 4)[1]

Section 2.6.1: Objective

For certain causes of death, mortality rates reported in VR systems are impossible to reconcile with observed trends in disease prevalence and excess mortality. For dementia, Parkinson's disease, and atrial fibrillation and flutter, these disparities can largely be attributed to death certification practices. We sought to address the known bias in CoD data by first identifying the proportion of all deaths that should be assigned to these causes and next determining the GBD causes and garbage groups to which these deaths are being incorrectly assigned.

In past GBD iterations, we estimated Alzheimer's disease and other dementias, Parkinson's disease, and atrial fibrillation and flutter on the basis of longitudinal prevalence and excess-mortality data to help account for changing patterns in death certification and corresponding implausible time trends in many VR sources. This method was first implemented for Alzheimer's disease and other dementias in GBD 2013. We added atrial fibrillation and flutter to the causes modelled in GBD 2015 and Parkinson's disease to the causes modelled in GBD 2016 by using this strategy. All of these causes were processed in CoDCorrect in a manner that was agnostic to the likely targets of misclassification, which inappropriately led to changes in mortality estimates for causes unrelated to these three in GBD 2015. For GBD 2016, we improved this process by completing a literature review to identify the causes of death most closely associated with Parkinson's and Alzheimer's diseases[11–14] and limiting the CoDCorrect adjustments to include only those causes. For GBD 2017, we refined this approach further by using multiple CoD data to determine the GBD causes and garbage codes from which we move deaths as well as the pattern of misclassification.

Section 2.6.2: Correction process

Changes in coding practices for Alzheimer's diseases and other dementias, Parkinson's disease and Atrial fibrillation and flutter, cause results in spatial-temporal mortality trends that are incompatible with prevalence and case-fatality trends. These changes in coding practices are believed to be the result of shifting consensus in cause of death certification, meaning there is a bias in vital registration (VR) data that needs correction. For Parkinson's disease and atrial fibrillation and flutter, we first estimated excess mortality from prevalence and CoD data in countries with the highest ratio of cause-specific mortality to prevalence, which represents the greatest willingness to code to an under-coded cause. Then, using DisMod-MR 2.1 (see Section 4.5), we derived estimates of cause-specific mortality rates from available prevalence surveys as well as the estimates of excess mortality rate, applied across all countries and over time. We divide this value by the all-cause mortality rate to determine the fraction of overall mortality to attribute to each under-coded cause. For dementia, the modelling process was redesigned in 2019 to no longer depend on vital registration data from the highest dementia mortality locations. Instead, we used relative risk data from cohort studies to calculate total number of excess deaths due to dementia, and end-stage disease proportions from linked hospital to death records to subset these deaths to the proportion of excess deaths with end-stage conditions, which we attributed to dementia.

Finally, we used log-linear interpolation to interpolate final estimates of death due to dementia for the entire time series, and saved as a custom CoD model.

To ascertain the causes from which we would move deaths to under-coded causes, we leveraged multiple CoD data from the USA—by looking to the combinations of intermediate and immediate causes (ie, chain causes) present on death certificates with an under-coded cause listed as underlying, and identifying other causes with similar or identical chain causes, we can determine the expected pattern of miscoded deaths.

The first stage in this process is to parse out years we believe coding practices in the USA to be relatively stable. For dementia, this "gold standard" dataset features 2010–2015, for Parkinson's 2005–2015, and for atrial fibrillation and flutter 2014–2015. We then collect all deaths in those years with the under-coded cause listed as underlying and remove any mention of the under-coded cause from the death certificate. Next, for each unique chain, we search the entire time series of data (1980–2015) to identify the distribution of underlying causes that share that chain. The premise here is that if the diagnosis of dementia, Parkinson's, or atrial fibrillation and flutter were missed, the other causes listed on the death certificate would have been the basis for certification. We then reallocate the under-coded deaths by chain based on that alternative underlying cause distribution.

Upon iterating through all unique chains, we are left with a dataset excluding under-coded causes of death, each remaining cause able to be subdivided into correctly coded deaths and deaths that have been recoded from an under-coded cause by the process described (although not all causes are necessarily targeted by the recoding algorithm). The quantity of interest is the ratio of miscoded deaths to total deaths by cause, age, and sex in our counterfactual dataset.

We apply the ratios derived from the multiple cause data to all VR data to determine the local pattern of miscoding. In this way, the method is sensitive to the observed epidemiology of a given place and time. Then, we calculate the deficit in under-coded cause mortality for each location, year, age, and sex by taking the difference in the expected cause fraction based on prevalence and excess mortality compared to the proportion of deaths actually certified by the VR system. Finally, we scale the cause-specific miscoded deaths to match the deficit and then move them accordingly. We assumed that misclassification of actual dementia and Parkinson's deaths in past years occurred only for reported causes of death that might have plausibly been the direct result of dementia or resulted from misdiagnosis of other organic brain diseases based on clinical expert judgement. A similar assumption is used for atrial fibrillation and flutter, for which only cardiovascular causes and ill-defined garbage codes are considered.

Because the deaths being reallocated vary by location-year, we need a mechanism to ensure plausible limits to how many deaths are extracted from each GBD cause and garbage code. To achieve this, we first run the above-mentioned algorithm on all 5-star VR data (see Section 2.16 of this appendix for an explanation of the star data quality rating system). Then, we determine the 95th percentile of the proportion of deaths moved for each GBD cause and garbage code group by age and sex across location-years among these data. Those values are subsequently stored and applied as the limits for deaths moved by this process.

Section 2.7: Redistribute (Step 5)[1]

A crucial aspect of enhancing the comparability of data for CoD is to deal with uninformative, so-called garbage codes. Garbage codes to which deaths were assigned should not be considered as the underlying cause of death—for example: heart failure, ill-defined cancer site, senility, ill-defined external causes of injuries, and septicaemia. The methods for redistributing these garbage-coded deaths were outlined in detail in Naghavi et al,[15] and the underlying algorithm for redistributing deaths assigned to these codes has not changed since GBD 2013.

Section 2.7.1: Redistribute HIV-related garbage codes (step 5.1)

Because of the disparate nature of HIV/AIDS mortality across space and time, dynamic redistribution of HIV/AIDS-related garbage codes was needed (table S6). To inform this redistribution, we generated target proportions for each garbage group by age band (under 1 month, 1–59 months, 5–19 years, 20–49 years, 50–59 years, 60–69 years, 70–79 years, and 80 years and older), five-year time interval, and sex. The garbage groups either target HIV or a remainder target. The allotment of deaths to either of these is based on the regional increase in the mortality rate of all codes in the group relative to the rates seen from 1980 to 1984—an increase greater than 5% is assumed to be HIV/AIDS-related, and the proportion of those deaths exceeding 5% are redistributed to HIV/AIDS. Any increase less than or equal to 5% is then assigned to the remainder target.

Section 2.7.2: Regress garbage codes versus non-garbage codes (step 5.2)

For each redistribution package, we defined the "universe" of data as all deaths coded to either the package's garbage codes or the package's redistribution targets for each country, year, age, and sex. We then ran a regression based on the following equation separately for each target group and sex:

$$TG_{crt} = \alpha + \beta_1 Gar_{crt} + \beta_2 Age_{crt} Gar_{crt} + \theta_r Gar_{crt} + \gamma_r + \varepsilon_{ct}$$

Where:

TG_{crt} = percentage of deaths within the given garbage code's universe that were coded to a given target group, by country

Gar_{crt} = percentage of deaths within the given garbage code's universe that were coded to a given set of garbage codes

Age_{crt} = age interaction term for the fixed effect on the interaction of garbage and age

α = constant

β_1 = slope coefficient describing the association between Gar_{crt} and TG_{crt}

β_2 = slope coefficient describing the association between the interaction $Age_{crt} Gar_{crt}$ and G_{crt}

γ_r = region-specific random intercept (or super-region if the random effect on region is not significant)

θ_r = region-specific random slope (or super-region if the random effect on region is not significant)

ε_{ct} = standard error, normally distributed and calculated by bootstrapping

This regression was adjusted from GBD 2013 to include fixed effects on the interaction of garbage and age to ensure smooth age patterns. We made this decision after investigating diagnostic visualisations that showed unlikely gaps between proportions assigned to different age groups.

Once proportions were produced for each country, sex, age, and target group, certain adjustments were made to conform our packages to the best medical evidence available. In some cases, we implemented restrictions on the proportions that the regressions could yield. For example, we did not allow any redistribution onto "Chagas disease" outside of Latin America and the Caribbean or "suicide" under the age of 15 years. In other cases, we capped the proportion for some targets to the level that would be produced from proportional redistribution; for example, "haemoglobinopathy" and "haemolytic anaemia" were restricted to the level of proportional redistribution in the redistribution of "left heart failure". Occasionally, further adjustments were made on a case-by-case basis per country, age, sex, and target group to suppress the impact of outliers based on existing epidemiological evidence and expert judgment.

In GBD 2019, we updated the regressions for stroke and diabetes. We dropped the proportion of garbage from the regression formula and ran regression on high-quality, low proportion garbage data (4/5 stars, < 50% GC). We also included all covariates included in the CODEm models for both stroke and diabetes.

Section 2.7.3: Development of an algorithm for redistribution of garbage codes based on multiple CoD data

Multiple CoD data are a form of individual record causes of death data that include an underlying CoD along with other causes in the death chain, including intermediate and immediate causes. By analysing this type of data, we can sometimes find the true underlying CoD in other CoD data where the underlying cause is a garbage code or a mis-assigned CoD.

For GBD 2019, this method was expanded and used in redistribution of the following intermediate causes: sepsis, embolism (pulmonary and arterial), heart failure (left, right, and unspecified), acute kidney injury, hepatic failure, acute respiratory failure, pneumonitis, and unspecified central nervous system disorders. Using multiple CoD records for the USA, Mexico, Brazil, Taiwan (province of China), Italy, and Colombia we identified the fraction of deaths where the underlying cause of death and the intermediate cause was in the causal chain. Using a mixed effect linear regression, we estimated the fraction of intermediate-cause related deaths by underlying GBD cause. These fractions were multiplied by the GBD 2017 CoDCorrect result to calculate the number of deaths intermediate cause-related deaths for each GBD cause. Lastly, we calculated the "intermediate cause fraction", with total intermediate-cause related deaths as the denominator, by age, sex, location, year GBD cause. These fractions were used to redistribute the intermediate-cause-related deaths to a GBD cause. An example

is given below for sepsis where a, s, l, y, c denotes a given age group, sex, location, year, and underlying cause of death:

1. $sepsis\ fraction = \beta_{HAQ\ Index} + \beta_{age\ group} + \beta_{sex} + Y_{cause} + \varepsilon$

2. $sepsis\ deaths_{a,s,l,y,c} = sepsis\ fraction_{a,s,l,y,c} * GBD\ deaths_{a,s,l,y,c}$

3. $total\ sepsis\ deaths_{a,s,l,y} = \sum_c sepsis\ deaths_{a,s,l,y,c}$

4. $fraction\ of\ sepsis\ to\ redistribute_{a,s,l,y} = \frac{sepsis\ deaths_{a,s,l,y,c}}{total\ sepsis\ deaths_{a,s,l,y}}$

To redistribute X59 and Y34 (unspecified injuries) deaths, we used a multi-step approach that utilised the pattern of nature of injury codes in the causal chain in the multiple CoD data. First, we looked at deaths where X59, Y34, and GBD injuries causes were the underlying cause of death and got the pattern of nature of injury codes in the chain. We then derived a cause-specific redistribution proportion based on the probability of a given pattern being coded to X59/Y34 or a GBD injuries cause and summing up these proportions for all patterns. An example below is given for X59:

5. $P_{(pattern_j | UCoD\ X59)} = \frac{\#\ of\ pattern_j\ deaths\ |\ UCoD\ X59}{\sum_{j=0}^{m}(\#\ of\ pattern_j\ deaths\ |UCoD\ X59)}$

6. $P_{(GBD\ injuries\ cause_i | pattern_j)} = \frac{\#\ of\ UCoD\ GBD\ injuries\ cause_i\ deaths\ |\ pattern_j}{\sum_{i=0}^{n}(\#\ of\ UCoD\ GBD\ injuries\ cause_i\ deaths\ |pattern_j)}$

7. $redistribution\ proportion_{GBD\ injuries\ cause_i} = \sum_{j=0}^{m}\left(P\left(pattern_j \middle| UCoDX59\right) * P(GBD\ injuries\ cause_i | pattern_j)\right)$

Where:

$pattern_j$ = a given nature of injury code pattern in the chain of the multiple CoD data

UCoD X59 = a death with X59 coded as the underlying cause of death (UCoD)

UCoD GBD injuries $cause_i$ = a death with a GBD injuries causes coded as the UCoD

We applied these cause-specific redistribution proportions on the data where X59/Y34 were the underlying cause of death to get the number of X59/Y34 deaths "attributable" to each GBD injuries cause. Then, for each GBD injuries cause in the multiple CoD data, we calculated the fraction of redistributed X59/Y34 deaths over the fraction of total injuries death for that cause and modelled this intermediate cause fraction using a mixed effects linear regression similar to the one mentioned above. Like mentioned above, these fractions were then multiplied by GBD 2017 CoDCorrect results, and the cause fractions for X59 and Y34 were calculated by age, sex, location, year, and GBD injuries cause, and then used to redistribute X59 and Y34 deaths to GBD injuries causes.

Additionally, multiple CoD data were used in the correction of the mis-assignment of deaths due to drug

overdoses to unintentional other poisoning. More than 90% of these types of poisonings are due to exposure to narcotics, psychodysleptics, and other drugs, specified or unspecified. More than 97% of these poisonings by substance or drug occurred in ages 15–65 years. These are clearly not cases of accidental ingestion of substances but rather deliberate ingestion and unintentional poisoning. Using multiple CoD records for the USA, Mexico, Brazil, Taiwan (province of China), Italy, Colombia, Australia, and various European countries from 1980 to 2017, we selected all deaths with underlying causes coded to X40–X44 (table A below). Table B shows the combination of other potential causes that can be found in the multiple CoD data for these underlying causes, and table A shows the ICD-10 codes corresponding to these causes. On the basis of Table B, we proportionally redistributed mis-assigned unintentional poisoning deaths to one of these causes. The main assumption behind this algorithm is the predominance of the fatality of some substances when a combination of drugs is considered. Given the combination of different drugs and substances in these codes, opium is the main cause of fatality.[16,17] Other substances, like cocaine, methamphetamine, and alcohol in combination with cannabis are less likely to be dominant in fatality.[18]

For example, if the multiple CoD data show that 40% of deaths include opioid use disorders as an intermediate cause where the underlying cause is X40–X44, the redistribution proportion for opioid use disorders will be exactly 40% due to the dominance of the fatality of opioid use disorders compared to other drugs in the above table. Additionally, in our final results, cannabis and psychoactive and psychedelic drug use disorder deaths were mapped to other drug use disorders.

Table A. ICD-10 codes for substances or drugs used to assign deaths coded to an underlying cause of unintentional poisoning by using multiple CoD data

Accidental poisoning codes	All X40, X41, X42, X43, X44 codes
Opioid Codes	T40.0, T40.1, T40.2, T40.3, T40.4, T40.6, F11.0, F11.1, F11.2, F11.3, F11.4, F11.5, F11.6, F11.7, F11.8, F11.9
Amphetamine Codes	T43.6, F15.0, F15.1, F15.2, F15.3, F15.4, F15.5, F15.6, F15.7, F15.8, F15.9
Cocaine Codes	T40.5, F14.0, F14.1, F14.2, F14.3, F14.4, F14.5, F14.6, F14.7, F14.8, F14.9
Psychoactive and psychedelic drug	T40.8, T40.9, T43.6, F16.0, F16.1, F16.2, F16.3, F16.4, F16.5, F16.6, F16.7, F16.8, F16.9
Alcohol Codes	T51.0, F10.0, F10.1, F10.2, F10.3, F10.4, F10.5, F10.6, F10.7, F10.8, F10.9
Cannabis Codes	T40.7, F12.0, F12.1, F12.2, F12.3, F12.4, F12.5, F12.6, F12.7, F12.8, F12.9

Table B. Multiple cause of death selection algorithm used for redistributing unintentional poisoning causes of death to substance or drug use cause of death

Selection Algorithm						
	Opioids	Cannabis	Cocaine	Amphetamines	Alcohol	Psychoactive and psychedelic drugs
Opioids	Opioids	Opioids	Opioids	Opioids	Opioids	Opioids

Cannabis	Opioids	Cannabis	Cocaine	Amphetamines	Alcohol	Psychoactive and psychedelic drugs
Cocaine	Opioids	Cocaine	Cocaine	Amphetamines + cocaine	Cocaine + alcohol	Cocaine
Amphetamines	Opioids	Amphetamines	Amphetamines + cocaine	Amphetamines	Amphetamines + alcohol	Amphetamines
Alcohol	Opioids	Alcohol	Cocaine + alcohol	Amphetamines + alcohol	Alcohol	Psychoactive and psychedelic drugs
Psychoactive and psychedelic drugs	Opioids	Psychoactive and psychedelic drugs	Cocaine	Amphetamines	Psychoactive and psychedelic drugs	Psychoactive and psychedelic drugs

Multiple CoD data were only available to us for the USA, Mexico, Brazil, Taiwan (province of China), Italy, Colombia, Australia, and various European countries. Because of this limited sample, we applied the result from the multiple CoD analysis from each country to its respective super-region and used global proportions for sub-Saharan Africa. We hope for increased availability of multiple CoD data in future analyses to achieve a more precise distribution for more locations.

Section 2.7.4: Verbal autopsy anaemia adjustment (step 5.3)

To compensate for the over-representative cause fractions from anaemia found in VA studies, we redistributed these deaths based on the causal attribution of severe anaemia from GBD 2015. The proportions were country-year-age-sex specific.

Section 2.7.5: Calculate redistribution uncertainty (step 5.4)

We categorised garbage codes into four levels in order of increasing specificity (see Section 2.4). Some garbage codes are redistributed on all causes (eg, unspecified causes of death) and others are only redistributed onto specific causes (eg, unspecified cancer). Major garbage refers to garbage codes in Levels 1 or 2. Because of the variation in redistribution, estimating uncertainty from garbage redistribution for CODEm modelling was an important goal for GBD 2019.

We assigned redistribution variance to each data point in the CoD database by calculating residual variance from a regression predicting the percentage of garbage coded deaths redistributed to a cause, given the proportion of garbage codes we observed for that location, year, age, sex, cause, and the age standardised relative rate of major garbage codes across all causes. If there is a cause that has greater residual variance, we assume greater redistribution uncertainty.

The two model inputs are the observed percentage of Levels 1, 2, and 3 garbage codes (by cause, age, sex, location, and year) in redistributed CoD data and the percentage of garbage codes in the raw data (calculated as the age standardised mortality rate ratio of major garbage coded deaths to all deaths in the raw data by location, year, and sex). Level 4 garbage codes were excluded from the model to avoid over estimating uncertainty in countries with high percentages of major garbage codes. Additionally, the classification of Level 4 garbage codes is not stable between successive GBD rounds—for example, "unspecified diabetes" was not a garbage code in GBD 2016, and in GBD 2017 was re-classified as a

Level 4 garbage code to permit estimation of diabetes by type. These deaths are still taken into account later in the uncertainty estimation process. The model predicts the percentage of garbage coded deaths redistributed to a cause, given the proportion of garbage codes we observed for that location, year, age, sex, cause, and the age standardised relative rate of major garbage codes across all causes. From this model, we calculate residual variance. It is important to note that the variance here is a measurement of uncertainty of redistribution, not of the level of miscoding in the raw CoD data for a given demographic.

To calculate variance, a dataset was generated that contained percent garbage by location, year, age, sex, and cause, where percent garbage is determined by the equation

$$pct_{garbage} = \frac{deaths_{redistributed} - deaths_{raw}}{deaths_{redistributed}}$$

A mixed-effect linear regression model was then fit to predict the logit percent of deaths from redistribution by age-standardised relative rate of major garbage codes.

$$logit\left(pct_{garbage_{ij}}\right) = \beta_0 + \beta_1 * \log\left(ASR_{majorgarbage_{ij}}\right) + \beta_2 * 15yearage_{ij} + \gamma_{1j} * \log\left(ASR_{majorgarbage_{ij}}\right) + u_j + e_{ij},\ \ \theta_{\{i\}} \sim N(0, \sigma^2)$$

Where:

i indexes dataset-location-year-age-sex-cause data points nested within j groups by GBD region

$ASR_{majorgarbage_{ij}}$ is age-standardised relative rate of major garbage

Residual variance, as estimated by the mean absolute deviation, was calculated for each cause, sex, and age.

The next step was to use the residual variance to calculate uncertainty around each data point in the CoD database. First, we calculated the percent garbage of each data point by treating all deaths that could not be directly mapped to a GBD cause as garbage, including Level 4 garbage codes. Percent garbage was calculated as

$$pct_{garbage} = \frac{deaths_{redistributed} - deaths_{corrected}}{deaths_{corrected}}$$

Where:

$deaths_{corrected}$: deaths post misdiagnosis correction (Section 2.6)

$deaths_{redistributed}$: deaths post redistribution (Section 2.7)

Residual variance was matched to each data point and 100 draws were sampled from a normal distribution by using the cause, age, sex, specific residual variance, and mean of 0. The logit transformed

percent garbage was added to each value in the distribution. Each draw was then transformed out of logit space, and the post-redistribution deaths were calculated as

$$deaths = \frac{deaths_{corrected}}{1 - pct\_garbage}$$

Draws of deaths were processed through noise reduction before calculating the final redistribution variance passed to CODEm, which was added to the total data variance. The mean of the draws was not used as the final estimate because it was found that the logit transformation biased the distribution of cause fractions higher. Instead, only point estimates were used.

Section 2.8: HIV/AIDS misclassification correction (step 6)[1]

In many location-years, certain causes of death known to be comorbid with HIV/AIDS (eg, tuberculosis, other infectious diseases) are seen to have age patterns that diverge from those observed in location-years without widespread HIV epidemics and are in fact more reflective of HIV mortality trends. To identify these instances, a global relative age pattern is generated by using all VR deaths in countries with observed HIV prevalence less than 1% by using the following equation

$$RR_{asc} = \frac{R_{asc}}{\bar{x}(R_{65sc}, R_{70sc}, R_{75sc})}$$

Where:

RR_{asc} is the relative death rate for age group *a*, sex *s*, cause *c*;

R_{asc} is the rate for that age group

$\bar{x}(R_{65sc}, R_{70sc}, R_{75sc})$ is the mean of the rates in ages 65–69, 60–74, and 75–79 for that sex and cause.

This is preferable to comparing mortality rates because we are able to isolate divergence in age pattern while accounting for varying levels of overall mortality by fixing death rates to age groups that are unlikely to be confounded by the presence of HIV. Expected deaths for an identified cause were then determined by the equation

$$ED_{lyasc} = \bar{x}\left(R_{ly65sc}, R_{ly70sc}, R_{ly75sc}\right) \times p_{lasc} \times RR_{asc}$$

Where:

ED_{lasc} are deaths for location *l*, year *y*, age group *a*, sex *s*, and cause *c*;

$\bar{x}(R_{l65sc}, R_{l70sc}, R_{l75sc})$ is the mean of the rates for ages 65–69, 60–74, and 75–79 for that location-year-sex-cause;

p_{lasc} is the population for that location-year-age-sex-cause

RR_{asc} is the global standard relative rate determined in the previous step for that age-sex-cause.

The expected deaths remain attributed to that particular cause, while the difference between observed and expected are reallocated to HIV/AIDS.

Section 2.9: Scale strata to province (step 7)[1]

Over time, a higher proportion of deaths have been registered in China through the expansion of the DSP system and provincial/county efforts to increase CoD registration. With the expansion of coverage, it is possible that province aggregates do not accurately represent the population distribution between urban and rural areas in each year. For this reason, we stratified the data preparation by urban and rural status for each county within each province. Stratification was based on the median level of urbanisation across counties within each province as recorded in the 2010 China census. In the provinces of Tibet and Hainan, all counties were placed into one strata based on largely homogeneous urbanisation levels within each province. This yielded a total of 62 analytical province-strata. Macao and Hong Kong were not included in this stratification system as the VR systems there are independent from that on the mainland; no weighting scheme needs to be carried out in these complete VR systems with quality CoD data.

Within each province-strata, a larger proportion of deaths in-hospital might be reported than that of deaths outside of hospital because of the internet hospital reporting system. To avoid bias, we reweighted in-hospital and out-of-hospital deaths based on the age-sex-province-specific fraction of deaths in and out of hospital in the DSP system. DSP data have been used to establish these percentages because in these communities, there is a concerted effort to identify all out-of-hospital deaths. Province-strata death rates are combined to produce overall province death rates by weighting each strata by population in each age-sex-year group. Province death rates are rescaled so that all-cause mortality equals the estimated death rate in each age-sex-year estimated in the life-table analysis. The Bayesian noise reduction algorithm was used to deal with zero counts and small number issues for rare causes.[18]

Section 2.10: Restrictions post-redistribution (step 8)[1]

Some causes of death can only be reliably assigned through an autopsy by a trained physician. For example, a VA would be unlikely to reliably distinguish between ischaemic and haemorrhagic stroke.

This step ensures that the detail of the cause list at this point in the data prep process is reasonable given the detail of the original data source and the methods by which the CoD was assigned. A “bridge map” is applied over a certain set of sources to ensure that these sources do not contain causes that could not reliably be determined by the methods used. These causes, identified to be too detailed, are then aggregated to their parent cause. This correction is applied to ICD-9 detail, ICD-9 BTL, ICD-10 tabulated, ICD-8 detail, ICD-8 A, China DSP (tabulated ICD-9), India MCCD, India SRS, USSR tabulated ICD-9, the Philippine Vital Statistics Reports, Iran ICD-10 VR from the Ministry of Health and Medical Education, and all VA. An example of this would be the aggregation of all sub-types of lower respiratory infection to lower

respiratory infection in ICD-9 BTL.

Section 2.11: Drop VR country years or mark as non-representative (step 9)[1]

Lozano and colleagues[20] describe the negative impact that low-completeness VR data could have on CoD modelling for GBD 2010. In particular, in settings where a data source does not capture all deaths in a population, the cause composition of deaths captured might be different from those that are not. However, a completeness sensitivity test found that low-completeness VR data had little impact on the cause-specific mortality trends at the global level.

For GBD 2019, we investigated the impact of these data at the country and subnational and determined that these data produced unlikely trends in the models affected. Despite the minimal impact on global trends, better models were produced by eliminating or marking as non-representative data with extremely low completeness. VR completeness was estimated as the number of deaths registered divided by the number of deaths estimated in the GBD mortality envelope.

For this round, VR location-years with completeness less than 50% were dropped, while location-years with completeness between 50% and 69% were marked as non-representative.
In addition, any country-year with a number of deaths registered to major garbage codes greater than 50% of the deaths registered was dropped. Major garbage coding refers to garbage codes redistributed across Levels 1 and 2 of the cause hierarchy. When we redistribute garbage codes across Levels 1 and 2 of the cause hierarchy, this is because we do not have enough information to distribute them to more detailed Levels [3 and 4].

Section 2.12: Cause aggregation (step 10)[1]

The cause list is organised in a top-down hierarchical format containing four levels. The first group, or Level 1, sums all causes. Following all-cause mortality are Level 2 causes, which include three broad groupings of causes of deaths: “communicable, maternal, neonatal, and nutritional diseases”; “non-communicable diseases”; and “injuries”. Within those Level 2 groupings are finer levels used for modelling. Level 3, or parent causes, are aggregated; the mortality estimate for a parent cause in the hierarchy represents the sum of the causes under that rubric. Sub-causes within Level 3 causes—Level 4—are more detailed. For example, the parent cause “intestinal infectious diseases” contains the three sub-causes: “typhoid fever”, “paratyphoid fever”, and “other intestinal infectious diseases”. Included in the parent cause estimate are deaths mapped directly to the parent and any Level 4 sub-causes. In data where there was not enough information to assign a Level 4 cause, we aggregated to the Level 3 parent cause. Exceptions to aggregating the Level 4 sub-causes to the parent are instances when certain sub-causes are not present. The United Nations Crime Trends police data only identify homicides, and aggregating homicides to injuries would not accurately represent all injuries.

Section 2.13: Remove shocks and HIV/AIDS maternal adjustments (step 11)[1]

For GBD 2019, CODEm models use an HIV/AIDS- and shock-free envelope. To be comparable, cause fractions must also be HIV/AIDS- and shock-free. Cause fractions were uploaded to the CoD database as the number of deaths due to the cause over an adjusted sample in which the number of deaths due to “HIV/AIDS”, “conflict and terrorism”, “police conflict and executions”, and “exposure to forces of nature”

were removed.

Section 2.13.1: Remove HIV/AIDS and shocks from denominator where cause list includes HIV/AIDS (step 11.1)

The first step to generate HIV- and shock-free cause fractions was to remove any deaths from the sample that were directly coded to “HIV/AIDS”, “collective violence and legal intervention”, or “exposure to forces of nature”. The cause fraction uploaded to the database can be calculated by a simple equation.

$$CF_{l,t,a,x,c} = \frac{D_{l,t,a,x,c}}{D_{l,t,a,x} - D_{l,t,a,x,hiv} - D_{l,t,a,x,war} - D_{l,t,a,x,disaster}}$$

Where:

$CF_{l,t,a,x,c}$ is the cause fraction for a location *l*, year *t*, age *a*, sex *x*, and cause *c*

$D_{l,t,a,x,c}$ is the number of deaths observed for cause *c* in location *l*, year *t*, age *a*, and sex *x*

$D_{l,t,a,x}$ is the total number of deaths due to all causes observed in location *l*, year *t*, age *a*, and sex *x*

$D_{l,t,a,x,hiv}$, $D_{l,t,a,x,war}$, and $D_{l,t,a,x,disaster}$ are the numbers of deaths observed in location *l*, year *t*, age *a*, and sex *x* for causes “HIV/AIDS”, “collective violence and legal intervention”, and “exposure to forces of nature”, respectively

Cause fractions for HIV/AIDS and shock causes were also uploaded to the database for use in separate estimation processes described by Wang et al.[21] In this case, cause fractions followed the standard equation, with variables following the same explanation.

$$CF_{l,t,a,x,c} = \frac{D_{l,t,a,x,c}}{D_{l,t,a,x}}$$

Section 2.13.2: Remove HIV/AIDS deaths from maternal mortality sources (step 11.2)

HIV-free cause fractions were also uploaded for sources on mortality due to maternal causes. In these cases, the sample of all deaths observed in the study is likely to contain some amount of deaths due to HIV/AIDS and shocks, but the sample only includes cause information on maternal deaths. To account for the presence of HIV/AIDS and shocks in the entire sample, we assumed the same proportion of total deaths due to HIV/AIDS by location, age, sex, and year as provided from the estimation of HIV/AIDS and all-cause mortality described by Wang et al.[21]

Maternal mortality studies were only corrected for HIV/AIDS if the sample of total deaths was provided in the data source. Where sources provided only the MMR, we applied the rate to the HIV- and shock-free envelope produced by the analysis described in Wang et al.[21] and thus did not need to adjust cause fractions at this point in the process.

Where a correction was applied, we used the following equation:

$$CF_{l,t,a,x,mat} = \frac{D_{l,t,a,x,maternal}}{D_{l,t,a,x,maternal} + \frac{E[D_{l,t,a,x,hiv\_shock\_free}]}{E[D_{l,t,a,x}]} D_{l,t,a,x,non-maternal}}$$

Where:

$CF_{l,t,a,x,mat}$ is the resulting cause fraction due to maternal causes for the location (l), year (t), age (a), sex (x);

$D_{l,t,a,x,mat}$ is the number of observed deaths in the sample due to maternal causes

$D_{l,t,a,x,non-maternal}$ is the number of observed deaths in the sample due to non-maternal causes

$E[D_{l,t,a,x}]$ is the GBD estimate of all-cause mortality in the location, year, age, and sex

$E[D_{l,t,a,x,hiv\_shock\_free}]$ is the GBD estimate of HIV- and shock-free mortality in the location, year, age, and sex

Section 2.13.3: HIV/AIDS correction of sibling history, census, and survey data (step 11.3)

As described in our analysis from GBD 2013, many studies have failed to find increased mortality in HIV+ pregnant mothers, but those who have advanced HIV are known to have increased baseline mortality. Prior to GBD 2013, we did not distinguish between deaths in HIV+ women that were caused by pregnancy and those for whom the pregnancy was incidental to their death. To more explicitly quantify the contribution of pregnancy to death in HIV+ women, and therefore more accurately estimate the maternal death count, we completed two additional analyses for GBD 2013 and all subsequent GBD analyses. First, we determined the population attributable fraction (PAF) of HIV/AIDS to pregnancy-related death. Second, we determined the proportion of pregnancy-related deaths in HIV+ pregnant mothers that are aggravated by pregnancy and are therefore by definition maternal deaths.

$$PAF = \frac{P(RR-1)}{1+P(RR-1)}$$

Where:

PAF is the population attributable fraction

P denotes the prevalence of HIV in pregnancy

RR is relative risk of mortality in HIV+ vs HIV- pregnant mothers.

To recap our analysis for GBD 2013, we used the paper published by Calvert and Ronsmans[22] to identify sources that could inform Step 1 of our HIV-correction analysis. We independently reviewed each of

the component studies in Calvert and Ronsmans' review and extracted data directly, not from the systematic review paper. We identified only one additional study that was not used in Calvert and Ronsmans' analysis. We have, however, not used all the studies included in that review. Specific details are as follows:

1) Figueroa-Damian et al.[23] was excluded for not including any postpartum deaths at all.

2) In the case of Ryder et al.[24] and Zvandasara et al.[25] we excluded those deaths that occurred more than 12 months after delivery.

3) We excluded the results from Chilongozi et al.[26] from the site that did not include any HIV- patients.

4) Leroy et al.[27] was not in the bibliography. We could not locate it for review so it was excluded.

5) Kourtis et al.[28] was extracted with adjustment of the denominator based on the average number of hospitalisations per delivery in each group.

6) Ticconi et al.[29] was excluded for being both non-representative and including subgroup data from mothers with malaria infection.

A total of 21 sources were included in our analysis of the increased mortality risk of HIV+ versus HIV- women in pregnancy.[30] We performed DerSimonian-Laird random effects meta-analysis to derive a pooled estimate of RR of death during pregnancy given HIV positivity.[31] The pooled effect size was 6·40 (95% uncertainty interval [UI] 3·98–10·29), which was then used to calculate an HIV PAF for each country, age group, and year. To determine the proportion of those HIV-related deaths that were attributable to maternal causes, we performed a second systematic literature review. This time we sought evidence for the excess mortality risk of pregnancy in those women who are already HIV+. Most studies have failed to find such an effect, but most also did not stratify their study population by stage of HIV or ART (antiretroviral therapy) status. Only two studies did this stratification, with a pooled effect size of 1·13 (95% UI 0·73–1·77).[32,33]

An updated literature review to inform the relative risk of mortality in pregnancy in HIV+ versus HIV- women had 14 non-usable sources. We completed this search on May 10, 2019, using the following search strings:

((HIV[Title/Abstract] OR "Acquired Immunodeficiency Syndrome"[Title/Abstract] OR AIDS[Title/Abstract]) AND (“pregnant”[Title/Abstract] OR “pregnancy”[Title/Abstract] OR “postpartum”[Title/Abstract] OR "post partum"[Title/Abstract]) AND (“mortality”[Title/Abstract] OR “death”[Title/Abstract]) NOT "case report" NOT (animals[MeSH] NOT humans[MeSH])

AND (2016/08/15[PDat] : 3000/12/31[PDat]))

Prevalence of HIV in pregnant women was calculated by using the Joint United Nations Programme on HIV and AIDS (UNAIDS) Spectrum model,[34] a compartmental HIV progression model used to generate age-specific incidence, prevalence, and death rates from pre-calculated incidence curves and assumptions about intervention scale-up and local variation in epidemiology. For each location, we used UNAIDS' age-specific ratios of fertility in women living with HIV to fertility in women not living with HIV. In most locations, this ratio is assumed to be greater than one in women aged 15–24 years and less than one and decreasing as age increases beyond 24 years. Since Spectrum assumes fertile ages of 15–49 years, we used the ratio of HIV prevalence in pregnant women to HIV prevalence in the general population at either end of that range to extend estimates to age bands 10–14 years and 50–54 years.

Unlike GBD 2013, when we applied the PAF correction to the envelope of maternal deaths predicted by CODEm, we instead applied country-year-age-group-specific PAF to maternal mortality input data prior to modelling in CODEm. This ensured that both the numerator and denominator of all CF data were internally consistent in their exclusion of background HIV/AIDS mortality. The cause fractions for maternal deaths in sibling history, survey, and census data were therefore adjusted as follows:

$$CF_{l,t,a,x,mat_{adj}} = CF_{l,t,a,x,mat} \times \left(1 - ProP_{hiv_{l,t,a,x}}\right)$$

$$ProP_{hiv_{l,t,a,x}} = PAF_{l,t,a,x,hivpos} \times (1 - rr_{mat})$$

$$CF_{l,t,a,x,mat_{hiv}} = CF_{l,t,a,x,mat} \times ProP_{maternalhiv_{l,t,a,x}}$$

$$ProP_{maternalhiv_{l,t,a,x}} = PAF_{l,t,a,x,hivpos} \times rr_{mat}$$

Where:

$CF_{l,t,a,x,mat}$ = The proportion of deaths due to all maternal causes before HIV/AIDS correction for the location, year, age, and sex.

$CF_{l,t,a,x,mat_{adj}}$= The proportion of deaths due to maternal causes after the adjustment for the location, year, age, and sex.

$CF_{l,t,a,x,mat_{hiv}}$ = The proportion of deaths due to maternal deaths aggravated by HIV/AIDS after the adjustment for the location, year, age, and sex.

$PAF_{l,t,a,x,hivpos}$ = The PAF that describes the percentage of all maternal deaths that were HIV-related for the location, year, age, and sex

$ProP_{hiv_{l,t,a,x}}$ = The proportion of deaths in pregnancy for the location, year, age, and sex that are estimated to be incidental deaths due to HIV/AIDS and therefore not a maternal CoD.

$ProP_{maternalhiv_{l,t,a,x}}$ =The proportion of deaths in pregnancy for the location, year, age, and sex that are estimated to be HIV+ and maternal deaths that are aggravated by HIV/AIDS.

rr_{mat} = 0.13/1.13 = The proportion of HIV/AIDS deaths during pregnancy that were exacerbated by the pregnancy.

Section 2.13.4: HIV/AIDS correction of other maternal mortality data (step 11.4)

Although a specific subset of codes in ICD-10 corresponds to HIV/AIDS deaths aggravated by pregnancy, these codes are sparsely used and unreliable. We therefore adapted the method described to also correct VR and VA sources for the systematic exclusion of HIV-related maternal deaths. This correction was calculated in the same manner, by using the same input data as above, with the only difference being that HIV correction of VR and VA sources resulted in a net increase in the maternal correction factor maternal deaths aggravated by HIV/AIDS are calculated in the following way:

$$CF_{l,t,a,x,mat_{hiv}} = CF_{l,t,a,x,mat} \times ProP_{maternalhiv_{l,t,a,x}}$$

$$ProP_{maternalhiv_{l,t,a,x}} = \frac{PAF_{l,t,a,x,hivpos} \times rr_{mat}}{1 - PAF_{l,t,a,x,hivpos} \times rr_{mat}}$$

Section 2.14: Noise reduction (step 12)[1]

To deal with problems of zero counts in VR, VA, cancer registries, or sibling histories for a given age group in a given year, we use a Bayesian noise-reduction algorithm. For this algorithm, we assume a normal prior and a normal data likelihood. We estimate the normal prior for a given country-series of data by running a Poisson regression to estimate the number of deaths due to each respective cause and sex with dummy variables for age and year. With two notable exceptions (detailed below), these regressions are sex-, cause-, and country-specific, so borrowing strength over age and year is only within a given data type, country, cause, and sex. The variance of the prior, τ^2, is estimated from the Poisson regression, taking into account the variance-covariance matrix of the regression coefficients. For the data variance, we use the Wilson approximation which provides an estimate of σ^2 even in cases with a zero count of cause-specific deaths. The posterior estimate for each data point is

$$Mean = \left(\frac{\tau^2}{\tau^2 + \sigma^2} X + \frac{\sigma^2}{\tau^2 + \sigma^2} \mu\right)$$

$$Variance = \left(\frac{\tau^2 \sigma^2}{\tau^2 + \sigma^2}\right)$$

Where

X is the mean of the data

μ is the mean of the prior.

This approach to noise reduction avoids the problem that zero counts in an *ln* rates model or a logit cause fraction model will be dropped from the regression and lead to upward bias in the estimates. This is particularly important in two settings: high-income countries with small numbers of cause-specific

deaths, and the analysis of sibling history data where for any given age group in any given year the number of deaths reported in the survey that are pregnancy-related or the number of deaths from all causes in that age group may be small.

Regarding the exceptions to the regression, the first is that country-years with populations under 1 million are pooled with the region data to prevent over-dispersion and provide a stronger signal. Additionally, VA data diverge from the above description in two ways. First, all data for a given super-region are pooled together and a study dummy variable is added, allowing for different studies and surveillance sites to borrow strength from one another within a super-region. Second, unless the data are part of a time series (eg, the Matlab Health and Demographic Surveillance System), the regression has no year component.

Section 2.15: Cause of death database and outlier identification (step 13)[1]

Death rates for different causes of death generally have a stable age pattern. In large populations, these patterns will not change very rapidly over time. We can assume a relatively stable pattern in death rates for all causes except for some epidemic diseases and specific types of injuries. Rare causes in large populations and prevalent causes in small populations usually have stochastic patterns. To correct for these stochastic patterns, we implemented a noise-reduction process, explained in Step 12.

In VR data, we infrequently find one or more data points for specific geography/age/sex/year combinations that lie very far from the stable pattern of death rates. In these situations, the model usually ignores the data point(s). If the model fails to ignore these data, dramatic jumps or drops can occur in the death rates. When no logical explanation exists for variation in the death rates to this degree, we regard the data point(s) as outlier(s). The selection of data points to regard as outliers occurs after data have been prepped for modelling, as well as during preliminary reviews of the models.

In non-VR sources, data-collection methods and data quality can vary widely from source to source. Where data points in each age-sex-geography-year are very sparse, extreme data points can have a bad effect on regional estimation. In these situations, we investigate the study's methods and consider lower-quality data points as outliers.

Identifying outliers in the CoD data occurs prior to finalisation of models for each cause. We do not automate the selection of outliers but investigate the source of the offending data as well as reviewing other data sources for the same cause, geography, and year. Ultimately, outliers are identified based on the judgement of the modeller and senior faculty. Outlier decisions are reversible and may be revisited.

Section 2.16: Causes of death data star rating calculation[1]

GBD estimates are most accurate when computed with a full time series of complete VR with a low percentage of garbage codes. For GBD 2016, we developed a simple star-rating system from 0 to 5 to give a picture of the quality of data available in a given country over the full time series used in GBD estimates. Countries improve in the star rating as they increase availability, completeness, and detail of their mortality data and reduce the percentage of deaths coded to ill-defined garbage codes or highly

aggregated causes (table 7, figures 5a and 5b). Underlying indicators for the percent well-certified calculation are listed in table S8.

We assign star ratings to rate the quality of data for any given location year. Two dimensions determine this star rating: (I) the percentage of total deaths determined to be major garbage (such as ill-defined). Causes such as "injuries" or "cancer" will also be included in major garbage percentage because this percentage includes use of highly aggregated causes; and (II) the level of completeness of death registration. These two values were used to create a "percent well-certified" value between 0 and 1, determined as:

$$pct_{wellcertified} = Completeness \times (1 - pct_{majgarbage})$$

The mapping of percent well certified to star rating is as followed:

0 star: $0\% = pct_{wellcertified}$

1 star: $0\% < pct_{wellcertified} < 10\%$

2 star: $10\% <= pct_{wellcertified} < 35\%$

3 star: $35\% <= pct_{wellcertified} < 65\%$

4 star: $65\% <= pct_{wellcertified} < 85\%$

5 star: $pct_{wellcertified} \geq 85\%$

While stars are calculated for each five-year time interval as well as the full time series from 1980 to 2019, stars in the main text are presented for the full time series only.

In the case of VA, all garbage codes are considered ill-defined because redistribution for VA is highly imprecise.

For each VA data source, percent well-certified is

$$pct_{wellcertified} = VerbalAutopsyAdjustment \times (1 - pct_{majgarbage})$$

Where:

$$VerbalAutopsyAdjustment = SubAdj \times RegAdj \times AgeSexCoverage$$

SubAdj is 10% for subnationally representative studies; 100% for nationally representative studies. This adjustment, while arbitrary in its specific value, reflects the bias that can be associated with studies that only cover a potentially non-representative sample of a country's population.

RegAdj is 64% for all VA data sources. This accounts for the inaccuracy of VA in assigning CoD compared to medically verified VR. The specific multiplier 0·64 is based on the chance-corrected concordance of Physician Certified Verbal Autopsy (PCVA) versus medical certification by the Population Health Metrics Research Consortium.[35]

Age-Sex Coverage is the number of deaths estimated in the GBD mortality envelope for the ages and sexes in the study for the country and year divided by the number of deaths estimated in the GBD mortality envelope for the country and year. Studies that only cover children under 5 years or maternal mortality, for example, will be highly discounted by this multiplier.

Once percent well-certified is calculated for each location-year of VR and each VA study-year, we then combine these into one measurement for each five-year time interval and the full time series 1980–2019. For each five-year time interval, we take the maximum percent well-certified. Then for 1980–2019, we take the average of the maximum percentages well-certified for the seven five-year time intervals. Any five-year time interval in which no data were available were given a percent well-certified value of zero.

Prior to GBD 2019, the causes of death team used an all ages, both sex cause fraction to estimate the percentage of garbage coded deaths in a given location year. Thus, the percentage of garbage for a given location year was determined as:

$$CF_G = \frac{D_G}{D}$$

Where:

CF_G represents the cause fraction of percent garbage

D_G represents total garbage coded deaths

D represents the total deaths in a given location/year.

In GBD 2019, we moved to calculating the percentage of garbage coded deaths using an age-standardised cause fraction. The steps for creating these age-standardised cause fractions, in the case of garbage, are as follows:

1. Create both-sex, age-specific cause fractions of garbage for each age group
2. Scale these cause fractions by a set of both-sex age weights, determined by global mortality estimates from 2010 to present. That is, weights for each GBD age group were determined as:

$$W_a = \frac{D_a}{D}$$

Where:

W_a is the weight for given age group “a”

D_a is the total both sex, global deaths from 2010 to present in age group “a”

D is the total both sex, global deaths from 2010 to present across all ages.

3. Sum these weighted cause fractions across all age groups to produce the age-standardised cause fraction

In the case of percent garbage for a given location year, the formula to calculate percent garbage would be given as the sum of the weighted age specific cause fractions across all age groups "a":

$$CF_G = \Sigma_a(\frac{G_a}{D_a} \times W_a)$$

Where:

G_a represents the total both sex garbage deaths in age group "a"

D_a represents the total both sex deaths in age group "a"

W_a represents the weight generated from mortality estimates for age group "a"

ICD-10 and ICD-9 codes assigned to Level 1 or 2 garbage can be found in table S4.

Section 3: Causes of death modelling methods

Section 3.1: CODEm[1]

Section 3.1.1: Overview of methods

Cause of death ensemble modelling (CODEm) is the framework used to model most cause-specific death rates in the GBD.[36] It relies on four key components:

First, all available data are identified and gathered to be used in the modelling process. Although the data may vary in quality, they all contain some signal of the true epidemiological process.

Second, a diverse set of plausible models are developed to capture well-documented associations in the estimates. Using a wide variety of individual models to create an ensemble predictive model has been shown to outperform techniques using only a single model both in CoD estimation[36] and in more general prediction applications.[37,38]

Third, the out-of-sample predictive validity is assessed for all individual models, which are then ranked for use in the ensemble modelling stage.

Finally, differently weighted combinations of individual models are evaluated to select the ensemble model with the highest out-of-sample predictive validity.

For some causes (eg, lower respiratory infections), evidence exists that the relationship between covariates and death rates might differ between children and adults. Separate models are therefore run for different age ranges, when applicable. Additionally, separate models are developed for countries with extensive, complete, and representative VR for every cause to ensure that uncertainty can better reflect the more complete data in these locations.

In order to ensure the addition of subnational locations are not driving changes in estimates, in GBD 2019, we run a global model that excludes data from non-standard locations; the resulting covariate

betas are then used as priors for the true global model.

In addition to CoD modelling, we also estimate fatal discontinuities. Fatal discontinuities are events that are stochastic in nature, that cannot be modelled because they do not have a predictable time trend. The fatal discontinuities by cause are aggregated by age and sex and added to the estimated number of deaths in CoD modelling for those causes during CoDCorrect. Details on their methods can be found in Section 3.4.

Section 3.1.2: Model pool development

Because many factors may co-vary with any given CoD, a range of plausible statistical models are developed for each cause. In the CODEm framework, four families of statistical models are used: linear mixed effects regression (LMER) models of the natural log of the cause-specific death rate, LMER models of the logit of the cause fraction, spatiotemporal Gaussian process regression (ST-GPR) models of the natural logarithm of the cause-specific death rate, and ST-GPR models of the logit of the cause fraction (see the 2x2 table in Foreman et al).[36] For more on ST-GPR, see section 4.3.3. For each family of models, all plausible relationships between covariates and the response variable are identified. Because all possible combinations of selected covariates are considered for each family of models, multi-collinearity between covariates may produce implausible signs on coefficients or unstable coefficients. Each combination is therefore tested for statistical significance (covariate coefficients must have a coefficient with p-value < 0·05) and plausibility (the coefficients must have the directions expected on the basis of the literature). Only covariate combinations meeting these criteria are retained. This selection process is run for both cause fractions and death rates, then ST-GPR and LMER-only models are created for each set of covariates. For a detailed explanation of the covariate selection algorithm, see Foreman et al.[36]

Section 3.1.3: Data variance estimation

The families of models that go through ST-GPR described in Section 3.1.2 incorporate information about data variance. The main inputs for a Gaussian process regression (GPR) are a mean function, a covariance function, and data variance for each data point. These inputs are described in detail in Foreman et al.[36] For GBD 2019, we have updated this calculation to incorporate garbage code redistribution uncertainty.

Three components of data variance are now used in CODEm: sampling variance, non-sampling variance, and garbage code redistribution variance. The computation of sampling variance and non-sampling variance has not changed since previous iterations of the GBD and is also described in Foreman et al.[36] Garbage code redistribution variance is computed in the CoD database process described in Section 2.7 of this appendix. Since variance is additive, we calculate total data variance as the sum of sampling variance, non-sampling variance, and redistribution variance. Increased data variance in GPR results in the GPR draws not following the data point as closely.

Section 3.1.4: Testing model pool on 15% sample

The performance of all models (individual and ensemble) is evaluated by means of out-of-sample predictive validity tests. Thirty percent of the data are randomly excluded from the initial model fits.

These individual model fits are evaluated and ranked by using half of the excluded data (15% of the total), then used to construct the ensembles on the basis of their performance. Data are held out from the analysis on the basis of the cause-specific missingness patterns for ages and years across locations. Out-of-sample predictive validity testing is repeated 20 times for each model, which has been shown to produce stable results.[36] These performance tests include the root mean square error (RMSE) for the log of the cause-specific death rate, the direction of the predicted versus actual trend in the data, and the coverage of the predicted 95% UI.

Section 3.1.5: Ensemble development and testing

The component models are weighted on the basis of their predictive validity rank to determine their contribution to the ensemble estimate. The relative weights are determined both by the model ranks and by a parameter ψ, whose value determines how quickly the weights taper off as rank decreases. The distribution of ψ is described in more detail in Foreman et al.[36] A set of ensemble models is then created by using the weights constructed from the combinations of ranks and ψ values. These ensembles are tested by using the predictive validity metrics described in Section 3.1.4 on the remaining 15% of the data, and the ensemble with the best performance in out-of-sample trend and RMSE is chosen as the final model.

Section 3.1.6: Final estimation

Once a weighting scheme has been chosen, 1000 draws are created for the final ensemble, and the number of draws contributed by each model is proportional to its weight. The mean of the draws is used as the final estimate for the CODEm process, and a 95% UI is created from the 0·025 and 0·975 quantiles of the draws. The validity of the UI can be checked via its coverage of the out-of-sample data; ideally, the 95% UI would capture 95% of these data. Higher coverage suggests that the UIs are too large, and lower coverage suggests overfitting.

Section 3.1.7: Selection of causes for which CODEm is used

CODEm is used to model 193 causes, described in detail in Section 3.3. However, it is unsuitable for use in modelling certain causes, including those with very low death counts, those where cause-specific death record availability is inadequate, or those for which there are marked biases or variability for CoD certification over time that cannot be fully accounted for with the current garbage code redistribution algorithms. Criteria for causes where CODEm is not used are discussed in further detail in Section 3.2.

Section 3.1.8: Model-specific covariates

Modellers select covariates to be used in CODEm, but those covariates may not be significant or in the direction specified during the covariate selection step of CODEm and will therefore not be used in the model. These covariates are listed with a '—' for number of draws. Additionally, covariates may be selected by CODEm but only exist in submodels that perform poorly and may end up with zero draws included in the final ensemble. Finally, all other covariates are listed with the number of draws in the final ensemble from submodels that had the covariate.

Section 3.2: Causes modelled outside of CODEm[1]

Section 3.2.1: Overview

A number of causes required alternative modelling strategies to those used for CODEm because they were not compatible with CODEm estimation infrastructure and processes. Such unsuitability included having very low death counts; inadequate availability of cause-specific death records; and marked biases or variability for CoD certification over time that could not be fully accounted for with current garbage code redistribution algorithms. The inclusion of these causes in CODEm often renders its out-of-sample predictive validity testing unstable, but the validity of this type of testing is a key advantage of using CODEm for CoD estimation. Alternately, CODEm simply fails to generate plausible mortality rates in the absence of enough VR or VA data when these causes are included. Because of increased data availability and redistribution algorithm refinements, we were able to incorporate several new causes, which were modelled separately for GBD 2013, into CODEm for this iteration of the GBD study; with each annual update of GBD, we aim to add more causes within the CODEm estimation space. For GBD 2019, we used alternative modelling approaches for these causes, including negative binomial models, natural history models, sub-cause proportion models, and prevalence-based models (table S10).

Section 3.2.2: Negative binomial models

For eight rare causes of death, too few observed deaths were included in the CoD database to produce stable estimates. For these causes, we ran negative binomial regression models, with either a constant or a constant multiplied by the mean assumption for the dispersion parameter, by using reverse step-wise model building. We selected one of the two model dispersion assumptions based on best fit to the data by using the same method as GBD 2013. For GBD 2015, we also tested zero-inflated Poisson models for these rare causes of death but rejected them after finding that they did not substantially affect the mean predictions but instead produced unrealistically large UIs. Descriptions of the modelling process for each of these causes follows in the next sections.

Section 3.2.3: DisMod-MR 2.1

Until GBD 2010, non-fatal estimates were based on a single data source on prevalence, incidence, remission, or a mortality risk selected by the researcher as most relevant to a particular location and time. For GBD 2010, we set a more ambitious goal: to evaluate all available information on a disease that passes a minimum quality standard. That required a different analytical tool that would be able to pool disparate information presented in varying age groupings and from data sources by using different methods. The DisMod-MR 1.0 tool used in GBD 2010 evaluated and pooled all available data, adjusted data for systematic bias associated with methods that varied from the reference, and produced estimates with UIs by world regions. For GBD 2013, the improved DisMod-MR 2.0 had increased computational speed, allowing computations that were consistent between all disease parameters at the country rather than the region level. The hundred-fold increase in speed of DisMod-MR 2.0 was partly due to a more efficient rewrite of the code in C++ but also to changing to a model specification using log rates rather than a negative binomial model used in DisMod-MR 1.0. In cross-validation tests, the log rates specification worked as well as or better than the negative binomial specification.[39] For GBD 2015, the computational engine (DisMod-MR 2.1) remained substantively unchanged, but we re-

wrote the wrapper code that organised the flow of data and settings at each level of the analytical cascade. The sequence of estimation occurred at five levels: global, super-region, region, country, and, where applicable, subnational locations (see flow diagram of DisMod-MR 2.1 cascade that follows). The super-region priors were generated at the global level with mixed-effects, non-linear regression by using all available data; the super-region fit, in turn, informed the region fit and so on down the cascade. The wrapper gave analysts the choice to branch the cascade in terms of time and sex at different levels depending on data density. The default used in most models was to branch by sex after the global fit but to retain all years of data until the lowest level in the cascade. For GBD 2015, we generated fits for the years 1990, 1995, 2000, 2005, 2010, and 2015.

In updating the wrapper, we consolidated the code base into a single language, Python, to make the code more transparent and efficient and to better deal with subnational estimation. The computational engine is limited to three levels of random effects; we differentiated estimates at the super-region, region, and country levels. In GBD 2013, the subnational units of China, Mexico, and the UK were treated as countries, such that a random effect was estimated for every location with contributing data. However, the lack of a hierarchy between country and subnational units meant that the fit to country data contributed as much to the estimation of a subnational unit as the fits for all other countries in the region. We found inconsistency between the country fit and the aggregation of subnational estimates when the country's epidemiology varied from the average of the region. Adding an additional level of random effects required a prohibitively comprehensive rewrite of the underlying DisMod-MR engine. Instead, we added a fifth layer to the cascade, with subnational estimation informed by the country fit and country covariates, plus an adjustment based on the average of the residuals between the subnational unit's available data and its prior. This procedure mimicked the impact of a random effect on estimates between subnationals.

For GBD 2015, we improved how country covariates differentiate non-fatal estimates for diseases with sparse data. The coefficients for country covariates were re-estimated at each level of the cascade. For a given location, country coefficients were calculated by using both data and prior information available for that location. In the absence of data, the coefficient of its parent location was chosen to utilise the predictive power of our covariates in data sparse situations.

For GBD 2017, the DisMod-MR 2.1 tool was used. Updates included estimation of new age groups through the GBD 2017 terminal age group of 95 years and older in addition to the new locations added for the GBD 2017 cycle.

Section 3.2.4: DisMod-MR 2.1 likelihood estimation

Analysts have the choice of using a Gaussian, log-Gaussian, Laplace, or log-Laplace likelihood function in DisMod-MR 2.1. The default log-Gaussian equation for the data likelihood is as follows:

$$-log\left[p\left(y_j\middle|\Phi\right)\right] = \log\left(\sqrt{2\pi}\right) + \log\left(\delta_j + s_j\right) + \frac{1}{2}\left(\frac{\log\left(a_j + \eta_j\right) - \log\left(m_j + \eta_j\right)}{\delta_j + s_j}\right)^2$$

Where:

y_j is a measurement value (ie, data point)

Φ denotes all model random variables

η_j is the offset value, *eta*, for a particular integrand (prevalence, incidence, remission, excess mortality rate, with-condition mortality rate, cause-specific mortality rate, relative risk, or standardised mortality ratio)

a_j is the adjusted measurement for data point j, defined by

$$a_j = e^{(-u_j - c_j)} y_j$$

Where:

u_j is the total area effect (ie, the sum of the random effects at three levels of the cascade: super-region, region, and country)

c_j is the total covariate effect (ie, the mean combined fixed effects for sex, study-level, and country-level covariates), defined by

$$c_j = \sum_{k=0}^{K[I(j)]-1} \beta_{I(j),k} \hat{X}_{k,j}$$

with standard deviation (SD)

$$s_j = \sum_{l=0}^{L[I(j)]-1} \zeta_{I(j),l} \hat{Z}_{k,j}$$

Where:

k denotes the mean value of each data point in relation to a covariate (also called x-covariate)

$I(j)$ denotes a data point for a particular integrand, j

$\beta_{I(j),k}$ is the multiplier of the kth x-covariate for the ith integrand

$\hat{X}_{k,j}$ is the covariate value corresponding to the data point j for covariate k

l denotes the SD of each data point in relation to a covariate (also called z-covariate)

$\zeta_{I(j),k}$ is the multiplier of the lth z-covariate for the ith integrand

δ_j is the SD for adjusted measurement j, defined by

$$\delta_j = log[y_j + e^{(-u_j - c_j)}\eta_j + c_j] - log[y_j + e^{(-u_j - c_j)}\eta_j]$$

Where mj denotes the model for the jth measurement, not counting effects or measurement noise and defined by

$$m_j = \frac{1}{B(j) - A(j)} \int_{A(j)}^{B(j)} I_j(\text{a})\ \text{da}$$

Where:

$A(j)$ is the lower bound of the age range for a data point j

$B(j)$ is the upper bound of the age range for a data point j

$I(j)$ denotes the function of age corresponding to the integrand for data point j

The source code for DisMod-MR 2.1 as well as the wrapper code is available at https://github.com/ihmeuw/ihme-modelling/tree/master/gbd_2017/shared_code/central_comp/nonfatal/dismod.

Section 3.2.5: Natural history models

For some causes for which CoD data may be systematically biased either owing to misclassification or because the disease exists in focal communities without VR or VA studies, we have developed natural history models. In natural history models, incidence and case-fatality rates are modelled separately and then combined to produce estimates of cause-specific mortality.

Section 3.2.6: Prevalence-based models

The modelling strategies for atrial fibrillation and flutter are distinct from those used for other causes modelled as natural history models. These models use prevalence estimates and excess mortality rates (EMR) generated through DisMod-MR 2.1 rather than incidence and case-fatality rates.

Section 3.2.7: Sub-cause proportion models

For certain sub-causes for which accurate diagnoses are known to be very difficult, we first modelled the parent cause in the GBD hierarchy with CODEm and then allocated deaths to specific causes by using proportions of the parent cause for each age-sex-location-year for each sub-cause. For these causes, we identified no significant predictors in negative binomial regressions. This approach was taken because the available data on these specific causes may come from sources other than VR, such as end-stage renal disease registries, or may come from too few places to model the death rates directly. Details for each cluster of causes analysed in this way follow.

Section 3.3: Central computation[1]

Section 3.3.1: Imported cases

Imported cases are fatalities that occur in a geographic area where a particular CoD is known to be eradicated in a specific time period or where infection cannot occur. We apply space-time restrictions to these causes in the modelling strategy for that location and time period. However, in some rare cases, deaths from these causes occur outside of restricted locations and time periods. These deaths are referred to as imported cases.

Illustrating this concept, Chagas disease is transmitted by insect vectors that only exist in the Americas. For this reason, Chagas disease is restricted in the models for countries such as Russia. However, someone traveling in Latin America could contract Chagas disease and then die after returning home to Russia. Imported cases accounts for these kinds of deaths.

To calculate these imported cases, we find all cases from the VRs of data-rich countries for any CoD that is otherwise geographically or temporally restricted. We then create a beta distribution from that data point by using the sample size of the VR for that data point and upload these draws as a custom CoD model. This model is then used as an input to CoDCorrect.

Section 3.3.2: CoDCorrect

Section 3.3.2.1 Objective of CoDCorrect

As mentioned in the main text, the CoD models are cause-specific. As such, there is no guarantee that the sum of these models will equal the results of the all-cause mortality estimates or that model results of child causes add up to the parent model results. The CoDCorrect process is used to make the CoD and all-cause mortality estimates internally consistent by using a very simple algorithm.

Section 3.3.2.2 Algorithm and levels

The core algorithm remains the same as it did in GBD 2013. The equation can be written as follows:

$$CD_{lyasjd} = D_{lyasjd}\left(\frac{PD_{lyasjd}}{\sum_{j=1}^{j=k} D_{lyasjd}}\right)$$

Where:

CD_{lyasjd} is the corrected number of deaths for a location l, year y, age a, sex s, cause j, and draw d

PD_{lyasjd} is the parent CoD for a location l, year y, age a, sex s, cause j, and draw d

D_{lyasjd} is the uncorrected number of deaths estimated from a cause-specific model for a l, year y, age a, sex s, cause j, and draw d

The CoDCorrect process starts by rescaling the Level 1 causes to match the all-cause mortality estimates (used for PD_{lyasjd} in the previous equation). Level 2 causes are then rescaled to their corrected parent causes. This process continues until all levels of the hierarchy have been rescaled. Causes and their levels within the CoDCorrect hierarchy can be found in table S9.

Since GBD 2017, HIV has not been included in the CoDCorrect process. To account for this change, Level 1 CoDCorrect causes are rescaled to HIV-deleted mortality estimates that are produced as part of the mortality and HIV estimation process. Results from the GBD version of Spectrum are added to the post-CoDCorrect death estimates with fatal discontinuities and imported cases to generate the full set of death estimates.

Section 3.3.2.3 Diagnostic results of CoDCorrect by cause and location

For more detail on diagnostic results of CodCorrect by cause see table S15.

Section 3.3.3: Years of life lost calculation

Years of life lost (YLLs) owing to premature mortality were computed for 1082 locations and 39 years. First, we used the lowest observed age-specific mortality rates by location and sex across all estimation years from locations with total populations greater than 5 million in 2016 to establish a theoretical minimum risk reference life table.

The YLL is a metric that is computed by multiplying the number of estimated deaths by the standard life expectancy at age of death. The metric therefore highlights premature deaths by applying a larger weight to deaths that occur in younger age groups. We propagated uncertainty from CoDCorrected deaths for all demographics. The core equation can be written as follows:

$$YLL = \sum_{c=1,a=0,s=1}^{\infty} d_{cas} e_a$$

Section 3.3.4: GBD world population age standard

Age-standardised populations in the GBD were calculated by using the GBD world population age standard. For GBD 2013, GBD 2015, and GBD 2016, the age-specific proportional distributions of all national locations from the UN Population Division World Population Prospects 2012 revision for all years from 2010 to 2035 were used to generate a standard population age structure by using the non-weighted mean across all the aforementioned country-years. For GBD 2017, we used the non-weighted mean of 2017 age-specific proportional distributions from the GBD 2017 population estimates for all national locations with a population greater than 5 million people in 2017 to generate an updated standard population age structure.[40] For GBD 2019, we have continued to use this method using GBD 2019 population estimates.[8]

References

1 Roth GA, Abate D, Abate KH, *et al.* Global, regional, and national age-sex-specific mortality for 282 causes of death in 195 countries and territories, 1980–2017: a systematic analysis for the Global Burden of Disease Study 2017. *The Lancet* 2018; **392**: 1736–88.

2 James SL, Abate D, Abate KH, *et al.* Global, regional, and national incidence, prevalence, and years lived with disability for 354 diseases and injuries for 195 countries and territories, 1990–2017: a systematic analysis for the Global Burden of Disease Study 2017. *The Lancet* 2018; **392**: 1789–858.

3 Kyu HH, Abate D, Abate KH, *et al.* Global, regional, and national disability-adjusted life-years (DALYs) for 359 diseases and injuries and healthy life expectancy (HALE) for 195 countries and territories, 1990–2017: a systematic analysis for the Global Burden of Disease Study 2017. *Lancet* 2018; **392**: 1859–922.

4 Stanaway JD, Afshin A, Gakidou E, *et al.* Global, regional, and national comparative risk assessment of 84 behavioural, environmental and occupational, and metabolic risks or clusters of risks for 195 countries and territories, 1990–2017: a systematic analysis for the Global Burden of Disease Study 2017. *The Lancet* 2018; **392**: 1923–94.

5 Stevens GA, Alkema L, Black RE, *et al.* Guidelines for Accurate and Transparent Health Estimates Reporting: the GATHER statement. *The Lancet* 2016; **388**: e19–23.

6 Sankoh O, Byass P. The INDEPTH Network: filling vital gaps in global epidemiology. *Int J Epidemiol* 2012; **41**: 579–88.

7 Lozano R, Freeman MK, James SL, *et al.* Performance of InterVA for assigning causes of death to verbal autopsies: multisite validation study using clinical diagnostic gold standards. *Popul Health Metr* 2011; **9**: 50.

8 GBD 2019 Demographics Collaborators. Global, regional, and national age-sex-specific fertility, mortality, and population estimates, 1950–2019: a comprehensive demographic analysis for the Global Burden of Disease Study 2019. *The Lancet* In Press.

9 Office of the Registrar General and Census Commissioner. India Medical Certification of Cause of Death Reports 1990-2010. India, 2014.

10 Office of the Registrar General and Census Commissione. India Sample Registration System Statistical Report 2017. India, 2018.

11 Todd S, Barr S, Passmore AP. Cause of death in Alzheimer's disease: a cohort study. *QJM Mon J Assoc Physicians* 2013; **106**: 747–53.

12 Brunnström HR, Englund EM. Cause of death in patients with dementia disorders. *Eur J Neurol* 2009; **16**: 488–92.

13 Keene J, Hope T, Fairburn CG, Jacoby R. Death and dementia. *Int J Geriatr Psychiatry* 2001; **16**: 969–74.

14 Thomas BM, Starr JM, Whalley LJ. Death certification in treated cases of presenile Alzheimer's disease and vascular dementia in Scotland. *Age Ageing* 1997; **26**: 401–6.

15 Naghavi M, Makela S, Foreman K, O'Brien J, Pourmalek F, Lozano R. Algorithms for enhancing public health utility of national causes-of-death data. *Popul Health Metr* 2010; **8**: 9.

16 Barker B, Degenhardt L, National Drug and Alcohol Research Centre (Australia). Accidental drug-induced deaths in Australia 1997-2001. Sydney, Australia: National Drug and Alcohol Research Centre, University of New South Wales, 2003.

17 Roxburgh A, Burns L. Accidental drug-induced deaths due to opioids in Australia, 2011. Sydney, Australia: National Drug and Alcohol Research Centre, University of New South Wales, 2015.

18 Roxburgh A, Burns L. Cocaine and methamphetamine related drug-induced deaths in Australia, 2011. Sydney, Australia: National Drug and Alcohol Research Centre, University of New South Wales, 2015.

19 Naghavi M, Wang H, Lozano R, *et al.* Global, regional, and national age-sex specific all-cause and cause-specific mortality for 240 causes of death, 1990–2013: a systematic analysis for the Global Burden of Disease Study 2013. *The Lancet* 2015; **385**: 117–71.

20 Lozano R, Naghavi M, Foreman K, *et al.* Global and regional mortality from 235 causes of death for 20 age groups in 1990 and 2010: a systematic analysis for the Global Burden of Disease Study 2010. *The Lancet* 2012; **380**: 2095–128.

21 Wang H, Abajobir AA, Abate KH, *et al.* Global, regional, and national under-5 mortality, adult mortality, age-specific mortality, and life expectancy, 1970–2016: a systematic analysis for the Global Burden of Disease Study 2016. *The Lancet* 2017; **390**: 1084–150.

22 Calvert C, Ronsmans C. The contribution of HIV to pregnancy-related mortality: a systematic review and meta-analysis. *Aids* 2013; **27**: 1631–9.

23 Figueroa-Damián R. Pregnancy outcome in women infected with the human immunodeficiency virus. *Salud Publica Mex* 1999; **41**: 362–7.

24 Ryder RW, Nsuami M, Nsa W, *et al.* Mortality in HIV-1-seropositive women, their spouses and their newly born children during 36 months of follow-up in Kinshasa, Zaïre. *AIDS Lond Engl* 1994; **8**: 667–72.

25 Zvandasara P, Saungweme G, Mlambo JT, Moyo J. Post Caesarean section infective morbidity in HIV-positive women at a tertiary training hospital in Zimbabwe. *Cent Afr J Med* 2007; **53**: 43–7.

26 Chilongozi D, Wang L, Brown L, *et al.* Morbidity and mortality among a cohort of human immunodeficiency virus type 1-infected and uninfected pregnant women and their infants from Malawi, Zambia, and Tanzania. *Pediatr Infect Dis J* 2008; **27**: 808–14.

27 Leroy V, Ladner J, Nyiraziraje M, *et al.* Effect of HIV-1 infection on pregnancy outcome in women in Kigali, Rwanda, 1992-1994. Pregnancy and HIV Study Group. *AIDS* 1998; **12**: 643–50.

28 Kourtis A, Bansil P, McPheeters M, Meikle S, Posner S, Jamieson D. Hospitalizations of pregnant HIV-infected women in the USA prior to and during the era of HAART, 1994–2003. *AIDS* 2006; **20**: 1823–31.

29 Ticconi C, Mapfumo M, Dorrucci M, *et al.* Effect of maternal HIV and malaria infection on pregnancy and perinatal outcome in Zimbabwe. *Jaids J Acquir Immune Defic Syndr* 2003; **34**: 289–94.

30 Brown T, Peerapatanapokin W. The Asian Epidemic Model: a process model for exploring HIV policy and programme alternatives in Asia. *Sex Transm Infect* 2004; **80**: i19–24.

31 DerSimonian R, Laird N. Meta-analysis in clinical trials. *Control Clin Trials* 1986; **7**: 177–88.

32 Matthews LT, Kaida A, Kanters S, *et al.* HIV-infected women on antiretroviral treatment have increased mortality during pregnant and postpartum periods. *AIDS Lond Engl* 2013; **27 Suppl 1**: S105-112.

33 Westreich D, Maskew M, Evans D, Firnhaber C, Majuba P, Sanne I. Incident pregnancy and time to death or AIDS among HIV-positive women receiving antiretroviral therapy. *PloS One* 2013; **8**: e58117.

34 Stover J. AIM: A computer program for making HIV/AIDS projections and examining the social and economic impact of AIDS. Glastonbury, CT: Futures Group, 2005.

35 Lozano R, Lopez AD, Atkinson C, Naghavi M, Flaxman AD, Murray CJ. Performance of physician-certified verbal autopsies: multisite validation study using clinical diagnostic gold standards. *Popul Health Metr* 2011; **9**: 32.

36 Foreman KJ, Lozano R, Lopez AD, Murray CJ. Modeling causes of death: an integrated approach using CODEm. *Popul Health Metr* 2012; **10**: 1.

37 Bell RM, Koren Y. Lessons from the Netflix Prize Challenge. *SIGKDD Explor Newsl* 2007; **9**: 75–79.

38 Bell RM, Koren Y, Volinsky C. All Together Now: A Perspective on the Netflix Prize. *CHANCE* 2010; **23**: 24–9.

39 Flaxman AD, Vos T, Murray CJL, Kiyono P, editors. An integrative metaregression framework for descriptive epidemiology, 1 edition. Seattle: University of Washington Press, 2015.

40 Murray CJL, Callender CSKH, Kulikoff XR, *et al.* Population and fertility by age and sex for 195 countries and territories, 1950–2017: a systematic analysis for the Global Burden of Disease Study 2017. *Lancet* 2018; **392**: 1995–2051.

Section 4: Non-fatal outcome estimation[2]

The GBD 2019 non-fatal estimation process describes the steps necessary to estimate incidence, prevalence, and YLDs for disease and injury sequelae in GBD 2019. Conceptually, the estimation effort is divided into eight major components: (1) compiling data sources through data identification and extraction; (2) data adjustment; (3) estimation of prevalence and incidence by cause and sequelae by using DisMod-MR 2.1 or alternative modelling strategies for selected cause groups; (4) estimation by impairment; (5) severity distributions; (6) incorporation of disability weights (DWs); (7) comorbidity adjustment; and (8) the estimation of YLDs by sequelae and causes. Section 4.12 contains additional detail specific to each non-fatal disease, impairment, and injury, and their sequelae. Non-fatal modelling strategies vary significantly between causes.

Section 4.1: Data sources, identification, and extraction[2]

Section 4.1.1: Systematic reviews

For GBD 2019, updated systematic reviews were conducted for 49 causes. Over 123,925 studies were screened for inclusion, and over 1250 articles were newly incorporated into GBD 2019 non-fatal models. For other disease sequelae, only a small fraction of the existing data appears in the published literature, and other sources predominate, such as survey data, disease registers, notification data, or hospital inpatient data. As was done in past rounds of GBD, data were systematically screened from household surveys archived in the GHDx (http://ghdx.healthdata.org/), including Demographic and Health Surveys, Multiple Indicator Cluster Surveys, Living Standards Measurement Surveys, and Reproductive Health Surveys. Other national health surveys were identified on the basis of survey series that had yielded usable data for past rounds of GBD, sources suggested to us by in-country collaborators, and surveys identified in major multinational survey data catalogues such as the International Household Survey Network and the WHO Central Data Catalog, as well as through country Ministry of Health and Central Statistical Office websites. Case notifications reported to the WHO were updated through 2019. Citations for all data sources used for non-fatal estimation in GBD 2019 are provided in searchable form through a web tool (http://ghdx.healthdata.org/). A description of the search terms used for cause-specific systematic reviews are detailed by cause in Section 4.12.

Section 4.1.2: Survey data preparation

For GBD 2019, survey data for which we have access to the unit record data constitute a substantial part of the underlying data used in the estimation process. During extraction, we concentrated on demographic variables (eg, location, sex, age), survey design variables (eg, sampling strategy and sampling weights), and the variables used to define the population estimate (eg, prevalence or a proportion) and a measure of uncertainty (standard error, confidence interval or sample size, and number of cases).

Section 4.1.3: Disease registries

For GBD 2019 non-fatal estimation, disease registries were an important source for a select number of conditions such as cancers, end-stage renal disease, and congenital disorders.

Registry data is particularly key in the estimation of neoplasms when we consider the increasing attention to non-communicable diseases, particularly cancers, in low and middle-income areas of the world. The GHDx source tool (http://ghdx.healthdata.org/data-type/disease-registry) provides a comprehensive list of registry data used in GBD estimation processes.

Section 4.1.4: Estimation of hospital envelope

Figure A. Overview process of estimation of hospital envelope.
This process utilises administrative data, reported tabulations, and survey microdata to estimate the rates of inpatient admissions per capita for every location and demographic group in the GBD hierarchy.

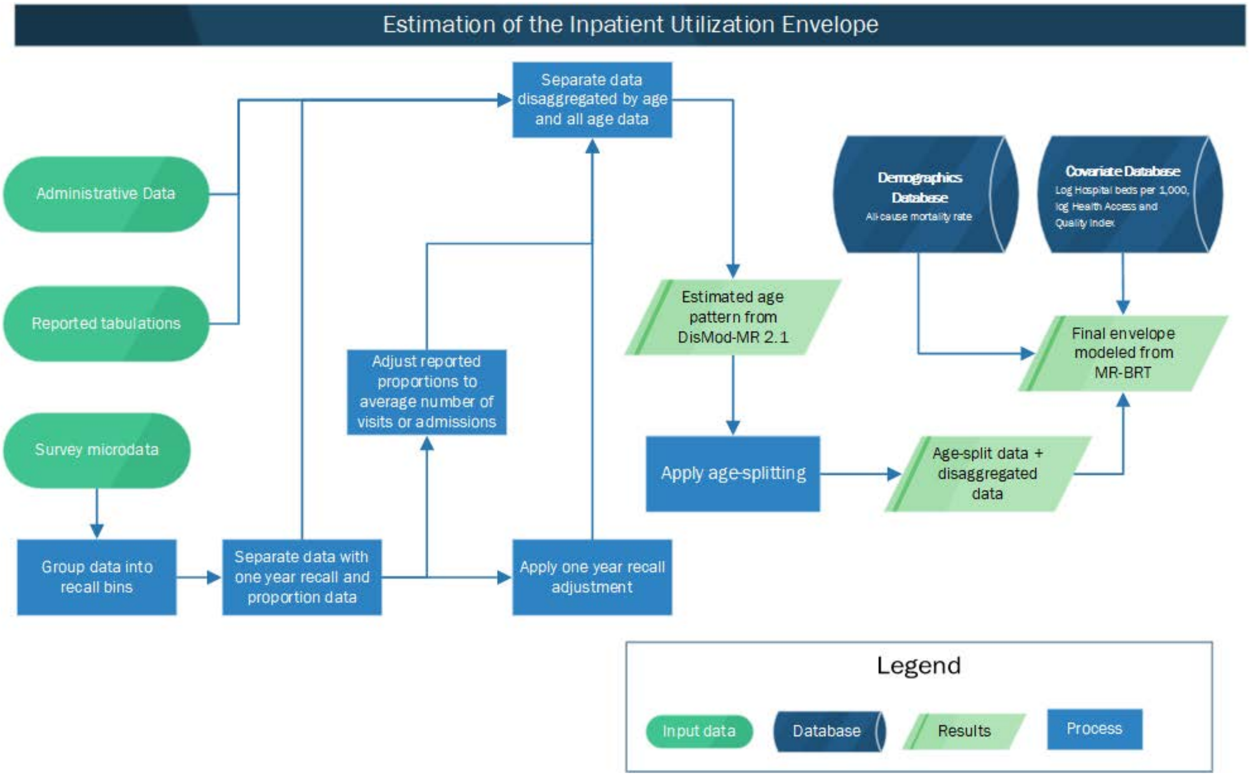

Section 4.2: Input data and methods summary[2]

Section 4.2.1: Case definition

We defined a hospital admission as admission into a formal health care facility for an overnight stay. However, we excluded admissions to long-term care facilities (>120 days), nursing care facilities, and facilities staffed by traditional or spiritual healers.

Section 4.2.2: Input data

We searched the GHDx for population surveys, administrative records, and censuses from January 1990 to September 2017. We applied the following keyword filters: “Health care use” OR “Length of stay” AND “Hospitals” OR “Health care services”. We applied no language restrictions to our search and required all returned records to contain either microdata or tabulated reports. We searched the returned records’ metadata for measures of inpatient care. For inclusion, we required all measures to be

nationally or subnationally representative. Additionally, we consulted with experts and GBD collaborators to gather data sources that were not within the GHDx.

To estimate inpatient admission rates for newborns, we input estimates of the in-facility delivery (IFD) rates for every subnational and national location at 5-year intervals starting at 1990 and including the most recent 2019 estimate. IFD was estimated by using an ST-GPR model based on population-representative surveys and administrative data. We accepted data sources from 28,646 location-years (1413 from administrative records and 27,233 from population surveys).

Section 4.3: Modelling strategy[2]

Section 4.3.1: Data adjustment

We classified each of the accepted data sources into four data types: (1) proportion of survey respondents who were admitted into the hospital in the last 30 days; (2) proportion of survey respondents who were admitted to the hospital in the last year; (3) average number of admissions (utilisation rate) reported by survey respondents in the last year; and (4) average number of visits reported by annual administrative records. We assigned measures reported by annual administrative records as our reference group because these data types were free from recall bias and most closely matched our case definition. From data sources for which microdata were available, we extracted and binned the data based on gender and age groups of less than 1 year, 1–4 years, 4–9 years, 10–14 years, and similar increments of years up to 95 years and older.

We crosswalked each of the three non-reference (survey) data types to the reference (administrative record) data type through the use of penalised spline regressions to account for non-systematic differences between the data types. For each non-reference data type and each sex, we looked for overlap between the non-reference data type and the reference data type based on location, year, age group, and sex. With the overlapping data, we calculated the ratio of the point estimate from the reference data type, μ_{ref}, to the non-reference data type, μ_s. We fit these ratios with a penalised spline regression equation

$$\ln\left(\frac{\mu_{ref,i}}{\mu_{s,i}}\right) = h(age_i) + \varepsilon_i \ (1)$$

Where:

i denotes a given matched observation

$h(age_i)$ represents a basis function that estimated a cross-validated, penalised spline over the population weighted mean age of the age group

ε represents the residual

In the figures that follow, for each non-reference data type, we plot the ratio of μ_{ref} and μ_s across age and by sex and the predictions from the penalised spline regressions.

Figure B. Global age-sex specific crosswalks to equate each non-reference data type to the reference data type.

For each non-reference data type and each sex, we plotted the ratio of reference data points to non-reference data points, which were matched based on location, age group, year, and sex. Using a penalized spline regression, we estimated the crosswalk between each non-reference data type and the reference type. We plotted the crosswalk and the associated prediction error in the following figures:

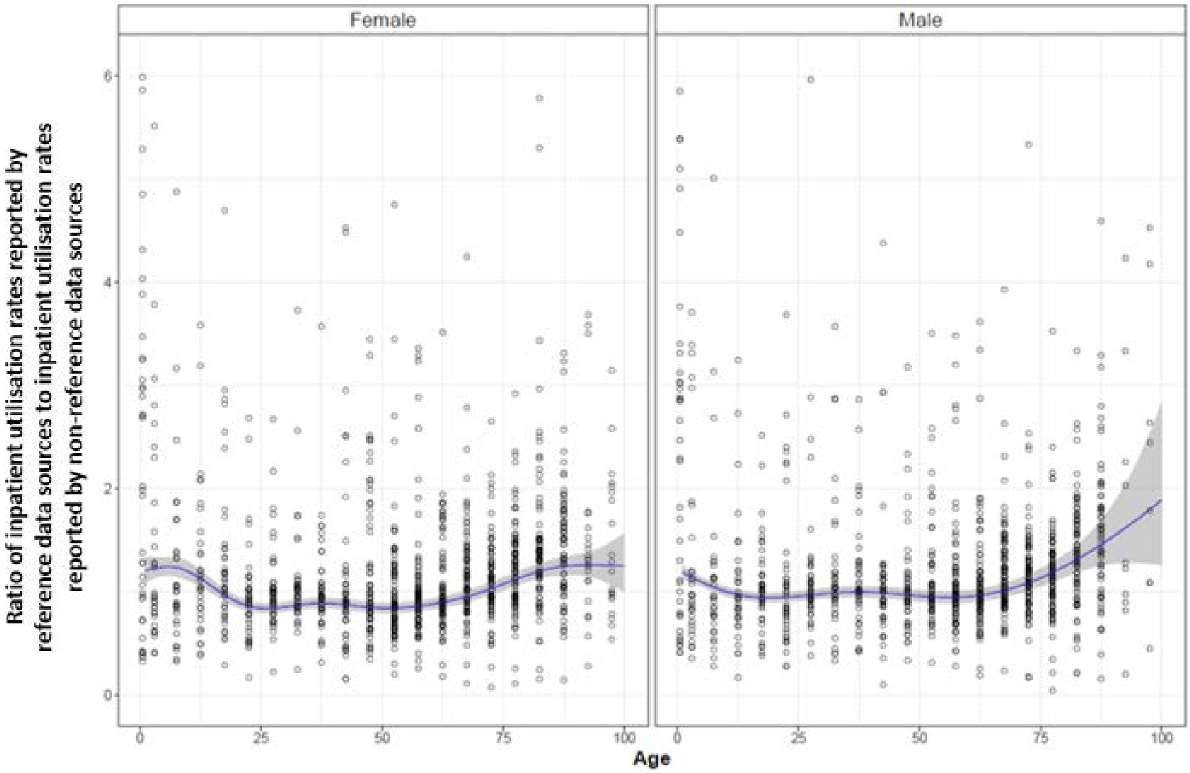

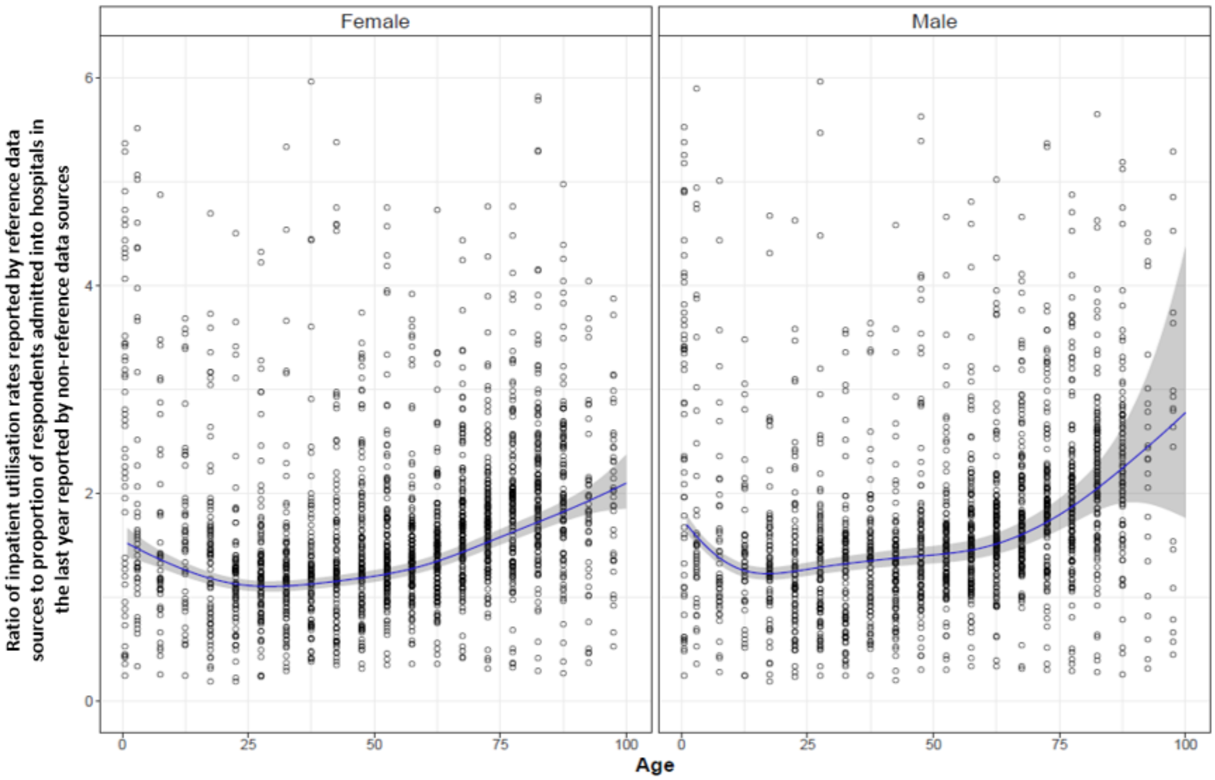

Female
Male
Ratio of inpatient utilisation rates reported by reference data sources to proportion of respondents admitted into hospitals in the last year reported by non-reference data sources
Age

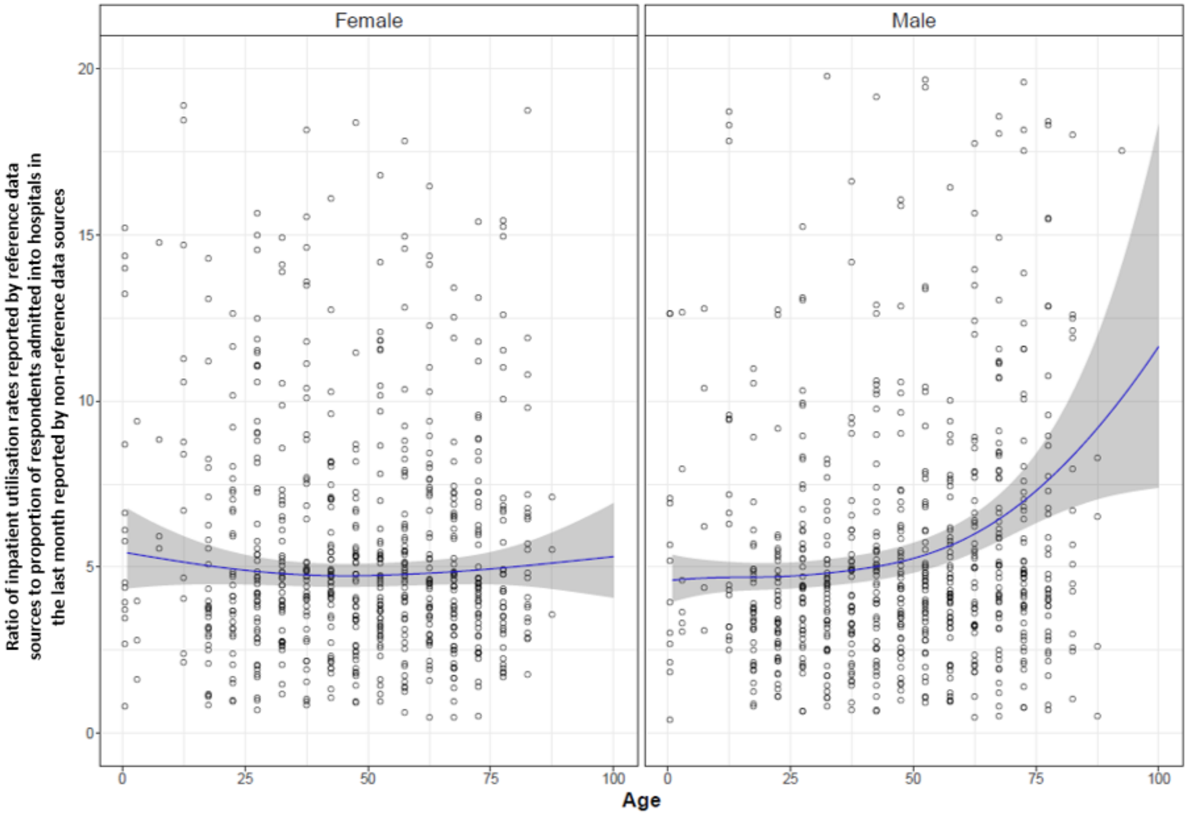

Female
Male
Ratio of inpatient utilisation rates reported by reference data sources to proportion of respondents admitted into hospitals in the last month reported by non-reference data sources
Age

To crosswalk non-reference data types to reference data types, we multiplied non-reference data types by the exponentiated predictions from respective penalised spline regressions. Uncertainty from the adjustments was accounted for by the equation

$$se_a = \sqrt{se_m^2 \cdot se_s^2 + se_m^2 \cdot \mu_s^2 + se_s^2 \cdot \mu_m^2} \quad (2)$$

Where:

se_a is the standard error of the adjusted non-reference data point

se_m is the standard error of the exponentiated crosswalk prediction

se_s is the standard error of the non-reference data point

μ_s is the mean of the non-reference data point

μ_m is the exponentiated crosswalk prediction from the penalised spline regression

Section 4.3.2: Age-sex splitting

Before modelling, we ran a Dismod-MR 2.1 model with data disaggregated by age to estimate countries' age-pattern and then applied the estimated age-pattern to split aggregated all-age data into the age groups that are necessary 5-year age groups encouraged by ST-GPR. This procedure was done by calculating a constant, k, which was the ratio of the aggregated all-age data point, $\mu_{all\ age}$, to the all-age estimated utilisation rate from the DisMod-MR 2.1 model, $\widehat{\mu_d}$

$$k = \frac{\mu_{all\ age}}{\widehat{\mu_d}} \quad (3)$$

The constant, k, was then multiplied by age-specific utilisation rates from the DisMod-MR 2.1 model. The uncertainty from the data and the age-pattern were propagated by following Equation 2. The split data were then incorporated into the final DisMod-MR 2.1 model.

Section 4.3.3: Spatiotemporal Gaussian process regression (ST-GPR) modelling[4]

The input data were modelled by using ST-GPR to allow for smoothing over age, time, and location in locations that were missing complete datasets.

The flowchart showing the analytic steps can be found elsewhere.[41] The approach is a stochastic modelling technique that is designed to detect signals amidst noisy data. It also serves as a powerful tool for interpolating non-linear trends.[42,43] Unlike classical linear models that assume that the trend underlying data follows a definitive functional form, GPR assumes that the specific trend of interest follows a Gaussian process, which is defined by a mean function $m(\cdot)$ and a covariance function $Cov(\cdot)$. For example, let $p_{c,a,s,t}$ be the prevalence, in normal, log, or logit space, observed in country c, for age group a, and sex s at time t:

$$(p_{c,a,s,t}) = g_{c,a,s}(t) + \epsilon_{c,a,s,t}$$

where

$$\epsilon_{c,a,s,t} \sim Normal(0, \sigma_p^2),$$

$$g_{c,a,s}(t) \sim GP\left(m_{c,a,s}(t), Cov\left(g_{c,a,s}(t)\right)\right).$$

The derivation of the mean and covariance functions, $m_{c,a,s}(t)$ and $Cov\left(g_{c,a,s}(t)\right)$, along with a more detailed description of the error variance (σ_p^2), is described below.

Section 4.3.3.1: Estimating mean functions

We estimated mean functions by using a two-step approach. To be more specific, $m_{c,a,s}(t)$ can be expressed, depending on the prevalence transformation, as:

$$log\left(p_{c,a,s}(t)\right) = X_{c,a,s}\beta + h(r_{c,a,s,t})$$

$$logit\left(p_{c,a,s}(t)\right) = X_{c,a,s}\beta + h(r_{c,a,s,t})$$

$$p_{c,a,s}(t) = X_{c,a,s}\beta + h(r_{c,a,s,t})$$

where $X\beta$ is the summation of the components of a hierarchical mixed-effects linear regression, including the intercept and the product of covariates with their corresponding fixed-effect coefficients. Some models were run as hierarchical mixed-effects linear regressions with random effects on the levels of the location hierarchy. For most mixed-effects models, random effects were only used in the fit, not in the prediction. The second part of the equation, $h(r_{c,a,s,t})$, is a smoothing function for the residuals, $r_{c,a,s,t}$, derived from the linear model.[44] Cause-specific methods details can be found in appendix sections 3.4 and 4.12.

Although the linear component captures general trends over time, much of the data variability may still not be adequately accounted for. To address this, we fit a locally weighted polynomial regression (locally estimated scatterplot smoothing, or LOESS) function $h(r_{c,a,s,t})$ to systematically estimate this residual variability by borrowing strength across time, age, and space patterns (the spatiotemporal component of ST-GPR).[45,46] The time adjustment parameter, defined by λ , aims to borrow strength from neighboring time points (ie, the prevalence in this year is highly correlated with prevalence in the previous year but less so further back in time). The age-adjustment parameter, defined by ω, borrows strength from data in neighboring age groups. The space-adjustment parameter, defined by ξ, aims to borrow strength across the hierarchy of geographical locations. The spatial and temporal weights are combined into a single space-time weight to allow the amount of spatial weight given to a particular point $r_{c,a,s,t}$ to fluctuate given the data availability at each time *t* and location-level *l* in the location hierarchy.

Let $w_{c,a,s,t}$ be the final weight assigned to observation $r_{c,a,s,t}$ with reference to a focal observation r_{c_0,a_0,s_0,t_0}. We first generated a temporal weight $t.w_{c,a,s,t}$ for smoothing over time, which was based on the scaled distance along the time dimension of the two observations[46]:

$$t.w_{c,a,s,t} = \frac{1}{e^{\lambda|t-t_0|}}$$

Next, we generated a spatial weight to smooth over geography. Specifically, we defined a geospatial relationship by categorizing data based on the GBD location hierarchy (table S3). *zeta* acts as a scalar on a given datapoint given its proximity to the target location:

$$t.w_{c,a,s,t} = \zeta^{|c-c_0|}$$

For example, estimating a country, would use the following weighting scheme:

- Country data: ζ^0 = 1
- Regional data not from the country being estimated: ζ^1
- Data from other regions in the same super region: ξ^2
- Global data from other super regions: ζ^3

Under the spatial weighting specification, typical values of ζ range from [0.001, 0.2], where ζ can be interpreted as the amount to downweight regional datapoints compared to country datapoints for a given estimating country. For example, for a given datapoint $r_{c,a,s,t}$ and ζ = 0.01, a datapoint not within country *c* but within the same region *r* as $r_{c,a,s,t}$ would be assigned $\frac{1}{100}$ the weight of a datapoint within the country.

The spatial and temporal weights were then multiplied and summed across each level of the location hierarchy and normalised for each time period *t* . This procedure allowed the space-time weight to implicitly take into account the amount of data available at the country vs. region vs. super-region level and attribute spatial weight accordingly.

Given a normalisation constant,

$$K_i = \sum_{c \epsilon C} s.w_{c,t} * t.w_{c.t} + \sum_{c \epsilon R} s.w_{c,t} * t.w_{c.t} + \sum_{c \epsilon SR} s.w_{c,t} * t.w_{c.t}$$

the final space-time weight would then equal

$$w'_{c,a,s,t} = \frac{s.w_{c,t} * t.w_{c,t}}{K_i}$$

Finally, we calculated the weight $w''_{c,a,s,t}$ to smooth over age, which is based on a distance along the age dimension of two observations. For a point between the age a of the observation $r_{c,a,s,t}$ and a focal observation r_{c_0,a_0,s_0,t_0}, the weight is defined as follows:

$$w''_{c,a,s,t} = \frac{1}{e^{\omega|a-a_0|}}$$

The final weights were then computed by simply multiplying the space-time weights and age weights and normalising so all weights for a given time period *t* sum to 1. A full derivation of weights for each category, assuming the location being estimated was a country, follows:

1) If the observation $r_{c,t}$ belongs to the same country c_0 of the focal observation r_{c_0,t_0}:

$$w_{c,a,s,t} = \frac{(w'_{c,a,s,t}w''_{c,a,s,t})}{\sum_{c=c_0}(w'_{c,a,s,t}w''_{c,a,s,t})} \qquad \forall c = c_0$$

2) If the observation $r_{c,t}$ belongs to a different country than the focal observation r_{c_0,t_0}, but both belong to the same region *R*:

$$w_{c,a,s,t} = \frac{(w'_{c,a,s,t}w''_{c,a,s,t})}{\sum_{c\neq c_0}(w'_{c,a,s,t}w''_{c,a,s,t})} \qquad \forall c \neq c_0 \cap R[c] = R[c_0]$$

3) If the observation $r_{c,t}$ belongs to the same super region *SR* but to both a different country c_0 and a different region $R[c_0]$ than the focal observation r_{c_0,t_0}:

$$w_{c,a,s,t} = \frac{(w'_{c,a,s,t}w''_{c,a,s,t})}{\sum_{c\neq c_0}(w'_{c,a,s,t}w''_{c,a,s,t})} \qquad \forall c \neq c_0 \cap R[c] \neq R[c_0] \cap SR[c] = SR[c_0]$$

4) If the observation $r_{c,t}$ is from a different super region than the focal observation r_{c_0,t_0}(Ie, all other data currently not receiving a weight):

$$w_{c,a,s,t} = \frac{(w'_{c,a,s,t}w''_{c,a,s,t})}{\sum_{c\neq c_0}(w'_{c,a,s,t}w''_{c,a,s,t})} \qquad \forall c \neq c_0 \cap R[c] \neq R[c_0] \cap SR[c] \neq SR[c_0]$$

Observations could be downweighted by a factor of 0.1, usually because they were not geographically representative at the unit of estimation. Details of reasons for downweighting can be found in cause-specific modeling summaries. The final weights were then normalised such that the sum of weights across age, time, and geographic hierarchy for a reference group was 1.

Section 4.3.3.2: Estimating error variance

σ_p^2 represents the error variance in normal or transformed space including the sampling variance of the estimates and prediction error from any crosswalks performed. First, variance was systematically imputed if the data extraction did not include any measure of uncertainty. When some sample sizes for data were available, missing sample sizes were imputed as the 5th percentile of available sample sizes. Missing variances were then calculated as $\sigma_p^2 = \frac{p*(1-p)}{n}$ for proportions or were predicted from the mean by using a regression for continuous values. When sample sizes were entirely missing and could not be imputed, the 95th percentile of available variances at the most granular geographic level (ie, first country, then region, etc.) were used to impute missing variances. For proportions where *p*n* or *(1-p)*n* is <20, variance was replaced by using the Wilson Interval Score method.

Next, if prevalence was modelled as a log transformation, the error variance was transformed into log-space by using the delta method approximation as follows:

$$\sigma_p^2 \cong \frac{\sigma_{p'}^2}{p_{c,a,s,t}^2}$$

where $\sigma_{p'}^2$ represents the error variance in normal space. If prevalence was modelled as a logit transformation, the error variance was transformed into logit-space by using the delta method approximation as follows:

$$\sigma_p^2 \cong \frac{\sigma_{p'}^2}{(p_{c,a,s,t} * (1 - p_{c,a,s,t}))^2}$$

Finally, prior to GPR, an approximation of non-sampling variance was added to the error variance. Calculations of non-sampling variance were done on normal-space variances. Non-sampling variance was calculated as the variance of inverse-variance weighted residuals from the space-time estimate at a given location-level hierarchy. If there were <10 data points at a given level of the location hierarchy, the non-sampling variance was replaced with that of the next highest geography level with >10 data points.

Section 4.3.3.3: Estimating the covariance function

The final input into GPR is the covariance function, which defines the shape and distribution of the trends. Here, we have chosen the Matern-Euclidian covariance function, which offers the flexibility to model a wide spectrum of trends with varying degrees of smoothness. The function is defined as follows:

$$M(t,t') = \sigma^2 \frac{2^{1-v}}{\Gamma(v)} \left(\frac{d(t,t')\sqrt{2v}}{l}\right)^v K_v \left(\frac{d(t,t')\sqrt{2v}}{l}\right)$$

where $d(\cdot)$is a distance function; σ^2, v, l, and K_v are hyperparameters of the covariance function—specifically σ^2 is the marginal variance, v is the smoothness parameter that defines the differentiability of the function, l is the length scale, which roughly defines the distance between which two points become uncorrelated, and K_v is the Bessel function. We approximated σ^2 by taking the normalised median absolute deviation $MADN(r_c')$ of the difference, which is the normalised absolute deviation of the difference of the first-stage linear regression estimate from the second-stage spatiotemporal smoothing step for each country. We then took the mean of these country-level MADN estimates for all countries with 10+ country-years of data to ensure that differences between first- and second-stage estimates had sufficient data to truly convey meaningful information on model uncertainty. We used the parameter specification $v = 2$ for all models. The scale parameter l used for each cause is reported in appendix sections 3.4 and 4.12.

Section 4.3.3.4: Prediction using GPR

We integrated over $g_{c,t}(t_*)$ to predict a full time series for country c, age a, sex s, and prediction time t_*as follows:

$$p_{c,a,s}(t_*) \sim N\left(m_{c,a,s,t}(t_*), \sigma_p^2 I + Cov\left(g_{c,a,s,t}(t_*)\right)\right)$$

Random draws of 1000 samples were obtained from the distributions above for every country for a given indicator. The final estimated mean for each country was the mean of the draws. In addition, 95% UIs were calculated by taking the 2.5 and 97.5 percentile of the sample distribution. The linear modelling process was implemented by using the lmer4 package in R, and the ST-GPR analysis was implemented through the PyMC2 package in Python.

Section 4.3.3.5: Subnational scaling and aggregation

To ensure internal consistency of the estimates between countries and their respective subnational locations, national estimates were either created by population-weighted aggregation or subnational estimates were adjusted by population-weighted scaling to the national estimates, depending on the data coverage of a given country compared to that of its subnational locations. For example, if data coverage was better at the national level than at its corresponding subnational locations for a given country and cause across age, sex, and time, estimates were rescaled to be consistent with the national level. Conversely, if data coverage was better at the subnational level, estimates for its parent country were generated through population-weighted aggregation of subnational estimates.

Estimates can also be scaled within logit space. Scaling in logit space ensures that subnational estimates of proportion models do not exceed one after being rescaled to the national estimate.

Section 4.3.3.6: Example: ST-GPR hospital bed estimation

To further help explain variation in geographies with little to no data, we used the covariates of the natural log of hospital beds per 1000 and the HAQ Index for every location. Hospital beds per 1000 was estimated by using ST-GPR on data sourced from the World Bank. Coefficients for the covariates are presented in the table that follows.

Table B. Estimated coefficients of the hospital envelope model.

**Covariate**	**Sex**	**Coefficient (95% UI)**	**Exponentiated Coefficient**
Log hospital beds per 1000	Male	0.41 (0.36 to 0.45)	1.50 (1.44 to 1.57)
	Female	0.41 (0.37 to 0.45)	1.50 (1.45 to 1.56)
HAQ Index	Male	0.029 (0.027 to 0.030)	1.029 (1.027 to 1.030)
	Female	0.028 (0.026 to 0.029)	1.028 (1.027 to 1.029)
All-cause mortality	Male	2.14 (2.11 to 2.17)	8.49 (8.25 to 8.73)
	Female	2.33 (2.30 to 2.36)	10.24 (9.93 to 10.55)

Section 4.3.4: Claims, inpatient hospital, and outpatient data

Claims, inpatient hospital, and outpatient data played a key role in the process of estimating many non-fatal causes in GBD 2019. All sources of administrative clinical data were aggregated and processed together for all causes of disease that included this type of data in their estimates. Data sources were heterogeneous in granularity, comprehensiveness, and level of detail, and the methods described below were used to transform data to be comparable and complete across locations, ages, sexes, and years, and causes.

Section 4.3.4.1 Claims data

For GBD 2019, we accessed aggregate data derived from the Truven database of USA private health insurance and subset of public insurance schemes of Medicaid and Medicare for the years 2000, 2010-2016. The population covered in each year was 3.3 million in 2000, 40.4 million in 2010, 44.4 million in 2011, 40.8 million in 2012, 42.2 million in 2013, 36.4 million in 2014, 22.6 million in 2015, and 22.4 million in 2016. For each of these individuals, information on every health service encounter was collected and all episodes of care were linked to individuals by unique identifiers. Outpatient claims could have up to four diagnoses while inpatient claims had up to 15 diagnoses. Data from Taiwan (province of China), the Philippines, Poland, Russia, and Singapore were also incorporated as claims data. We mapped ICD diagnoses in each source to GBD causes. GBD conditions were extracted as "prevalence" or "incidence" depending on cause duration and based on the specification of the research team responsible for the cause. In a given year, for each individual in the claims data, a prevalent case was defined as any mention in any diagnostic field associated with any claim, including inpatient and outpatient encounters. To reduce noise from spurious coding practices, an additional requirement is placed on prevalence in outpatient claims whereby a minimum of two claims must be filed in a calendar year to count as a prevalent case. An incident case was defined the same way but assumed that claims within a condition-specific duration were the same case. In this way, an individual could have multiple incident cases in a given year, but double-counting of cases with multiple claims from a single illness episode was avoided.

Figure C. GBD 2019 Claims Data Processing

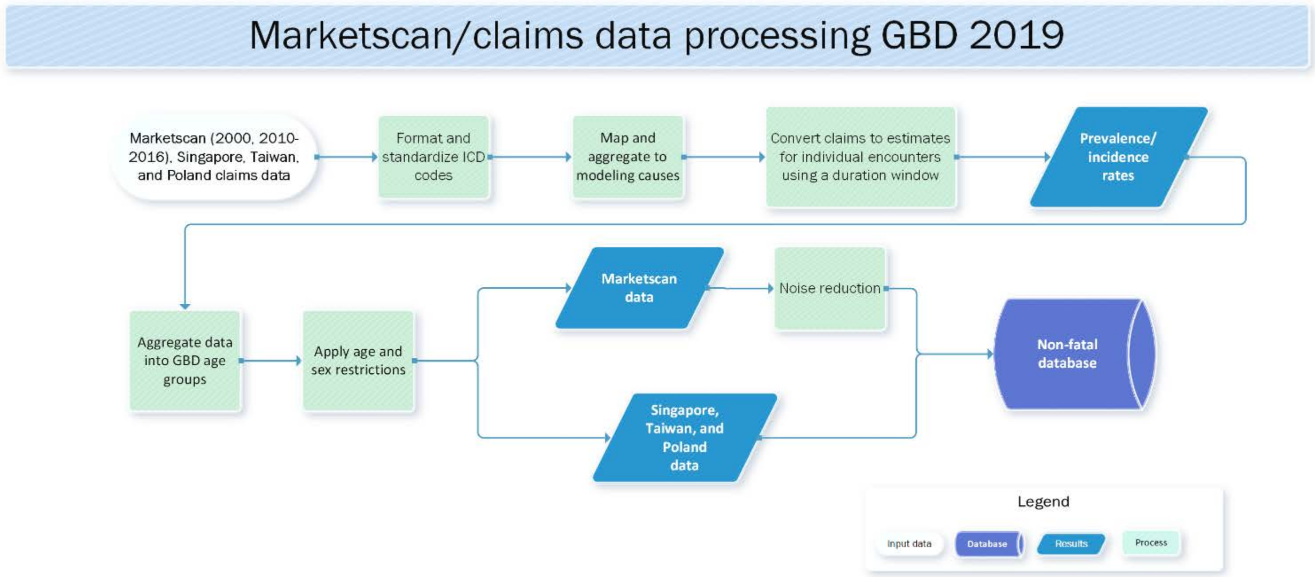

Section 4.3.4.2 Inpatient hospital admissions

Inpatient hospital data were extracted from 4401 location-years in 45 countries. ICD coding was standardised across sources and versions of ICD. Counts of admissions with a primary diagnosis of each cause were extracted from all sources and modelled through the inpatient hospital process. Secondary diagnostic detail was included in estimation through corrections as described below. A case of any cause of disease was defined as an overnight inpatient admission with a primary diagnosis of that cause.

For GBD 2015, our use of hospital data in non-fatal disease estimation was limited by the challenge of accessing accurate information on coverage populations for any given data source. Section 4.1.4 of the appendix describes the modelling strategy that was developed for the hospital utilisation envelope, an estimate of admission per capita in each location. In GBD 2016, we used the hospital utilisation envelope in place of information on coverage population. We calculated age-specific and sex-specific cause fractions in each inpatient hospital data source and multiplied these fractions by the hospital utilisation envelope to produce incidence or prevalence rates. In GBD 2017, we used the same approach except the hospital envelope was measured in ST-GPR to accommodate admissions data reflecting newborns being delivered in facilities. In GBD 2019, we updated the modelling framework to the hospital utilisation envelope, adding all-cause mortality as a covariate and improving the space-time smoothing to more accurately fit locations with and without data.

We performed three adjustments on inpatient hospital data to synthesise all inpatient sources to the same definition of care and to account for cases that were not captured in some inpatient sources depending on data availability. Data were first adjusted to account for multiple admissions for a single case of disease. It was then adjusted to account for cases of any cause that were non-primary reasons for admission. Finally, admissions were scaled by the ratio of outpatient cases observed for any inpatient case of disease to account for additional cases that did not warrant an inpatient admission. Combined with the uncorrected version (with no scalar applied), this process resulted in four stages of incidence and prevalence estimates from inpatient hospital data: (1) (un-corrected) inpatient admissions by episode, primary diagnosis; (2) inpatient admissions by individual, primary diagnosis only; (3) inpatient hospital admissions, accounting for all diagnoses; and (4) an estimate of inpatient admissions and outpatient visits by individual, accounting for all diagnoses. Estimate 4 was applied to all causes except those where outpatient care or non-primary diagnosis was not expected based on the nature of the disease. Adjustment ratios were calculated using all clinical inpatient sources that had patient-level data and primary and non-primary diagnoses. Sources of this data include Marketscan and Taiwan (province of China) claims data as described above; claims and inpatient data from Singapore, the Philippines, Ecuador, and New Zealand; and the HCUP SID database spanning years 2003–2008. Only Marketscan and Taiwan (province of China) claims data included a link between inpatient and outpatient care to be used in the fourth estimate described. Ratios from these sources were modelled over age and sex using a mixed-effects model in MR-BRT for each cause. If data for any ratio did not exist for the youngest or oldest age groups, we assumed a uniform tail on the model from the nearest age group with data. All models were conducted in log-space in order to bound the model to be greater than one for any age, sex, and cause. We used the following equations for each of the three scalars:

1) Correction to account for multiple admissions, which gives us inpatient admissions by individual, primary diagnosis only
    a. $$inpatient_{admin}^{1°} * \left(\frac{inpatient_{indiv}^{1°}}{inpatient_{admin}^{1°}}\right) = inpatient_{indiv}^{1°}$$
2) Correction to adjust for non-primary diagnoses, which gives us inpatient admissions by individual, all diagnoses
    a. $$inpatient_{admin}^{1°} * \left(\frac{inpatient_{indiv}^{all}}{inpatient_{admin}^{1°}}\right) = inpatient_{indiv}^{all}$$

3) Correction to account for inpatient and outpatient care, which gives us inpatient admissions and outpatient visits by individual for all diagnoses

   a. $inpatient_{admission}^{1°} * \left(\frac{inpatient_{indiv}^{all} \cup outpatient_{indiv}^{all}}{inpatient_{admissions}^{1°}}\right) = inpatient|outpatient_{indiv}^{all}$

Determination of maternal causes used separate cause-fractions and a different scalar calculated from a maternal hospital admissions rate instead of the hospital envelope, and the equation

$$\left(\frac{events}{\#\ of\ total\ hospital\ visits}\right) * \left(\frac{hospital\ visits}{live\ births}\right) * \left(\frac{births}{population}\right)$$

Determination of injuries used a separate correction factor from those described above which adjusted data that was only E-coded by data that contained E-codes and N-codes (nature of injury codes) with the following equation

$$\frac{1}{\frac{E\text{-}code\ primary\ dx}{E\text{-}code\ any\ dx + N\text{-}code\ any\ dx}}$$

A final adjustment was applied to each of the above estimates. The HAQ Index was used to account for differences in access and quality of health care across time and space. The HAQ Index adjustment was applied by dividing the above estimates by a scalar ranging from 0 to 100, where 0 represents the first percentile of observed access and quality and 100 the 99th percentile.

Figure D. GBD 2019 Inpatient Hospital Data Processing

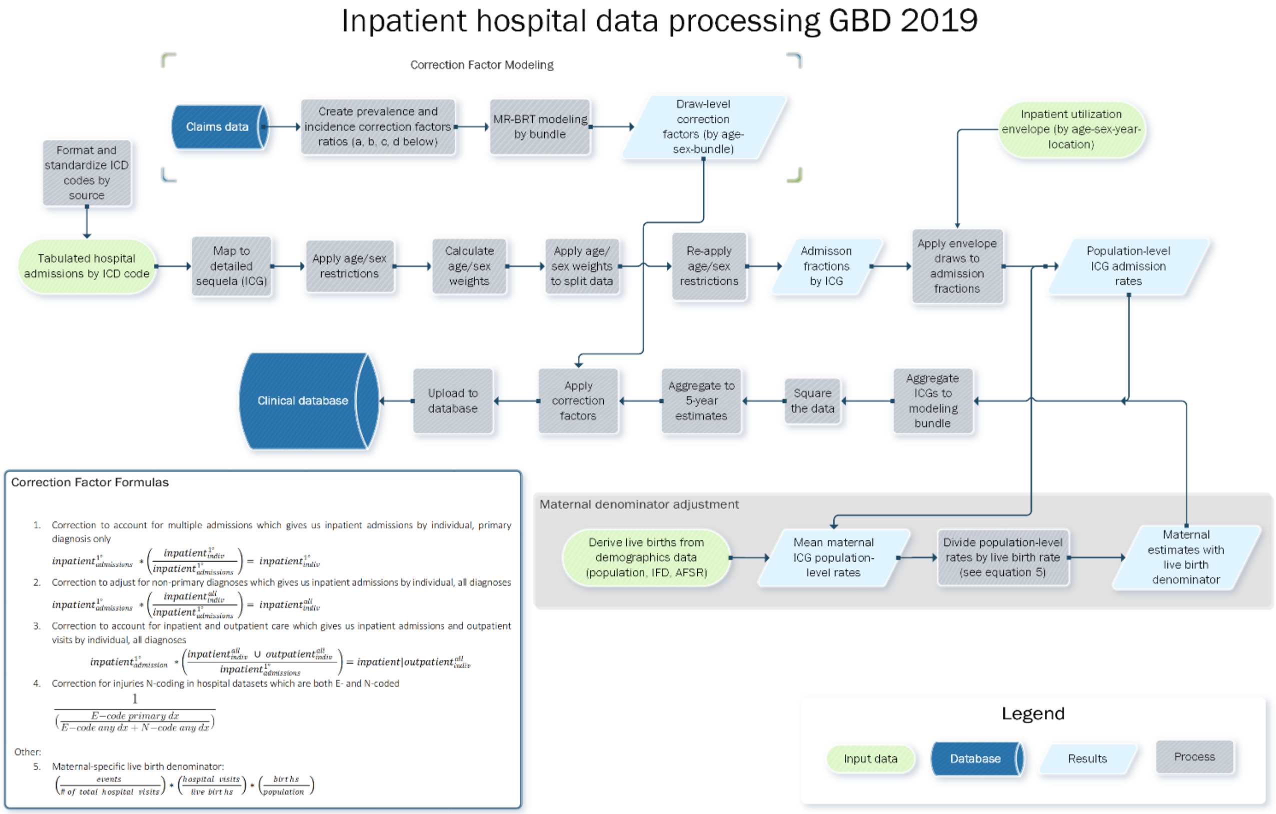

Section 4.3.4.3 Outpatient encounter data

Outpatient encounter data were available from the USA and Sweden for 109 location-years. No changes were made in the processing of outpatient data from GBD 2017, except for updates to the ICD mappings to GBD cause.

As with the inpatient hospital data, a scalar was calculated by using Marketscan claims data to adjust for multiple visits per individual within one year (for prevalent conditions) and within a cause-specific duration (for incident causes).

Figure E. GBD 2019 Outpatient data extraction process

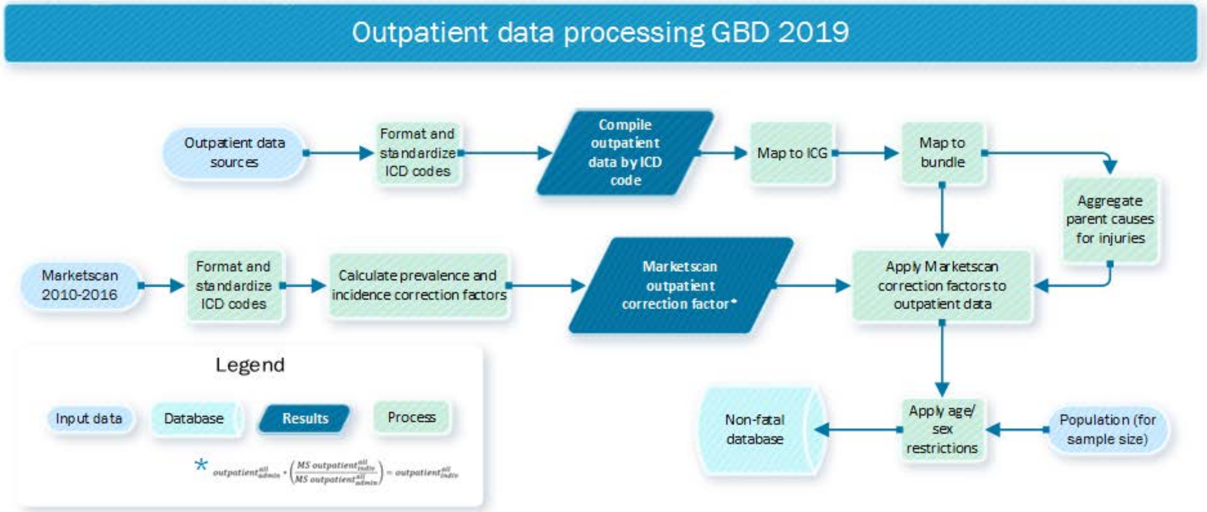

Section 4.3.5: Case notifications

Case notifications, active screening, intervention coverage studies, and surveillance contributed to estimates of infectious diseases. If data were available, we extracted it from survey and administrative microdata; otherwise, data were extracted from published literature and reports. For many infectious diseases and neglected tropical diseases (NTDs), we used of cases for which notification was made by countries to the WHO and other global monitoring entities. The causes for which we used WHO case notification data included tuberculosis, measles, yellow fever, rabies, dengue, cholera, whooping cough, human African trypanosomiasis (HAT), meningitis, all sexually transmitted infections, and other infectious diseases and NTDs, such as Ebola.

Section 4.4: Data adjustment

Section 4.4.1: MR-BRT and Fitting Procedures

This section details the statistical models underlying MR-BRT, and fitting procedure used to obtain estimates. Further details on models and algorithms can be found in the technical report.[47]

The MR-BRT program is a set of wrappers customized for global health problems that use the open source mixed effects package `LimeTr` (https://github.com/zhengp0/limetr). We describe the basic functionality in the sections below.

Section 4.4.1.1 Mixed-Effects Model

We consider the following nonlinear mixed effects model:

$$\boldsymbol{y}_i = \mathbf{F}_i(\beta) + \mathbf{Z}_i\boldsymbol{u}_i + \boldsymbol{\epsilon}_i$$

$$\boldsymbol{u}_i \sim N(\mathbf{0}, \mathbf{\Gamma}),\ \mathbf{\Gamma} = \mathrm{diag}(\gamma), \qquad \boldsymbol{\epsilon}_i \sim N(\mathbf{0}, \mathbf{\Lambda}), \tag{1}$$

where $y_i \in \mathbb{R}^{n_i}$ is the vector of observations from the ith study, $\boldsymbol{\epsilon}_i \in \mathbb{R}^{n_i}$ are measurement errors with given covariance $\mathbf{\Lambda}$, $\boldsymbol{u}_i \in \mathbb{R}^{k_\gamma}$ are independent random effects, and $\mathbf{Z}_i \in \mathbb{R}^{n_i \times k_\gamma}$is a linear map, and β are regression coefficients. The models F_i may be nonlinear.

To fit (β, γ) we solve the marginal likelihood problem:

$$\min_{\beta,\gamma} f(\beta,\gamma) := \sum_{i=1}^{m} \frac{1}{2}\left(y_i - \mathbf{F}_i(\beta)\right)^\top \left(\mathbf{Z}_i\mathbf{\Gamma}\mathbf{Z}_\mathrm{i}^\top + \boldsymbol{\Lambda_i}\right)^{-1}\left(\boldsymbol{y}_i - \mathbf{F}_i(\beta)\right) + \frac{1}{2} ln\left|\mathbf{Z}_i\mathbf{\Gamma}\mathbf{Z}_\mathrm{i}^\top + \mathbf{\Lambda}_i\right|. \tag{2}$$

When the model is linear, we can write:

$$\mathbf{F}_i(\beta) = \boldsymbol{X}\beta. \tag{3}$$

Linear models are very common in cross-walks, and for network analysis, which is detailed below.

Section 4.4.1.2. Network Analysis

Network analysis is a special case of the linear model (3) that is used to compare multiple treatment effects. To explain the coding we use a running example with four treatments A, B, C, D.

For simplicity assume A is this reference treatment. We then have the following coding.

$$AB \to B - A: \quad [1 \quad 0 \quad 0]$$

$$AC \to C - A: \quad [0 \quad 1 \quad 0]$$

$$AD \to D - A: \quad [0 \quad 0 \quad 1].$$

We see from this simple example that the design matrix under the basic network assumption is always full rank, since a subset of rows forms the identity matrix.

Comparisons that do not include the reference can be computed. For example,

$$BC \to C - B = (C - A) - (B - A)$$

$$= [0 \quad 1 \quad 0] - [1 \quad 0 \quad 0]$$

$$= [-1 \quad 1 \quad 0]$$

Using this simple algebra, we quickly obtain the remaining codings.

$$BC \to C - B: \quad [-1 \quad 1 \quad 0]$$

$$BD \to D - B: \quad [-1 \quad 0 \quad 1]$$

$$CD \to D - C: \quad [0 \quad -1 \quad 1]$$

Each row of the design matrix $\boldsymbol{X}$ is coded according to the comparison.

When doing network analysis, the design matrix $\boldsymbol{X}$ does not include the intercept term (**1** column).

Section 4.4.1.3. Constraints and Priors

The ML estimate (2) can be extended to incorporate nonlinear inequality constraints

$$\mathbf{C}(\boldsymbol{\theta}) \le c,$$

where $\boldsymbol{\theta} = (\beta, \gamma)$. Constraints play a key role for polynomial splines.

It is also essential to allow priors on parameters of interest. We assume that priors are given by a functional form

$$\boldsymbol{\theta} \sim \exp(-\rho(\boldsymbol{\theta}))$$

The likelihood problem is then augmented by adding the term $\rho(\boldsymbol{\theta})$ to the ML objective. The function ρ may be nonlinear and nonconvex, but we assume it is smooth.

Section 4.4.1.4. Trimming outliers

Least trimmed squares (LTS) is a robust estimator[48,49] for the standard regression problem. Given the problem

$$\min_{\beta} \sum_{i=1}^{n} \frac{1}{2} (y_i - \langle \boldsymbol{X}_i, \beta \rangle)^2, \tag{4}$$

the LTS estimator minimizes the sum of *smallest h* residuals rather than all residuals. These estimators were initially introduced to develop linear regression estimators that have a high breakdown point (in this case 50%) and good statistical efficiency (in this case $n^{-1/2}$). Breakdown refers to the percentage of outlying points which can be added to a dataset before the resulting M-estimator can change in an unbounded way. Here, outliers can affect both the outcomes and training data (features).

LTS estimators are robust against outliers, and arbitrarily large deviations that are trimmed do not affect the final $\hat{\beta}$.

Rather than writing the objective in terms of order statistics, it is far simpler to extend the likelihood using an auxiliary variable $\boldsymbol{W}$:

$$\min_{\beta, \mathbf{W}} \sum_{i=1}^{n} w_i \left(\frac{1}{2} (y_i - \langle \mathbf{X}_i, \beta \rangle)\right)^2 \quad \text{s.t.} \quad \mathbf{1}^\top \boldsymbol{W} = h, \quad \mathbf{0} \le \boldsymbol{W} \le \mathbf{1}. \tag{5}$$

The set

$$\Delta_h := \{\mathbf{W} : \mathbf{1}^\top \mathbf{W} = h, \quad \mathbf{0} \le \mathbf{W} \le \mathbf{1}\} \tag{6}$$

is known as the *capped simplex*, since it is the intersection of the h-simplex with the unit box.[48] For a fixed β, the optimal solution of (5) with respect to $\mathbf{W}$ assigns weight 1 to each of the smallest h residuals, and 0 to the rest. Problem (5) is solved *jointly* in $(\beta, \mathbf{W})$, simultaneously finding the regression

estimate and classifying the observations into inliers and outliers. This joint strategy makes LTS different from post-hoc analysis, where a model is fit first with all data, and then outliers are detected using that estimate.

To explain how trimming enters the marginal likelihood problem, we focus on a single group term from the ML likelihood (2):

$$\left(\frac{1}{2}\left(\boldsymbol{y}_i - \mathbf{F}_{i(\beta)}\right)^\top \left(\mathbf{Z}_i\boldsymbol{\Gamma}^{-1}\mathbf{Z}_i^\top + \boldsymbol{\Lambda}_i\right)^{-1}\left(\boldsymbol{y}_i - \mathbf{F}_i(\beta)\right) + \frac{1}{2}\ln\left|\mathbf{Z}_i\boldsymbol{\Gamma}^{-1}\mathbf{Z}_i^\top + \boldsymbol{\Lambda}_i\right|\right)$$

We introduce auxiliary variables $\mathbf{W}_i \in \mathbb{R}^{n_i}$, and define

$$\boldsymbol{r}_i := y_i - \mathbf{F}_i(\beta), \quad \mathbf{W}_i := \mathrm{diag}(\mathbf{W}_i), \quad \sqrt{\mathbf{W}_i} := \mathrm{diag}(\sqrt{\mathbf{W}_i}).$$

We now form the objective

$$\tfrac{1}{2}\boldsymbol{r}_I^\top \sqrt{\mathbf{W}_i}\left(\sqrt{\mathbf{W}_i}\mathbf{Z}_i\boldsymbol{\Gamma}^{-1}\mathbf{Z}_i^\top\sqrt{\mathbf{W}_i} + \boldsymbol{\Lambda}_i^{\odot\mathbf{W}_i}\right)^{-1} \sqrt{\mathbf{W}_i}\,\boldsymbol{r}_i + \tfrac{1}{2}\ln\left|\sqrt{\mathbf{W}_i}\,\mathbf{Z}_i\boldsymbol{\Gamma}^{-1}\mathbf{Z}_i^\top\sqrt{\mathbf{W}_i} + \boldsymbol{\Lambda}_i^{\odot\mathbf{W}_i}\right|, \quad (7)$$

where $\odot$ denotes the elementwise power operation:

$$\boldsymbol{\Lambda}_i^{\odot\mathbf{W}_i} := \begin{bmatrix} (\lambda_{1j})^{w_{i1}} & 0 & \cdots & 0 \\ 0 & \ddots & \ddots & \vdots \\ 0 & \cdots & 0 & (\lambda_{in_i})^{w_{in_i}} \end{bmatrix} \quad (8)$$

When $w_{ij} = 1$, we recover the contribution of the ijth observation to the original likelihood. As $w_{ij} \downarrow 0$, the ijth contribution to the residual is correctly eliminated by $\sqrt{w_{ij}} \downarrow 0$. The jth row and column of $\sqrt{\mathbf{W}_i}\mathbf{Z}_i\boldsymbol{\Gamma}^{-1}\mathbf{Z}_i^\top\sqrt{\mathbf{W}_i}$ both go to 0, while the jth entry of $\boldsymbol{\Lambda}_i^{\odot\mathbf{W}_i}$ goes to 1, which effectively removes all impact of the jth point on the covariance matrix.

For full details and analysis, please see the technical report.[47]

Section 4.4.1.5. Final Estimator

Putting together the trimmed ML with priors and constraints, we arrive at the following estimator.

$$\begin{aligned} \min_{\beta,\gamma,\mathbf{W}} f(\beta,\gamma,\mathbf{W}) := \sum_{i=1}^{m} \frac{1}{2}\, r_i^\top \sqrt{\mathbf{W}_i}\left(\sqrt{\mathbf{W}_i}\,\mathbf{Z}_i\boldsymbol{\Gamma}^{-1}\mathbf{Z}_i^\top\sqrt{\mathbf{W}_i} + \boldsymbol{\Lambda}_i^{\odot\mathbf{W}_i}\right)^{-1} \\ \sqrt{\mathbf{W}_i}\, r_i + \tfrac{1}{2}\ln\left|\sqrt{\mathbf{W}_i}\,\mathbf{Z}_i\boldsymbol{\Gamma}^{-1}\mathbf{Z}_i^\top\sqrt{\mathbf{W}_i} + \boldsymbol{\Lambda}_i^{\odot\mathbf{W}_i}\right| + \rho(\beta,\gamma,\boldsymbol{\Lambda}) \\ \text{s.t.}\ \ \boldsymbol{r}_i = \boldsymbol{y}_i - \mathbf{F}_i(\beta), \quad \mathbf{1}^\top\mathbf{W} = h, \quad 0 \le \mathbf{W} \le 1, \quad \boldsymbol{C}\begin{pmatrix}\beta\\ \gamma\end{pmatrix} \le c\,. \end{aligned} \quad (9)$$

The fit is obtained using iterative optimization techniques. Problem (9) is nonlinear and non-smooth, and the optimization is implemented in the `LimeTR` package[3] (https://github.com/zhengp0), and relies on the IPopt interior point method.[50]

Section 4.4.1.6. Nonlinear Dose-Response Curves with Constrained Splines

In this section we discuss spline models for dose-response relationships. General background on splines and spline regression are available elsewhere.[51,52]

Section 4.4.1.6.1. B-splines and bases

A spline basis is a set of piecewise polynomial functions with designated degree and domain. If we denote polynomial order by p, and the number of knots by k, we need $p + k$ basis elements s_j^p, which can be generated recursively as illustrated in Figure A.

Figure A. Recursive generation of b-spline basis elements (orders 0, 1, 2)

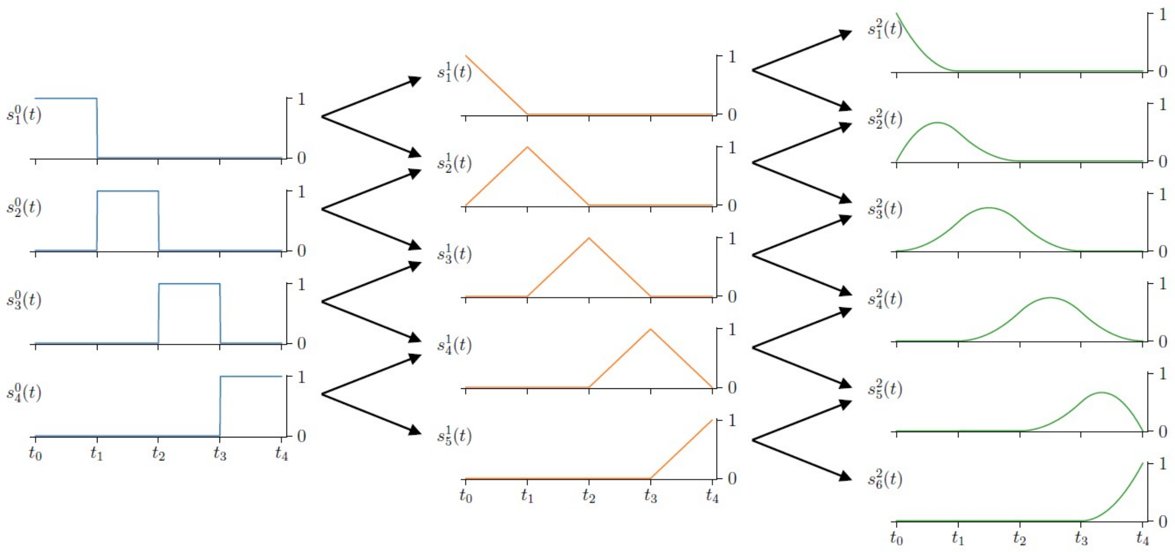

.

Given such a basis, we can represent any dose-response relationship as the linear combination of the spline basis elements, with coefficients $\beta \in \mathbb{R}^{\mathrm{p+k}}$:

$$f(t) = \sum_{j=1}^{p+k} \beta_j^p s_j^p(t). \tag{10}$$

These coefficients are then inferred as part of the general estimator (9) as discussed in the previous section. An explicit representation of (11) is obtained by building a design matrix $\mathbf{X}$. Given a set of t values at which we have data, the jth column of $\mathbf{X}$ is given by the expression

$$\mathbf{X}_{\cdot,j} = \begin{bmatrix} s_j^p(t_0) \\ \vdots \\ s_j^p(t_k) \end{bmatrix}. \tag{11}$$

The model for direct observations data coming from (11) can now be written compactly as

$$\boldsymbol{y} = \mathbf{X}\beta + \mathbf{Z}_i \boldsymbol{u}_i + \ \boldsymbol{\epsilon}_i \ ,$$

which is a special case of the main problem class (1).

Section 4.4.1.6.2. Shape constraints

We can impose shape constraints such as monotonicity, concavity, and convexity on splines. Constraints on splines have been developed in the past through reformulation techniques.[53] The development in this section uses explicit constraints instead.

Monotonicity. Spline monotonicity across the domain of interest follows from monotonicity of the spline coefficients.[51] Given coefficients

$$\beta = \begin{bmatrix} \beta_1 \\ \vdots \\ \beta_n \end{bmatrix},$$

the curve $f(t)$ in (11) is monotonically non-decreasing when

$$\alpha_1 \leq \alpha_2 \leq \cdots \leq \alpha_n$$

and monotonically non-increasing if

$$\alpha_1 \geq \alpha_2 \geq \cdots \geq \alpha_n.$$

The relationship $\alpha_1 \leq \alpha_2$ can be written as $\alpha_1 - \alpha_2 \leq 0$. Stacking these inequality constraints for each pair (α_i, α_{i+1}) we can write all constraints simultaneously as

$$\underbrace{\begin{bmatrix} 1 & -1 & 0 & \cdots & 0 \\ 0 & 1 & -1 & \cdots & 0 \\ \ddots & \ddots & \ddots & \ddots & \vdots \\ 0 & \cdots & \cdots & 1 & -1 \end{bmatrix}}_{\mathbf{C}} \begin{bmatrix} \alpha_1 \\ \alpha_2 \\ \alpha_3 \\ \vdots \\ \alpha_n \end{bmatrix} \leq \begin{bmatrix} 0 \\ 0 \\ \vdots \\ 0 \end{bmatrix}.$$

These linear constraints are a special case of the general estimator (9) that allows $\mathbf{C}(\beta) \leq c_\beta$.

Convexity and Concavity. For any twice continuously differentiable function: $f : \mathbb{R} \to \mathbb{R}$, convexity and concavity are captured by the signs of the second derivative. Specifically, f is convex if $f''(t) \geq 0$ is everywhere, and concave if $f''(t) \leq 0$ everywhere. We can compute $f''(t)$ for each interval, and impose linear inequality constraints on these expressions.

Enforcing linear tails. For large consumption with little data, we need the capability to ensure that the last segment of the spline is linear, with slopes that match the adjacent segment at the knot. The estimated spline is then a best fit to the data, subject to this specification. Priors on the tails can also be provided.

Figure B. Spline extrapolation. Left: linear extrapolation. Right: nonlinear extrapolation.

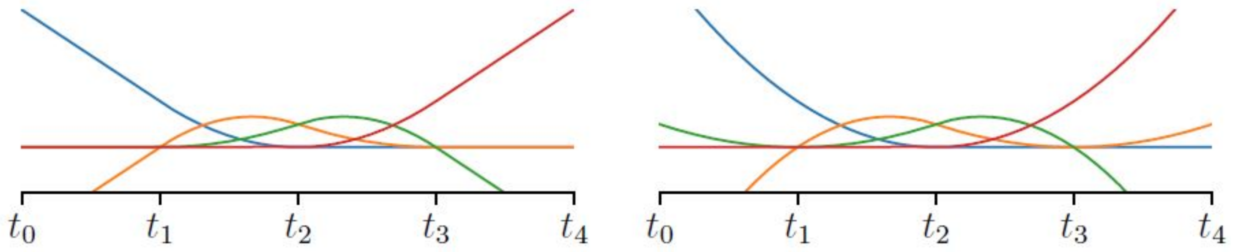

In general, using linear head and/or tail pieces to extrapolate outside the original domain or interpolate in the data sparse region is far more stable that using higher order polynomials, see figure B. The figure shows symmetric linear tail modifications, but for the analyses in the paper we only impose a right linear tail shape constraint.

Section 4.4.1.6.3. Posterior Variance Estimation

To obtain posterior uncertainty, we use a parametric bootstrap.[54] Once we solve (9) to obtain estimates $\hat{\beta}$ and $\hat{\gamma}$, we have a model distribution of the errors (1):

$$\boldsymbol{y}_i = \mathbf{F}_i(\hat{\beta}) + \mathbf{Z}_i \boldsymbol{u}_i + \boldsymbol{\epsilon}_i$$

We sample datasets from this distribution to generate full data sets $\{\mathbf{Y}\}^j$, for $j = 1, \ldots, N$. For each dataset $\mathbf{Y}^j$, we then re-solve the fitting problem (9) to obtain estimates $\hat{\beta}^j$ and $\hat{\gamma}^j$, and the set $\{\hat{\beta}^j, \hat{\gamma}^j\}$ over all j allows us to estimate any posterior statistic we need.

In particular, the posterior set of dose-response curves is given by

$$\{f(t)^j + u_0^j\}$$

where $f(t)^j$ is the curve obtained by using the re-fit value $\hat{\beta}^j$, and u_0^j is a sample from $N(0, \hat{\gamma}_0^j)$, the associated unexplained heterogeneity parameter.

Section 4.4.2: Bias adjustment for alternative case definitions and study methods

In GBD 2019, we decided to do all our adjustments of non-fatal and risk exposure data to deal with alternative case definitions or study methods prior to entering data into our main analytical tools of DisMod-MR 2.1 and ST-GPR. This decision also included the adjustment of data presented for both sexes to a male and female equivalent. The starting point was to explicitly state the reference case definition and study method and identify alternative definitions and study characteristics that fall within our inclusion criteria.

We compiled data from both within-study comparisons (ie, data that used alternative and reference definitions in the same population) and between-study comparisons (ie, data that used an alternative definition in one population and a reference definition in another population that overlap in location, time, age, and sex) of different case definitions. For between-study comparisons, we allowed a maximum calendar year difference between studies of five years. Where validation studies (ie, those carried out at the introduction of a new set of diagnostic criteria comparing to previous criteria) were available, we extracted data on the comparison of alternative to reference. For quantities of interest with multiple alternative definitions/methods we also look for pairs comparing two alternatives. In a network analysis, if A is the reference and B and C are two alternatives, a comparison of A vs B and B vs C provides an indirect comparison of the alternative C against the reference A.

We pooled either the logit difference between alternative and reference or the natural log of the ratio of alternative to reference. From simulations we found that the two methods provide almost identical results for quantities that after adjustment do not exceed a value of 0.5 (eg, prevalence or proportion). The logit difference method much better dealt with higher values and avoided prevalence or proportions to exceed one. If the values of either the reference or alternative were zero, we aggregated

values across age groups until both values had non-zero observations. We used the delta method to compute the standard error of the reference and alternative measures in logit space. The standard error of the logit difference was computed as the square root of the sum of the variances of each data point in a pair.

Section 4.4.2.1 Age-sex splitting

Age-sex splitting was commonly applied to literature data reported by age or sex but not by age and sex. For GBD 2019, we split all data reported in age groups with a width greater than 20 years, and we did so by using age patterns from available survey microdata or regional patterns derived from an initial run of the main modelling tool, DisMod-MR 2.1.

Section 4.4.2.2 Data analysis

We used a network random effects meta-regression in meta-regression—Bayesian, regularised, trimmed (MR-BRT). In a network analysis, if A is the reference and B and C are two alternatives, a comparison of A vs B and B vs C provides an indirect comparison of the alternative C against the reference A. To implement the network we included dummy variables with a particular structure. This was implemented as follows, where A is the reference definition/method:

- Create *k* dummy variables where *k* are all definitions/methods other than A (eg, *k* = B, C)
- Code dummy *k* as
  - 1 if the first term of the logit difference is *k*;
  - -1 if *k* is second term of the logit difference;
  - 0 otherwise

For example:

Study	Comparison	DummyB	DummyC
1	logit(B)-logit(A)	1	0
2	logit(B)-logit(A)	1	0
3	logit(C)-logit(A)	0	1
4	logit(C)-logit(A)	0	1
5	logit(C)-logit(B)	-1	1
6	logit(C)-logit(B)	-1	1

The coding structure outlined above in step 1 assumes that all case definitions are mutually exclusive. In some cases, however, individual case definitions are a function of different components

or dimensions. For example, case definitions may vary by the type of symptoms that a respondent experiences as well as the recall period over which those symptoms are experienced. In the presence of sparse data, it may be difficult to find both direct and indirect comparisons of all individual case definitions. In these case, an alternative approach is to assume different dimensions of case definitions have a multiplicative effect. In other words, the effect of recall period has the same relative effect across different categories of symptoms reported by respondents. To implement this coding scheme:

- Create *k* dummy variable columns for each case definition dimension
- For each dummy variable k:
    - Add 1 if *k* is a component of the first term in the logit difference
    - Subtract 1 if *k* is a component of the second term in the logit difference

In MR-BRT, we ran random effects meta-regression of the logit difference (or log ratio) with all the *k* dummy variables as covariates, omitting the intercept in the meta-regression. We used a study_id variable for the unique identifier of the reference and alternative studies (or alternative1 to alternative2). The coefficients on the *k* dummy variables represent the pooled logit difference of the *k* alternative definition to the reference taking into account evidence from both direct and indirect comparisons. In the example above, the coefficient on DummyA is the pooled logit difference of B minus A; the coefficient on DummyB is the pooled logit difference of C minus A. The standard error of the pooled logit difference incorporating the between study variance was calculated as:

$$se(\text{logit}(difference_k)) = \sqrt{var_k + \gamma^2}$$

Where:

$se(\text{logit}(difference_k)$ is the standard error of the pooled logit difference of alternative *k* to the reference

var_k is the variance of the coefficient on dummy variable *k*

γ^2 is the between-study variance

If both between and within study pairs were available, we examined whether there was a systematic difference between these. If there was a significant difference, we made judgement call as to whether within-study or between study data comparisons were most appropriate. In general, this was the within-study data, however, there were important measurement or conceptual reasons for choosing between-study data. For example, for crosswalks between self-reported height and weight compared to measured height and weight, between-study comparisons may be preferable if respondents knew they would be measured and, therefore, were less likely to misreport their height and weight.

We also examined whether there were systematic differences in the adjustments by key demographics (age, sex, geographic location, year) and other potential factors that may lead to variation in crosswalks. This could only be done at present in a direct comparison model and not in a network. We did this when there was a strong rationale, eg, biological plausibility, for variation by such characteristics.

After obtaining the pooled logit difference or log ratio estimates, we predicted adjustments based on the statistical model, including uncertainty in the adjustment and sampling error of each data point. For non-significant logit differences or log ratios we still applied the adjustments if there was a conceptual reason to believe that the alternative definition is biased. This expands the variance of these alternative definition data points.

Interpreting the coefficients of a logit difference model is not so straightforward as the adjustment to alternative data points is dependent of its value. For instance, the figure below on the left, shows the MR-BRT fit using a spline function by age to the logit differences of all overlapping pairs. The graph on the right indicates the adjustment by age for a hypothetical data point of 5%. The larger logit difference at younger ages, and to a lesser extent older ages, leads to a greater downward (in this case) adjustment of the 5% data point than at the mid age range.

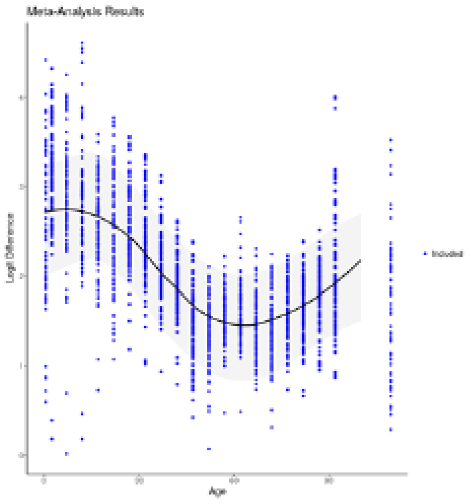

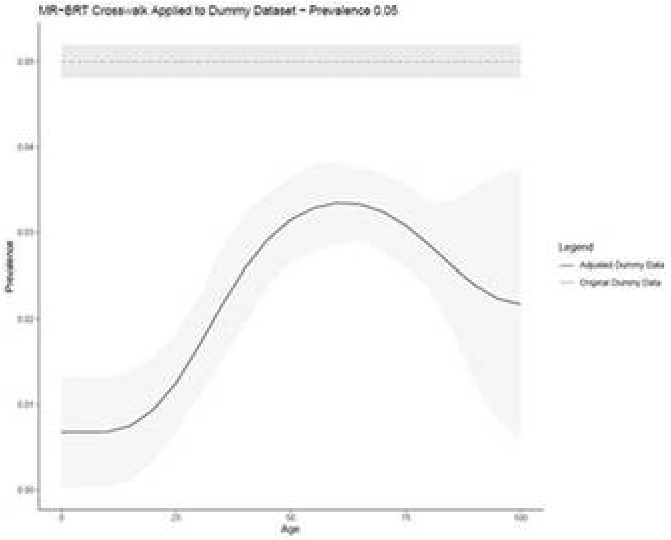

Section 4.5: DisMod-MR 2.1 estimation[2]

Section 4.5.1: Estimation of sequelae and causes

The most extensively used estimation method is the Bayesian meta-regression method DisMod-MR 2.1. For some causes such as HIV/AIDS or measles, disease-specific natural history models have been used for which the underlying three state model in DisMod-MR 2.1 (susceptible, cases, dead) is insufficient to capture the complexity of a disease process. For some diseases with a range of sequelae differentiated by severity, such as COPD or diabetes mellitus, DisMod-MR 2.1 was used to meta-analyse the data on overall prevalence with separate DisMod-MR 2.1 models of the proportions of cases with different severity levels or sequelae. Likewise, DisMod-MR 2.1 was used to meta-analyse data on the proportions of liver cancer and cirrhosis due to underlying aetiologies such as hepatitis B, hepatitis C, and alcohol use.

Section 4.5.2: DisMod-MR 2.1 description

Until GBD 2010, non-fatal estimates in burden of disease assessments were based on a single data source on prevalence, incidence, remission, or a mortality risk selected by the researcher as most relevant to a particular location and time. For GBD 2010, we set a more ambitious goal: to evaluate all

available information on a disease that passes a minimum quality standard. That required a different analytical tool that would be able to pool disparate information presented for varying age groupings and from data sources by using different methods. The DisMod-MR 1.0 tool used in GBD 2010 evaluated and pooled all available data, adjusted data for systematic bias associated with methods that varied from the reference, and produced estimates by world regions with UIs by using Bayesian statistical methods. For GBD 2013, the improved DisMod-MR 2.0 increased computational speed, which allowed computations to be consistent between all disease parameters at the country rather than the region level. The hundred-fold increase in speed of DisMod-MR 2.0 was partly due to a more efficient rewrite of the code in C++ but also to changing to a model specification by using log rates rather than a negative binomial model used in DisMod-MR 1.0. In cross-validation tests, the log rates specification worked as well or better than the negative binomial specification.[39] The sequence of estimation occurs at five levels: global, super-region, region, country and, where applicable, subnational location. The super-region priors are generated at the global level with mixed-effects, nonlinear regression by using all available data; the super-region fit, in turn, informs the region fit, and so on down the cascade. The wrapper gives analysts the choice to branch the cascade in terms of time and sex at different levels depending on data density. The default used in most models is to branch by sex after the global fit but to retain all years of data until the lowest level in the cascade is reached.

The computational engine is limited to three levels of random effects; we differentiate estimates at the super-region, region and country level. In GBD 2013, the subnational units of China, the UK and Mexico were treated as “countries” to enable a random effect to be estimated for every location with contributing data. However, the lack of a hierarchy between country and subnational units meant that the fit to country data contributed as much to the estimation of a subnational unit as the fits for all other countries in the region. We found inconsistency between the country fit and the aggregation of subnational estimates when the country’s epidemiology varied from the average of the region. Adding an additional level of random effects required a prohibitively comprehensive rewrite of the underlying DisMod-MR engine. Instead, we added a fifth layer to the cascade, with subnational estimation informed by the country fit and country covariates, plus an adjustment based on the average of the residuals between the subnational location’s available data and its prior. This technique mimicked the impact of a random effect on estimates between subnationals.

In GBD 2015, we also improved how country covariates differentiate non-fatal estimates for diseases with sparse data. The coefficients for country covariates are re-estimated at each level of the cascade. For a given location, country coefficients are calculated by using both data and prior information available for that location. In the absence of data, the coefficient of its parent location is used to utilise the predictive power of our covariates in data-sparse situations.

For GBD 2016, the computational engine (DisMod-MR 2.1) remained substantively unchanged from GBD 2015. We changed the prediction year set to generate fits for the years 1990, 1995, 2000, 2005, 2010, and 2016. We updated the age prediction sets to include age groups 80–84 years, 85–89 years, 90–94 years, and 95 years and older to comply with changes across all functional areas of the GBD. We also expanded the set of locations where subnational units are modelled; the set now includes Brazil, China, England, India, Indonesia, Japan, Kenya, Mexico, South Africa, Sweden, and the US.

In GBD 2017, we continued to use DisMod-MR 2.1 because no substantial changes were made. Updates to computation include extending the terminal prediction year to 2017 and additional subnational units in Ethiopia, Iran, New Zealand, Norway, and Russia. Saudi Arabia was also modelled only at the national level in 2017.

In GBD 2019, no substantial changes were made to DisMod-MR 2.1 but we made more substantial changes to how we use the tool. First, we added the year 2019 as an additional year of estimation. Second, we also included the option again to have random effects on cause-specific mortality rates (CSMR) and EMR This functionality had been dropped a couple of GBD rounds earlier. Third, as we did all our adjustments for alternative case definition and study methods as well as adjustments to both sex data points prior to entering data into DisMod-MR 2.1, we no longer used the functionality in DisMod-MR 2.1 to estimate coefficients for study covariates.

Fourth, based on simulation testing we found that coverage improved and errors reduced when passing down priors with a wider setting of minimum coefficient of variation (which determines the uncertainty around priors and hence how 'informative' the priors are) than had generally been used in past GBD iterations. We settled on a default value of 0.8 where in the past values of 0.4 or less had been more commonly used. We made some exceptions for high prevalent conditions where a lower minimum coefficient of variation (CV) setting achieved the task of making priors less informative but not completely uninformative.

We carried out simulation testing using DisMod-MR 2.1 based on an internally consistent set of 15,601 data points for prevalence, incidence, excess mortality, CSMR, and remission. The dataset was generated by the simulation capability of the DisMod-AT tool that is under development. We aimed to test what level of minimum CV would create the best fit based on the following three performance statistics:

(1) Coverage, ie, the proportion of data point mean values that fall between the 2.5th and 97.5th percentile of the draws of the fit values;
(2) Root mean square error: the square root of the mean of the squares of the difference between data point mean values and the mean fit value; and
(3) Bias: the difference between the mean fit value and the data point mean value.

We created different datasets culling the initial complete set with values at every age, sex, and location to more realistic data sparsity scenarios for analysis.

A first strategy was to randomly reduce the dataset to 10%, 5%, 2.5%, 1%, and 0.5% of the original data points. Initial results indicated little variation between the data samples culled to 10%, 5%, 2.5%, and 1%. The 0.5% culled dataset was an exception with markedly worse performance statistics, particularly with regard to bias and RMSE as illustrated in figure 1. We conducted further studies using the datasets culled to 10%, 5%, and 0.5%.

Figure 1. Performance statistics for randomly culled datasets

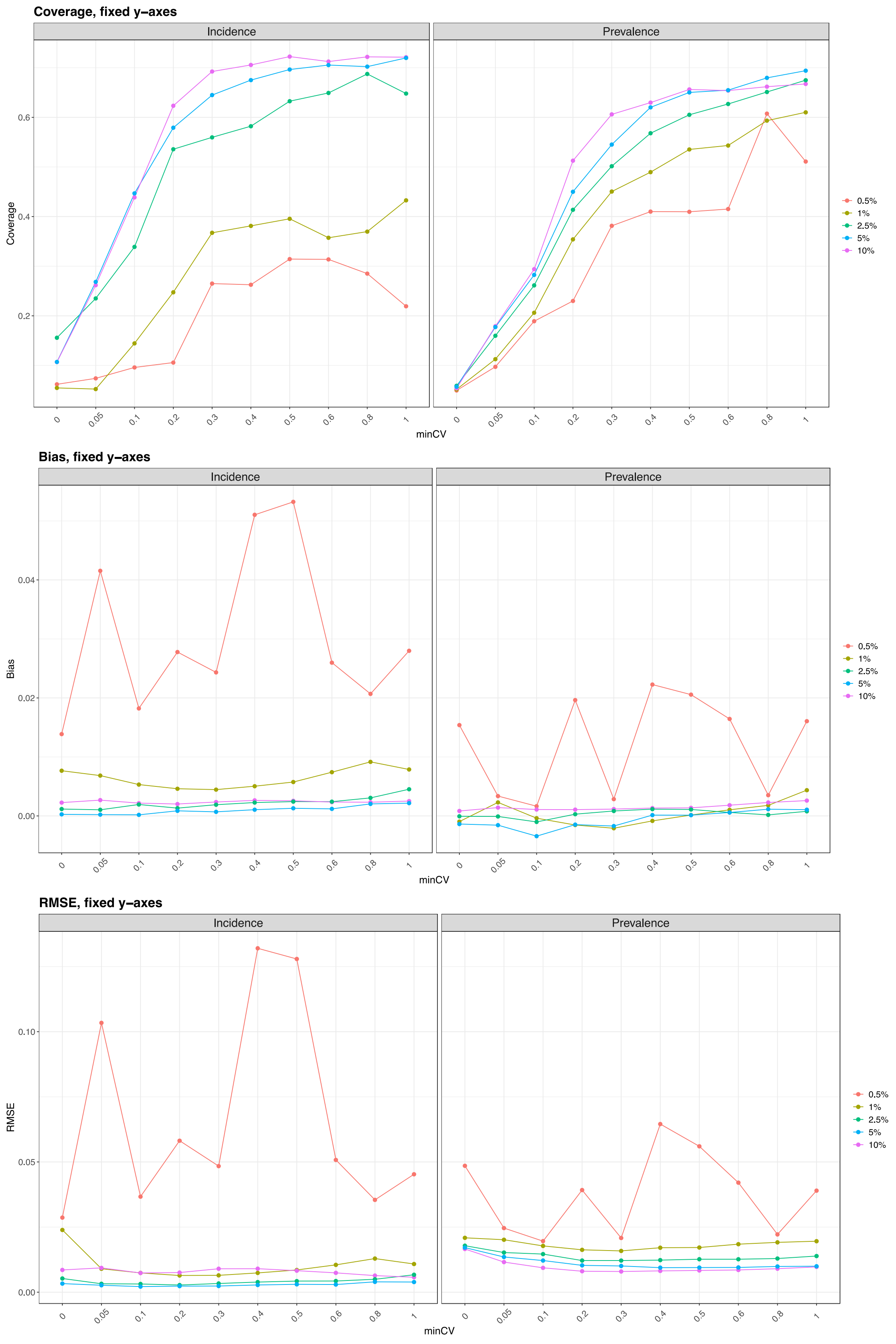

The second strategy was to compare randomly culled dataset for 10%, 5%, and 0.5% with datasets culled to the same percentages, but differentially by SDI, such that we culled all the data in sub-Saharan Africa and for the other super-regions based on the probability diminishing with increasing SDI. This pattern of differential data coverage by SDI is commonly observed in datasets used for modelling. The plots shown in figure 2, generally also show diminished performance for this more realistic scenario of differential sparseness by location based on SDI.

Figure 2. Performance statistics comparing randomly and differentially culled datasets.

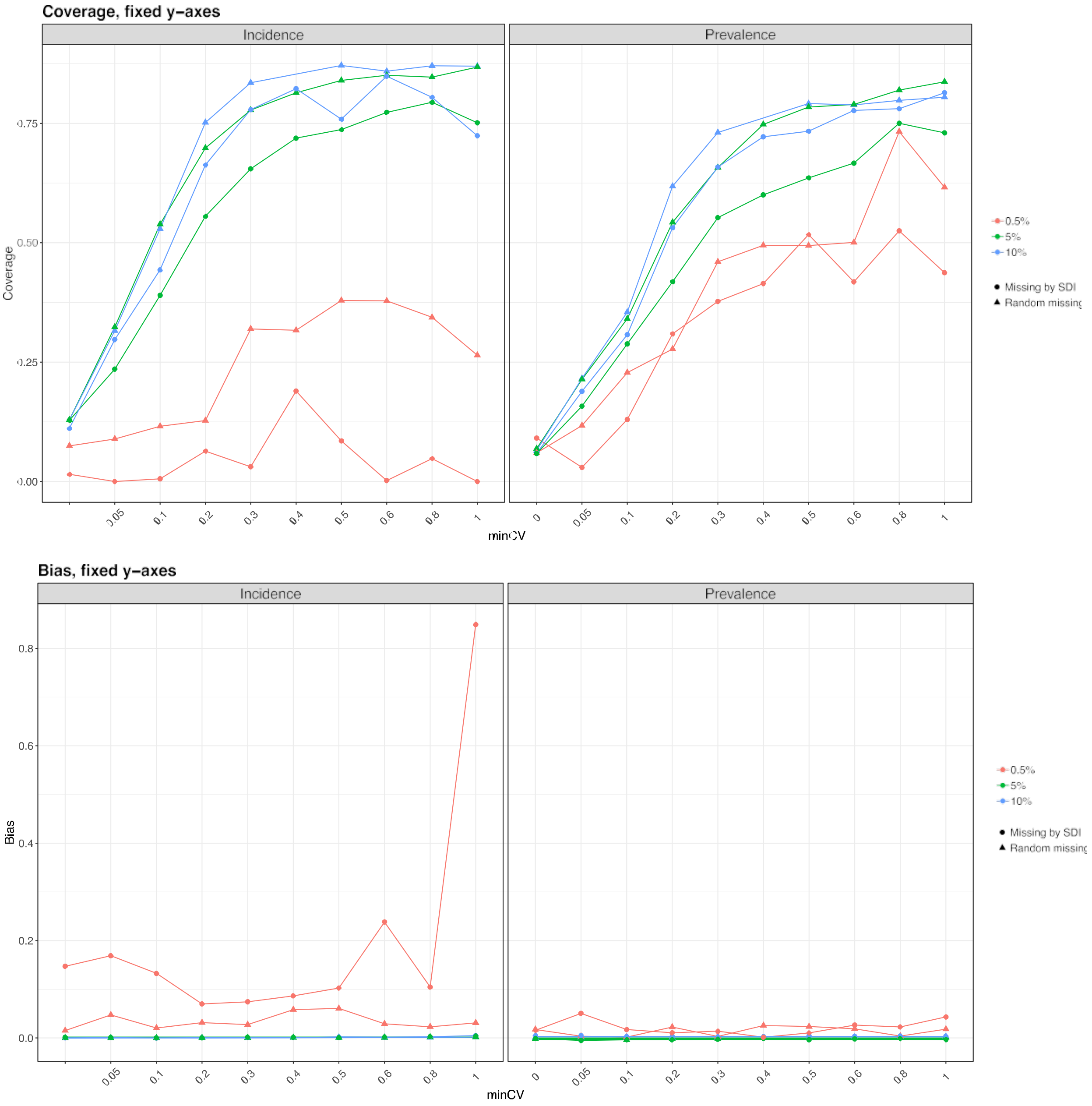

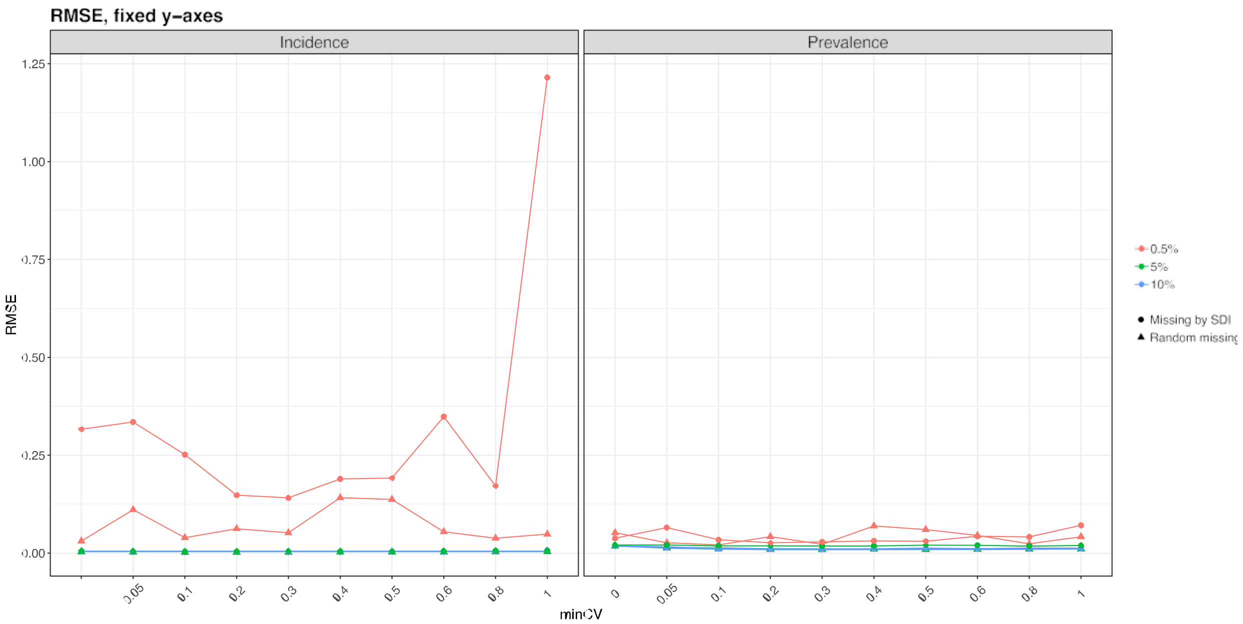

A third strategy was to apply a further distinction of complete culling of either prevalence and CSMR, or incidence data points, using the 10% randomly culled or 10% differentially culled datasets as comparators. In these scenarios, we found that the coverage statistic starts to level off at a value of 0.8 for minimum CV. All three metrics are much worse for datasets with incidence data culled. Performance statistics for this strategy are shown in figure 3.

Figure 3. Performance statistics comparing datasets with specific measures held out vs. randomly or differentially culled datasets.

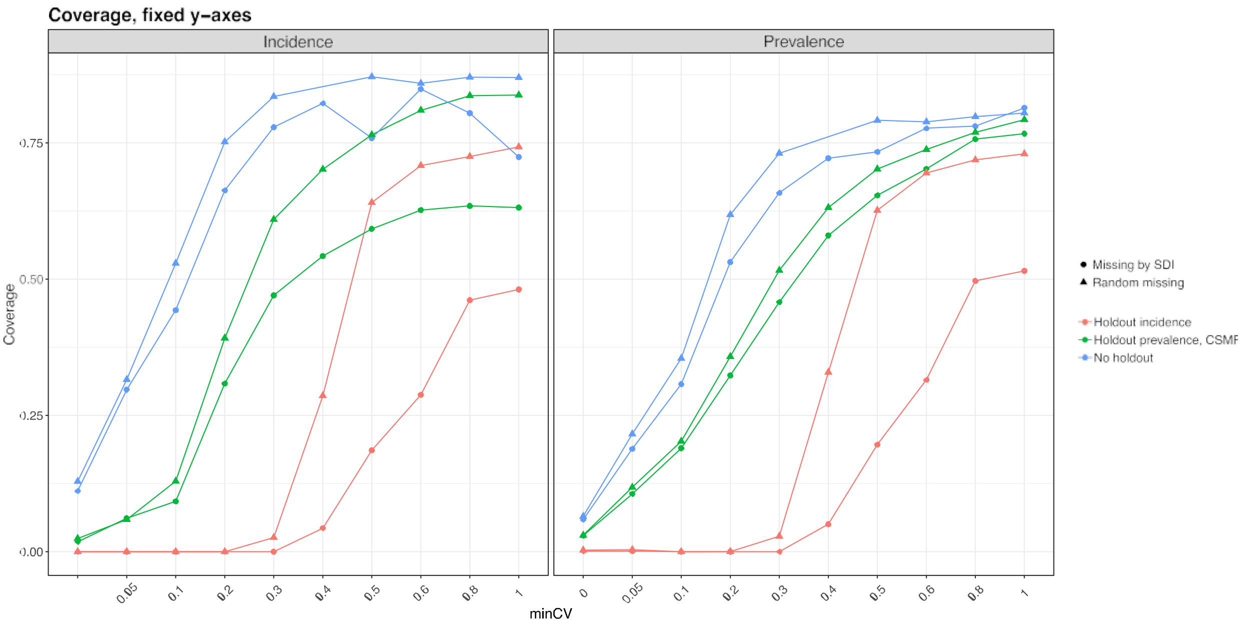

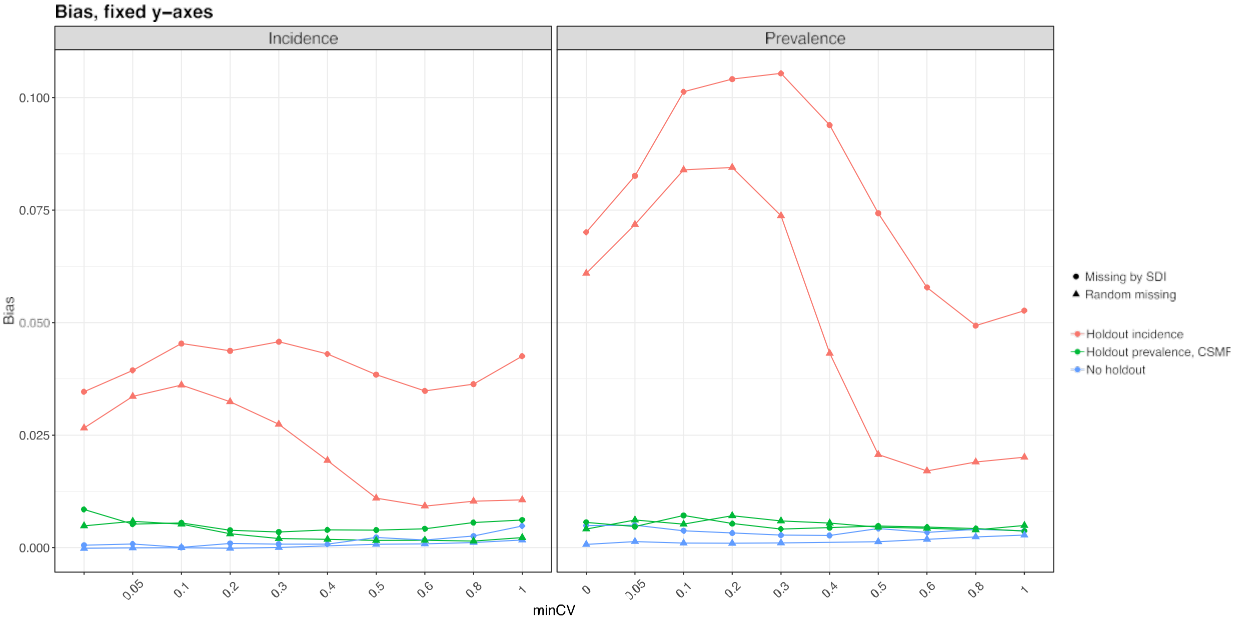

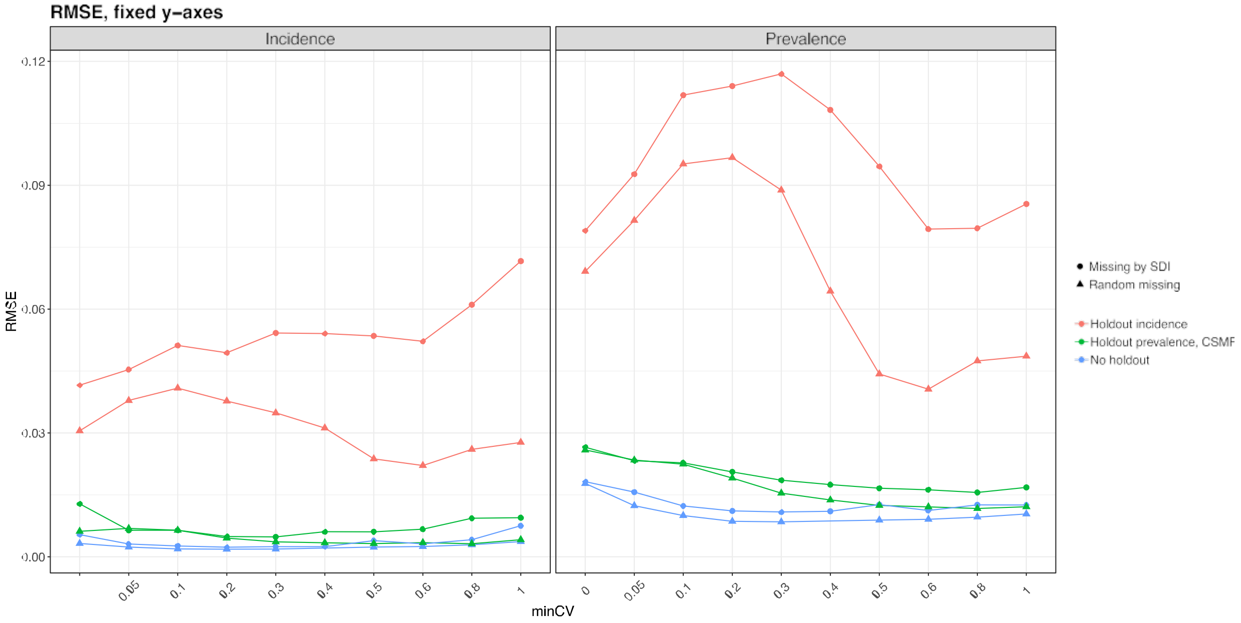

Fifth, we changed our approach to estimating excess mortality rates, the key link in the model between cause-specific mortality rates (CSMR) and incidence and prevalence. In the past two GBD rounds we calculated priors on excess mortality and entered these as data points by matching sex-specific prevalence data with an age width of 20 or less with the corresponding CSMR for the same location and year. For stability sake, we excluded calculation of EMR for prevalence data points of less than 1 in a million. EMR is simply calculated as CSMR divided by prevalence. As with previous GBD years, for diseases with an average duration of less than a year (as indicated by a setting of remission greater than one), we ran an initial global model to get an equivalent prevalence and used the following formula to calculate EMR:

$$EMR = {}^{CSMR * (remission + (ACMR - CSMR) + EMR\_pred)}/_{incidence}$$

where,

ASMR is the all-cause mortality rate

EMR_pred is the EMR fit from an initial global DisMod model

Despite using the log of LDI or the HAQ Index as a covariate with a prior that the coefficient had to be negative, we found many disease models with an implausible distribution of mortality to prevalence (or incidence) ratios implying lower case fatality in locations with lower HAQ Index than in countries with higher HAQ Index. This likely signals an inconsistency between fatal and non-fatal data inputs. For GBD 2019, we decided to run regressions on EMR data (calculated as described above) first using MR-BRT with HAQ Index as a predictor. In general, we tend to think that CSMR estimates are more robust than non-fatal data because of much greater data availability and a lesser task in adjusting cause death data for garbage coding than the complex task of adjusting non-fatal data sources for alternative case definitions and study methods. To indicate that we would reduce the random effects on EMR and the minimum coefficient of variation for priors on EMR being created at each next level down the cascade. However, there were exceptions. For drug use disorders, the risk of overdose deaths is less a function of a country's quality of health services but driven more by the availability of harm reduction strategies such as opioid substitution therapy and the availability of highly potent opioids such as fentanyl, which have been an important contributor to the large increase in overdose deaths in the USA in the last decade. We settled on a model for opioid use disorder with wider random effects and higher minimum coefficient of variation to give less emphasis on CSMR when enforcing consistency with prevalence data. In a next round, we will work to find covariates that are more relevant to drug overdose deaths such as a grading of harm reduction strategies by country and over time. In the case of COPD, we noted that following the data on CSMR and EMR led to large increases in prevalence estimates in east Asia, Oceania and, to a lesser extent, south Asia. In the oldest age groups, prevalence estimates would be higher than the prevalence data for these locations and reach a level of close to 80% in the oldest age groups. In these locations, we will pay attention to how garbage codes are being redistributed onto COPD in the next round of GBD.

Section 4.5.3: DisMod-MR 2.1 likelihood estimation

Analysts have the choice of using a Gaussian, log-Gaussian, Laplace, or Log-Laplace likelihood function in DisMod-MR 2.1. The default log-Gaussian equation for the data likelihood is

$$-log\left[p\left(y_j|\Phi\right)\right] = \log\left(\sqrt{2\pi}\right) + \log\left(\delta_j + s_j\right) + \frac{1}{2}\left(\frac{\log\left(a_j + \eta_j\right) - \log\left(m_j + \eta_j\right)}{\delta_j + s_j}\right)^2$$

Where,

y_j is a "measurement value" (ie, data point)

Φ denotes all model random variables

η_j is the offset value, *eta*, for a particular "integrand" (prevalence, incidence, remission, excess mortality rate, with-condition mortality rate, cause-specific mortality rate, relative risk, or standardised mortality ratio)

a_j is the adjusted measurement for data point j, defined by

$$a_j = e^{(-u_j - c_j)} y_j$$

Where:

u_j is the total "area effect" (ie, the sum of the random effects at three levels of the cascade: super-region, region and country) and

c_j is the total covariate effect (ie, the mean combined fixed effects for sex, study level, and country level covariates), defined by

$$c_j = \sum_{k=0}^{K[I(j)]-1} \beta_{I(j),k} \hat{X}_{k,j}$$

with SD

$$s_j = \sum_{l=0}^{L[I(j)]-1} \zeta_{I(j),l} \hat{Z}_{k,j}$$

Where:

k denotes the mean value of each data point in relation to a covariate (also called x-covariate)

I(j) denotes a data point for a particular integrand, *j*

$\beta_{I(j),k}$ is the multiplier of the kth x-covariate for the ith integrand

$\hat{X}_{k,j}$ is the covariate value corresponding to the data point *j* for covariate *k*;

l denotes the SD of each data point in relation to a covariate (also called z-covariate)

$\zeta_{I(j),k}$ is the multiplier of the lth z-covariate for the ith integrand

δ_j is the SD for adjusted measurement *j*, defined by:

$$\delta_j = log\left[y_j + e^{(-u_j - c_j)}\eta_j + c_j\right] - log\left[y_j + e^{(-u_j - c_j)}\eta_j\right]$$

Where:

m_j denotes the model for the jth measurement, not counting effects or measurement noise, and defined by:

$$m_j = \frac{1}{B(j) - A(j)} \int_{A(j)}^{B(j)} I_j(\text{a})\ \text{da}$$

Where:

A(j) is the lower bound of the age range for a data point

B(j) is the upper bound of the age range for a data point

I_j denotes the function of age corresponding to the integrand for data point *j*

Section 4.6: Impairment and underlying cause estimation[2]

For GBD 2019, as in GBD 2017 and GBD 2016, we estimated the country-age-sex-year prevalence of nine impairments. Impairments in GBD are conditions or specific domains of functional health loss that are spread across many GBD causes as sequelae and for which there are better data to estimate the occurrence of the overall impairment than for each sequela based on the underlying cause. These impairments included anaemia, epilepsy, hearing loss, heart failure, intellectual disability, infertility, vision loss, Guillain-Barré syndrome, and pelvic inflammatory disease. Overall impairment prevalence was estimated by using DisMod-MR 2.1. We constrained cause-specific estimates of impairments, as in the 19 causes of blindness, to sum to the total prevalence estimated for that impairment. Anaemia, epilepsy, hearing loss, heart failure, and intellectual disability were estimated at different levels of severity. Estimates were made separately for primary infertility (those unable to conceive), secondary infertility (those having trouble conceiving again), and whether the impairment affected men and/or women. In the case of epilepsy, we determined the proportions with idiopathic and secondary epilepsy as well as the proportions with severe and less severe epilepsy by using mixed effects regressions. The sparse data for the proportion of seizure-free, treated epilepsy were pooled in a random effects meta-analysis. DisMod-MR 2.1 models produced country-, age-, sex-, and year-specific severity levels of hearing loss and vision loss. Because of limited information on the severity levels of intellectual disability, we assumed a similar distribution of severity globally based on random effects meta-analysis of IQ-specific data for the overall impairment. This assumption was supplemented by cause-specific severity distributions for chromosomal causes and iodine deficiency; the severity of intellectual disability included in the long-term sequelae of causes including neonatal disorders, meningitis, encephalitis, neonatal tetanus, and malaria was estimated in combined health states of multiple impairments such as motor impairment, blindness, and/or seizures.[55] We changed the name of the intellectual disability impairment to specify that estimates reflect cases arising during the developmental period, which we have defined as ages under 20 years. The severity of heart failure was derived from our Medical Expenditure Panel Surveys (MEPS) analysis and therefore was not specific for country, year, age, or sex.

A detailed description of the methods of each impairment can be found at the end of Section 4.12 of this appendix.

Section 4.6.1: Impairment squeeze

For impairments like epilepsy, intellectual disability, and blindness, mentioned above in Step 4, we often have better information regarding the total prevalence of the impairment rather than the prevalence of said impairment due to its various causes. For example, we have more data and a better idea of the total number of blind individuals (which we refer to herein as the blindness "envelope") in the world than we do the number of individuals who are blind due to a specific cause like retinopathy of prematurity or

cataract. We achieve this consistency by either squeezing or inflating the individual sequela prevalence values so that their sums fit into each appropriate envelope. Blindness, epilepsy, and/or intellectual disability appear in various combinations with motor impairment levels as sequelae for a number of neonatal disorders and infectious diseases like malaria and neonatal tetanus ("Moderate motor impairment with blindness and epilepsy due to neonatal tetanus", for example). This presents an extra challenge because any squeeze or inflation of one of the impairments making up a sequela affects the others.

We set some rules on how to do these adjustments sequentially. First, when the envelope of an impairment is smaller than the sum of all contributing causes, we redistribute the excess prevalent cases of combined impairment sequelae onto the sequelae that only have motor impairment (at a mild, moderate, or severe level) within the same cause grouping. Second, we apply the adjustments in a particular order such that we always fit at least one of the envelopes exactly where the other one or two envelopes may be exceeded by some amount. We first enforce a fit to the epilepsy impairment envelope, then intellectual disability, and last, blindness. Thus, the epilepsy envelope always matches exactly, whereas the intellectual disability and blindness envelopes may occasionally be exceeded on a draw-by-draw basis.

Section 4.7: Severity distribution[2]

Sequelae were defined in terms of severity for 169 causes. We generally followed the same approach for estimating the distribution of severity we used in GBD 2017. In cases in which severity was related to a particular impairment, such as mild, moderate, and severe heart failure due to ischaemic heart disease or the newly added cause of pulmonary arterial hypertension, the analysis was driven by impairment estimation methods. Severity levels for causes such as chronic kidney disease, epilepsy and COPD were modelled using DisMod-MR 2.1 or ST-GPR, whereas we performed meta-analyses to estimate the allocation of severity for causes such as rheumatoid arthritis, and multiple sclerosis. For dementia, we changed from using meta-analysis of three age categories to a more flexible model in MR-BRT using a spline on age. That allowed us to increase the number of studies informing severity from 7 to 67. For gallbladder and biliary diseases, we performed a meta-analysis of six community-based studies of the proportion of cases of gallbladder disease identified by ultrasonography who are symptomatic. In previous rounds, inpatient admission for gall bladder and biliary disease as a primary diagnosis were taken to represent symptomatic cases. For the new cancer sites included in GBD 2019, we used the same strategy as for all other cancer sites. For the newly added sites of osteoarthritis of the hand and sites other than hip or knee, we assumed the same severity distribution as for osteoarthritis of the knee.

For many causes, we continue to have inadequate data on severity from surveys or the epidemiological literature. For those diseases, we made use of three population surveys: the MEPS 2000–2014, the [US] National Epidemiological Survey on Alcohol and Related Conditions (NESARC) 2000–2001 and 2004–2005, and the Australian National Survey of Mental Health and Wellbeing of Adults (NSMHWB) 1997.[56–58] Each dataset contained individual-level measurements of functional health status made by using the 12-Item Short Form Health Survey (SF-12) as well as diagnostic information on the causes affecting each individual.

To use the data collected by measuring the distribution of severity with the SF-12, the individual SF-12 summary scores were mapped to an equivalent DW. A convenience sample of respondents was asked to complete SF-12 for the hypothetical individual living in a health state described by using a selection of 60 of the 235 health states with their lay descriptions from the GBD DW surveys reflecting the full range of severity. Each of these health states has a measured DW associated with it on a zero to one scale. We collected 1980 usable responses in total. To deal with heterogeneity in responses, we excluded from the statistical analysis responses that were more than two median absolute deviations from the median for each health state. After correcting for outliers, the rank order correlation between SF-12 scores for the hypothetical individuals in each health state characterised by the lay description with the measured DW was -0.815. The health states served as random effect groups such that the composite score would be equal to the intercept plus the random effect estimated for that health state, or

$$DW_i = \alpha + U_{health\ state}$$

The final relationship between SF-12 score and DW is depicted in figure A:

Figure A. SF-12 composite scores and disability weights for 60 health states with fitted loess regression

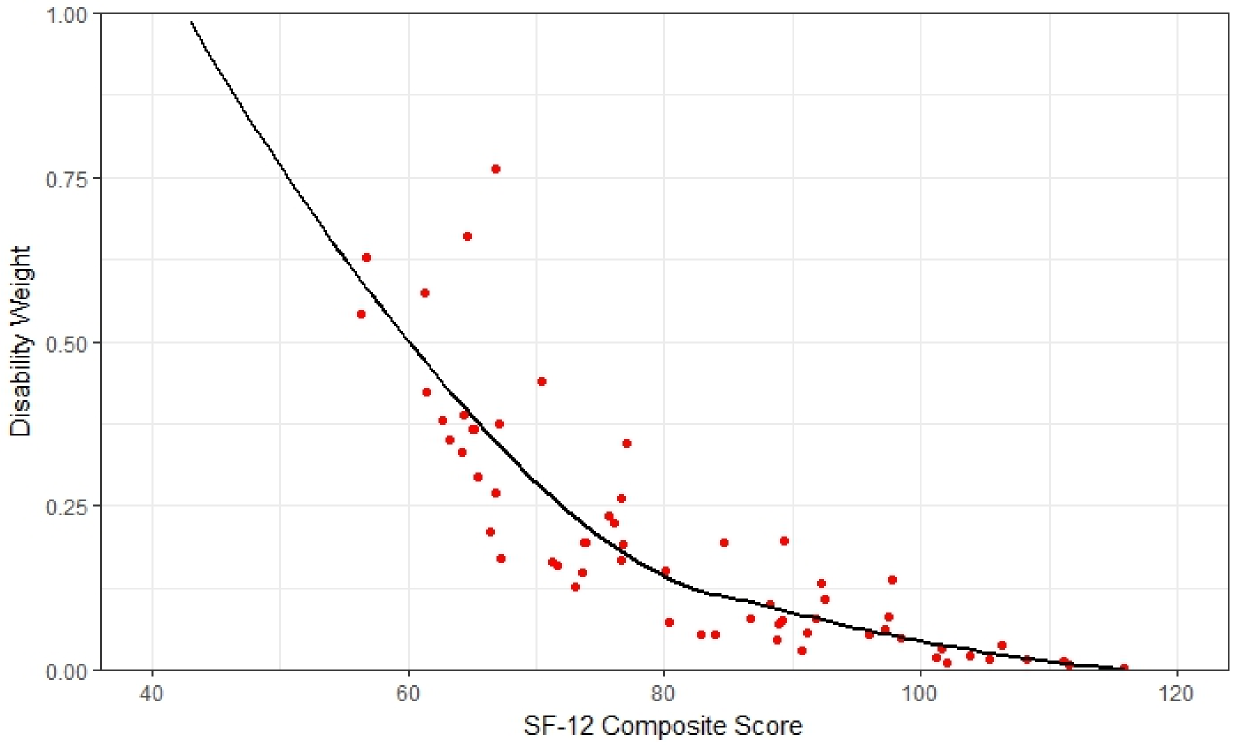

To generate a smooth mapping from SF-12 combined scores to the GBD DW space, we used locally estimated scatterplot smoothing regression on the random effects for each health state. Because DWs are defined in the range from 0 to 1, we truncated the function at a combined SF-12 score of 116.36 (any combined score above this level was set to 0) and truncated the function at 42.7 so that any combined score less than that value was set to 1. All SF-12 survey data were thus transformed into DW space.

The second stage of the analysis was to build models predicting the transformed SF-12 scores as a function of the number of causes suffered by each individual. First, variable selection was performed by using least absolute shrinkage and selection operator (LASSO) regression to penalize the regression coefficients of highly correlated causes. The tuning parameter, λ, controls the strength of the least-squares penalty. When λ=0, LASSO regression returns the same results as ordinary least-squares regression. Higher values of λ impose a stronger penalty and constrain a greater number of model parameters to 0. A ten-fold cross-validation was used to find the value of the λ that minimized the mean cross-validated error. This process resulted in a λ value of 0.0013 and eliminated 10 causes from the analysis. Transformed SF-12 scores into the DW scale for the remaining 190 causes were then modelled for each measure *m* of each individual *i* over *n* total causes in the survey as follows:

$$logit(DW)_{im} = \beta_0 + \beta_1 Condition1_{im} + \cdots + \beta_n Condition\ n_{im}$$

This equation effectively assumes that comorbid causes act to change SF-12 scores in a multiplicative fashion rather than an additive fashion.

To estimate the comorbidity-corrected effect of each cause (ie, in isolation) on total disability, we compared the predicted DW without the cause of interest (counterfactual DW) with the predicted DW including the cause of interest. Following the multiplicative comorbidity equation, the joint effect can be written

$$Condition\ specificDW = 1 - \frac{1 - predictedDW_m}{1 - counterfactualDW_m}$$

The mean of this cause-specific effect over all observations is the population marginal effect of a cause.

Using the model above, we estimate a counterfactual DW – the total individual DW excluding the effect of the cause of interest. We compared the observed distribution of functional health status with this counterfactual distribution to determine the marginal effect of the cause of interest. In other words, we estimated the health state for each individual and for each cause as the cumulative individual weight minus the effects of all comorbid causes.

$$Health\ state\ DW = 1 - \frac{1 - individual\ cumulativeDW_m}{1 - counterfactualDW_m}$$

The estimation strategy for health state-specific severity distributions for which there are multiple severity categories involved binning individuals' weights into severity cut-offs (eg, mild, moderate, and severe) for which DWs were derived. These bins were defined by using results from the GBD Disability Weights Studies[59] for causes that had multiple health states defined. Cut-offs were taken as the midpoints between levels of health state and cases distributed into severity bins accordingly. Cases were considered asymptomatic if the counterfactual weight was equal to or greater than the individual cumulative weight.

Section 4.8: Disability weights[2]

To compute YLDs for a particular health outcome in a given population, the number of people living with that outcome is multiplied by a DW that represents the magnitude of health loss associated with the

outcome. DWs are measured on a scale from 0 to 1; 0 implies a state equivalent to full health, and 1, a state equivalent to death.

DWs used in GBD studies before GBD 2010 have been criticized for the method used (ie, person trade-off), the small elite panel of international public health experts who determined the weights, and the lack of consistency over time as the GBD cause list expanded and additional DWs from a study in the Netherlands[60] were added or others were derived by ad-hoc methods.

Section 4.8.1: GBD 2010 disability weights measurement study

For GBD 2010, a primary data collection effort focused on measuring health loss rather than welfare loss by using a standardised approach of simple comparison questions directed to the general public across diverse communities.

Multi-country household surveys were conducted between Oct 28, 2009 and June 23, 2010 in five countries (Bangladesh, Indonesia, Peru, Tanzania, and the USA) selected to provide diversity across culture, language, and socioeconomic status.

Personal face-to-face computer-assisted interviews were conducted for all household surveys except for the survey in the US, which was conducted by computer-assisted telephone interview. Households were randomly selected by using a multistage stratified sampling design for which the probability of selection was proportional to the population size. In all cases, samples were designed to be representative of a given geographical area and, in the USA, to provide national representation.

For every contacted household, an adult respondent age 18 years or older was randomly selected by the survey program by means of the Kish approach. For face-to-face interviews, as many as three visits were made to selected households to establish contact. When a respondent was identified, as many as three return visits were made to do the survey at a time when the respondent was available. For the US telephone surveys, repeated calls were made up to seven times.

A web-based survey was posted at a dedicated URL between July 26, 2010 and May 16, 2011. The survey was initially available in English and subsequently available in Spanish and Mandarin. Recruitment of respondents occurred through several channels, such as news items and editorials in scientific journals, announcements at scientific meetings, postings on websites of institutions participating in the GBD, and social networking and communication mobilisation channels as well as direct contact with individuals and groups with known global health interests by tapping into the professional networks of the study investigators and their colleagues. Participants in the web-based survey were required to be ages 18 or older. Household surveys obtained oral informed consent from all participants; written informed consent was obtained from participants in the web survey. Ethical review board approval was obtained from each household survey site and the University of Washington, Seattle, WA.

Standardised survey instruments were developed to obtain comparative assessments of the full array of disease and injury sequelae, parsimoniously captured in 220 unique health states. Lay descriptions of health states formed the basis for all comparisons. These descriptions used simple, non-clinical vocabulary that emphasised the major functional consequences and symptoms associated with each

health state. Development of these descriptions involved an iterative process of detailed consultation with experts participating in the GBD 2010 study; the goals was to capture the most relevant details of each health state while avoiding ambiguity and ensuring consistency. When possible, health states were grounded in standard clinical classifications systems. For example, the Canadian Cardiovascular Society grading scale was referenced for descriptions of stages of angina,[61] and the New York Heart Association functional classification was referenced for severity of heart failure.[62] Pilot testing indicated that the lay descriptions in face-to-face interviews should not exceed 30 words.

A paired comparison question formed the basis of all surveys. The questions in the survey were framed with the following statement, “A person’s health may limit how well parts of his body or mind work. As a result, some people are not able to do all of the things in life that others may do, and some people are more severely limited than others. I am going to ask you a series of questions about different health problems. In each question, I will describe two different people…” Descriptions of two hypothetical people, each with a particular health state, were presented to respondents who were then asked which person they regarded as healthier. Health pairs in all surveys were selected by a randomizing computer algorithm. In the five household surveys, paired comparisons were presented for a subset of 108 health states pertaining to chronic conditions. The framing of chronic and acute conditions is different as they were presented as causing life-long or temporary health loss. We chose to only field health states that could be framed as lasting a lifetime in the household surveys as we hypothesized that presenting differently framed comparisons would be difficult to convey in face-to-face interviews. In the web survey, we considered this more feasible because respondents could read and refer to the framing of the question for each pair-wise comparison. All 220 health states were thus evaluated in the web survey.

In addition, the web survey included questions relating to population health and health programs specifically—such as “Imagine two different health programs. The first program prevented 1000 people from getting an illness that causes rapid death. The second program prevented 2000 people from getting an illness that is not fatal but causes lifelong health problems resulting in moderate to severe disability. Which program would you say produced the greater overall health benefits?” This information was used to anchor the results from the pair-wise comparisons on the 0–1 DW scale.

Section 4.8.2: GBD 2013 European disability weights measurement study

The GBD 2010 DWs were critically dependent on the ways that outcomes were described to survey respondents. Descriptions for health states were designed to balance validity and parsimony, and this approach necessarily meant that some details of different health states had to be omitted. Because lay descriptions were developed collaboratively through individual expert groups organised around a particular set of health issues, some amount of variability in language and detail inevitably occurred. Criticisms and suggestions for improvement came from a number of commentators on the GBD 2010 DWs measurement study.[63–65]

GBD 2013 expanded the list of disease and injury causes and sequelae mapped to 235 unique health states. Additional data for the European Disability Weights Measurement Study were collected between September 23, 2013 and November 11, 2013 in Hungary, Italy, the Netherlands, and Sweden. The

initiation of these surveys was connected to a project sponsored by the European Centre for Disease Prevention and Control (the Burden of Communicable Diseases in Europe project).[66] The four selected countries were chosen to be representative of the four regions of Europe (east, south, middle, and north) in terms of age, sex, and education of the respondents. Respondents were recruited from standing internet panels in each country on the basis of quota sampling with reference to age, sex, and education in such a way as to maintain the population representativeness of these characteristics. Eligible participants were 18–65 years old and were preselected in the Netherlands, where the age, sex, and education of respondents were already known, or in the other three countries, invited to participate via a web-link and then selected on the basis of their individual characteristics.

The protocol for the European DWs measurement study followed the protocol that was developed and implemented in the GBD 2010 DWs measurement study. Lay descriptions for some health states that lacked mention of an important symptom or for which consistency of wording across different levels of severity had been noted were reworded. The European DWs measurement study included 255 health states, of which 183 were used in the analyses of GBD 2013. Those 183 consisted of 135 of the 220 health states that were included in the European DWs measurement study with unmodified lay descriptions and 30 from GBD 2010 for which alternative lay descriptions were included. DWs were estimated for additional sequelae that were incorporated into GBD 2013 but had not been included in GBD 2010.

Finding high correlation in resulting DW values between the country surveys and the web survey, we analysed the results of all surveys together. We ran probit regression analyses on the answers to the pair-wise comparison questions by using dummies for each health state with a value of 1 for the first state in a pair, –1 for the second state in a pair, and 0 for all states other than the pair. This method formalizes the intuition that if two health states in a pair produce similar health loss, the answers are likely to be evenly split; a pair of health states with very different health loss get many more responses favouring one over the other. The statistical methods infer the distances between values attached to different health states based on the frequencies of responses to the paired comparisons.

A second analytic step is needed to anchor the resulting estimates onto the 0–1 DWs scale. We anchored results from the probit regression analysis onto the 0–1 scale by using population health equivalence data from the GBD 2010 web survey by using a linear regression of the probit coefficients from the analysis of paired comparisons on the logit-transformed DW estimates derived from interval regression of the population health equivalence responses. Using numerical integration, we then estimated mean values for DWs on the natural 0–1 scale. Uncertainty was estimated by bootstrapping with 1000 samples.

A complete listing of the lay descriptions and values for the 440 health states (including combined health states) used in GBD 2019 is provided in table S12.

Section 4.9: Comorbidity correction (COMO)[2]

The final stage in the estimation of YLDs is a micro-simulation, which adjusts for comorbidity. We refer to this micro-simulation process as “COMO” (for comorbidity correction). For GBD 2019, we estimated the co-occurrence of different diseases by simulating 40,000 individuals in each location-age-sex-year

combination as exposed to the independent probability of having any of the sequelae included in GBD 2019 based on disease prevalence. We tested the contribution of dependent and independent comorbidity in the US MEPS data and found that independent comorbidity was the dominant factor even though well-known examples of dependent comorbidity exist, such as clustering of conditions like diabetes and stroke or anxiety and alcohol use disorders. Age was the main predictor of comorbidity such that age-specific micro-simulations accommodated most of the required comorbidity correction.[67]

The two components necessary for the computation of YLDs, prevalence of each disease sequelae and DWs, are the two inputs into COMO. The prevalence values are primarily produced by using DisMod-MR 2.1. The DWs have been described earlier in this appendix.

The micro-simulation, as performed for each age-sex-location-year, can best be represented as a four-step process. First, simulants are exposed to independent probabilities of having each sequela, where the probability is equal to the prevalence estimate. For each simulant, the probability of having a disease sequela is equal to the estimated prevalence from that draw from the uncertainty distribution. Each simulant is determined to have or not have the disease sequelae based on a draw from a binomial distribution. From this simulation, simulants end up having from no to multiple disease sequelae. Second, the DW for each simulant is estimated on the basis of the disease sequelae that they have acquired. The formula for the cumulative DW for a simulant is one minus the multiplicative sum of one minus each DW present

$$Simulant\ DW_l = 1 - \prod_{k=i}^{j}(1 - DW_k)$$

Where:

DW_k is the DW for the kth disease sequela that the simulant *l* has acquired.

Once the simulant DW is computed, the DW attributable to each sequela for the simulant is calculated by using the following formula:

$$ADW_{lk} = \frac{DW_k}{\sum_{k=i}^{k=j} DW_k} * Simulant\ DW_l$$

Where:

ADW_{lk} is the attributable DW for disease sequela *k* in simulant *l*

DW_k is the DW for disease sequela *k*

Simulant DW_l is the DW for simulant *l* from the combination of all sequelae that they have acquired.

This formula apportions the overall simulant DW to each condition in proportion to the DW of each condition in isolation.

Finally, YLDs per capita in an age-sex-country-year are computed by taking the sum of the attributable DWs for a disease sequela across simulants.

$$YLD\ Rate_k = \frac{\sum_{l=1}^{n} ADW_{lk}}{n}$$

The actual number of YLDs from disease sequela *k* in an age-sex-location-year is then computed as the YLD rate *k* times the appropriate age-sex-location-year population.

By repeating the simulation process for each age-sex-country-year 1000 times, the uncertainty in the prevalence of each disease sequela and the DW is propagated into the final comorbidity corrected YLD results. We selected 40,000 simulants for each age-sex-location-year group on the basis of simulation testing, which has shown that results are stable for YLDs at this number of simulants even in the younger age groups when prevalence is relatively low. Mean results for YLDs that reflect 40 million simulants (40,000 simulants multiplied by 1000 iterations to capture uncertainty) are very stable in each age-sex-location-year. For any given location-year-age-sex group, sequelae with a prevalence of less than one in 20,000 were excluded from the micro-simulation.

Section 4.10: YLD computation, uncertainty, and residual YLDs[2]

For GBD 2019, we computed YLDs by sequela as prevalence multiplied by the DW for the health state associated with that sequela. The uncertainty ranges reported around YLDs incorporate uncertainty in prevalence and uncertainty in the DW. To do this, we take the 1000 samples of comorbidity-corrected YLDs and 1000 samples of the DW to generate 1000 samples of the YLD distribution. We assume no correlation in the uncertainty in prevalence and DWs. The 95% uncertainty interval is reported as the 25th and 975th values of the distribution. UIs for YLDs at different points in time (1990, 1995, 2000, 2005, 2010, and 2016) for a given disease or sequela are correlated because of the shared uncertainty in the DW. For this reason, changes in YLDs over time can be significant even if the UIs of the two estimates of YLDs largely overlap because significance is determined by the uncertainty around the prevalence estimates.

Section 4.10.1: Residual YLDs

Despite expanding our list of causes and sequelae in successive GBD iterations, many diseases remain for which we do not explicitly estimate disease prevalence and YLDs. Less common diseases and their sequelae were included in 35 residual categories (table S13). For 22 of these residual categories, epidemiological data on incidence or prevalence were available, so these were modelled accordingly. For 13 residual categories, epidemiological data on incidence and prevalence were not available, but sufficient CoD data allowed for CoD estimates. For these residual categories, we estimated YLDs by multiplying the residual YLL estimates by the ratio of YLDs to YLLs from the estimates Level 3 causes in the same disease category that were explicitly modelled. This scaling was done for each country-sex-year. This approach made the simplifying assumption that the residual diseases caused disability proportionate to the ratio of disability to mortality in explicitly modelled diseases. We did not include causes with large disability but no or little mortality in estimating these ratios. For example, we estimated the YLDs from other neurological disorders from the YLD to YLL ratios for dementia, multiple sclerosis, and Parkinson's disease but did not include the YLDs from headaches and epilepsy in the ratio.

Supplementary Figures and Tables

Global burden of peripheral artery diseases and its risk factors, 1990-2019: a systematic analysis for the Global Burden of Disease Study 2019

This appendix is an integral component of the article titled "Global Burden of Peripheral Artery Diseases and its Risk Factors, 1990-2019: A Systematic Analysis for the Global Burden of Disease Study 2019". Its purpose is to provide supplementary tables and figures that complement the primary analyses.

Figure S1. Age-standardised DALYs, deaths, prevalent cases, incident cases, YLDs, YLLs rates (per 100 000 persons) of PAD at the SDI region level. PAD= Peripheral artery disease. SDI=sociodemographic index.

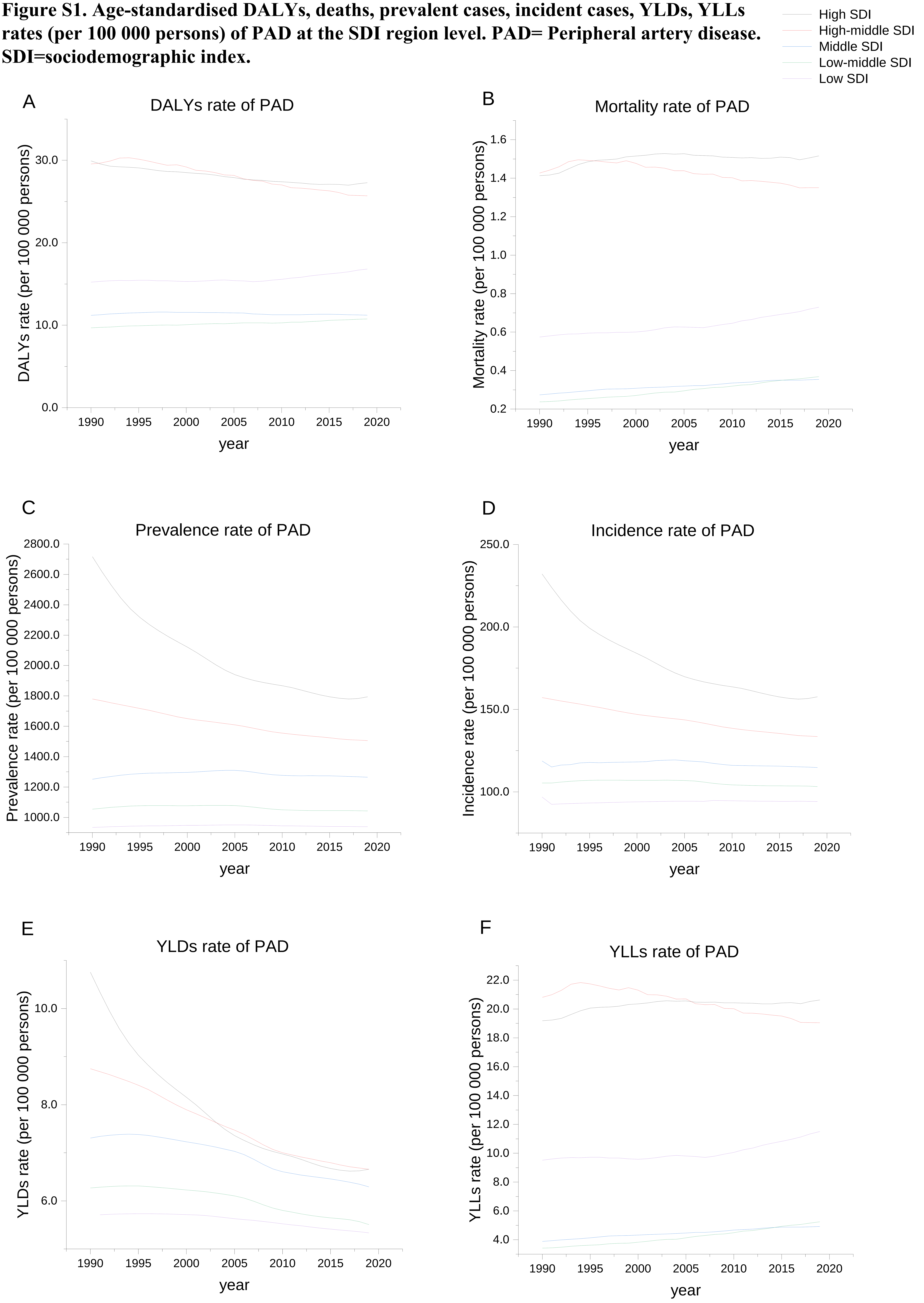

Figure S2. Age-standardised rate (per 100 000 persons) of DALYs, mortality, prevalence, and incidence of PAD at the SDI region level. SDI=sociodemographic index.

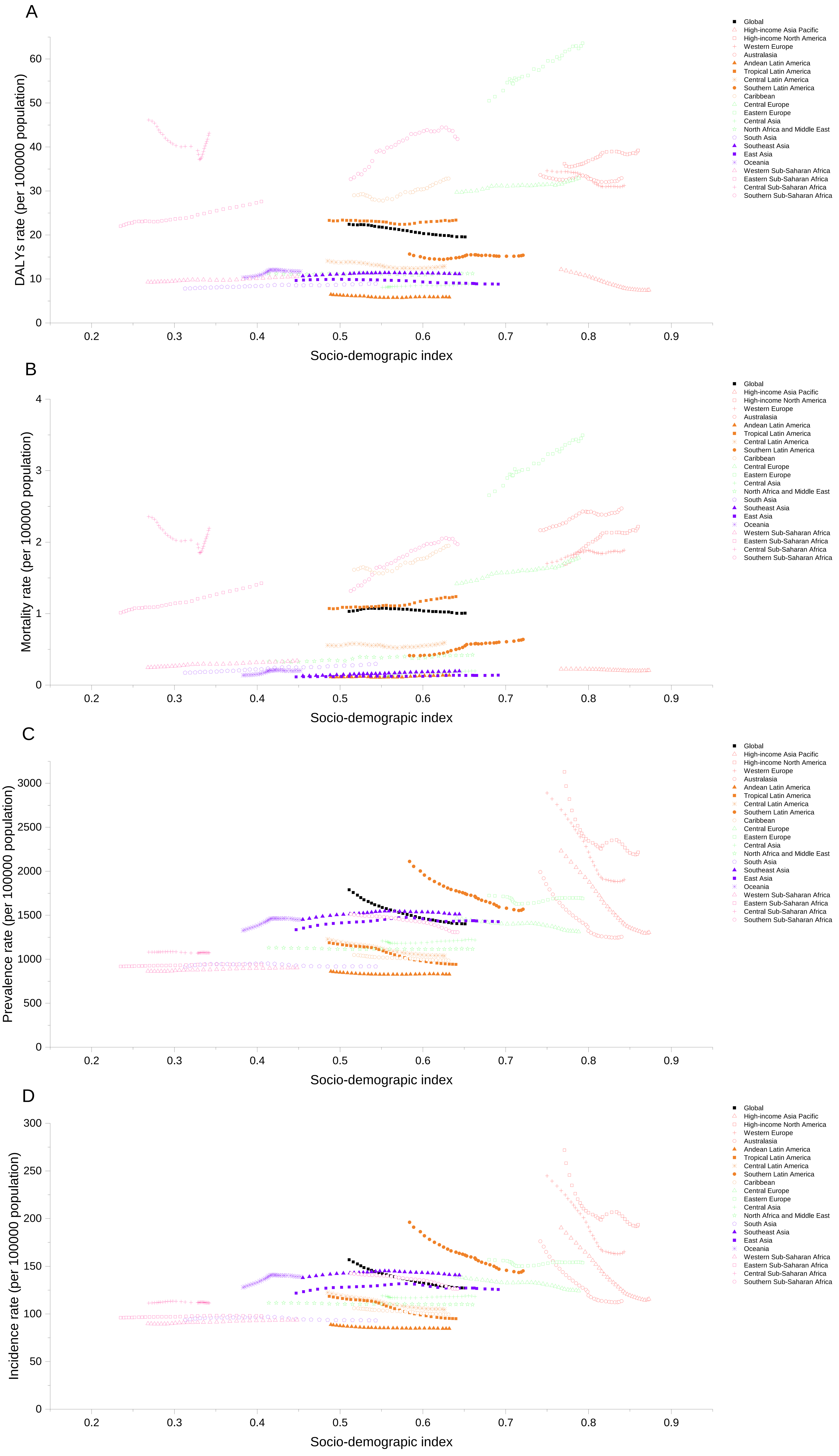

Figure S3. Correlation between SDI and age-standardised DALYs (per 100 000 persons) of PAD at the country level, 2019. The solid line denotes the linear trend between the two variables. Outlier countries are not shown.

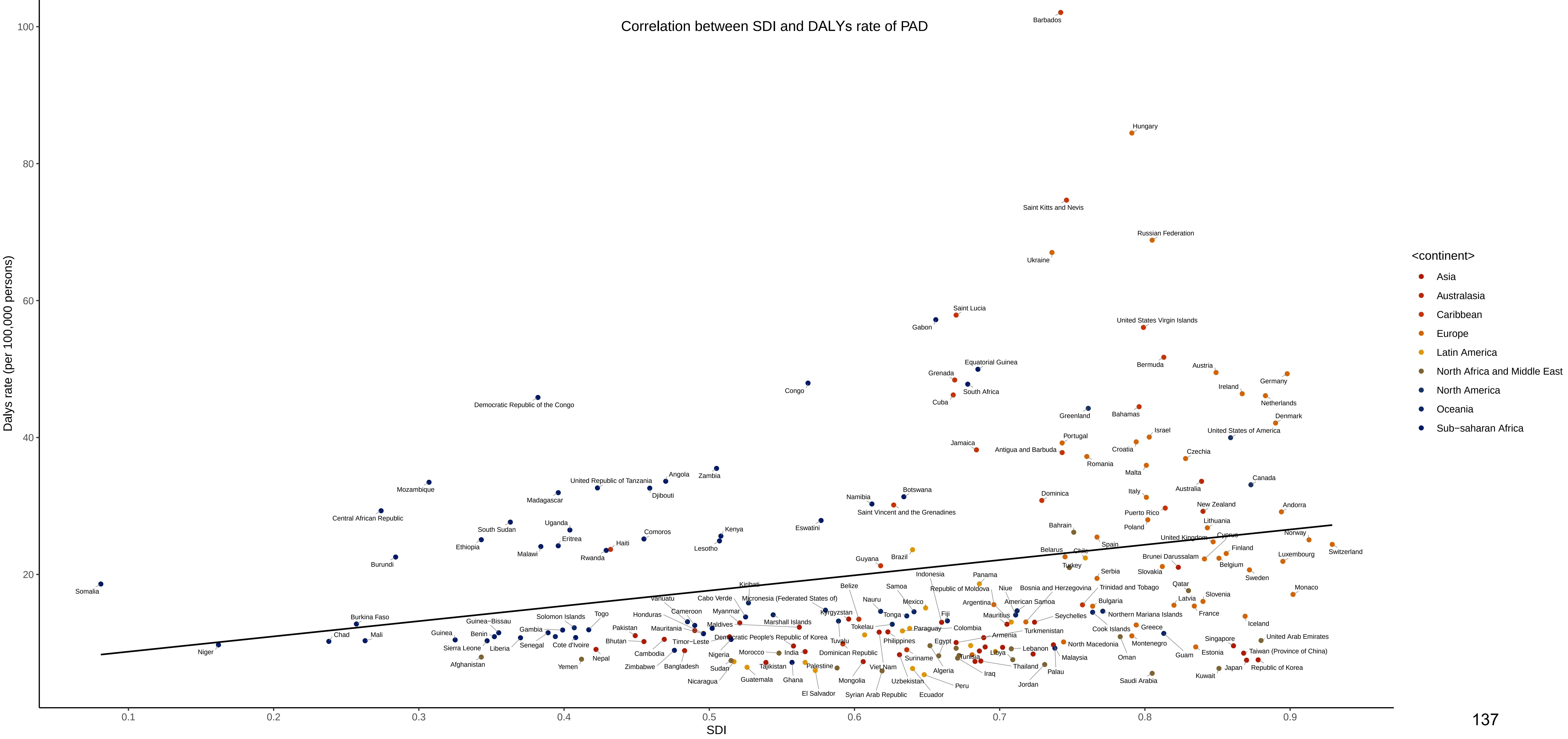

Figure S4. Numbers and age-standardized rates (per 100 000 persons) of PAD prevalence, incidence, DALYs, and deaths at the global level, 1990 to 2019. Error bars and shaded regions indicate 95% uncertainty intervals. PAD= Peripheral artery disease. DALYs=disability adjusted life-years

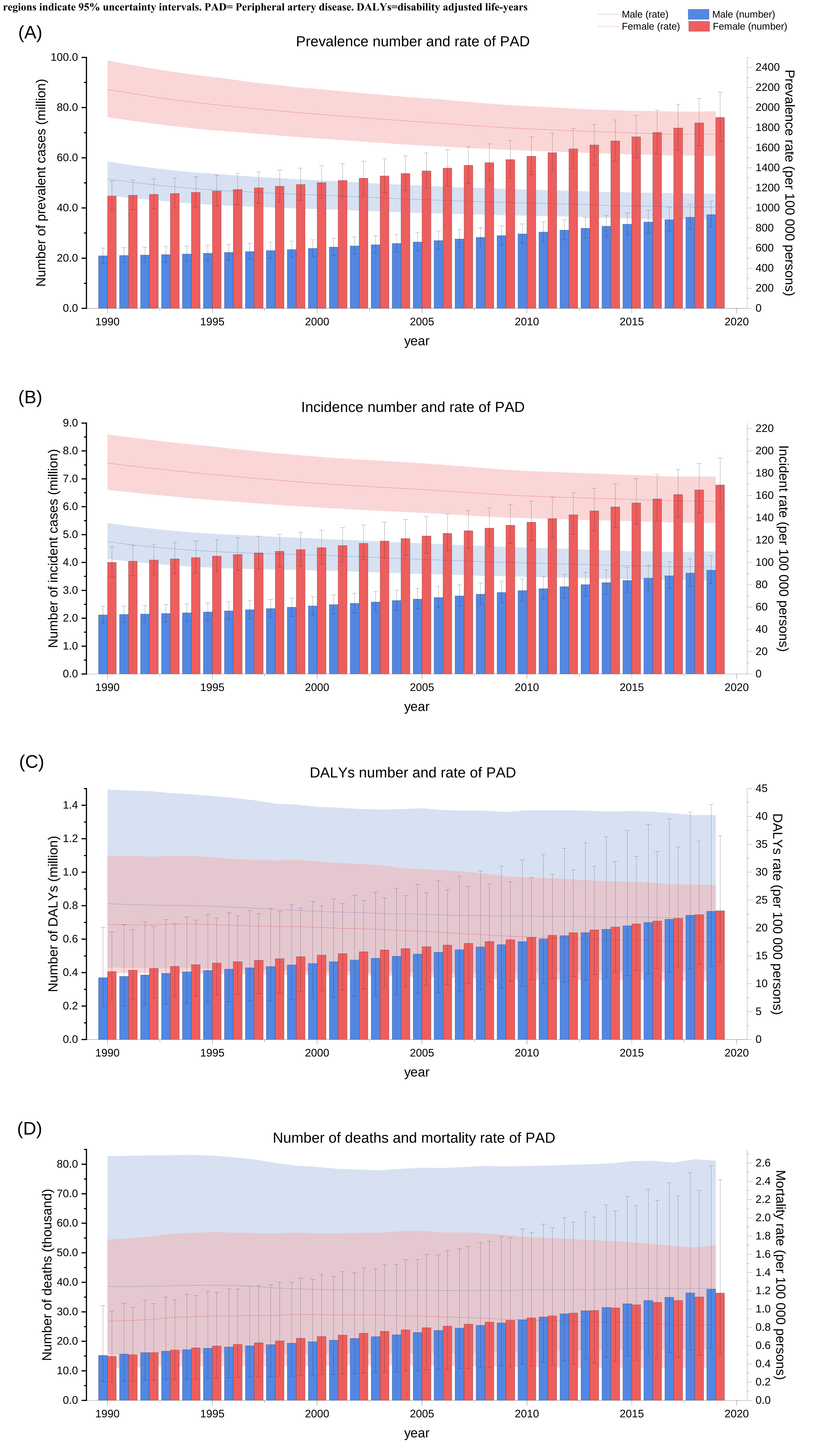

Figure S5. Age-standardised DALY rates (per 100 000 persons) of PAD attributable to risk factors by SDI regions and sex. DALYs=disability-adjusted life-years. SDI= sociodemographic index.

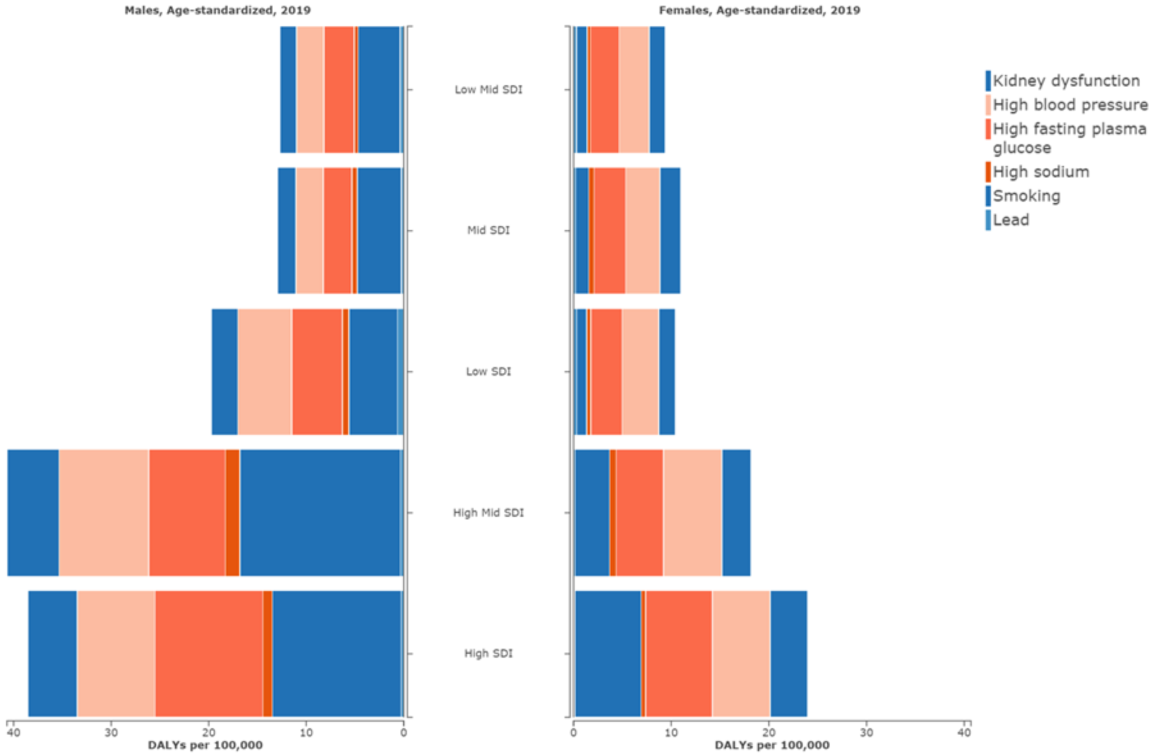

Table S1: PAD prevalence in 1990 and 2019 for both sexes and percentage change in age-standardised rates by location. PAD= Peripheral artery disease.

Prevalence of PAD	1990		2019		Percentage change in age-standardised rates between 1990 and 2019
	Counts (95% UI)	Age-standardised rate (95% UI)	Counts (95% UI)	Age-standardised rate (95% UI)	
Global	65764499 (57211022, 74527808)	1,790.0 (1,564.2, 2,033.3)	113443017 (99158208, 128415296)	1,401.8 (1,228.5, 1,589.4)	-21.7 (-22.8, -20.5)
East Asia	10842540 (9300880, 12389879)	1,335.8 (1,158.2, 1,523.1)	29589160 (25557455, 33841453)	1,426.7 (1,238.8, 1,627.0)	6.8 (5.5, 8.1)
China	10399944 (8919419, 11886890)	1,330.4 (1,153.9, 1,516.9)	28489637 (24548557, 32612500)	1,423.8 (1,234.8, 1,625.3)	7.0 (5.7, 8.3)
Democratic People's Republic of Korea	203119 (173614, 231765)	1,362.0 (1,182.9, 1,551.1)	444888 (382633, 511303)	1,403.8 (1,210.9, 1,614.9)	3.1 (-1.8, 7.9)
Taiwan	239477 (204364, 275097)	1,597.3 (1,376.5, 1,825.5)	654636 (579325, 723865)	1,635.9 (1,445.7, 1,810.8)	2.4 (-4.4, 11.6)
Southeast Asia	3407929 (2915562, 3909295)	1,450.1 (1,259.0, 1,649.3)	8805691 (7584637, 10038684)	1,511.7 (1,314.0, 1,725.4)	4.2 (2.8, 5.7)
Cambodia	59742 (51065, 68598)	1,471.9 (1,268.2, 1,683.4)	159972 (137152, 182687)	1,432.9 (1,240.1, 1,630.6)	-2.6 (-7.4, 1.7)
Indonesia	1300888 (1110345, 1490527)	1,461.3 (1,264.6, 1,666.7)	3324594 (2850201, 3802883)	1,640.1 (1,423.8, 1,871.0)	12.2 (10.2, 14.2)
Lao People's Democratic Republic	28408 (24312, 32720)	1,509.5 (1,306.8, 1,734.2)	59332 (50969, 67820)	1,464.4 (1,268.9, 1,669.9)	-3.0 (-7.2, 1.5)
Malaysia	125685 (108231, 144028)	1,465.0 (1,263.0, 1,684.8)	364958 (314992, 415415)	1,428.6 (1,239.9, 1,623.5)	-2.5 (-6.4, 2.0)
Maldives	1123 (953, 1298)	1,370.4 (1,185.4, 1,564.5)	4060 (3494, 4619)	1,410.2 (1,213.8, 1,607.2)	2.9 (-2.0, 7.4)
Mauritius	10638 (9114, 12217)	1,560.3 (1,350.0, 1,786.7)	29038 (25041, 33410)	1,679.0 (1,448.0, 1,928.6)	7.6 (3.2, 12.0)
Myanmar	380798 (325398, 439660)	1,796.5 (1,550.3, 2,055.8)	740494 (635261, 845852)	1,678.4 (1,454.3, 1,914.2)	-6.6 (-11.3, -2.0)
Philippines	382758 (328845, 438524)	1,397.6 (1,215.7, 1,597.8)	1103080 (949102, 1262397)	1,513.0 (1,314.5, 1,724.1)	8.3 (7.4, 9.1)
Seychelles	802 (692, 916)	1,434.9 (1,241.8, 1,632.8)	1614 (1388, 1843)	1,515.7 (1,312.9, 1,735.4)	5.6 (1.2, 10.4)
Sri Lanka	136273 (116210, 155495)	1,344.0 (1,159.5, 1,529.2)	367420 (316266, 421422)	1,464.3 (1,267.8, 1,671.0)	8.9 (4.7, 14.6)
Thailand	463416 (397569, 531341)	1,403.5 (1,211.3, 1,599.2)	1303408 (1122865, 1484540)	1,269.0 (1,094.5, 1,449.2)	-9.6 (-13.5, -5.4)
Timor-Leste	3417 (2906, 3944)	1,361.1 (1,171.8, 1,547.4)	11021 (9462, 12722)	1,425.5 (1,231.5, 1,632.6)	4.7 (-0.1, 9.9)

Viet Nam	509453 (438546, 581452)	1,337.4 (1,158.9, 1,521.5)	1325164 (1137058, 1514630)	1,487.3 (1,284.3, 1,694.0)	11.2 (6.5, 16.9)
Oceania	35028 (30019, 40277)	1,326.8 (1,157.3, 1,513.9)	92183 (78753, 105437)	1,450.5 (1,258.7, 1,653.5)	9.3 (6.6, 12.9)
American Samoa	315 (270, 361)	1,536.7 (1,329.0, 1,750.6)	780 (669, 892)	1,695.9 (1,466.5, 1,935.0)	10.4 (5.8, 15.0)
Cook Islands	167 (143, 191)	1,402.9 (1,204.1, 1,592.1)	395 (341, 452)	1,590.7 (1,374.8, 1,815.4)	13.4 (8.3, 18.2)
Fiji	5186 (4458, 5950)	1,606.6 (1,391.3, 1,842.1)	12301 (10531, 14104)	1,774.9 (1,535.1, 2,020.3)	10.5 (6.2, 14.8)
Guam	840 (718, 960)	1,231.3 (1,067.1, 1,396.9)	2713 (2330, 3086)	1,437.8 (1,239.0, 1,638.4)	16.8 (12.2, 22.1)
Kiribati	506 (431, 587)	1,562.2 (1,347.6, 1,798.7)	1219 (1036, 1398)	2,009.4 (1,734.3, 2,306.4)	28.6 (23.1, 34.7)
Marshall Islands	196 (168, 226)	1,345.0 (1,157.7, 1,545.1)	453 (387, 520)	1,467.4 (1,269.2, 1,670.8)	9.1 (4.6, 13.7)
Micronesia (Federated States of)	568 (482, 656)	1,358.9 (1,174.5, 1,556.3)	952 (813, 1102)	1,498.4 (1,294.4, 1,722.9)	10.3 (5.2, 15.2)
Nauru	44 (38, 51)	1,400.2 (1,204.1, 1,585.9)	55 (46, 64)	1,569.7 (1,350.0, 1,788.5)	12.1 (7.3, 17.3)
Niue	32 (28, 37)	1,428.4 (1,232.9, 1,638.4)	35 (30, 39)	1,589.9 (1,376.4, 1,814.8)	11.3 (6.3, 15.9)
Northern Mariana Islands	192 (163, 221)	1,284.9 (1,107.7, 1,470.1)	707 (601, 820)	1,410.8 (1,225.4, 1,602.0)	9.8 (5.6, 14.5)
Palau	126 (108, 146)	1,400.1 (1,206.9, 1,605.0)	314 (269, 360)	1,567.0 (1,353.4, 1,782.9)	11.9 (7.5, 17.1)
Papua New Guinea	20370 (17424, 23564)	1,241.5 (1,081.0, 1,418.7)	57490 (49211, 65843)	1,356.0 (1,173.6, 1,550.6)	9.2 (4.8, 14.4)
Samoa	1281 (1096, 1480)	1,569.3 (1,351.2, 1,814.6)	2479 (2140, 2839)	1,783.9 (1,542.9, 2,044.5)	13.7 (8.8, 18.3)
Solomon Islands	1540 (1303, 1778)	1,295.7 (1,122.5, 1,478.4)	3959 (3372, 4550)	1,462.7 (1,269.7, 1,676.4)	12.9 (8.3, 17.9)
Tokelau	19 (16, 21)	1,402.2 (1,207.5, 1,600.2)	20 (17, 23)	1,554.6 (1,339.8, 1,788.2)	10.9 (6.2, 15.6)
Tonga	804 (687, 922)	1,529.3 (1,320.7, 1,746.4)	1320 (1138, 1512)	1,695.2 (1,460.3, 1,944.2)	10.9 (5.9, 15.2)
Tuvalu	89 (76, 102)	1,417.6 (1,231.1, 1,616.7)	153 (131, 175)	1,563.8 (1,352.9, 1,791.4)	10.3 (5.1, 15.2)
Vanuatu	812 (696, 936)	1,365.4 (1,174.8, 1,573.4)	2486 (2132, 2832)	1,547.3 (1,337.0, 1,764.1)	13.3 (8.7, 18.7)
Central Asia	526692 (456717, 603097)	1,208.6 (1,048.1, 1,388.5)	809162 (698260, 926301)	1,218.1 (1,060.0, 1,392.9)	0.8 (-1.1, 2.8)

Armenia	35872 (30855, 41325)	1,444.4 (1,243.2, 1,670.1)	57535 (49552, 65613)	1,396.8 (1,207.7, 1,597.0)	-3.3 (-7.3, 1.4)
Azerbaijan	59713 (51837, 68140)	1,289.8 (1,117.3, 1,481.1)	106309 (91739, 122379)	1,256.5 (1,093.1, 1,440.1)	-2.6 (-6.5, 1.8)
Georgia	74486 (63956, 85212)	1,264.9 (1,092.6, 1,437.3)	84434 (72827, 97240)	1,368.5 (1,182.4, 1,573.4)	8.2 (3.7, 13.2)
Kazakhstan	152819 (131534, 174655)	1,304.0 (1,125.7, 1,491.0)	200342 (173659, 231065)	1,246.2 (1,080.1, 1,430.8)	-4.4 (-8.5, 0.3)
Kyrgyzstan	32213 (27904, 36903)	1,108.4 (957.3, 1,275.6)	45905 (39751, 52493)	1,086.9 (940.4, 1,250.5)	-1.9 (-6.2, 2.5)
Mongolia	11401 (9821, 13113)	1,190.2 (1,034.3, 1,360.3)	23998 (20678, 27487)	1,169.7 (1,015.1, 1,339.9)	-1.7 (-6.3, 2.6)
Tajikistan	34032 (29560, 38855)	1,267.2 (1,090.4, 1,454.4)	49151 (41894, 56593)	1,122.6 (973.1, 1,291.3)	-11.4 (-16.0, -6.8)
Turkmenistan	20738 (17797, 23875)	1,205.1 (1,039.4, 1,383.0)	42235 (36566, 48083)	1,186.9 (1,027.4, 1,345.8)	-1.5 (-5.6, 3.1)
Uzbekistan	105419 (91433, 121244)	999.4 (866.3, 1,151.3)	199254 (168132, 231663)	1,117.1 (961.3, 1,277.5)	11.8 (6.7, 17.0)
Central Europe	2105487 (1825699, 2413509)	1,467.7 (1,271.0, 1,675.2)	2849874 (2451339, 3276016)	1,313.0 (1,140.8, 1,498.9)	-10.5 (-11.6, -9.5)
Albania	24456 (21111, 28028)	1,261.0 (1,092.6, 1,441.6)	52897 (45490, 60930)	1,224.8 (1,058.1, 1,399.1)	-2.9 (-6.7, 1.3)
Bosnia and Herzegovina	51213 (44071, 59084)	1,361.9 (1,175.2, 1,573.5)	89386 (76650, 102522)	1,502.5 (1,297.3, 1,713.8)	10.3 (5.3, 15.6)
Bulgaria	163925 (139966, 189814)	1,362.7 (1,175.0, 1,561.8)	192659 (164421, 221475)	1,303.4 (1,124.4, 1,492.7)	-4.4 (-8.3, -0.4)
Croatia	96603 (83523, 110773)	1,551.0 (1,343.9, 1,777.6)	111798 (95867, 128248)	1,258.3 (1,082.5, 1,439.8)	-18.9 (-22.3, -15.1)
Czechia	200059 (173708, 229568)	1,460.9 (1,272.6, 1,663.4)	285774 (244180, 330121)	1,346.7 (1,162.5, 1,542.5)	-7.8 (-11.6, -4.0)
Hungary	233903 (199967, 268592)	1,604.0 (1,376.2, 1,836.9)	279171 (239475, 321333)	1,421.9 (1,229.0, 1,629.5)	-11.4 (-15.2, -7.4)
North Macedonia	24634 (21160, 28162)	1,402.5 (1,205.5, 1,599.8)	42496 (36453, 48809)	1,362.2 (1,182.2, 1,557.0)	-2.9 (-6.8, 1.1)
Montenegro	9457 (8188, 10846)	1,568.4 (1,359.2, 1,800.7)	15090 (12992, 17348)	1,539.3 (1,331.9, 1,759.3)	-1.9 (-5.9, 2.5)
Poland	688174 (594955, 786657)	1,608.1 (1,395.9, 1,831.5)	938060 (807643, 1076653)	1,324.8 (1,151.1, 1,518.8)	-17.6 (-18.8, -16.4)
Romania	348937 (299671, 401027)	1,291.2 (1,115.6, 1,479.3)	459460 (395531, 527547)	1,212.8 (1,045.8, 1,384.9)	-6.1 (-10.0, -1.9)
Serbia	145566 (125245, 166712)	1,338.6 (1,151.9, 1,532.1)	219796 (188926, 254334)	1,377.6 (1,193.5, 1,578.5)	2.9 (-1.4, 7.3)

Slovakia	84783 (72685, 97672)	1,443.0 (1,240.9, 1,655.8)	109160 (93211, 125431)	1,184.6 (1,017.6, 1,348.1)	-17.9 (-21.7, -13.7)
Slovenia	33776 (29278, 38669)	1,392.3 (1,208.7, 1,591.9)	54127 (46953, 62117)	1,228.8 (1,071.3, 1,397.5)	-11.7 (-15.7, -7.9)
Eastern Europe	4662015 (4033876, 5345501)	1,723.1 (1,490.0, 1,973.3)	5908656 (5117977, 6767587)	1,690.4 (1,467.3, 1,936.0)	-1.9 (-3.3, -0.5)
Belarus	190228 (164471, 219925)	1,486.0 (1,286.5, 1,716.2)	235302 (202815, 269690)	1,463.7 (1,262.5, 1,672.9)	-1.5 (-6.2, 3.2)
Estonia	31672 (27232, 36334)	1,552.4 (1,334.7, 1,774.8)	40511 (34845, 46696)	1,489.9 (1,288.7, 1,705.7)	-4.0 (-8.6, 0.7)
Latvia	48497 (41928, 55847)	1,352.6 (1,172.8, 1,556.1)	60018 (51278, 69057)	1,455.4 (1,251.9, 1,667.5)	7.6 (2.9, 12.9)
Lithuania	67788 (58461, 77458)	1,500.0 (1,294.5, 1,714.4)	83808 (71564, 96553)	1,407.2 (1,207.7, 1,621.3)	-6.2 (-10.5, -1.8)
Republic of Moldova	54214 (46476, 62556)	1,311.9 (1,133.2, 1,501.4)	80506 (69710, 92801)	1,389.8 (1,203.0, 1,600.3)	5.9 (1.3, 11.2)
Russian Federation	3038477 (2623840, 3478742)	1,756.5 (1,518.7, 2,010.3)	4092169 (3545347, 4676008)	1,725.6 (1,496.6, 1,973.1)	-1.8 (-2.6, -0.9)
Ukraine	1231139 (1055844, 1415289)	1,751.4 (1,513.0, 2,009.3)	1316341 (1133913, 1511731)	1,696.3 (1,467.0, 1,944.4)	-3.1 (-8.1, 1.7)
High-income Asia Pacific	4393074 (3832060, 4967066)	2,231.4 (1,949.4, 2,522.1)	6036002 (5210182, 6898991)	1,303.9 (1,132.7, 1,480.4)	-41.6 (-42.3, -40.8)
Brunei Darussalam	1831 (1578, 2062)	2,223.9 (1,920.8, 2,513.8)	3974 (3418, 4538)	1,490.0 (1,291.5, 1,704.8)	-33.0 (-35.8, -30.2)
Japan	3833448 (3339325, 4334234)	2,281.0 (1,991.6, 2,579.8)	4865501 (4190126, 5559461)	1,336.5 (1,163.3, 1,515.6)	-41.4 (-42.1, -40.8)
Republic of Korea	521975 (449839, 594596)	1,954.0 (1,698.4, 2,217.3)	1082554 (938137, 1236334)	1,212.8 (1,052.7, 1,381.3)	-37.9 (-40.9, -35.0)
Singapore	35820 (31003, 40663)	1,745.6 (1,504.3, 1,985.5)	83973 (72873, 95072)	1,090.8 (943.8, 1,236.6)	-37.5 (-40.0, -34.5)
Australasia	463198 (403604, 527003)	1,990.8 (1,739.8, 2,247.3)	625086 (539710, 712347)	1,254.3 (1,086.1, 1,424.4)	-37.0 (-39.3, -34.4)
Australia	383345 (333190, 436054)	1,979.0 (1,727.1, 2,231.1)	528028 (456204, 601185)	1,257.8 (1,088.2, 1,428.7)	-36.4 (-39.3, -33.5)
New Zealand	79853 (69355, 91158)	2,049.3 (1,789.7, 2,327.9)	97059 (83708, 110516)	1,235.1 (1,070.2, 1,401.5)	-39.7 (-42.3, -37.3)
Western Europe	16785956 (14626676, 18995580)	2,889.6 (2,533.8, 3,254.4)	17338264 (14957430, 19677419)	1,902.5 (1,659.4, 2,145.4)	-34.2 (-35.1, -33.3)
Andorra	1341 (1161, 1528)	2,539.5 (2,214.3, 2,870.6)	2673 (2313, 3030)	1,874.6 (1,618.5, 2,134.4)	-26.2 (-29.5, -22.9)
Austria	312973 (271005, 354655)	2,624.7 (2,282.2, 2,949.3)	348196 (301100, 398594)	1,941.6 (1,682.6, 2,210.2)	-26.0 (-29.0, -22.5)

Belgium	441459 (383764, 501889)	2,869.2 (2,497.5, 3,247.8)	487141 (419132, 556470)	2,138.0 (1,851.4, 2,441.6)	-25.5 (-28.7, -22.3)
Cyprus	22856 (19675, 26014)	2,830.3 (2,465.9, 3,191.3)	42474 (36644, 48632)	2,206.0 (1,912.5, 2,509.0)	-22.1 (-25.3, -18.7)
Denmark	339605 (294403, 384709)	4,164.3 (3,637.5, 4,679.2)	309279 (266224, 355866)	2,702.0 (2,342.0, 3,069.7)	-35.1 (-37.7, -32.4)
Finland	200628 (174748, 229058)	2,810.7 (2,454.2, 3,186.1)	227689 (195199, 261100)	1,870.1 (1,620.9, 2,116.1)	-33.5 (-36.3, -30.1)
France	2230656 (1939349, 2526037)	2,667.5 (2,316.5, 3,022.6)	2512844 (2163932, 2858215)	1,840.4 (1,590.2, 2,078.0)	-31.0 (-34.1, -27.8)
Germany	3773381 (3271873, 4264448)	2,959.3 (2,582.3, 3,345.4)	3631855 (3144031, 4151785)	1,902.4 (1,653.3, 2,157.6)	-35.7 (-38.3, -32.8)
Greece	440959 (381156, 502646)	2,908.0 (2,520.6, 3,302.7)	492622 (423552, 564616)	2,074.4 (1,794.7, 2,361.8)	-28.7 (-31.5, -25.6)
Iceland	8103 (7078, 9196)	2,838.0 (2,480.8, 3,202.3)	10485 (9083, 11894)	1,891.2 (1,643.7, 2,141.3)	-33.4 (-36.2, -30.5)
Ireland	130612 (112750, 149223)	3,211.8 (2,797.3, 3,630.8)	158354 (136384, 179786)	2,118.0 (1,829.2, 2,398.1)	-34.1 (-37.0, -31.4)
Israel	133387 (114802, 152519)	2,776.1 (2,395.3, 3,155.2)	237241 (206084, 269726)	2,050.4 (1,789.6, 2,324.6)	-26.1 (-29.2, -23.2)
Italy	2765866 (2408718, 3131092)	3,114.2 (2,729.8, 3,499.2)	2810306 (2432307, 3192485)	1,956.9 (1,706.0, 2,207.2)	-37.2 (-38.0, -36.2)
Luxembourg	16257 (14175, 18390)	2,977.0 (2,597.6, 3,347.5)	21667 (18844, 24544)	2,152.6 (1,868.6, 2,437.8)	-27.7 (-30.8, -24.7)
Malta	12999 (11351, 14667)	3,075.8 (2,702.1, 3,463.3)	20675 (17603, 23675)	2,245.0 (1,945.1, 2,546.4)	-27.0 (-30.1, -23.4)
Monaco	2055 (1778, 2331)	2,877.7 (2,510.7, 3,234.4)	1921 (1656, 2191)	2,009.9 (1,745.1, 2,272.0)	-30.2 (-33.0, -27.1)
Netherlands	569014 (506222, 627295)	2,850.9 (2,536.5, 3,130.8)	654478 (566471, 748473)	1,927.8 (1,669.9, 2,191.0)	-32.4 (-37.0, -27.6)
Norway	202217 (174789, 230108)	2,950.1 (2,580.6, 3,324.9)	196430 (170587, 223342)	2,048.2 (1,786.7, 2,324.4)	-30.6 (-31.4, -29.7)
Portugal	368345 (320053, 419542)	2,665.4 (2,328.2, 3,009.9)	467762 (400304, 534014)	1,944.2 (1,688.6, 2,206.4)	-27.1 (-30.1, -23.0)
Spain	1439309 (1255071, 1633465)	2,645.3 (2,317.3, 2,996.7)	1765206 (1525982, 2009998)	1,829.2 (1,586.2, 2,077.3)	-30.9 (-33.8, -28.1)
Sweden	435765 (378119, 497346)	2,862.7 (2,504.3, 3,239.6)	417845 (359234, 477785)	2,004.0 (1,743.7, 2,272.2)	-30.0 (-32.5, -27.5)
Switzerland	290442 (252117, 329679)	2,762.6 (2,412.8, 3,126.7)	342462 (296779, 390798)	1,966.0 (1,707.6, 2,240.3)	-28.8 (-31.7, -25.9)
United Kingdom	2632839 (2284041, 2999515)	2,897.6 (2,533.8, 3,275.1)	2162252 (1877277, 2461701)	1,725.7 (1,504.5, 1,956.4)	-40.4 (-41.0, -39.9)

Southern Latin America	949198 (827047, 1081767)	2,110.5 (1,841.1, 2,396.7)	1316077 (1125201, 1494772)	1,568.2 (1,344.5, 1,775.4)	-25.7 (-28.1, -23.0)
Argentina	659200 (572403, 753057)	2,090.8 (1,820.9, 2,382.9)	848538 (724285, 971680)	1,561.9 (1,337.0, 1,775.6)	-25.3 (-28.4, -21.5)
Chile	197119 (171168, 223393)	2,061.8 (1,790.7, 2,339.0)	372192 (319464, 426983)	1,545.9 (1,328.5, 1,771.4)	-25.0 (-28.4, -21.2)
Uruguay	92840 (80329, 105582)	2,381.5 (2,074.0, 2,689.1)	95280 (82000, 108623)	1,727.4 (1,486.8, 1,966.7)	-27.5 (-30.7, -24.3)
High-income North America	11112735 (9560431, 12726053)	3,129.7 (2,696.3, 3,559.7)	13961440 (12552572, 15445785)	2,214.3 (1,986.7, 2,433.8)	-29.2 (-33.7, -23.7)
Canada	1207429 (1050899, 1365121)	3,738.4 (3,265.7, 4,208.0)	1498376 (1293298, 1716850)	2,175.2 (1,881.7, 2,472.5)	-41.8 (-44.4, -39.0)
United States of America	9903935 (8490068, 11331986)	3,067.7 (2,638.6, 3,488.7)	12461375 (11235661, 13740797)	2,219.2 (1,998.2, 2,434.4)	-27.7 (-32.7, -21.4)
Greenland	1116 (974, 1258)	3,675.8 (3,225.2, 4,138.0)	1468 (1270, 1673)	2,252.3 (1,960.7, 2,570.8)	-38.7 (-41.1, -36.4)
Caribbean	263996 (227650, 303858)	1,047.7 (904.0, 1,203.0)	511218 (440162, 585168)	985.7 (848.4, 1,129.5)	-5.9 (-7.8, -3.8)
Antigua and Barbuda	561 (480, 646)	1,028.0 (884.1, 1,177.1)	947 (817, 1083)	969.3 (836.0, 1,107.9)	-5.7 (-9.6, -1.1)
Bahamas	1423 (1226, 1635)	1,000.3 (861.7, 1,144.8)	3486 (3029, 3964)	946.5 (814.2, 1,081.9)	-5.4 (-9.4, -0.8)
Barbados	3086 (2637, 3549)	1,025.3 (884.7, 1,167.4)	4858 (4178, 5582)	977.9 (844.6, 1,118.7)	-4.6 (-8.8, 0.2)
Belize	888 (767, 1020)	990.7 (854.4, 1,136.6)	2478 (2141, 2845)	962.2 (831.5, 1,103.3)	-2.9 (-6.7, 1.9)
Bermuda	600 (516, 689)	994.6 (859.4, 1,137.8)	1253 (1083, 1434)	942.0 (813.5, 1,075.2)	-5.3 (-9.2, -1.1)
Cuba	113065 (97325, 130951)	1,108.8 (955.1, 1,276.3)	192564 (164516, 221088)	991.0 (846.0, 1,139.0)	-10.6 (-14.2, -6.4)
Dominica	725 (618, 838)	988.6 (846.9, 1,133.9)	849 (726, 972)	932.6 (799.9, 1,067.6)	-5.7 (-9.7, -1.5)
Dominican Republic	35501 (30571, 40733)	1,030.1 (888.8, 1,176.3)	90328 (77879, 103688)	1,008.3 (867.8, 1,156.7)	-2.1 (-6.6, 2.9)
Grenada	764 (656, 883)	1,016.8 (880.2, 1,164.4)	1074 (922, 1231)	1,007.9 (866.7, 1,152.7)	-0.9 (-5.4, 4.0)
Guyana	3418 (2943, 3917)	998.2 (865.6, 1,144.9)	5581 (4797, 6441)	974.2 (843.1, 1,127.0)	-2.4 (-7.1, 2.6)
Haiti	26628 (22664, 30692)	938.5 (805.6, 1,073.1)	54515 (46730, 63026)	878.8 (754.9, 1,013.7)	-6.4 (-10.3, -2.1)
Jamaica	17848 (15308, 20482)	994.0 (860.8, 1,136.8)	32259 (27973, 36661)	1,068.1 (921.2, 1,217.9)	7.5 (2.7, 12.4)

Puerto Rico	36028 (30851, 41505)	995.1 (857.5, 1,138.8)	71957 (61462, 83018)	971.9 (836.1, 1,114.1)	-2.3 (-6.8, 1.7)
Saint Kitts and Nevis	377 (317, 440)	1,010.2 (869.2, 1,157.9)	591 (507, 677)	975.8 (839.8, 1,113.5)	-3.4 (-7.4, 1.0)
Saint Lucia	914 (775, 1056)	1,074.6 (916.3, 1,230.5)	2127 (1835, 2423)	1,002.3 (864.3, 1,146.2)	-6.7 (-10.3, -2.6)
Saint Vincent and the Grenadines	715 (611, 829)	1,018.9 (880.1, 1,171.6)	1332 (1151, 1525)	1,005.1 (867.2, 1,147.8)	-1.4 (-5.7, 3.1)
Suriname	2546 (2205, 2902)	1,033.3 (895.4, 1,182.6)	6153 (5286, 7038)	1,061.2 (910.0, 1,212.0)	2.7 (-1.6, 7.8)
Trinidad and Tobago	9365 (8009, 10780)	1,167.5 (1,001.5, 1,338.8)	19799 (17028, 22828)	1,083.1 (932.6, 1,243.3)	-7.2 (-11.4, -3.3)
United States Virgin Islands	748 (644, 857)	951.6 (816.9, 1,089.6)	1751 (1485, 2020)	946.0 (818.5, 1,078.9)	-0.6 (-5.4, 4.0)
Andean Latin America	164453 (141531, 188224)	861.3 (742.9, 988.6)	454206 (392058, 520945)	828.7 (715.4, 951.2)	-3.8 (-6.9, -0.8)
Bolivia (Plurinational State of)	27429 (23502, 31519)	945.4 (813.0, 1,080.5)	71634 (61492, 81722)	861.1 (741.8, 981.8)	-8.9 (-12.9, -4.7)
Ecuador	45728 (39412, 52468)	916.0 (789.4, 1,051.2)	126961 (109249, 147102)	866.8 (745.5, 999.2)	-5.4 (-9.2, -1.1)
Peru	91296 (78868, 104375)	815.7 (703.5, 936.2)	255611 (220996, 292493)	802.7 (690.0, 923.4)	-1.6 (-6.1, 3.2)
Central Latin America	943781 (815371, 1082009)	1,227.5 (1,058.9, 1,408.2)	2392336 (2073987, 2739949)	1,038.9 (897.5, 1,190.4)	-15.4 (-16.6, -14.2)
Colombia	189938 (162749, 219304)	1,186.9 (1,017.0, 1,364.1)	517035 (446922, 590506)	971.5 (838.5, 1,113.2)	-18.1 (-21.9, -14.8)
Costa Rica	19080 (16376, 21889)	1,146.1 (983.7, 1,313.7)	50918 (43614, 58363)	999.9 (854.8, 1,146.7)	-12.7 (-16.9, -8.7)
El Salvador	28241 (24433, 32358)	1,000.6 (863.1, 1,144.4)	58521 (50538, 67269)	971.5 (835.5, 1,119.2)	-2.9 (-7.5, 2.2)
Guatemala	31429 (26844, 36245)	975.0 (840.6, 1,119.7)	103508 (88912, 118873)	978.0 (842.8, 1,122.8)	0.3 (-4.2, 4.7)
Honduras	20824 (17811, 24101)	1,101.5 (946.1, 1,266.3)	57966 (49824, 66473)	1,027.6 (886.6, 1,182.1)	-6.7 (-11.0, -1.6)
Mexico	507716 (441147, 580759)	1,281.9 (1,108.1, 1,474.6)	1231095 (1068404, 1408469)	1,087.4 (940.4, 1,246.9)	-15.2 (-16.3, -13.9)
Nicaragua	15645 (13452, 17995)	1,125.5 (967.4, 1,290.9)	42195 (36431, 48475)	1,029.2 (886.2, 1,177.4)	-8.6 (-12.5, -4.3)
Panama	15053 (12832, 17253)	1,054.5 (902.3, 1,206.9)	40172 (34512, 45945)	968.6 (831.8, 1,110.1)	-8.1 (-12.0, -4.4)
Venezuela (Bolivarian Republic of)	115857 (99912, 132516)	1,302.0 (1,120.3, 1,494.7)	290926 (248944, 334939)	1,031.6 (883.1, 1,184.9)	-20.8 (-24.2, -16.5)

Tropical Latin America	986665 (851435, 1132472)	1,187.3 (1,029.4, 1,354.1)	2233807 (1944381, 2556679)	941.6 (817.7, 1,072.8)	-20.7 (-21.9, -19.6)
Brazil	962998 (830627, 1105385)	1,189.0 (1,031.5, 1,355.4)	2176800 (1893801, 2491765)	938.7 (815.1, 1,068.9)	-21.1 (-22.2, -19.9)
Paraguay	23667 (20438, 27112)	1,128.2 (971.3, 1,293.2)	57007 (49154, 65617)	1,068.4 (919.2, 1,232.4)	-5.3 (-9.5, -0.8)
North Africa and Middle East	1744752 (1500809, 2003449)	1,130.5 (984.2, 1,293.1)	4483708 (3873089, 5120535)	1,115.6 (966.1, 1,276.4)	-1.3 (-2.6, 0.1)
Afghanistan	63013 (53530, 73219)	959.0 (830.0, 1,103.1)	116559 (100012, 134096)	1,057.5 (911.8, 1,214.2)	10.3 (5.4, 15.4)
Algeria	126926 (107384, 147726)	1,131.9 (972.8, 1,300.8)	354303 (302934, 406257)	1,117.6 (957.5, 1,279.1)	-1.3 (-5.4, 3.2)
Bahrain	1843 (1574, 2126)	1,217.1 (1,052.5, 1,392.0)	10950 (9239, 12602)	1,185.5 (1,030.1, 1,351.3)	-2.6 (-7.0, 1.5)
Egypt	268125 (231469, 307926)	1,020.5 (892.1, 1,173.1)	634100 (541030, 733285)	1,098.2 (943.3, 1,270.7)	7.6 (3.1, 12.8)
Iran (Islamic Republic of)	245078 (209425, 282333)	1,077.0 (936.0, 1,228.8)	788551 (682504, 896622)	1,139.5 (989.9, 1,301.2)	5.8 (4.6, 7.1)
Iraq	99898 (86142, 114430)	1,388.7 (1,201.8, 1,591.7)	257562 (220751, 295983)	1,233.9 (1,061.9, 1,416.8)	-11.1 (-15.1, -7.5)
Jordan	14374 (12259, 16612)	1,215.2 (1,051.5, 1,401.8)	71948 (61963, 83080)	1,191.4 (1,029.6, 1,367.4)	-2.0 (-5.7, 2.3)
Kuwait	5831 (5016, 6635)	1,096.9 (949.4, 1,251.8)	27445 (23692, 31147)	1,119.9 (964.7, 1,278.0)	2.1 (-1.8, 6.0)
Lebanon	27761 (23803, 31933)	1,314.6 (1,132.7, 1,510.4)	70655 (60571, 81507)	1,364.4 (1,173.5, 1,570.3)	3.8 (-0.5, 8.1)
Libya	18575 (16019, 21137)	1,079.1 (931.1, 1,227.7)	53297 (46184, 60623)	1,113.0 (963.7, 1,277.6)	3.1 (-1.1, 7.5)
Morocco	135426 (116125, 155971)	1,088.7 (941.0, 1,250.3)	316061 (273103, 361695)	1,082.0 (936.2, 1,239.1)	-0.6 (-4.5, 3.5)
Palestine	8655 (7419, 9958)	1,073.9 (922.1, 1,226.8)	23350 (20007, 26706)	1,082.9 (937.9, 1,244.8)	0.8 (-3.7, 5.3)
Oman	6056 (5159, 6964)	1,075.7 (926.8, 1,228.4)	17970 (15379, 20724)	1,171.0 (1,007.0, 1,335.1)	8.9 (4.5, 13.7)
Qatar	1230 (1042, 1428)	1,264.6 (1,089.2, 1,453.8)	9863 (8247, 11536)	1,074.1 (930.3, 1,221.9)	-15.1 (-19.0, -11.4)
Saudi Arabia	50312 (43441, 57299)	967.1 (837.2, 1,104.2)	153378 (131220, 175890)	978.5 (844.5, 1,111.6)	1.2 (-3.5, 5.2)
Sudan	73000 (62456, 84433)	860.3 (745.3, 990.4)	157178 (135358, 179196)	918.3 (793.6, 1,047.5)	6.7 (2.0, 11.4)
Syrian Arab Republic	52878 (45357, 60719)	1,104.0 (957.2, 1,265.7)	121772 (104084, 139529)	1,044.8 (899.5, 1,195.2)	-5.4 (-9.2, -1.2)

Tunisia	52568 (45066, 60318)	1,129.5 (979.6, 1,291.3)	142335 (123094, 162365)	1,164.6 (1,007.2, 1,327.4)	3.1 (-1.8, 8.3)
Türkiye	445580 (383085, 513015)	1,340.1 (1,154.4, 1,538.9)	991808 (859414, 1135755)	1,149.4 (994.1, 1,313.7)	-14.2 (-17.9, -10.3)
United Arab Emirates	3907 (3295, 4505)	1,096.9 (945.8, 1,249.5)	38210 (31705, 44911)	1,062.5 (915.3, 1,220.1)	-3.1 (-7.5, 1.0)
Yemen	42544 (36495, 49002)	999.5 (868.6, 1,139.7)	121858 (104299, 139518)	1,023.0 (885.9, 1,164.3)	2.3 (-2.1, 7.2)
South Asia	4514895 (3871587, 5193630)	918.3 (797.3, 1,052.2)	12004464 (10387163, 13752790)	917.3 (797.7, 1,047.2)	-0.1 (-1.1, 0.8)
Bangladesh	358655 (308090, 411336)	845.4 (729.9, 970.9)	1022704 (874879, 1176458)	819.9 (708.8, 939.1)	-3.0 (-7.1, 1.2)
Bhutan	1870 (1614, 2144)	888.1 (772.2, 1,013.9)	4514 (3883, 5190)	853.5 (736.3, 971.8)	-3.9 (-8.1, 0.5)
India	3536023 (3022608, 4068053)	910.6 (790.8, 1,041.7)	9775720 (8457655, 11207856)	918.9 (797.5, 1,050.7)	0.9 (-0.1, 1.9)
Nepal	76461 (65594, 88725)	928.8 (804.1, 1,063.2)	187122 (160343, 216783)	899.0 (777.8, 1,033.4)	-3.2 (-7.1, 1.0)
Pakistan	541886 (468565, 620906)	1,017.0 (880.7, 1,157.1)	1014403 (871243, 1162602)	1,032.9 (893.3, 1,180.7)	1.6 (-1.2, 4.6)
Central Sub-Saharan Africa	208069 (177026, 240619)	1,080.5 (932.0, 1,242.3)	489866 (419830, 560169)	1,073.5 (924.1, 1,224.6)	-0.6 (-3.9, 2.8)
Angola	36524 (31250, 42522)	1,136.6 (978.3, 1,295.8)	114480 (97964, 132163)	1,209.7 (1,044.2, 1,381.0)	6.4 (1.4, 11.3)
Central African Republic	10564 (8981, 12268)	1,101.7 (948.7, 1,262.0)	19649 (16801, 22678)	1,116.2 (963.6, 1,283.6)	1.3 (-3.1, 6.8)
Congo	10981 (9351, 12745)	1,201.3 (1,032.9, 1,374.9)	25637 (21902, 29426)	1,132.7 (973.4, 1,301.4)	-5.7 (-9.8, -1.4)
Democratic Republic of the Congo	142255 (121309, 164664)	1,049.7 (903.5, 1,209.6)	314181 (268549, 358748)	1,021.9 (880.5, 1,163.8)	-2.7 (-7.1, 2.1)
Equatorial Guinea	1902 (1623, 2204)	1,119.3 (965.1, 1,288.4)	4956 (4256, 5633)	1,186.9 (1,025.7, 1,355.1)	6.0 (0.1, 11.9)
Gabon	5842 (5022, 6701)	1,164.4 (1,007.4, 1,328.3)	10962 (9409, 12517)	1,175.2 (1,014.8, 1,343.9)	0.9 (-3.3, 5.8)
Eastern Sub-Saharan Africa	596225 (511670, 687329)	916.9 (796.8, 1,053.1)	1349014 (1160033, 1547847)	939.1 (813.8, 1,076.6)	2.4 (1.2, 3.8)
Burundi	19736 (16854, 22755)	942.4 (815.0, 1,080.9)	36133 (30811, 41507)	918.5 (800.2, 1,050.9)	-2.5 (-7.5, 2.4)
Comoros	1924 (1648, 2226)	944.5 (816.1, 1,090.2)	4312 (3707, 4977)	945.2 (815.7, 1,088.5)	0.1 (-4.3, 4.5)
Djibouti	1099 (938, 1267)	957.0 (826.7, 1,096.2)	5025 (4293, 5787)	980.0 (846.8, 1,120.9)	2.4 (-1.8, 6.8)

Eritrea	7191 (6128, 8292)	915.2 (790.7, 1,048.5)	20254 (17286, 23339)	910.5 (787.3, 1,040.7)	-0.5 (-5.2, 4.2)
Ethiopia	135829 (115398, 157049)	817.7 (708.2, 939.6)	300425 (259009, 343902)	807.2 (699.6, 926.8)	-1.3 (-3.4, 0.9)
Kenya	71955 (62029, 82518)	977.8 (846.8, 1,116.4)	191374 (164495, 219133)	995.1 (860.7, 1,140.3)	1.8 (0.9, 2.6)
Madagascar	40917 (35129, 47048)	894.2 (773.1, 1,024.3)	85314 (72766, 98270)	915.9 (792.6, 1,053.2)	2.4 (-1.8, 6.8)
Malawi	35713 (30516, 41174)	1,061.2 (914.3, 1,213.5)	66357 (57109, 76108)	1,030.7 (891.5, 1,183.5)	-2.9 (-7.0, 1.3)
Mozambique	52393 (44940, 60563)	994.5 (858.9, 1,147.9)	101182 (86743, 117030)	1,058.9 (916.1, 1,215.9)	6.5 (1.8, 11.5)
Rwanda	25419 (21629, 29354)	1,011.4 (869.0, 1,164.3)	53743 (45884, 61367)	1,017.1 (875.3, 1,171.1)	0.6 (-3.6, 5.4)
Somalia	19369 (16535, 22283)	923.9 (804.0, 1,060.5)	53486 (45638, 61831)	974.1 (839.7, 1,114.9)	5.4 (0.7, 10.8)
South Sudan	18840 (16221, 21548)	889.4 (766.4, 1,013.4)	32862 (28268, 37333)	963.5 (835.2, 1,097.5)	8.3 (3.7, 12.8)
Uganda	51834 (44610, 59310)	899.6 (779.5, 1,028.4)	115864 (99377, 132329)	933.8 (804.9, 1,072.6)	3.8 (-0.7, 8.4)
United Republic of Tanzania	89553 (77028, 103356)	926.5 (801.6, 1,062.6)	225106 (194217, 258723)	1,015.9 (874.6, 1,162.2)	9.6 (5.0, 14.5)
Zambia	24015 (20620, 27709)	964.9 (831.7, 1,114.2)	56498 (48601, 64729)	974.6 (833.0, 1,115.7)	1.0 (-3.4, 5.6)
Southern Sub-Saharan Africa	379295 (327243, 433543)	1,511.3 (1,305.0, 1,732.9)	676050 (584849, 774097)	1,307.5 (1,135.5, 1,490.7)	-13.5 (-14.9, -12.0)
Botswana	6448 (5496, 7504)	1,287.3 (1,106.1, 1,483.1)	15483 (13314, 17814)	1,302.6 (1,127.5, 1,502.0)	1.2 (-3.6, 6.3)
Lesotho	10811 (9317, 12401)	1,213.6 (1,054.2, 1,380.1)	14799 (12632, 17020)	1,326.7 (1,145.7, 1,519.8)	9.3 (3.9, 14.8)
Namibia	8525 (7270, 9890)	1,292.8 (1,116.9, 1,481.1)	15871 (13705, 18148)	1,225.3 (1,061.5, 1,400.9)	-5.2 (-9.7, -0.5)
South Africa	307645 (265352, 351539)	1,599.1 (1,380.5, 1,830.2)	544572 (470638, 622398)	1,311.6 (1,139.3, 1,497.0)	-18.0 (-19.7, -16.3)
Eswatini	3418 (2944, 3924)	1,340.4 (1,154.9, 1,535.2)	6974 (5958, 8039)	1,381.0 (1,183.2, 1,590.2)	3.0 (-1.7, 7.9)
Zimbabwe	42447 (36442, 49337)	1,185.6 (1,022.2, 1,360.2)	78349 (67121, 90505)	1,279.2 (1,108.2, 1,477.1)	7.9 (2.2, 13.1)
Western Sub-Saharan Africa	678517 (583780, 781794)	865.5 (750.4, 995.6)	1516753 (1304684, 1737206)	902.7 (783.4, 1,036.4)	4.3 (3.5, 5.1)
Benin	17343 (14861, 19889)	941.1 (810.4, 1,073.1)	42077 (36280, 48496)	963.4 (839.0, 1,108.9)	2.4 (-2.4, 7.3)

Burkina Faso	33300 (28396, 38586)	851.4 (736.9, 977.6)	71422 (61561, 82320)	878.8 (762.0, 1,011.5)	3.2 (-1.1, 8.0)
Cameroon	33772 (28899, 38787)	859.0 (738.7, 989.1)	95183 (81652, 109434)	908.4 (784.1, 1,043.2)	5.8 (0.9, 10.6)
Cabo Verde	2240 (1917, 2582)	961.5 (826.5, 1,104.7)	4119 (3564, 4683)	1,003.1 (864.9, 1,152.1)	4.3 (0.0, 9.0)
Chad	21530 (18613, 24862)	825.3 (718.1, 945.5)	42710 (36883, 49007)	853.8 (740.3, 978.1)	3.4 (-0.8, 7.7)
Cote d'Ivoire	31819 (27141, 36675)	947.3 (818.2, 1,086.2)	89451 (76435, 102918)	972.3 (836.3, 1,116.3)	2.6 (-1.5, 7.4)
Gambia	3046 (2605, 3525)	986.0 (849.0, 1,136.1)	8778 (7576, 10074)	1,002.5 (867.2, 1,152.0)	1.7 (-2.8, 6.7)
Ghana	49921 (42933, 57720)	914.3 (791.3, 1,050.8)	136300 (117486, 156448)	950.8 (822.6, 1,098.0)	4.0 (-0.6, 8.6)
Guinea	25028 (21481, 28766)	818.0 (705.2, 940.5)	42833 (36863, 49342)	849.8 (734.7, 973.8)	3.9 (-0.6, 8.5)
Guinea-Bissau	3237 (2770, 3726)	921.1 (797.7, 1,055.7)	5743 (4933, 6600)	931.4 (804.6, 1,067.6)	1.1 (-3.3, 6.2)
Liberia	9801 (8418, 11299)	956.4 (829.9, 1,091.4)	17819 (15283, 20412)	972.9 (839.0, 1,115.6)	1.7 (-2.8, 6.6)
Mali	30769 (26310, 35366)	840.6 (724.7, 963.1)	67843 (58647, 77842)	879.2 (762.3, 1,003.9)	4.6 (-0.1, 9.4)
Mauritania	8173 (6989, 9540)	890.9 (771.3, 1,025.3)	17811 (15452, 20532)	920.1 (796.3, 1,051.7)	3.3 (-0.7, 7.5)
Niger	19640 (16760, 22727)	823.2 (711.0, 948.7)	58787 (50305, 67177)	861.0 (746.2, 974.6)	4.6 (0.3, 9.1)
Nigeria	331670 (285629, 381235)	840.0 (726.2, 964.5)	686878 (590520, 786555)	882.1 (764.1, 1,009.6)	5.0 (4.0, 6.0)
Sao Tome and Principe	558 (474, 649)	930.4 (799.6, 1,068.9)	915 (787, 1045)	960.1 (828.5, 1,104.4)	3.2 (-0.9, 7.7)
Senegal	27965 (24025, 32201)	958.9 (828.7, 1,100.6)	65157 (56177, 74821)	950.3 (822.0, 1,089.4)	-0.9 (-4.9, 3.6)
Sierra Leone	18573 (15875, 21425)	1,042.1 (895.1, 1,196.9)	32338 (27923, 37178)	999.0 (866.8, 1,153.8)	-4.1 (-8.3, 0.4)
Togo	10110 (8661, 11662)	931.0 (804.2, 1,073.6)	30567 (26184, 35256)	958.8 (829.1, 1,103.5)	3.0 (-1.7, 7.6)
High SDI	28471688 (24732858, 32414698)	2,716.6 (2,375.4, 3,078.4)	34291798 (30198669, 38329603)	1,794.0 (1,585.1, 2,006.1)	-34.0 (-35.8, -32.0)
High-middle SDI	18005646 (15658151, 20458991)	1,779.5 (1,554.7, 2,019.7)	30755470 (26773011, 34985543)	1,506.1 (1,314.6, 1,711.8)	-15.4 (-16.4, -14.2)
Middle SDI	11723338 (10082158, 13476984)	1,251.5 (1,088.6, 1,428.3)	30576763 (26400708, 35052196)	1,264.8 (1,098.3, 1,446.0)	1.1 (0.1, 2.0)

Low-middle SDI	5608666 (4819057, 6450687)	1,053.9 (915.0, 1,205.5)	13410114 (11591984, 15328969)	1,042.2 (905.4, 1,190.1)	-1.1 (-2.2, -0.1)
Low SDI	1924035 (1651238, 2208167)	933.9 (812.4, 1,073.0)	4354605 (3744362, 5006216)	938.6 (815.0, 1,074.6)	0.5 (-0.4, 1.5)

Table S2. DALYs due to PAD in 1990 and 2019 for both sexes and percentage change in age-standardised rates by location. PAD= Peripheral artery disease. DALYs=disability-adjusted life-years.

DALYs of PAD	1990		2019		Percentage change in age-standardised rates between 1990 and 2019
	Counts (95% UI)	Age-standardised rate (95% UI)	Counts (95% UI)	Age-standardised rate (95% UI)	
Global	775515 (487987, 1178138)	22.4 (14.1, 34.1)	1536381 (1006770, 2370813)	19.6 (12.9, 30.2)	-12.8 (-24.1, -4.6)
East Asia	70055 (39189, 114310)	9.6 (5.4, 16.0)	172068 (99343, 284632)	8.8 (5.1, 14.4)	-8.4 (-15.4, -0.3)
China	67569 (37810, 110283)	9.6 (5.4, 16.0)	165729 (96082, 274013)	8.8 (5.1, 14.4)	-8.5 (-15.8, -0.3)
Democratic People's Republic of Korea	1203 (671, 2016)	9.4 (5.2, 15.7)	2893 (1610, 4872)	9.6 (5.3, 16.1)	2.2 (-7.2, 12.4)
Taiwan	1284 (664, 2249)	9.3 (4.8, 15.8)	3447 (1865, 5814)	8.5 (4.6, 14.2)	-8.3 (-18.0, 4.3)
Southeast Asia	22595 (12667, 36780)	10.7 (6.0, 17.6)	59449 (36655, 92907)	11.1 (6.8, 17.7)	4.5 (-2.0, 14.6)
Cambodia	398 (215, 677)	10.9 (5.9, 18.6)	1073 (611, 1729)	10.5 (6.1, 16.8)	-3.8 (-14.3, 11.0)
Indonesia	8534 (4855, 13807)	11.0 (6.2, 18.1)	23087 (13699, 36346)	13.0 (7.8, 20.5)	18.7 (9.3, 34.7)
Lao People's Democratic Republic	205 (112, 341)	12.0 (6.7, 20.1)	425 (253, 671)	11.8 (7.0, 18.7)	-1.7 (-11.6, 14.2)
Malaysia	764 (421, 1260)	9.8 (5.4, 16.5)	2303 (1386, 3686)	9.7 (5.9, 15.3)	-1.2 (-10.9, 14.7)
Maldives	8 (5, 14)	11.8 (7.1, 19.5)	30 (20, 45)	12.3 (8.3, 18.7)	4.6 (-26.2, 36.1)
Mauritius	71 (39, 116)	11.1 (6.3, 18.5)	213 (124, 355)	12.7 (7.5, 21.4)	14.3 (-2.2, 39.1)
Myanmar	2730 (1539, 4460)	13.9 (7.8, 23.2)	5252 (3140, 8424)	12.9 (7.7, 20.8)	-7.3 (-17.1, 5.7)
Philippines	2451 (1396, 4035)	10.1 (5.7, 16.8)	7732 (4772, 12090)	11.6 (7.1, 18.5)	15.0 (7.7, 26.8)
Seychelles	7 (4, 11)	12.3 (7.8, 19.2)	13 (9, 20)	13.0 (8.5, 20.0)	6.0 (-5.6, 24.7)
Sri Lanka	837 (464, 1379)	9.0 (5.0, 14.8)	2301 (1373, 3787)	9.4 (5.6, 15.3)	4.3 (-8.7, 21.5)
Thailand	2610 (1356, 4545)	8.9 (4.7, 15.6)	7354 (4069, 12530)	7.4 (4.1, 12.4)	-17.7 (-24.8, -8.8)
Timor-Leste	20 (11, 34)	10.2 (5.5, 17.0)	80 (45, 132)	10.9 (6.3, 17.7)	7.1 (-4.4, 26.0)

Viet Nam	3930<br>(2272, 6278)	10.6<br>(6.2, 16.9)	9509<br>(5910, 14585)	11.6<br>(7.2, 17.9)	9.2<br>(-5.0, 28.0)
Oceania	236<br>(134, 375)	10.3<br>(5.8, 16.6)	639<br>(388, 994)	11.7<br>(7.0, 18.5)	13.8<br>(5.9, 26.1)
American Samoa	2<br>(1, 3)	12.3<br>(7.6, 19.2)	6<br>(4, 9)	14.7<br>(9.8, 21.8)	19.9<br>(6.7, 40.1)
Cook Islands	1<br>(1, 2)	11.8<br>(7.5, 17.9)	4<br>(2, 5)	14.5<br>(9.7, 21.3)	22.8<br>(1.7, 52.4)
Fiji	31<br>(18, 51)	11.5<br>(6.5, 19.0)	80<br>(48, 128)	13.2<br>(7.9, 20.9)	14.8<br>(2.4, 33.1)
Guam	5<br>(3, 9)	9.2<br>(5.6, 14.6)	21<br>(14, 32)	11.4<br>(7.3, 17.5)	23.6<br>(7.1, 45.1)
Kiribati	4<br>(2, 6)	13.2<br>(7.6, 21.3)	8<br>(5, 13)	15.8<br>(8.9, 26.3)	19.9<br>(7.6, 32.6)
Marshall Islands	2<br>(1, 3)	12.2<br>(7.6, 19.2)	4<br>(2, 6)	14.1<br>(9.0, 21.0)	15.7<br>(0.9, 36.5)
Micronesia (Federated States of)	5<br>(3, 7)	12.2<br>(7.2, 19.2)	8<br>(5, 12)	14.8<br>(9.7, 22.1)	21.2<br>(2.6, 46.5)
Nauru	0<br>(0, 1)	13.2<br>(8.5, 19.6)	0<br>(0, 1)	14.6<br>(9.6, 22.0)	10.5<br>(-4.3, 27.6)
Niue	0<br>(0, 0)	11.8<br>(7.3, 18.0)	0<br>(0, 0)	14.1<br>(9.6, 20.8)	19.1<br>(3.4, 42.0)
Northern Mariana Islands	1<br>(1, 2)	11.5<br>(7.6, 17.5)	6<br>(4, 9)	14.6<br>(10.2, 20.8)	27.1<br>(6.9, 53.7)
Palau	1<br>(0, 1)	8.7<br>(4.6, 14.4)	2<br>(1, 3)	9.2<br>(5.0, 15.7)	6.4<br>(-4.0, 18.2)
Papua New Guinea	136<br>(76, 219)	9.6<br>(5.2, 15.9)	390<br>(230, 612)	10.9<br>(6.4, 17.5)	14.0<br>(3.7, 29.0)
Samoa	10<br>(6, 15)	12.9<br>(7.6, 20.1)	19<br>(12, 29)	14.6<br>(9.2, 22.4)	12.8<br>(-0.1, 30.2)
Solomon Islands	11<br>(6, 18)	10.6<br>(6.1, 17.0)	29<br>(17, 44)	12.2<br>(7.2, 19.0)	15.5<br>(2.6, 31.9)
Tokelau	0<br>(0, 0)	11.0<br>(6.2, 17.4)	0<br>(0, 0)	12.7<br>(8.1, 19.4)	16.1<br>(2.7, 37.0)
Tonga	6<br>(3, 9)	11.8<br>(7.0, 18.8)	11<br>(7, 16)	13.9<br>(8.8, 21.3)	18.4<br>(6.2, 37.6)
Tuvalu	1<br>(0, 1)	11.7<br>(6.8, 18.8)	1<br>(1, 2)	13.2<br>(8.2, 20.2)	12.6<br>(-2.1, 32.3)
Vanuatu	6<br>(3, 9)	10.5<br>(5.8, 17.5)	19<br>(12, 31)	13.1<br>(8.0, 20.7)	25.0<br>(11.3, 46.9)
Central Asia	3381<br>(1885, 5429)	8.1<br>(4.5, 13.1)	5318<br>(3113, 8612)	8.8<br>(5.2, 14.4)	8.7<br>(-1.7, 26.2)

Armenia	260<br>(131, 476)	11.2<br>(5.6, 20.5)	445<br>(268, 723)	10.8<br>(6.5, 17.5)	-3.8<br>(-45.8, 30.9)
Azerbaijan	329<br>(169, 559)	7.6<br>(4.0, 13.1)	521<br>(284, 885)	7.3<br>(4.1, 12.2)	-4.2<br>(-12.2, 7.9)
Georgia	454<br>(253, 747)	7.9<br>(4.4, 13.0)	597<br>(339, 1000)	9.4<br>(5.3, 15.8)	17.8<br>(3.4, 76.4)
Kazakhstan	930<br>(509, 1546)	8.3<br>(4.6, 13.8)	1277<br>(736, 2117)	8.4<br>(4.8, 14.0)	0.7<br>(-11.1, 18.9)
Kyrgyzstan	339<br>(195, 543)	11.8<br>(6.8, 18.9)	544<br>(310, 934)	13.5<br>(7.7, 22.7)	14.7<br>(-12.2, 40.3)
Mongolia	70<br>(37, 120)	7.9<br>(4.2, 13.4)	121<br>(66, 206)	7.3<br>(4.1, 12.2)	-7.6<br>(-15.4, 1.1)
Tajikistan	205<br>(106, 348)	7.9<br>(4.1, 13.4)	247<br>(136, 411)	7.2<br>(4.0, 11.8)	-9.1<br>(-17.1, 1.6)
Turkmenistan	143<br>(81, 230)	8.7<br>(5.0, 13.9)	338<br>(196, 554)	10.1<br>(5.8, 16.7)	15.0<br>(-4.0, 46.0)
Uzbekistan	653<br>(354, 1060)	6.5<br>(3.5, 10.7)	1227<br>(699, 2062)	8.3<br>(4.8, 13.8)	28.3<br>(6.9, 64.2)
Central Europe	42359<br>(23792, 72069)	29.7<br>(16.9, 50.3)	73010<br>(40613, 127696)	32.9<br>(18.3, 57.9)	10.8<br>(-17.2, 40.4)
Albania	145<br>(84, 238)	8.0<br>(4.7, 13.1)	364<br>(237, 561)	8.3<br>(5.4, 12.7)	3.0<br>(-10.6, 26.6)
Bosnia and Herzegovina	366<br>(235, 544)	10.5<br>(6.8, 15.8)	792<br>(554, 1115)	13.1<br>(9.2, 18.5)	24.0<br>(6.1, 53.3)
Bulgaria	1767<br>(992, 2977)	14.8<br>(8.3, 24.7)	2333<br>(1407, 3986)	15.4<br>(9.2, 26.4)	4.0<br>(-14.2, 31.9)
Croatia	2411<br>(1222, 4161)	39.5<br>(19.9, 67.8)	3625<br>(1815, 6697)	39.4<br>(19.9, 74.4)	-0.4<br>(-27.4, 31.5)
Czechia	4920<br>(2663, 8942)	36.0<br>(19.4, 65.1)	8036<br>(4319, 15270)	36.9<br>(19.6, 71.0)	2.7<br>(-30.4, 41.0)
Hungary	11989<br>(5853, 21370)	82.3<br>(40.0, 146.7)	16823<br>(7619, 34636)	84.5<br>(38.9, 175.1)	2.6<br>(-56.8, 41.0)
North Macedonia	182<br>(119, 273)	11.0<br>(7.3, 16.5)	306<br>(201, 459)	10.1<br>(6.8, 15.0)	-8.2<br>(-19.7, 8.5)
Montenegro	64<br>(41, 99)	10.9<br>(7.0, 16.9)	109<br>(75, 164)	11.0<br>(7.6, 16.5)	1.1<br>(-9.9, 18.2)
Poland	9588<br>(4100, 16942)	22.5<br>(9.7, 39.4)	20479<br>(9832, 41639)	28.0<br>(13.5, 57.1)	24.6<br>(-25.5, 458.3)
Romania	8521<br>(4392, 15873)	31.9<br>(16.7, 59.0)	14302<br>(7047, 26634)	37.2<br>(18.2, 69.6)	16.6<br>(-12.1, 46.7)
Serbia	1211<br>(813, 1798)	11.5<br>(7.7, 17.3)	3127<br>(2259, 4097)	19.4<br>(14.2, 25.3)	69.0<br>(23.3, 132.9)

Slovakia	652<br>(433, 978)	11.1<br>(7.3, 16.7)	1978<br>(1363, 2628)	21.2<br>(14.6, 28.1)	91.4<br>(22.3, 182.8)
Slovenia	541<br>(241, 1337)	22.3<br>(9.9, 54.5)	737<br>(249, 1404)	16.1<br>(5.2, 30.4)	-28.1<br>(-90.1, 34.7)
Eastern Europe	135865<br>(71020, 234344)	50.5<br>(26.4, 86.7)	225458<br>(118281, 415622)	63.6<br>(33.5, 117.8)	25.9<br>(-9.8, 54.4)
Belarus	2137<br>(1170, 3532)	16.7<br>(9.1, 27.5)	3706<br>(1905, 7160)	22.6<br>(11.7, 43.8)	35.0<br>(-0.3, 84.5)
Estonia	186<br>(99, 311)	9.1<br>(4.9, 15.2)	275<br>(159, 457)	9.4<br>(5.4, 15.7)	3.2<br>(-14.1, 50.9)
Latvia	365<br>(202, 587)	10.2<br>(5.7, 16.3)	679<br>(327, 1612)	15.5<br>(7.5, 36.9)	52.6<br>(0.2, 411.9)
Lithuania	885<br>(480, 1539)	19.5<br>(10.6, 33.9)	1655<br>(860, 2865)	26.8<br>(13.7, 46.7)	37.2<br>(0.8, 207.1)
Republic of Moldova	462<br>(240, 763)	11.5<br>(6.1, 18.7)	916<br>(481, 1789)	15.6<br>(8.2, 30.5)	35.1<br>(-3.6, 310.7)
Russian Federation	94119<br>(48349, 159960)	54.8<br>(28.1, 92.9)	165615<br>(85433, 313908)	68.8<br>(35.5, 130.0)	25.6<br>(-23.9, 64.2)
Ukraine	37711<br>(19957, 68534)	54.1<br>(28.6, 97.3)	52612<br>(26807, 95005)	67.0<br>(34.3, 121.5)	23.9<br>(0.3, 52.0)
High-income Asia Pacific	23176<br>(13326, 37871)	12.2<br>(7.0, 19.7)	38819<br>(23441, 63182)	7.5<br>(4.5, 12.0)	-38.4<br>(-45.3, -27.7)
Brunei Darussalam	16<br>(11, 22)	23.1<br>(16.4, 32.4)	41<br>(31, 55)	21.1<br>(16.5, 27.6)	-8.8<br>(-27.5, 14.0)
Japan	19502<br>(10787, 32447)	11.8<br>(6.6, 19.4)	31458<br>(18262, 51617)	7.5<br>(4.4, 12.1)	-36.8<br>(-44.5, -25.3)
Republic of Korea	3440<br>(2324, 5168)	14.3<br>(9.8, 21.3)	6610<br>(4469, 10077)	7.5<br>(5.1, 11.4)	-47.4<br>(-54.7, -40.1)
Singapore	218<br>(124, 354)	11.8<br>(6.9, 19.4)	710<br>(382, 1271)	9.6<br>(5.1, 17.1)	-19.1<br>(-45.6, 70.6)
Australasia	7686<br>(4185, 12366)	33.6<br>(18.2, 54.3)	18175<br>(9921, 32779)	32.9<br>(17.9, 59.7)	-2.1<br>(-44.8, 24.4)
Australia	6499<br>(3516, 10423)	34.3<br>(18.5, 55.4)	15659<br>(8479, 28277)	33.6<br>(18.3, 60.9)	-2.0<br>(-45.6, 25.1)
New Zealand	1187<br>(660, 1883)	30.6<br>(17.0, 48.8)	2515<br>(1380, 4538)	29.2<br>(15.9, 52.6)	-4.4<br>(-41.6, 21.2)
Western Europe	208197<br>(124357, 331165)	34.6<br>(20.6, 55.2)	323162<br>(181925, 546615)	31.2<br>(17.7, 53.8)	-9.7<br>(-29.6, 6.2)
Andorra	11<br>(7, 17)	22.2<br>(15.1, 33.6)	43<br>(28, 60)	29.1<br>(19.0, 40.6)	31.4<br>(-7.9, 89.3)
Austria	5740<br>(3239, 9389)	46.3<br>(26.1, 76.1)	9902<br>(5299, 17974)	49.5<br>(26.5, 90.9)	6.8<br>(-24.1, 36.1)

Belgium	3932<br>(2345, 6235)	24.6<br>(14.7, 38.6)	5741<br>(3444, 9379)	22.4<br>(13.5, 37.0)	-9.1<br>(-25.9, 9.8)
Cyprus	175<br>(114, 264)	24.1<br>(15.5, 34.9)	408<br>(290, 558)	22.3<br>(15.3, 30.3)	-7.5<br>(-25.8, 17.9)
Denmark	4215<br>(2447, 6603)	48.7<br>(28.1, 76.3)	5308<br>(3098, 9056)	42.1<br>(24.6, 71.9)	-13.6<br>(-40.3, 8.7)
Finland	1863<br>(1032, 2915)	25.7<br>(14.3, 40.2)	3219<br>(1755, 5390)	23.1<br>(12.7, 38.2)	-10.4<br>(-30.1, 11.3)
France	17621<br>(10435, 28469)	20.1<br>(11.9, 32.6)	24062<br>(14640, 38960)	15.4<br>(9.4, 25.1)	-23.2<br>(-35.8, -5.2)
Germany	65500<br>(35046, 113289)	49.6<br>(26.7, 85.9)	106426<br>(57327, 187259)	49.3<br>(26.7, 86.9)	-0.7<br>(-28.3, 62.2)
Greece	2531<br>(1420, 4158)	16.4<br>(9.3, 26.9)	3406<br>(2034, 5493)	12.6<br>(7.5, 20.6)	-23.2<br>(-31.1, -11.4)
Iceland	56<br>(33, 92)	18.6<br>(11.1, 30.8)	85<br>(52, 134)	13.9<br>(8.4, 22.1)	-25.3<br>(-37.2, -8.5)
Ireland	2115<br>(1183, 3377)	52.0<br>(29.4, 83.0)	3613<br>(1944, 6658)	46.4<br>(25.0, 85.7)	-10.8<br>(-53.3, 17.9)
Israel	1889<br>(1076, 3128)	39.8<br>(22.8, 66.0)	4936<br>(2783, 8468)	40.1<br>(22.5, 68.1)	0.6<br>(-22.6, 25.8)
Italy	36171<br>(21003, 58266)	39.9<br>(23.1, 63.9)	52219<br>(29401, 92033)	31.3<br>(17.5, 55.9)	-21.6<br>(-49.4, -2.4)
Luxembourg	142<br>(86, 225)	25.7<br>(15.5, 40.4)	234<br>(135, 406)	21.9<br>(12.6, 37.5)	-14.8<br>(-36.8, 8.3)
Malta	157<br>(90, 249)	38.3<br>(22.3, 61.7)	359<br>(204, 619)	35.9<br>(20.4, 62.8)	-6.1<br>(-28.7, 16.3)
Monaco	16<br>(11, 24)	20.4<br>(13.4, 30.4)	18<br>(13, 26)	17.1<br>(12.3, 24.1)	-16.4<br>(-28.5, 1.6)
Netherlands	9895<br>(5699, 16363)	48.3<br>(27.7, 79.7)	17008<br>(9449, 29803)	46.1<br>(25.7, 81.0)	-4.4<br>(-21.3, 10.2)
Norway	2195<br>(1240, 3478)	29.5<br>(16.7, 47.0)	2633<br>(1504, 4420)	25.1<br>(14.4, 42.5)	-15.2<br>(-47.8, 6.4)
Portugal	5668<br>(3160, 8981)	42.1<br>(23.3, 67.1)	10791<br>(5860, 19150)	39.2<br>(21.4, 69.9)	-6.9<br>(-32.3, 20.3)
Spain	16164<br>(9516, 25912)	29.2<br>(17.2, 46.9)	28049<br>(16056, 47328)	25.5<br>(14.5, 43.5)	-12.8<br>(-28.9, 8.6)
Sweden	4024<br>(2253, 6690)	24.2<br>(13.7, 39.4)	4994<br>(2970, 8062)	20.7<br>(12.4, 33.2)	-14.5<br>(-43.5, 8.4)
Switzerland	3165<br>(1792, 5054)	28.6<br>(16.3, 45.2)	4793<br>(2803, 7990)	24.4<br>(14.4, 41.1)	-14.6<br>(-37.9, 4.7)
United Kingdom	24772<br>(13782, 39778)	25.9<br>(14.4, 41.7)	34619<br>(18542, 57201)	24.8<br>(13.3, 40.7)	-4.5<br>(-31.8, 103.7)

Southern Latin America	6923 (3998, 11227)	15.7 (9.1, 25.3)	13172 (7928, 21541)	15.4 (9.3, 25.2)	-1.8 (-20.4, 24.5)
Argentina	4632 (2663, 7536)	14.9 (8.6, 24.3)	7247 (4386, 11803)	13.0 (7.9, 21.1)	-12.3 (-25.0, 5.4)
Chile	1807 (1083, 2902)	20.0 (12.0, 32.1)	5412 (3073, 9147)	22.4 (12.7, 37.9)	11.8 (-16.3, 51.2)
Uruguay	483 (246, 840)	12.1 (6.2, 20.9)	512 (277, 879)	8.7 (4.7, 14.8)	-27.5 (-36.1, -17.7)
High-income North America	131239 (77560, 208887)	36.2 (21.4, 58.3)	260131 (146497, 452563)	39.2 (22.0, 68.2)	8.4 (-9.0, 24.6)
Canada	11966 (7021, 18840)	37.0 (21.6, 58.2)	24069 (13970, 41481)	33.1 (19.3, 56.7)	-10.4 (-36.1, 11.1)
United States of America	119254 (70689, 191107)	36.1 (21.4, 58.8)	236031 (132468, 410580)	40.0 (22.3, 69.5)	10.7 (-7.3, 27.1)
Greenland	16 (13, 21)	61.7 (48.6, 79.6)	27 (21, 36)	44.3 (35.4, 58.5)	-28.3 (-42.5, -10.1)
Caribbean	7120 (4175, 12068)	29.0 (17.0, 49.2)	17034 (9848, 28092)	32.8 (19.0, 54.1)	13.0 (-17.3, 34.8)
Antigua and Barbuda	16 (9, 29)	29.5 (16.0, 51.7)	35 (19, 64)	37.8 (19.9, 67.6)	28.1 (-2.5, 69.4)
Bahamas	53 (28, 93)	38.9 (21.3, 67.3)	158 (83, 282)	44.5 (23.8, 79.1)	14.4 (-14.4, 41.3)
Barbados	257 (134, 465)	85.1 (44.2, 153.9)	507 (256, 904)	102.1 (51.7, 182.2)	19.9 (-13.1, 50.9)
Belize	10 (6, 16)	11.2 (6.4, 18.1)	33 (20, 55)	13.5 (8.1, 22.3)	20.7 (-7.2, 55.3)
Bermuda	32 (16, 57)	55.0 (27.8, 99.9)	71 (38, 128)	51.7 (27.2, 93.4)	-5.9 (-39.4, 35.7)
Cuba	3852 (2039, 7165)	38.2 (20.2, 70.8)	9100 (4606, 16502)	46.2 (23.2, 83.3)	21.1 (-18.4, 61.4)
Dominica	18 (14, 23)	24.3 (19.0, 30.9)	28 (23, 35)	30.8 (24.7, 38.8)	26.6 (1.1, 59.1)
Dominican Republic	278 (174, 431)	8.7 (5.5, 13.3)	851 (580, 1219)	9.9 (6.7, 14.1)	13.8 (-2.8, 40.7)
Grenada	27 (15, 49)	34.9 (18.7, 63.3)	49 (26, 87)	48.4 (26.2, 85.2)	38.6 (-8.6, 77.3)
Guyana	54 (29, 91)	16.6 (9.2, 27.6)	116 (65, 205)	21.3 (12.0, 37.9)	28.3 (-7.2, 79.2)
Haiti	636 (438, 958)	24.0 (16.4, 35.5)	1374 (924, 2137)	23.7 (16.2, 36.2)	-1.5 (-26.8, 29.7)
Jamaica	506 (243, 985)	27.9 (13.4, 54.4)	1178 (594, 2236)	38.2 (19.2, 74.0)	37.0 (-17.3, 94.5)

Puerto Rico	899<br>(487, 1516)	25.3<br>(13.7, 42.7)	2324<br>(1254, 4378)	29.7<br>(15.9, 56.3)	17.2<br>(-18.6, 54.0)
Saint Kitts and Nevis	22<br>(11, 41)	61.1<br>(30.8, 115.1)	41<br>(20, 76)	74.7<br>(38.2, 138.8)	22.2<br>(-26.0, 62.9)
Saint Lucia	41<br>(22, 73)	50.4<br>(27.2, 91.2)	119<br>(63, 217)	57.9<br>(30.6, 104.8)	14.8<br>(-26.0, 43.7)
Saint Vincent and the Grenadines	16<br>(9, 27)	23.6<br>(12.6, 39.2)	39<br>(21, 68)	30.1<br>(16.8, 52.5)	27.5<br>(-10.8, 69.2)
Suriname	20<br>(13, 31)	8.4<br>(5.3, 13.0)	51<br>(34, 76)	9.0<br>(6.0, 13.6)	6.7<br>(-4.6, 23.3)
Trinidad and Tobago	115<br>(62, 192)	15.1<br>(8.0, 25.5)	282<br>(158, 497)	15.6<br>(8.7, 27.2)	3.1<br>(-27.2, 39.0)
United States Virgin Islands	30<br>(23, 40)	40.6<br>(31.9, 54.1)	100<br>(79, 131)	56.1<br>(44.6, 74.0)	38.2<br>(8.3, 76.2)
Andean Latin America	1161<br>(683, 1847)	6.5<br>(3.8, 10.3)	3138<br>(1983, 4919)	5.9<br>(3.7, 9.2)	-9.5<br>(-16.3, 1.7)
Bolivia (Plurinational State of)	203<br>(117, 324)	7.6<br>(4.4, 12.1)	556<br>(344, 853)	7.2<br>(4.5, 11.0)	-5.5<br>(-17.5, 12.8)
Ecuador	301<br>(170, 503)	6.4<br>(3.6, 10.6)	879<br>(547, 1369)	6.3<br>(3.9, 9.7)	-1.5<br>(-12.4, 16.0)
Peru	657<br>(396, 1029)	6.2<br>(3.7, 9.7)	1704<br>(1058, 2735)	5.4<br>(3.3, 8.7)	-13.5<br>(-23.3, -0.2)
Central Latin America	10065<br>(6060, 15887)	14.1<br>(8.5, 22.3)	28959<br>(17665, 48099)	12.9<br>(7.9, 21.4)	-8.5<br>(-32.1, 17.7)
Colombia	1951<br>(1146, 3102)	13.1<br>(7.8, 20.9)	6340<br>(3694, 11011)	11.8<br>(6.9, 20.2)	-10.1<br>(-37.3, 24.9)
Costa Rica	153<br>(90, 243)	9.5<br>(5.6, 15.0)	488<br>(296, 827)	9.6<br>(5.8, 16.2)	1.6<br>(-17.5, 27.7)
El Salvador	182<br>(100, 302)	6.6<br>(3.7, 11.1)	368<br>(216, 601)	6.0<br>(3.5, 9.8)	-9.9<br>(-18.3, 2.1)
Guatemala	200<br>(104, 337)	6.9<br>(3.6, 11.4)	654<br>(363, 1109)	6.4<br>(3.6, 11.0)	-6.8<br>(-16.5, 8.1)
Honduras	198<br>(121, 300)	11.3<br>(6.8, 17.2)	585<br>(385, 847)	11.4<br>(7.5, 16.4)	0.5<br>(-15.8, 21.6)
Mexico	6013<br>(3516, 9513)	16.5<br>(9.7, 25.9)	16410<br>(9548, 27791)	15.1<br>(8.8, 25.6)	-8.5<br>(-35.3, 20.7)
Nicaragua	98<br>(55, 163)	7.5<br>(4.2, 12.5)	272<br>(170, 429)	7.3<br>(4.6, 11.3)	-3.5<br>(-13.6, 12.4)
Panama	226<br>(124, 377)	16.3<br>(9.0, 27.2)	782<br>(445, 1409)	18.6<br>(10.6, 33.6)	14.1<br>(-28.9, 55.7)
Venezuela (Bolivarian Republic of)	1044<br>(623, 1695)	12.5<br>(7.5, 20.0)	3061<br>(1855, 4976)	11.2<br>(6.8, 18.3)	-10.3<br>(-28.2, 16.2)

Tropical Latin America	18664<br>(10265, 30667)	23.3<br>(12.9, 38.0)	54737<br>(29708, 93528)	23.4<br>(12.7, 39.8)	0.2<br>(-21.9, 26.4)
Brazil	18487<br>(10124, 30427)	23.7<br>(13.1, 38.6)	54103<br>(29179, 92662)	23.6<br>(12.8, 40.4)	-0.5<br>(-23.0, 25.8)
Paraguay	176<br>(111, 272)	8.7<br>(5.5, 13.5)	633<br>(447, 883)	12.1<br>(8.5, 17.0)	39.1<br>(11.0, 86.8)
North Africa and Middle East	16151<br>(10810, 23296)	11.2<br>(7.6, 16.5)	41136<br>(30016, 58107)	11.2<br>(8.2, 15.7)	-0.2<br>(-13.8, 17.1)
Afghanistan	431<br>(232, 716)	7.1<br>(3.9, 11.7)	708<br>(414, 1098)	7.9<br>(4.6, 12.4)	12.2<br>(0.7, 28.9)
Algeria	873<br>(512, 1403)	8.8<br>(5.3, 13.6)	2704<br>(1690, 4064)	9.6<br>(6.0, 14.4)	8.7<br>(-6.5, 32.4)
Bahrain	33<br>(25, 42)	25.0<br>(19.2, 32.0)	178<br>(131, 231)	26.2<br>(20.0, 33.6)	4.9<br>(-22.6, 39.5)
Egypt	1647<br>(903, 2715)	7.1<br>(4.0, 11.5)	4072<br>(2549, 6319)	8.1<br>(5.1, 12.5)	14.8<br>(1.6, 40.5)
Iran (Islamic Republic of)	1391<br>(781, 2290)	7.1<br>(4.1, 11.4)	5932<br>(4078, 8689)	9.2<br>(6.3, 13.6)	30.3<br>(9.2, 64.0)
Iraq	625<br>(354, 1035)	9.1<br>(5.1, 15.2)	1487<br>(921, 2380)	7.8<br>(4.7, 12.5)	-14.3<br>(-23.3, -1.4)
Jordan	76<br>(41, 127)	7.6<br>(4.2, 12.7)	348<br>(196, 578)	6.8<br>(3.9, 11.3)	-10.2<br>(-17.9, -0.8)
Kuwait	26<br>(14, 44)	6.2<br>(3.3, 10.5)	120<br>(67, 196)	6.3<br>(3.5, 10.3)	0.9<br>(-10.2, 19.8)
Lebanon	168<br>(97, 277)	8.7<br>(5.1, 14.1)	472<br>(305, 721)	9.1<br>(5.9, 14.0)	5.7<br>(-9.0, 29.6)
Libya	107<br>(60, 178)	6.8<br>(3.8, 11.3)	321<br>(193, 498)	7.6<br>(4.5, 11.8)	11.6<br>(-2.1, 35.2)
Morocco	862<br>(469, 1464)	7.6<br>(4.2, 12.5)	2233<br>(1428, 3382)	8.5<br>(5.5, 12.8)	12.4<br>(-2.8, 38.8)
Palestine	50<br>(27, 84)	6.5<br>(3.6, 11.1)	117<br>(67, 192)	6.3<br>(3.6, 10.3)	-2.5<br>(-11.5, 8.2)
Oman	39<br>(24, 61)	8.3<br>(5.0, 12.8)	123<br>(81, 179)	10.9<br>(7.2, 15.6)	32.2<br>(7.4, 75.5)
Qatar	7<br>(5, 11)	13.5<br>(8.2, 19.7)	47<br>(30, 69)	17.6<br>(11.7, 24.2)	30.7<br>(-2.0, 85.1)
Saudi Arabia	270<br>(151, 446)	6.0<br>(3.3, 9.9)	631<br>(367, 1026)	5.6<br>(3.3, 9.0)	-7.1<br>(-16.2, 7.6)
Sudan	502<br>(284, 828)	6.4<br>(3.6, 10.3)	1130<br>(707, 1738)	7.4<br>(4.7, 11.5)	16.5<br>(0.9, 40.8)
Syrian Arab Republic	298<br>(159, 507)	6.8<br>(3.7, 11.5)	602<br>(330, 1015)	5.9<br>(3.3, 9.8)	-13.5<br>(-20.5, -2.9)

Tunisia	316<br>(176, 530)	7.4<br>(4.2, 12.1)	949<br>(607, 1454)	8.2<br>(5.3, 12.5)	10.9<br>(-4.3, 37.1)
Türkiye	8130<br>(6162, 10632)	24.8<br>(18.7, 32.7)	17879<br>(13628, 24347)	21.0<br>(16.0, 28.8)	-15.4<br>(-36.9, 10.2)
United Arab Emirates	22<br>(13, 34)	9.0<br>(5.5, 14.6)	238<br>(140, 388)	10.4<br>(6.0, 16.5)	14.6<br>(-10.4, 61.4)
Yemen	268<br>(148, 449)	7.1<br>(3.9, 11.8)	804<br>(470, 1257)	7.6<br>(4.4, 11.9)	7.8<br>(-4.6, 27.1)
South Asia	33065<br>(20730, 50339)	7.8<br>(4.9, 12.0)	107763<br>(74349, 154410)	8.9<br>(6.2, 12.9)	14.1<br>(-2.9, 35.9)
Bangladesh	2818<br>(1676, 4388)	7.3<br>(4.4, 11.3)	10295<br>(6275, 15281)	8.9<br>(5.4, 13.2)	22.0<br>(-5.0, 61.0)
Bhutan	14<br>(9, 23)	7.8<br>(4.8, 12.4)	50<br>(32, 74)	10.2<br>(6.5, 14.9)	29.9<br>(-0.8, 76.7)
India	25077<br>(15541, 38290)	7.6<br>(4.7, 11.8)	86161<br>(59690, 124696)	8.7<br>(6.0, 12.6)	14.4<br>(-4.2, 36.5)
Nepal	548<br>(315, 874)	7.5<br>(4.4, 11.9)	1741<br>(1112, 2565)	9.1<br>(5.9, 13.4)	20.6<br>(-0.6, 56.4)
Pakistan	4608<br>(2922, 7100)	9.2<br>(5.9, 14.1)	9517<br>(6426, 14000)	11.1<br>(7.4, 16.4)	20.1<br>(2.1, 46.0)
Central Sub-Saharan Africa	8608<br>(3597, 15128)	46.1<br>(19.9, 80.4)	19070<br>(10672, 28794)	43.1<br>(24.0, 65.5)	-6.5<br>(-32.1, 39.5)
Angola	803<br>(500, 1145)	25.8<br>(15.5, 36.4)	2989<br>(1808, 4034)	33.6<br>(20.2, 45.1)	30.4<br>(-2.7, 78.9)
Central African Republic	276<br>(156, 418)	28.8<br>(15.7, 42.4)	500<br>(320, 737)	29.3<br>(17.8, 42.2)	1.6<br>(-21.8, 30.1)
Congo	378<br>(233, 518)	41.6<br>(26.0, 56.9)	1024<br>(675, 1413)	47.9<br>(31.6, 65.7)	15.3<br>(-16.5, 56.8)
Democratic Republic of the Congo	6889<br>(2453, 12930)	53.3<br>(19.6, 98.6)	13839<br>(6686, 23222)	45.9<br>(22.5, 75.5)	-14.0<br>(-39.8, 34.2)
Equatorial Guinea	47<br>(25, 69)	27.9<br>(15.0, 39.7)	194<br>(95, 327)	50.0<br>(24.9, 81.5)	79.0<br>(9.1, 188.3)
Gabon	214<br>(108, 344)	41.7<br>(20.8, 66.3)	523<br>(330, 727)	57.2<br>(35.8, 79.5)	37.1<br>(-2.9, 98.7)
Eastern Sub-Saharan Africa	13643<br>(8261, 19151)	22.0<br>(12.9, 31.0)	37019<br>(22579, 48292)	27.6<br>(16.3, 36.3)	25.5<br>(0.4, 52.7)
Burundi	496<br>(291, 722)	24.2<br>(14.0, 35.6)	823<br>(510, 1166)	22.5<br>(13.4, 31.8)	-6.8<br>(-35.1, 27.7)
Comoros	46<br>(25, 68)	23.4<br>(12.6, 33.9)	110<br>(60, 156)	25.2<br>(13.6, 36.0)	7.8<br>(-22.3, 53.3)
Djibouti	28<br>(15, 43)	25.9<br>(13.8, 39.7)	153<br>(81, 228)	32.6<br>(17.2, 47.4)	26.1<br>(-8.2, 72.4)

Eritrea	146<br>(88, 231)	19.2<br>(10.9, 30.0)	497<br>(284, 718)	24.2<br>(13.0, 35.5)	26.1<br>(-7.9, 79.8)
Ethiopia	3249<br>(2047, 4756)	20.3<br>(12.3, 30.1)	8700<br>(5276, 12303)	25.1<br>(15.0, 35.3)	23.8<br>(-17.7, 73.9)
Kenya	1272<br>(781, 1797)	18.5<br>(11.1, 26.2)	4477<br>(2768, 5905)	25.6<br>(14.9, 34.1)	38.6<br>(13.2, 70.5)
Madagascar	1208<br>(703, 1729)	27.0<br>(15.5, 38.5)	2862<br>(1600, 4164)	31.9<br>(17.4, 45.6)	18.3<br>(-15.0, 62.0)
Malawi	615<br>(351, 894)	19.7<br>(10.9, 28.8)	1468<br>(850, 2028)	24.1<br>(13.6, 33.2)	22.2<br>(-5.1, 58.5)
Mozambique	1083<br>(574, 1545)	22.0<br>(11.6, 31.1)	3060<br>(1801, 4242)	33.5<br>(19.0, 46.2)	52.4<br>(16.2, 101.5)
Rwanda	563<br>(314, 911)	23.1<br>(12.6, 38.0)	1156<br>(609, 1695)	23.5<br>(12.2, 34.7)	1.6<br>(-30.3, 37.7)
Somalia	379<br>(213, 602)	19.2<br>(10.6, 30.1)	951<br>(499, 1493)	18.6<br>(9.6, 28.7)	-3.2<br>(-26.6, 25.1)
South Sudan	553<br>(303, 824)	26.5<br>(14.4, 39.4)	865<br>(472, 1304)	27.6<br>(14.9, 40.8)	4.5<br>(-23.8, 47.0)
Uganda	1141<br>(507, 1645)	20.5<br>(9.1, 29.8)	3113<br>(1463, 4524)	26.5<br>(12.2, 38.3)	29.2<br>(-1.6, 70.4)
United Republic of Tanzania	2354<br>(1338, 3445)	25.3<br>(14.3, 37.7)	6831<br>(3170, 10286)	32.6<br>(15.0, 48.6)	28.8<br>(-10.3, 82.0)
Zambia	500<br>(340, 742)	21.8<br>(14.3, 32.0)	1925<br>(1290, 2879)	35.5<br>(23.5, 52.4)	62.7<br>(15.0, 127.8)
Southern Sub-Saharan Africa	8420<br>(6537, 10483)	32.7<br>(25.2, 41.3)	22036<br>(17674, 26085)	41.8<br>(33.4, 49.9)	27.9<br>(10.4, 45.2)
Botswana	128<br>(86, 173)	24.1<br>(16.2, 32.8)	396<br>(256, 555)	31.4<br>(20.1, 43.0)	29.9<br>(-5.2, 81.2)
Lesotho	154<br>(105, 222)	16.9<br>(11.5, 24.3)	282<br>(189, 387)	24.9<br>(16.8, 34.1)	47.1<br>(15.1, 96.2)
Namibia	175<br>(120, 239)	25.7<br>(17.3, 35.1)	399<br>(277, 563)	30.3<br>(20.9, 41.9)	17.9<br>(-12.4, 59.1)
South Africa	7642<br>(5929, 9403)	38.5<br>(29.8, 47.8)	20321<br>(16339, 23826)	47.8<br>(38.3, 57.0)	24.2<br>(6.4, 42.0)
Eswatini	55<br>(38, 75)	21.0<br>(14.4, 29.1)	145<br>(102, 203)	27.9<br>(19.3, 38.3)	33.0<br>(0.6, 81.9)
Zimbabwe	266<br>(147, 440)	8.1<br>(4.5, 13.2)	493<br>(280, 805)	8.9<br>(5.1, 14.7)	10.2<br>(1.1, 19.6)
Western Sub-Saharan Africa	6908<br>(4643, 9971)	9.3<br>(6.3, 13.6)	16088<br>(10061, 22820)	10.5<br>(6.6, 15.3)	13.2<br>(-7.5, 37.1)
Benin	173<br>(110, 264)	9.6<br>(6.1, 14.7)	437<br>(270, 641)	10.9<br>(6.8, 15.8)	13.1<br>(-7.4, 43.8)

Burkina Faso	346<br>(213, 524)	9.1<br>(5.7, 13.6)	982<br>(578, 1509)	12.8<br>(7.6, 19.4)	40.5<br>(7.5, 92.5)
Cameroon	377<br>(240, 553)	10.0<br>(6.4, 14.6)	1206<br>(743, 1775)	12.6<br>(7.9, 18.1)	25.7<br>(-5.1, 70.7)
Cabo Verde	25<br>(16, 37)	10.4<br>(6.7, 15.4)	56<br>(35, 78)	13.8<br>(8.6, 19.3)	33.1<br>(2.4, 87.6)
Chad	229<br>(147, 344)	9.1<br>(5.8, 13.3)	471<br>(285, 691)	10.2<br>(6.3, 14.9)	12.9<br>(-7.6, 42.8)
Cote d'Ivoire	290<br>(186, 418)	9.8<br>(6.3, 14.2)	874<br>(544, 1279)	10.8<br>(6.8, 15.9)	10.6<br>(-9.4, 45.4)
Gambia	27<br>(17, 42)	9.6<br>(5.9, 14.5)	97<br>(59, 145)	11.9<br>(7.3, 17.6)	23.5<br>(-1.9, 64.9)
Ghana	386<br>(233, 594)	7.6<br>(4.6, 11.8)	927<br>(557, 1494)	7.2<br>(4.4, 11.4)	-6.1<br>(-21.2, 12.0)
Guinea	276<br>(168, 420)	9.2<br>(5.6, 13.8)	503<br>(295, 762)	10.4<br>(6.2, 15.6)	14.0<br>(-8.3, 50.2)
Guinea-Bissau	34<br>(21, 52)	10.0<br>(6.4, 15.3)	65<br>(39, 98)	11.5<br>(7.1, 16.9)	14.8<br>(-10.6, 54.3)
Liberia	100<br>(65, 146)	10.0<br>(6.5, 14.6)	173<br>(104, 260)	10.7<br>(6.5, 16.2)	7.7<br>(-11.8, 37.9)
Mali	314<br>(192, 486)	8.9<br>(5.6, 13.5)	743<br>(462, 1104)	10.3<br>(6.5, 15.1)	15.9<br>(-6.5, 52.3)
Mauritania	96<br>(61, 140)	10.9<br>(7.1, 16.0)	210<br>(116, 311)	11.4<br>(6.4, 16.6)	3.9<br>(-21.5, 36.1)
Niger	196<br>(123, 293)	8.9<br>(5.6, 13.3)	618<br>(367, 962)	9.7<br>(5.8, 14.6)	9.5<br>(-9.7, 41.6)
Nigeria	3475<br>(2350, 5077)	9.3<br>(6.3, 13.7)	7321<br>(4395, 10933)	10.5<br>(6.4, 15.7)	12.9<br>(-18.1, 53.3)
Sao Tome and Principe	6<br>(4, 8)	9.5<br>(6.0, 14.2)	11<br>(6, 16)	12.1<br>(7.5, 17.7)	28.2<br>(1.6, 72.8)
Senegal	288<br>(177, 424)	10.3<br>(6.6, 15.4)	747<br>(443, 1096)	11.5<br>(6.9, 16.6)	11.4<br>(-10.1, 44.6)
Sierra Leone	169<br>(104, 266)	9.8<br>(6.0, 15.4)	307<br>(186, 469)	10.3<br>(6.3, 15.6)	5.4<br>(-11.9, 36.0)
Togo	103<br>(65, 150)	10.3<br>(6.6, 14.9)	341<br>(201, 516)	11.9<br>(7.1, 17.7)	16.2<br>(-7.9, 54.2)
High SDI	317532<br>(192568, 501764)	29.9<br>(18.1, 47.4)	562292<br>(323078, 930388)	27.3<br>(15.7, 45.4)	-8.9<br>(-24.7, 3.4)
High-middle SDI	287815<br>(166330, 460824)	29.6<br>(17.1, 46.7)	520674<br>(318434, 857842)	25.7<br>(15.6, 42.1)	-13.0<br>(-28.4, -1.1)
Middle SDI	93993<br>(58814, 143627)	11.2<br>(7.0, 16.9)	250750<br>(170324, 373841)	11.2<br>(7.6, 16.9)	0.1<br>(-8.1, 12.0)

Low-middle SDI	45940<br>(29139, 69952)	9.7<br>(6.1, 14.7)	128679<br>(89770, 184475)	10.8<br>(7.5, 15.5)	11.0<br>(-0.8, 27.8)
Low SDI	29758<br>(18023, 41464)	15.2<br>(9.2, 21.4)	72943<br>(46271, 98650)	16.8<br>(10.4, 22.9)	10.3<br>(-7.5, 30.5)

Table S3: Deaths due to PAD in 1990 and 2019 for both sexes and percentage change in age-standardised rates by location. PAD= Peripheral artery disease.

Deaths of PAD	1990		2019		Percentage change in age-standardised rates between 1990 and 2019
	Counts (95% UI)	Age-standardised rate (95% UI)	Counts (95% UI)	Age-standardised rate (95% UI)	
Global	30168 (16181, 52436)	1.0 (0.5, 1.8)	74063 (41183, 128164)	1.0 (0.6, 1.7)	-2.5 (-21.9, 9.8)
East Asia	628 (498, 845)	0.1 (0.1, 0.2)	2279 (1874, 2830)	0.1 (0.1, 0.2)	21.1 (-13.7, 51.8)
China	609 (480, 826)	0.1 (0.1, 0.2)	2209 (1810, 2745)	0.1 (0.1, 0.2)	21.2 (-14.3, 52.3)
Democratic People's Republic of Korea	11 (7, 15)	0.1 (0.1, 0.1)	29 (20, 37)	0.1 (0.1, 0.1)	6.9 (-22.1, 50.2)
Taiwan	8 (4, 16)	0.1 (0.0, 0.1)	41 (18, 81)	0.1 (0.0, 0.2)	26.0 (-18.3, 84.1)
Southeast Asia	226 (183, 278)	0.1 (0.1, 0.2)	913 (717, 1128)	0.2 (0.2, 0.2)	52.9 (23.5, 80.5)
Cambodia	3 (2, 4)	0.1 (0.1, 0.1)	13 (9, 20)	0.2 (0.1, 0.2)	72.7 (13.1, 147.6)
Indonesia	87 (65, 115)	0.1 (0.1, 0.2)	363 (245, 528)	0.3 (0.2, 0.4)	90.2 (47.4, 140.5)
Lao People's Democratic Republic	2 (1, 2)	0.1 (0.1, 0.2)	6 (4, 8)	0.2 (0.1, 0.3)	63.5 (15.7, 134.3)
Malaysia	7 (6, 9)	0.1 (0.1, 0.1)	35 (26, 45)	0.2 (0.1, 0.2)	80.1 (30.9, 144.8)
Maldives	0 (0, 0)	0.2 (0.2, 0.5)	1 (1, 2)	0.4 (0.3, 0.7)	76.2 (-22.8, 178.5)
Mauritius	1 (0, 2)	0.2 (0.1, 0.4)	5 (2, 9)	0.3 (0.1, 0.6)	66.2 (5.0, 152.2)
Myanmar	24 (15, 34)	0.1 (0.1, 0.2)	76 (57, 102)	0.2 (0.2, 0.3)	47.6 (4.6, 105.4)
Philippines	23 (19, 27)	0.1 (0.1, 0.1)	107 (86, 130)	0.2 (0.1, 0.2)	46.4 (18.4, 75.4)
Seychelles	0 (0, 0)	0.2 (0.2, 0.3)	0 (0, 0)	0.3 (0.2, 0.5)	31.0 (-4.1, 79.2)
Sri Lanka	7 (5, 11)	0.1 (0.1, 0.1)	31 (21, 42)	0.1 (0.1, 0.2)	55.7 (-9.5, 140.2)
Thailand	17 (13, 21)	0.1 (0.1, 0.1)	81 (56, 106)	0.1 (0.1, 0.1)	11.9 (-22.5, 55.9)
Timor-Leste	0 (0, 0)	0.1 (0.1, 0.1)	1 (1, 2)	0.2 (0.1, 0.3)	95.7 (34.0, 201.8)

Viet Nam	56<br>(41, 73)	0.2<br>(0.1, 0.2)	193<br>(140, 252)	0.3<br>(0.2, 0.3)	55.3<br>(4.3, 119.9)
Oceania	2<br>(2, 4)	0.1<br>(0.1, 0.2)	9<br>(7, 13)	0.2<br>(0.2, 0.3)	47.8<br>(10.3, 92.8)
American Samoa	0<br>(0, 0)	0.2<br>(0.2, 0.3)	0<br>(0, 0)	0.3<br>(0.3, 0.4)	61.7<br>(18.0, 116.1)
Cook Islands	0<br>(0, 0)	0.3<br>(0.2, 0.4)	0<br>(0, 0)	0.4<br>(0.3, 0.5)	57.7<br>(2.9, 128.7)
Fiji	0<br>(0, 0)	0.1<br>(0.1, 0.2)	1<br>(1, 1)	0.2<br>(0.2, 0.3)	66.4<br>(-0.6, 155.3)
Guam	0<br>(0, 0)	0.2<br>(0.2, 0.3)	0<br>(0, 1)	0.2<br>(0.2, 0.3)	16.6<br>(-22.7, 62.8)
Kiribati	0<br>(0, 0)	0.2<br>(0.1, 0.4)	0<br>(0, 0)	0.2<br>(0.1, 0.5)	15.9<br>(-15.2, 60.8)
Marshall Islands	0<br>(0, 0)	0.2<br>(0.2, 0.3)	0<br>(0, 0)	0.3<br>(0.2, 0.5)	48.8<br>(10.3, 109.5)
Micronesia (Federated States of)	0<br>(0, 0)	0.2<br>(0.2, 0.4)	0<br>(0, 0)	0.4<br>(0.3, 0.5)	70.1<br>(17.7, 142.8)
Nauru	0<br>(0, 0)	0.3<br>(0.2, 0.4)	0<br>(0, 0)	0.4<br>(0.3, 0.5)	17.5<br>(-13.3, 54.7)
Niue	0<br>(0, 0)	0.2<br>(0.2, 0.4)	0<br>(0, 0)	0.4<br>(0.3, 0.5)	52.3<br>(10.3, 111.3)
Northern Mariana Islands	0<br>(0, 0)	0.4<br>(0.2, 0.5)	0<br>(0, 0)	0.6<br>(0.4, 0.8)	65.5<br>(13.6, 138.5)
Palau	0<br>(0, 0)	0.1<br>(0.0, 0.1)	0<br>(0, 0)	0.1<br>(0.1, 0.1)	19.7<br>(-21.2, 81.8)
Papua New Guinea	1<br>(1, 2)	0.1<br>(0.1, 0.2)	5<br>(3, 8)	0.2<br>(0.1, 0.3)	51.0<br>(7.1, 115.8)
Samoa	0<br>(0, 0)	0.2<br>(0.2, 0.4)	0<br>(0, 0)	0.3<br>(0.2, 0.4)	28.1<br>(-5.1, 79.3)
Solomon Islands	0<br>(0, 0)	0.1<br>(0.1, 0.2)	0<br>(0, 1)	0.2<br>(0.2, 0.3)	48.4<br>(8.2, 102.4)
Tokelau	0<br>(0, 0)	0.2<br>(0.1, 0.3)	0<br>(0, 0)	0.3<br>(0.2, 0.4)	66.6<br>(17.0, 136.2)
Tonga	0<br>(0, 0)	0.2<br>(0.1, 0.3)	0<br>(0, 0)	0.3<br>(0.2, 0.4)	51.2<br>(12.3, 108.1)
Tuvalu	0<br>(0, 0)	0.2<br>(0.1, 0.3)	0<br>(0, 0)	0.3<br>(0.2, 0.4)	51.2<br>(3.2, 119.9)
Vanuatu	0<br>(0, 0)	0.1<br>(0.1, 0.3)	0<br>(0, 0)	0.2<br>(0.2, 0.4)	87.9<br>(22.2, 182.9)
Central Asia	45<br>(22, 78)	0.1<br>(0.1, 0.2)	101<br>(52, 181)	0.2<br>(0.1, 0.3)	71.8<br>(28.2, 116.7)

Armenia	5<br>(2, 16)	0.3<br>(0.1, 0.8)	12<br>(5, 21)	0.3<br>(0.1, 0.5)	17.2<br>(-56.0, 119.1)
Azerbaijan	2<br>(1, 3)	0.1<br>(0.0, 0.1)	5<br>(3, 6)	0.1<br>(0.1, 0.1)	76.2<br>(13.1, 222.9)
Georgia	5<br>(3, 11)	0.1<br>(0.1, 0.2)	376<br>(172, 667)	0.2<br>(0.1, 0.4)	62.7<br>(8.2, 348.0)
Kazakhstan	10<br>(5, 19)	0.1<br>(0.0, 0.2)	22<br>(11, 42)	0.2<br>(0.1, 0.3)	64.7<br>(19.8, 118.5)
Kyrgyzstan	10<br>(5, 18)	0.3<br>(0.2, 0.6)	19<br>(9, 38)	0.5<br>(0.2, 1.1)	55.5<br>(1.2, 93.2)
Mongolia	1<br>(0, 1)	0.1<br>(0.1, 0.1)	1<br>(1, 2)	0.1<br>(0.1, 0.2)	33.4<br>(0.4, 86.1)
Tajikistan	2<br>(1, 3)	0.1<br>(0.0, 0.1)	2<br>(2, 3)	0.1<br>(0.1, 0.1)	42.2<br>(0.1, 153.0)
Turkmenistan	2<br>(1, 4)	0.1<br>(0.1, 0.3)	8<br>(3, 15)	0.2<br>(0.1, 0.5)	78.8<br>(18.6, 136.2)
Uzbekistan	7<br>(3, 14)	0.1<br>(0.0, 0.1)	20<br>(9, 39)	0.2<br>(0.1, 0.3)	139.5<br>(41.1, 386.8)
Central Europe	1851<br>(895, 3469)	1.4<br>(0.7, 2.6)	4004<br>(1934, 7372)	1.8<br>(0.9, 3.3)	25.5<br>(-15.0, 73.7)
Albania	2<br>(2, 2)	0.1<br>(0.1, 0.1)	8<br>(6, 11)	0.2<br>(0.1, 0.3)	77.6<br>(20.9, 153.9)
Bosnia and Herzegovina	7<br>(6, 9)	0.2<br>(0.2, 0.3)	25<br>(19, 33)	0.4<br>(0.3, 0.6)	80.0<br>(32.2, 150.7)
Bulgaria	52<br>(23, 108)	0.5<br>(0.2, 1.0)	90<br>(42, 176)	0.6<br>(0.3, 1.2)	18.7<br>(-13.5, 73.4)
Croatia	117<br>(49, 211)	2.1<br>(0.9, 3.8)	219<br>(93, 407)	2.3<br>(1.0, 4.3)	8.1<br>(-23.2, 46.7)
Czechia	244<br>(114, 471)	1.9<br>(0.9, 3.6)	457<br>(215, 921)	2.0<br>(1.0, 4.1)	8.1<br>(-31.1, 56.7)
Hungary	626<br>(295, 1138)	4.6<br>(2.2, 8.4)	1011<br>(389, 2162)	4.9<br>(1.9, 10.4)	5.6<br>(-59.8, 45.9)
North Macedonia	4<br>(3, 5)	0.3<br>(0.2, 0.4)	7<br>(5, 9)	0.3<br>(0.2, 0.4)	4.4<br>(-24.6, 50.7)
Montenegro	1<br>(1, 2)	0.2<br>(0.2, 0.3)	3<br>(2, 3)	0.3<br>(0.2, 0.4)	24.4<br>(-7.6, 71.6)
Poland	356<br>(71, 719)	0.9<br>(0.2, 1.8)	1142<br>(406, 2649)	1.5<br>(0.5, 3.5)	69.4<br>(-21.3, 1,810.1)
Romania	371<br>(159, 745)	1.6<br>(0.7, 3.1)	776<br>(349, 1514)	1.9<br>(0.9, 3.8)	23.4<br>(-12.5, 56.8)
Serbia	29<br>(23, 44)	0.3<br>(0.2, 0.5)	138<br>(94, 180)	0.9<br>(0.6, 1.2)	201.2<br>(65.5, 348.3)

Slovakia	14<br>(12, 21)	0.3<br>(0.2, 0.4)	93<br>(54, 125)	1.0<br>(0.6, 1.4)	292.8<br>(95.3, 478.3)
Slovenia	26<br>(7, 84)	1.1<br>(0.3, 3.6)	36<br>(5, 78)	0.7<br>(0.1, 1.5)	-36.6<br>(-97.6, 64.9)
Eastern Europe	6485<br>(3026, 11783)	2.7<br>(1.2, 4.8)	12331<br>(5956, 23564)	3.5<br>(1.7, 6.7)	31.6<br>(-7.4, 63.5)
Belarus	76<br>(31, 142)	0.6<br>(0.3, 1.2)	175<br>(67, 376)	1.1<br>(0.4, 2.3)	69.9<br>(13.5, 131.7)
Estonia	2<br>(1, 4)	0.1<br>(0.1, 0.2)	7<br>(3, 15)	0.2<br>(0.1, 0.4)	92.9<br>(-1.6, 535.3)
Latvia	8<br>(3, 17)	0.2<br>(0.1, 0.5)	30<br>(8, 94)	0.6<br>(0.2, 2.1)	161.3<br>(9.8, 2,368.9)
Lithuania	35<br>(15, 70)	0.8<br>(0.3, 1.6)	88<br>(38, 161)	1.3<br>(0.6, 2.4)	66.7<br>(10.6, 448.5)
Republic of Moldova	11<br>(3, 22)	0.3<br>(0.1, 0.6)	34<br>(14, 76)	0.6<br>(0.2, 1.3)	84.3<br>(-7.6, 1,403.3)
Russian Federation	4468<br>(2023, 8076)	2.9<br>(1.3, 5.2)	9052<br>(4413, 17633)	3.8<br>(1.9, 7.4)	31.4<br>(-21.3, 76.1)
Ukraine	1884<br>(904, 3575)	3.0<br>(1.4, 5.5)	2945<br>(1405, 5400)	3.8<br>(1.8, 7.0)	27.2<br>(2.4, 55.5)
High-income Asia Pacific	377<br>(213, 663)	0.2<br>(0.1, 0.4)	1282<br>(651, 2114)	0.2<br>(0.1, 0.4)	-7.6<br>(-38.9, 37.3)
Brunei Darussalam	0<br>(0, 1)	1.0<br>(0.7, 1.3)	2<br>(1, 2)	1.2<br>(0.9, 1.5)	25.8<br>(-12.4, 75.8)
Japan	296<br>(139, 580)	0.2<br>(0.1, 0.4)	1044<br>(429, 1853)	0.2<br>(0.1, 0.4)	-2.6<br>(-39.1, 53.0)
Republic of Korea	75<br>(60, 95)	0.4<br>(0.3, 0.5)	205<br>(163, 249)	0.3<br>(0.2, 0.3)	-33.8<br>(-53.1, -13.3)
Singapore	5<br>(2, 10)	0.3<br>(0.2, 0.7)	31<br>(10, 70)	0.4<br>(0.1, 1.0)	28.9<br>(-41.5, 410.0)
Australasia	454<br>(207, 787)	2.2<br>(1.0, 3.8)	1455<br>(697, 2744)	2.5<br>(1.2, 4.7)	14.0<br>(-40.3, 50.8)
Australia	387<br>(175, 671)	2.2<br>(1.0, 3.9)	1259<br>(598, 2360)	2.5<br>(1.2, 4.8)	13.4<br>(-41.7, 50.7)
New Zealand	67<br>(31, 116)	1.9<br>(0.9, 3.2)	196<br>(94, 373)	2.2<br>(1.0, 4.1)	15.9<br>(-32.7, 50.8)
Western Europe	9953<br>(4791, 18686)	1.7<br>(0.8, 3.2)	21922<br>(10276, 40349)	1.9<br>(0.9, 3.5)	11.0<br>(-20.5, 32.6)
Andorra	0<br>(0, 0)	0.9<br>(0.6, 1.3)	3<br>(2, 4)	1.7<br>(1.0, 2.5)	102.0<br>(16.0, 216.3)
Austria	332<br>(156, 606)	2.8<br>(1.3, 5.0)	732<br>(351, 1394)	3.3<br>(1.6, 6.4)	19.3<br>(-18.4, 53.6)

Belgium	149<br>(72, 276)	1.0<br>(0.5, 1.8)	329<br>(153, 627)	1.1<br>(0.5, 2.1)	15.8<br>(-15.1, 41.1)
Cyprus	5<br>(3, 7)	1.0<br>(0.5, 1.5)	20<br>(12, 25)	1.3<br>(0.7, 1.6)	32.4<br>(-13.2, 104.8)
Denmark	207<br>(98, 372)	2.4<br>(1.1, 4.2)	345<br>(164, 643)	2.6<br>(1.2, 4.8)	9.0<br>(-33.8, 38.4)
Finland	76<br>(30, 136)	1.1<br>(0.4, 2.0)	207<br>(84, 371)	1.3<br>(0.5, 2.4)	19.8<br>(-15.2, 53.1)
France	594<br>(289, 1148)	0.7<br>(0.3, 1.3)	1250<br>(546, 2376)	0.7<br>(0.3, 1.3)	-1.3<br>(-30.4, 41.4)
Germany	3607<br>(1549, 7082)	2.8<br>(1.2, 5.4)	7750<br>(3791, 14300)	3.3<br>(1.6, 6.2)	18.9<br>(-18.9, 139.2)
Greece	47<br>(21, 84)	0.3<br>(0.2, 0.6)	119<br>(53, 212)	0.4<br>(0.2, 0.7)	5.8<br>(-19.5, 27.8)
Iceland	2<br>(1, 3)	0.6<br>(0.3, 1.0)	4<br>(2, 8)	0.6<br>(0.3, 1.1)	4.3<br>(-28.4, 28.6)
Ireland	110<br>(50, 195)	3.0<br>(1.4, 5.3)	246<br>(116, 482)	3.1<br>(1.5, 6.1)	3.7<br>(-50.4, 38.1)
Israel	90<br>(42, 172)	2.1<br>(1.0, 4.1)	333<br>(163, 602)	2.5<br>(1.3, 4.6)	18.5<br>(-12.6, 67.8)
Italy	1736<br>(799, 3135)	2.0<br>(0.9, 3.7)	3615<br>(1699, 6909)	1.9<br>(0.9, 3.7)	-7.3<br>(-44.6, 19.4)
Luxembourg	5<br>(2, 9)	1.0<br>(0.5, 1.8)	13<br>(6, 25)	1.1<br>(0.5, 2.2)	10.4<br>(-31.4, 45.1)
Malta	7<br>(3, 13)	1.9<br>(0.9, 3.5)	22<br>(10, 43)	2.1<br>(1.0, 4.1)	13.2<br>(-21.8, 40.4)
Monaco	1<br>(0, 1)	0.6<br>(0.5, 0.9)	1<br>(1, 1)	0.7<br>(0.6, 1.0)	14.3<br>(-13.5, 53.0)
Netherlands	573<br>(278, 1041)	2.8<br>(1.4, 5.1)	1205<br>(578, 2197)	3.1<br>(1.5, 5.7)	9.8<br>(-11.1, 26.8)
Norway	100<br>(41, 174)	1.3<br>(0.5, 2.3)	158<br>(74, 297)	1.4<br>(0.6, 2.6)	3.8<br>(-47.0, 33.3)
Portugal	279<br>(123, 485)	2.4<br>(1.1, 4.2)	776<br>(362, 1439)	2.5<br>(1.2, 4.8)	6.7<br>(-28.1, 42.7)
Spain	717<br>(330, 1309)	1.4<br>(0.6, 2.5)	1890<br>(885, 3505)	1.5<br>(0.7, 2.8)	6.6<br>(-19.2, 34.8)
Sweden	176<br>(68, 364)	1.0<br>(0.4, 2.1)	296<br>(135, 544)	1.1<br>(0.5, 2.0)	2.8<br>(-47.3, 41.0)
Switzerland	152<br>(67, 269)	1.3<br>(0.6, 2.4)	309<br>(151, 574)	1.4<br>(0.7, 2.6)	2.4<br>(-37.2, 32.3)
United Kingdom	980<br>(337, 1950)	1.1<br>(0.4, 2.1)	2280<br>(948, 3973)	1.5<br>(0.6, 2.6)	41.9<br>(-12.4, 510.2)

Southern Latin America	163<br>(75, 305)	0.4<br>(0.2, 0.8)	553<br>(263, 1038)	0.6<br>(0.3, 1.2)	54.9<br>(14.2, 89.0)
Argentina	100<br>(46, 189)	0.4<br>(0.2, 0.7)	257<br>(124, 504)	0.5<br>(0.2, 0.9)	28.2<br>(-4.5, 51.3)
Chile	60<br>(27, 112)	0.8<br>(0.3, 1.4)	288<br>(137, 537)	1.2<br>(0.6, 2.2)	55.9<br>(7.0, 109.3)
Uruguay	3<br>(2, 6)	0.1<br>(0.0, 0.2)	7<br>(4, 14)	0.1<br>(0.1, 0.2)	24.1<br>(-36.0, 50.0)
High-income North America	6264<br>(3052, 11559)	1.7<br>(0.8, 3.1)	15958<br>(7777, 29844)	2.2<br>(1.1, 4.2)	30.9<br>(5.6, 50.7)
Canada	506<br>(232, 889)	1.6<br>(0.7, 2.9)	1429<br>(694, 2679)	1.8<br>(0.9, 3.4)	12.0<br>(-29.1, 41.1)
United States of America	5757<br>(2792, 10694)	1.7<br>(0.8, 3.2)	14527<br>(6980, 27189)	2.3<br>(1.1, 4.3)	33.2<br>(6.7, 53.9)
Greenland	1<br>(1, 1)	3.1<br>(2.5, 4.0)	1<br>(1, 2)	2.5<br>(1.9, 3.2)	-21.1<br>(-39.8, 3.0)
Caribbean	361<br>(189, 663)	1.6<br>(0.8, 2.9)	1024<br>(537, 1803)	1.9<br>(1.0, 3.4)	20.8<br>(-17.0, 48.0)
Antigua and Barbuda	1<br>(0, 2)	1.6<br>(0.8, 3.0)	2<br>(1, 4)	2.3<br>(1.1, 4.4)	46.1<br>(5.9, 95.7)
Bahamas	3<br>(1, 5)	2.3<br>(1.1, 4.3)	9<br>(4, 17)	2.8<br>(1.3, 5.3)	21.5<br>(-11.7, 53.1)
Barbados	17<br>(8, 32)	5.8<br>(2.8, 10.8)	35<br>(16, 64)	7.1<br>(3.4, 13.2)	23.2<br>(-11.8, 53.2)
Belize	0<br>(0, 1)	0.4<br>(0.2, 0.8)	1<br>(1, 3)	0.6<br>(0.3, 1.1)	50.9<br>(-5.5, 106.5)
Bermuda	2<br>(1, 4)	3.7<br>(1.7, 7.2)	5<br>(3, 10)	3.7<br>(1.8, 6.7)	0.1<br>(-34.6, 42.8)
Cuba	205<br>(96, 402)	2.1<br>(1.0, 4.2)	577<br>(265, 1055)	2.8<br>(1.3, 5.1)	29.8<br>(-17.4, 78.7)
Dominica	1<br>(1, 1)	1.3<br>(1.0, 1.6)	2<br>(1, 2)	1.8<br>(1.4, 2.3)	37.7<br>(5.0, 80.2)
Dominican Republic	6<br>(4, 7)	0.2<br>(0.1, 0.3)	28<br>(19, 38)	0.3<br>(0.2, 0.5)	70.0<br>(26.3, 128.8)
Grenada	2<br>(1, 3)	2.0<br>(0.9, 3.9)	3<br>(1, 5)	3.0<br>(1.4, 5.6)	53.1<br>(-6.8, 109.4)
Guyana	2<br>(1, 4)	0.7<br>(0.3, 1.4)	5<br>(2, 10)	1.1<br>(0.5, 2.1)	50.9<br>(-3.5, 148.6)
Haiti	25<br>(16, 40)	1.2<br>(0.7, 1.9)	58<br>(37, 94)	1.2<br>(0.8, 2.0)	0.8<br>(-32.0, 48.9)
Jamaica	28<br>(11, 59)	1.5<br>(0.6, 3.2)	74<br>(32, 138)	2.2<br>(0.9, 4.2)	40.5<br>(-19.8, 106.4)

Puerto Rico	48<br>(23, 89)	1.4<br>(0.7, 2.7)	160<br>(73, 312)	1.8<br>(0.8, 3.6)	23.8<br>(-21.1, 69.6)
Saint Kitts and Nevis	1<br>(1, 2)	3.9<br>(1.8, 7.9)	2<br>(1, 5)	5.1<br>(2.4, 9.9)	30.0<br>(-24.1, 70.5)
Saint Lucia	2<br>(1, 4)	3.2<br>(1.5, 6.1)	8<br>(4, 14)	3.9<br>(1.9, 7.4)	20.4<br>(-27.7, 50.4)
Saint Vincent and the Grenadines	1<br>(0, 1)	1.3<br>(0.6, 2.3)	2<br>(1, 4)	1.8<br>(0.8, 3.3)	37.4<br>(-10.8, 98.4)
Suriname	0<br>(0, 0)	0.2<br>(0.1, 0.2)	1<br>(1, 1)	0.2<br>(0.2, 0.3)	27.7<br>(-3.0, 64.9)
Trinidad and Tobago	4<br>(2, 8)	0.6<br>(0.2, 1.5)	12<br>(5, 23)	0.7<br>(0.3, 1.3)	12.1<br>(-35.1, 72.0)
United States Virgin Islands	1<br>(1, 2)	2.4<br>(1.9, 3.4)	6<br>(5, 8)	3.6<br>(2.8, 5.0)	49.1<br>(12.3, 94.7)
Andean Latin America	18<br>(13, 22)	0.1<br>(0.1, 0.1)	71<br>(52, 89)	0.1<br>(0.1, 0.2)	19.6<br>(-8.6, 58.1)
Bolivia (Plurinational State of)	3<br>(2, 4)	0.1<br>(0.1, 0.2)	13<br>(8, 18)	0.2<br>(0.1, 0.3)	56.9<br>(9.4, 122.2)
Ecuador	3<br>(3, 4)	0.1<br>(0.1, 0.1)	19<br>(14, 25)	0.2<br>(0.1, 0.2)	100.4<br>(48.2, 171.4)
Peru	12<br>(8, 15)	0.1<br>(0.1, 0.2)	39<br>(27, 54)	0.1<br>(0.1, 0.2)	-3.9<br>(-33.5, 44.0)
Central Latin America	344<br>(165, 622)	0.6<br>(0.3, 1.0)	1298<br>(632, 2562)	0.6<br>(0.3, 1.2)	6.5<br>(-36.6, 46.1)
Colombia	63<br>(28, 112)	0.5<br>(0.2, 0.9)	307<br>(133, 639)	0.5<br>(0.2, 1.1)	11.4<br>(-40.9, 68.3)
Costa Rica	4<br>(2, 8)	0.3<br>(0.1, 0.5)	20<br>(9, 39)	0.4<br>(0.2, 0.7)	33.3<br>(-12.5, 79.3)
El Salvador	2<br>(1, 2)	0.1<br>(0.1, 0.1)	7<br>(4, 9)	0.1<br>(0.1, 0.1)	38.9<br>(1.2, 87.3)
Guatemala	2<br>(1, 3)	0.1<br>(0.0, 0.2)	9<br>(4, 17)	0.1<br>(0.0, 0.2)	24.4<br>(-25.7, 97.3)
Honduras	6<br>(3, 10)	0.4<br>(0.2, 0.7)	20<br>(12, 28)	0.5<br>(0.3, 0.7)	24.3<br>(-13.1, 87.3)
Mexico	226<br>(106, 411)	0.7<br>(0.3, 1.3)	759<br>(348, 1545)	0.7<br>(0.3, 1.5)	1.6<br>(-42.3, 46.3)
Nicaragua	1<br>(1, 1)	0.1<br>(0.1, 0.1)	6<br>(4, 7)	0.2<br>(0.1, 0.2)	100.9<br>(56.7, 156.1)
Panama	11<br>(5, 21)	0.8<br>(0.3, 1.6)	47<br>(21, 93)	1.1<br>(0.5, 2.1)	29.5<br>(-31.5, 85.6)
Venezuela (Bolivarian Republic of)	30<br>(14, 56)	0.4<br>(0.2, 0.8)	124<br>(56, 251)	0.5<br>(0.2, 0.9)	14.6<br>(-22.9, 54.7)

Tropical Latin America	731 (331, 1340)	1.1 (0.5, 1.9)	2794 (1281, 5177)	1.2 (0.6, 2.3)	15.5 (-16.7, 52.1)
Brazil	727 (327, 1336)	1.1 (0.5, 2.0)	2770 (1257, 5156)	1.3 (0.6, 2.3)	13.9 (-18.4, 50.1)
Paraguay	3 (2, 4)	0.2 (0.1, 0.2)	24 (16, 32)	0.5 (0.3, 0.6)	160.9 (76.4, 263.9)
North Africa and Middle East	395 (303, 537)	0.3 (0.2, 0.5)	1339 (1093, 1831)	0.4 (0.3, 0.6)	28.7 (-2.3, 66.2)
Afghanistan	4 (2, 6)	0.1 (0.0, 0.1)	10 (6, 15)	0.1 (0.1, 0.2)	66.2 (15.0, 141.1)
Algeria	14 (8, 22)	0.2 (0.1, 0.3)	76 (47, 116)	0.3 (0.2, 0.5)	57.8 (11.7, 130.2)
Bahrain	1 (1, 2)	1.2 (0.9, 1.5)	7 (5, 9)	1.5 (1.1, 1.9)	26.2 (-12.2, 76.4)
Egypt	17 (13, 26)	0.1 (0.1, 0.1)	74 (50, 108)	0.2 (0.1, 0.3)	101.6 (43.3, 186.0)
Iran (Islamic Republic of)	16 (12, 23)	0.1 (0.1, 0.2)	171 (123, 197)	0.3 (0.2, 0.3)	153.3 (45.0, 280.4)
Iraq	6 (4, 11)	0.1 (0.1, 0.2)	20 (15, 32)	0.1 (0.1, 0.2)	20.3 (-17.4, 86.9)
Jordan	1 (1, 1)	0.1 (0.1, 0.1)	4 (3, 5)	0.1 (0.1, 0.1)	20.5 (-13.3, 60.1)
Kuwait	0 (0, 0)	0.1 (0.0, 0.1)	2 (1, 4)	0.1 (0.1, 0.2)	54.7 (-4.5, 127.3)
Lebanon	2 (1, 3)	0.1 (0.1, 0.2)	13 (8, 17)	0.3 (0.2, 0.3)	75.7 (7.1, 175.7)
Libya	1 (1, 2)	0.1 (0.1, 0.1)	7 (4, 9)	0.2 (0.1, 0.2)	98.4 (25.4, 209.8)
Morocco	9 (6, 13)	0.1 (0.1, 0.1)	52 (35, 70)	0.2 (0.2, 0.3)	133.6 (61.2, 223.8)
Palestine	0 (0, 1)	0.1 (0.0, 0.1)	1 (1, 2)	0.1 (0.1, 0.1)	35.3 (-6.4, 119.3)
Oman	1 (0, 1)	0.2 (0.1, 0.3)	3 (2, 5)	0.4 (0.2, 0.6)	126.9 (54.9, 253.7)
Qatar	0 (0, 0)	0.5 (0.3, 0.8)	1 (1, 2)	1.3 (0.8, 1.8)	136.1 (53.8, 311.2)
Saudi Arabia	3 (2, 3)	0.1 (0.0, 0.1)	8 (6, 10)	0.1 (0.1, 0.1)	43.0 (-8.8, 164.2)
Sudan	6 (3, 9)	0.1 (0.0, 0.1)	25 (15, 37)	0.2 (0.1, 0.3)	103.7 (36.7, 199.1)
Syrian Arab Republic	3 (2, 3)	0.1 (0.0, 0.1)	6 (4, 8)	0.1 (0.1, 0.1)	22.3 (-14.2, 77.4)

Tunisia	4<br>(3, 5)	0.1<br>(0.1, 0.2)	23<br>(16, 31)	0.2<br>(0.2, 0.3)	90.8<br>(30.8, 169.1)
Türkiye	303<br>(230, 419)	1.0<br>(0.8, 1.5)	815<br>(607, 1289)	1.0<br>(0.7, 1.6)	-0.7<br>(-31.7, 39.1)
United Arab Emirates	0<br>(0, 1)	0.2<br>(0.1, 0.4)	5<br>(2, 10)	0.3<br>(0.2, 0.7)	63.3<br>(-5.2, 185.3)
Yemen	3<br>(1, 4)	0.1<br>(0.1, 0.1)	13<br>(9, 21)	0.2<br>(0.1, 0.2)	83.3<br>(17.3, 180.2)
South Asia	550<br>(414, 896)	0.2<br>(0.1, 0.3)	2964<br>(2160, 3795)	0.3<br>(0.2, 0.4)	71.1<br>(11.6, 114.9)
Bangladesh	49<br>(33, 88)	0.2<br>(0.1, 0.3)	338<br>(180, 495)	0.3<br>(0.2, 0.5)	126.0<br>(29.7, 248.9)
Bhutan	0<br>(0, 0)	0.2<br>(0.1, 0.3)	2<br>(1, 3)	0.4<br>(0.3, 0.6)	152.1<br>(38.9, 286.1)
India	397<br>(302, 652)	0.2<br>(0.1, 0.3)	2315<br>(1724, 2974)	0.3<br>(0.2, 0.4)	70.3<br>(5.7, 118.0)
Nepal	7<br>(4, 14)	0.1<br>(0.1, 0.2)	49<br>(30, 68)	0.3<br>(0.2, 0.4)	145.7<br>(51.7, 262.7)
Pakistan	96<br>(60, 158)	0.2<br>(0.1, 0.4)	261<br>(176, 376)	0.4<br>(0.3, 0.5)	71.4<br>(19.3, 130.0)
Central Sub-Saharan Africa	347<br>(122, 647)	2.4<br>(0.9, 4.4)	800<br>(408, 1271)	2.2<br>(1.1, 3.5)	-6.9<br>(-33.8, 57.9)
Angola	27<br>(15, 41)	1.1<br>(0.5, 1.6)	116<br>(64, 160)	1.6<br>(0.8, 2.2)	52.5<br>(4.6, 124.9)
Central African Republic	10<br>(5, 15)	1.2<br>(0.5, 1.9)	17<br>(9, 27)	1.3<br>(0.6, 2.0)	3.6<br>(-24.3, 43.1)
Congo	15<br>(8, 21)	2.0<br>(1.1, 2.8)	44<br>(27, 62)	2.6<br>(1.5, 3.6)	27.6<br>(-9.7, 86.9)
Democratic Republic of the Congo	285<br>(82, 556)	2.8<br>(0.8, 5.6)	589<br>(265, 1020)	2.3<br>(1.0, 4.0)	-18.0<br>(-43.9, 47.9)
Equatorial Guinea	2<br>(1, 3)	1.2<br>(0.5, 1.8)	9<br>(4, 16)	2.9<br>(1.2, 4.7)	142.6<br>(39.0, 364.1)
Gabon	9<br>(4, 15)	2.0<br>(0.8, 3.2)	24<br>(14, 35)	3.1<br>(1.7, 4.3)	53.1<br>(7.4, 136.6)
Eastern Sub-Saharan Africa	511<br>(272, 781)	1.0<br>(0.5, 1.6)	1604<br>(852, 2134)	1.4<br>(0.7, 1.9)	40.7<br>(8.9, 77.3)
Burundi	19<br>(10, 31)	1.1<br>(0.5, 1.8)	32<br>(16, 48)	1.1<br>(0.5, 1.6)	-2.5<br>(-35.8, 44.8)
Comoros	2<br>(1, 3)	1.1<br>(0.5, 1.8)	5<br>(2, 8)	1.3<br>(0.6, 1.9)	18.2<br>(-20.1, 74.0)
Djibouti	1<br>(0, 2)	1.2<br>(0.5, 2.0)	6<br>(3, 10)	1.7<br>(0.7, 2.6)	40.4<br>(-2.3, 105.6)

Eritrea	5<br>(2, 8)	0.8<br>(0.3, 1.4)	19<br>(9, 29)	1.2<br>(0.5, 1.9)	56.2<br>(-0.4, 158.5)
Ethiopia	113<br>(61, 181)	0.9<br>(0.5, 1.5)	404<br>(219, 585)	1.3<br>(0.7, 1.9)	49.4<br>(-6.2, 125.7)
Kenya	49<br>(26, 74)	0.8<br>(0.4, 1.3)	191<br>(98, 260)	1.3<br>(0.6, 1.8)	59.9<br>(23.2, 103.9)
Madagascar	49<br>(26, 76)	1.3<br>(0.6, 2.0)	113<br>(56, 168)	1.6<br>(0.7, 2.4)	23.0<br>(-16.3, 74.4)
Malawi	21<br>(9, 35)	0.9<br>(0.4, 1.4)	60<br>(29, 88)	1.2<br>(0.5, 1.7)	34.8<br>(-3.4, 89.8)
Mozambique	39<br>(17, 58)	1.0<br>(0.4, 1.5)	128<br>(66, 183)	1.7<br>(0.8, 2.4)	69.8<br>(22.0, 151.7)
Rwanda	20<br>(9, 38)	1.0<br>(0.4, 2.0)	47<br>(19, 74)	1.2<br>(0.5, 1.9)	15.0<br>(-28.2, 70.3)
Somalia	13<br>(6, 22)	0.8<br>(0.3, 1.4)	31<br>(12, 53)	0.8<br>(0.3, 1.4)	-2.2<br>(-34.8, 40.4)
South Sudan	23<br>(11, 36)	1.3<br>(0.6, 2.0)	37<br>(17, 57)	1.4<br>(0.6, 2.1)	11.1<br>(-23.1, 60.4)
Uganda	43<br>(13, 68)	0.9<br>(0.3, 1.5)	133<br>(50, 204)	1.3<br>(0.5, 2.0)	44.7<br>(2.4, 116.7)
United Republic of Tanzania	94<br>(46, 154)	1.2<br>(0.6, 2.1)	313<br>(117, 489)	1.7<br>(0.6, 2.7)	39.0<br>(-9.0, 101.0)
Zambia	19<br>(12, 32)	1.0<br>(0.6, 1.7)	85<br>(53, 130)	1.9<br>(1.1, 2.9)	85.1<br>(22.2, 181.1)
Southern Sub-Saharan Africa	303<br>(234, 350)	1.3<br>(1.0, 1.5)	913<br>(722, 1030)	2.0<br>(1.6, 2.2)	49.8<br>(27.5, 70.6)
Botswana	4<br>(3, 6)	0.8<br>(0.5, 1.2)	14<br>(9, 20)	1.3<br>(0.8, 1.8)	53.9<br>(4.6, 124.3)
Lesotho	4<br>(3, 6)	0.5<br>(0.3, 0.7)	9<br>(6, 13)	0.9<br>(0.6, 1.3)	91.4<br>(33.8, 178.5)
Namibia	6<br>(4, 8)	0.9<br>(0.6, 1.3)	15<br>(10, 22)	1.3<br>(0.8, 1.8)	43.8<br>(1.6, 103.5)
South Africa	285<br>(220, 331)	1.6<br>(1.2, 1.8)	865<br>(688, 980)	2.3<br>(1.8, 2.6)	45.2<br>(22.7, 66.0)
Eswatini	2<br>(1, 2)	0.7<br>(0.4, 0.9)	5<br>(3, 7)	1.1<br>(0.7, 1.6)	62.7<br>(11.3, 134.7)
Zimbabwe	2<br>(2, 3)	0.1<br>(0.1, 0.1)	5<br>(3, 6)	0.1<br>(0.1, 0.1)	14.9<br>(-13.5, 54.1)
Western Sub-Saharan Africa	159<br>(114, 221)	0.2<br>(0.2, 0.3)	452<br>(247, 603)	0.3<br>(0.2, 0.4)	38.3<br>(-8.9, 94.0)
Benin	4<br>(2, 6)	0.2<br>(0.1, 0.3)	12<br>(6, 17)	0.3<br>(0.2, 0.5)	51.0<br>(1.4, 125.0)

Burkina Faso	7<br>(4, 12)	0.2<br>(0.1, 0.3)	30<br>(14, 51)	0.4<br>(0.2, 0.7)	105.6<br>(29.9, 209.5)
Cameroon	9<br>(6, 15)	0.3<br>(0.2, 0.4)	38<br>(21, 60)	0.5<br>(0.3, 0.7)	67.6<br>(10.6, 150.8)
Cabo Verde	1<br>(0, 1)	0.3<br>(0.2, 0.4)	2<br>(1, 3)	0.6<br>(0.3, 0.8)	96.1<br>(32.1, 226.8)
Chad	5<br>(3, 8)	0.2<br>(0.1, 0.3)	12<br>(6, 19)	0.3<br>(0.2, 0.5)	36.9<br>(-5.3, 98.9)
Cote d'Ivoire	6<br>(4, 9)	0.3<br>(0.2, 0.3)	22<br>(13, 35)	0.3<br>(0.2, 0.5)	32.7<br>(-10.7, 102.1)
Gambia	1<br>(0, 1)	0.2<br>(0.1, 0.3)	3<br>(1, 4)	0.4<br>(0.2, 0.6)	73.7<br>(11.8, 164.3)
Ghana	6<br>(4, 8)	0.1<br>(0.1, 0.2)	14<br>(10, 21)	0.1<br>(0.1, 0.2)	4.1<br>(-30.0, 56.7)
Guinea	6<br>(3, 10)	0.2<br>(0.1, 0.4)	14<br>(7, 23)	0.3<br>(0.2, 0.5)	42.0<br>(-4.9, 121.4)
Guinea-Bissau	1<br>(0, 1)	0.2<br>(0.2, 0.4)	2<br>(1, 3)	0.4<br>(0.2, 0.6)	52.1<br>(-8.7, 141.1)
Liberia	2<br>(1, 3)	0.2<br>(0.2, 0.3)	5<br>(3, 8)	0.3<br>(0.2, 0.5)	36.6<br>(-6.1, 102.1)
Mali	7<br>(4, 11)	0.2<br>(0.1, 0.4)	20<br>(11, 33)	0.3<br>(0.2, 0.5)	50.1<br>(2.0, 124.1)
Mauritania	2<br>(1, 3)	0.3<br>(0.2, 0.4)	7<br>(3, 10)	0.4<br>(0.2, 0.6)	25.4<br>(-18.8, 91.8)
Niger	4<br>(2, 6)	0.2<br>(0.1, 0.3)	15<br>(7, 27)	0.3<br>(0.1, 0.5)	32.6<br>(-12.5, 97.1)
Nigeria	86<br>(61, 128)	0.3<br>(0.2, 0.4)	216<br>(110, 320)	0.4<br>(0.2, 0.5)	32.9<br>(-27.9, 117.3)
Sao Tome and Principe	0<br>(0, 0)	0.2<br>(0.2, 0.3)	0<br>(0, 0)	0.4<br>(0.2, 0.6)	84.8<br>(25.6, 174.9)
Senegal	7<br>(4, 10)	0.3<br>(0.2, 0.4)	22<br>(11, 32)	0.4<br>(0.2, 0.5)	41.6<br>(-2.9, 109.1)
Sierra Leone	3<br>(2, 5)	0.2<br>(0.1, 0.3)	7<br>(4, 12)	0.3<br>(0.1, 0.4)	39.3<br>(-9.8, 105.0)
Togo	2<br>(2, 4)	0.3<br>(0.2, 0.4)	10<br>(5, 15)	0.4<br>(0.2, 0.6)	45.4<br>(-4.0, 113.9)
High SDI	14510<br>(7181, 26927)	1.4<br>(0.7, 2.6)	34713<br>(16832, 63581)	1.5<br>(0.7, 2.8)	7.3<br>(-17.9, 22.0)
High-middle SDI	12053<br>(5948, 21025)	1.4<br>(0.7, 2.5)	26243<br>(13528, 47034)	1.4<br>(0.7, 2.4)	-5.3<br>(-25.2, 11.4)
Middle SDI	1778<br>(1316, 2471)	0.3<br>(0.2, 0.4)	6643<br>(5028, 9304)	0.4<br>(0.3, 0.5)	29.3<br>(4.3, 49.8)

Low-middle SDI	868<br>(633, 1231)	0.2<br>(0.2, 0.3)	3721<br>(2773, 4762)	0.4<br>(0.3, 0.5)	55.4<br>(21.0, 82.4)
Low SDI	937<br>(502, 1369)	0.6<br>(0.3, 0.8)	2684<br>(1500, 3641)	0.7<br>(0.4, 1.0)	27.0<br>(-2.4, 66.4)

Table S4: Incidence rates and total incident cases of PAD in 1990 and 2019 for both sexes and percentage change in age-standardised rates by location. PAD= Peripheral artery disease.

Incidence of PAD	1990		2019		Percentage change in age-standardised rates between 1990 and 2019
	Counts (95% UI)	Age-standardised rate (95% UI)	Counts (95% UI)	Age-standardised rate (95% UI)	
Global	6126260 (5324212, 6999740)	156.7 (137.0, 178.3)	10504092 (9162529, 11999888)	127.1 (111.3, 145.4)	-18.9 (-19.8, -18.0)
East Asia	1071759 (923858, 1225435)	121.7 (105.8, 138.9)	2711137 (2334791, 3113092)	125.6 (109.3, 143.5)	3.2 (2.0, 4.4)
China	1029594 (887381, 1177955)	121.4 (105.5, 138.6)	2615880 (2251154, 3008605)	125.4 (109.1, 143.4)	3.3 (2.0, 4.6)
Democratic People's Republic of Korea	20524 (17548, 23636)	125.9 (108.7, 143.8)	43216 (37408, 49285)	132.6 (115.0, 151.1)	5.4 (0.9, 10.1)
Taiwan	21641 (18549, 25077)	133.9 (115.7, 153.9)	52041 (45829, 58146)	132.2 (117.2, 147.1)	-1.2 (-7.5, 6.8)
Southeast Asia	350907 (302675, 402164)	137.8 (119.4, 157.7)	876385 (758564, 1002747)	140.6 (122.2, 161.0)	2.1 (0.8, 3.3)
Cambodia	6412 (5498, 7367)	144.6 (125.0, 164.4)	16634 (14308, 19224)	139.3 (121.0, 160.0)	-3.7 (-8.2, 0.7)
Indonesia	136499 (117307, 156345)	138.6 (120.5, 158.0)	339300 (292596, 389855)	151.3 (131.9, 172.7)	9.2 (7.3, 11.1)
Lao People's Democratic Republic	3023 (2587, 3500)	146.2 (125.6, 168.0)	6180 (5329, 7113)	139.9 (121.1, 160.9)	-4.3 (-8.4, 0.2)
Malaysia	12337 (10672, 14292)	133.5 (115.1, 154.9)	34949 (30135, 40048)	128.5 (112.0, 147.5)	-3.8 (-7.5, 1.0)
Maldives	118 (100, 137)	129.2 (112.5, 148.6)	404 (347, 462)	129.2 (111.6, 148.2)	0.0 (-4.7, 4.2)
Mauritius	1041 (895, 1203)	141.7 (123.0, 162.4)	2675 (2305, 3100)	149.3 (129.6, 170.9)	5.4 (1.2, 9.4)
Myanmar	40103 (34408, 46825)	173.5 (150.6, 200.0)	74873 (64641, 85660)	158.5 (138.2, 180.1)	-8.6 (-13.4, -4.2)
Philippines	39438 (34067, 45172)	132.1 (115.1, 151.5)	111374 (96297, 127738)	140.9 (122.9, 161.4)	6.7 (5.9, 7.5)
Seychelles	71 (61, 82)	127.8 (110.9, 146.1)	151 (130, 173)	133.6 (116.2, 153.1)	4.6 (0.3, 8.7)
Sri Lanka	13824 (11888, 15831)	126.8 (109.8, 145.5)	34941 (30261, 40345)	133.8 (117.1, 153.5)	5.5 (1.4, 10.5)
Thailand	46220 (39907, 52816)	129.3 (112.2, 147.3)	121132 (104352, 140681)	116.2 (100.1, 134.4)	-10.1 (-14.2, -5.7)
Timor-Leste	375 (320, 429)	131.6 (113.7, 151.3)	1129 (966, 1301)	136.7 (118.5, 156.2)	3.8 (-1.0, 9.1)

Viet Nam	50980 (43916, 58613)	129.6 (112.6, 147.9)	131496 (113066, 150048)	139.5 (120.8, 159.6)	7.6 (2.9, 13.0)
Oceania	3784 (3262, 4371)	127.8 (110.6, 146.4)	9894 (8500, 11350)	139.2 (120.9, 159.1)	8.9 (6.0, 12.1)
American Samoa	31 (27, 36)	135.0 (117.2, 155.3)	75 (65, 86)	152.9 (132.1, 176.0)	13.3 (8.8, 17.7)
Cook Islands	16 (14, 18)	126.1 (109.3, 143.9)	35 (30, 40)	140.6 (121.4, 161.4)	11.4 (6.7, 16.3)
Fiji	548 (468, 634)	150.3 (130.3, 172.0)	1257 (1081, 1452)	164.0 (142.5, 187.4)	9.1 (5.1, 13.5)
Guam	82 (70, 95)	106.1 (92.2, 122.2)	238 (205, 275)	124.1 (107.4, 142.6)	16.9 (12.7, 21.9)
Kiribati	55 (47, 63)	150.8 (130.8, 172.4)	133 (113, 153)	192.4 (166.6, 220.9)	27.5 (22.4, 33.7)
Marshall Islands	21 (18, 24)	130.5 (112.4, 149.1)	50 (43, 58)	143.2 (124.4, 164.2)	9.8 (5.1, 14.9)
Micronesia (Federated States of)	60 (52, 70)	132.1 (114.6, 151.8)	103 (87, 120)	145.5 (125.7, 166.8)	10.2 (5.6, 14.7)
Nauru	5 (4, 5)	127.3 (110.4, 146.2)	6 (5, 7)	148.6 (127.9, 169.9)	16.8 (12.1, 22.0)
Niue	3 (2, 3)	131.8 (113.8, 150.5)	3 (3, 4)	146.2 (127.2, 167.7)	10.9 (6.4, 16.0)
Northern Mariana Islands	20 (17, 23)	111.1 (95.6, 128.4)	71 (60, 83)	126.6 (109.1, 145.0)	14.0 (9.9, 18.9)
Palau	13 (11, 14)	128.6 (111.2, 147.3)	32 (27, 38)	143.9 (125.1, 164.7)	11.9 (7.2, 17.2)
Papua New Guinea	2240 (1928, 2593)	122.0 (105.5, 139.5)	6328 (5410, 7303)	132.7 (115.3, 152.4)	8.8 (4.4, 13.7)
Samoa	128 (110, 149)	147.8 (128.0, 170.8)	244 (210, 282)	166.8 (144.1, 192.4)	12.9 (8.0, 18.1)
Solomon Islands	173 (148, 200)	128.8 (111.5, 146.6)	445 (380, 512)	145.2 (126.0, 165.7)	12.7 (8.1, 17.4)
Tokelau	2 (2, 2)	135.2 (117.3, 154.4)	2 (2, 2)	146.4 (126.5, 167.7)	8.3 (4.0, 12.7)
Tonga	81 (69, 93)	144.2 (125.0, 165.3)	125 (108, 144)	158.7 (136.3, 181.9)	10.1 (5.5, 14.4)
Tuvalu	9 (8, 11)	138.2 (120.7, 158.4)	16 (13, 18)	151.3 (130.0, 172.9)	9.5 (4.3, 13.9)
Vanuatu	87 (75, 100)	133.5 (114.9, 152.8)	262 (226, 301)	151.1 (131.2, 173.5)	13.2 (7.6, 18.6)
Central Asia	53540 (46486, 61686)	119.0 (103.1, 136.3)	85439 (73678, 98428)	118.2 (102.5, 135.6)	-0.6 (-2.5, 1.4)

Armenia	3661<br>(3161, 4245)	140.1<br>(121.4, 161.7)	5573<br>(4798, 6449)	134.3<br>(116.0, 154.0)	-4.1<br>(-8.0, 0.6)
Azerbaijan	5973<br>(5172, 6885)	125.2<br>(108.6, 143.6)	11280<br>(9713, 13173)	121.4<br>(105.3, 139.3)	-3.0<br>(-7.2, 1.5)
Georgia	18565<br>(15998, 21308)	264.6<br>(228.2, 303.2)	29841<br>(26739, 33256)	131.9<br>(114.0, 152.1)	-30.0<br>(-36.6, -22.3)
Kazakhstan	15360<br>(13316, 17745)	125.4<br>(109.3, 144.0)	20293<br>(17509, 23510)	118.4<br>(102.5, 136.8)	-5.6<br>(-9.8, -1.0)
Kyrgyzstan	3280<br>(2829, 3776)	111.6<br>(96.9, 128.0)	4950<br>(4274, 5717)	111.1<br>(96.1, 128.0)	-0.5<br>(-4.7, 4.0)
Mongolia	1216<br>(1050, 1395)	120.1<br>(104.4, 137.6)	2630<br>(2274, 3025)	116.0<br>(100.6, 133.3)	-3.4<br>(-7.8, 1.1)
Tajikistan	3420<br>(2971, 3918)	126.6<br>(109.0, 144.9)	5621<br>(4767, 6513)	114.9<br>(99.3, 131.8)	-9.2<br>(-13.7, -4.1)
Turkmenistan	2160<br>(1862, 2501)	118.7<br>(102.8, 136.2)	4369<br>(3763, 5026)	114.4<br>(98.6, 131.0)	-3.6<br>(-7.6, 1.1)
Uzbekistan	11051<br>(9510, 12792)	102.6<br>(88.6, 118.6)	22887<br>(19239, 26680)	112.4<br>(97.2, 128.9)	9.6<br>(4.7, 14.6)
Central Europe	205182<br>(177091, 235330)	138.9<br>(120.2, 158.8)	262490<br>(225127, 303309)	124.1<br>(107.3, 142.0)	-10.7<br>(-11.7, -9.7)
Albania	2498<br>(2162, 2875)	122.5<br>(106.0, 140.9)	5056<br>(4327, 5826)	117.5<br>(101.5, 134.0)	-4.1<br>(-8.1, 0.2)
Bosnia and Herzegovina	5457<br>(4673, 6342)	136.0<br>(117.7, 155.8)	8602<br>(7361, 9906)	143.8<br>(124.0, 164.6)	5.7<br>(1.2, 10.4)
Bulgaria	16664<br>(14242, 19327)	132.1<br>(113.9, 152.0)	17974<br>(15409, 20880)	125.3<br>(108.0, 144.1)	-5.2<br>(-9.1, -1.2)
Croatia	9234<br>(7964, 10727)	143.8<br>(124.5, 165.8)	10119<br>(8721, 11756)	119.0<br>(102.8, 136.6)	-17.2<br>(-21.1, -13.3)
Czechia	18721<br>(16192, 21497)	135.7<br>(117.9, 156.1)	25704<br>(21952, 29954)	125.0<br>(107.8, 143.9)	-7.9<br>(-11.6, -3.9)
Hungary	22233<br>(19193, 25476)	150.4<br>(130.3, 171.7)	25257<br>(21741, 29366)	133.3<br>(116.1, 153.1)	-11.4<br>(-15.2, -7.4)
North Macedonia	2529<br>(2174, 2920)	136.0<br>(117.2, 156.3)	4291<br>(3666, 4974)	131.9<br>(114.2, 151.4)	-3.1<br>(-7.3, 1.3)
Montenegro	909<br>(788, 1046)	147.1<br>(127.5, 168.9)	1436<br>(1237, 1653)	145.1<br>(125.3, 166.3)	-1.3<br>(-5.4, 2.9)
Poland	65584<br>(56729, 75141)	150.4<br>(130.1, 171.2)	85157<br>(73120, 98501)	123.9<br>(107.5, 142.0)	-17.6<br>(-18.8, -16.4)
Romania	35182<br>(30049, 40517)	125.6<br>(107.9, 143.8)	42658<br>(36548, 49232)	116.4<br>(100.8, 133.3)	-7.3<br>(-11.6, -3.1)
Serbia	14873<br>(12602, 17298)	130.4<br>(112.4, 149.0)	21166<br>(18052, 24604)	133.2<br>(116.1, 153.2)	2.2<br>(-2.3, 6.5)

Slovakia	8156<br>(7025, 9370)	136.8<br>(118.0, 156.8)	10317<br>(8811, 11884)	112.0<br>(96.5, 127.9)	-18.1<br>(-21.7, -14.3)
Slovenia	3142<br>(2721, 3646)	128.9<br>(112.1, 148.9)	4752<br>(4113, 5493)	114.5<br>(99.6, 132.3)	-11.2<br>(-15.0, -7.2)
Eastern Europe	436405<br>(377566, 500222)	156.7<br>(135.4, 178.4)	530342<br>(458792, 609014)	153.7<br>(133.6, 175.5)	-1.9<br>(-3.3, -0.6)
Belarus	18608<br>(15877, 21465)	143.9<br>(124.3, 165.3)	21964<br>(18929, 25332)	138.8<br>(120.5, 159.4)	-3.5<br>(-8.0, 0.8)
Estonia	2968<br>(2555, 3436)	144.7<br>(124.7, 166.6)	3424<br>(2961, 3958)	135.9<br>(117.5, 155.8)	-6.1<br>(-10.3, -1.5)
Latvia	4610<br>(4001, 5341)	128.5<br>(111.7, 148.8)	5160<br>(4445, 5981)	134.5<br>(116.2, 154.9)	4.7<br>(-0.1, 9.9)
Lithuania	6354<br>(5495, 7374)	140.9<br>(121.1, 163.7)	7170<br>(6195, 8300)	129.5<br>(111.5, 149.5)	-8.1<br>(-12.4, -3.8)
Republic of Moldova	5668<br>(4879, 6528)	129.9<br>(113.1, 148.4)	7973<br>(6820, 9239)	137.8<br>(118.5, 158.4)	6.0<br>(1.3, 11.1)
Russian Federation	283113<br>(244973, 326193)	158.0<br>(136.7, 181.0)	364732<br>(314648, 420894)	154.9<br>(134.0, 177.4)	-1.9<br>(-2.8, -1.1)
Ukraine	115084<br>(99092, 131283)	160.4<br>(138.6, 181.9)	119920<br>(103832, 137658)	157.5<br>(136.9, 179.5)	-1.8<br>(-6.6, 3.0)
High-income Asia Pacific	388227<br>(338423, 442137)	190.3<br>(166.6, 216.0)	489495<br>(426049, 565312)	115.5<br>(100.5, 131.8)	-39.3<br>(-40.2, -38.4)
Brunei Darussalam	178<br>(154, 202)	186.6<br>(162.8, 213.8)	400<br>(343, 460)	131.2<br>(114.2, 150.4)	-29.7<br>(-32.6, -26.7)
Japan	332924<br>(291379, 379153)	193.2<br>(169.4, 219.4)	382153<br>(332674, 442062)	118.0<br>(102.4, 135.1)	-39.0<br>(-39.8, -38.2)
Republic of Korea	51691<br>(44587, 59198)	178.1<br>(154.8, 203.3)	99178<br>(85614, 114035)	110.2<br>(95.2, 126.2)	-38.1<br>(-41.1, -35.1)
Singapore	3434<br>(2980, 3939)	156.7<br>(135.8, 181.0)	7765<br>(6703, 8955)	98.0<br>(85.0, 112.7)	-37.5<br>(-39.9, -34.8)
Australasia	41594<br>(35951, 47669)	176.2<br>(153.0, 200.4)	54525<br>(46977, 62630)	113.3<br>(98.3, 130.1)	-35.7<br>(-37.9, -33.1)
Australia	34391<br>(29575, 39395)	174.7<br>(152.0, 199.2)	45835<br>(39534, 52648)	113.2<br>(97.9, 129.8)	-35.2<br>(-37.9, -32.2)
New Zealand	7203<br>(6241, 8263)	183.6<br>(160.3, 210.1)	8689<br>(7488, 9989)	114.1<br>(98.8, 130.6)	-37.9<br>(-40.7, -35.4)
Western Europe	1390569<br>(1222539, 1579639)	244.8<br>(214.7, 277.9)	1393111<br>(1215264, 1597268)	165.2<br>(143.3, 189.1)	-32.5<br>(-33.6, -31.5)
Andorra	119<br>(103, 138)	210.1<br>(182.6, 239.8)	219<br>(190, 249)	156.8<br>(135.7, 179.1)	-25.4<br>(-28.7, -22.0)
Austria	26225<br>(22736, 29889)	226.3<br>(197.4, 256.3)	27965<br>(24324, 32064)	167.7<br>(145.2, 191.8)	-25.9<br>(-29.3, -22.2)

Belgium	36815 (32113, 42059)	243.9 (214.1, 276.7)	38579 (33524, 44772)	183.0 (159.1, 211.1)	-25.0 (-28.3, -21.7)
Cyprus	2078 (1799, 2379)	244.1 (213.8, 276.3)	3683 (3164, 4260)	189.0 (162.8, 217.1)	-22.6 (-25.8, -19.1)
Denmark	25893 (22713, 29304)	332.5 (292.3, 375.6)	24444 (20923, 28170)	224.9 (194.6, 257.5)	-32.4 (-35.3, -29.2)
Finland	17061 (14923, 19629)	239.9 (210.5, 275.0)	18704 (16009, 21672)	163.0 (141.6, 186.0)	-32.1 (-35.1, -28.8)
France	183510 (160444, 208980)	227.8 (199.8, 261.1)	200516 (172867, 231304)	159.6 (137.0, 183.6)	-29.9 (-33.3, -26.6)
Germany	309841 (270719, 351553)	251.2 (219.8, 284.3)	289033 (251628, 332268)	165.2 (143.4, 190.5)	-34.2 (-36.9, -31.2)
Greece	37744 (32581, 43051)	248.7 (216.1, 282.9)	38611 (33510, 44657)	180.4 (155.0, 207.4)	-27.5 (-30.8, -24.5)
Iceland	669 (586, 759)	239.7 (210.4, 270.8)	866 (750, 1000)	162.8 (141.3, 187.1)	-32.1 (-35.3, -28.8)
Ireland	11142 (9722, 12704)	271.8 (238.9, 307.4)	13174 (11403, 15135)	179.4 (155.4, 205.3)	-34.0 (-36.9, -31.0)
Israel	11641 (10039, 13311)	239.4 (207.8, 273.2)	20089 (17306, 23237)	177.2 (154.2, 203.7)	-26.0 (-29.1, -23.2)
Italy	225747 (197450, 257479)	256.6 (224.6, 289.7)	220872 (192590, 251583)	169.5 (146.8, 192.4)	-34.0 (-34.9, -32.9)
Luxembourg	1345 (1180, 1541)	248.6 (218.5, 283.5)	1729 (1508, 1993)	179.9 (156.3, 206.9)	-27.7 (-30.8, -24.7)
Malta	1145 (1002, 1303)	263.1 (231.4, 299.2)	1707 (1460, 1986)	191.7 (165.9, 219.0)	-27.1 (-30.4, -23.6)
Monaco	151 (132, 172)	230.2 (201.5, 260.3)	142 (123, 164)	163.2 (140.9, 186.6)	-29.1 (-32.2, -25.9)
Netherlands	47603 (42279, 53208)	242.1 (215.4, 269.8)	54089 (46692, 62540)	165.8 (144.2, 191.3)	-31.5 (-36.1, -26.2)
Norway	16080 (14002, 18347)	246.0 (215.1, 278.6)	15869 (13791, 18247)	174.0 (151.2, 198.8)	-29.3 (-30.3, -28.3)
Portugal	32695 (28236, 37425)	232.1 (202.8, 264.4)	37947 (32744, 43571)	170.1 (146.8, 194.9)	-26.7 (-29.9, -22.4)
Spain	123551 (107319, 142557)	226.8 (198.8, 259.6)	140759 (122901, 161423)	158.4 (137.5, 182.2)	-30.2 (-33.2, -27.1)
Sweden	34923 (30599, 39842)	242.0 (212.5, 274.6)	33364 (28824, 38712)	172.9 (149.6, 198.7)	-28.5 (-31.0, -25.9)
Switzerland	23193 (20230, 26463)	230.3 (200.2, 263.4)	26930 (23278, 31209)	166.5 (143.8, 192.1)	-27.7 (-31.1, -24.8)
United Kingdom	220166 (192422, 249951)	249.0 (217.9, 281.4)	182505 (158815, 209020)	153.9 (133.5, 175.6)	-38.2 (-38.9, -37.4)

Southern Latin America	90943 (78778, 104700)	196.1 (170.3, 224.0)	120461 (104212, 138563)	145.0 (125.3, 166.7)	-26.1 (-28.5, -23.2)
Argentina	63315 (54630, 73271)	194.7 (169.6, 224.3)	78132 (67173, 90212)	145.2 (125.0, 167.7)	-25.4 (-28.8, -21.4)
Chile	19116 (16610, 21711)	192.3 (167.0, 218.4)	34104 (29370, 39125)	141.8 (122.4, 162.7)	-26.2 (-29.5, -22.5)
Uruguay	8508 (7395, 9744)	217.5 (190.0, 247.5)	8219 (7134, 9371)	157.8 (136.5, 179.9)	-27.5 (-30.5, -24.4)
High-income North America	944244 (818052, 1078722)	271.8 (235.8, 308.9)	1191366 (1063013, 1335557)	193.5 (173.7, 215.6)	-28.8 (-33.1, -23.8)
Canada	101410 (88677, 114888)	311.9 (274.5, 351.6)	126540 (109188, 145389)	189.9 (165.0, 215.8)	-39.1 (-42.0, -36.1)
United States of America	842705 (730147, 964536)	267.5 (231.8, 304.4)	1064669 (951501, 1189965)	193.9 (174.5, 215.5)	-27.5 (-32.3, -21.6)
Greenland	107 (94, 122)	312.9 (275.5, 352.6)	139 (119, 161)	197.8 (172.4, 226.9)	-36.8 (-39.3, -34.2)
Caribbean	27342 (23488, 31485)	106.0 (91.3, 122.1)	51482 (44448, 59163)	99.3 (85.8, 114.2)	-6.3 (-8.0, -4.1)
Antigua and Barbuda	54 (46, 62)	101.6 (87.6, 117.0)	97 (83, 112)	95.4 (82.1, 109.8)	-6.1 (-10.5, -1.7)
Bahamas	145 (125, 167)	97.2 (83.7, 112.6)	360 (311, 416)	93.2 (80.4, 107.5)	-4.1 (-8.4, 0.3)
Barbados	294 (251, 339)	101.4 (87.4, 116.5)	481 (413, 556)	97.3 (84.4, 112.0)	-4.1 (-8.4, 1.0)
Belize	93 (81, 107)	102.7 (88.7, 118.3)	266 (229, 306)	98.8 (85.3, 113.9)	-3.8 (-7.8, 0.9)
Bermuda	59 (50, 68)	94.1 (80.8, 108.7)	114 (98, 131)	88.5 (76.2, 102.0)	-6.0 (-9.8, -1.9)
Cuba	11472 (9925, 13283)	111.7 (96.6, 129.1)	19094 (16440, 21899)	100.6 (86.1, 115.8)	-9.9 (-13.6, -5.4)
Dominica	72 (62, 85)	101.1 (87.2, 117.1)	85 (74, 99)	94.9 (81.7, 110.0)	-6.1 (-10.0, -2.1)
Dominican Republic	3810 (3289, 4380)	105.5 (90.6, 120.9)	9217 (7973, 10599)	100.5 (87.0, 116.3)	-4.7 (-8.8, -0.3)
Grenada	75 (64, 87)	104.8 (89.6, 120.7)	113 (96, 130)	100.9 (86.6, 115.7)	-3.7 (-8.5, 0.6)
Guyana	386 (333, 442)	105.9 (91.4, 121.1)	624 (535, 726)	101.8 (88.5, 117.5)	-3.9 (-8.3, 0.8)
Haiti	3117 (2673, 3616)	100.7 (87.6, 116.0)	6372 (5436, 7365)	95.4 (81.7, 109.8)	-5.3 (-9.2, -0.9)
Jamaica	1796 (1540, 2061)	101.5 (87.2, 116.6)	3201 (2757, 3682)	108.0 (92.7, 124.7)	6.4 (1.8, 11.1)

Puerto Rico	3532 (3026, 4086)	96.7 (83.2, 111.7)	6502 (5614, 7553)	93.1 (80.5, 107.8)	-3.7 (-7.8, 0.6)
Saint Kitts and Nevis	39 (33, 46)	102.6 (88.6, 119.0)	64 (54, 74)	96.9 (83.3, 111.8)	-5.6 (-9.7, -0.8)
Saint Lucia	95 (81, 111)	108.7 (93.7, 125.4)	217 (187, 250)	101.0 (86.7, 116.9)	-7.1 (-10.7, -3.0)
Saint Vincent and the Grenadines	75 (64, 87)	104.7 (89.8, 120.1)	138 (119, 159)	101.9 (87.8, 116.4)	-2.7 (-7.2, 1.6)
Suriname	266 (230, 305)	105.2 (90.7, 120.8)	636 (550, 732)	106.7 (92.0, 122.4)	1.4 (-2.9, 6.8)
Trinidad and Tobago	973 (834, 1121)	116.4 (100.3, 133.7)	1986 (1699, 2310)	105.9 (91.5, 123.0)	-9.0 (-13.1, -4.4)
United States Virgin Islands	79 (68, 91)	93.9 (80.8, 108.1)	172 (146, 199)	90.6 (78.3, 103.9)	-3.5 (-8.1, 0.7)
Andean Latin America	17571 (15177, 20108)	88.7 (76.4, 101.5)	46920 (40588, 53969)	84.5 (73.1, 96.9)	-4.7 (-7.7, -2.0)
Bolivia (Plurinational State of)	3066 (2643, 3520)	99.1 (85.6, 114.6)	7861 (6776, 9007)	90.1 (77.9, 102.9)	-9.1 (-13.2, -4.5)
Ecuador	4834 (4178, 5546)	93.4 (80.7, 107.1)	13195 (11327, 15191)	87.9 (75.8, 100.9)	-5.9 (-9.7, -2.0)
Peru	9671 (8332, 11074)	83.8 (72.0, 95.5)	25863 (22326, 29692)	81.3 (69.9, 93.6)	-2.9 (-7.5, 1.4)
Central Latin America	99343 (85825, 113998)	122.5 (105.7, 141.2)	245047 (211434, 281535)	104.5 (90.2, 120.4)	-14.6 (-15.9, -13.4)
Colombia	20353 (17441, 23519)	120.0 (103.0, 138.9)	51620 (44522, 59637)	98.0 (84.2, 113.1)	-18.3 (-22.1, -14.7)
Costa Rica	1958 (1687, 2249)	114.9 (98.8, 132.5)	5085 (4384, 5856)	99.3 (85.3, 114.1)	-13.6 (-17.5, -9.5)
El Salvador	3005 (2596, 3444)	104.3 (90.4, 119.9)	5885 (5062, 6769)	99.9 (85.8, 115.1)	-4.3 (-8.9, 1.2)
Guatemala	3614 (3100, 4172)	103.0 (89.2, 118.5)	11088 (9517, 12789)	102.0 (87.6, 117.8)	-1.0 (-5.2, 3.8)
Honduras	2296 (1974, 2659)	115.7 (99.6, 133.5)	6410 (5539, 7378)	107.8 (93.2, 124.3)	-6.8 (-11.2, -1.3)
Mexico	52832 (45676, 60508)	126.5 (109.5, 145.8)	126072 (108915, 144836)	108.3 (93.5, 124.6)	-14.4 (-15.6, -13.3)
Nicaragua	1683 (1456, 1944)	114.8 (99.1, 132.7)	4575 (3950, 5258)	105.6 (91.1, 121.4)	-8.0 (-12.2, -3.7)
Panama	1551 (1331, 1774)	105.9 (90.8, 121.6)	3922 (3379, 4520)	95.0 (81.7, 110.1)	-10.3 (-14.2, -6.7)
Venezuela (Bolivarian Republic of)	12051 (10437, 13828)	128.8 (111.7, 147.4)	30388 (25851, 35064)	104.5 (89.8, 120.7)	-18.8 (-22.7, -14.0)

Tropical Latin America	105614 (91130, 121097)	118.4 (102.1, 135.5)	229885 (198050, 263869)	95.0 (82.1, 108.8)	-19.8 (-21.0, -18.6)
Brazil	103137 (88949, 118284)	118.5 (102.3, 135.6)	224008 (192996, 257137)	94.7 (81.9, 108.4)	-20.1 (-21.3, -18.9)
Paraguay	2477 (2130, 2846)	114.3 (98.3, 131.6)	5877 (5057, 6772)	107.5 (92.8, 124.3)	-6.0 (-10.3, -1.3)
North Africa and Middle East	185982 (160214, 214589)	111.5 (96.4, 127.9)	470128 (407448, 537521)	109.7 (95.0, 125.8)	-1.6 (-2.9, -0.3)
Afghanistan	7086 (6078, 8167)	101.1 (88.0, 115.8)	13378 (11479, 15318)	111.6 (96.9, 127.0)	10.4 (5.4, 15.5)
Algeria	13496 (11500, 15776)	111.4 (95.9, 128.3)	36813 (31831, 42159)	109.9 (94.9, 125.8)	-1.3 (-5.4, 2.8)
Bahrain	200 (172, 229)	112.6 (97.5, 130.1)	1223 (1038, 1425)	110.1 (95.0, 126.8)	-2.3 (-6.5, 2.1)
Egypt	29697 (25599, 34272)	104.7 (91.0, 119.6)	69881 (59921, 81408)	111.2 (95.7, 128.8)	6.2 (1.6, 11.2)
Iran (Islamic Republic of)	26707 (22830, 30950)	105.5 (91.5, 120.7)	80255 (69882, 91879)	110.7 (95.9, 127.0)	4.9 (3.7, 6.2)
Iraq	10182 (8822, 11662)	134.2 (116.0, 154.4)	27561 (23774, 31488)	121.3 (105.1, 139.7)	-9.6 (-13.7, -5.7)
Jordan	1554 (1330, 1790)	119.4 (102.1, 137.5)	7741 (6660, 8923)	117.1 (100.9, 134.6)	-1.9 (-6.0, 2.9)
Kuwait	610 (523, 698)	99.8 (86.9, 113.8)	2849 (2421, 3261)	102.6 (88.5, 118.1)	2.7 (-1.5, 7.2)
Lebanon	2868 (2454, 3319)	126.6 (110.0, 145.6)	6750 (5835, 7779)	130.3 (112.6, 151.0)	2.9 (-1.9, 7.3)
Libya	1863 (1613, 2122)	102.9 (89.1, 117.4)	5559 (4839, 6345)	109.6 (94.9, 125.6)	6.6 (2.1, 11.0)
Morocco	14740 (12666, 17029)	110.7 (95.8, 126.7)	33771 (29103, 38960)	108.9 (94.1, 125.2)	-1.6 (-5.7, 2.8)
Palestine	926 (792, 1061)	110.0 (94.8, 125.7)	2557 (2215, 2940)	109.4 (94.9, 124.7)	-0.5 (-4.9, 4.0)
Oman	658 (564, 756)	102.1 (88.6, 117.0)	2008 (1711, 2322)	110.5 (94.9, 127.3)	8.3 (4.1, 13.5)
Qatar	140 (118, 163)	111.2 (96.2, 128.2)	1145 (958, 1350)	97.6 (84.6, 112.5)	-12.2 (-16.1, -8.3)
Saudi Arabia	5260 (4545, 5993)	91.7 (79.2, 105.4)	17163 (14537, 19762)	93.1 (80.4, 107.5)	1.6 (-3.5, 5.7)
Sudan	8160 (7032, 9413)	90.7 (78.7, 103.8)	17396 (14994, 19882)	95.8 (82.5, 109.3)	5.7 (0.7, 10.1)
Syrian Arab Republic	5701 (4906, 6613)	112.2 (97.0, 130.1)	13404 (11450, 15422)	107.3 (92.7, 123.3)	-4.4 (-8.0, 0.0)

Tunisia	5554<br>(4735, 6415)	111.8<br>(96.5, 128.6)	14331<br>(12428, 16529)	113.2<br>(98.6, 129.8)	1.3<br>(-3.5, 6.0)
Türkiye	45181<br>(38848, 51980)	128.8<br>(111.2, 147.9)	97349<br>(84428, 111440)	110.0<br>(95.5, 126.1)	-14.6<br>(-18.3, -10.5)
United Arab Emirates	452<br>(379, 525)	98.1<br>(84.3, 112.6)	4719<br>(3924, 5598)	99.5<br>(86.0, 115.2)	1.4<br>(-3.2, 6.2)
Yemen	4823<br>(4155, 5530)	103.4<br>(89.7, 117.7)	13801<br>(11861, 15817)	107.2<br>(92.6, 123.4)	3.7<br>(-0.4, 8.9)
South Asia	506897<br>(434634, 583458)	93.6<br>(80.8, 107.3)	1286587<br>(1106373, 1476216)	93.1<br>(80.5, 106.5)	-0.6<br>(-1.6, 0.3)
Bangladesh	39150<br>(33617, 45168)	87.5<br>(75.1, 100.7)	109950<br>(94512, 125913)	85.1<br>(73.5, 97.5)	-2.7<br>(-6.5, 1.5)
Bhutan	206<br>(178, 238)	89.3<br>(77.3, 102.5)	474<br>(409, 545)	85.8<br>(74.1, 98.4)	-4.0<br>(-8.3, 0.6)
India	401803<br>(344112, 463738)	93.2<br>(80.7, 106.8)	1043339<br>(895724, 1197112)	92.9<br>(80.3, 106.4)	-0.3<br>(-1.3, 0.7)
Nepal	8604<br>(7329, 9904)	95.7<br>(82.9, 109.7)	20545<br>(17645, 23648)	93.5<br>(80.6, 107.4)	-2.3<br>(-6.4, 2.3)
Pakistan	57134<br>(49480, 65489)	102.2<br>(88.3, 117.2)	112280<br>(96692, 128654)	104.6<br>(90.5, 119.6)	2.3<br>(-0.5, 5.2)
Central Sub-Saharan Africa	24145<br>(20560, 28036)	111.3<br>(96.3, 127.8)	56336<br>(48531, 64854)	111.4<br>(96.6, 127.6)	0.1<br>(-3.1, 3.5)
Angola	4189<br>(3593, 4804)	114.4<br>(99.0, 130.8)	12909<br>(11078, 14865)	119.7<br>(103.4, 137.7)	4.7<br>(-0.3, 9.1)
Central African Republic	1251<br>(1066, 1454)	114.9<br>(99.7, 132.2)	2372<br>(2038, 2736)	118.3<br>(102.7, 135.3)	3.0<br>(-1.5, 8.0)
Congo	1216<br>(1035, 1401)	118.7<br>(101.9, 135.7)	2884<br>(2487, 3306)	113.8<br>(98.4, 131.2)	-4.1<br>(-8.1, 0.2)
Democratic Republic of the Congo	16685<br>(14098, 19390)	109.8<br>(94.9, 126.6)	36526<br>(31335, 42135)	108.2<br>(93.5, 124.4)	-1.5<br>(-5.8, 3.3)
Equatorial Guinea	218<br>(186, 253)	115.9<br>(100.8, 132.7)	511<br>(443, 585)	109.9<br>(94.9, 126.2)	-5.1<br>(-10.1, 0.1)
Gabon	587<br>(500, 679)	107.9<br>(93.1, 123.8)	1133<br>(978, 1298)	110.8<br>(95.5, 127.8)	2.7<br>(-1.4, 7.7)
Eastern Sub-Saharan Africa	68324<br>(58748, 78605)	95.9<br>(82.9, 109.7)	153080<br>(132225, 175448)	97.8<br>(84.4, 111.9)	2.0<br>(0.8, 3.4)
Burundi	2228<br>(1918, 2552)	99.8<br>(86.5, 114.0)	4255<br>(3686, 4904)	98.3<br>(84.9, 112.4)	-1.5<br>(-6.4, 3.3)
Comoros	210<br>(180, 243)	97.4<br>(84.5, 112.5)	471<br>(405, 542)	98.8<br>(85.1, 114.1)	1.5<br>(-2.8, 5.9)
Djibouti	127<br>(108, 147)	95.9<br>(82.8, 110.6)	578<br>(497, 662)	99.4<br>(86.0, 113.6)	3.6<br>(-0.7, 7.9)

Eritrea	865<br>(738, 999)	96.8<br>(83.4, 111.3)	2389<br>(2042, 2755)	95.8<br>(82.6, 109.2)	-1.0<br>(-5.5, 3.6)
Ethiopia	16070<br>(13723, 18649)	86.6<br>(74.6, 99.2)	34059<br>(29348, 38874)	85.5<br>(73.6, 98.0)	-1.3<br>(-3.4, 0.8)
Kenya	7946<br>(6851, 9129)	100.2<br>(86.7, 114.8)	21502<br>(18600, 24698)	101.2<br>(87.7, 115.7)	1.0<br>(0.1, 1.9)
Madagascar	4582<br>(3938, 5308)	93.6<br>(81.0, 107.4)	10006<br>(8623, 11579)	96.6<br>(83.4, 110.9)	3.2<br>(-1.1, 7.6)
Malawi	4141<br>(3539, 4763)	111.7<br>(97.0, 128.0)	7553<br>(6495, 8647)	108.3<br>(93.5, 123.5)	-3.0<br>(-7.3, 0.9)
Mozambique	6143<br>(5278, 7107)	106.4<br>(91.9, 121.7)	11604<br>(10024, 13299)	110.7<br>(95.7, 126.2)	4.1<br>(0.1, 8.6)
Rwanda	2907<br>(2495, 3349)	106.0<br>(92.1, 121.5)	6117<br>(5223, 7074)	105.7<br>(90.9, 121.6)	-0.3<br>(-4.4, 4.9)
Somalia	2348<br>(2023, 2705)	100.2<br>(86.6, 114.2)	6586<br>(5634, 7600)	106.1<br>(91.8, 121.3)	5.9<br>(1.6, 11.1)
South Sudan	1963<br>(1685, 2261)	85.8<br>(74.3, 98.8)	3553<br>(3066, 4068)	94.5<br>(81.9, 108.7)	10.1<br>(5.4, 14.8)
Uganda	5911<br>(5066, 6769)	95.4<br>(82.7, 108.8)	13114<br>(11277, 15056)	97.5<br>(83.7, 111.9)	2.2<br>(-2.6, 7.2)
United Republic of Tanzania	10120<br>(8662, 11656)	96.6<br>(83.0, 110.2)	24810<br>(21513, 28394)	104.1<br>(90.8, 119.6)	7.8<br>(3.2, 12.6)
Zambia	2713<br>(2332, 3103)	99.5<br>(86.3, 114.3)	6361<br>(5499, 7252)	99.8<br>(85.7, 114.3)	0.2<br>(-4.1, 4.5)
Southern Sub-Saharan Africa	38316<br>(33103, 44033)	142.4<br>(123.0, 163.5)	69933<br>(60300, 80477)	126.2<br>(109.5, 144.8)	-11.4<br>(-12.9, -9.8)
Botswana	697<br>(594, 810)	125.3<br>(108.3, 143.8)	1653<br>(1421, 1911)	123.5<br>(106.5, 142.5)	-1.5<br>(-5.6, 3.2)
Lesotho	1193<br>(1029, 1371)	124.5<br>(108.4, 142.2)	1647<br>(1406, 1901)	134.2<br>(116.0, 153.6)	7.8<br>(2.9, 12.6)
Namibia	897<br>(761, 1042)	125.5<br>(108.3, 144.4)	1639<br>(1420, 1881)	118.9<br>(103.0, 137.2)	-5.3<br>(-9.5, -0.5)
South Africa	30455<br>(26341, 35015)	149.2<br>(128.7, 171.7)	55344<br>(47710, 63746)	125.4<br>(108.8, 143.8)	-16.0<br>(-17.9, -14.3)
Eswatini	369<br>(321, 422)	130.5<br>(113.5, 148.9)	751<br>(644, 865)	133.6<br>(115.6, 154.3)	2.4<br>(-2.0, 7.4)
Zimbabwe	4704<br>(4036, 5453)	119.1<br>(103.1, 136.6)	8899<br>(7629, 10298)	131.7<br>(114.5, 152.4)	10.6<br>(5.2, 15.7)
Western Sub-Saharan Africa	75571<br>(64941, 86985)	89.7<br>(77.6, 102.7)	170050<br>(146947, 195012)	93.5<br>(81.0, 107.2)	4.3<br>(3.5, 5.0)
Benin	1894<br>(1621, 2167)	98.3<br>(84.6, 112.6)	4714<br>(4060, 5442)	100.4<br>(86.8, 116.0)	2.1<br>(-2.4, 7.2)

Burkina Faso	3831<br>(3275, 4426)	90.9<br>(78.5, 104.2)	8166<br>(7052, 9367)	93.1<br>(80.4, 106.1)	2.4<br>(-1.6, 7.0)
Cameroon	3764<br>(3222, 4368)	88.3<br>(76.1, 102.2)	10759<br>(9264, 12393)	94.3<br>(81.9, 108.5)	6.8<br>(2.4, 11.7)
Cabo Verde	220<br>(189, 253)	97.0<br>(83.5, 111.8)	409<br>(356, 470)	98.3<br>(84.4, 113.3)	1.4<br>(-3.1, 6.5)
Chad	2397<br>(2069, 2762)	87.8<br>(76.1, 100.8)	4851<br>(4207, 5577)	90.1<br>(77.9, 103.0)	2.7<br>(-1.8, 7.4)
Cote d'Ivoire	3665<br>(3147, 4234)	96.3<br>(83.4, 110.4)	10218<br>(8816, 11739)	100.1<br>(86.9, 114.7)	4.0<br>(-0.2, 8.8)
Gambia	343<br>(295, 396)	101.6<br>(87.1, 117.3)	969<br>(838, 1109)	104.7<br>(91.0, 119.9)	3.1<br>(-1.5, 8.0)
Ghana	5694<br>(4887, 6554)	95.3<br>(82.5, 108.9)	15191<br>(13150, 17544)	97.6<br>(84.9, 112.3)	2.4<br>(-1.9, 6.6)
Guinea	2790<br>(2391, 3227)	86.5<br>(74.7, 99.9)	4781<br>(4115, 5464)	90.2<br>(77.8, 103.0)	4.2<br>(-0.3, 8.7)
Guinea-Bissau	373<br>(321, 434)	97.2<br>(84.2, 112.2)	670<br>(577, 768)	98.8<br>(85.5, 112.7)	1.6<br>(-3.0, 5.7)
Liberia	1091<br>(932, 1253)	100.2<br>(86.4, 114.6)	2060<br>(1777, 2353)	103.6<br>(89.4, 119.5)	3.3<br>(-0.8, 8.0)
Mali	3540<br>(3006, 4101)	89.4<br>(77.3, 102.6)	7697<br>(6613, 8857)	93.1<br>(80.3, 106.9)	4.2<br>(-0.2, 8.6)
Mauritania	889<br>(759, 1031)	91.7<br>(79.1, 105.6)	1935<br>(1675, 2230)	95.0<br>(82.3, 109.2)	3.7<br>(-0.3, 8.0)
Niger	2304<br>(1980, 2668)	87.3<br>(75.2, 100.9)	6861<br>(5886, 7891)	92.3<br>(79.4, 105.4)	5.8<br>(1.3, 10.0)
Nigeria	36445<br>(31345, 41954)	86.2<br>(74.6, 98.7)	76282<br>(65942, 87623)	90.3<br>(78.1, 103.7)	4.7<br>(3.7, 5.7)
Sao Tome and Principe	60<br>(51, 70)	95.2<br>(82.1, 110.3)	101<br>(87, 116)	98.5<br>(84.9, 113.4)	3.4<br>(-0.9, 8.0)
Senegal	3090<br>(2655, 3557)	98.9<br>(85.2, 113.4)	7191<br>(6193, 8242)	98.7<br>(85.1, 112.8)	-0.2<br>(-4.2, 4.2)
Sierra Leone	2025<br>(1743, 2320)	108.3<br>(93.9, 124.5)	3644<br>(3169, 4184)	105.3<br>(91.5, 120.8)	-2.8<br>(-6.9, 1.6)
Togo	1152<br>(992, 1334)	97.3<br>(84.0, 112.8)	3548<br>(3058, 4098)	100.9<br>(87.3, 116.7)	3.8<br>(-0.6, 8.2)
High SDI	2415146<br>(2106717, 2750980)	232.2<br>(203.2, 263.3)	2855740<br>(2518228, 3241195)	157.7<br>(139.5, 178.5)	-32.1<br>(-33.9, -30.2)
High-middle SDI	1671377<br>(1447418, 1911682)	157.1<br>(136.8, 179.4)	2745997<br>(2383531, 3147380)	133.6<br>(116.2, 153.1)	-15.0<br>(-16.0, -14.1)
Middle SDI	1206340<br>(1042406, 1379271)	118.7<br>(103.1, 136.0)	2915099<br>(2520526, 3342399)	114.7<br>(99.6, 131.5)	-3.4<br>(-4.3, -2.4)

Low-middle SDI	611734<br>(526686, 701868)	105.4<br>(91.2, 120.7)	1398309<br>(1206028, 1602467)	103.2<br>(89.5, 118.2)	-2.1<br>(-3.0, -1.1)
Low SDI	218718<br>(187730, 252085)	96.9<br>(83.9, 111.1)	470118<br>(404625, 538077)	94.1<br>(81.5, 107.4)	-2.9<br>(-3.8, -1.9)

Table S5: YLDs of PAD in 1990 and 2019 for both sexes and percentage change in age-standardised rates by location. PAD= Peripheral artery disease. YLDs=years lived with disability.

YLDs of PAD	1990		2019		Percentage change in age-standardised rates between 1990 and 2019
	Counts (95% UI)	Age-standardised rate (95% UI)	Counts (95% UI)	Age-standardised rate (95% UI)	
Global	302821 (141753, 539467)	8.6 (4.0, 15.3)	500893 (234625, 898104)	6.3 (3.0, 11.3)	-26.5 (-27.7, -25.2)
East Asia	57916 (26757, 102465)	8.0 (3.7, 14.2)	135332 (61923, 243445)	6.9 (3.2, 12.5)	-13.4 (-17.8, -8.4)
China	55800 (25795, 98808)	8.0 (3.7, 14.2)	130060 (59469, 234455)	6.9 (3.2, 12.5)	-13.6 (-18.1, -8.4)
Democratic People's Republic of Korea	983 (456, 1792)	7.8 (3.7, 14.1)	2367 (1103, 4335)	7.8 (3.6, 14.3)	0.9 (-7.4, 9.8)
Taiwan	1133 (521, 2064)	8.2 (3.8, 14.7)	2904 (1359, 5243)	7.2 (3.3, 12.9)	-12.2 (-20.9, -2.1)
Southeast Asia	18282 (8593, 32209)	8.7 (4.1, 15.6)	42887 (20031, 76413)	8.1 (3.8, 14.7)	-6.4 (-9.4, -2.6)
Cambodia	342 (159, 615)	9.5 (4.4, 17.0)	824 (387, 1451)	8.1 (3.8, 14.4)	-14.3 (-21.6, -5.5)
Indonesia	6793 (3136, 11986)	8.9 (4.3, 16.0)	16124 (7535, 28639)	9.2 (4.3, 16.8)	4.1 (0.0, 9.6)
Lao People's Democratic Republic	171 (80, 308)	10.1 (4.8, 18.4)	309 (143, 551)	8.7 (4.1, 15.8)	-13.3 (-20.6, -3.6)
Malaysia	642 (302, 1141)	8.3 (3.9, 15.0)	1707 (790, 3114)	7.2 (3.4, 12.9)	-14.0 (-20.4, -6.9)
Maldives	6 (3, 10)	8.1 (3.8, 14.5)	17 (8, 30)	6.9 (3.2, 12.4)	-14.9 (-21.8, -6.0)
Mauritius	54 (25, 96)	8.5 (3.9, 15.1)	141 (67, 259)	8.4 (4.0, 15.4)	-0.5 (-8.5, 7.4)
Myanmar	2237 (1055, 4021)	11.5 (5.6, 20.6)	3864 (1827, 6960)	9.6 (4.6, 17.5)	-16.7 (-24.5, -7.9)
Philippines	1994 (928, 3511)	8.3 (3.9, 14.9)	5549 (2590, 9820)	8.6 (4.0, 15.6)	4.2 (2.1, 6.6)
Seychelles	5 (2, 8)	8.3 (3.8, 14.7)	8 (4, 14)	7.9 (3.7, 14.1)	-4.8 (-12.3, 3.2)
Sri Lanka	693 (321, 1233)	7.5 (3.5, 13.5)	1746 (815, 3178)	7.2 (3.3, 13.0)	-5.0 (-11.8, 3.3)
Thailand	2337 (1087, 4273)	8.0 (3.7, 14.4)	6322 (2971, 11442)	6.3 (3.0, 11.4)	-20.8 (-27.6, -13.6)
Timor-Leste	17 (8, 30)	8.8 (4.2, 15.8)	61 (28, 109)	8.2 (3.8, 14.7)	-6.3 (-14.1, 2.4)

Viet Nam	2968 (1382, 5325)	8.0 (3.8, 14.3)	6159 (2850, 11023)	7.7 (3.5, 13.6)	-4.4 (-11.5, 4.4)
Oceania	184 (85, 323)	8.2 (3.8, 14.6)	451 (208, 807)	8.5 (4.0, 15.5)	4.7 (-0.4, 10.4)
American Samoa	2 (1, 3)	8.7 (4.0, 15.6)	4 (2, 7)	9.1 (4.2, 16.1)	4.4 (-3.0, 12.6)
Cook Islands	1 (0, 1)	7.4 (3.4, 13.2)	2 (1, 3)	7.7 (3.6, 14.0)	4.8 (-2.4, 12.8)
Fiji	25 (12, 46)	9.4 (4.4, 17.1)	61 (28, 108)	10.0 (4.7, 17.8)	5.7 (-1.8, 13.5)
Guam	4 (2, 7)	6.6 (3.1, 11.9)	14 (6, 25)	7.5 (3.5, 13.6)	13.9 (5.4, 22.9)
Kiribati	3 (1, 5)	10.1 (4.7, 18.1)	6 (3, 11)	12.5 (5.9, 23.0)	23.4 (13.2, 34.7)
Marshall Islands	1 (1, 2)	8.3 (4.0, 14.9)	2 (1, 4)	8.5 (4.1, 15.2)	2.6 (-5.1, 11.4)
Micronesia (Federated States of)	3 (2, 6)	8.5 (4.0, 15.5)	5 (2, 8)	8.6 (4.0, 15.5)	0.9 (-6.8, 9.5)
Nauru	0 (0, 0)	8.0 (3.8, 14.4)	0 (0, 0)	8.5 (3.9, 15.0)	5.4 (-2.8, 14.3)
Niue	0 (0, 0)	7.9 (3.7, 14.0)	0 (0, 0)	8.0 (3.8, 14.4)	1.3 (-6.4, 9.7)
Northern Mariana Islands	1 (0, 1)	6.7 (3.1, 12.0)	3 (1, 5)	7.0 (3.2, 12.6)	3.4 (-4.2, 12.3)
Palau	1 (0, 1)	7.5 (3.5, 13.4)	1 (1, 2)	7.9 (3.7, 14.2)	4.8 (-3.0, 13.8)
Papua New Guinea	109 (50, 190)	7.8 (3.7, 14.0)	279 (130, 502)	8.2 (3.9, 14.9)	4.8 (-2.9, 13.2)
Samoa	7 (3, 12)	9.1 (4.2, 16.2)	12 (6, 22)	9.8 (4.5, 17.4)	7.0 (-0.7, 15.2)
Solomon Islands	8 (4, 14)	8.0 (3.7, 14.3)	18 (8, 32)	8.5 (4.0, 15.1)	6.1 (-1.7, 14.6)
Tokelau	0 (0, 0)	8.3 (3.9, 14.8)	0 (0, 0)	8.3 (3.8, 14.7)	-0.3 (-7.9, 8.4)
Tonga	4 (2, 8)	8.9 (4.1, 15.7)	7 (3, 13)	9.4 (4.4, 17.0)	5.8 (-1.5, 13.9)
Tuvalu	1 (0, 1)	8.7 (4.1, 15.7)	1 (0, 1)	8.8 (4.1, 15.7)	0.8 (-7.1, 9.3)
Vanuatu	4 (2, 8)	8.5 (4.1, 15.2)	14 (6, 24)	9.3 (4.5, 16.5)	9.4 (1.2, 17.6)
Central Asia	2585 (1215, 4611)	6.3 (3.0, 11.1)	3421 (1601, 6199)	5.8 (2.8, 10.5)	-6.9 (-9.9, -3.2)

Armenia	167<br>(78, 302)	7.3<br>(3.4, 13.3)	262<br>(124, 476)	6.3<br>(3.0, 11.4)	-13.4<br>(-20.4, -6.7)
Azerbaijan	297<br>(141, 522)	6.9<br>(3.3, 12.3)	443<br>(209, 801)	6.1<br>(2.9, 11.0)	-11.5<br>(-18.2, -4.3)
Georgia	353<br>(164, 634)	6.2<br>(3.0, 11.1)	415<br>(195, 748)	6.4<br>(3.0, 11.7)	3.8<br>(-3.8, 12.3)
Kazakhstan	733<br>(349, 1319)	6.6<br>(3.1, 11.9)	852<br>(396, 1544)	5.7<br>(2.7, 10.4)	-13.7<br>(-21.1, -5.1)
Kyrgyzstan	165<br>(77, 294)	5.8<br>(2.7, 10.4)	199<br>(93, 361)	5.3<br>(2.5, 9.7)	-9.5<br>(-16.1, -2.2)
Mongolia	61<br>(28, 111)	6.8<br>(3.1, 12.3)	100<br>(47, 184)	6.0<br>(2.8, 10.9)	-12.4<br>(-19.8, -4.7)
Tajikistan	180<br>(83, 316)	6.9<br>(3.2, 12.3)	204<br>(94, 369)	5.8<br>(2.7, 10.6)	-16.3<br>(-23.3, -9.0)
Turkmenistan	103<br>(48, 188)	6.5<br>(3.0, 11.8)	183<br>(86, 331)	5.8<br>(2.8, 10.6)	-9.9<br>(-16.9, -2.0)
Uzbekistan	525<br>(248, 937)	5.2<br>(2.5, 9.4)	763<br>(348, 1371)	5.4<br>(2.5, 9.8)	3.5<br>(-5.8, 12.2)
Central Europe	9984<br>(4662, 17986)	7.0<br>(3.4, 12.7)	12853<br>(6176, 23498)	5.6<br>(2.7, 10.2)	-20.5<br>(-22.1, -18.7)
Albania	116<br>(55, 207)	6.4<br>(3.0, 11.5)	237<br>(110, 428)	5.3<br>(2.5, 9.6)	-16.5<br>(-22.7, -9.8)
Bosnia and Herzegovina	230<br>(107, 412)	6.8<br>(3.2, 12.2)	401<br>(194, 745)	6.6<br>(3.2, 12.2)	-2.8<br>(-10.4, 4.9)
Bulgaria	771<br>(357, 1402)	6.5<br>(3.0, 11.7)	915<br>(431, 1683)	5.8<br>(2.7, 10.5)	-10.3<br>(-16.9, -3.8)
Croatia	417<br>(194, 755)	6.8<br>(3.2, 12.4)	477<br>(227, 891)	5.0<br>(2.4, 9.3)	-26.2<br>(-32.3, -20.0)
Czechia	917<br>(430, 1681)	6.6<br>(3.1, 12.0)	1229<br>(583, 2243)	5.4<br>(2.6, 9.9)	-17.5<br>(-23.6, -10.9)
Hungary	1097<br>(516, 2040)	7.4<br>(3.5, 13.8)	1244<br>(591, 2282)	5.9<br>(2.8, 10.8)	-19.7<br>(-25.3, -13.3)
North Macedonia	115<br>(53, 207)	6.9<br>(3.2, 12.3)	184<br>(87, 340)	5.9<br>(2.8, 11.0)	-14.3<br>(-20.6, -7.6)
Montenegro	41<br>(19, 75)	7.1<br>(3.3, 13.0)	65<br>(32, 120)	6.5<br>(3.2, 12.0)	-8.7<br>(-14.4, -1.6)
Poland	3373<br>(1609, 6043)	7.9<br>(3.8, 14.2)	4285<br>(2045, 7753)	5.7<br>(2.7, 10.3)	-27.7<br>(-29.7, -25.2)
Romania	1685<br>(770, 3044)	6.4<br>(3.0, 11.5)	2128<br>(993, 3846)	5.3<br>(2.5, 9.5)	-16.9<br>(-23.0, -10.5)
Serbia	681<br>(321, 1244)	6.5<br>(3.1, 11.8)	989<br>(466, 1818)	5.9<br>(2.8, 10.8)	-9.6<br>(-16.2, -2.6)

Slovakia	395<br>(187, 709)	6.7<br>(3.2, 11.8)	471<br>(223, 862)	4.9<br>(2.3, 9.0)	-25.8<br>(-31.4, -19.4)
Slovenia	148<br>(69, 269)	6.1<br>(2.9, 11.1)	228<br>(109, 415)	4.8<br>(2.4, 8.8)	-20.6<br>(-26.1, -14.8)
Eastern Europe	22153<br>(10503, 40000)	8.4<br>(4.0, 15.2)	26864<br>(12865, 49225)	7.5<br>(3.6, 13.7)	-10.4<br>(-12.7, -7.7)
Belarus	900<br>(423, 1656)	7.1<br>(3.4, 13.1)	1026<br>(482, 1887)	6.2<br>(2.9, 11.5)	-12.0<br>(-19.7, -4.0)
Estonia	150<br>(69, 275)	7.3<br>(3.4, 13.5)	182<br>(86, 337)	6.2<br>(3.0, 11.4)	-15.4<br>(-22.4, -7.8)
Latvia	231<br>(108, 417)	6.4<br>(3.0, 11.6)	279<br>(130, 508)	6.3<br>(2.9, 11.3)	-1.8<br>(-9.6, 6.6)
Lithuania	315<br>(148, 573)	7.0<br>(3.3, 12.5)	391<br>(184, 709)	6.1<br>(2.9, 11.3)	-11.9<br>(-18.4, -4.4)
Republic of Moldova	260<br>(122, 478)	6.5<br>(3.1, 12.0)	373<br>(179, 681)	6.3<br>(3.0, 11.7)	-3.1<br>(-10.7, 5.4)
Russian Federation	14403<br>(6853, 25938)	8.6<br>(4.1, 15.5)	18469<br>(8775, 33575)	7.6<br>(3.6, 13.9)	-10.9<br>(-12.6, -9.2)
Ukraine	5894<br>(2836, 10812)	8.5<br>(4.0, 15.4)	6144<br>(2965, 11316)	7.7<br>(3.7, 14.1)	-9.4<br>(-17.4, -0.1)
High-income Asia Pacific	17331<br>(8174, 31517)	9.1<br>(4.3, 16.4)	24680<br>(11717, 44731)	4.8<br>(2.3, 8.8)	-46.5<br>(-47.6, -45.4)
Brunei Darussalam	7<br>(3, 13)	10.1<br>(4.7, 18.3)	14<br>(6, 25)	6.2<br>(2.9, 11.5)	-38.3<br>(-42.8, -33.0)
Japan	15100<br>(7150, 27495)	9.1<br>(4.3, 16.5)	20385<br>(9767, 37058)	4.9<br>(2.3, 9.0)	-45.9<br>(-46.9, -44.9)
Republic of Korea	2088<br>(970, 3812)	8.7<br>(4.1, 15.6)	3975<br>(1868, 7269)	4.5<br>(2.1, 8.2)	-48.7<br>(-52.7, -44.6)
Singapore	136<br>(62, 250)	7.3<br>(3.4, 13.4)	307<br>(142, 563)	4.1<br>(1.9, 7.5)	-44.1<br>(-48.2, -39.7)
Australasia	1798<br>(853, 3262)	7.6<br>(3.6, 13.6)	2355<br>(1114, 4265)	4.5<br>(2.1, 8.1)	-41.1<br>(-44.7, -37.3)
Australia	1482<br>(701, 2720)	7.5<br>(3.5, 13.7)	1980<br>(936, 3593)	4.5<br>(2.1, 8.1)	-40.6<br>(-44.9, -35.9)
New Zealand	317<br>(148, 580)	7.9<br>(3.7, 14.4)	375<br>(180, 680)	4.5<br>(2.1, 8.1)	-43.2<br>(-47.3, -38.9)
Western Europe	74358<br>(34861, 134556)	12.2<br>(5.7, 22.2)	71984<br>(33793, 130193)	7.3<br>(3.4, 13.2)	-40.4<br>(-41.8, -39.0)
Andorra	5<br>(3, 10)	10.6<br>(4.9, 19.2)	10<br>(5, 18)	7.1<br>(3.4, 12.8)	-33.0<br>(-37.4, -27.3)
Austria	1374<br>(656, 2492)	11.0<br>(5.2, 20.0)	1415<br>(672, 2580)	7.4<br>(3.5, 13.5)	-32.8<br>(-37.4, -27.2)

Belgium	1961 (922, 3567)	12.2 (5.7, 22.2)	2002 (946, 3638)	8.2 (3.9, 15.0)	-32.9 (-38.0, -27.8)
Cyprus	103 (47, 188)	12.7 (5.9, 23.1)	167 (79, 306)	8.4 (4.0, 15.4)	-33.5 (-38.1, -28.6)
Denmark	1513 (714, 2751)	17.5 (8.2, 31.9)	1286 (612, 2366)	10.4 (4.9, 19.0)	-40.7 (-44.5, -36.4)
Finland	876 (411, 1607)	11.9 (5.6, 21.6)	954 (453, 1713)	7.1 (3.3, 12.8)	-40.3 (-44.7, -35.8)
France	10062 (4714, 18228)	11.5 (5.3, 20.6)	10528 (4988, 18918)	7.1 (3.3, 12.7)	-38.3 (-42.8, -33.8)
Germany	16692 (7792, 30750)	12.6 (5.9, 23.5)	15083 (7048, 27038)	7.3 (3.4, 13.2)	-42.3 (-46.4, -37.9)
Greece	1892 (863, 3458)	12.1 (5.5, 22.2)	2094 (992, 3804)	8.1 (3.8, 14.7)	-33.3 (-38.3, -28.2)
Iceland	34 (16, 63)	11.6 (5.4, 21.3)	42 (19, 75)	7.1 (3.3, 12.9)	-38.4 (-42.8, -33.4)
Ireland	579 (269, 1060)	13.7 (6.5, 25.4)	617 (287, 1119)	8.0 (3.7, 14.4)	-41.9 (-46.0, -37.7)
Israel	610 (290, 1116)	12.3 (5.8, 22.4)	984 (463, 1795)	8.1 (3.8, 14.8)	-34.2 (-38.5, -29.6)
Italy	12039 (5725, 21929)	13.0 (6.2, 23.5)	11743 (5606, 21474)	7.4 (3.5, 13.6)	-42.8 (-44.2, -41.4)
Luxembourg	71 (33, 129)	12.7 (5.9, 23.0)	84 (40, 152)	8.1 (3.8, 14.6)	-36.1 (-40.5, -30.9)
Malta	56 (26, 101)	13.2 (6.2, 23.8)	88 (42, 160)	8.7 (4.1, 16.0)	-33.9 (-38.4, -28.6)
Monaco	9 (4, 17)	11.8 (5.5, 21.3)	8 (4, 15)	7.7 (3.6, 14.1)	-35.0 (-39.6, -30.3)
Netherlands	2417 (1130, 4461)	11.7 (5.4, 21.4)	2633 (1226, 4879)	7.2 (3.4, 13.3)	-38.1 (-43.7, -32.2)
Norway	924 (441, 1676)	12.4 (5.8, 22.3)	802 (381, 1459)	7.8 (3.7, 14.1)	-36.9 (-38.2, -35.7)
Portugal	1713 (797, 3121)	12.0 (5.7, 21.9)	1982 (923, 3634)	7.6 (3.5, 13.8)	-36.8 (-41.6, -31.8)
Spain	6258 (2839, 11375)	11.0 (5.0, 19.9)	7041 (3336, 12628)	6.8 (3.2, 12.2)	-38.5 (-43.5, -34.0)
Sweden	1944 (931, 3526)	11.7 (5.6, 21.4)	1872 (867, 3368)	8.1 (3.7, 14.8)	-30.8 (-35.5, -26.1)
Switzerland	1237 (579, 2260)	11.3 (5.3, 20.2)	1380 (655, 2506)	7.4 (3.5, 13.5)	-34.1 (-39.1, -29.0)
United Kingdom	11923 (5655, 21659)	12.3 (5.8, 22.4)	9102 (4307, 16616)	6.7 (3.2, 12.2)	-45.3 (-46.1, -44.5)

Southern Latin America	4378<br>(2022, 8042)	9.8<br>(4.5, 18.0)	5636<br>(2646, 10152)	6.6<br>(3.1, 11.9)	-33.0<br>(-36.5, -29.0)
Argentina	3076<br>(1419, 5700)	9.8<br>(4.6, 18.1)	3715<br>(1742, 6751)	6.7<br>(3.1, 12.1)	-31.8<br>(-36.5, -26.1)
Chile	869<br>(409, 1585)	9.4<br>(4.5, 17.1)	1500<br>(700, 2712)	6.2<br>(2.9, 11.2)	-34.5<br>(-39.0, -29.7)
Uruguay	433<br>(202, 794)	10.8<br>(5.0, 19.7)	421<br>(197, 771)	7.2<br>(3.4, 13.2)	-33.0<br>(-37.9, -28.0)
High-income North America	40501<br>(19113, 72877)	11.0<br>(5.2, 19.8)	50935<br>(23761, 90890)	7.7<br>(3.6, 13.7)	-30.4<br>(-35.2, -24.8)
Canada	4427<br>(2069, 8192)	13.4<br>(6.3, 24.8)	5506<br>(2565, 9985)	7.6<br>(3.5, 13.9)	-43.7<br>(-47.5, -39.5)
United States of America	36070<br>(17069, 64990)	10.8<br>(5.1, 19.3)	45422<br>(21165, 81286)	7.7<br>(3.6, 13.7)	-28.7<br>(-34.2, -22.1)
Greenland	4<br>(2, 8)	15.9<br>(7.4, 28.5)	6<br>(3, 11)	9.5<br>(4.5, 17.2)	-40.6<br>(-44.8, -36.5)
Caribbean	1357<br>(640, 2438)	5.5<br>(2.6, 9.9)	2446<br>(1166, 4399)	4.7<br>(2.3, 8.5)	-13.5<br>(-16.9, -10.3)
Antigua and Barbuda	3<br>(1, 6)	5.3<br>(2.5, 9.7)	4<br>(2, 8)	4.6<br>(2.2, 8.5)	-13.2<br>(-20.0, -5.8)
Bahamas	7<br>(3, 13)	5.3<br>(2.5, 9.6)	16<br>(7, 29)	4.7<br>(2.2, 8.5)	-10.8<br>(-17.9, -3.1)
Barbados	17<br>(8, 31)	5.3<br>(2.5, 9.4)	24<br>(11, 42)	4.6<br>(2.2, 8.3)	-12.2<br>(-18.5, -4.3)
Belize	5<br>(2, 9)	5.4<br>(2.6, 9.8)	12<br>(6, 21)	4.9<br>(2.3, 8.8)	-9.4<br>(-16.8, -1.6)
Bermuda	3<br>(1, 5)	4.9<br>(2.3, 9.0)	6<br>(3, 10)	4.1<br>(1.9, 7.5)	-16.9<br>(-22.8, -10.3)
Cuba	555<br>(266, 1006)	5.4<br>(2.6, 9.8)	878<br>(414, 1597)	4.5<br>(2.1, 8.1)	-18.3<br>(-24.9, -11.5)
Dominica	4<br>(2, 7)	5.2<br>(2.4, 9.4)	4<br>(2, 8)	4.8<br>(2.3, 8.5)	-8.7<br>(-15.9, -1.7)
Dominican Republic	192<br>(90, 344)	5.9<br>(2.8, 10.6)	447<br>(212, 802)	5.2<br>(2.5, 9.3)	-12.6<br>(-19.9, -5.3)
Grenada	4<br>(2, 8)	5.5<br>(2.6, 10.1)	5<br>(2, 9)	5.0<br>(2.4, 9.1)	-8.9<br>(-15.6, -1.4)
Guyana	18<br>(8, 32)	5.7<br>(2.7, 10.0)	27<br>(12, 49)	5.2<br>(2.4, 9.2)	-8.9<br>(-16.3, -1.3)
Haiti	154<br>(70, 277)	6.0<br>(2.8, 10.6)	286<br>(133, 507)	5.2<br>(2.4, 9.2)	-13.8<br>(-20.6, -7.2)
Jamaica	96<br>(45, 175)	5.2<br>(2.4, 9.4)	160<br>(76, 290)	5.3<br>(2.5, 9.6)	1.7<br>(-5.9, 10.5)

Puerto Rico	176<br>(82, 319)	4.8<br>(2.3, 8.7)	339<br>(164, 606)	4.2<br>(2.0, 7.6)	-12.1<br>(-18.9, -5.1)
Saint Kitts and Nevis	2<br>(1, 4)	5.2<br>(2.4, 9.3)	3<br>(1, 5)	4.6<br>(2.1, 8.1)	-11.8<br>(-18.8, -3.9)
Saint Lucia	5<br>(2, 9)	5.8<br>(2.7, 10.4)	10<br>(5, 18)	4.9<br>(2.3, 8.7)	-15.1<br>(-21.4, -8.0)
Saint Vincent and the Grenadines	4<br>(2, 7)	5.5<br>(2.6, 9.8)	7<br>(3, 12)	5.1<br>(2.4, 9.2)	-5.7<br>(-12.4, 1.7)
Suriname	14<br>(6, 24)	5.8<br>(2.8, 10.3)	31<br>(14, 55)	5.5<br>(2.6, 9.9)	-4.7<br>(-11.9, 3.4)
Trinidad and Tobago	49<br>(23, 89)	6.3<br>(2.9, 11.4)	98<br>(46, 177)	5.4<br>(2.6, 9.7)	-14.3<br>(-22.0, -6.3)
United States Virgin Islands	4<br>(2, 6)	4.9<br>(2.3, 8.9)	9<br>(4, 16)	4.5<br>(2.1, 8.2)	-8.0<br>(-15.3, -0.4)
Andean Latin America	895<br>(423, 1597)	5.0<br>(2.4, 8.8)	2185<br>(1020, 3973)	4.1<br>(1.9, 7.4)	-18.0<br>(-22.3, -13.3)
Bolivia (Plurinational State of)	156<br>(73, 274)	5.8<br>(2.7, 10.3)	361<br>(169, 652)	4.6<br>(2.1, 8.2)	-21.2<br>(-27.7, -14.4)
Ecuador	249<br>(116, 451)	5.3<br>(2.5, 9.5)	606<br>(279, 1086)	4.3<br>(2.0, 7.6)	-19.2<br>(-25.2, -12.4)
Peru	490<br>(232, 871)	4.6<br>(2.2, 8.2)	1218<br>(569, 2244)	3.9<br>(1.8, 7.2)	-16.4<br>(-22.5, -9.2)
Central Latin America	4758<br>(2217, 8509)	6.6<br>(3.1, 11.8)	11222<br>(5257, 20137)	5.0<br>(2.4, 9.1)	-24.4<br>(-26.6, -21.4)
Colombia	946<br>(444, 1707)	6.4<br>(3.0, 11.4)	2424<br>(1130, 4321)	4.6<br>(2.1, 8.1)	-28.4<br>(-33.9, -22.3)
Costa Rica	91<br>(43, 162)	5.6<br>(2.7, 10.0)	229<br>(108, 407)	4.6<br>(2.1, 8.1)	-19.0<br>(-25.6, -12.1)
El Salvador	154<br>(73, 274)	5.6<br>(2.7, 10.0)	288<br>(134, 522)	4.7<br>(2.2, 8.5)	-16.3<br>(-23.4, -8.4)
Guatemala	169<br>(79, 301)	5.8<br>(2.8, 10.4)	523<br>(244, 962)	5.1<br>(2.4, 9.4)	-12.1<br>(-18.9, -4.7)
Honduras	114<br>(53, 204)	6.4<br>(3.0, 11.4)	287<br>(133, 509)	5.4<br>(2.5, 9.6)	-16.0<br>(-22.9, -8.6)
Mexico	2544<br>(1203, 4575)	6.9<br>(3.3, 12.4)	5739<br>(2710, 10323)	5.3<br>(2.5, 9.5)	-23.3<br>(-25.7, -19.9)
Nicaragua	81<br>(38, 146)	6.2<br>(2.9, 11.3)	189<br>(87, 346)	5.0<br>(2.3, 9.1)	-20.2<br>(-26.4, -13.2)
Panama	76<br>(36, 134)	5.5<br>(2.6, 9.7)	188<br>(89, 343)	4.6<br>(2.2, 8.3)	-16.8<br>(-23.0, -10.4)
Venezuela (Bolivarian Republic of)	583<br>(272, 1035)	7.0<br>(3.3, 12.4)	1354<br>(639, 2469)	5.0<br>(2.4, 8.9)	-29.1<br>(-35.1, -22.5)

Tropical Latin America	4941<br>(2323, 8892)	6.5<br>(3.1, 11.5)	10643<br>(5013, 19411)	4.6<br>(2.2, 8.4)	-28.5<br>(-31.0, -25.3)
Brazil	4816<br>(2265, 8671)	6.5<br>(3.1, 11.5)	10371<br>(4896, 18931)	4.6<br>(2.2, 8.3)	-28.9<br>(-31.3, -25.6)
Paraguay	124<br>(58, 224)	6.2<br>(2.9, 11.0)	272<br>(127, 496)	5.3<br>(2.5, 9.6)	-14.6<br>(-20.9, -7.3)
North Africa and Middle East	8636<br>(3969, 15577)	6.2<br>(2.9, 11.0)	18907<br>(8866, 34351)	5.2<br>(2.5, 9.4)	-15.3<br>(-17.7, -11.7)
Afghanistan	362<br>(167, 653)	5.9<br>(2.8, 10.7)	525<br>(244, 929)	6.0<br>(2.8, 10.7)	1.9<br>(-5.9, 10.3)
Algeria	629<br>(292, 1149)	6.0<br>(2.8, 11.0)	1518<br>(720, 2739)	5.2<br>(2.4, 9.4)	-13.4<br>(-20.5, -5.8)
Bahrain	8<br>(4, 14)	6.3<br>(2.9, 11.4)	34<br>(16, 63)	5.2<br>(2.4, 9.4)	-17.0<br>(-23.5, -10.2)
Egypt	1352<br>(618, 2451)	5.8<br>(2.7, 10.3)	2752<br>(1278, 4975)	5.4<br>(2.5, 9.6)	-5.9<br>(-13.0, 1.8)
Iran (Islamic Republic of)	1093<br>(501, 1983)	5.5<br>(2.6, 9.9)	3267<br>(1548, 5937)	5.1<br>(2.5, 9.3)	-6.9<br>(-9.5, -3.2)
Iraq	495<br>(230, 894)	7.4<br>(3.4, 13.3)	1065<br>(494, 1946)	5.8<br>(2.7, 10.6)	-20.7<br>(-26.7, -14.0)
Jordan	64<br>(30, 114)	6.4<br>(3.0, 11.5)	277<br>(129, 505)	5.4<br>(2.5, 9.8)	-15.2<br>(-21.1, -7.4)
Kuwait	22<br>(10, 39)	5.2<br>(2.4, 9.4)	89<br>(41, 160)	4.8<br>(2.2, 8.6)	-8.5<br>(-15.2, -1.9)
Lebanon	131<br>(61, 242)	6.7<br>(3.1, 12.3)	296<br>(140, 547)	5.7<br>(2.7, 10.5)	-14.5<br>(-20.8, -7.6)
Libya	89<br>(41, 161)	5.6<br>(2.6, 10.1)	220<br>(103, 396)	5.2<br>(2.4, 9.5)	-6.8<br>(-13.8, 0.2)
Morocco	710<br>(325, 1301)	6.2<br>(2.8, 11.2)	1434<br>(675, 2619)	5.4<br>(2.5, 9.8)	-13.0<br>(-19.1, -5.0)
Palestine	42<br>(20, 76)	5.5<br>(2.6, 9.9)	94<br>(44, 168)	5.1<br>(2.4, 8.9)	-8.2<br>(-14.9, -0.8)
Oman	25<br>(12, 46)	5.5<br>(2.6, 10.0)	58<br>(27, 106)	5.1<br>(2.4, 9.3)	-7.0<br>(-13.4, -0.1)
Qatar	4<br>(2, 7)	6.5<br>(3.1, 11.7)	24<br>(11, 45)	4.6<br>(2.2, 8.3)	-29.3<br>(-34.9, -23.5)
Saudi Arabia	224<br>(105, 403)	5.0<br>(2.4, 9.0)	478<br>(217, 874)	4.3<br>(2.0, 7.8)	-14.7<br>(-21.4, -7.7)
Sudan	398<br>(189, 721)	5.0<br>(2.4, 8.9)	723<br>(335, 1303)	4.8<br>(2.2, 8.6)	-5.2<br>(-12.9, 3.0)
Syrian Arab Republic	258<br>(118, 466)	5.9<br>(2.7, 10.6)	502<br>(231, 920)	4.8<br>(2.3, 8.6)	-18.3<br>(-24.0, -11.4)

Tunisia	253<br>(116, 463)	5.8<br>(2.7, 10.4)	606<br>(285, 1117)	5.2<br>(2.5, 9.6)	-10.8<br>(-18.0, -3.1)
Türkiye	2240<br>(1012, 4010)	7.3<br>(3.4, 13.1)	4266<br>(2017, 7829)	5.1<br>(2.4, 9.4)	-30.0<br>(-35.5, -23.6)
United Arab Emirates	12<br>(6, 23)	5.9<br>(2.7, 10.4)	87<br>(39, 161)	5.1<br>(2.4, 9.3)	-12.6<br>(-19.1, -5.9)
Yemen	219<br>(101, 394)	5.8<br>(2.8, 10.4)	570<br>(266, 1019)	5.4<br>(2.5, 9.7)	-6.8<br>(-13.7, 0.6)
South Asia	22574<br>(10457, 39928)	5.3<br>(2.5, 9.5)	58013<br>(27257, 103644)	4.8<br>(2.3, 8.4)	-10.8<br>(-13.2, -7.4)
Bangladesh	1983<br>(949, 3550)	5.1<br>(2.4, 9.1)	5039<br>(2388, 9005)	4.2<br>(2.0, 7.5)	-16.8<br>(-23.5, -8.9)
Bhutan	10<br>(5, 18)	5.4<br>(2.5, 9.5)	22<br>(10, 39)	4.3<br>(2.0, 7.7)	-19.6<br>(-26.1, -11.8)
India	17162<br>(7941, 30529)	5.2<br>(2.5, 9.3)	47210<br>(22187, 84447)	4.8<br>(2.3, 8.4)	-9.2<br>(-11.8, -5.7)
Nepal	408<br>(190, 716)	5.6<br>(2.7, 10.0)	932<br>(439, 1676)	4.8<br>(2.3, 8.4)	-15.4<br>(-22.2, -7.2)
Pakistan	3011<br>(1399, 5428)	6.0<br>(2.9, 10.9)	4811<br>(2251, 8567)	5.7<br>(2.7, 10.2)	-5.8<br>(-10.5, -0.7)
Central Sub-Saharan Africa	1132<br>(527, 2019)	6.7<br>(3.1, 12.0)	2456<br>(1129, 4402)	6.3<br>(3.0, 11.4)	-6.0<br>(-11.3, -0.3)
Angola	199<br>(95, 350)	7.3<br>(3.5, 13.1)	553<br>(257, 1005)	7.0<br>(3.3, 12.6)	-4.1<br>(-11.7, 3.8)
Central African Republic	58<br>(27, 101)	7.0<br>(3.3, 12.5)	102<br>(47, 183)	7.1<br>(3.4, 12.8)	1.5<br>(-7.3, 9.9)
Congo	61<br>(28, 110)	7.4<br>(3.4, 13.3)	124<br>(57, 222)	6.5<br>(3.0, 11.7)	-12.5<br>(-19.7, -5.2)
Democratic Republic of the Congo	770<br>(356, 1396)	6.4<br>(3.0, 11.6)	1599<br>(735, 2852)	6.0<br>(2.9, 10.7)	-6.3<br>(-13.4, 1.4)
Equatorial Guinea	11<br>(5, 19)	7.2<br>(3.4, 12.7)	23<br>(11, 42)	6.4<br>(3.0, 11.3)	-11.2<br>(-19.0, -2.6)
Gabon	33<br>(15, 58)	6.9<br>(3.3, 12.2)	54<br>(25, 97)	6.4<br>(3.0, 11.6)	-7.2<br>(-14.0, 1.0)
Eastern Sub-Saharan Africa	3299<br>(1522, 5883)	5.7<br>(2.7, 10.2)	6805<br>(3186, 12174)	5.4<br>(2.5, 9.6)	-4.7<br>(-7.3, -1.5)
Burundi	117<br>(54, 211)	6.0<br>(2.8, 10.7)	185<br>(86, 332)	5.5<br>(2.5, 9.7)	-8.3<br>(-16.1, -0.2)
Comoros	11<br>(5, 21)	5.9<br>(2.8, 10.7)	23<br>(11, 41)	5.4<br>(2.6, 9.6)	-8.5<br>(-15.2, -0.8)
Djibouti	5<br>(2, 10)	5.9<br>(2.7, 10.6)	23<br>(11, 42)	5.5<br>(2.6, 10.0)	-5.8<br>(-13.0, 2.5)

Eritrea	38<br>(17, 67)	6.0<br>(2.9, 10.8)	98<br>(45, 175)	5.4<br>(2.5, 9.8)	-9.8<br>(-17.5, -0.8)
Ethiopia	742<br>(339, 1324)	5.2<br>(2.5, 9.4)	1537<br>(721, 2775)	4.6<br>(2.2, 8.3)	-10.9<br>(-15.2, -5.9)
Kenya	384<br>(177, 687)	5.8<br>(2.7, 10.3)	929<br>(430, 1655)	5.6<br>(2.6, 10.1)	-2.2<br>(-4.0, 0.3)
Madagascar	233<br>(110, 415)	5.5<br>(2.6, 9.9)	417<br>(192, 736)	5.4<br>(2.6, 9.5)	-2.4<br>(-9.5, 5.3)
Malawi	198<br>(92, 349)	6.5<br>(3.0, 11.5)	348<br>(162, 632)	6.0<br>(2.8, 10.7)	-8.1<br>(-15.8, -1.0)
Mozambique	297<br>(138, 536)	6.3<br>(3.0, 11.4)	522<br>(244, 928)	6.2<br>(2.9, 11.1)	-2.0<br>(-9.0, 7.0)
Rwanda	149<br>(69, 268)	6.4<br>(3.1, 11.5)	268<br>(123, 492)	5.7<br>(2.7, 10.5)	-10.9<br>(-18.3, -2.6)
Somalia	101<br>(47, 181)	5.9<br>(2.8, 10.6)	284<br>(131, 501)	6.1<br>(2.8, 10.8)	2.6<br>(-5.7, 11.2)
South Sudan	108<br>(49, 193)	5.5<br>(2.5, 9.8)	168<br>(77, 299)	5.8<br>(2.7, 10.3)	5.2<br>(-2.5, 13.8)
Uganda	288<br>(133, 519)	5.5<br>(2.6, 9.8)	577<br>(271, 1041)	5.3<br>(2.5, 9.5)	-3.0<br>(-10.5, 5.9)
United Republic of Tanzania	493<br>(227, 898)	5.6<br>(2.6, 10.1)	1145<br>(538, 2027)	5.8<br>(2.7, 10.2)	3.6<br>(-4.5, 12.9)
Zambia	132<br>(60, 235)	6.0<br>(2.8, 10.6)	274<br>(127, 496)	5.5<br>(2.6, 9.9)	-8.8<br>(-16.4, 0.0)
Southern Sub-Saharan Africa	2012<br>(936, 3588)	8.5<br>(4.1, 15.3)	3334<br>(1568, 6000)	7.0<br>(3.3, 12.5)	-18.4<br>(-20.8, -15.5)
Botswana	34<br>(15, 61)	7.3<br>(3.4, 13.2)	69<br>(32, 123)	6.6<br>(3.1, 11.7)	-9.5<br>(-17.0, -1.5)
Lesotho	61<br>(28, 109)	7.2<br>(3.4, 12.9)	78<br>(35, 142)	7.6<br>(3.5, 13.9)	5.5<br>(-2.8, 14.5)
Namibia	49<br>(22, 86)	7.7<br>(3.6, 13.8)	78<br>(36, 141)	6.5<br>(3.0, 11.6)	-16.2<br>(-22.9, -8.8)
South Africa	1632<br>(764, 2922)	9.0<br>(4.3, 16.1)	2672<br>(1249, 4836)	6.9<br>(3.3, 12.5)	-23.0<br>(-25.7, -19.9)
Eswatini	18<br>(8, 32)	7.9<br>(3.7, 14.2)	35<br>(16, 64)	7.7<br>(3.6, 13.7)	-3.2<br>(-10.6, 4.9)
Zimbabwe	219<br>(101, 393)	6.7<br>(3.2, 11.8)	403<br>(189, 720)	7.3<br>(3.5, 13.0)	9.5<br>(1.1, 17.9)
Western Sub-Saharan Africa	3748<br>(1731, 6741)	5.2<br>(2.5, 9.3)	7484<br>(3475, 13479)	5.1<br>(2.4, 9.2)	-2.5<br>(-4.6, 0.0)
Benin	101<br>(47, 181)	5.7<br>(2.7, 10.3)	216<br>(101, 389)	5.6<br>(2.6, 10.0)	-3.0<br>(-10.6, 4.9)

Burkina Faso	187<br>(85, 333)	5.2<br>(2.5, 9.2)	365<br>(171, 654)	5.1<br>(2.4, 9.1)	-2.2<br>(-9.3, 6.2)
Cameroon	182<br>(83, 325)	5.2<br>(2.5, 9.2)	468<br>(219, 848)	5.1<br>(2.4, 9.1)	-1.6<br>(-9.9, 7.0)
Cabo Verde	13<br>(6, 24)	5.4<br>(2.5, 9.7)	20<br>(9, 35)	5.0<br>(2.3, 9.2)	-7.8<br>(-15.4, 0.2)
Chad	129<br>(61, 232)	5.1<br>(2.4, 9.2)	229<br>(107, 414)	5.2<br>(2.4, 9.1)	0.3<br>(-7.3, 7.6)
Cote d'Ivoire	158<br>(72, 280)	5.7<br>(2.6, 10.1)	437<br>(205, 775)	5.6<br>(2.7, 9.9)	-0.7<br>(-7.8, 8.0)
Gambia	16<br>(8, 30)	5.9<br>(2.8, 10.7)	46<br>(22, 84)	5.7<br>(2.7, 10.3)	-3.6<br>(-10.8, 4.0)
Ghana	262<br>(122, 469)	5.4<br>(2.6, 9.6)	664<br>(308, 1206)	5.2<br>(2.4, 9.3)	-3.9<br>(-11.8, 4.8)
Guinea	150<br>(71, 272)	5.1<br>(2.4, 9.3)	237<br>(112, 427)	5.1<br>(2.4, 9.0)	-1.2<br>(-8.4, 6.5)
Guinea-Bissau	19<br>(9, 33)	5.8<br>(2.8, 10.6)	30<br>(14, 54)	5.6<br>(2.7, 10.0)	-4.6<br>(-11.9, 3.3)
Liberia	58<br>(27, 104)	5.9<br>(2.8, 10.8)	86<br>(40, 154)	5.5<br>(2.6, 9.9)	-6.2<br>(-13.2, 2.2)
Mali	175<br>(80, 312)	5.2<br>(2.5, 9.4)	355<br>(165, 639)	5.1<br>(2.4, 9.2)	-2.4<br>(-9.4, 5.4)
Mauritania	47<br>(22, 84)	5.4<br>(2.5, 9.6)	91<br>(43, 165)	5.1<br>(2.4, 9.1)	-6.8<br>(-13.9, 1.1)
Niger	108<br>(49, 193)	5.2<br>(2.5, 9.5)	305<br>(143, 554)	5.1<br>(2.4, 9.2)	-2.7<br>(-10.1, 5.0)
Nigeria	1818<br>(847, 3272)	5.0<br>(2.3, 9.0)	3271<br>(1527, 5875)	4.9<br>(2.3, 8.8)	-2.4<br>(-4.5, -0.1)
Sao Tome and Principe	3<br>(1, 6)	5.5<br>(2.6, 9.7)	4<br>(2, 8)	5.2<br>(2.4, 9.1)	-6.8<br>(-14.1, 0.6)
Senegal	158<br>(73, 284)	5.8<br>(2.8, 10.5)	342<br>(157, 622)	5.4<br>(2.5, 9.8)	-6.9<br>(-13.8, 0.5)
Sierra Leone	110<br>(51, 198)	6.4<br>(3.1, 11.5)	169<br>(79, 306)	5.8<br>(2.7, 10.5)	-9.9<br>(-16.5, -3.0)
Togo	53<br>(24, 95)	5.5<br>(2.6, 9.9)	148<br>(69, 269)	5.4<br>(2.6, 9.8)	-2.0<br>(-9.7, 6.1)
High SDI	115580<br>(54486, 208784)	10.8<br>(5.0, 19.4)	135353<br>(63670, 244469)	6.7<br>(3.1, 12.1)	-38.1<br>(-40.0, -36.1)
High-middle SDI	85617<br>(39959, 153237)	8.7<br>(4.1, 15.6)	135870<br>(63778, 244664)	6.7<br>(3.1, 12.0)	-23.9<br>(-25.4, -22.3)
Middle SDI	61442<br>(28541, 108071)	7.3<br>(3.4, 13.2)	141511<br>(65058, 254571)	6.3<br>(2.9, 11.3)	-13.9<br>(-16.9, -10.0)

Low-middle SDI	29607 (13719, 52398)	6.3 (3.0, 11.2)	66031 (30925, 118081)	5.5 (2.6, 9.9)	-12.2 (-14.5, -9.1)
Low SDI	10423 (4834, 18432)	5.7 (2.7, 10.3)	21879 (10260, 39153)	5.3 (2.5, 9.5)	-7.3 (-9.5, -4.4)

Table S6: YLLs of PAD in 1990 and 2019 for both sexes and percentage change in age-standardised rates by location. PAD= Peripheral artery disease. YLLs=years of life lost.

YLDs of PAD	1990		2019		Percentage change in age-standardised rates between 1990 and 2019
	Counts (95% UI)	Age-standardised rate (95% UI)	Counts (95% UI)	Age-standardised rate (95% UI)	
Global	472694 (257706, 813351)	13.8 (7.5, 23.8)	1035487 (604347, 1778564)	13.3 (7.7, 22.6)	-4.2 (-22.3, 8.8)
East Asia	12139 (9630, 16539)	1.7 (1.3, 2.3)	36737 (30206, 44656)	1.9 (1.6, 2.4)	15.3 (-18.4, 46.5)
China	11769 (9287, 16186)	1.7 (1.3, 2.3)	35669 (29275, 43399)	2.0 (1.6, 2.4)	15.4 (-19.3, 46.8)
Democratic People's Republic of Korea	220 (152, 310)	1.6 (1.1, 2.2)	526 (395, 681)	1.7 (1.3, 2.2)	8.5 (-21.7, 57.1)
Taiwan	150 (72, 282)	1.1 (0.5, 2.1)	542 (248, 1113)	1.3 (0.6, 2.8)	19.2 (-22.8, 78.3)
Southeast Asia	4313 (3478, 5320)	2.0 (1.6, 2.4)	16561 (12891, 20407)	3.0 (2.3, 3.7)	52.7 (22.4, 82.0)
Cambodia	56 (39, 83)	1.5 (1.0, 2.2)	249 (167, 366)	2.4 (1.6, 3.5)	64.6 (7.1, 143.7)
Indonesia	1741 (1296, 2333)	2.1 (1.5, 2.8)	6963 (4502, 10287)	3.8 (2.5, 5.5)	81.2 (36.9, 131.3)
Lao People's Democratic Republic	34 (21, 51)	1.9 (1.3, 2.8)	116 (79, 162)	3.1 (2.1, 4.1)	59.7 (8.2, 143.6)
Malaysia	122 (100, 153)	1.5 (1.2, 1.9)	595 (445, 774)	2.6 (1.9, 3.3)	69.9 (22.2, 132.2)
Maldives	3 (2, 6)	3.7 (2.3, 8.0)	14 (10, 21)	5.4 (3.9, 8.7)	47.2 (-37.2, 145.1)
Mauritius	17 (8, 33)	2.7 (1.2, 5.2)	71 (33, 148)	4.3 (2.0, 8.9)	61.0 (0.7, 144.0)
Myanmar	493 (306, 730)	2.4 (1.5, 3.5)	1387 (1008, 1870)	3.3 (2.5, 4.5)	38.1 (-6.7, 99.3)
Philippines	457 (377, 555)	1.8 (1.5, 2.2)	2183 (1718, 2713)	3.0 (2.4, 3.7)	63.6 (25.5, 104.4)
Seychelles	2 (2, 3)	4.0 (2.8, 5.3)	5 (3, 8)	5.2 (3.1, 7.4)	28.2 (-4.9, 68.8)
Sri Lanka	144 (100, 223)	1.5 (1.0, 2.3)	555 (373, 782)	2.3 (1.5, 3.1)	51.1 (-19.7, 144.1)
Thailand	273 (217, 341)	1.0 (0.8, 1.2)	1032 (749, 1366)	1.0 (0.8, 1.4)	7.8 (-24.4, 52.9)
Timor-Leste	3 (2, 5)	1.4 (0.9, 2.1)	19 (11, 29)	2.7 (1.6, 4.0)	93.7 (28.5, 198.4)

Viet Nam	962 (685, 1280)	2.6 (1.9, 3.4)	3350 (2375, 4476)	3.9 (2.8, 5.1)	51.1 (-2.6, 124.6)
Oceania	51 (37, 88)	2.1 (1.6, 3.5)	188 (139, 275)	3.1 (2.4, 4.6)	49.0 (13.7, 95.2)
American Samoa	1 (1, 1)	3.6 (2.8, 4.6)	3 (2, 3)	5.6 (4.5, 7.0)	58.0 (15.9, 113.0)
Cook Islands	1 (0, 1)	4.4 (3.1, 6.3)	2 (1, 2)	6.8 (4.8, 9.0)	52.7 (-1.3, 129.4)
Fiji	6 (5, 9)	2.1 (1.6, 3.1)	19 (13, 25)	3.3 (2.2, 4.3)	55.5 (-5.0, 134.0)
Guam	1 (1, 2)	2.6 (2.0, 3.9)	7 (6, 10)	3.9 (3.0, 4.9)	47.8 (-4.6, 109.2)
Kiribati	1 (1, 2)	3.1 (2.1, 6.1)	2 (1, 3)	3.3 (2.3, 6.6)	8.4 (-23.2, 50.5)
Marshall Islands	1 (0, 1)	3.9 (2.8, 5.6)	2 (1, 3)	5.6 (3.9, 7.9)	44.0 (3.2, 107.6)
Micronesia (Federated States of)	1 (1, 2)	3.7 (2.5, 5.9)	4 (2, 6)	6.2 (4.3, 9.0)	67.4 (7.9, 146.1)
Nauru	0 (0, 0)	5.2 (3.8, 7.2)	0 (0, 0)	6.2 (4.4, 8.7)	18.3 (-14.5, 59.0)
Niue	0 (0, 0)	3.9 (2.8, 5.9)	0 (0, 0)	6.1 (4.6, 8.0)	54.8 (9.9, 118.7)
Northern Mariana Islands	1 (0, 1)	4.8 (3.3, 6.7)	3 (2, 4)	7.7 (5.4, 9.6)	60.8 (11.2, 128.2)
Palau	0 (0, 0)	1.2 (0.7, 1.8)	0 (0, 0)	1.4 (1.0, 2.0)	16.8 (-29.7, 94.3)
Papua New Guinea	27 (17, 52)	1.8 (1.1, 3.3)	111 (71, 182)	2.7 (1.8, 4.7)	55.4 (11.1, 125.2)
Samoa	3 (2, 5)	3.8 (2.7, 5.7)	7 (5, 9)	4.8 (3.5, 6.5)	26.9 (-10.1, 81.9)
Solomon Islands	3 (2, 6)	2.6 (1.7, 4.6)	10 (7, 17)	3.7 (2.5, 5.7)	44.3 (2.9, 103.6)
Tokelau	0 (0, 0)	2.7 (1.9, 4.2)	0 (0, 0)	4.5 (3.1, 6.0)	67.1 (17.7, 140.5)
Tonga	1 (1, 2)	2.9 (2.1, 4.1)	3 (2, 5)	4.5 (3.2, 6.1)	57.8 (13.7, 123.4)
Tuvalu	0 (0, 0)	3.0 (2.1, 5.1)	0 (0, 1)	4.4 (3.2, 6.4)	46.6 (-2.3, 113.8)
Vanuatu	1 (1, 3)	2.0 (1.2, 4.4)	6 (4, 9)	3.8 (2.6, 5.9)	91.9 (21.4, 202.3)
Central Asia	797 (390, 1450)	1.8 (0.9, 3.3)	1897 (971, 3534)	3.0 (1.5, 5.4)	61.8 (19.3, 94.9)

Armenia	93<br>(30, 287)	3.9<br>(1.3, 12.4)	184<br>(86, 344)	4.5<br>(2.1, 8.3)	14.1<br>(-62.6, 105.3)
Azerbaijan	32<br>(22, 42)	0.7<br>(0.5, 1.0)	78<br>(60, 99)	1.2<br>(0.9, 1.5)	65.9<br>(13.5, 161.0)
Georgia	101<br>(48, 202)	1.7<br>(0.8, 3.4)	182<br>(75, 386)	2.9<br>(1.2, 6.2)	26.8<br>(-4.4, 63.5)
Kazakhstan	197<br>(90, 375)	1.7<br>(0.8, 3.2)	425<br>(200, 822)	2.7<br>(1.3, 5.0)	58.0<br>(13.6, 105.0)
Kyrgyzstan	174<br>(77, 334)	5.9<br>(2.7, 11.4)	346<br>(155, 694)	8.2<br>(3.8, 16.5)	38.6<br>(-14.6, 77.3)
Mongolia	9<br>(7, 14)	1.1<br>(0.8, 1.6)	20<br>(15, 32)	1.3<br>(1.0, 1.9)	23.2<br>(-7.8, 67.4)
Tajikistan	24<br>(15, 32)	0.9<br>(0.6, 1.2)	43<br>(32, 54)	1.3<br>(0.9, 1.7)	45.0<br>(7.3, 121.9)
Turkmenistan	39<br>(17, 78)	2.3<br>(1.0, 4.4)	155<br>(65, 301)	4.2<br>(1.8, 8.0)	84.9<br>(22.7, 148.3)
Uzbekistan	127<br>(50, 251)	1.2<br>(0.5, 2.4)	464<br>(215, 951)	2.9<br>(1.3, 5.5)	135.0<br>(30.8, 326.5)
Central Europe	32375<br>(15234, 62746)	22.7<br>(10.8, 43.5)	60156<br>(29168, 113573)	27.3<br>(13.2, 51.5)	20.5<br>(-16.2, 58.1)
Albania	30<br>(25, 37)	1.6<br>(1.4, 2.1)	127<br>(89, 177)	2.9<br>(2.1, 4.0)	79.4<br>(20.6, 160.2)
Bosnia and Herzegovina	136<br>(108, 170)	3.8<br>(3.0, 4.7)	391<br>(293, 503)	6.5<br>(4.9, 8.3)	72.2<br>(26.4, 141.2)
Bulgaria	997<br>(425, 2110)	8.3<br>(3.6, 17.5)	1418<br>(646, 2849)	9.6<br>(4.3, 19.3)	15.0<br>(-17.2, 68.0)
Croatia	1995<br>(845, 3776)	32.7<br>(13.8, 60.9)	3148<br>(1349, 6215)	34.3<br>(14.6, 68.7)	5.0<br>(-28.0, 46.9)
Czechia	4003<br>(1841, 7919)	29.4<br>(13.5, 58.1)	6806<br>(3176, 13921)	31.5<br>(14.7, 65.0)	7.2<br>(-34.1, 55.9)
Hungary	10891<br>(4843, 20642)	74.9<br>(33.6, 140.0)	15579<br>(6331, 33709)	78.5<br>(32.9, 169.5)	4.8<br>(-60.1, 47.1)
North Macedonia	68<br>(53, 84)	4.1<br>(3.1, 5.1)	122<br>(91, 160)	4.2<br>(3.1, 5.4)	2.1<br>(-26.3, 43.8)
Montenegro	23<br>(18, 28)	3.8<br>(3.1, 4.7)	44<br>(35, 57)	4.5<br>(3.6, 5.8)	19.3<br>(-11.6, 60.4)
Poland	6215<br>(1192, 13211)	14.5<br>(2.8, 30.6)	16193<br>(5994, 37555)	22.3<br>(8.2, 51.2)	53.1<br>(-24.1, 1,677.4)
Romania	6837<br>(2906, 14259)	25.5<br>(10.8, 52.1)	12174<br>(5244, 24123)	31.9<br>(13.6, 63.4)	25.0<br>(-11.3, 61.9)
Serbia	530<br>(422, 730)	5.0<br>(4.0, 7.0)	2138<br>(1485, 2791)	13.5<br>(9.5, 17.7)	171.8<br>(58.3, 297.2)

Slovakia	258<br>(214, 348)	4.4<br>(3.7, 6.0)	1507<br>(954, 2024)	16.2<br>(10.3, 21.8)	269.9<br>(106.3, 434.6)
Slovenia	393<br>(117, 1196)	16.2<br>(4.8, 49.3)	509<br>(52, 1093)	11.2<br>(1.1, 24.5)	-30.9<br>(-97.8, 68.0)
Eastern Europe	113712<br>(51356, 212969)	42.1<br>(19.4, 77.2)	198594<br>(92879, 386142)	56.1<br>(26.2, 109.4)	33.1<br>(-9.7, 67.0)
Belarus	1237<br>(492, 2456)	9.6<br>(3.9, 19.0)	2680<br>(989, 6000)	16.4<br>(6.0, 36.9)	69.6<br>(13.3, 130.0)
Estonia	36<br>(17, 70)	1.8<br>(0.8, 3.5)	93<br>(36, 184)	3.2<br>(1.2, 6.1)	79.6<br>(-2.3, 500.3)
Latvia	134<br>(42, 274)	3.7<br>(1.2, 7.7)	400<br>(122, 1318)	9.2<br>(2.9, 30.7)	145.7<br>(7.0, 2,415.9)
Lithuania	571<br>(240, 1182)	12.6<br>(5.3, 25.9)	1264<br>(519, 2346)	20.7<br>(8.5, 39.3)	64.4<br>(7.2, 468.9)
Republic of Moldova	203<br>(45, 408)	5.0<br>(1.2, 10.1)	543<br>(215, 1411)	9.3<br>(3.7, 24.0)	85.2<br>(-4.4, 1,868.9)
Russian Federation	79715<br>(35798, 144841)	46.2<br>(20.9, 83.2)	147147<br>(68735, 295787)	61.2<br>(28.6, 122.4)	32.4<br>(-25.2, 78.0)
Ukraine	31817<br>(14702, 62715)	45.7<br>(21.2, 88.2)	46468<br>(21875, 88328)	59.4<br>(27.8, 113.3)	30.0<br>(2.2, 65.6)
High-income Asia Pacific	5845<br>(3382, 10353)	3.1<br>(1.8, 5.5)	14139<br>(7621, 24364)	2.7<br>(1.5, 4.6)	-14.6<br>(-41.6, 19.7)
Brunei Darussalam	9<br>(7, 12)	13.0<br>(9.8, 18.2)	27<br>(22, 34)	14.8<br>(11.7, 18.8)	14.1<br>(-20.4, 56.3)
Japan	4402<br>(2017, 8755)	2.7<br>(1.3, 5.5)	11074<br>(4803, 20824)	2.6<br>(1.1, 4.9)	-6.2<br>(-40.2, 39.5)
Republic of Korea	1352<br>(1100, 1738)	5.6<br>(4.6, 7.1)	2635<br>(2159, 3174)	3.1<br>(2.5, 3.7)	-45.3<br>(-61.1, -28.2)
Singapore	82<br>(40, 161)	4.6<br>(2.2, 8.8)	403<br>(138, 935)	5.5<br>(1.9, 12.7)	20.6<br>(-47.8, 352.4)
Australasia	5888<br>(2706, 10161)	26.1<br>(12.0, 44.9)	15820<br>(7596, 30166)	28.4<br>(13.6, 54.6)	9.2<br>(-45.7, 43.6)
Australia	5017<br>(2312, 8707)	26.8<br>(12.4, 46.3)	13679<br>(6579, 26084)	29.1<br>(13.9, 56.1)	8.9<br>(-46.4, 43.8)
New Zealand	871<br>(398, 1515)	22.6<br>(10.4, 39.5)	2141<br>(1033, 4118)	24.7<br>(11.9, 47.7)	9.2<br>(-41.2, 41.8)
Western Europe	133839<br>(63823, 251490)	22.4<br>(10.6, 42.0)	251178<br>(119208, 479586)	23.9<br>(11.4, 45.4)	7.0<br>(-23.7, 27.6)
Andorra	5<br>(4, 8)	11.5<br>(7.7, 17.5)	33<br>(20, 48)	22.0<br>(13.2, 31.8)	90.8<br>(10.6, 198.6)
Austria	4365<br>(2075, 7891)	35.3<br>(16.8, 64.0)	8487<br>(4071, 16419)	42.1<br>(19.9, 81.9)	19.1<br>(-20.9, 52.9)

Belgium	1971<br>(953, 3649)	12.4<br>(6.0, 23.1)	3740<br>(1777, 7318)	14.2<br>(6.8, 28.3)	14.2<br>(-15.5, 39.6)
Cyprus	73<br>(45, 103)	11.4<br>(6.5, 16.6)	241<br>(164, 298)	13.8<br>(8.8, 17.1)	21.4<br>(-17.6, 84.1)
Denmark	2702<br>(1252, 4807)	31.2<br>(14.4, 55.6)	4022<br>(1972, 7838)	31.7<br>(15.4, 62.4)	1.6<br>(-40.4, 31.4)
Finland	986<br>(403, 1770)	13.8<br>(5.7, 24.9)	2264<br>(959, 4038)	16.0<br>(7.0, 28.5)	15.4<br>(-21.0, 46.4)
France	7560<br>(3649, 14603)	8.6<br>(4.1, 16.7)	13534<br>(5954, 26505)	8.3<br>(3.7, 16.3)	-3.2<br>(-32.1, 44.4)
Germany	48808<br>(22486, 93349)	37.0<br>(17.2, 70.7)	91343<br>(44175, 168799)	42.0<br>(20.1, 78.4)	13.6<br>(-23.7, 132.3)
Greece	639<br>(288, 1162)	4.3<br>(1.9, 7.8)	1312<br>(595, 2354)	4.5<br>(2.1, 8.3)	5.2<br>(-19.9, 28.3)
Iceland	21<br>(10, 40)	7.0<br>(3.4, 13.2)	43<br>(20, 83)	6.8<br>(3.2, 12.9)	-3.9<br>(-33.8, 21.1)
Ireland	1537<br>(684, 2734)	38.3<br>(17.5, 67.8)	2996<br>(1431, 6112)	38.4<br>(18.4, 78.5)	0.3<br>(-55.9, 35.4)
Israel	1279<br>(592, 2434)	27.5<br>(12.8, 52.2)	3952<br>(1916, 7302)	31.9<br>(15.4, 59.0)	16.3<br>(-15.8, 61.0)
Italy	24132<br>(10973, 45346)	26.9<br>(12.2, 50.5)	40476<br>(19189, 79835)	23.8<br>(11.3, 47.4)	-11.4<br>(-51.7, 14.1)
Luxembourg	71<br>(33, 133)	13.0<br>(6.2, 24.2)	150<br>(67, 307)	13.8<br>(6.1, 28.6)	5.9<br>(-36.0, 38.1)
Malta	101<br>(47, 186)	25.1<br>(11.9, 46.3)	272<br>(128, 529)	27.2<br>(12.8, 53.3)	8.4<br>(-25.2, 37.0)
Monaco	7<br>(5, 9)	8.6<br>(6.4, 11.4)	10<br>(8, 13)	9.4<br>(7.1, 11.9)	9.1<br>(-18.9, 49.6)
Netherlands	7478<br>(3643, 13686)	36.5<br>(17.9, 66.9)	14375<br>(6918, 27092)	38.9<br>(18.8, 73.7)	6.3<br>(-13.6, 22.0)
Norway	1271<br>(522, 2293)	17.2<br>(7.1, 31.3)	1832<br>(875, 3523)	17.2<br>(8.0, 33.5)	0.5<br>(-51.9, 28.9)
Portugal	3956<br>(1782, 7058)	30.1<br>(13.5, 52.9)	8809<br>(4182, 17135)	31.6<br>(14.9, 62.0)	5.1<br>(-30.4, 39.8)
Spain	9906<br>(4513, 18410)	18.2<br>(8.2, 33.9)	21008<br>(9729, 40461)	18.7<br>(8.6, 37.0)	2.7<br>(-20.5, 30.1)
Sweden	2080<br>(834, 4216)	12.4<br>(5.0, 24.5)	3122<br>(1475, 5836)	12.5<br>(6.0, 23.6)	0.9<br>(-49.7, 34.7)
Switzerland	1928<br>(829, 3517)	17.3<br>(7.4, 31.8)	3413<br>(1657, 6428)	17.0<br>(8.1, 32.7)	-1.9<br>(-40.4, 25.7)
United Kingdom	12849<br>(4767, 25950)	13.6<br>(5.1, 27.5)	25517<br>(10774, 44848)	18.0<br>(7.7, 32.2)	32.5<br>(-18.7, 423.8)

Southern Latin America	2545<br>(1149, 4852)	5.9<br>(2.7, 11.0)	7536<br>(3622, 14329)	8.8<br>(4.2, 16.7)	50.4<br>(10.1, 82.9)
Argentina	1556<br>(702, 2986)	5.1<br>(2.3, 9.8)	3532<br>(1687, 6811)	6.4<br>(3.0, 12.3)	25.2<br>(-8.1, 45.5)
Chile	938<br>(409, 1788)	10.6<br>(4.7, 20.1)	3912<br>(1853, 7517)	16.2<br>(7.7, 31.0)	53.1<br>(5.1, 107.4)
Uruguay	51<br>(24, 95)	1.3<br>(0.6, 2.4)	92<br>(46, 173)	1.5<br>(0.8, 2.9)	17.8<br>(-46.7, 44.9)
High-income North America	90737<br>(45266, 167062)	25.2<br>(12.5, 46.5)	209196<br>(101860, 395722)	31.6<br>(15.3, 59.8)	25.3<br>(2.2, 43.5)
Canada	7539<br>(3558, 13284)	23.5<br>(11.1, 41.2)	18563<br>(9133, 34811)	25.5<br>(12.7, 48.3)	8.6<br>(-32.7, 35.5)
United States of America	83184<br>(41621, 153996)	25.4<br>(12.7, 47.1)	190609<br>(92046, 360089)	32.3<br>(15.6, 60.9)	27.3<br>(3.6, 46.3)
Greenland	12<br>(10, 15)	45.8<br>(36.6, 57.6)	21<br>(16, 28)	34.8<br>(26.9, 46.0)	-24.0<br>(-43.3, 0.6)
Caribbean	5763<br>(3041, 10575)	23.6<br>(12.5, 43.2)	14588<br>(7719, 25844)	28.1<br>(14.9, 49.7)	19.1<br>(-17.7, 47.1)
Antigua and Barbuda	13<br>(6, 26)	24.2<br>(11.4, 46.4)	31<br>(15, 58)	33.2<br>(15.7, 62.5)	37.3<br>(0.1, 86.6)
Bahamas	46<br>(22, 85)	33.6<br>(16.1, 62.2)	142<br>(68, 265)	39.8<br>(19.1, 75.1)	18.3<br>(-14.7, 51.5)
Barbados	240<br>(118, 448)	79.9<br>(38.9, 148.7)	484<br>(231, 874)	97.4<br>(46.6, 176.1)	22.0<br>(-13.1, 55.8)
Belize	5<br>(2, 10)	5.7<br>(2.6, 11.0)	21<br>(10, 42)	8.5<br>(4.1, 16.8)	49.4<br>(-4.9, 108.3)
Bermuda	29<br>(13, 55)	50.1<br>(22.7, 94.8)	66<br>(33, 123)	47.7<br>(23.4, 89.5)	-4.8<br>(-40.8, 41.0)
Cuba	3297<br>(1556, 6574)	32.7<br>(15.4, 65.1)	8223<br>(3764, 15448)	41.8<br>(19.0, 78.7)	27.6<br>(-18.6, 76.7)
Dominica	14<br>(11, 18)	19.1<br>(15.1, 23.7)	24<br>(19, 30)	26.0<br>(20.4, 32.8)	36.2<br>(3.6, 79.2)
Dominican Republic	86<br>(58, 105)	2.7<br>(1.8, 3.4)	403<br>(287, 541)	4.7<br>(3.3, 6.3)	70.8<br>(24.0, 129.7)
Grenada	23<br>(11, 45)	29.4<br>(14.0, 58.1)	44<br>(21, 82)	43.4<br>(20.3, 80.1)	47.6<br>(-8.3, 94.2)
Guyana	36<br>(15, 72)	10.9<br>(4.6, 21.8)	89<br>(40, 175)	16.1<br>(7.4, 31.7)	47.7<br>(-6.7, 145.3)
Haiti	482<br>(310, 809)	18.0<br>(11.6, 29.4)	1089<br>(688, 1766)	18.5<br>(11.8, 29.8)	2.6<br>(-30.7, 46.5)
Jamaica	410<br>(163, 884)	22.7<br>(9.0, 48.8)	1018<br>(438, 2040)	32.9<br>(14.2, 66.9)	45.0<br>(-19.9, 116.1)

Puerto Rico	723 (338, 1327)	20.5 (9.6, 37.4)	1985 (913, 3976)	25.5 (11.6, 51.1)	24.2 (-19.8, 72.6)
Saint Kitts and Nevis	20 (9, 39)	55.9 (25.3, 108.7)	39 (18, 74)	70.1 (32.9, 135.0)	25.3 (-27.3, 71.3)
Saint Lucia	36 (17, 67)	44.6 (21.5, 85.4)	109 (53, 208)	53.0 (25.5, 101.5)	18.7 (-27.5, 52.3)
Saint Vincent and the Grenadines	12 (6, 23)	18.2 (8.3, 33.4)	32 (15, 60)	25.0 (11.8, 47.2)	37.4 (-12.0, 97.3)
Suriname	7 (5, 8)	2.6 (2.2, 3.2)	20 (16, 26)	3.5 (2.8, 4.4)	31.6 (-0.2, 73.0)
Trinidad and Tobago	66 (26, 131)	8.8 (3.4, 18.2)	184 (77, 371)	10.2 (4.3, 20.4)	15.5 (-33.0, 74.0)
United States Virgin Islands	26 (20, 36)	35.6 (27.2, 50.2)	91 (72, 122)	51.5 (40.9, 68.9)	44.6 (9.9, 89.0)
Andean Latin America	265 (207, 319)	1.5 (1.1, 1.8)	953 (716, 1194)	1.8 (1.3, 2.2)	18.8 (-8.9, 54.6)
Bolivia (Plurinational State of)	47 (30, 68)	1.8 (1.2, 2.5)	194 (124, 278)	2.6 (1.7, 3.6)	46.4 (2.8, 104.9)
Ecuador	52 (43, 68)	1.1 (0.9, 1.4)	274 (207, 356)	2.0 (1.5, 2.6)	83.0 (34.1, 148.8)
Peru	167 (121, 207)	1.6 (1.1, 2.0)	485 (338, 673)	1.5 (1.0, 2.1)	-5.0 (-35.4, 42.4)
Central Latin America	5307 (2527, 9506)	7.5 (3.6, 13.4)	17737 (8566, 35692)	7.9 (3.8, 15.8)	5.5 (-37.9, 45.9)
Colombia	1006 (450, 1828)	6.7 (3.0, 12.2)	3916 (1743, 8228)	7.2 (3.2, 15.2)	7.4 (-44.0, 69.5)
Costa Rica	62 (26, 117)	3.8 (1.6, 7.2)	258 (116, 516)	5.1 (2.3, 10.1)	31.7 (-14.4, 79.4)
El Salvador	28 (21, 33)	1.0 (0.7, 1.2)	80 (53, 107)	1.3 (0.8, 1.7)	26.4 (-8.4, 70.0)
Guatemala	30 (15, 57)	1.1 (0.5, 2.1)	131 (61, 256)	1.3 (0.6, 2.6)	20.7 (-26.5, 87.8)
Honduras	84 (41, 142)	4.9 (2.3, 8.5)	298 (184, 413)	6.0 (3.6, 8.4)	22.3 (-13.8, 80.3)
Mexico	3469 (1626, 6442)	9.6 (4.5, 17.6)	10671 (4868, 21813)	9.8 (4.5, 20.2)	2.1 (-41.6, 47.2)
Nicaragua	17 (14, 21)	1.3 (1.0, 1.5)	83 (64, 101)	2.3 (1.8, 2.8)	77.2 (38.6, 126.0)
Panama	150 (63, 287)	10.8 (4.5, 20.6)	594 (266, 1209)	14.1 (6.3, 28.6)	29.7 (-33.6, 87.8)
Venezuela (Bolivarian Republic of)	461 (218, 845)	5.5 (2.6, 10.2)	1707 (779, 3431)	6.2 (2.9, 12.5)	13.5 (-25.0, 57.8)

Tropical Latin America	13723<br>(6110, 25529)	16.9<br>(7.5, 31.1)	44094<br>(20308, 83166)	18.7<br>(8.6, 35.3)	11.2<br>(-18.9, 46.7)
Brazil	13671<br>(6064, 25480)	17.3<br>(7.7, 31.9)	43732<br>(19959, 82757)	19.0<br>(8.7, 35.9)	10.2<br>(-20.3, 45.1)
Paraguay	52<br>(38, 64)	2.5<br>(1.9, 3.1)	361<br>(254, 484)	6.8<br>(4.8, 9.1)	170.5<br>(85.2, 277.5)
North Africa and Middle East	7515<br>(5859, 9895)	5.1<br>(3.9, 6.8)	22229<br>(18309, 28035)	6.0<br>(4.9, 7.8)	18.2<br>(-12.7, 51.6)
Afghanistan	69<br>(37, 110)	1.2<br>(0.6, 1.8)	183<br>(115, 272)	1.9<br>(1.2, 2.9)	64.2<br>(9.4, 139.9)
Algeria	243<br>(140, 388)	2.8<br>(1.6, 4.4)	1186<br>(739, 1861)	4.4<br>(2.7, 6.7)	55.8<br>(9.1, 126.7)
Bahrain	25<br>(19, 33)	18.7<br>(14.6, 23.9)	144<br>(105, 192)	21.0<br>(15.8, 27.0)	12.2<br>(-24.0, 58.8)
Egypt	296<br>(218, 440)	1.3<br>(1.0, 2.0)	1320<br>(860, 1969)	2.7<br>(1.8, 3.9)	104.7<br>(43.1, 188.5)
Iran (Islamic Republic of)	298<br>(230, 398)	1.6<br>(1.2, 2.3)	2664<br>(1961, 3043)	4.1<br>(3.0, 4.7)	162.8<br>(58.5, 280.5)
Iraq	129<br>(87, 197)	1.7<br>(1.1, 2.8)	421<br>(310, 573)	1.9<br>(1.5, 2.9)	12.9<br>(-25.9, 77.0)
Jordan	12<br>(10, 16)	1.2<br>(1.0, 1.6)	71<br>(56, 92)	1.4<br>(1.1, 1.8)	15.4<br>(-15.7, 52.6)
Kuwait	5<br>(3, 8)	1.0<br>(0.5, 1.9)	31<br>(15, 60)	1.5<br>(0.7, 3.1)	48.4<br>(-9.8, 112.7)
Lebanon	37<br>(24, 52)	2.0<br>(1.3, 2.8)	176<br>(118, 231)	3.4<br>(2.3, 4.5)	74.3<br>(7.0, 172.8)
Libya	18<br>(13, 24)	1.1<br>(0.8, 1.5)	101<br>(64, 140)	2.3<br>(1.4, 3.2)	102.1<br>(27.3, 218.7)
Morocco	152<br>(102, 214)	1.4<br>(0.9, 2.0)	799<br>(529, 1064)	3.1<br>(2.1, 4.2)	124.6<br>(56.6, 207.7)
Palestine	7<br>(5, 10)	1.0<br>(0.6, 1.3)	23<br>(18, 28)	1.3<br>(1.0, 1.6)	29.8<br>(-9.2, 100.9)
Oman	13<br>(7, 21)	2.8<br>(1.4, 4.3)	66<br>(36, 94)	5.8<br>(3.2, 8.4)	110.1<br>(40.7, 229.7)
Qatar	4<br>(2, 5)	7.0<br>(3.4, 10.1)	23<br>(14, 32)	13.1<br>(8.3, 18.3)	86.2<br>(20.3, 236.5)
Saudi Arabia	47<br>(31, 63)	1.0<br>(0.6, 1.3)	152<br>(118, 198)	1.3<br>(1.0, 1.6)	32.7<br>(-13.5, 139.2)
Sudan	104<br>(53, 159)	1.3<br>(0.7, 2.0)	407<br>(238, 612)	2.6<br>(1.6, 3.9)	98.7<br>(35.0, 192.0)
Syrian Arab Republic	40<br>(30, 51)	0.9<br>(0.7, 1.2)	100<br>(74, 133)	1.1<br>(0.8, 1.4)	17.9<br>(-16.8, 69.5)

Tunisia	63<br>(47, 87)	1.6<br>(1.2, 2.1)	342<br>(233, 479)	3.0<br>(2.0, 4.1)	90.5<br>(29.9, 172.2)
Türkiye	5890<br>(4480, 7884)	17.5<br>(13.4, 24.1)	13612<br>(10356, 19399)	15.9<br>(12.1, 23.1)	-9.3<br>(-38.6, 27.2)
United Arab Emirates	9<br>(5, 17)	3.2<br>(1.5, 6.2)	151<br>(70, 289)	5.2<br>(2.4, 9.9)	65.1<br>(-6.2, 195.9)
Yemen	49<br>(28, 79)	1.2<br>(0.7, 2.0)	234<br>(148, 364)	2.2<br>(1.4, 3.4)	76.1<br>(13.8, 166.5)
South Asia	10491<br>(8051, 16863)	2.5<br>(1.9, 4.0)	49750<br>(37108, 63767)	4.2<br>(3.1, 5.3)	67.9<br>(8.9, 113.3)
Bangladesh	835<br>(583, 1517)	2.2<br>(1.5, 3.9)	5256<br>(2795, 7747)	4.6<br>(2.4, 6.8)	113.2<br>(21.6, 233.4)
Bhutan	5<br>(3, 8)	2.5<br>(1.4, 4.2)	29<br>(17, 45)	5.9<br>(3.5, 9.0)	138.1<br>(30.6, 262.9)
India	7915<br>(6138, 12898)	2.4<br>(1.8, 3.9)	38951<br>(29507, 50357)	4.0<br>(3.0, 5.1)	66.1<br>(3.3, 117.0)
Nepal	140<br>(84, 261)	1.9<br>(1.1, 3.5)	809<br>(505, 1138)	4.3<br>(2.7, 6.0)	128.2<br>(37.1, 234.2)
Pakistan	1597<br>(990, 2606)	3.2<br>(2.0, 5.2)	4706<br>(3118, 6843)	5.4<br>(3.6, 7.7)	69.5<br>(16.0, 132.0)
Central Sub-Saharan Africa	7475<br>(2572, 13804)	39.4<br>(13.9, 73.7)	16614<br>(8574, 26885)	36.8<br>(18.9, 58.6)	-6.6<br>(-34.8, 57.1)
Angola	604<br>(348, 930)	18.5<br>(10.1, 27.7)	2436<br>(1377, 3407)	26.6<br>(14.6, 36.6)	44.0<br>(-2.6, 120.2)
Central African Republic	218<br>(112, 358)	21.8<br>(10.6, 34.9)	397<br>(223, 640)	22.2<br>(11.8, 34.6)	1.6<br>(-27.6, 42.5)
Congo	316<br>(179, 450)	34.2<br>(19.6, 48.2)	900<br>(573, 1263)	41.5<br>(25.5, 58.2)	21.3<br>(-17.6, 79.9)
Democratic Republic of the Congo	6119<br>(1727, 11789)	46.9<br>(13.6, 91.4)	12240<br>(5182, 21531)	39.8<br>(17.8, 69.1)	-15.1<br>(-43.3, 50.0)
Equatorial Guinea	36<br>(16, 55)	20.7<br>(9.2, 31.1)	171<br>(74, 297)	43.6<br>(18.7, 73.8)	110.3<br>(16.2, 302.1)
Gabon	181<br>(82, 307)	34.8<br>(15.2, 58.2)	469<br>(282, 677)	50.8<br>(29.9, 72.5)	45.9<br>(-2.2, 131.6)
Eastern Sub-Saharan Africa	10344<br>(5776, 15613)	16.3<br>(8.7, 24.9)	30214<br>(17081, 39880)	22.2<br>(12.0, 29.5)	35.9<br>(2.1, 73.0)
Burundi	379<br>(202, 598)	18.2<br>(9.5, 28.7)	637<br>(337, 962)	17.1<br>(8.6, 25.6)	-6.4<br>(-40.9, 40.4)
Comoros	35<br>(16, 55)	17.4<br>(8.2, 27.7)	87<br>(41, 130)	19.7<br>(9.2, 29.9)	13.3<br>(-27.7, 76.9)
Djibouti	22<br>(11, 36)	20.0<br>(9.2, 32.6)	129<br>(61, 202)	27.1<br>(12.7, 41.1)	35.5<br>(-8.7, 102.5)

Eritrea	108<br>(57, 197)	13.2<br>(6.2, 23.7)	398<br>(202, 601)	18.8<br>(8.4, 29.0)	42.3<br>(-7.3, 126.6)
Ethiopia	2507<br>(1338, 4066)	15.1<br>(8.2, 24.2)	7162<br>(3996, 10275)	20.4<br>(11.2, 29.3)	35.8<br>(-19.3, 111.3)
Kenya	889<br>(493, 1374)	12.7<br>(6.9, 19.5)	3548<br>(1999, 4852)	20.0<br>(10.4, 27.1)	57.1<br>(22.4, 103.6)
Madagascar	975<br>(516, 1517)	21.5<br>(11.4, 33.4)	2445<br>(1251, 3660)	26.5<br>(13.1, 39.3)	23.7<br>(-18.7, 82.5)
Malawi	417<br>(199, 677)	13.2<br>(6.0, 22.0)	1120<br>(578, 1640)	18.1<br>(9.0, 26.2)	37.1<br>(-3.6, 95.3)
Mozambique	786<br>(347, 1172)	15.6<br>(6.6, 23.1)	2539<br>(1396, 3653)	27.3<br>(14.1, 39.0)	74.3<br>(24.0, 162.2)
Rwanda	415<br>(198, 769)	16.7<br>(7.5, 31.4)	887<br>(398, 1407)	17.8<br>(7.5, 28.3)	6.4<br>(-35.7, 59.8)
Somalia	278<br>(135, 497)	13.3<br>(6.2, 23.5)	666<br>(284, 1152)	12.5<br>(4.9, 22.2)	-5.9<br>(-37.8, 35.5)
South Sudan	445<br>(212, 715)	21.0<br>(9.9, 33.3)	696<br>(334, 1112)	21.9<br>(10.0, 33.9)	4.3<br>(-30.7, 58.4)
Uganda	853<br>(282, 1334)	15.0<br>(4.7, 23.4)	2536<br>(1002, 3901)	21.2<br>(8.0, 32.5)	40.9<br>(-0.7, 114.6)
United Republic of Tanzania	1861<br>(937, 2958)	19.8<br>(9.6, 32.0)	5687<br>(2218, 8916)	26.9<br>(10.3, 42.3)	35.9<br>(-13.7, 104.5)
Zambia	367<br>(235, 614)	15.8<br>(9.7, 26.3)	1651<br>(1053, 2588)	30.0<br>(18.9, 46.6)	90.0<br>(23.8, 186.4)
Southern Sub-Saharan Africa	6408<br>(5064, 7559)	24.1<br>(18.9, 28.2)	18702<br>(14785, 21249)	34.8<br>(27.5, 39.7)	44.3<br>(21.2, 67.0)
Botswana	94<br>(58, 135)	16.8<br>(10.7, 23.7)	328<br>(204, 479)	24.7<br>(15.7, 35.5)	46.9<br>(-3.4, 124.9)
Lesotho	93<br>(63, 150)	9.7<br>(6.5, 15.4)	204<br>(135, 291)	17.3<br>(11.2, 24.4)	78.3<br>(20.0, 169.5)
Namibia	126<br>(79, 183)	18.0<br>(11.2, 25.9)	321<br>(208, 457)	23.8<br>(15.4, 33.7)	32.6<br>(-12.3, 94.8)
South Africa	6011<br>(4711, 7129)	29.5<br>(23.1, 34.8)	17649<br>(13998, 20142)	40.9<br>(32.5, 46.7)	38.6<br>(15.5, 61.1)
Eswatini	37<br>(25, 52)	13.1<br>(8.8, 18.1)	110<br>(74, 165)	20.2<br>(13.5, 29.4)	54.9<br>(3.2, 128.1)
Zimbabwe	47<br>(35, 60)	1.4<br>(1.0, 1.8)	91<br>(64, 121)	1.6<br>(1.0, 2.1)	13.2<br>(-15.0, 51.9)
Western Sub-Saharan Africa	3161<br>(2163, 4468)	4.1<br>(2.8, 5.7)	8604<br>(4520, 11702)	5.4<br>(2.9, 7.3)	33.3<br>(-13.6, 89.7)
Benin	72<br>(45, 113)	3.9<br>(2.5, 6.1)	221<br>(114, 343)	5.4<br>(2.8, 8.0)	36.8<br>(-12.3, 112.4)

Burkina Faso	159<br>(90, 279)	3.9<br>(2.3, 6.6)	617<br>(284, 1079)	7.7<br>(3.6, 13.0)	97.6<br>(24.1, 215.0)
Cameroon	195<br>(108, 335)	4.8<br>(2.9, 7.8)	738<br>(390, 1222)	7.5<br>(4.0, 11.9)	55.2<br>(-5.6, 154.9)
Cabo Verde	12<br>(6, 17)	5.0<br>(2.8, 7.4)	36<br>(20, 51)	8.8<br>(4.7, 12.3)	77.8<br>(13.2, 205.3)
Chad	100<br>(56, 158)	3.9<br>(2.2, 6.2)	242<br>(118, 387)	5.1<br>(2.5, 8.0)	29.4<br>(-15.0, 95.0)
Cote d'Ivoire	132<br>(81, 197)	4.1<br>(2.6, 5.8)	437<br>(238, 716)	5.2<br>(3.0, 8.1)	26.1<br>(-18.3, 106.7)
Gambia	11<br>(6, 17)	3.7<br>(2.2, 5.5)	51<br>(25, 79)	6.2<br>(3.1, 9.5)	66.8<br>(2.5, 168.2)
Ghana	124<br>(75, 184)	2.2<br>(1.4, 3.1)	263<br>(187, 427)	1.9<br>(1.4, 3.0)	-11.6<br>(-46.8, 49.1)
Guinea	126<br>(63, 223)	4.0<br>(2.1, 7.0)	266<br>(128, 458)	5.4<br>(2.6, 9.1)	33.4<br>(-15.1, 126.7)
Guinea-Bissau	15<br>(9, 26)	4.2<br>(2.6, 7.1)	35<br>(18, 62)	5.9<br>(3.1, 9.8)	42.1<br>(-16.5, 137.5)
Liberia	42<br>(26, 65)	4.1<br>(2.6, 6.2)	86<br>(44, 144)	5.2<br>(2.7, 8.6)	27.8<br>(-16.3, 98.8)
Mali	139<br>(81, 256)	3.7<br>(2.3, 6.5)	389<br>(195, 662)	5.2<br>(2.7, 8.6)	41.9<br>(-11.3, 133.0)
Mauritania	48<br>(28, 72)	5.5<br>(3.1, 8.2)	119<br>(51, 201)	6.3<br>(2.8, 10.2)	14.6<br>(-30.9, 85.1)
Niger	88<br>(48, 146)	3.6<br>(2.0, 5.8)	313<br>(141, 589)	4.6<br>(2.2, 8.3)	27.1<br>(-19.9, 104.8)
Nigeria	1656<br>(1140, 2449)	4.3<br>(3.0, 6.4)	4050<br>(1941, 6139)	5.6<br>(2.8, 8.3)	30.8<br>(-32.1, 120.0)
Sao Tome and Principe	2<br>(1, 4)	3.9<br>(2.4, 5.6)	6<br>(3, 10)	7.0<br>(3.7, 10.4)	77.6<br>(17.3, 180.1)
Senegal	130<br>(74, 196)	4.5<br>(2.6, 6.6)	405<br>(193, 650)	6.1<br>(3.0, 9.3)	35.0<br>(-11.7, 115.2)
Sierra Leone	59<br>(36, 89)	3.4<br>(2.1, 5.0)	138<br>(69, 232)	4.5<br>(2.3, 7.5)	34.6<br>(-15.0, 115.4)
Togo	50<br>(30, 79)	4.7<br>(2.9, 7.2)	193<br>(90, 317)	6.5<br>(3.1, 10.4)	37.6<br>(-13.3, 117.8)
High SDI	201952<br>(100677, 373964)	19.2<br>(9.6, 35.6)	426939<br>(208630, 793277)	20.6<br>(10.2, 38.4)	7.5<br>(-16.7, 21.1)
High-middle SDI	202197<br>(97628, 362548)	20.8<br>(10.2, 36.8)	384804<br>(201007, 702372)	19.0<br>(10.0, 34.7)	-8.4<br>(-30.2, 8.4)
Middle SDI	32552<br>(24612, 44742)	3.9<br>(2.9, 5.4)	109239<br>(84336, 147880)	4.9<br>(3.8, 6.8)	26.5<br>(3.1, 46.9)

Low-middle SDI	16333<br>(12141, 23174)	3.4<br>(2.5, 4.8)	62647<br>(48113, 78309)	5.2<br>(4.0, 6.6)	53.5<br>(19.3, 81.5)
Low SDI	19334<br>(10143, 28353)	9.5<br>(5.1, 13.9)	51064<br>(29678, 69282)	11.5<br>(6.5, 15.6)	20.9<br>(-7.7, 58.0)

Table S7. DALYs rate (per 100 000 persons) of male and female PAD patient at world bank income level in 2019 by age group. PAD= Peripheral artery disease. DALYs=disability-adjusted life-years. HICs=high-income countries. UMICs=Upper middle-income countries, LMICs=Lower middle-income countries, LICs=Low-income countries.

DALYs rate of PAD	DALYs rate of male PAD patients in 2019					DALYs rate of female PAD patients in 2019				
	HICs	UMLCs	LMICs	LICs	Global	HICs	UMLCs	LMICs	LICs	Global
40-44 years	3.40 (1.28, 7.87)	3.31 (2.17, 5.42)	1.59 (1.26, 2.02)	6.13 (3.34, 9.80)	2.83 (1.93, 4.50)	3.15 (1.22, 6.62)	1.66 (1.02, 2.57)	0.69 (0.48, 0.87)	2.31 (0.54, 4.14)	1.57 (0.97, 2.52)
45-49 years	8.46 (2.98, 20.55)	6.43 (3.73, 12.37)	3.10 (2.29, 4.63)	10.59 (4.95, 17.10)	5.85 (3.49, 10.97)	5.41 (2.10, 11.90)	2.43 (1.35, 4.37)	0.88 (0.58, 1.18)	2.56 (0.63, 5.11)	2.42 (1.35, 4.37)
50-54 years	21.98 (8.40, 49.19)	12.91 (7.54, 24.24)	8.52 (6.32, 11.93)	25.57 (12.60, 40.75)	13.80 (8.34, 24.95)	14.06 (6.57, 28.12)	6.35 (3.82, 10.08)	4.01 (2.54, 5.95)	8.45 (2.54, 15.58)	7.13 (4.33, 11.64)
55-59 years	49.13 (20.66, 102.68)	31.43 (16.68, 58.84)	21.37 (15.14, 30.89)	57.36 (31.03, 89.13)	33.15 (18.78, 59.28)	29.88 (14.88, 54.70)	17.11 (9.52, 28.21)	11.84 (6.70, 20.17)	18.64 (7.07, 31.33)	18.25 (10.31, 29.15)
60-64 years	94.37 (41.64, 194.80)	56.67 (30.09, 109.49)	37.69 (25.60, 55.75)	89.80 (52.09, 137.49)	60.88 (34.46, 111.12)	55.93 (28.63, 102.20)	37.17 (21.50, 61.05)	31.82 (19.23, 48.89)	59.86 (25.68, 97.84)	40.78 (24.70, 64.99)
65-69 years	151.60 (68.16, 314.20)	84.99 (47.42, 161.27)	62.41 (42.74, 94.20)	141.71 (84.76, 203.82)	97.01 (54.80, 175.01)	91.98 (48.91, 161.05)	71.55 (39.81, 121.92)	52.35 (30.86, 86.96)	69.05 (30.02, 112.15)	71.21 (41.04, 119.38)
70-74 years	226.03 (104.02, 463.67)	113.36 (65.44, 211.43)	92.19 (64.57, 133.92)	210.10 (128.57, 298.20)	143.14 (82.09, 257.92)	146.47 (78.04, 254.39)	108.84 (62.36, 184.38)	80.57 (47.71, 133.21)	106.10 (48.28, 171.75)	112.41 (65.72, 181.28)
75-79 years	328.23 (143.45, 673.22)	135.84 (80.17, 239.73)	115.40 (79.91, 171.70)	264.08 (157.54, 381.26)	195.69 (106.54, 355.02)	218.18 (111.15, 395.58)	162.07 (91.99, 266.79)	120.16 (72.06, 200.87)	151.91 (65.13, 241.91)	169.45 (99.32, 275.11)
80-84 years	458.94 (200.61, 1,017.48)	196.81 (113.69, 359.24)	165.80 (112.92, 248.42)	318.49 (193.70, 450.32)	284.47 (150.92, 549.49)	340.99 (161.41, 661.20)	238.72 (137.81, 391.34)	181.02 (113.67, 280.89)	218.43 (91.26, 347.01)	261.96 (147.31, 443.92)
85-89 years	624.07 (263.80, 1,404.07)	249.90 (147.83, 460.81)	181.92 (123.98, 263.40)	349.80 (213.54, 500.15)	393.62 (197.08, 802.49)	491.26 (229.46, 961.64)	285.58 (170.34, 469.92)	223.06 (142.54, 340.94)	264.19 (112.06, 411.20)	363.22 (199.65, 646.11)
90-94 years	774.74 (318.20, 1,689.01)	379.07 (205.56, 727.53)	239.06 (165.68, 344.75)	434.80 (259.01, 643.19)	553.78 (263.58, 1,134.73)	712.97 (322.07, 1,419.50)	403.29 (230.27, 702.87)	317.07 (206.83, 482.34)	329.96 (133.27, 515.25)	552.28 (286.95, 1,021.06)
total (>40 years)	29.38 (13.11, 61.40)	10.42 (6.11, 19.24)	5.82 (4.14, 8.40)	10.64 (6.58, 14.69)	12.64 (7.20, 23.16)	26.37 (13.26, 47.89)	10.98 (6.65, 17.54)	5.70 (3.60, 8.59)	6.17 (2.65, 9.74)	12.09 (7.28, 19.13)

Table S8. Mortality rate (per 100 000 persons) of male and female PAD patient at world bank income level in 2019 by age group. PAD= Peripheral artery disease. UMICs=Upper middle-income countries, LMICs=Lower middle-income countries, LICs=Low-income countries.

Mortality rate of PAD	Mortality rate of male PAD patients in 2019					Mortality rate of female PAD patients in 2019				
	HICs	UMLCs	LMICs	LICs	Global	HICs	UMLCs	LMICs	LICs	Global
40-44 years	0.07 (0.03, 0.17)	0.07 (0.05, 0.12)	0.03 (0.03, 0.04)	0.13 (0.07, 0.21)	0.06 (0.04, 0.10)	0.07 (0.03, 0.14)	0.04 (0.02, 0.05)	0.01 (0.01, 0.02)	0.05 (0.01, 0.09)	0.03 (0.02, 0.05)
45-49 years	0.20 (0.07, 0.49)	0.15 (0.09, 0.29)	0.07 (0.05, 0.11)	0.25 (0.12, 0.41)	0.14 (0.08, 0.26)	0.13 (0.05, 0.28)	0.06 (0.03, 0.10)	0.02 (0.01, 0.03)	0.06 (0.01, 0.12)	0.06 (0.03, 0.10)
50-54 years	0.55 (0.18, 1.28)	0.32 (0.18, 0.62)	0.20 (0.14, 0.28)	0.65 (0.30, 1.06)	0.34 (0.19, 0.63)	0.31 (0.11, 0.68)	0.11 (0.06, 0.21)	0.06 (0.04, 0.09)	0.18 (0.03, 0.38)	0.14 (0.07, 0.25)
55-59 years	1.26 (0.41, 2.95)	0.83 (0.41, 1.67)	0.49 (0.35, 0.73)	1.58 (0.77, 2.56)	0.84 (0.45, 1.62)	0.62 (0.22, 1.37)	0.24 (0.12, 0.44)	0.12 (0.07, 0.18)	0.34 (0.06, 0.68)	0.29 (0.14, 0.55)
60-64 years	2.70 (0.92, 6.49)	1.65 (0.75, 3.54)	0.90 (0.61, 1.47)	2.73 (1.38, 4.35)	1.71 (0.84, 3.50)	1.28 (0.44, 2.83)	0.52 (0.24, 1.04)	0.47 (0.26, 0.67)	1.51 (0.36, 2.83)	0.72 (0.38, 1.34)
65-69 years	5.02 (1.66, 11.76)	2.76 (1.29, 5.93)	1.65 (1.13, 2.69)	4.96 (2.55, 7.45)	3.08 (1.49, 6.35)	2.28 (0.84, 4.98)	1.06 (0.53, 2.05)	0.66 (0.44, 1.03)	1.49 (0.28, 2.92)	1.27 (0.63, 2.48)
70-74 years	9.18 (3.08, 21.20)	4.32 (2.11, 9.04)	2.91 (2.05, 4.36)	8.91 (5.01, 13.00)	5.49 (2.71, 11.20)	4.48 (1.59, 9.60)	1.81 (0.88, 3.43)	1.17 (0.75, 1.71)	2.70 (0.53, 4.94)	2.45 (1.10, 4.76)
75-79 years	17.73 (5.99, 40.74)	6.45 (3.31, 12.85)	4.55 (3.19, 6.87)	14.10 (7.72, 21.06)	9.78 (4.61, 20.07)	9.42 (3.38, 20.96)	4.28 (2.11, 8.56)	2.98 (1.95, 4.64)	5.29 (1.02, 10.06)	5.66 (2.63, 11.51)
80-84 years	33.43 (11.38, 82.07)	12.93 (6.44, 26.42)	9.27 (6.20, 14.42)	21.98 (12.46, 31.93)	19.49 (8.69, 42.89)	21.49 (7.36, 48.83)	10.47 (4.90, 22.21)	7.56 (4.51, 11.82)	11.10 (2.25, 19.63)	13.77 (5.93, 28.94)
85-89 years	61.03 (21.44, 148.58)	21.77 (11.50, 44.73)	12.55 (8.62, 19.15)	30.85 (17.23, 45.40)	36.38 (15.78, 82.38)	43.35 (14.85, 96.95)	17.74 (8.49, 36.49)	12.81 (7.76, 19.83)	18.07 (3.71, 32.49)	27.96 (11.53, 58.74)
90-94 years	98.53 (33.33, 233.07)	44.54 (20.90, 95.00)	22.78 (15.29, 34.59)	50.52 (27.34, 77.65)	67.82 (27.91, 152.39)	86.06 (29.87, 182.08)	39.02 (17.68, 80.30)	28.36 (17.27, 47.22)	31.45 (6.63, 55.64)	62.03 (24.38, 126.13)
total (>40 years)	4.59 (1.61, 10.74)	1.09 (0.55, 2.22)	0.46 (0.32, 0.70)	0.92 (0.52, 1.27)	1.48 (0.69, 3.11)	4.82 (1.70, 10.52)	0.93 (0.44, 1.87)	0.35 (0.23, 0.54)	0.45 (0.09, 0.83)	1.39 (0.60, 2.87)

Table S9. Prevalence rate (per 100 000 persons) of male and female PAD patient at world bank income level in 2019 by age group. PAD= Peripheral artery disease. HICs=high-income countries. UMICs=Upper middle-income countries, LMICs=Lower middle-income countries, LICs=Low-income countries.

Prevalence rate of PAD	Prevalence rate of male PAD patients in 2019					Prevalence rate of female PAD patients in 2019				
	HICs	UMLCs	LMICs	LICs	Global	HICs	UMLCs	LMICs	LICs	Global
40-44 years	476.67 (361.81, 618.48)	308.44 (227, 411.98)	300.48 (219.9, 401.32)	279.74 (203.25, 373.17)	332.32 (245.59, 439.62)	1225.99 (950.36, 1544.51)	598.9 (442.59, 774.72)	432.89 (316.36, 571.03)	355.35 (258.92, 472.22)	621.11 (468.32, 805.97)
45-49 years	991.86 (792.49, 1213.95)	670.17 (521.03, 841.9)	656.22 (509.78, 827.73)	619.63 (477.27, 780.75)	720.01 (563.58, 900.6)	2068.94 (1684.43, 2481.61)	1450.82 (1120.72, 1824.83)	987.46 (761.3, 1244)	812.94 (624.54, 1023.86)	1361.61 (1074.07, 1679.39)
50-54 years	1810.13 (1429.98, 2231.66)	1201.94 (921.31, 1526.98)	1185.15 (904.62, 1513.22)	1119.94 (852.85, 1435.31)	1307.68 (1008.97, 1653.38)	2974.64 (2389.21, 3626.58)	2765.72 (2105.13, 3576.12)	1835.83 (1394.05, 2368.8)	1511.71 (1147.16, 1947.21)	2448.68 (1888.76, 3102.94)
55-59 years	3089.05 (2526.08, 3646.83)	1875.03 (1480.55, 2267.96)	1871.35 (1474.47, 2272.73)	1765.62 (1382.79, 2137.84)	2132.01 (1701.97, 2566.04)	4097.07 (3348.88, 4860.65)	4287.99 (3340.19, 5225.06)	2948.6 (2309.33, 3581.85)	2410.91 (1885.47, 2945.61)	3744.99 (2967.44, 4509.31)
60-64 years	4804.04 (3905.01, 5802.85)	2669.15 (2117.11, 3263.82)	2673.41 (2120.19, 3296.87)	2566.6 (2021.8, 3165.38)	3161.94 (2530.89, 3859.06)	5737.17 (4705.06, 6856.42)	6118 (4780.12, 7464.96)	4288.2 (3365.38, 5267.36)	3552.33 (2791.65, 4402.26)	5375.07 (4255.67, 6510.99)
65-69 years	7288.43 (6040.72, 8646.65)	3726.13 (3046.52, 4543.76)	3804.88 (3095.29, 4646.59)	3748.07 (3022.29, 4603.04)	4634.49 (3800.36, 5610.01)	8984.76 (7459.69, 10615.08)	8781.77 (7134.84, 10665.53)	6267.08 (5084.66, 7658.75)	5284.22 (4260.36, 6473.18)	8011.52 (6583.39, 9646.16)
70-74 years	10266.12 (8251.89, 12476.05)	5011.73 (3926.73, 6180.02)	5225.31 (4075.14, 6488.1)	5265.9 (4070.97, 6570.2)	6586.78 (5226.11, 8055.36)	13367.62 (10688.56, 16290.41)	11762.61 (9252.93, 14545.61)	8770.93 (6820.89, 10889.23)	7627.57 (5907.8, 9544.08)	11316.4 (8945.28, 13891.09)
75-79 years	12957.18 (10664.21, 15404.94)	6348.97 (5114.47, 7686.21)	6770.05 (5469.88, 8206.42)	6861.65 (5555.78, 8283.74)	8577.22 (6977.68, 10285.78)	17629.94 (14572.6, 21092.22)	14632.99 (11828.59, 17691.01)	11378.2 (9138.09, 13789.41)	10061.04 (8053.13, 12258.68)	14641.67 (11879.77, 17657.93)
80-84 years	15373.93 (12953.72, 18292.95)	7702.91 (6299.31, 9411.96)	8366.09 (6835.55, 10267.04)	8455.66 (6912.24, 10378.84)	10555.47 (8777.92, 12755.76)	21543.51 (18078.95, 25543)	17471.9 (14320.48, 21154.61)	14012.11 (11484.14, 17015.63)	12338.18 (10111.26, 15087)	18028.44 (15012.72, 21631.97)
85-89 years	17315.04 (14663.16, 20358.57)	9185.54 (7567.15, 10988.89)	9837.12 (8126.3, 11772.05)	9880.72 (8124.7, 11817.86)	12722.2 (10687.32, 15045.47)	24593.05 (20810.37, 29013.42)	19162 (15856.93, 23017.9)	15817.14 (13083.13, 18840.01)	13963.07 (11458.31, 16692.92)	20824.09 (17439.86, 24726.07)
90-94 years	18903.8 (16202.16, 21950.13)	11328.99 (9362.27, 13548.97)	11212.84 (9212.37, 13401.34)	11180.99 (9257.63, 13368.93)	15159.52 (12793.11, 17760.85)	26801.02 (22938.83, 31046.67)	20350.53 (17076.29, 24083.94)	17374.92 (14579.18, 20575.81)	15108.66 (12564.96, 17990.35)	23236.74 (19774.84, 27126.1)
All ages (>40 years)	2548.31 (2228.35, 2878.98)	930.9 (800.45, 1069.99)	542.88 (466.83, 621.32)	303.43 (261.16, 349.29)	962.38 (837.8, 1098.87)	4330.25 (3805.53, 4852.23)	2481.06 (2149.72, 2833.22)	983.22 (853.32, 1123.37)	486.04 (418.25, 556.15)	1973.12 (1726.63, 2234.45)

Table S10. Incidence rate (per 100 000 persons) of male and female PAD patient at world bank income level in 2019 by age group. PAD= Peripheral artery disease. HICs=high-income countries. UMICs=Upper middle-income countries, LMICs=Lower middle-income countries, LICs=Low-income countries.

Incidence rate of PAD	Incidence rate of male PAD patients in 2019					Incidence rate of female PAD patients in 2019				
	HICs	UMLCs	LMICs	LICs	Global	HICs	UMLCs	LMICs	LICs	Global
40-44 years	81.31 (64.83, 99.86)	55.39 (42.87, 69.73)	54.54 (42.21, 68.81)	51.23 (39.41, 64.73)	59.23 (46.36, 74.24)	165.40 (135.15, 198.21)	116.67 (90.05, 147.57)	81.78 (62.94, 103.58)	66.01 (50.60, 83.26)	107.77 (84.52, 133.86)
45-49 years	127.06 (85.04, 176.02)	92.34 (61.44, 128.05)	91.60 (60.20, 128.32)	87.68 (57.72, 123.53)	98.03 (65.24, 136.31)	170.93 (114.32, 240.50)	219.15 (142.86, 308.17)	143.93 (94.89, 202.31)	116.79 (75.97, 165.21)	178.75 (117.65, 250.84)
50-54 years	225.69 (172.29, 277.56)	141.86 (105.94, 177.91)	144.42 (107.30, 181.10)	140.84 (104.82, 177.01)	158.47 (119.25, 197.33)	223.98 (158.69, 293.79)	329.67 (244.65, 416.24)	226.32 (167.47, 285.85)	190.95 (142.37, 240.40)	270.51 (198.70, 342.69)
55-59 years	374.66 (250.13, 499.94)	203.47 (133.46, 278.92)	211.92 (136.51, 292.96)	208.58 (135.50, 288.20)	243.39 (159.92, 332.68)	323.48 (199.27, 462.54)	435.89 (263.11, 613.02)	327.87 (201.01, 458.51)	283.18 (177.08, 399.66)	371.30 (225.60, 522.31)
60-64 years	594.70 (471.41, 729.22)	291.05 (220.34, 369.35)	311.36 (233.77, 394.46)	313.25 (235.39, 398.04)	368.53 (285.34, 460.65)	624.59 (480.69, 785.35)	624.97 (461.03, 806.57)	496.29 (371.04, 634.33)	438.69 (328.72, 558.39)	578.64 (433.04, 738.67)
65-69 years	860.96 (598.62, 1,144.28)	397.46 (268.23, 555.10)	438.81 (295.10, 614.66)	451.60 (298.97, 625.81)	526.21 (360.71, 720.55)	1,091.77 (751.95, 1,468.20)	885.46 (582.24, 1,260.11)	721.25 (482.06, 1,011.75)	647.87 (428.44, 910.82)	882.99 (593.71, 1,228.61)
70-74 years	959.53 (738.96, 1,220.26)	473.64 (343.61, 617.27)	531.27 (386.56, 686.02)	555.39 (405.69, 718.00)	631.27 (471.66, 813.32)	1,293.95 (1,006.53, 1,617.91)	1,012.03 (737.76, 1,346.37)	853.20 (629.49, 1,123.22)	795.11 (583.67, 1,035.84)	1,045.85 (784.40, 1,350.18)
75-79 years	925.36 (653.34, 1,230.74)	521.07 (357.63, 714.16)	596.58 (406.40, 815.11)	626.80 (427.40, 854.58)	672.50 (466.98, 909.00)	1,273.36 (887.26, 1,714.64)	1,039.65 (704.87, 1,408.17)	911.71 (623.65, 1,240.99)	879.11 (601.66, 1,198.40)	1,078.23 (740.38, 1,457.71)
80-84 years	874.65 (621.00, 1,192.61)	554.46 (396.48, 759.25)	639.27 (454.84, 870.82)	665.59 (478.37, 903.88)	688.63 (491.59, 939.72)	1,190.60 (841.24, 1,618.69)	988.89 (678.24, 1,370.02)	905.91 (630.31, 1,243.70)	885.32 (632.27, 1,221.98)	1,039.77 (721.48, 1,431.18)
85-89 years	807.06 (584.84, 1,068.20)	582.94 (413.71, 784.07)	658.20 (467.21, 891.19)	678.03 (483.87, 916.44)	694.08 (497.53, 926.01)	1,069.52 (774.93, 1,420.19)	854.99 (605.35, 1,159.06)	827.39 (588.75, 1,124.17)	822.44 (585.91, 1,115.57)	942.18 (677.37, 1,260.51)
90-94 years	734.73 (504.00, 1,019.59)	666.09 (466.87, 917.99)	699.90 (494.33, 969.03)	703.87 (494.66, 970.95)	708.46 (497.23, 976.55)	937.06 (639.55, 1,290.12)	785.17 (524.07, 1,114.84)	787.34 (536.08, 1,106.63)	784.97 (535.39, 1,079.39)	864.65 (586.89, 1,204.60)

**total (>40 years)**	303.45 (265.53, 345.55)	128.29 (110.19, 147.34)	99.51 (85.53, 114.16)	76.02 (65.61, 86.88)	145.99 (126.89, 166.79)	426.66 (376.72, 484.85)	296.85 (257.50, 340.60)	165.66 (143.15, 190.16)	111.34 (96.39, 127.04)	260.09 (226.90, 297.29)

Table S11. Age-standardised incidence rate due to PAD in 2019 for both sexes by SDI group. PAD= Peripheral artery disease. SDI= sociodemographic index. UI = Uncertainty interval.

Region	Sex	Age-standardised incidence rate	Lower UI	Upper UI
**Global**	Males	96.18	83.93	109.99
**Global**	Females	154.91	135.53	177.03
**Global**	Both sexes	127.11	111.28	145.44
**High SDI**	Males	140.87	124.03	158.76
**High SDI**	Females	172.11	152.18	195.05
**High SDI**	Both sexes	157.66	139.45	178.54
**High-middle SDI**	Males	90.27	78.08	103.4
**High-middle SDI**	Females	171.36	149.5	196
**High-middle SDI**	Both sexes	133.72	116.35	153.3
**Middle SDI**	Males	81.28	70.32	93.15
**Middle SDI**	Females	151.62	131.61	173.73
**Middle SDI**	Both sexes	117.85	102.37	135.08
**Low-middle SDI**	Males	80.75	69.82	92.63
**Low-middle SDI**	Females	125.13	108.85	143.29
**Low-middle SDI**	Both sexes	103.84	90.03	118.93
**Low SDI**	Males	82.02	70.87	93.97
**Low SDI**	Females	113.4	98.28	129.65
**Low SDI**	Both sexes	98.03	84.89	111.96

Table S12. Age-standardised DALYs rate (per 100 000 persons) of PAD attributed to risk factors for males, females, and sex combined in 2019. PAD= Peripheral artery disease. DALY= disability-adjusted life-years. UI = Uncertainty interval.

Risk factors	Sex	Age-standardised DALY rate attributed to risk factors	Lower UI	Upper UI
Lead exposure	Males	0.32	0.15	0.63
Tobacco	Males	9.47	5.11	17.15
High fasting plasma glucose	Males	6.40	3.50	11.95
High systolic blood pressure	Males	5.88	3.15	10.80
Diet high in sodium	Males	0.88	0.18	2.35
Kidney dysfunction	Males	3.56	1.91	6.84
Lead exposure	Females	0.20	0.07	0.42
Tobacco	Females	3.12	1.70	5.37
High fasting plasma glucose	Females	4.61	2.74	7.64
High systolic blood pressure	Females	4.83	2.70	8.29
Diet high in sodium	Females	0.53	0.09	1.49
Kidney dysfunction	Females	2.69	1.54	4.44
Diet high in sodium	Both sexes	0.69	0.14	1.82
Kidney dysfunction	Both sexes	3.11	1.92	4.90
Lead exposure	Both sexes	0.25	0.11	0.48
Tobacco	Both sexes	6.06	3.63	9.90
High fasting plasma glucose	Both sexes	5.43	3.48	8.52
High systolic blood pressure	Both sexes	5.34	3.34	8.53

Table S13. Estimated age-specific prevalence (%) of women and men living with peripheral artery disease in 204 countries and territories in 2019. UI = Uncertainty interval.

Location	Sex	Age	Prevalence (%)	Lower UI	Upper UI	Location	Sex	Age	Prevalence (%)	Lower UI	Upper UI	Location	Sex	Age	Prevalence (%)	Lower UI	Upper UI
Afghanistan	Both	40-44 years	0.39	0.28	0.52	Afghanistan	Male	50-54 years	1.26	0.94	1.61	Afghanistan	Female	50-54 years	1.62	1.22	2.09
Afghanistan	Both	45-49 years	0.82	0.63	1.03	Afghanistan	Male	55-59 years	1.96	1.53	2.39	Afghanistan	Female	55-59 years	2.53	1.97	3.14
Afghanistan	Both	50-54 years	1.43	1.09	1.85	Afghanistan	Male	60-64 years	2.83	2.20	3.54	Afghanistan	Female	60-64 years	3.67	2.90	4.58
Afghanistan	Both	55-59 years	2.26	1.77	2.78	Afghanistan	Male	65-69 years	4.19	3.32	5.17	Afghanistan	Female	65-69 years	5.51	4.36	6.78
Afghanistan	Both	60-64 years	3.29	2.58	4.12	Afghanistan	Male	70-74 years	5.96	4.62	7.47	Afghanistan	Female	70-74 years	7.89	6.00	9.99
Afghanistan	Both	65-69 years	4.93	3.92	6.04	Afghanistan	Male	75-79 years	7.80	6.25	9.46	Afghanistan	Female	75-79 years	10.28	8.23	12.57
Afghanistan	Both	70-74 years	7.02	5.39	8.79	Afghanistan	Male	All ages	0.27	0.23	0.31	Afghanistan	Female	All ages	0.38	0.32	0.44
Afghanistan	Both	75-79 years	9.10	7.29	11.02	Afghanistan	Male	80-84	9.56	7.69	11.71	Afghanistan	Female	80-84	12.47	10.17	15.24
Afghanistan	Both	All ages	0.32	0.28	0.37	Afghanistan	Male	85-89	11.07	9.01	13.36	Afghanistan	Female	85-89	14.09	11.45	16.98
Afghanistan	Both	80-84	11.00	8.97	13.34	Afghanistan	Male	90-94	12.35	10.14	14.86	Afghanistan	Female	90-94	15.21	12.54	18.39
Afghanistan	Both	85-89	12.50	10.24	14.96	Afghanistan	Male	40-44 years	0.34	0.25	0.46	Afghanistan	Female	40-44 years	0.43	0.32	0.59
Afghanistan	Both	90-94	13.65	11.28	16.35	Afghanistan	Male	45-49 years	0.72	0.55	0.91	Afghanistan	Female	45-49 years	0.92	0.71	1.17
Albania	Both	80-84	12.73	10.26	15.39	Albania	Male	80-84	11.13	9.02	13.71	Albania	Female	40-44 years	0.47	0.34	0.64
Albania	Both	85-89	14.62	11.96	17.45	Albania	Male	85-89	12.85	10.50	15.61	Albania	Female	45-49 years	1.00	0.77	1.28
Albania	Both	90-94	16.20	13.45	19.33	Albania	Male	90-94	14.35	11.83	17.16	Albania	Female	50-54 years	1.79	1.35	2.35
Albania	Both	40-44 years	0.44	0.32	0.59	Albania	Male	40-44 years	0.41	0.30	0.55	Albania	Female	55-59 years	2.89	2.27	3.59
Albania	Both	45-49 years	0.92	0.72	1.17	Albania	Male	45-49 years	0.84	0.66	1.05	Albania	Female	60-64 years	4.33	3.41	5.34
Albania	Both	50-54 years	1.63	1.24	2.10	Albania	Male	50-54 years	1.46	1.11	1.85	Albania	Female	65-69 years	6.48	5.25	7.90
Albania	Both	55-59 years	2.61	2.05	3.19	Albania	Male	55-59 years	2.32	1.82	2.82	Albania	Female	70-74 years	9.12	6.95	11.41
Albania	Both	60-64 years	3.88	3.07	4.75	Albania	Male	60-64 years	3.43	2.71	4.24	Albania	Female	75-79 years	11.70	9.25	14.27
Albania	Both	65-69 years	5.77	4.66	7.01	Albania	Male	65-69 years	5.05	4.04	6.18	Albania	Female	All ages	2.37	2.03	2.71

Albania	Both	70-74 years	8.11	6.26	10.07	Albania	Male	70-74 years	7.05	5.45	8.85	Albania	Female	80-84	14.03	11.26	16.92
Albania	Both	75-79 years	10.48	8.36	12.76	Albania	Male	75-79 years	9.12	7.31	11.06	Albania	Female	85-89	15.78	12.90	19.01
Albania	Both	All ages	2.04	1.75	2.34	Albania	Male	All ages	1.70	1.45	1.96	Albania	Female	90-94	17.01	14.03	20.51
Algeria	Both	65-69 years	5.21	4.19	6.37	Algeria	Male	40-44 years	0.35	0.26	0.48	Algeria	Female	65-69 years	5.96	4.77	7.30
Algeria	Both	70-74 years	7.42	5.69	9.37	Algeria	Male	45-49 years	0.75	0.58	0.94	Algeria	Female	70-74 years	8.48	6.44	10.81
Algeria	Both	75-79 years	9.64	7.65	11.88	Algeria	Male	50-54 years	1.32	0.99	1.68	Algeria	Female	75-79 years	10.99	8.63	13.58
Algeria	Both	All ages	0.90	0.77	1.03	Algeria	Male	55-59 years	2.08	1.63	2.57	Algeria	Female	All ages	1.00	0.85	1.15
Algeria	Both	80-84	11.77	9.52	14.31	Algeria	Male	60-64 years	3.03	2.37	3.77	Algeria	Female	80-84	13.33	10.71	16.25
Algeria	Both	85-89	13.38	10.97	16.10	Algeria	Male	65-69 years	4.50	3.61	5.56	Algeria	Female	85-89	15.20	12.45	18.46
Algeria	Both	90-94	14.23	11.68	17.02	Algeria	Male	70-74 years	6.38	4.93	8.03	Algeria	Female	90-94	16.64	13.68	19.89
Algeria	Both	40-44 years	0.40	0.29	0.55	Algeria	Male	75-79 years	8.31	6.68	10.16	Algeria	Female	40-44 years	0.45	0.32	0.61
Algeria	Both	45-49 years	0.85	0.66	1.06	Algeria	Male	All ages	0.79	0.68	0.92	Algeria	Female	45-49 years	0.95	0.73	1.18
Algeria	Both	50-54 years	1.50	1.13	1.89	Algeria	Male	80-84	10.19	8.22	12.50	Algeria	Female	50-54 years	1.69	1.26	2.14
Algeria	Both	55-59 years	2.38	1.87	2.92	Algeria	Male	85-89	11.86	9.73	14.35	Algeria	Female	55-59 years	2.69	2.10	3.32
Algeria	Both	60-64 years	3.49	2.73	4.32	Algeria	Male	90-94	13.38	10.96	16.00	Algeria	Female	60-64 years	3.98	3.12	4.95
American Samoa	Both	40-44 years	0.64	0.48	0.84	American Samoa	Male	40-44 years	0.57	0.43	0.77	American Samoa	Female	40-44 years	0.71	0.52	0.94
American Samoa	Both	45-49 years	1.41	1.09	1.78	American Samoa	Male	45-49 years	1.24	0.98	1.56	American Samoa	Female	45-49 years	1.58	1.19	2.00
American Samoa	Both	50-54 years	2.54	1.92	3.26	American Samoa	Male	50-54 years	2.21	1.68	2.84	American Samoa	Female	50-54 years	2.86	2.14	3.72
American Samoa	Both	55-59 years	3.96	3.09	4.84	American Samoa	Male	55-59 years	3.42	2.68	4.21	American Samoa	Female	55-59 years	4.48	3.49	5.51
American Samoa	Both	60-64 years	5.64	4.43	6.99	American Samoa	Male	60-64 years	4.82	3.80	6.06	American Samoa	Female	60-64 years	6.40	5.00	7.96
American Samoa	Both	65-69 years	8.00	6.48	9.82	American Samoa	Male	65-69 years	6.72	5.40	8.34	American Samoa	Female	65-69 years	9.23	7.41	11.34
American Samoa	Both	70-74 years	10.91	8.49	13.67	American Samoa	Male	70-74 years	9.01	7.02	11.14	American Samoa	Female	70-74 years	12.72	9.89	16.00
American Samoa	Both	75-79 years	13.77	11.10	16.84	American Samoa	Male	75-79 years	11.24	9.00	13.74	American Samoa	Female	75-79 years	16.09	12.72	20.03
American Samoa	Both	All ages	1.48	1.27	1.70	American Samoa	Male	All ages	1.21	1.03	1.39	American Samoa	Female	All ages	1.75	1.50	2.01

American Samoa	Both	80-84	16.48	13.41	20.05	American Samoa	Male	80-84	13.27	10.76	16.13	American Samoa	Female	80-84	19.09	15.58	23.20
American Samoa	Both	85-89	18.61	15.38	22.41	American Samoa	Male	85-89	14.83	12.24	17.79	American Samoa	Female	85-89	21.16	17.47	25.49
American Samoa	Both	90-94	20.16	16.79	24.02	American Samoa	Male	90-94	16.01	13.31	19.13	American Samoa	Female	90-94	22.42	18.67	26.83
Andorra	Both	40-44 years	0.95	0.71	1.26	Andorra	Male	40-44 years	0.53	0.38	0.72	Andorra	Female	40-44 years	1.39	1.05	1.84
Andorra	Both	45-49 years	1.66	1.31	2.07	Andorra	Male	45-49 years	1.09	0.84	1.39	Andorra	Female	45-49 years	2.26	1.76	2.86
Andorra	Both	50-54 years	2.53	1.96	3.17	Andorra	Male	50-54 years	1.95	1.48	2.47	Andorra	Female	50-54 years	3.14	2.43	3.93
Andorra	Both	55-59 years	3.77	3.00	4.52	Andorra	Male	55-59 years	3.29	2.60	3.99	Andorra	Female	55-59 years	4.28	3.42	5.18
Andorra	Both	60-64 years	5.54	4.48	6.74	Andorra	Male	60-64 years	5.12	4.01	6.33	Andorra	Female	60-64 years	6.02	4.78	7.31
Andorra	Both	65-69 years	8.56	6.89	10.32	Andorra	Male	65-69 years	7.77	6.20	9.54	Andorra	Female	65-69 years	9.44	7.69	11.42
Andorra	Both	70-74 years	12.40	9.80	15.30	Andorra	Male	70-74 years	10.94	8.54	13.72	Andorra	Female	70-74 years	14.06	11.11	17.57
Andorra	Both	75-79 years	16.24	13.15	19.70	Andorra	Male	75-79 years	13.91	11.22	17.11	Andorra	Female	75-79 years	18.44	14.93	22.32
Andorra	Both	All ages	3.38	2.92	3.83	Andorra	Male	All ages	2.79	2.38	3.21	Andorra	Female	All ages	3.98	3.44	4.51
Andorra	Both	80-84	19.64	16.28	23.58	Andorra	Male	80-84	16.51	13.52	20.07	Andorra	Female	80-84	22.28	18.51	26.77
Andorra	Both	85-89	22.38	18.68	26.46	Andorra	Male	85-89	18.51	15.30	22.27	Andorra	Female	85-89	25.22	21.05	29.87
Andorra	Both	90-94	24.52	20.79	28.68	Andorra	Male	90-94	19.88	16.56	23.45	Andorra	Female	90-94	27.20	23.00	31.76
Angola	Both	40-44 years	0.36	0.26	0.49	Angola	Male	40-44 years	0.30	0.22	0.41	Angola	Female	40-44 years	0.42	0.30	0.57
Angola	Both	45-49 years	0.81	0.63	1.02	Angola	Male	45-49 years	0.67	0.51	0.85	Angola	Female	45-49 years	0.94	0.72	1.18
Angola	Both	50-54 years	1.49	1.12	1.91	Angola	Male	50-54 years	1.22	0.92	1.54	Angola	Female	50-54 years	1.74	1.30	2.24
Angola	Both	55-59 years	2.44	1.90	2.98	Angola	Male	55-59 years	1.97	1.55	2.40	Angola	Female	55-59 years	2.84	2.21	3.51
Angola	Both	60-64 years	3.64	2.86	4.51	Angola	Male	60-64 years	2.91	2.30	3.63	Angola	Female	60-64 years	4.28	3.34	5.34
Angola	Both	65-69 years	5.58	4.49	6.81	Angola	Male	65-69 years	4.34	3.48	5.37	Angola	Female	65-69 years	6.60	5.27	8.04
Angola	Both	70-74 years	8.13	6.19	10.24	Angola	Male	70-74 years	6.16	4.73	7.77	Angola	Female	70-74 years	9.62	7.19	12.14
Angola	Both	75-79 years	10.78	8.63	13.15	Angola	Male	75-79 years	8.13	6.44	9.97	Angola	Female	75-79 years	12.68	10.11	15.40
Angola	Both	All ages	0.39	0.34	0.45	Angola	Male	All ages	0.29	0.24	0.33	Angola	Female	All ages	0.49	0.42	0.56

Angola	Both	80-84	13.35	10.82	16.18	Angola	Male	80-84	10.11	8.13	12.33	Angola	Female	80-84	15.54	12.64	18.87
Angola	Both	85-89	15.59	12.87	18.70	Angola	Male	85-89	11.85	9.73	14.23	Angola	Female	85-89	17.70	14.47	21.33
Angola	Both	90-94	17.46	14.48	20.87	Angola	Male	90-94	13.38	11.01	16.07	Angola	Female	90-94	19.20	15.88	22.96
Antigua and Barbuda	Both	40-44 years	0.32	0.23	0.42	Antigua and Barbuda	Male	40-44 years	0.27	0.19	0.36	Antigua and Barbuda	Female	40-44 years	0.36	0.26	0.49
Antigua and Barbuda	Both	45-49 years	0.66	0.51	0.83	Antigua and Barbuda	Male	45-49 years	0.55	0.42	0.70	Antigua and Barbuda	Female	45-49 years	0.75	0.58	0.96
Antigua and Barbuda	Both	50-54 years	1.17	0.88	1.48	Antigua and Barbuda	Male	50-54 years	0.97	0.73	1.23	Antigua and Barbuda	Female	50-54 years	1.34	1.02	1.72
Antigua and Barbuda	Both	55-59 years	1.89	1.45	2.30	Antigua and Barbuda	Male	55-59 years	1.57	1.21	1.92	Antigua and Barbuda	Female	55-59 years	2.18	1.69	2.68
Antigua and Barbuda	Both	60-64 years	2.85	2.21	3.51	Antigua and Barbuda	Male	60-64 years	2.36	1.83	2.96	Antigua and Barbuda	Female	60-64 years	3.32	2.59	4.14
Antigua and Barbuda	Both	65-69 years	4.43	3.55	5.40	Antigua and Barbuda	Male	65-69 years	3.65	2.91	4.51	Antigua and Barbuda	Female	65-69 years	5.18	4.15	6.34
Antigua and Barbuda	Both	70-74 years	6.50	4.99	8.11	Antigua and Barbuda	Male	70-74 years	5.35	4.11	6.72	Antigua and Barbuda	Female	70-74 years	7.58	5.81	9.54
Antigua and Barbuda	Both	75-79 years	8.67	6.86	10.56	Antigua and Barbuda	Male	75-79 years	7.15	5.70	8.70	Antigua and Barbuda	Female	75-79 years	10.05	7.94	12.31
Antigua and Barbuda	Both	All ages	1.11	0.95	1.27	Antigua and Barbuda	Male	All ages	0.88	0.75	1.01	Antigua and Barbuda	Female	All ages	1.32	1.13	1.51
Antigua and Barbuda	Both	80-84	10.88	8.87	13.25	Antigua and Barbuda	Male	80-84	8.93	7.20	10.98	Antigua and Barbuda	Female	80-84	12.38	10.06	15.13
Antigua and Barbuda	Both	85-89	12.85	10.56	15.46	Antigua and Barbuda	Male	85-89	10.53	8.59	12.85	Antigua and Barbuda	Female	85-89	14.26	11.66	17.24
Antigua and Barbuda	Both	90-94	14.45	11.92	17.37	Antigua and Barbuda	Male	90-94	12.00	9.81	14.47	Antigua and Barbuda	Female	90-94	15.73	12.97	18.89
Argentin<br>a	Both	40-44 years	0.75	0.56	1.00	Argentin<br>a	Male	40-44 years	0.40	0.29	0.54	Argentin<br>a	Female	45-49 years	1.82	1.42	2.26
Argentin<br>a	Both	45-49 years	1.35	1.06	1.68	Argentin<br>a	Male	45-49 years	0.85	0.65	1.09	Argentin<br>a	Female	50-54 years	2.60	2.01	3.27
Argentin<br>a	Both	50-54 years	2.09	1.63	2.61	Argentin<br>a	Male	50-54 years	1.53	1.16	1.99	Argentin<br>a	Female	55-59 years	3.59	2.85	4.38
Argentin<br>a	Both	55-59 years	3.10	2.48	3.73	Argentin<br>a	Male	55-59 years	2.57	2.04	3.15	Argentin<br>a	Female	60-64 years	5.03	4.03	6.14

Argentin a	Both	60-64 years	4.52	3.64	5.53	Argentin a	Male	60-64 years	3.94	3.09	4.97	Argentin a	Female	65-69 years	7.90	6.39	9.56
Argentin a	Both	65-69 years	7.02	5.69	8.51	Argentin a	Male	65-69 years	5.98	4.81	7.36	Argentin a	Female	70-74 years	11.80	9.25	14.84
Argentin a	Both	70-74 years	10.34	8.09	12.98	Argentin a	Male	70-74 years	8.49	6.52	10.71	Argentin a	Female	75-79 years	15.64	12.58	19.30
Argentin a	Both	75-79 years	13.65	10.97	16.78	Argentin a	Male	75-79 years	10.83	8.53	13.40	Argentin a	Female	All ages	2.54	2.16	2.91
Argentin a	Both	All ages	2.00	1.71	2.28	Argentin a	Male	All ages	1.41	1.21	1.64	Argentin a	Female	80-84	19.14	15.55	23.39
Argentin a	Both	80-84	16.78	13.67	20.41	Argentin a	Male	80-84	12.88	10.33	15.82	Argentin a	Female	85-89	21.68	17.94	26.25
Argentin a	Both	85-89	19.30	15.93	23.27	Argentin a	Male	85-89	14.43	11.79	17.51	Argentin a	Female	90-94	23.22	19.32	27.78
Argentin a	Both	90-94	20.99	17.55	24.96	Argentin a	Male	90-94	15.49	12.76	18.57	Argentin a	Female	40-44 years	1.09	0.81	1.44
Armenia	Both	40-44 years	0.49	0.35	0.64	Armenia	Male	40-44 years	0.43	0.31	0.56	Armenia	Female	40-44 years	0.54	0.39	0.72
Armenia	Both	45-49 years	1.04	0.80	1.33	Armenia	Male	45-49 years	0.88	0.68	1.10	Armenia	Female	45-49 years	1.18	0.90	1.53
Armenia	Both	50-54 years	1.86	1.39	2.42	Armenia	Male	50-54 years	1.54	1.17	1.96	Armenia	Female	50-54 years	2.13	1.59	2.82
Armenia	Both	55-59 years	2.95	2.30	3.63	Armenia	Male	55-59 years	2.42	1.89	2.95	Armenia	Female	55-59 years	3.39	2.61	4.20
Armenia	Both	60-64 years	4.35	3.39	5.40	Armenia	Male	60-64 years	3.55	2.77	4.40	Armenia	Female	60-64 years	5.00	3.85	6.27
Armenia	Both	65-69 years	6.54	5.26	8.01	Armenia	Male	65-69 years	5.28	4.28	6.45	Armenia	Female	65-69 years	7.52	5.98	9.21
Armenia	Both	70-74 years	9.36	7.19	11.78	Armenia	Male	70-74 years	7.48	5.78	9.45	Armenia	Female	70-74 years	10.68	8.14	13.62
Armenia	Both	75-79 years	12.18	9.71	15.07	Armenia	Male	75-79 years	9.71	7.78	11.86	Armenia	Female	75-79 years	13.72	10.88	17.14
Armenia	Both	All ages	2.00	1.73	2.28	Armenia	Male	All ages	1.43	1.24	1.64	Armenia	Female	All ages	2.52	2.15	2.89
Armenia	Both	80-84	14.74	11.92	18.04	Armenia	Male	80-84	11.83	9.64	14.48	Armenia	Female	80-84	16.41	13.24	20.24
Armenia	Both	85-89	16.59	13.61	19.95	Armenia	Male	85-89	13.62	11.19	16.53	Armenia	Female	85-89	18.34	14.98	22.21
Armenia	Both	90-94	18.00	15.00	21.50	Armenia	Male	90-94	15.15	12.47	18.30	Armenia	Female	90-94	19.58	16.30	23.34
Australia	Both	80-84	13.41	11.01	16.22	Australia	Male	80-84	9.58	7.77	11.69	Australia	Female	40-44 years	0.89	0.66	1.18
Australia	Both	85-89	15.82	13.10	18.92	Australia	Male	85-89	11.03	8.98	13.33	Australia	Female	45-49 years	1.51	1.18	1.88
Australia	Both	90-94	17.95	14.97	21.31	Australia	Male	90-94	12.28	10.08	14.89	Australia	Female	50-54 years	2.19	1.69	2.78
Australia	Both	40-44 years	0.60	0.45	0.79	Australia	Male	40-44 years	0.30	0.22	0.41	Australia	Female	55-59 years	3.09	2.47	3.80

Australia	Both	45-49 years	1.08	0.85	1.35	Australia	Male	45-49 years	0.64	0.49	0.81	Australia	Female	60-64 years	4.40	3.52	5.45
Australia	Both	50-54 years	1.68	1.31	2.12	Australia	Male	50-54 years	1.14	0.87	1.46	Australia	Female	65-69 years	6.82	5.54	8.31
Australia	Both	55-59 years	2.52	2.01	3.07	Australia	Male	55-59 years	1.91	1.50	2.35	Australia	Female	70-74 years	10.07	7.89	12.61
Australia	Both	60-64 years	3.70	2.98	4.55	Australia	Male	60-64 years	2.94	2.31	3.70	Australia	Female	75-79 years	13.38	10.80	16.01
Australia	Both	65-69 years	5.66	4.58	6.87	Australia	Male	65-69 years	4.42	3.52	5.43	Australia	Female	All ages	2.96	2.56	3.36
Australia	Both	70-74 years	8.19	6.37	10.19	Australia	Male	70-74 years	6.20	4.68	7.81	Australia	Female	80-84	16.54	13.60	19.83
Australia	Both	75-79 years	10.80	8.65	13.01	Australia	Male	75-79 years	7.94	6.24	9.79	Australia	Female	85-89	19.13	15.86	22.91
Australia	Both	All ages	2.30	1.98	2.61	Australia	Male	All ages	1.60	1.36	1.85	Australia	Female	90-94	21.04	17.48	24.79
Austria	Both	80-84	20.39	16.73	24.60	Austria	Male	40-44 years	0.54	0.39	0.73	Austria	Female	80-84	22.77	18.76	27.66
Austria	Both	85-89	23.36	19.46	27.81	Austria	Male	45-49 years	1.13	0.88	1.43	Austria	Female	85-89	25.78	21.56	30.79
Austria	Both	90-94	25.50	21.51	29.98	Austria	Male	50-54 years	2.02	1.54	2.59	Austria	Female	90-94	27.72	23.36	32.66
Austria	Both	40-44 years	1.01	0.76	1.33	Austria	Male	55-59 years	3.38	2.63	4.19	Austria	Female	40-44 years	1.47	1.10	1.91
Austria	Both	45-49 years	1.76	1.40	2.16	Austria	Male	60-64 years	5.19	4.05	6.55	Austria	Female	45-49 years	2.37	1.87	2.94
Austria	Both	50-54 years	2.64	2.07	3.28	Austria	Male	65-69 years	7.88	6.32	9.67	Austria	Female	50-54 years	3.26	2.56	4.06
Austria	Both	55-59 years	3.88	3.08	4.69	Austria	Male	70-74 years	11.16	8.57	13.94	Austria	Female	55-59 years	4.38	3.49	5.30
Austria	Both	60-64 years	5.64	4.49	6.92	Austria	Male	75-79 years	14.24	11.36	17.39	Austria	Female	60-64 years	6.07	4.86	7.42
Austria	Both	65-69 years	8.75	7.07	10.56	Austria	Male	All ages	3.12	2.67	3.60	Austria	Female	65-69 years	9.53	7.71	11.45
Austria	Both	70-74 years	12.82	10.03	15.93	Austria	Male	80-84	16.96	13.63	20.47	Austria	Female	70-74 years	14.23	11.07	17.91
Austria	Both	75-79 years	16.76	13.41	20.50	Austria	Male	85-89	18.95	15.56	22.66	Austria	Female	75-79 years	18.74	14.89	23.02
Austria	Both	All ages	4.11	3.55	4.70	Austria	Male	90-94	20.21	16.90	23.80	Austria	Female	All ages	5.03	4.32	5.78
Azerbaijan	Both	40-44 years	0.44	0.32	0.59	Azerbaijan	Male	40-44 years	0.39	0.28	0.52	Azerbaijan	Female	40-44 years	0.49	0.36	0.67
Azerbaijan	Both	45-49 years	0.94	0.72	1.18	Azerbaijan	Male	45-49 years	0.80	0.62	1.02	Azerbaijan	Female	45-49 years	1.06	0.81	1.35
Azerbaijan	Both	50-54 years	1.66	1.27	2.13	Azerbaijan	Male	50-54 years	1.39	1.07	1.79	Azerbaijan	Female	50-54 years	1.91	1.42	2.47
Azerbaijan	Both	55-59 years	2.63	2.07	3.23	Azerbaijan	Male	55-59 years	2.19	1.73	2.67	Azerbaijan	Female	55-59 years	3.02	2.34	3.74

Azerbaijan	Both	60-64 years	3.87	3.06	4.80	Azerbaijan	Male	60-64 years	3.21	2.54	3.99	Azerbaijan	Female	60-64 years	4.44	3.46	5.59
Azerbaijan	Both	65-69 years	5.82	4.73	7.16	Azerbaijan	Male	65-69 years	4.75	3.81	5.80	Azerbaijan	Female	65-69 years	6.68	5.44	8.29
Azerbaijan	Both	70-74 years	8.36	6.52	10.51	Azerbaijan	Male	70-74 years	6.73	5.18	8.38	Azerbaijan	Female	70-74 years	9.53	7.44	12.12
Azerbaijan	Both	75-79 years	10.93	8.73	13.55	Azerbaijan	Male	75-79 years	8.77	6.99	10.67	Azerbaijan	Female	75-79 years	12.36	9.99	15.43
Azerbaijan	Both	All ages	1.09	0.94	1.26	Azerbaijan	Male	All ages	0.81	0.69	0.93	Azerbaijan	Female	All ages	1.36	1.17	1.58
Azerbaijan	Both	80-84	13.38	10.83	16.44	Azerbaijan	Male	80-84	10.76	8.70	13.23	Azerbaijan	Female	80-84	14.97	12.13	18.42
Azerbaijan	Both	85-89	15.31	12.64	18.44	Azerbaijan	Male	85-89	12.53	10.29	15.09	Azerbaijan	Female	85-89	16.99	14.01	20.53
Azerbaijan	Both	90-94	16.70	13.79	20.04	Azerbaijan	Male	90-94	14.15	11.62	17.11	Azerbaijan	Female	90-94	18.46	15.26	22.13
Bahamas	Both	40-44 years	0.31	0.22	0.41	Bahamas	Male	40-44 years	0.26	0.19	0.35	Bahamas	Female	40-44 years	0.35	0.25	0.48
Bahamas	Both	45-49 years	0.64	0.49	0.81	Bahamas	Male	45-49 years	0.54	0.41	0.68	Bahamas	Female	45-49 years	0.73	0.57	0.93
Bahamas	Both	50-54 years	1.13	0.86	1.45	Bahamas	Male	50-54 years	0.94	0.72	1.19	Bahamas	Female	50-54 years	1.30	0.98	1.67
Bahamas	Both	55-59 years	1.83	1.44	2.23	Bahamas	Male	55-59 years	1.51	1.19	1.85	Bahamas	Female	55-59 years	2.11	1.64	2.59
Bahamas	Both	60-64 years	2.76	2.19	3.41	Bahamas	Male	60-64 years	2.26	1.80	2.79	Bahamas	Female	60-64 years	3.19	2.51	3.97
Bahamas	Both	65-69 years	4.30	3.49	5.25	Bahamas	Male	65-69 years	3.50	2.82	4.33	Bahamas	Female	65-69 years	4.99	3.99	6.09
Bahamas	Both	70-74 years	6.38	4.90	7.95	Bahamas	Male	70-74 years	5.16	3.96	6.52	Bahamas	Female	70-74 years	7.34	5.62	9.25
Bahamas	Both	75-79 years	8.55	6.80	10.47	Bahamas	Male	75-79 years	6.93	5.45	8.60	Bahamas	Female	75-79 years	9.75	7.71	11.88
Bahamas	Both	All ages	0.96	0.83	1.10	Bahamas	Male	All ages	0.73	0.63	0.84	Bahamas	Female	All ages	1.17	1.01	1.33
Bahamas	Both	80-84	10.65	8.57	13.06	Bahamas	Male	80-84	8.71	6.86	10.85	Bahamas	Female	80-84	12.05	9.65	14.71
Bahamas	Both	85-89	12.51	10.24	15.11	Bahamas	Male	85-89	10.32	8.38	12.62	Bahamas	Female	85-89	13.94	11.44	16.90
Bahamas	Both	90-94	14.25	11.85	17.03	Bahamas	Male	90-94	11.81	9.65	14.19	Bahamas	Female	90-94	15.46	12.82	18.51
Bahrain	Both	40-44 years	0.40	0.29	0.54	Bahrain	Male	40-44 years	0.37	0.27	0.51	Bahrain	Female	40-44 years	0.47	0.34	0.64
Bahrain	Both	45-49 years	0.86	0.67	1.07	Bahrain	Male	45-49 years	0.78	0.61	0.98	Bahrain	Female	45-49 years	1.01	0.77	1.26
Bahrain	Both	50-54 years	1.53	1.17	1.95	Bahrain	Male	50-54 years	1.39	1.05	1.76	Bahrain	Female	50-54 years	1.80	1.37	2.29
Bahrain	Both	55-59 years	2.44	1.90	2.97	Bahrain	Male	55-59 years	2.21	1.72	2.71	Bahrain	Female	55-59 years	2.87	2.23	3.48

Bahrain	Both	60-64 years	3.59	2.81	4.42	Bahrain	Male	60-64 years	3.24	2.53	4.04	Bahrain	Female	60-64 years	4.24	3.28	5.18
Bahrain	Both	65-69 years	5.35	4.34	6.52	Bahrain	Male	65-69 years	4.81	3.86	5.88	Bahrain	Female	65-69 years	6.35	5.09	7.73
Bahrain	Both	70-74 years	7.80	6.09	9.84	Bahrain	Male	70-74 years	6.81	5.30	8.55	Bahrain	Female	70-74 years	9.05	7.04	11.41
Bahrain	Both	75-79 years	10.44	8.31	12.70	Bahrain	Male	75-79 years	8.96	7.13	10.92	Bahrain	Female	75-79 years	11.85	9.40	14.51
Bahrain	Both	All ages	0.78	0.66	0.90	Bahrain	Male	All ages	0.71	0.60	0.83	Bahrain	Female	All ages	0.89	0.76	1.02
Bahrain	Both	80-84	13.01	10.49	15.73	Bahrain	Male	80-84	11.16	8.98	13.59	Bahrain	Female	80-84	14.58	11.69	17.70
Bahrain	Both	85-89	15.12	12.34	18.02	Bahrain	Male	85-89	13.19	10.80	15.87	Bahrain	Female	85-89	16.84	13.77	20.23
Bahrain	Both	90-94	17.03	14.20	20.21	Bahrain	Male	90-94	15.05	12.48	18.14	Bahrain	Female	90-94	18.65	15.54	22.04
Bangladesh	Both	40-44 years	0.31	0.22	0.41	Bangladesh	Male	40-44 years	0.27	0.19	0.37	Bangladesh	Female	40-44 years	0.34	0.24	0.45
Bangladesh	Both	45-49 years	0.64	0.49	0.83	Bangladesh	Male	45-49 years	0.56	0.43	0.72	Bangladesh	Female	45-49 years	0.72	0.54	0.92
Bangladesh	Both	50-54 years	1.13	0.86	1.45	Bangladesh	Male	50-54 years	0.98	0.74	1.26	Bangladesh	Female	50-54 years	1.27	0.96	1.67
Bangladesh	Both	55-59 years	1.76	1.38	2.15	Bangladesh	Male	55-59 years	1.53	1.20	1.89	Bangladesh	Female	55-59 years	2.00	1.55	2.44
Bangladesh	Both	60-64 years	2.55	2.00	3.15	Bangladesh	Male	60-64 years	2.22	1.74	2.80	Bangladesh	Female	60-64 years	2.91	2.28	3.58
Bangladesh	Both	65-69 years	3.76	3.02	4.64	Bangladesh	Male	65-69 years	3.25	2.60	4.03	Bangladesh	Female	65-69 years	4.32	3.46	5.29
Bangladesh	Both	70-74 years	5.30	4.12	6.62	Bangladesh	Male	70-74 years	4.58	3.51	5.76	Bangladesh	Female	70-74 years	6.14	4.77	7.84
Bangladesh	Both	75-79 years	6.94	5.55	8.42	Bangladesh	Male	75-79 years	6.01	4.75	7.34	Bangladesh	Female	75-79 years	8.03	6.47	9.81
Bangladesh	Both	All ages	0.66	0.57	0.76	Bangladesh	Male	All ages	0.61	0.52	0.71	Bangladesh	Female	All ages	0.71	0.60	0.81
Bangladesh	Both	80-84	8.58	6.89	10.58	Bangladesh	Male	80-84	7.46	5.96	9.18	Bangladesh	Female	80-84	9.83	7.90	12.14
Bangladesh	Both	85-89	9.98	8.12	12.13	Bangladesh	Male	85-89	8.80	7.18	10.74	Bangladesh	Female	85-89	11.35	9.17	13.90
Bangladesh	Both	90-94	11.24	9.20	13.57	Bangladesh	Male	90-94	10.07	8.21	12.26	Bangladesh	Female	90-94	12.61	10.32	15.17
Barbados	Both	40-44 years	0.32	0.23	0.43	Barbados	Male	80-84	8.91	7.10	11.11	Barbados	Female	40-44 years	0.36	0.26	0.49
Barbados	Both	45-49 years	0.66	0.51	0.84	Barbados	Male	85-89	10.45	8.48	12.73	Barbados	Female	45-49 years	0.76	0.58	0.96
Barbados	Both	50-54 years	1.18	0.89	1.51	Barbados	Male	90-94	11.83	9.70	14.35	Barbados	Female	50-54 years	1.35	1.01	1.76
Barbados	Both	55-59 years	1.91	1.49	2.34	Barbados	Male	40-44 years	0.27	0.19	0.36	Barbados	Female	55-59 years	2.20	1.72	2.71

Barbados	Both	60-64 years	2.89	2.27	3.59	Barbados	Male	45-49 years	0.56	0.43	0.71	Barbados	Female	60-64 years	3.35	2.62	4.18
Barbados	Both	65-69 years	4.50	3.63	5.50	Barbados	Male	50-54 years	0.98	0.75	1.26	Barbados	Female	65-69 years	5.22	4.17	6.36
Barbados	Both	70-74 years	6.63	5.10	8.30	Barbados	Male	55-59 years	1.57	1.22	1.94	Barbados	Female	70-74 years	7.65	5.86	9.65
Barbados	Both	75-79 years	8.83	7.04	10.91	Barbados	Male	60-64 years	2.37	1.85	2.95	Barbados	Female	75-79 years	10.10	8.05	12.47
Barbados	Both	All ages	1.68	1.45	1.94	Barbados	Male	65-69 years	3.68	2.95	4.57	Barbados	Female	All ages	2.05	1.76	2.35
Barbados	Both	80-84	10.90	8.85	13.33	Barbados	Male	70-74 years	5.39	4.12	6.82	Barbados	Female	80-84	12.38	10.03	15.09
Barbados	Both	85-89	12.67	10.39	15.24	Barbados	Male	75-79 years	7.18	5.71	8.83	Barbados	Female	85-89	14.17	11.61	17.11
Barbados	Both	90-94	14.10	11.59	16.93	Barbados	Male	All ages	1.29	1.10	1.49	Barbados	Female	90-94	15.53	12.73	18.70
Belarus	Both	40-44 years	0.49	0.36	0.65	Belarus	Male	40-44 years	0.39	0.29	0.52	Belarus	Female	40-44 years	0.59	0.43	0.80
Belarus	Both	45-49 years	1.05	0.82	1.32	Belarus	Male	45-49 years	0.80	0.63	1.00	Belarus	Female	45-49 years	1.28	0.98	1.61
Belarus	Both	50-54 years	1.87	1.43	2.37	Belarus	Male	50-54 years	1.39	1.07	1.76	Belarus	Female	50-54 years	2.29	1.73	2.93
Belarus	Both	55-59 years	2.98	2.33	3.64	Belarus	Male	55-59 years	2.18	1.73	2.65	Belarus	Female	55-59 years	3.63	2.80	4.47
Belarus	Both	60-64 years	4.42	3.47	5.45	Belarus	Male	60-64 years	3.18	2.52	3.91	Belarus	Female	60-64 years	5.35	4.14	6.67
Belarus	Both	65-69 years	6.77	5.49	8.25	Belarus	Male	65-69 years	4.69	3.79	5.71	Belarus	Female	65-69 years	8.13	6.53	9.97
Belarus	Both	70-74 years	9.84	7.64	12.33	Belarus	Male	70-74 years	6.62	5.19	8.23	Belarus	Female	70-74 years	11.65	8.96	14.68
Belarus	Both	75-79 years	13.09	10.48	16.22	Belarus	Male	75-79 years	8.64	6.85	10.48	Belarus	Female	75-79 years	15.01	11.91	18.68
Belarus	Both	All ages	2.60	2.24	2.97	Belarus	Male	All ages	1.40	1.21	1.61	Belarus	Female	All ages	3.61	3.09	4.16
Belarus	Both	80-84	16.02	13.00	19.48	Belarus	Male	80-84	10.63	8.63	13.09	Belarus	Female	80-84	17.91	14.45	21.76
Belarus	Both	85-89	18.08	14.83	21.75	Belarus	Male	85-89	12.36	10.12	14.95	Belarus	Female	85-89	19.84	16.14	23.96
Belarus	Both	90-94	19.50	16.19	23.14	Belarus	Male	90-94	13.89	11.39	16.76	Belarus	Female	90-94	20.93	17.35	24.85
Belgium	Both	45-49 years	1.95	1.55	2.41	Belgium	Male	45-49 years	1.25	0.97	1.56	Belgium	Female	40-44 years	1.66	1.25	2.19
Belgium	Both	50-54 years	2.92	2.28	3.65	Belgium	Male	50-54 years	2.23	1.72	2.84	Belgium	Female	45-49 years	2.66	2.08	3.32
Belgium	Both	55-59 years	4.26	3.40	5.17	Belgium	Male	55-59 years	3.73	2.93	4.55	Belgium	Female	50-54 years	3.61	2.82	4.56
Belgium	Both	60-64 years	6.19	4.91	7.56	Belgium	Male	60-64 years	5.74	4.51	7.18	Belgium	Female	55-59 years	4.80	3.82	5.88

Belgium	Both	65-69 years	9.67	7.84	11.73	Belgium	Male	65-69 years	8.80	7.04	10.83	Belgium	Female	60-64 years	6.63	5.28	8.13
Belgium	Both	70-74 years	14.25	10.99	18.02	Belgium	Male	70-74 years	12.53	9.80	15.87	Belgium	Female	65-69 years	10.50	8.51	12.66
Belgium	Both	75-79 years	18.56	14.88	22.84	Belgium	Male	75-79 years	15.90	12.69	19.73	Belgium	Female	70-74 years	15.77	12.24	19.97
Belgium	Both	All ages	4.53	3.90	5.17	Belgium	Male	All ages	3.52	3.01	4.07	Belgium	Female	75-79 years	20.69	16.56	25.35
Belgium	Both	80-84	22.38	18.37	27.08	Belgium	Male	80-84	18.74	15.27	22.69	Belgium	Female	All ages	5.46	4.70	6.24
Belgium	Both	85-89	25.36	21.15	30.61	Belgium	Male	85-89	20.74	17.18	24.89	Belgium	Female	80-84	24.92	20.37	30.00
Belgium	Both	90-94	27.41	22.98	32.26	Belgium	Male	90-94	21.92	18.28	25.91	Belgium	Female	85-89	27.92	23.23	33.82
Belgium	Both	40-44 years	1.13	0.85	1.49	Belgium	Male	40-44 years	0.60	0.44	0.80	Belgium	Female	90-94	29.69	24.92	35.21
Belize	Both	40-44 years	0.32	0.23	0.44	Belize	Male	40-44 years	0.27	0.19	0.37	Belize	Female	40-44 years	0.37	0.26	0.51
Belize	Both	45-49 years	0.67	0.52	0.85	Belize	Male	45-49 years	0.56	0.43	0.72	Belize	Female	45-49 years	0.77	0.60	0.98
Belize	Both	50-54 years	1.18	0.90	1.50	Belize	Male	50-54 years	0.99	0.75	1.27	Belize	Female	50-54 years	1.37	1.03	1.76
Belize	Both	55-59 years	1.90	1.48	2.33	Belize	Male	55-59 years	1.59	1.24	1.95	Belize	Female	55-59 years	2.22	1.72	2.76
Belize	Both	60-64 years	2.85	2.24	3.59	Belize	Male	60-64 years	2.38	1.86	2.98	Belize	Female	60-64 years	3.34	2.61	4.21
Belize	Both	65-69 years	4.43	3.55	5.43	Belize	Male	65-69 years	3.70	2.93	4.54	Belize	Female	65-69 years	5.22	4.16	6.44
Belize	Both	70-74 years	6.51	5.04	8.21	Belize	Male	70-74 years	5.45	4.20	6.92	Belize	Female	70-74 years	7.68	5.90	9.85
Belize	Both	75-79 years	8.60	6.90	10.72	Belize	Male	75-79 years	7.25	5.85	8.88	Belize	Female	75-79 years	10.14	8.10	12.55
Belize	Both	All ages	0.63	0.55	0.72	Belize	Male	All ages	0.54	0.46	0.63	Belize	Female	All ages	0.72	0.62	0.83
Belize	Both	80-84	10.63	8.71	13.06	Belize	Male	80-84	8.97	7.33	11.09	Belize	Female	80-84	12.38	10.08	15.09
Belize	Both	85-89	12.38	10.16	14.95	Belize	Male	85-89	10.42	8.54	12.69	Belize	Female	85-89	14.06	11.53	17.08
Belize	Both	90-94	13.68	11.30	16.32	Belize	Male	90-94	11.66	9.52	13.98	Belize	Female	90-94	15.24	12.53	18.23
Benin	Both	40-44 years	0.31	0.22	0.41	Benin	Male	40-44 years	0.28	0.20	0.39	Benin	Female	40-44 years	0.33	0.23	0.44
Benin	Both	45-49 years	0.67	0.52	0.86	Benin	Male	45-49 years	0.62	0.47	0.80	Benin	Female	45-49 years	0.72	0.55	0.93
Benin	Both	50-54 years	1.23	0.93	1.59	Benin	Male	50-54 years	1.13	0.85	1.46	Benin	Female	50-54 years	1.33	0.98	1.70
Benin	Both	55-59 years	1.98	1.53	2.44	Benin	Male	55-59 years	1.80	1.40	2.23	Benin	Female	55-59 years	2.16	1.66	2.66

Benin	Both	60-64 years	2.94	2.28	3.71	Benin	Male	60-64 years	2.65	2.05	3.36	Benin	Female	60-64 years	3.22	2.50	4.07
Benin	Both	65-69 years	4.44	3.53	5.48	Benin	Male	65-69 years	3.93	3.12	4.88	Benin	Female	65-69 years	4.90	3.91	6.08
Benin	Both	70-74 years	6.38	4.94	8.09	Benin	Male	70-74 years	5.59	4.30	7.09	Benin	Female	70-74 years	7.07	5.44	8.97
Benin	Both	75-79 years	8.45	6.80	10.37	Benin	Male	75-79 years	7.35	5.84	9.08	Benin	Female	75-79 years	9.30	7.49	11.43
Benin	Both	All ages	0.34	0.29	0.39	Benin	Male	All ages	0.29	0.25	0.33	Benin	Female	All ages	0.39	0.34	0.45
Benin	Both	80-84	10.47	8.41	12.84	Benin	Male	80-84	9.10	7.31	11.24	Benin	Female	80-84	11.43	9.24	14.02
Benin	Both	85-89	12.24	9.96	14.71	Benin	Male	85-89	10.66	8.62	12.87	Benin	Female	85-89	13.16	10.74	15.89
Benin	Both	90-94	13.73	11.30	16.40	Benin	Male	90-94	12.04	9.92	14.36	Benin	Female	90-94	14.53	11.93	17.41
Bermuda	Both	40-44 years	0.30	0.21	0.41	Bermuda	Male	40-44 years	0.26	0.18	0.36	Bermuda	Female	40-44 years	0.34	0.24	0.47
Bermuda	Both	45-49 years	0.62	0.48	0.79	Bermuda	Male	45-49 years	0.53	0.40	0.67	Bermuda	Female	45-49 years	0.72	0.55	0.92
Bermuda	Both	50-54 years	1.10	0.84	1.39	Bermuda	Male	50-54 years	0.92	0.70	1.16	Bermuda	Female	50-54 years	1.28	0.97	1.64
Bermuda	Both	55-59 years	1.80	1.40	2.18	Bermuda	Male	55-59 years	1.48	1.16	1.79	Bermuda	Female	55-59 years	2.09	1.62	2.55
Bermuda	Both	60-64 years	2.76	2.18	3.43	Bermuda	Male	60-64 years	2.25	1.76	2.81	Bermuda	Female	60-64 years	3.22	2.52	3.99
Bermuda	Both	65-69 years	4.30	3.49	5.22	Bermuda	Male	65-69 years	3.47	2.79	4.20	Bermuda	Female	65-69 years	5.01	4.06	6.08
Bermuda	Both	70-74 years	6.32	4.89	7.91	Bermuda	Male	70-74 years	5.08	3.95	6.32	Bermuda	Female	70-74 years	7.33	5.67	9.17
Bermuda	Both	75-79 years	8.50	6.86	10.38	Bermuda	Male	75-79 years	6.82	5.48	8.39	Bermuda	Female	75-79 years	9.74	7.87	11.91
Bermuda	Both	All ages	2.02	1.74	2.31	Bermuda	Male	All ages	1.48	1.26	1.70	Bermuda	Female	All ages	2.51	2.16	2.89
Bermuda	Both	80-84	10.64	8.65	12.97	Bermuda	Male	80-84	8.61	6.96	10.53	Bermuda	Female	80-84	12.08	9.79	14.71
Bermuda	Both	85-89	12.69	10.48	15.26	Bermuda	Male	85-89	10.33	8.49	12.51	Bermuda	Female	85-89	14.07	11.52	16.96
Bermuda	Both	90-94	14.70	12.25	17.41	Bermuda	Male	90-94	12.01	9.87	14.42	Bermuda	Female	90-94	15.75	13.14	18.71
Bhutan	Both	40-44 years	0.31	0.22	0.42	Bhutan	Male	40-44 years	0.28	0.20	0.39	Bhutan	Female	90-94	13.27	10.89	16.08
Bhutan	Both	45-49 years	0.65	0.50	0.82	Bhutan	Male	45-49 years	0.58	0.44	0.74	Bhutan	Female	40-44 years	0.35	0.25	0.47
Bhutan	Both	50-54 years	1.15	0.86	1.49	Bhutan	Male	50-54 years	1.00	0.76	1.29	Bhutan	Female	45-49 years	0.74	0.56	0.93
Bhutan	Both	55-59 years	1.81	1.42	2.21	Bhutan	Male	55-59 years	1.58	1.24	1.92	Bhutan	Female	50-54 years	1.31	0.98	1.69

Bhutan	Both	60-64 years	2.65	2.09	3.26	Bhutan	Male	60-64 years	2.30	1.81	2.83	Bhutan	Female	55-59 years	2.07	1.61	2.54
Bhutan	Both	65-69 years	3.92	3.18	4.80	Bhutan	Male	65-69 years	3.39	2.73	4.15	Bhutan	Female	60-64 years	3.02	2.37	3.73
Bhutan	Both	70-74 years	5.55	4.28	6.95	Bhutan	Male	70-74 years	4.78	3.69	6.01	Bhutan	Female	65-69 years	4.47	3.59	5.48
Bhutan	Both	75-79 years	7.27	5.78	8.90	Bhutan	Male	75-79 years	6.27	5.07	7.69	Bhutan	Female	70-74 years	6.34	4.90	7.98
Bhutan	Both	All ages	0.62	0.53	0.71	Bhutan	Male	All ages	0.53	0.46	0.61	Bhutan	Female	75-79 years	8.30	6.56	10.10
Bhutan	Both	80-84	8.98	7.24	10.97	Bhutan	Male	80-84	7.79	6.27	9.58	Bhutan	Female	All ages	0.71	0.60	0.81
Bhutan	Both	85-89	10.53	8.65	12.62	Bhutan	Male	85-89	9.23	7.53	11.24	Bhutan	Female	80-84	10.21	8.18	12.53
Bhutan	Both	90-94	11.98	9.85	14.47	Bhutan	Male	90-94	10.62	8.73	12.85	Bhutan	Female	85-89	11.86	9.69	14.26
Bolivia (Plurinational State of)	Both	70-74 years	5.81	4.42	7.33	Bolivia (Plurinational State of)	Male	70-74 years	4.90	3.74	6.11	Bolivia (Plurinational State of)	Female	70-74 years	6.61	4.99	8.60
Bolivia (Plurinational State of)	Both	75-79 years	7.72	6.17	9.48	Bolivia (Plurinational State of)	Male	75-79 years	6.52	5.21	7.98	Bolivia (Plurinational State of)	Female	75-79 years	8.76	6.98	10.93
Bolivia (Plurinational State of)	Both	All ages	0.63	0.54	0.71	Bolivia (Plurinational State of)	Male	All ages	0.51	0.44	0.59	Bolivia (Plurinational State of)	Female	All ages	0.74	0.63	0.85
Bolivia (Plurinational State of)	Both	80-84	9.55	7.76	11.76	Bolivia (Plurinational State of)	Male	80-84	8.10	6.53	10.01	Bolivia (Plurinational State of)	Female	80-84	10.78	8.70	13.35
Bolivia (Plurinational State of)	Both	85-89	11.10	9.09	13.37	Bolivia (Plurinational State of)	Male	85-89	9.49	7.73	11.43	Bolivia (Plurinational State of)	Female	85-89	12.38	10.09	14.97
Bolivia (Plurinational State of)	Both	90-94	12.40	10.23	14.92	Bolivia (Plurinational State of)	Male	90-94	10.76	8.83	12.97	Bolivia (Plurinational State of)	Female	90-94	13.62	11.19	16.46
Bolivia (Plurinational State of)	Both	40-44 years	0.29	0.21	0.39	Bolivia (Plurinational State of)	Male	40-44 years	0.25	0.18	0.34	Bolivia (Plurinational State of)	Female	40-44 years	0.32	0.23	0.44
Bolivia (Plurinational State of)	Both	45-49 years	0.60	0.46	0.77	Bolivia (Plurinational State of)	Male	45-49 years	0.52	0.40	0.67	Bolivia (Plurinational State of)	Female	45-49 years	0.68	0.52	0.87
Bolivia (Plurinational State of)	Both	50-54 years	1.06	0.80	1.38	Bolivia (Plurinational State of)	Male	50-54 years	0.91	0.68	1.17	Bolivia (Plurinational State of)	Female	50-54 years	1.20	0.90	1.57

Bolivia (Plurinational State of)	Both	55-59 years	1.69	1.31	2.08	Bolivia (Plurinational State of)	Male	55-59 years	1.45	1.11	1.77	Bolivia (Plurinational State of)	Female	55-59 years	1.93	1.49	2.40
Bolivia (Plurinational State of)	Both	60-64 years	2.54	1.99	3.15	Bolivia (Plurinational State of)	Male	60-64 years	2.15	1.69	2.67	Bolivia (Plurinational State of)	Female	60-64 years	2.90	2.26	3.63
Bolivia (Plurinational State of)	Both	65-69 years	3.95	3.14	4.85	Bolivia (Plurinational State of)	Male	65-69 years	3.33	2.65	4.12	Bolivia (Plurinational State of)	Female	65-69 years	4.50	3.58	5.54
Bosnia and Herzegovina	Both	40-44 years	0.53	0.38	0.71	Bosnia and Herzegovina	Male	40-44 years	0.48	0.35	0.66	Bosnia and Herzegovina	Female	40-44 years	0.57	0.42	0.77
Bosnia and Herzegovina	Both	45-49 years	1.12	0.87	1.42	Bosnia and Herzegovina	Male	45-49 years	1.01	0.78	1.28	Bosnia and Herzegovina	Female	45-49 years	1.23	0.95	1.57
Bosnia and Herzegovina	Both	50-54 years	2.00	1.52	2.61	Bosnia and Herzegovina	Male	50-54 years	1.78	1.37	2.30	Bosnia and Herzegovina	Female	50-54 years	2.22	1.64	2.89
Bosnia and Herzegovina	Both	55-59 years	3.17	2.49	3.84	Bosnia and Herzegovina	Male	55-59 years	2.81	2.21	3.44	Bosnia and Herzegovina	Female	55-59 years	3.52	2.73	4.31
Bosnia and Herzegovina	Both	60-64 years	4.68	3.70	5.73	Bosnia and Herzegovina	Male	60-64 years	4.11	3.24	5.13	Bosnia and Herzegovina	Female	60-64 years	5.19	4.10	6.40
Bosnia and Herzegovina	Both	65-69 years	7.06	5.67	8.57	Bosnia and Herzegovina	Male	65-69 years	6.12	4.88	7.53	Bosnia and Herzegovina	Female	65-69 years	7.85	6.27	9.50
Bosnia and Herzegovina	Both	70-74 years	10.09	7.68	12.66	Bosnia and Herzegovina	Male	70-74 years	8.67	6.63	10.98	Bosnia and Herzegovina	Female	70-74 years	11.18	8.57	14.10
Bosnia and Herzegovina	Both	75-79 years	13.10	10.51	16.08	Bosnia and Herzegovina	Male	75-79 years	11.25	8.90	13.76	Bosnia and Herzegovina	Female	75-79 years	14.36	11.40	17.56
Bosnia and Herzegovina	Both	All ages	2.81	2.42	3.23	Bosnia and Herzegovina	Male	All ages	2.22	1.88	2.55	Bosnia and Herzegovina	Female	All ages	3.37	2.89	3.89
Bosnia and Herzegovina	Both	80-84	15.82	12.97	19.20	Bosnia and Herzegovina	Male	80-84	13.67	11.04	16.88	Bosnia and Herzegovina	Female	80-84	17.14	13.95	20.91

Bosnia and Herzegovina	Both	85-89	17.79	14.68	21.27	Bosnia and Herzegovina	Male	85-89	15.58	12.68	18.99	Bosnia and Herzegovina	Female	85-89	19.06	15.66	22.90
Bosnia and Herzegovina	Both	90-94	19.14	15.98	22.64	Bosnia and Herzegovina	Male	90-94	17.06	13.98	20.33	Bosnia and Herzegovina	Female	90-94	20.20	16.85	24.08
Botswana	Both	40-44 years	0.38	0.28	0.52	Botswana	Male	40-44 years	0.32	0.23	0.44	Botswana	Female	40-44 years	0.44	0.33	0.60
Botswana	Both	45-49 years	0.86	0.66	1.09	Botswana	Male	45-49 years	0.72	0.55	0.91	Botswana	Female	45-49 years	0.99	0.76	1.27
Botswana	Both	50-54 years	1.59	1.19	2.05	Botswana	Male	50-54 years	1.31	1.00	1.69	Botswana	Female	50-54 years	1.84	1.37	2.37
Botswana	Both	55-59 years	2.61	2.02	3.18	Botswana	Male	55-59 years	2.12	1.66	2.61	Botswana	Female	55-59 years	3.00	2.32	3.67
Botswana	Both	60-64 years	3.91	3.08	4.86	Botswana	Male	60-64 years	3.14	2.46	3.95	Botswana	Female	60-64 years	4.51	3.54	5.62
Botswana	Both	65-69 years	5.93	4.83	7.29	Botswana	Male	65-69 years	4.68	3.75	5.77	Botswana	Female	65-69 years	6.91	5.59	8.46
Botswana	Both	70-74 years	8.61	6.64	10.86	Botswana	Male	70-74 years	6.66	5.14	8.37	Botswana	Female	70-74 years	10.02	7.73	12.77
Botswana	Both	75-79 years	11.61	9.31	14.25	Botswana	Male	75-79 years	8.83	6.99	10.81	Botswana	Female	75-79 years	13.26	10.65	16.20
Botswana	Both	All ages	0.69	0.60	0.80	Botswana	Male	All ages	0.48	0.41	0.55	Botswana	Female	All ages	0.90	0.77	1.04
Botswana	Both	80-84	14.68	11.90	17.90	Botswana	Male	80-84	11.05	8.91	13.60	Botswana	Female	80-84	16.38	13.32	19.91
Botswana	Both	85-89	17.30	14.28	20.82	Botswana	Male	85-89	13.01	10.61	15.79	Botswana	Female	85-89	18.80	15.50	22.69
Botswana	Both	90-94	19.40	16.07	22.96	Botswana	Male	90-94	14.72	12.06	17.66	Botswana	Female	90-94	20.54	17.03	24.38
Brazil	Both	40-44 years	0.28	0.20	0.37	Brazil	Male	65-69 years	3.48	2.80	4.28	Brazil	Female	40-44 years	0.32	0.23	0.43
Brazil	Both	45-49 years	0.62	0.48	0.78	Brazil	Male	70-74 years	5.08	3.90	6.38	Brazil	Female	45-49 years	0.71	0.55	0.91
Brazil	Both	50-54 years	1.13	0.86	1.45	Brazil	Male	75-79 years	6.77	5.40	8.32	Brazil	Female	50-54 years	1.32	1.00	1.70
Brazil	Both	55-59 years	1.86	1.45	2.26	Brazil	Male	All ages	0.77	0.67	0.90	Brazil	Female	55-59 years	2.16	1.69	2.63
Brazil	Both	60-64 years	2.81	2.22	3.48	Brazil	Male	80-84	8.46	6.88	10.42	Brazil	Female	60-64 years	3.28	2.59	4.08
Brazil	Both	65-69 years	4.32	3.50	5.29	Brazil	Male	85-89	10.01	8.22	12.01	Brazil	Female	65-69 years	5.03	4.08	6.16
Brazil	Both	70-74 years	6.30	4.86	7.88	Brazil	Male	90-94	11.49	9.47	13.72	Brazil	Female	70-74 years	7.29	5.63	9.09
Brazil	Both	75-79 years	8.40	6.70	10.23	Brazil	Male	40-44 years	0.24	0.17	0.32	Brazil	Female	75-79 years	9.63	7.68	11.71
Brazil	Both	All ages	1.04	0.91	1.20	Brazil	Male	45-49 years	0.52	0.40	0.65	Brazil	Female	All ages	1.29	1.12	1.48

Brazil	Both	80-84	10.49	8.59	12.84	Brazil	Male	50-54 years	0.93	0.71	1.19	Brazil	Female	80-84	11.86	9.72	14.53
Brazil	Both	85-89	12.33	10.16	14.78	Brazil	Male	55-59 years	1.51	1.18	1.84	Brazil	Female	85-89	13.69	11.26	16.45
Brazil	Both	90-94	13.89	11.51	16.48	Brazil	Male	60-64 years	2.27	1.78	2.81	Brazil	Female	90-94	15.18	12.58	18.01
Brunei Darussalam	Both	40-44 years	0.70	0.52	0.91	Brunei Darussalam	Male	40-44 years	0.34	0.24	0.45	Brunei Darussalam	Female	40-44 years	1.11	0.83	1.43
Brunei Darussalam	Both	45-49 years	1.26	0.99	1.59	Brunei Darussalam	Male	45-49 years	0.71	0.54	0.91	Brunei Darussalam	Female	45-49 years	1.84	1.44	2.30
Brunei Darussalam	Both	50-54 years	1.93	1.49	2.43	Brunei Darussalam	Male	50-54 years	1.27	0.96	1.63	Brunei Darussalam	Female	50-54 years	2.61	2.02	3.32
Brunei Darussalam	Both	55-59 years	2.84	2.23	3.46	Brunei Darussalam	Male	55-59 years	2.11	1.67	2.57	Brunei Darussalam	Female	55-59 years	3.59	2.80	4.42
Brunei Darussalam	Both	60-64 years	4.13	3.27	5.11	Brunei Darussalam	Male	60-64 years	3.23	2.53	3.99	Brunei Darussalam	Female	60-64 years	5.01	3.94	6.16
Brunei Darussalam	Both	65-69 years	6.40	5.19	7.75	Brunei Darussalam	Male	65-69 years	4.84	3.91	5.86	Brunei Darussalam	Female	65-69 years	7.88	6.35	9.59
Brunei Darussalam	Both	70-74 years	9.47	7.35	11.94	Brunei Darussalam	Male	70-74 years	6.84	5.31	8.55	Brunei Darussalam	Female	70-74 years	11.84	9.20	15.05
Brunei Darussalam	Both	75-79 years	12.82	10.26	15.82	Brunei Darussalam	Male	75-79 years	8.85	7.04	10.93	Brunei Darussalam	Female	75-79 years	15.89	12.70	19.55
Brunei Darussalam	Both	All ages	0.97	0.84	1.11	Brunei Darussalam	Male	All ages	0.62	0.52	0.72	Brunei Darussalam	Female	All ages	1.35	1.16	1.55
Brunei Darussalam	Both	80-84	16.73	13.59	20.29	Brunei Darussalam	Male	80-84	10.82	8.62	13.26	Brunei Darussalam	Female	80-84	19.80	16.11	24.06
Brunei Darussalam	Both	85-89	21.29	17.62	25.51	Brunei Darussalam	Male	85-89	12.59	10.33	15.30	Brunei Darussalam	Female	85-89	22.88	18.95	27.47
Brunei Darussalam	Both	90-94	23.70	19.78	28.00	Brunei Darussalam	Male	90-94	14.09	11.65	16.86	Brunei Darussalam	Female	90-94	24.99	20.86	29.46
Bulgaria	Both	40-44 years	0.46	0.34	0.62	Bulgaria	Male	40-44 years	0.43	0.31	0.59	Bulgaria	Female	40-44 years	0.49	0.36	0.67
Bulgaria	Both	45-49 years	0.97	0.75	1.21	Bulgaria	Male	45-49 years	0.88	0.68	1.12	Bulgaria	Female	45-49 years	1.06	0.81	1.34
Bulgaria	Both	50-54 years	1.71	1.31	2.18	Bulgaria	Male	50-54 years	1.54	1.18	1.96	Bulgaria	Female	50-54 years	1.89	1.43	2.42
Bulgaria	Both	55-59 years	2.72	2.13	3.28	Bulgaria	Male	55-59 years	2.42	1.89	2.93	Bulgaria	Female	55-59 years	3.01	2.35	3.70

Bulgaria	Both	60-64 years	4.02	3.16	4.97	Bulgaria	Male	60-64 years	3.53	2.78	4.39	Bulgaria	Female	60-64 years	4.46	3.48	5.57
Bulgaria	Both	65-69 years	6.06	4.88	7.42	Bulgaria	Male	65-69 years	5.26	4.23	6.45	Bulgaria	Female	65-69 years	6.70	5.35	8.23
Bulgaria	Both	70-74 years	8.69	6.68	10.85	Bulgaria	Male	70-74 years	7.47	5.76	9.40	Bulgaria	Female	70-74 years	9.55	7.33	11.99
Bulgaria	Both	75-79 years	11.35	9.02	13.92	Bulgaria	Male	75-79 years	9.75	7.76	11.95	Bulgaria	Female	75-79 years	12.36	9.81	15.11
Bulgaria	Both	All ages	2.88	2.46	3.32	Bulgaria	Male	All ages	2.17	1.85	2.51	Bulgaria	Female	All ages	3.54	3.00	4.08
Bulgaria	Both	80-84	13.88	11.19	16.92	Bulgaria	Male	80-84	11.96	9.50	14.70	Bulgaria	Female	80-84	14.95	12.05	18.16
Bulgaria	Both	85-89	15.89	13.03	19.14	Bulgaria	Male	85-89	13.86	11.31	16.80	Bulgaria	Female	85-89	16.93	13.89	20.51
Bulgaria	Both	90-94	17.46	14.52	21.05	Bulgaria	Male	90-94	15.51	12.81	18.70	Bulgaria	Female	90-94	18.37	15.21	22.03
Burkina Faso	Both	40-44 years	0.29	0.20	0.38	Burkina Faso	Male	40-44 years	0.26	0.19	0.35	Burkina Faso	Female	40-44 years	0.30	0.22	0.41
Burkina Faso	Both	45-49 years	0.62	0.48	0.79	Burkina Faso	Male	45-49 years	0.57	0.44	0.73	Burkina Faso	Female	45-49 years	0.67	0.51	0.84
Burkina Faso	Both	50-54 years	1.13	0.86	1.45	Burkina Faso	Male	50-54 years	1.04	0.79	1.34	Burkina Faso	Female	50-54 years	1.21	0.91	1.55
Burkina Faso	Both	55-59 years	1.83	1.43	2.27	Burkina Faso	Male	55-59 years	1.66	1.29	2.06	Burkina Faso	Female	55-59 years	1.97	1.54	2.44
Burkina Faso	Both	60-64 years	2.70	2.14	3.39	Burkina Faso	Male	60-64 years	2.43	1.90	3.08	Burkina Faso	Female	60-64 years	2.94	2.35	3.68
Burkina Faso	Both	65-69 years	4.04	3.22	4.99	Burkina Faso	Male	65-69 years	3.58	2.83	4.45	Burkina Faso	Female	65-69 years	4.44	3.56	5.46
Burkina Faso	Both	70-74 years	5.78	4.52	7.32	Burkina Faso	Male	70-74 years	5.05	3.90	6.37	Burkina Faso	Female	70-74 years	6.37	4.97	8.12
Burkina Faso	Both	75-79 years	7.63	6.15	9.29	Burkina Faso	Male	75-79 years	6.65	5.31	8.14	Burkina Faso	Female	75-79 years	8.41	6.79	10.21
Burkina Faso	Both	All ages	0.32	0.28	0.37	Burkina Faso	Male	All ages	0.26	0.23	0.31	Burkina Faso	Female	All ages	0.37	0.32	0.43
Burkina Faso	Both	80-84	9.49	7.68	11.65	Burkina Faso	Male	80-84	8.28	6.62	10.14	Burkina Faso	Female	80-84	10.39	8.34	12.82
Burkina Faso	Both	85-89	11.12	9.00	13.46	Burkina Faso	Male	85-89	9.75	7.95	11.79	Burkina Faso	Female	85-89	12.03	9.77	14.68
Burkina Faso	Both	90-94	12.57	10.23	15.23	Burkina Faso	Male	90-94	11.08	9.12	13.46	Burkina Faso	Female	90-94	13.37	10.88	16.34
Burundi	Both	40-44 years	0.30	0.21	0.40	Burundi	Male	40-44 years	0.27	0.19	0.36	Burundi	Female	40-44 years	0.33	0.24	0.45
Burundi	Both	45-49 years	0.65	0.50	0.83	Burundi	Male	45-49 years	0.58	0.44	0.74	Burundi	Female	45-49 years	0.73	0.56	0.93
Burundi	Both	50-54 years	1.18	0.90	1.49	Burundi	Male	50-54 years	1.04	0.79	1.32	Burundi	Female	50-54 years	1.33	1.01	1.71
Burundi	Both	55-59 years	1.90	1.48	2.33	Burundi	Male	55-59 years	1.67	1.31	2.05	Burundi	Female	55-59 years	2.16	1.68	2.69

Burundi	Both	60-64 years	2.81	2.21	3.53	Burundi	Male	60-64 years	2.45	1.92	3.08	Burundi	Female	60-64 years	3.22	2.52	4.07
Burundi	Both	65-69 years	4.21	3.35	5.16	Burundi	Male	65-69 years	3.63	2.88	4.48	Burundi	Female	65-69 years	4.87	3.85	6.06
Burundi	Both	70-74 years	6.04	4.62	7.54	Burundi	Male	70-74 years	5.15	3.98	6.45	Burundi	Female	70-74 years	7.00	5.38	8.77
Burundi	Both	75-79 years	8.01	6.47	9.74	Burundi	Male	75-79 years	6.78	5.46	8.25	Burundi	Female	75-79 years	9.25	7.40	11.28
Burundi	Both	All ages	0.31	0.27	0.36	Burundi	Male	All ages	0.28	0.24	0.33	Burundi	Female	All ages	0.34	0.29	0.39
Burundi	Both	80-84	10.01	8.06	12.20	Burundi	Male	80-84	8.41	6.70	10.34	Burundi	Female	80-84	11.41	9.27	13.91
Burundi	Both	85-89	11.72	9.61	14.06	Burundi	Male	85-89	9.84	7.93	11.86	Burundi	Female	85-89	13.07	10.75	15.75
Burundi	Both	90-94	13.17	10.88	15.78	Burundi	Male	90-94	11.11	9.05	13.36	Burundi	Female	90-94	14.30	11.78	17.24
Cabo Verde	Both	60-64 years	3.10	2.44	3.85	Cabo Verde	Male	60-64 years	2.73	2.14	3.40	Cabo Verde	Female	40-44 years	0.33	0.23	0.46
Cabo Verde	Both	65-69 years	4.68	3.77	5.75	Cabo Verde	Male	65-69 years	4.06	3.25	4.99	Cabo Verde	Female	45-49 years	0.73	0.56	0.93
Cabo Verde	Both	70-74 years	6.69	5.14	8.45	Cabo Verde	Male	70-74 years	5.76	4.52	7.29	Cabo Verde	Female	50-54 years	1.34	1.00	1.74
Cabo Verde	Both	75-79 years	8.84	7.07	10.77	Cabo Verde	Male	75-79 years	7.57	6.11	9.33	Cabo Verde	Female	55-59 years	2.23	1.75	2.72
Cabo Verde	Both	All ages	0.77	0.66	0.87	Cabo Verde	Male	All ages	0.57	0.49	0.65	Cabo Verde	Female	60-64 years	3.39	2.66	4.22
Cabo Verde	Both	80-84	10.96	8.90	13.46	Cabo Verde	Male	80-84	9.38	7.51	11.55	Cabo Verde	Female	65-69 years	5.13	4.12	6.36
Cabo Verde	Both	85-89	12.74	10.45	15.40	Cabo Verde	Male	85-89	11.02	9.01	13.37	Cabo Verde	Female	70-74 years	7.32	5.58	9.27
Cabo Verde	Both	90-94	14.23	11.69	17.04	Cabo Verde	Male	90-94	12.51	10.19	15.10	Cabo Verde	Female	75-79 years	9.62	7.67	11.81
Cabo Verde	Both	40-44 years	0.31	0.22	0.42	Cabo Verde	Male	40-44 years	0.29	0.20	0.39	Cabo Verde	Female	All ages	0.96	0.84	1.10
Cabo Verde	Both	45-49 years	0.68	0.52	0.86	Cabo Verde	Male	45-49 years	0.63	0.49	0.81	Cabo Verde	Female	80-84	11.84	9.59	14.53
Cabo Verde	Both	50-54 years	1.24	0.93	1.61	Cabo Verde	Male	50-54 years	1.14	0.86	1.50	Cabo Verde	Female	85-89	13.70	11.23	16.62
Cabo Verde	Both	55-59 years	2.04	1.60	2.50	Cabo Verde	Male	55-59 years	1.84	1.43	2.26	Cabo Verde	Female	90-94	15.22	12.47	18.22
Cambodia	Both	40-44 years	0.55	0.41	0.72	Cambodia	Male	40-44 years	0.48	0.36	0.65	Cambodia	Female	40-44 years	0.61	0.45	0.80
Cambodia	Both	45-49 years	1.20	0.93	1.52	Cambodia	Male	45-49 years	1.02	0.80	1.28	Cambodia	Female	45-49 years	1.36	1.04	1.73
Cambodia	Both	50-54 years	2.15	1.64	2.78	Cambodia	Male	50-54 years	1.79	1.38	2.30	Cambodia	Female	50-54 years	2.46	1.86	3.20
Cambodia	Both	55-59 years	3.33	2.61	4.07	Cambodia	Male	55-59 years	2.74	2.12	3.35	Cambodia	Female	55-59 years	3.81	2.99	4.71

Cambodia	Both	60-64 years	4.74	3.68	5.84	Cambodia	Male	60-64 years	3.82	2.99	4.66	Cambodia	Female	60-64 years	5.39	4.15	6.67
Cambodia	Both	65-69 years	6.79	5.44	8.33	Cambodia	Male	65-69 years	5.29	4.24	6.45	Cambodia	Female	65-69 years	7.75	6.15	9.47
Cambodia	Both	70-74 years	9.28	7.22	11.70	Cambodia	Male	70-74 years	7.09	5.56	8.84	Cambodia	Female	70-74 years	10.69	8.29	13.56
Cambodia	Both	75-79 years	11.72	9.37	14.34	Cambodia	Male	75-79 years	8.84	7.08	10.75	Cambodia	Female	75-79 years	13.51	10.73	16.64
Cambodia	Both	All ages	0.99	0.85	1.14	Cambodia	Male	All ages	0.67	0.57	0.77	Cambodia	Female	All ages	1.30	1.11	1.49
Cambodia	Both	80-84	13.93	11.55	16.99	Cambodia	Male	80-84	10.43	8.48	12.72	Cambodia	Female	80-84	15.95	13.07	19.52
Cambodia	Both	85-89	15.50	12.82	18.67	Cambodia	Male	85-89	11.67	9.59	14.08	Cambodia	Female	85-89	17.56	14.48	21.18
Cambodia	Both	90-94	16.62	13.91	19.67	Cambodia	Male	90-94	12.62	10.45	15.00	Cambodia	Female	90-94	18.47	15.41	21.94
Cameroon	Both	40-44 years	0.29	0.21	0.39	Cameroon	Male	40-44 years	0.27	0.19	0.36	Cameroon	Female	80-84	10.81	8.67	13.39
Cameroon	Both	45-49 years	0.64	0.49	0.82	Cameroon	Male	45-49 years	0.59	0.45	0.76	Cameroon	Female	85-89	12.59	10.30	15.36
Cameroon	Both	50-54 years	1.16	0.88	1.52	Cameroon	Male	50-54 years	1.07	0.81	1.39	Cameroon	Female	90-94	14.06	11.55	17.04
Cameroon	Both	55-59 years	1.87	1.47	2.29	Cameroon	Male	55-59 years	1.71	1.32	2.10	Cameroon	Female	40-44 years	0.31	0.23	0.42
Cameroon	Both	60-64 years	2.77	2.16	3.46	Cameroon	Male	60-64 years	2.50	1.94	3.15	Cameroon	Female	45-49 years	0.69	0.53	0.89
Cameroon	Both	65-69 years	4.15	3.32	5.13	Cameroon	Male	65-69 years	3.71	2.96	4.60	Cameroon	Female	50-54 years	1.25	0.94	1.64
Cameroon	Both	70-74 years	5.96	4.59	7.53	Cameroon	Male	70-74 years	5.28	4.08	6.69	Cameroon	Female	55-59 years	2.04	1.58	2.52
Cameroon	Both	75-79 years	7.92	6.28	9.74	Cameroon	Male	75-79 years	6.97	5.55	8.60	Cameroon	Female	60-64 years	3.04	2.38	3.82
Cameroon	Both	All ages	0.34	0.29	0.39	Cameroon	Male	All ages	0.30	0.25	0.34	Cameroon	Female	65-69 years	4.59	3.65	5.64
Cameroon	Both	80-84	9.91	7.97	12.26	Cameroon	Male	80-84	8.67	6.90	10.73	Cameroon	Female	70-74 years	6.59	5.08	8.40
Cameroon	Both	85-89	11.67	9.54	14.15	Cameroon	Male	85-89	10.21	8.27	12.35	Cameroon	Female	75-79 years	8.72	6.97	10.80
Cameroon	Both	90-94	13.24	10.94	16.00	Cameroon	Male	90-94	11.62	9.56	13.92	Cameroon	Female	All ages	0.38	0.33	0.44
Canada	Both	40-44 years	1.09	0.82	1.41	Canada	Male	40-44 years	0.66	0.49	0.89	Canada	Female	40-44 years	1.49	1.11	1.94
Canada	Both	45-49 years	1.89	1.49	2.32	Canada	Male	45-49 years	1.37	1.06	1.73	Canada	Female	45-49 years	2.37	1.88	2.98
Canada	Both	50-54 years	2.86	2.23	3.55	Canada	Male	50-54 years	2.46	1.88	3.13	Canada	Female	50-54 years	3.23	2.52	4.07
Canada	Both	55-59 years	4.30	3.43	5.18	Canada	Male	55-59 years	4.19	3.26	5.09	Canada	Female	55-59 years	4.41	3.50	5.37

Canada	Both	60-64 years	6.42	5.14	7.85	Canada	Male	60-64 years	6.57	5.20	8.21	Canada	Female	60-64 years	6.27	4.99	7.62
Canada	Both	65-69 years	10.04	8.19	12.16	Canada	Male	65-69 years	10.04	8.08	12.34	Canada	Female	65-69 years	10.05	8.19	12.14
Canada	Both	70-74 years	14.62	11.40	18.24	Canada	Male	70-74 years	14.14	10.93	17.66	Canada	Female	70-74 years	15.05	11.73	19.07
Canada	Both	75-79 years	18.89	15.15	22.92	Canada	Male	75-79 years	17.85	14.09	21.99	Canada	Female	75-79 years	19.79	16.03	24.31
Canada	Both	All ages	4.37	3.78	4.98	Canada	Male	All ages	3.91	3.34	4.53	Canada	Female	All ages	4.80	4.14	5.48
Canada	Both	80-84	22.79	18.88	27.16	Canada	Male	80-84	21.09	17.20	25.43	Canada	Female	80-84	24.13	19.86	28.95
Canada	Both	85-89	26.15	21.99	30.97	Canada	Male	85-89	23.70	19.71	28.19	Canada	Female	85-89	27.78	23.42	33.19
Canada	Both	90-94	28.81	24.34	33.72	Canada	Male	90-94	25.52	21.36	30.04	Canada	Female	90-94	30.41	25.66	35.71
Central African Republic	Both	40-44 years	0.34	0.24	0.45	Central African Republic	Male	40-44 years	0.29	0.21	0.38	Central African Republic	Female	40-44 years	0.39	0.28	0.52
Central African Republic	Both	45-49 years	0.75	0.58	0.95	Central African Republic	Male	45-49 years	0.63	0.48	0.80	Central African Republic	Female	45-49 years	0.88	0.68	1.13
Central African Republic	Both	50-54 years	1.37	1.03	1.76	Central African Republic	Male	50-54 years	1.13	0.86	1.45	Central African Republic	Female	50-54 years	1.61	1.22	2.07
Central African Republic	Both	55-59 years	2.22	1.74	2.73	Central African Republic	Male	55-59 years	1.81	1.43	2.21	Central African Republic	Female	55-59 years	2.61	2.03	3.25
Central African Republic	Both	60-64 years	3.33	2.63	4.17	Central African Republic	Male	60-64 years	2.66	2.09	3.28	Central African Republic	Female	60-64 years	3.90	3.10	4.92
Central African Republic	Both	65-69 years	5.12	4.08	6.26	Central African Republic	Male	65-69 years	3.96	3.13	4.83	Central African Republic	Female	65-69 years	6.03	4.79	7.38
Central African Republic	Both	70-74 years	7.54	5.77	9.43	Central African Republic	Male	70-74 years	5.64	4.30	7.06	Central African Republic	Female	70-74 years	8.80	6.74	11.12
Central African Republic	Both	75-79 years	10.11	8.13	12.33	Central African Republic	Male	75-79 years	7.42	5.93	9.04	Central African Republic	Female	75-79 years	11.55	9.30	14.06
Central African Republic	Both	All ages	0.38	0.32	0.43	Central African Republic	Male	All ages	0.27	0.23	0.31	Central African Republic	Female	All ages	0.48	0.41	0.55
Central African Republic	Both	80-84	12.52	10.11	15.22	Central African Republic	Male	80-84	9.17	7.30	11.22	Central African Republic	Female	80-84	14.04	11.29	17.08
Central African Republic	Both	85-89	14.33	11.69	17.24	Central African Republic	Male	85-89	10.66	8.66	12.78	Central African Republic	Female	85-89	15.81	12.83	19.02
Central African	Both	90-94	15.62	12.87	18.56	Central African	Male	90-94	11.91	9.77	14.40	Central African	Female	90-94	16.95	13.85	20.13

Republic						Republic						Republic					
Chad	Both	40-44 years	0.28	0.20	0.38	Chad	Male	40-44 years	0.26	0.19	0.35	Chad	Female	40-44 years	0.30	0.22	0.41
Chad	Both	45-49 years	0.62	0.47	0.79	Chad	Male	45-49 years	0.57	0.44	0.74	Chad	Female	45-49 years	0.66	0.51	0.83
Chad	Both	50-54 years	1.11	0.83	1.43	Chad	Male	50-54 years	1.03	0.77	1.35	Chad	Female	50-54 years	1.20	0.90	1.53
Chad	Both	55-59 years	1.78	1.40	2.19	Chad	Male	55-59 years	1.65	1.27	2.03	Chad	Female	55-59 years	1.95	1.54	2.39
Chad	Both	60-64 years	2.63	2.07	3.27	Chad	Male	60-64 years	2.41	1.88	3.03	Chad	Female	60-64 years	2.89	2.30	3.59
Chad	Both	65-69 years	3.91	3.15	4.78	Chad	Male	65-69 years	3.56	2.88	4.41	Chad	Female	65-69 years	4.36	3.48	5.37
Chad	Both	70-74 years	5.56	4.34	6.97	Chad	Male	70-74 years	5.03	3.94	6.42	Chad	Female	70-74 years	6.26	4.82	7.92
Chad	Both	75-79 years	7.37	5.96	9.02	Chad	Male	75-79 years	6.62	5.34	8.19	Chad	Female	75-79 years	8.29	6.68	10.18
Chad	Both	All ages	0.27	0.23	0.31	Chad	Male	All ages	0.27	0.23	0.31	Chad	Female	All ages	0.27	0.23	0.31
Chad	Both	80-84	9.21	7.50	11.28	Chad	Male	80-84	8.24	6.64	10.20	Chad	Female	80-84	10.30	8.30	12.66
Chad	Both	85-89	10.84	8.87	13.03	Chad	Male	85-89	9.70	7.88	11.74	Chad	Female	85-89	11.97	9.75	14.39
Chad	Both	90-94	12.44	10.23	14.89	Chad	Male	90-94	11.04	9.00	13.27	Chad	Female	90-94	13.34	10.99	15.96
Chile	Both	40-44 years	0.74	0.55	0.99	Chile	Male	40-44 years	0.39	0.28	0.53	Chile	Female	40-44 years	1.07	0.79	1.43
Chile	Both	45-49 years	1.33	1.04	1.65	Chile	Male	45-49 years	0.84	0.64	1.05	Chile	Female	45-49 years	1.79	1.40	2.25
Chile	Both	50-54 years	2.06	1.60	2.58	Chile	Male	50-54 years	1.52	1.15	1.92	Chile	Female	50-54 years	2.57	1.98	3.24
Chile	Both	55-59 years	3.08	2.45	3.75	Chile	Male	55-59 years	2.56	2.01	3.15	Chile	Female	55-59 years	3.55	2.82	4.33
Chile	Both	60-64 years	4.50	3.61	5.53	Chile	Male	60-64 years	3.94	3.11	4.91	Chile	Female	60-64 years	5.00	4.01	6.14
Chile	Both	65-69 years	6.98	5.63	8.50	Chile	Male	65-69 years	5.96	4.76	7.30	Chile	Female	65-69 years	7.87	6.34	9.58
Chile	Both	70-74 years	10.24	7.94	12.79	Chile	Male	70-74 years	8.43	6.47	10.49	Chile	Female	70-74 years	11.75	9.12	14.83
Chile	Both	75-79 years	13.49	10.84	16.43	Chile	Male	75-79 years	10.74	8.50	13.14	Chile	Female	75-79 years	15.58	12.50	19.04
Chile	Both	All ages	2.17	1.86	2.48	Chile	Male	All ages	1.59	1.37	1.86	Chile	Female	All ages	2.71	2.32	3.08
Chile	Both	80-84	16.52	13.45	19.88	Chile	Male	80-84	12.79	10.40	15.71	Chile	Female	80-84	19.07	15.44	22.82
Chile	Both	85-89	18.95	15.78	22.48	Chile	Male	85-89	14.39	11.72	17.39	Chile	Female	85-89	21.69	18.02	25.94

Chile	Both	90-94	20.54	17.25	24.41	Chile	Male	90-94	15.56	12.87	18.53	Chile	Female	90-94	23.39	19.66	27.76
China	Both	40-44 years	0.51	0.38	0.67	China	Male	40-44 years	0.32	0.23	0.43	China	Female	40-44 years	0.71	0.53	0.91
China	Both	45-49 years	1.21	0.94	1.52	China	Male	45-49 years	0.69	0.54	0.87	China	Female	45-49 years	1.73	1.35	2.20
China	Both	50-54 years	2.26	1.72	2.92	China	Male	50-54 years	1.24	0.95	1.57	China	Female	50-54 years	3.29	2.49	4.26
China	Both	55-59 years	3.52	2.76	4.27	China	Male	55-59 years	1.90	1.51	2.30	China	Female	55-59 years	5.14	4.02	6.26
China	Both	60-64 years	4.90	3.83	5.95	China	Male	60-64 years	2.64	2.09	3.21	China	Female	60-64 years	7.18	5.60	8.75
China	Both	65-69 years	6.73	5.48	8.19	China	Male	65-69 years	3.53	2.90	4.30	China	Female	65-69 years	9.81	7.97	11.94
China	Both	70-74 years	8.80	6.94	10.88	China	Male	70-74 years	4.55	3.60	5.63	China	Female	70-74 years	12.84	10.09	15.88
China	Both	75-79 years	10.91	8.85	13.21	China	Male	75-79 years	5.57	4.49	6.71	China	Female	75-79 years	15.71	12.70	19.06
China	Both	All ages	2.09	1.80	2.39	China	Male	All ages	1.05	0.90	1.20	China	Female	All ages	3.15	2.71	3.60
China	Both	80-84	13.11	10.77	15.85	China	Male	80-84	6.57	5.40	7.96	China	Female	80-84	18.24	14.91	22.04
China	Both	85-89	15.61	12.97	18.74	China	Male	85-89	7.53	6.21	8.98	China	Female	85-89	19.96	16.55	23.94
China	Both	90-94	18.88	15.87	22.30	China	Male	90-94	8.66	7.15	10.30	China	Female	90-94	21.01	17.58	24.85
Colombia	Both	45-49 years	0.64	0.50	0.81	Colombia	Male	40-44 years	0.29	0.20	0.39	Colombia	Female	45-49 years	0.69	0.53	0.88
Colombia	Both	50-54 years	1.13	0.86	1.44	Colombia	Male	45-49 years	0.59	0.45	0.75	Colombia	Female	50-54 years	1.21	0.92	1.56
Colombia	Both	55-59 years	1.82	1.41	2.21	Colombia	Male	50-54 years	1.03	0.79	1.32	Colombia	Female	55-59 years	1.96	1.51	2.40
Colombia	Both	60-64 years	2.80	2.17	3.47	Colombia	Male	55-59 years	1.66	1.28	2.06	Colombia	Female	60-64 years	3.01	2.33	3.71
Colombia	Both	65-69 years	4.49	3.57	5.47	Colombia	Male	60-64 years	2.55	1.97	3.20	Colombia	Female	65-69 years	4.79	3.82	5.91
Colombia	Both	70-74 years	6.73	5.19	8.52	Colombia	Male	65-69 years	4.13	3.31	5.05	Colombia	Female	70-74 years	7.14	5.47	9.07
Colombia	Both	75-79 years	8.98	7.21	11.03	Colombia	Male	70-74 years	6.23	4.78	7.96	Colombia	Female	75-79 years	9.53	7.60	11.74
Colombia	Both	All ages	1.14	0.98	1.30	Colombia	Male	75-79 years	8.31	6.69	10.39	Colombia	Female	All ages	1.28	1.11	1.46
Colombia	Both	80-84	11.07	9.06	13.50	Colombia	Male	All ages	0.98	0.83	1.12	Colombia	Female	80-84	11.72	9.44	14.32
Colombia	Both	40-44 years	0.31	0.22	0.42	Colombia	Male	80-84	10.20	8.18	12.60	Colombia	Female	85-89	13.35	10.82	16.20
Colombia	Both	85-89	12.66	10.35	15.35	Colombia	Male	85-89	11.71	9.54	14.23	Colombia	Female	90-94	14.51	11.82	17.43

Colombia	Both	90-94	13.85	11.38	16.60	Colombia	Male	90-94	12.93	10.66	15.52	Colombia	Female	40-44 years	0.33	0.24	0.45
Comoros	Both	90-94	13.42	11.02	16.07	Comoros	Male	90-94	11.35	9.18	13.79	Comoros	Female	90-94	14.67	12.00	17.65
Comoros	Both	40-44 years	0.30	0.22	0.40	Comoros	Male	40-44 years	0.27	0.19	0.36	Comoros	Female	40-44 years	0.33	0.24	0.44
Comoros	Both	45-49 years	0.66	0.51	0.84	Comoros	Male	45-49 years	0.58	0.45	0.74	Comoros	Female	45-49 years	0.74	0.56	0.95
Comoros	Both	50-54 years	1.20	0.90	1.53	Comoros	Male	50-54 years	1.05	0.78	1.35	Comoros	Female	50-54 years	1.35	1.00	1.75
Comoros	Both	55-59 years	1.95	1.53	2.38	Comoros	Male	55-59 years	1.69	1.31	2.08	Comoros	Female	55-59 years	2.18	1.70	2.69
Comoros	Both	60-64 years	2.91	2.29	3.66	Comoros	Male	60-64 years	2.49	1.95	3.13	Comoros	Female	60-64 years	3.25	2.55	4.12
Comoros	Both	65-69 years	4.38	3.49	5.43	Comoros	Male	65-69 years	3.68	2.93	4.56	Comoros	Female	65-69 years	4.93	3.93	6.11
Comoros	Both	70-74 years	6.28	4.84	7.91	Comoros	Male	70-74 years	5.21	4.05	6.57	Comoros	Female	70-74 years	7.09	5.43	9.03
Comoros	Both	75-79 years	8.28	6.56	10.11	Comoros	Male	75-79 years	6.86	5.47	8.42	Comoros	Female	75-79 years	9.36	7.40	11.59
Comoros	Both	All ages	0.63	0.54	0.72	Comoros	Male	All ages	0.49	0.42	0.56	Comoros	Female	All ages	0.77	0.65	0.89
Comoros	Both	80-84	10.24	8.27	12.74	Comoros	Male	80-84	8.51	6.80	10.49	Comoros	Female	80-84	11.55	9.24	14.38
Comoros	Both	85-89	11.92	9.68	14.50	Comoros	Male	85-89	10.00	8.12	12.16	Comoros	Female	85-89	13.31	10.81	16.25
Congo	Both	80-84	12.54	10.15	15.32	Congo	Male	40-44 years	0.29	0.21	0.40	Congo	Female	40-44 years	0.40	0.29	0.54
Congo	Both	85-89	14.61	12.03	17.54	Congo	Male	45-49 years	0.64	0.49	0.82	Congo	Female	45-49 years	0.89	0.69	1.13
Congo	Both	90-94	16.23	13.53	19.47	Congo	Male	50-54 years	1.16	0.87	1.47	Congo	Female	50-54 years	1.65	1.24	2.11
Congo	Both	40-44 years	0.34	0.25	0.46	Congo	Male	55-59 years	1.87	1.45	2.30	Congo	Female	55-59 years	2.69	2.09	3.33
Congo	Both	45-49 years	0.76	0.59	0.96	Congo	Male	60-64 years	2.76	2.18	3.44	Congo	Female	60-64 years	4.04	3.17	5.06
Congo	Both	50-54 years	1.39	1.06	1.77	Congo	Male	65-69 years	4.11	3.32	5.08	Congo	Female	65-69 years	6.23	4.98	7.75
Congo	Both	55-59 years	2.27	1.76	2.80	Congo	Male	70-74 years	5.84	4.47	7.42	Congo	Female	70-74 years	9.10	6.99	11.60
Congo	Both	60-64 years	3.41	2.69	4.26	Congo	Male	75-79 years	7.71	6.09	9.48	Congo	Female	75-79 years	12.00	9.46	14.77
Congo	Both	65-69 years	5.25	4.22	6.48	Congo	Male	All ages	0.39	0.33	0.45	Congo	Female	80-84	14.71	11.90	17.97
Congo	Both	70-74 years	7.61	5.87	9.68	Congo	Male	80-84	9.59	7.70	11.77	Congo	Female	85-89	16.78	13.79	20.36
Congo	Both	75-79 years	10.07	8.02	12.37	Congo	Male	85-89	11.26	9.28	13.59	Congo	Female	90-94	18.26	15.17	21.92

Congo	Both	All ages	0.50	0.43	0.57	Congo	Male	90-94	12.73	10.56	15.14	Congo	Female	All ages	0.61	0.52	0.70
Cook Islands	Both	80-84	15.79	13.07	19.14	Cook Islands	Male	40-44 years	0.54	0.40	0.71	Cook Islands	Female	80-84	18.17	15.04	22.06
Cook Islands	Both	85-89	17.55	14.66	21.03	Cook Islands	Male	45-49 years	1.16	0.90	1.46	Cook Islands	Female	85-89	20.40	16.98	24.54
Cook Islands	Both	90-94	19.27	16.13	22.73	Cook Islands	Male	50-54 years	2.06	1.55	2.65	Cook Islands	Female	90-94	21.96	18.30	25.96
Cook Islands	Both	40-44 years	0.60	0.45	0.79	Cook Islands	Male	55-59 years	3.19	2.48	3.92	Cook Islands	Female	40-44 years	0.66	0.49	0.87
Cook Islands	Both	45-49 years	1.32	1.02	1.65	Cook Islands	Male	60-64 years	4.51	3.54	5.57	Cook Islands	Female	45-49 years	1.46	1.14	1.86
Cook Islands	Both	50-54 years	2.37	1.81	3.06	Cook Islands	Male	65-69 years	6.30	5.09	7.66	Cook Islands	Female	50-54 years	2.66	2.03	3.43
Cook Islands	Both	55-59 years	3.66	2.85	4.44	Cook Islands	Male	70-74 years	8.45	6.60	10.46	Cook Islands	Female	55-59 years	4.17	3.25	5.10
Cook Islands	Both	60-64 years	5.27	4.12	6.51	Cook Islands	Male	75-79 years	10.61	8.56	12.93	Cook Islands	Female	60-64 years	6.00	4.67	7.37
Cook Islands	Both	65-69 years	7.44	6.04	9.00	Cook Islands	Male	All ages	1.96	1.67	2.24	Cook Islands	Female	65-69 years	8.66	7.01	10.65
Cook Islands	Both	70-74 years	10.26	8.01	12.67	Cook Islands	Male	80-84	12.64	10.28	15.45	Cook Islands	Female	70-74 years	11.95	9.32	14.82
Cook Islands	Both	75-79 years	12.92	10.40	15.76	Cook Islands	Male	85-89	14.30	11.77	17.20	Cook Islands	Female	75-79 years	15.20	12.19	18.61
Cook Islands	Both	All ages	2.29	1.98	2.63	Cook Islands	Male	90-94	15.66	12.99	18.61	Cook Islands	Female	All ages	2.61	2.24	3.01
Costa Rica	Both	40-44 years	0.32	0.23	0.43	Costa Rica	Male	40-44 years	0.27	0.19	0.36	Costa Rica	Female	40-44 years	0.36	0.26	0.49
Costa Rica	Both	45-49 years	0.65	0.51	0.83	Costa Rica	Male	45-49 years	0.55	0.42	0.70	Costa Rica	Female	45-49 years	0.75	0.58	0.96
Costa Rica	Both	50-54 years	1.15	0.88	1.47	Costa Rica	Male	50-54 years	0.95	0.73	1.21	Costa Rica	Female	50-54 years	1.33	1.01	1.72
Costa Rica	Both	55-59 years	1.88	1.47	2.30	Costa Rica	Male	55-59 years	1.54	1.21	1.90	Costa Rica	Female	55-59 years	2.17	1.70	2.68
Costa Rica	Both	60-64 years	2.89	2.28	3.57	Costa Rica	Male	60-64 years	2.36	1.88	2.90	Costa Rica	Female	60-64 years	3.36	2.62	4.15
Costa Rica	Both	65-69 years	4.63	3.73	5.63	Costa Rica	Male	65-69 years	3.79	3.02	4.62	Costa Rica	Female	65-69 years	5.37	4.29	6.56
Costa Rica	Both	70-74 years	6.92	5.41	8.67	Costa Rica	Male	70-74 years	5.68	4.38	7.26	Costa Rica	Female	70-74 years	8.00	6.27	10.04
Costa Rica	Both	75-79 years	9.23	7.41	11.37	Costa Rica	Male	75-79 years	7.58	5.96	9.34	Costa Rica	Female	75-79 years	10.63	8.55	13.07
Costa Rica	Both	All ages	1.14	0.97	1.30	Costa Rica	Male	All ages	0.90	0.77	1.04	Costa Rica	Female	All ages	1.35	1.16	1.55
Costa Rica	Both	80-84	11.38	9.18	14.03	Costa Rica	Male	80-84	9.34	7.39	11.71	Costa Rica	Female	80-84	13.01	10.46	16.07
Costa Rica	Both	85-89	13.07	10.58	16.01	Costa Rica	Male	85-89	10.80	8.63	13.17	Costa Rica	Female	85-89	14.72	11.96	18.14

Costa Rica	Both	90-94	14.34	11.72	17.22	Costa Rica	Male	90-94	12.04	9.80	14.48	Costa Rica	Female	90-94	15.86	12.96	19.13
Croatia	Both	40-44 years	0.44	0.32	0.58	Croatia	Male	80-84	11.51	9.28	14.06	Croatia	Female	40-44 years	0.48	0.35	0.63
Croatia	Both	45-49 years	0.93	0.72	1.16	Croatia	Male	85-89	13.40	10.95	16.14	Croatia	Female	45-49 years	1.01	0.77	1.27
Croatia	Both	50-54 years	1.64	1.25	2.06	Croatia	Male	90-94	15.07	12.41	17.99	Croatia	Female	50-54 years	1.81	1.35	2.31
Croatia	Both	55-59 years	2.62	2.07	3.20	Croatia	Male	40-44 years	0.41	0.30	0.54	Croatia	Female	55-59 years	2.91	2.26	3.54
Croatia	Both	60-64 years	3.90	3.06	4.85	Croatia	Male	45-49 years	0.85	0.65	1.07	Croatia	Female	60-64 years	4.34	3.40	5.36
Croatia	Both	65-69 years	5.85	4.70	7.15	Croatia	Male	50-54 years	1.47	1.12	1.86	Croatia	Female	65-69 years	6.53	5.26	8.01
Croatia	Both	70-74 years	8.36	6.44	10.53	Croatia	Male	55-59 years	2.33	1.83	2.88	Croatia	Female	70-74 years	9.24	7.15	11.71
Croatia	Both	75-79 years	10.93	8.76	13.44	Croatia	Male	60-64 years	3.42	2.69	4.26	Croatia	Female	75-79 years	11.95	9.50	14.76
Croatia	Both	All ages	2.74	2.35	3.14	Croatia	Male	65-69 years	5.07	4.08	6.20	Croatia	Female	All ages	3.36	2.87	3.87
Croatia	Both	80-84	13.40	10.94	16.34	Croatia	Male	70-74 years	7.18	5.48	9.07	Croatia	Female	80-84	14.44	11.70	17.58
Croatia	Both	85-89	15.46	12.75	18.64	Croatia	Male	75-79 years	9.37	7.47	11.48	Croatia	Female	85-89	16.40	13.57	19.82
Croatia	Both	90-94	17.13	14.24	20.35	Croatia	Male	All ages	2.06	1.76	2.38	Croatia	Female	90-94	17.86	14.86	21.28
Cuba	Both	40-44 years	0.32	0.23	0.44	Cuba	Male	40-44 years	0.28	0.20	0.37	Cuba	Female	40-44 years	0.37	0.26	0.51
Cuba	Both	45-49 years	0.68	0.52	0.86	Cuba	Male	45-49 years	0.57	0.44	0.72	Cuba	Female	45-49 years	0.78	0.60	0.99
Cuba	Both	50-54 years	1.20	0.92	1.53	Cuba	Male	50-54 years	1.00	0.76	1.28	Cuba	Female	50-54 years	1.39	1.05	1.80
Cuba	Both	55-59 years	1.94	1.51	2.38	Cuba	Male	55-59 years	1.61	1.27	1.98	Cuba	Female	55-59 years	2.26	1.73	2.78
Cuba	Both	60-64 years	2.94	2.29	3.66	Cuba	Male	60-64 years	2.42	1.90	3.03	Cuba	Female	60-64 years	3.42	2.64	4.31
Cuba	Both	65-69 years	4.60	3.65	5.60	Cuba	Male	65-69 years	3.77	2.99	4.63	Cuba	Female	65-69 years	5.35	4.25	6.59
Cuba	Both	70-74 years	6.75	5.20	8.49	Cuba	Male	70-74 years	5.53	4.23	6.98	Cuba	Female	70-74 years	7.84	5.98	10.00
Cuba	Both	75-79 years	8.96	7.15	11.02	Cuba	Male	75-79 years	7.34	5.81	8.91	Cuba	Female	75-79 years	10.31	8.18	12.71
Cuba	Both	All ages	1.75	1.50	2.01	Cuba	Male	All ages	1.36	1.17	1.56	Cuba	Female	All ages	2.13	1.82	2.47
Cuba	Both	80-84	10.99	9.00	13.45	Cuba	Male	80-84	9.06	7.33	11.11	Cuba	Female	80-84	12.55	10.26	15.41
Cuba	Both	85-89	12.62	10.29	15.23	Cuba	Male	85-89	10.52	8.57	12.64	Cuba	Female	85-89	14.22	11.61	17.28

Cuba	Both	90-94	13.92	11.53	16.66	Cuba	Male	90-94	11.78	9.61	14.09	Cuba	Female	90-94	15.41	12.67	18.43
Cyprus	Both	40-44 years	1.23	0.94	1.60	Cyprus	Male	40-44 years	0.62	0.45	0.83	Cyprus	Female	40-44 years	1.74	1.32	2.31
Cyprus	Both	45-49 years	2.11	1.66	2.60	Cyprus	Male	45-49 years	1.30	1.00	1.64	Cyprus	Female	45-49 years	2.78	2.18	3.47
Cyprus	Both	50-54 years	3.08	2.39	3.85	Cyprus	Male	50-54 years	2.32	1.77	2.99	Cyprus	Female	50-54 years	3.76	2.92	4.68
Cyprus	Both	55-59 years	4.44	3.53	5.40	Cyprus	Male	55-59 years	3.87	3.04	4.74	Cyprus	Female	55-59 years	4.98	3.96	6.07
Cyprus	Both	60-64 years	6.41	5.15	7.93	Cyprus	Male	60-64 years	5.97	4.68	7.49	Cyprus	Female	60-64 years	6.85	5.48	8.50
Cyprus	Both	65-69 years	9.98	8.04	12.10	Cyprus	Male	65-69 years	9.12	7.31	11.15	Cyprus	Female	65-69 years	10.83	8.70	13.12
Cyprus	Both	70-74 years	14.61	11.32	18.24	Cyprus	Male	70-74 years	12.95	9.92	16.18	Cyprus	Female	70-74 years	16.21	12.55	20.48
Cyprus	Both	75-79 years	18.95	15.07	23.26	Cyprus	Male	75-79 years	16.40	12.93	20.22	Cyprus	Female	75-79 years	21.21	17.02	26.06
Cyprus	Both	All ages	3.41	2.94	3.90	Cyprus	Male	All ages	2.83	2.40	3.26	Cyprus	Female	All ages	3.95	3.40	4.55
Cyprus	Both	80-84	22.76	18.60	27.32	Cyprus	Male	80-84	19.27	15.57	23.19	Cyprus	Female	80-84	25.51	20.92	30.80
Cyprus	Both	85-89	25.62	21.27	30.38	Cyprus	Male	85-89	21.21	17.33	25.35	Cyprus	Female	85-89	28.49	23.72	33.99
Cyprus	Both	90-94	27.65	23.41	32.44	Cyprus	Male	90-94	22.25	18.46	26.42	Cyprus	Female	90-94	30.16	25.48	35.48
Czechia	Both	60-64 years	4.17	3.29	5.21	Czechia	Male	40-44 years	0.43	0.31	0.59	Czechia	Female	60-64 years	4.65	3.63	5.82
Czechia	Both	65-69 years	6.27	5.10	7.63	Czechia	Male	45-49 years	0.90	0.70	1.13	Czechia	Female	65-69 years	6.99	5.68	8.56
Czechia	Both	70-74 years	8.95	6.99	11.26	Czechia	Male	50-54 years	1.57	1.21	2.02	Czechia	Female	70-74 years	9.93	7.76	12.54
Czechia	Both	75-79 years	11.70	9.30	14.34	Czechia	Male	55-59 years	2.49	1.97	3.06	Czechia	Female	75-79 years	12.84	10.17	15.88
Czechia	Both	All ages	2.81	2.41	3.25	Czechia	Male	60-64 years	3.66	2.89	4.58	Czechia	Female	All ages	3.43	2.93	3.99
Czechia	Both	80-84	14.36	11.70	17.49	Czechia	Male	65-69 years	5.44	4.33	6.65	Czechia	Female	80-84	15.53	12.59	19.01
Czechia	Both	85-89	16.59	13.72	19.94	Czechia	Male	70-74 years	7.70	5.96	9.63	Czechia	Female	85-89	17.64	14.53	21.26
Czechia	Both	90-94	18.38	15.25	21.98	Czechia	Male	75-79 years	10.05	8.07	12.24	Czechia	Female	90-94	19.23	15.92	23.17
Czechia	Both	40-44 years	0.47	0.35	0.63	Czechia	Male	All ages	2.15	1.83	2.49	Czechia	Female	40-44 years	0.51	0.38	0.70
Czechia	Both	45-49 years	0.99	0.77	1.24	Czechia	Male	80-84	12.37	10.07	15.22	Czechia	Female	45-49 years	1.09	0.84	1.37
Czechia	Both	50-54 years	1.76	1.33	2.26	Czechia	Male	85-89	14.42	11.86	17.45	Czechia	Female	50-54 years	1.95	1.46	2.48

Czechia	Both	55-59 years	2.80	2.19	3.46	Czechia	Male	90-94	16.24	13.44	19.47	Czechia	Female	55-59 years	3.12	2.41	3.87
C척te d'Ivoire	Both	40-44 years	0.31	0.22	0.41	C척te d'Ivoire	Male	40-44 years	0.29	0.21	0.38	C척te d'Ivoire	Female	40-44 years	0.33	0.24	0.44
C척te d'Ivoire	Both	45-49 years	0.68	0.52	0.86	C척te d'Ivoire	Male	45-49 years	0.63	0.48	0.81	C척te d'Ivoire	Female	45-49 years	0.74	0.57	0.93
C척te d'Ivoire	Both	50-54 years	1.24	0.95	1.60	C척te d'Ivoire	Male	50-54 years	1.15	0.87	1.49	C척te d'Ivoire	Female	50-54 years	1.35	1.03	1.74
C척te d'Ivoire	Both	55-59 years	2.01	1.56	2.44	C척te d'Ivoire	Male	55-59 years	1.84	1.44	2.26	C척te d'Ivoire	Female	55-59 years	2.19	1.71	2.69
C척te d'Ivoire	Both	60-64 years	2.97	2.32	3.70	C척te d'Ivoire	Male	60-64 years	2.71	2.12	3.40	C척te d'Ivoire	Female	60-64 years	3.26	2.53	4.10
C척te d'Ivoire	Both	65-69 years	4.45	3.59	5.46	C척te d'Ivoire	Male	65-69 years	4.01	3.21	4.95	C척te d'Ivoire	Female	65-69 years	4.95	3.92	6.08
C척te d'Ivoire	Both	70-74 years	6.39	4.91	8.01	C척te d'Ivoire	Male	70-74 years	5.70	4.36	7.09	C척te d'Ivoire	Female	70-74 years	7.15	5.48	9.12
C척te d'Ivoire	Both	75-79 years	8.50	6.76	10.41	C척te d'Ivoire	Male	75-79 years	7.53	6.01	9.27	C척te d'Ivoire	Female	75-79 years	9.46	7.41	11.71
C척te d'Ivoire	Both	All ages	0.35	0.30	0.40	C척te d'Ivoire	Male	All ages	0.32	0.27	0.37	C척te d'Ivoire	Female	All ages	0.38	0.32	0.44
C척te d'Ivoire	Both	80-84	10.62	8.53	13.08	C척te d'Ivoire	Male	80-84	9.38	7.52	11.49	C척te d'Ivoire	Female	80-84	11.71	9.33	14.56
C척te d'Ivoire	Both	85-89	12.49	10.13	15.14	C척te d'Ivoire	Male	85-89	11.02	8.96	13.39	C척te d'Ivoire	Female	85-89	13.55	10.97	16.49
C척te d'Ivoire	Both	90-94	14.11	11.56	17.04	C척te d'Ivoire	Male	90-94	12.46	10.23	14.92	C척te d'Ivoire	Female	90-94	15.02	12.29	18.11
Democratic People's Republic of Korea	Both	40-44 years	0.55	0.41	0.74	Democratic People's Republic of Korea	Male	40-44 years	0.44	0.33	0.60	Democratic People's Republic of Korea	Female	40-44 years	0.68	0.50	0.91
Democratic People's Republic of Korea	Both	45-49 years	1.24	0.96	1.55	Democratic People's Republic of Korea	Male	45-49 years	0.95	0.74	1.18	Democratic People's Republic of Korea	Female	45-49 years	1.55	1.20	1.95
Democratic People's Republic of Korea	Both	50-54 years	2.25	1.71	2.92	Democratic People's Republic of Korea	Male	50-54 years	1.66	1.27	2.14	Democratic People's Republic of Korea	Female	50-54 years	2.82	2.11	3.64
Democratic People's Republic of Korea	Both	55-59 years	3.41	2.68	4.22	Democratic People's Republic of Korea	Male	55-59 years	2.50	1.97	3.07	Democratic People's Republic of Korea	Female	55-59 years	4.27	3.32	5.31

Democratic People's Republic of Korea	Both	60-64 years	4.68	3.66	5.78	Democratic People's Republic of Korea	Male	60-64 years	3.39	2.70	4.18	Democratic People's Republic of Korea	Female	60-64 years	5.81	4.46	7.19
Democratic People's Republic of Korea	Both	65-69 years	6.48	5.26	7.85	Democratic People's Republic of Korea	Male	65-69 years	4.54	3.66	5.56	Democratic People's Republic of Korea	Female	65-69 years	7.95	6.39	9.70
Democratic People's Republic of Korea	Both	70-74 years	8.79	6.91	10.97	Democratic People's Republic of Korea	Male	70-74 years	5.90	4.64	7.33	Democratic People's Republic of Korea	Female	70-74 years	10.53	8.18	13.21
Democratic People's Republic of Korea	Both	75-79 years	11.26	9.12	13.63	Democratic People's Republic of Korea	Male	75-79 years	7.20	5.82	8.75	Democratic People's Republic of Korea	Female	75-79 years	13.04	10.49	15.79
Democratic People's Republic of Korea	Both	All ages	1.77	1.52	2.03	Democratic People's Republic of Korea	Male	All ages	1.00	0.85	1.15	Democratic People's Republic of Korea	Female	All ages	2.51	2.15	2.89
Democratic People's Republic of Korea	Both	80-84	13.49	11.05	16.32	Democratic People's Republic of Korea	Male	80-84	8.36	6.79	10.13	Democratic People's Republic of Korea	Female	80-84	15.23	12.38	18.43
Democratic People's Republic of Korea	Both	85-89	15.28	12.52	18.26	Democratic People's Republic of Korea	Male	85-89	9.24	7.63	11.12	Democratic People's Republic of Korea	Female	85-89	16.58	13.55	19.81
Democratic People's Republic of Korea	Both	90-94	16.10	13.39	19.11	Democratic People's Republic of Korea	Male	90-94	9.92	8.23	11.98	Democratic People's Republic of Korea	Female	90-94	17.20	14.27	20.47
Democratic Republic of the Congo	Both	40-44 years	0.32	0.23	0.42	Democratic Republic of the Congo	Male	40-44 years	0.27	0.19	0.37	Democratic Republic of the Congo	Female	40-44 years	0.36	0.26	0.48
Democratic Republic of the Congo	Both	45-49 years	0.69	0.53	0.88	Democratic Republic of the Congo	Male	45-49 years	0.58	0.45	0.75	Democratic Republic of the Congo	Female	45-49 years	0.81	0.62	1.02
Democratic Republic	Both	50-54 years	1.26	0.95	1.64	Democratic Republic	Male	50-54 years	1.04	0.79	1.36	Democratic Republic	Female	50-54 years	1.48	1.11	1.92

of the Congo						of the Congo						of the Congo					
Democratic Republic of the Congo	Both	55-59 years	2.05	1.60	2.50	Democratic Republic of the Congo	Male	55-59 years	1.67	1.30	2.02	Democratic Republic of the Congo	Female	55-59 years	2.41	1.86	3.00
Democratic Republic of the Congo	Both	60-64 years	3.06	2.42	3.80	Democratic Republic of the Congo	Male	60-64 years	2.45	1.92	3.03	Democratic Republic of the Congo	Female	60-64 years	3.61	2.83	4.54
Democratic Republic of the Congo	Both	65-69 years	4.69	3.75	5.75	Democratic Republic of the Congo	Male	65-69 years	3.64	2.90	4.42	Democratic Republic of the Congo	Female	65-69 years	5.54	4.40	6.82
Democratic Republic of the Congo	Both	70-74 years	6.85	5.19	8.64	Democratic Republic of the Congo	Male	70-74 years	5.16	3.95	6.49	Democratic Republic of the Congo	Female	70-74 years	8.03	6.12	10.15
Democratic Republic of the Congo	Both	75-79 years	9.15	7.27	11.15	Democratic Republic of the Congo	Male	75-79 years	6.79	5.42	8.25	Democratic Republic of the Congo	Female	75-79 years	10.57	8.35	12.98
Democratic Republic of the Congo	Both	All ages	0.36	0.31	0.41	Democratic Republic of the Congo	Male	All ages	0.25	0.22	0.29	Democratic Republic of the Congo	Female	All ages	0.47	0.41	0.54
Democratic Republic of the Congo	Both	80-84	11.36	9.19	13.83	Democratic Republic of the Congo	Male	80-84	8.41	6.78	10.27	Democratic Republic of the Congo	Female	80-84	12.93	10.41	15.80
Democratic Republic of the Congo	Both	85-89	13.15	10.79	15.77	Democratic Republic of the Congo	Male	85-89	9.81	7.98	11.91	Democratic Republic of the Congo	Female	85-89	14.66	11.99	17.63
Democratic Republic of the Congo	Both	90-94	14.54	11.99	17.48	Democratic Republic of the Congo	Male	90-94	11.05	9.06	13.36	Democratic Republic of the Congo	Female	90-94	15.85	13.03	19.06
Denmark	Both	80-84	27.84	23.05	33.22	Denmark	Male	40-44 years	0.77	0.56	1.03	Denmark	Female	40-44 years	2.22	1.66	2.90
Denmark	Both	85-89	30.92	26.00	36.67	Denmark	Male	45-49 years	1.61	1.23	2.03	Denmark	Female	45-49 years	3.48	2.76	4.34

Denmark	Both	90-94	32.82	27.72	38.18	Denmark	Male	50-54 years	2.89	2.19	3.69	Denmark	Female	50-54 years	4.60	3.61	5.70
Denmark	Both	40-44 years	1.50	1.12	1.95	Denmark	Male	55-59 years	4.82	3.75	5.89	Denmark	Female	55-59 years	5.95	4.69	7.32
Denmark	Both	45-49 years	2.55	2.03	3.14	Denmark	Male	60-64 years	7.45	5.76	9.29	Denmark	Female	60-64 years	8.12	6.47	9.89
Denmark	Both	50-54 years	3.75	2.92	4.65	Denmark	Male	65-69 years	11.53	9.28	14.01	Denmark	Female	65-69 years	13.08	10.57	15.69
Denmark	Both	55-59 years	5.39	4.28	6.50	Denmark	Male	70-74 years	16.50	12.96	21.05	Denmark	Female	70-74 years	19.84	15.19	25.17
Denmark	Both	60-64 years	7.79	6.17	9.51	Denmark	Male	75-79 years	20.77	16.55	25.82	Denmark	Female	75-79 years	25.84	20.53	31.58
Denmark	Both	65-69 years	12.32	10.01	14.77	Denmark	Male	All ages	4.68	3.98	5.45	Denmark	Female	All ages	6.60	5.69	7.58
Denmark	Both	70-74 years	18.23	14.08	22.94	Denmark	Male	80-84	24.11	19.82	29.03	Denmark	Female	80-84	30.70	25.33	36.80
Denmark	Both	75-79 years	23.48	18.61	28.73	Denmark	Male	85-89	26.18	21.82	31.59	Denmark	Female	85-89	33.86	28.52	40.20
Denmark	Both	All ages	5.67	4.89	6.52	Denmark	Male	90-94	27.09	22.84	32.22	Denmark	Female	90-94	35.41	30.00	41.38
Djibouti	Both	40-44 years	0.31	0.22	0.42	Djibouti	Male	80-84	9.04	7.35	11.22	Djibouti	Female	40-44 years	0.35	0.25	0.46
Djibouti	Both	45-49 years	0.68	0.52	0.87	Djibouti	Male	85-89	10.63	8.77	12.95	Djibouti	Female	45-49 years	0.77	0.59	0.97
Djibouti	Both	50-54 years	1.24	0.94	1.58	Djibouti	Male	90-94	12.07	9.96	14.61	Djibouti	Female	50-54 years	1.41	1.06	1.80
Djibouti	Both	55-59 years	2.00	1.56	2.45	Djibouti	Male	40-44 years	0.28	0.20	0.38	Djibouti	Female	55-59 years	2.30	1.80	2.84
Djibouti	Both	60-64 years	2.98	2.35	3.74	Djibouti	Male	45-49 years	0.61	0.47	0.79	Djibouti	Female	60-64 years	3.46	2.67	4.33
Djibouti	Both	65-69 years	4.49	3.61	5.55	Djibouti	Male	50-54 years	1.11	0.84	1.43	Djibouti	Female	65-69 years	5.24	4.17	6.46
Djibouti	Both	70-74 years	6.43	4.92	8.10	Djibouti	Male	55-59 years	1.78	1.39	2.18	Djibouti	Female	70-74 years	7.53	5.77	9.55
Djibouti	Both	75-79 years	8.60	6.86	10.53	Djibouti	Male	60-64 years	2.63	2.08	3.32	Djibouti	Female	75-79 years	9.94	7.83	12.22
Djibouti	Both	All ages	0.44	0.37	0.50	Djibouti	Male	65-69 years	3.90	3.11	4.86	Djibouti	Female	All ages	0.49	0.41	0.56
Djibouti	Both	80-84	10.77	8.69	13.29	Djibouti	Male	70-74 years	5.53	4.26	6.96	Djibouti	Female	80-84	12.28	9.84	15.12
Djibouti	Both	85-89	12.68	10.43	15.31	Djibouti	Male	75-79 years	7.28	5.79	8.94	Djibouti	Female	85-89	14.19	11.58	17.18
Djibouti	Both	90-94	14.38	11.93	17.15	Djibouti	Male	All ages	0.39	0.34	0.46	Djibouti	Female	90-94	15.69	12.98	18.72
Dominic a	Both	40-44 years	0.31	0.22	0.41	Dominic a	Male	80-84	8.59	6.87	10.71	Dominic a	Female	80-84	11.89	9.65	14.65
Dominic a	Both	45-49 years	0.64	0.50	0.81	Dominic a	Male	85-89	10.06	8.13	12.21	Dominic a	Female	85-89	13.57	11.15	16.60

Dominica	Both	50-54 years	1.13	0.87	1.44	Dominica	Male	90-94	11.38	9.36	13.68	Dominica	Female	90-94	14.82	12.28	17.86
Dominica	Both	55-59 years	1.81	1.42	2.21	Dominica	Male	40-44 years	0.26	0.19	0.36	Dominica	Female	40-44 years	0.35	0.26	0.48
Dominica	Both	60-64 years	2.71	2.14	3.38	Dominica	Male	45-49 years	0.54	0.42	0.70	Dominica	Female	45-49 years	0.74	0.57	0.95
Dominica	Both	65-69 years	4.26	3.40	5.24	Dominica	Male	50-54 years	0.95	0.73	1.23	Dominica	Female	50-54 years	1.32	1.00	1.70
Dominica	Both	70-74 years	6.33	4.87	8.01	Dominica	Male	55-59 years	1.52	1.20	1.85	Dominica	Female	55-59 years	2.13	1.65	2.63
Dominica	Both	75-79 years	8.38	6.68	10.30	Dominica	Male	60-64 years	2.28	1.79	2.84	Dominica	Female	60-64 years	3.22	2.54	4.04
Dominica	Both	All ages	1.28	1.10	1.47	Dominica	Male	65-69 years	3.54	2.82	4.38	Dominica	Female	65-69 years	5.02	3.97	6.17
Dominica	Both	80-84	10.41	8.44	12.85	Dominica	Male	70-74 years	5.20	3.98	6.63	Dominica	Female	70-74 years	7.35	5.64	9.30
Dominica	Both	85-89	12.27	10.07	14.93	Dominica	Male	75-79 years	6.92	5.52	8.58	Dominica	Female	75-79 years	9.70	7.70	12.01
Dominica	Both	90-94	13.71	11.29	16.47	Dominica	Male	All ages	1.00	0.86	1.16	Dominica	Female	All ages	1.57	1.35	1.81
Dominican Republic	Both	55-59 years	1.96	1.53	2.38	Dominican Republic	Male	40-44 years	0.28	0.20	0.37	Dominican Republic	Female	40-44 years	0.37	0.27	0.51
Dominican Republic	Both	60-64 years	2.97	2.36	3.71	Dominican Republic	Male	45-49 years	0.58	0.44	0.73	Dominican Republic	Female	45-49 years	0.79	0.60	1.01
Dominican Republic	Both	65-69 years	4.66	3.74	5.70	Dominican Republic	Male	50-54 years	1.02	0.77	1.31	Dominican Republic	Female	50-54 years	1.41	1.06	1.81
Dominican Republic	Both	70-74 years	6.88	5.25	8.69	Dominican Republic	Male	55-59 years	1.63	1.26	1.98	Dominican Republic	Female	55-59 years	2.28	1.78	2.84
Dominican Republic	Both	75-79 years	9.14	7.32	11.26	Dominican Republic	Male	60-64 years	2.45	1.90	3.07	Dominican Republic	Female	60-64 years	3.48	2.76	4.35
Dominican Republic	Both	All ages	0.87	0.75	1.00	Dominican Republic	Male	65-69 years	3.83	3.05	4.73	Dominican Republic	Female	65-69 years	5.44	4.33	6.64
Dominican Republic	Both	80-84	11.27	9.12	13.75	Dominican Republic	Male	70-74 years	5.64	4.40	7.18	Dominican Republic	Female	70-74 years	7.99	6.05	10.26
Dominican Republic	Both	85-89	12.96	10.65	15.56	Dominican Republic	Male	75-79 years	7.50	6.04	9.22	Dominican Republic	Female	75-79 years	10.55	8.40	13.15
Dominican Republic	Both	90-94	14.27	11.77	16.95	Dominican Republic	Male	All ages	0.68	0.59	0.79	Dominican Republic	Female	All ages	1.05	0.91	1.20
Dominican Republic	Both	40-44 years	0.32	0.23	0.44	Dominican Republic	Male	80-84	9.28	7.48	11.49	Dominican Republic	Female	80-84	12.89	10.45	15.74

Dominican Republic	Both	45-49 years	0.68	0.52	0.86	Dominican Republic	Male	85-89	10.82	8.88	13.14	Dominican Republic	Female	85-89	14.67	11.93	17.64
Dominican Republic	Both	50-54 years	1.21	0.92	1.54	Dominican Republic	Male	90-94	12.16	10.00	14.55	Dominican Republic	Female	90-94	15.95	13.14	18.90
Ecuador	Both	40-44 years	0.29	0.21	0.40	Ecuador	Male	40-44 years	0.25	0.18	0.34	Ecuador	Female	40-44 years	0.32	0.23	0.44
Ecuador	Both	45-49 years	0.60	0.46	0.76	Ecuador	Male	45-49 years	0.52	0.40	0.65	Ecuador	Female	45-49 years	0.68	0.52	0.86
Ecuador	Both	50-54 years	1.06	0.80	1.36	Ecuador	Male	50-54 years	0.90	0.69	1.14	Ecuador	Female	50-54 years	1.20	0.90	1.57
Ecuador	Both	55-59 years	1.70	1.31	2.12	Ecuador	Male	55-59 years	1.44	1.13	1.79	Ecuador	Female	55-59 years	1.95	1.49	2.43
Ecuador	Both	60-64 years	2.57	2.00	3.20	Ecuador	Male	60-64 years	2.17	1.69	2.73	Ecuador	Female	60-64 years	2.96	2.29	3.71
Ecuador	Both	65-69 years	3.99	3.21	4.98	Ecuador	Male	65-69 years	3.35	2.69	4.18	Ecuador	Female	65-69 years	4.59	3.66	5.67
Ecuador	Both	70-74 years	5.83	4.51	7.34	Ecuador	Male	70-74 years	4.90	3.77	6.23	Ecuador	Female	70-74 years	6.70	5.11	8.46
Ecuador	Both	75-79 years	7.74	6.19	9.52	Ecuador	Male	75-79 years	6.52	5.14	8.02	Ecuador	Female	75-79 years	8.85	7.12	11.08
Ecuador	Both	All ages	0.76	0.66	0.88	Ecuador	Male	All ages	0.62	0.53	0.72	Ecuador	Female	All ages	0.90	0.76	1.03
Ecuador	Both	80-84	9.60	7.76	11.79	Ecuador	Male	80-84	8.10	6.50	10.04	Ecuador	Female	80-84	10.87	8.77	13.34
Ecuador	Both	85-89	11.25	9.17	13.52	Ecuador	Male	85-89	9.53	7.73	11.61	Ecuador	Female	85-89	12.53	10.15	15.14
Ecuador	Both	90-94	12.62	10.34	15.18	Ecuador	Male	90-94	10.87	8.85	13.20	Ecuador	Female	90-94	13.89	11.49	16.70
Egypt	Both	40-44 years	0.40	0.29	0.54	Egypt	Male	40-44 years	0.36	0.26	0.48	Egypt	Female	40-44 years	0.45	0.33	0.62
Egypt	Both	45-49 years	0.85	0.66	1.07	Egypt	Male	45-49 years	0.75	0.58	0.96	Egypt	Female	45-49 years	0.96	0.74	1.21
Egypt	Both	50-54 years	1.51	1.15	1.92	Egypt	Male	50-54 years	1.32	1.00	1.69	Egypt	Female	50-54 years	1.71	1.30	2.19
Egypt	Both	55-59 years	2.36	1.85	2.93	Egypt	Male	55-59 years	2.06	1.63	2.58	Egypt	Female	55-59 years	2.69	2.08	3.33
Egypt	Both	60-64 years	3.42	2.66	4.22	Egypt	Male	60-64 years	2.98	2.35	3.73	Egypt	Female	60-64 years	3.92	3.01	4.87
Egypt	Both	65-69 years	5.09	4.07	6.29	Egypt	Male	65-69 years	4.43	3.56	5.46	Egypt	Female	65-69 years	5.87	4.67	7.29
Egypt	Both	70-74 years	7.24	5.52	9.23	Egypt	Male	70-74 years	6.32	4.84	8.04	Egypt	Female	70-74 years	8.39	6.43	10.81
Egypt	Both	75-79 years	9.44	7.45	11.63	Egypt	Male	75-79 years	8.29	6.55	10.31	Egypt	Female	75-79 years	10.96	8.63	13.52
Egypt	Both	All ages	0.67	0.57	0.78	Egypt	Male	All ages	0.64	0.54	0.74	Egypt	Female	All ages	0.71	0.60	0.82
Egypt	Both	80-84	11.49	9.37	14.13	Egypt	Male	80-84	10.21	8.22	12.56	Egypt	Female	80-84	13.38	10.84	16.52

Egypt	Both	85-89	12.97	10.66	15.70	Egypt	Male	85-89	11.91	9.68	14.50	Egypt	Female	85-89	15.28	12.54	18.52
Egypt	Both	90-94	14.05	11.61	17.06	Egypt	Male	90-94	13.42	11.05	16.45	Egypt	Female	90-94	16.69	13.73	19.83
El Salvador	Both	40-44 years	0.31	0.22	0.43	El Salvador	Male	85-89	10.38	8.51	12.73	El Salvador	Female	40-44 years	0.35	0.25	0.48
El Salvador	Both	45-49 years	0.65	0.50	0.82	El Salvador	Male	90-94	11.52	9.48	13.89	El Salvador	Female	45-49 years	0.74	0.56	0.93
El Salvador	Both	50-54 years	1.14	0.86	1.46	El Salvador	Male	40-44 years	0.26	0.19	0.36	El Salvador	Female	50-54 years	1.30	0.97	1.68
El Salvador	Both	55-59 years	1.83	1.44	2.26	El Salvador	Male	45-49 years	0.54	0.41	0.67	El Salvador	Female	55-59 years	2.09	1.62	2.59
El Salvador	Both	60-64 years	2.79	2.20	3.48	El Salvador	Male	50-54 years	0.93	0.70	1.17	El Salvador	Female	60-64 years	3.19	2.52	3.99
El Salvador	Both	65-69 years	4.48	3.53	5.54	El Salvador	Male	55-59 years	1.48	1.17	1.80	El Salvador	Female	65-69 years	5.13	4.05	6.33
El Salvador	Both	70-74 years	6.73	5.11	8.47	El Salvador	Male	60-64 years	2.24	1.75	2.80	El Salvador	Female	70-74 years	7.69	5.85	9.83
El Salvador	Both	75-79 years	8.98	7.10	11.07	El Salvador	Male	65-69 years	3.61	2.85	4.48	El Salvador	Female	75-79 years	10.22	8.03	12.65
El Salvador	Both	All ages	0.99	0.85	1.13	El Salvador	Male	70-74 years	5.46	4.14	6.95	El Salvador	Female	All ages	1.20	1.03	1.37
El Salvador	Both	80-84	11.01	8.93	13.63	El Salvador	Male	75-79 years	7.31	5.79	8.98	El Salvador	Female	80-84	12.48	10.06	15.44
El Salvador	Both	85-89	12.56	10.34	15.23	El Salvador	Male	All ages	0.74	0.63	0.85	El Salvador	Female	85-89	14.07	11.49	17.16
El Salvador	Both	90-94	13.75	11.38	16.58	El Salvador	Male	80-84	9.00	7.31	11.31	El Salvador	Female	90-94	15.10	12.44	18.24
Equatorial Guinea	Both	40-44 years	0.34	0.25	0.46	Equatorial Guinea	Male	40-44 years	0.29	0.21	0.39	Equatorial Guinea	Female	40-44 years	0.39	0.28	0.53
Equatorial Guinea	Both	45-49 years	0.77	0.59	0.98	Equatorial Guinea	Male	45-49 years	0.63	0.49	0.81	Equatorial Guinea	Female	45-49 years	0.89	0.68	1.13
Equatorial Guinea	Both	50-54 years	1.43	1.09	1.83	Equatorial Guinea	Male	50-54 years	1.15	0.87	1.49	Equatorial Guinea	Female	50-54 years	1.65	1.24	2.12
Equatorial Guinea	Both	55-59 years	2.36	1.84	2.90	Equatorial Guinea	Male	55-59 years	1.88	1.45	2.30	Equatorial Guinea	Female	55-59 years	2.70	2.08	3.33
Equatorial Guinea	Both	60-64 years	3.56	2.79	4.37	Equatorial Guinea	Male	60-64 years	2.81	2.21	3.48	Equatorial Guinea	Female	60-64 years	4.09	3.17	5.05
Equatorial Guinea	Both	65-69 years	5.43	4.38	6.55	Equatorial Guinea	Male	65-69 years	4.18	3.39	5.07	Equatorial Guinea	Female	65-69 years	6.31	5.03	7.68
Equatorial Guinea	Both	70-74 years	7.93	6.15	10.01	Equatorial Guinea	Male	70-74 years	5.95	4.65	7.44	Equatorial Guinea	Female	70-74 years	9.24	7.15	11.71
Equatorial Guinea	Both	75-79 years	10.53	8.38	13.00	Equatorial Guinea	Male	75-79 years	7.94	6.36	9.74	Equatorial Guinea	Female	75-79 years	12.32	9.77	15.47
Equatorial Guinea	Both	All ages	0.36	0.31	0.41	Equatorial Guinea	Male	All ages	0.22	0.18	0.25	Equatorial Guinea	Female	All ages	0.53	0.45	0.60
Equatorial Guinea	Both	80-84	13.26	10.76	16.21	Equatorial Guinea	Male	80-84	10.04	8.13	12.37	Equatorial Guinea	Female	80-84	15.35	12.50	18.70

Equatorial Guinea	Both	85-89	15.76	13.07	18.87	Equatorial Guinea	Male	85-89	12.05	9.95	14.50	Equatorial Guinea	Female	85-89	17.91	14.82	21.48
Equatorial Guinea	Both	90-94	18.08	15.11	21.52	Equatorial Guinea	Male	90-94	13.96	11.51	16.58	Equatorial Guinea	Female	90-94	19.98	16.66	23.83
Eritrea	Both	40-44 years	0.29	0.21	0.39	Eritrea	Male	40-44 years	0.26	0.18	0.34	Eritrea	Female	40-44 years	0.32	0.23	0.44
Eritrea	Both	45-49 years	0.63	0.48	0.79	Eritrea	Male	45-49 years	0.56	0.42	0.70	Eritrea	Female	45-49 years	0.70	0.53	0.89
Eritrea	Both	50-54 years	1.14	0.86	1.46	Eritrea	Male	50-54 years	1.00	0.75	1.27	Eritrea	Female	50-54 years	1.27	0.95	1.65
Eritrea	Both	55-59 years	1.85	1.45	2.27	Eritrea	Male	55-59 years	1.60	1.25	1.96	Eritrea	Female	55-59 years	2.06	1.61	2.56
Eritrea	Both	60-64 years	2.76	2.17	3.45	Eritrea	Male	60-64 years	2.34	1.85	2.93	Eritrea	Female	60-64 years	3.07	2.42	3.84
Eritrea	Both	65-69 years	4.17	3.34	5.12	Eritrea	Male	65-69 years	3.45	2.76	4.25	Eritrea	Female	65-69 years	4.65	3.69	5.73
Eritrea	Both	70-74 years	6.02	4.60	7.49	Eritrea	Male	70-74 years	4.87	3.72	6.05	Eritrea	Female	70-74 years	6.68	5.11	8.35
Eritrea	Both	75-79 years	8.02	6.42	9.72	Eritrea	Male	75-79 years	6.43	5.16	7.74	Eritrea	Female	75-79 years	8.84	7.05	10.78
Eritrea	Both	All ages	0.31	0.27	0.36	Eritrea	Male	All ages	0.22	0.19	0.26	Eritrea	Female	All ages	0.41	0.35	0.47
Eritrea	Both	80-84	10.07	8.16	12.42	Eritrea	Male	80-84	8.02	6.52	9.84	Eritrea	Female	80-84	10.95	8.84	13.46
Eritrea	Both	85-89	11.82	9.68	14.26	Eritrea	Male	85-89	9.47	7.71	11.37	Eritrea	Female	85-89	12.66	10.33	15.32
Eritrea	Both	90-94	13.29	10.93	15.81	Eritrea	Male	90-94	10.79	8.87	12.94	Eritrea	Female	90-94	14.00	11.46	16.59
Estonia	Both	40-44 years	0.49	0.36	0.66	Estonia	Male	40-44 years	0.39	0.29	0.52	Estonia	Female	40-44 years	0.59	0.43	0.80
Estonia	Both	45-49 years	1.05	0.81	1.32	Estonia	Male	45-49 years	0.81	0.63	1.03	Estonia	Female	45-49 years	1.29	0.98	1.64
Estonia	Both	50-54 years	1.88	1.42	2.43	Estonia	Male	50-54 years	1.41	1.08	1.80	Estonia	Female	50-54 years	2.32	1.72	3.02
Estonia	Both	55-59 years	3.02	2.35	3.70	Estonia	Male	55-59 years	2.23	1.75	2.75	Estonia	Female	55-59 years	3.72	2.85	4.59
Estonia	Both	60-64 years	4.52	3.53	5.65	Estonia	Male	60-64 years	3.27	2.59	4.09	Estonia	Female	60-64 years	5.53	4.27	6.99
Estonia	Both	65-69 years	6.91	5.59	8.49	Estonia	Male	65-69 years	4.83	3.87	5.90	Estonia	Female	65-69 years	8.37	6.75	10.32
Estonia	Both	70-74 years	10.00	7.80	12.50	Estonia	Male	70-74 years	6.82	5.26	8.61	Estonia	Female	70-74 years	11.94	9.40	15.08
Estonia	Both	75-79 years	13.22	10.60	16.16	Estonia	Male	75-79 years	8.92	7.18	10.94	Estonia	Female	75-79 years	15.39	12.31	18.88
Estonia	Both	All ages	3.24	2.78	3.71	Estonia	Male	All ages	1.75	1.50	2.00	Estonia	Female	All ages	4.52	3.86	5.23
Estonia	Both	80-84	16.31	13.36	19.77	Estonia	Male	80-84	11.01	8.90	13.48	Estonia	Female	80-84	18.45	15.06	22.29

Estonia	Both	85-89	18.73	15.45	22.53	Estonia	Male	85-89	12.92	10.58	15.65	Estonia	Female	85-89	20.64	17.03	24.85
Estonia	Both	90-94	20.61	17.15	24.28	Estonia	Male	90-94	14.66	12.07	17.53	Estonia	Female	90-94	22.04	18.30	26.07
Eswatini	Both	40-44 years	0.40	0.29	0.54	Eswatini	Male	40-44 years	0.34	0.25	0.46	Eswatini	Female	40-44 years	0.46	0.34	0.62
Eswatini	Both	45-49 years	0.90	0.70	1.15	Eswatini	Male	45-49 years	0.75	0.59	0.95	Eswatini	Female	45-49 years	1.05	0.80	1.35
Eswatini	Both	50-54 years	1.68	1.27	2.19	Eswatini	Male	50-54 years	1.38	1.06	1.75	Eswatini	Female	50-54 years	1.95	1.46	2.56
Eswatini	Both	55-59 years	2.76	2.13	3.38	Eswatini	Male	55-59 years	2.22	1.73	2.73	Eswatini	Female	55-59 years	3.16	2.42	3.89
Eswatini	Both	60-64 years	4.12	3.24	5.11	Eswatini	Male	60-64 years	3.27	2.57	4.08	Eswatini	Female	60-64 years	4.73	3.68	5.88
Eswatini	Both	65-69 years	6.32	5.09	7.73	Eswatini	Male	65-69 years	4.89	3.96	6.01	Eswatini	Female	65-69 years	7.26	5.84	8.92
Eswatini	Both	70-74 years	9.32	7.16	11.70	Eswatini	Male	70-74 years	6.99	5.42	8.88	Eswatini	Female	70-74 years	10.57	8.11	13.41
Eswatini	Both	75-79 years	12.49	9.89	15.22	Eswatini	Male	75-79 years	9.22	7.39	11.33	Eswatini	Female	75-79 years	13.93	11.04	17.00
Eswatini	Both	All ages	0.64	0.55	0.74	Eswatini	Male	All ages	0.39	0.34	0.45	Eswatini	Female	All ages	0.87	0.74	1.01
Eswatini	Both	80-84	15.54	12.58	18.84	Eswatini	Male	80-84	11.43	9.16	14.07	Eswatini	Female	80-84	17.05	13.74	20.65
Eswatini	Both	85-89	17.95	14.89	21.51	Eswatini	Male	85-89	13.32	10.93	16.20	Eswatini	Female	85-89	19.35	15.99	23.22
Eswatini	Both	90-94	19.71	16.38	23.40	Eswatini	Male	90-94	14.91	12.28	17.90	Eswatini	Female	90-94	20.87	17.35	24.78
Ethiopia	Both	40-44 years	0.25	0.18	0.33	Ethiopia	Male	40-44 years	0.22	0.16	0.30	Ethiopia	Female	40-44 years	0.27	0.20	0.36
Ethiopia	Both	45-49 years	0.56	0.43	0.71	Ethiopia	Male	45-49 years	0.50	0.38	0.64	Ethiopia	Female	45-49 years	0.62	0.48	0.79
Ethiopia	Both	50-54 years	1.04	0.79	1.33	Ethiopia	Male	50-54 years	0.92	0.70	1.19	Ethiopia	Female	50-54 years	1.17	0.88	1.49
Ethiopia	Both	55-59 years	1.70	1.33	2.07	Ethiopia	Male	55-59 years	1.48	1.17	1.81	Ethiopia	Female	55-59 years	1.91	1.49	2.35
Ethiopia	Both	60-64 years	2.52	1.98	3.14	Ethiopia	Male	60-64 years	2.18	1.72	2.71	Ethiopia	Female	60-64 years	2.86	2.25	3.58
Ethiopia	Both	65-69 years	3.73	2.99	4.60	Ethiopia	Male	65-69 years	3.21	2.58	3.94	Ethiopia	Female	65-69 years	4.29	3.43	5.30
Ethiopia	Both	70-74 years	5.27	4.07	6.60	Ethiopia	Male	70-74 years	4.51	3.50	5.64	Ethiopia	Female	70-74 years	6.11	4.72	7.66
Ethiopia	Both	75-79 years	6.95	5.61	8.46	Ethiopia	Male	75-79 years	5.95	4.79	7.28	Ethiopia	Female	75-79 years	8.06	6.48	9.83
Ethiopia	Both	All ages	0.29	0.25	0.33	Ethiopia	Male	All ages	0.26	0.22	0.29	Ethiopia	Female	All ages	0.32	0.27	0.37
Ethiopia	Both	80-84	8.66	7.07	10.65	Ethiopia	Male	80-84	7.43	5.98	9.10	Ethiopia	Female	80-84	10.00	8.17	12.32

Ethiopia	Both	85-89	10.25	8.40	12.30	Ethiopia	Male	85-89	8.86	7.23	10.65	Ethiopia	Female	85-89	11.68	9.59	14.04
Ethiopia	Both	90-94	11.82	9.75	14.18	Ethiopia	Male	90-94	10.26	8.41	12.37	Ethiopia	Female	90-94	13.16	10.82	15.83
Fiji	Both	80-84	17.37	14.17	21.04	Fiji	Male	40-44 years	0.60	0.44	0.80	Fiji	Female	40-44 years	0.75	0.55	1.00
Fiji	Both	40-44 years	0.67	0.50	0.88	Fiji	Male	45-49 years	1.31	1.00	1.66	Fiji	Female	45-49 years	1.67	1.29	2.12
Fiji	Both	45-49 years	1.48	1.15	1.87	Fiji	Male	50-54 years	2.33	1.76	3.01	Fiji	Female	50-54 years	3.02	2.28	3.94
Fiji	Both	50-54 years	2.67	2.01	3.44	Fiji	Male	55-59 years	3.58	2.79	4.39	Fiji	Female	55-59 years	4.66	3.63	5.73
Fiji	Both	55-59 years	4.11	3.25	5.01	Fiji	Male	60-64 years	5.01	3.91	6.23	Fiji	Female	60-64 years	6.57	5.11	8.11
Fiji	Both	60-64 years	5.81	4.54	7.11	Fiji	Male	65-69 years	6.98	5.64	8.59	Fiji	Female	65-69 years	9.52	7.72	11.55
Fiji	Both	65-69 years	8.33	6.78	10.16	Fiji	Male	70-74 years	9.38	7.35	11.71	Fiji	Female	70-74 years	13.24	10.32	16.73
Fiji	Both	70-74 years	11.47	8.95	14.40	Fiji	Male	75-79 years	11.65	9.34	14.29	Fiji	Female	75-79 years	16.72	13.24	20.65
Fiji	Both	75-79 years	14.58	11.64	17.92	Fiji	Male	All ages	1.11	0.94	1.28	Fiji	Female	All ages	1.70	1.46	1.96
Fiji	Both	All ages	1.40	1.20	1.61	Fiji	Male	80-84	13.66	10.94	16.71	Fiji	Female	80-84	19.67	15.98	23.87
Fiji	Both	85-89	19.35	15.99	23.17	Fiji	Male	85-89	15.14	12.38	18.18	Fiji	Female	85-89	21.55	17.73	25.88
Fiji	Both	90-94	21.07	17.53	24.87	Fiji	Male	90-94	16.18	13.37	19.18	Fiji	Female	90-94	22.52	18.69	26.62
Finland	Both	40-44 years	0.96	0.73	1.27	Finland	Male	65-69 years	7.56	6.13	9.30	Finland	Female	65-69 years	9.22	7.47	11.20
Finland	Both	45-49 years	1.69	1.34	2.11	Finland	Male	70-74 years	10.75	8.23	13.46	Finland	Female	70-74 years	13.73	10.68	17.18
Finland	Both	50-54 years	2.56	1.99	3.25	Finland	Male	75-79 years	13.73	10.94	16.76	Finland	Female	75-79 years	18.08	14.54	22.23
Finland	Both	55-59 years	3.76	2.97	4.53	Finland	Male	All ages	3.33	2.83	3.87	Finland	Female	All ages	5.29	4.56	6.05
Finland	Both	60-64 years	5.44	4.33	6.72	Finland	Male	80-84	16.34	13.18	19.82	Finland	Female	80-84	21.99	18.06	26.48
Finland	Both	65-69 years	8.42	6.90	10.20	Finland	Male	85-89	18.25	15.09	21.93	Finland	Female	85-89	24.91	20.63	29.87
Finland	Both	70-74 years	12.33	9.59	15.39	Finland	Male	90-94	19.47	16.26	23.18	Finland	Female	90-94	26.77	22.27	31.80
Finland	Both	75-79 years	16.16	12.99	19.80	Finland	Male	40-44 years	0.53	0.39	0.72	Finland	Female	40-44 years	1.41	1.06	1.85
Finland	Both	All ages	4.35	3.71	4.99	Finland	Male	45-49 years	1.10	0.84	1.41	Finland	Female	45-49 years	2.29	1.80	2.87
Finland	Both	80-84	19.71	16.11	23.71	Finland	Male	50-54 years	1.96	1.48	2.54	Finland	Female	50-54 years	3.16	2.46	4.00

Finland	Both	85-89	22.57	18.74	26.80	Finland	Male	55-59 years	3.25	2.54	3.99	Finland	Female	55-59 years	4.26	3.37	5.15
Finland	Both	90-94	24.66	20.64	29.24	Finland	Male	60-64 years	4.98	3.90	6.22	Finland	Female	60-64 years	5.89	4.69	7.25
France	Both	40-44 years	0.96	0.72	1.24	France	Male	40-44 years	0.52	0.38	0.69	France	Female	40-44 years	1.37	1.03	1.80
France	Both	45-49 years	1.67	1.30	2.05	France	Male	45-49 years	1.08	0.82	1.36	France	Female	45-49 years	2.23	1.75	2.77
France	Both	50-54 years	2.52	1.96	3.18	France	Male	50-54 years	1.92	1.46	2.46	France	Female	50-54 years	3.09	2.42	3.91
France	Both	55-59 years	3.71	2.93	4.49	France	Male	55-59 years	3.20	2.52	3.94	France	Female	55-59 years	4.18	3.33	5.11
France	Both	60-64 years	5.38	4.28	6.51	France	Male	60-64 years	4.92	3.87	6.15	France	Female	60-64 years	5.81	4.65	6.99
France	Both	65-69 years	8.32	6.76	9.96	France	Male	65-69 years	7.45	5.96	9.07	France	Female	65-69 years	9.09	7.33	10.88
France	Both	70-74 years	12.16	9.45	15.22	France	Male	70-74 years	10.55	8.13	13.14	France	Female	70-74 years	13.55	10.55	17.07
France	Both	75-79 years	15.87	12.67	19.37	France	Male	75-79 years	13.45	10.71	16.37	France	Female	75-79 years	17.82	14.11	21.96
France	Both	All ages	4.04	3.48	4.58	France	Male	All ages	3.07	2.62	3.52	France	Female	All ages	4.91	4.23	5.59
France	Both	80-84	19.31	15.54	23.35	France	Male	80-84	15.98	12.91	19.26	France	Female	80-84	21.59	17.37	26.25
France	Both	85-89	22.11	18.37	26.49	France	Male	85-89	17.86	14.74	21.38	France	Female	85-89	24.42	20.14	29.22
France	Both	90-94	24.18	20.38	28.63	France	Male	90-94	19.07	15.79	22.79	France	Female	90-94	26.25	22.01	31.06
Gabon	Both	40-44 years	0.34	0.25	0.45	Gabon	Male	40-44 years	0.29	0.21	0.40	Gabon	Female	80-84	15.27	12.33	18.66
Gabon	Both	45-49 years	0.77	0.59	0.97	Gabon	Male	45-49 years	0.64	0.47	0.82	Gabon	Female	85-89	17.66	14.52	21.42
Gabon	Both	50-54 years	1.41	1.06	1.82	Gabon	Male	50-54 years	1.16	0.87	1.52	Gabon	Female	90-94	19.50	16.16	23.41
Gabon	Both	55-59 years	2.30	1.80	2.82	Gabon	Male	55-59 years	1.89	1.46	2.33	Gabon	Female	40-44 years	0.39	0.28	0.52
Gabon	Both	60-64 years	3.45	2.76	4.29	Gabon	Male	60-64 years	2.82	2.21	3.54	Gabon	Female	45-49 years	0.89	0.68	1.13
Gabon	Both	65-69 years	5.26	4.24	6.47	Gabon	Male	65-69 years	4.20	3.31	5.18	Gabon	Female	50-54 years	1.65	1.23	2.14
Gabon	Both	70-74 years	7.76	5.99	9.66	Gabon	Male	70-74 years	5.97	4.59	7.50	Gabon	Female	55-59 years	2.72	2.14	3.32
Gabon	Both	75-79 years	10.61	8.40	12.90	Gabon	Male	75-79 years	7.91	6.33	9.60	Gabon	Female	60-64 years	4.13	3.27	5.10
Gabon	Both	All ages	0.64	0.55	0.73	Gabon	Male	All ages	0.48	0.40	0.55	Gabon	Female	65-69 years	6.36	5.11	7.74
Gabon	Both	80-84	13.51	10.91	16.42	Gabon	Male	80-84	9.91	7.99	12.21	Gabon	Female	70-74 years	9.27	7.13	11.57

Gabon	Both	85-89	15.99	13.21	19.26	Gabon	Male	85-89	11.78	9.66	14.26	Gabon	Female	75-79 years	12.32	9.77	14.98
Gabon	Both	90-94	18.05	14.91	21.59	Gabon	Male	90-94	13.55	11.14	16.33	Gabon	Female	All ages	0.79	0.68	0.90
Gambia	Both	40-44 years	0.32	0.23	0.42	Gambia	Male	All ages	0.36	0.31	0.41	Gambia	Female	All ages	0.45	0.39	0.52
Gambia	Both	45-49 years	0.70	0.54	0.90	Gambia	Male	80-84	9.53	7.62	11.82	Gambia	Female	80-84	11.90	9.61	14.52
Gambia	Both	50-54 years	1.28	0.96	1.68	Gambia	Male	85-89	11.09	9.00	13.61	Gambia	Female	85-89	13.65	11.16	16.60
Gambia	Both	55-59 years	2.07	1.60	2.55	Gambia	Male	90-94	12.42	10.18	15.00	Gambia	Female	90-94	14.97	12.28	17.77
Gambia	Both	60-64 years	3.08	2.40	3.85	Gambia	Male	40-44 years	0.30	0.21	0.40	Gambia	Female	40-44 years	0.34	0.24	0.45
Gambia	Both	65-69 years	4.63	3.71	5.71	Gambia	Male	45-49 years	0.65	0.50	0.83	Gambia	Female	45-49 years	0.76	0.58	0.99
Gambia	Both	70-74 years	6.62	5.11	8.32	Gambia	Male	50-54 years	1.18	0.88	1.54	Gambia	Female	50-54 years	1.39	1.05	1.83
Gambia	Both	75-79 years	8.78	7.06	10.71	Gambia	Male	55-59 years	1.90	1.46	2.36	Gambia	Female	55-59 years	2.26	1.74	2.76
Gambia	Both	All ages	0.41	0.35	0.47	Gambia	Male	60-64 years	2.80	2.18	3.51	Gambia	Female	60-64 years	3.37	2.62	4.23
Gambia	Both	80-84	10.91	8.87	13.45	Gambia	Male	65-69 years	4.14	3.29	5.14	Gambia	Female	65-69 years	5.10	4.06	6.26
Gambia	Both	85-89	12.68	10.36	15.40	Gambia	Male	70-74 years	5.86	4.49	7.53	Gambia	Female	70-74 years	7.35	5.64	9.23
Gambia	Both	90-94	14.10	11.62	16.84	Gambia	Male	75-79 years	7.70	6.11	9.50	Gambia	Female	75-79 years	9.68	7.77	11.79
Georgia	Both	40-44 years	0.48	0.35	0.64	Georgia	Male	40-44 years	0.42	0.30	0.58	Georgia	Female	40-44 years	0.53	0.39	0.72
Georgia	Both	45-49 years	1.01	0.79	1.29	Georgia	Male	45-49 years	0.86	0.66	1.09	Georgia	Female	45-49 years	1.15	0.89	1.46
Georgia	Both	50-54 years	1.80	1.36	2.32	Georgia	Male	50-54 years	1.50	1.13	1.91	Georgia	Female	50-54 years	2.06	1.57	2.66
Georgia	Both	55-59 years	2.86	2.23	3.52	Georgia	Male	55-59 years	2.36	1.84	2.85	Georgia	Female	55-59 years	3.28	2.54	4.10
Georgia	Both	60-64 years	4.22	3.30	5.31	Georgia	Male	60-64 years	3.44	2.70	4.28	Georgia	Female	60-64 years	4.84	3.76	6.16
Georgia	Both	65-69 years	6.39	5.10	7.87	Georgia	Male	65-69 years	5.11	4.11	6.28	Georgia	Female	65-69 years	7.30	5.77	9.02
Georgia	Both	70-74 years	9.16	7.08	11.55	Georgia	Male	70-74 years	7.26	5.62	9.25	Georgia	Female	70-74 years	10.41	8.05	13.26
Georgia	Both	75-79 years	11.97	9.46	14.74	Georgia	Male	75-79 years	9.45	7.52	11.63	Georgia	Female	75-79 years	13.40	10.59	16.56
Georgia	Both	All ages	2.41	2.08	2.77	Georgia	Male	All ages	1.59	1.37	1.83	Georgia	Female	All ages	3.14	2.69	3.63
Georgia	Both	80-84	14.51	11.77	17.75	Georgia	Male	80-84	11.54	9.28	14.21	Georgia	Female	80-84	16.04	12.83	19.62

Georgia	Both	85-89	16.52	13.50	19.82	Georgia	Male	85-89	13.31	10.88	16.13	Georgia	Female	85-89	17.95	14.59	21.62
Georgia	Both	90-94	18.13	15.01	21.67	Georgia	Male	90-94	14.83	12.17	17.68	Georgia	Female	90-94	19.21	15.89	22.99
Germany	Both	40-44 years	0.98	0.73	1.27	Germany	Male	40-44 years	0.54	0.39	0.73	Germany	Female	40-44 years	1.42	1.05	1.84
Germany	Both	45-49 years	1.72	1.35	2.12	Germany	Male	45-49 years	1.13	0.87	1.43	Germany	Female	45-49 years	2.30	1.81	2.88
Germany	Both	50-54 years	2.59	2.02	3.28	Germany	Male	50-54 years	2.02	1.53	2.58	Germany	Female	50-54 years	3.17	2.46	4.03
Germany	Both	55-59 years	3.81	3.03	4.63	Germany	Male	55-59 years	3.36	2.65	4.12	Germany	Female	55-59 years	4.25	3.38	5.23
Germany	Both	60-64 years	5.53	4.45	6.77	Germany	Male	60-64 years	5.17	4.04	6.40	Germany	Female	60-64 years	5.88	4.71	7.23
Germany	Both	65-69 years	8.58	6.98	10.31	Germany	Male	65-69 years	7.88	6.38	9.55	Germany	Female	65-69 years	9.23	7.46	11.10
Germany	Both	70-74 years	12.57	9.87	15.82	Germany	Male	70-74 years	11.19	8.67	13.97	Germany	Female	70-74 years	13.79	10.83	17.25
Germany	Both	75-79 years	16.43	13.19	20.12	Germany	Male	75-79 years	14.29	11.31	17.48	Germany	Female	75-79 years	18.17	14.72	22.27
Germany	Both	All ages	4.49	3.88	5.14	Germany	Male	All ages	3.50	2.99	4.03	Germany	Female	All ages	5.43	4.70	6.20
Germany	Both	80-84	19.98	16.48	23.95	Germany	Male	80-84	17.01	13.74	20.76	Germany	Female	80-84	22.11	18.24	26.58
Germany	Both	85-89	22.84	19.06	27.17	Germany	Male	85-89	18.98	15.74	22.91	Germany	Female	85-89	25.11	20.93	29.66
Germany	Both	90-94	25.11	21.11	29.36	Germany	Male	90-94	20.21	16.90	24.03	Germany	Female	90-94	27.10	22.73	31.68
Ghana	Both	40-44 years	0.30	0.22	0.40	Ghana	Male	40-44 years	0.28	0.20	0.37	Ghana	Female	40-44 years	0.32	0.23	0.43
Ghana	Both	45-49 years	0.66	0.51	0.84	Ghana	Male	45-49 years	0.61	0.47	0.77	Ghana	Female	45-49 years	0.71	0.54	0.91
Ghana	Both	50-54 years	1.21	0.91	1.56	Ghana	Male	50-54 years	1.11	0.84	1.41	Ghana	Female	50-54 years	1.30	0.97	1.67
Ghana	Both	55-59 years	1.96	1.53	2.40	Ghana	Male	55-59 years	1.77	1.37	2.19	Ghana	Female	55-59 years	2.11	1.65	2.59
Ghana	Both	60-64 years	2.90	2.27	3.64	Ghana	Male	60-64 years	2.59	2.01	3.27	Ghana	Female	60-64 years	3.15	2.47	3.96
Ghana	Both	65-69 years	4.36	3.51	5.37	Ghana	Male	65-69 years	3.84	3.07	4.76	Ghana	Female	65-69 years	4.78	3.82	5.93
Ghana	Both	70-74 years	6.26	4.86	7.95	Ghana	Male	70-74 years	5.47	4.20	6.91	Ghana	Female	70-74 years	6.88	5.30	8.76
Ghana	Both	75-79 years	8.33	6.62	10.30	Ghana	Male	75-79 years	7.21	5.72	8.86	Ghana	Female	75-79 years	9.09	7.25	11.30
Ghana	Both	All ages	0.44	0.38	0.51	Ghana	Male	All ages	0.36	0.30	0.41	Ghana	Female	All ages	0.53	0.46	0.61
Ghana	Both	80-84	10.38	8.29	12.81	Ghana	Male	80-84	8.97	7.15	11.08	Ghana	Female	80-84	11.26	9.01	13.93

Ghana	Both	85-89	12.20	10.04	14.82	Ghana	Male	85-89	10.58	8.67	12.94	Ghana	Female	85-89	13.07	10.77	15.88
Ghana	Both	90-94	13.82	11.47	16.47	Ghana	Male	90-94	12.05	9.93	14.61	Ghana	Female	90-94	14.55	12.04	17.41
Greece	Both	All ages	4.99	4.30	5.73	Greece	Male	All ages	3.97	3.38	4.62	Greece	Female	40-44 years	1.61	1.20	2.12
Greece	Both	80-84	21.56	17.57	26.10	Greece	Male	80-84	18.00	14.65	21.95	Greece	Female	45-49 years	2.59	2.03	3.26
Greece	Both	85-89	24.07	19.93	28.85	Greece	Male	85-89	19.78	16.28	23.74	Greece	Female	50-54 years	3.53	2.75	4.44
Greece	Both	90-94	25.31	21.27	29.86	Greece	Male	90-94	20.72	17.21	24.80	Greece	Female	55-59 years	4.72	3.74	5.78
Greece	Both	40-44 years	1.11	0.83	1.45	Greece	Male	40-44 years	0.58	0.42	0.79	Greece	Female	60-64 years	6.53	5.25	7.93
Greece	Both	45-49 years	1.92	1.52	2.38	Greece	Male	45-49 years	1.22	0.93	1.55	Greece	Female	65-69 years	10.30	8.33	12.38
Greece	Both	50-54 years	2.88	2.24	3.60	Greece	Male	50-54 years	2.18	1.65	2.78	Greece	Female	70-74 years	15.37	11.89	19.38
Greece	Both	55-59 years	4.20	3.34	5.09	Greece	Male	55-59 years	3.64	2.85	4.54	Greece	Female	75-79 years	20.07	16.16	24.72
Greece	Both	60-64 years	6.09	4.86	7.41	Greece	Male	60-64 years	5.60	4.35	7.09	Greece	Female	All ages	5.93	5.09	6.81
Greece	Both	65-69 years	9.46	7.66	11.40	Greece	Male	65-69 years	8.55	6.83	10.45	Greece	Female	80-84	24.09	19.56	29.04
Greece	Both	70-74 years	13.86	10.77	17.45	Greece	Male	70-74 years	12.12	9.32	15.53	Greece	Female	85-89	26.90	22.30	32.11
Greece	Both	75-79 years	17.95	14.41	21.99	Greece	Male	75-79 years	15.34	12.22	19.03	Greece	Female	90-94	28.49	23.78	33.64
Greenland	Both	40-44 years	1.10	0.82	1.41	Greenland	Male	50-54 years	2.57	1.97	3.29	Greenland	Female	40-44 years	1.58	1.19	2.03
Greenland	Both	45-49 years	1.93	1.54	2.37	Greenland	Male	55-59 years	4.33	3.42	5.28	Greenland	Female	45-49 years	2.50	1.97	3.10
Greenland	Both	50-54 years	2.95	2.31	3.65	Greenland	Male	60-64 years	6.72	5.27	8.48	Greenland	Female	50-54 years	3.37	2.65	4.16
Greenland	Both	55-59 years	4.42	3.51	5.30	Greenland	Male	65-69 years	10.35	8.34	12.71	Greenland	Female	55-59 years	4.51	3.58	5.47
Greenland	Both	60-64 years	6.55	5.23	8.08	Greenland	Male	70-74 years	14.80	11.61	18.77	Greenland	Female	60-64 years	6.32	5.03	7.79
Greenland	Both	65-69 years	10.27	8.33	12.47	Greenland	Male	75-79 years	18.88	15.25	23.52	Greenland	Female	65-69 years	10.17	8.23	12.34
Greenland	Both	70-74 years	15.05	11.78	18.98	Greenland	Male	All ages	2.71	2.33	3.14	Greenland	Female	70-74 years	15.39	11.99	19.31
Greenland	Both	75-79 years	19.68	15.92	24.03	Greenland	Male	80-84	22.50	18.55	27.28	Greenland	Female	75-79 years	20.44	16.54	25.05
Greenland	Both	All ages	2.79	2.42	3.17	Greenland	Male	85-89	25.45	21.16	30.22	Greenland	Female	All ages	2.88	2.50	3.27
Greenland	Both	80-84	24.00	19.95	28.60	Greenland	Male	90-94	27.52	23.13	32.18	Greenland	Female	80-84	25.22	20.81	30.11

Greenland	Both	85-89	27.84	23.29	32.85	Greenland	Male	40-44 years	0.69	0.50	0.91	Greenland	Female	85-89	29.28	24.57	34.47
Greenland	Both	90-94	30.55	25.84	35.36	Greenland	Male	45-49 years	1.43	1.11	1.80	Greenland	Female	90-94	32.27	27.37	37.51
Grenada	Both	40-44 years	0.33	0.24	0.44	Grenada	Male	40-44 years	0.28	0.20	0.37	Grenada	Female	40-44 years	0.38	0.27	0.51
Grenada	Both	45-49 years	0.68	0.52	0.87	Grenada	Male	45-49 years	0.58	0.45	0.73	Grenada	Female	45-49 years	0.79	0.60	1.01
Grenada	Both	50-54 years	1.20	0.91	1.54	Grenada	Male	50-54 years	1.01	0.77	1.30	Grenada	Female	50-54 years	1.40	1.05	1.80
Grenada	Both	55-59 years	1.93	1.50	2.35	Grenada	Male	55-59 years	1.62	1.26	1.99	Grenada	Female	55-59 years	2.26	1.75	2.79
Grenada	Both	60-64 years	2.90	2.30	3.61	Grenada	Male	60-64 years	2.43	1.91	3.03	Grenada	Female	60-64 years	3.41	2.68	4.27
Grenada	Both	65-69 years	4.56	3.64	5.59	Grenada	Male	65-69 years	3.79	3.01	4.66	Grenada	Female	65-69 years	5.33	4.24	6.55
Grenada	Both	70-74 years	6.76	5.19	8.53	Grenada	Male	70-74 years	5.58	4.26	7.02	Grenada	Female	70-74 years	7.84	6.02	10.05
Grenada	Both	75-79 years	9.07	7.19	11.13	Grenada	Male	75-79 years	7.43	5.91	9.15	Grenada	Female	75-79 years	10.38	8.24	12.86
Grenada	Both	All ages	1.08	0.93	1.24	Grenada	Male	All ages	0.81	0.68	0.93	Grenada	Female	All ages	1.36	1.17	1.57
Grenada	Both	80-84	11.53	9.42	14.19	Grenada	Male	80-84	9.23	7.43	11.32	Grenada	Female	80-84	12.75	10.35	15.81
Grenada	Both	85-89	13.70	11.34	16.71	Grenada	Male	85-89	10.80	8.83	13.18	Grenada	Female	85-89	14.58	12.01	17.75
Grenada	Both	90-94	15.34	12.66	18.38	Grenada	Male	90-94	12.18	9.92	14.70	Grenada	Female	90-94	15.90	13.12	19.08
Guam	Both	40-44 years	0.54	0.40	0.72	Guam	Male	40-44 years	0.48	0.36	0.66	Guam	Female	40-44 years	0.59	0.44	0.79
Guam	Both	45-49 years	1.16	0.90	1.44	Guam	Male	45-49 years	1.03	0.80	1.29	Guam	Female	45-49 years	1.30	1.01	1.62
Guam	Both	50-54 years	2.06	1.57	2.62	Guam	Male	50-54 years	1.81	1.38	2.31	Guam	Female	50-54 years	2.35	1.78	2.97
Guam	Both	55-59 years	3.25	2.55	3.93	Guam	Male	55-59 years	2.84	2.20	3.45	Guam	Female	55-59 years	3.70	2.91	4.54
Guam	Both	60-64 years	4.70	3.74	5.75	Guam	Male	60-64 years	4.06	3.20	4.98	Guam	Female	60-64 years	5.37	4.25	6.58
Guam	Both	65-69 years	6.76	5.53	8.17	Guam	Male	65-69 years	5.67	4.58	6.89	Guam	Female	65-69 years	7.76	6.31	9.44
Guam	Both	70-74 years	9.15	7.20	11.27	Guam	Male	70-74 years	7.59	5.91	9.36	Guam	Female	70-74 years	10.72	8.53	13.18
Guam	Both	75-79 years	11.83	9.63	14.40	Guam	Male	75-79 years	9.57	7.72	11.61	Guam	Female	75-79 years	13.70	11.10	16.77
Guam	Both	All ages	1.67	1.43	1.90	Guam	Male	All ages	1.31	1.11	1.51	Guam	Female	All ages	2.04	1.76	2.33
Guam	Both	80-84	14.46	11.95	17.65	Guam	Male	80-84	11.50	9.39	14.05	Guam	Female	80-84	16.51	13.64	20.15

Guam	Both	85-89	16.65	13.93	20.04	Guam	Male	85-89	13.20	10.96	15.88	Guam	Female	85-89	18.75	15.72	22.64
Guam	Both	90-94	18.30	15.36	21.74	Guam	Male	90-94	14.72	12.17	17.53	Guam	Female	90-94	20.49	17.25	24.35
Guatemal a	Both	70-74 years	6.74	5.18	8.46	Guatemal a	Male	40-44 years	0.27	0.19	0.36	Guatemal a	Female	70-74 years	7.78	5.89	9.95
Guatemal a	Both	75-79 years	9.00	7.19	10.85	Guatemal a	Male	45-49 years	0.55	0.42	0.69	Guatemal a	Female	75-79 years	10.37	8.20	12.72
Guatemal a	Both	All ages	0.61	0.53	0.70	Guatemal a	Male	50-54 years	0.95	0.72	1.22	Guatemal a	Female	All ages	0.74	0.63	0.85
Guatemal a	Both	80-84	11.09	9.02	13.53	Guatemal a	Male	55-59 years	1.51	1.17	1.85	Guatemal a	Female	80-84	12.69	10.25	15.58
Guatemal a	Both	85-89	12.79	10.53	15.48	Guatemal a	Male	60-64 years	2.29	1.80	2.84	Guatemal a	Female	85-89	14.33	11.80	17.53
Guatemal a	Both	90-94	14.29	11.89	17.10	Guatemal a	Male	65-69 years	3.67	2.94	4.48	Guatemal a	Female	90-94	15.42	12.84	18.53
Guatemal a	Both	40-44 years	0.32	0.23	0.43	Guatemal a	Male	70-74 years	5.52	4.27	6.85	Guatemal a	Female	40-44 years	0.36	0.26	0.48
Guatemal a	Both	45-49 years	0.66	0.51	0.83	Guatemal a	Male	75-79 years	7.39	5.95	8.98	Guatemal a	Female	45-49 years	0.75	0.58	0.95
Guatemal a	Both	50-54 years	1.15	0.87	1.48	Guatemal a	Male	All ages	0.47	0.40	0.54	Guatemal a	Female	50-54 years	1.32	0.99	1.69
Guatemal a	Both	55-59 years	1.84	1.43	2.24	Guatemal a	Male	80-84	9.13	7.44	11.24	Guatemal a	Female	55-59 years	2.11	1.63	2.58
Guatemal a	Both	60-64 years	2.78	2.19	3.45	Guatemal a	Male	85-89	10.54	8.56	12.74	Guatemal a	Female	60-64 years	3.20	2.52	4.04
Guatemal a	Both	65-69 years	4.47	3.56	5.50	Guatemal a	Male	90-94	11.71	9.55	14.07	Guatemal a	Female	65-69 years	5.16	4.09	6.38
Guinea	Both	40-44 years	0.28	0.20	0.37	Guinea	Male	40-44 years	0.26	0.19	0.35	Guinea	Female	40-44 years	0.30	0.22	0.40
Guinea	Both	45-49 years	0.61	0.47	0.78	Guinea	Male	45-49 years	0.56	0.43	0.73	Guinea	Female	45-49 years	0.66	0.50	0.84
Guinea	Both	50-54 years	1.10	0.84	1.41	Guinea	Male	50-54 years	1.01	0.77	1.30	Guinea	Female	50-54 years	1.19	0.89	1.52
Guinea	Both	55-59 years	1.77	1.39	2.17	Guinea	Male	55-59 years	1.62	1.27	1.99	Guinea	Female	55-59 years	1.93	1.51	2.38
Guinea	Both	60-64 years	2.60	2.04	3.23	Guinea	Male	60-64 years	2.37	1.86	2.94	Guinea	Female	60-64 years	2.86	2.24	3.54
Guinea	Both	65-69 years	3.88	3.10	4.76	Guinea	Male	65-69 years	3.50	2.80	4.31	Guinea	Female	65-69 years	4.32	3.42	5.36
Guinea	Both	70-74 years	5.55	4.29	6.94	Guinea	Male	70-74 years	4.95	3.80	6.20	Guinea	Female	70-74 years	6.21	4.77	7.89
Guinea	Both	75-79 years	7.35	5.88	8.91	Guinea	Male	75-79 years	6.53	5.23	7.91	Guinea	Female	75-79 years	8.22	6.53	10.04
Guinea	Both	All ages	0.35	0.30	0.40	Guinea	Male	All ages	0.33	0.28	0.38	Guinea	Female	All ages	0.37	0.31	0.42
Guinea	Both	80-84	9.17	7.41	11.31	Guinea	Male	80-84	8.14	6.55	10.07	Guinea	Female	80-84	10.18	8.14	12.62

Guinea	Both	85-89	10.89	8.91	13.12	Guinea	Male	85-89	9.59	7.79	11.60	Guinea	Female	85-89	11.84	9.66	14.29
Guinea	Both	90-94	12.35	10.21	14.84	Guinea	Male	90-94	10.90	8.90	13.17	Guinea	Female	90-94	13.20	10.85	15.83
Guinea-Bissau	Both	40-44 years	0.30	0.22	0.40	Guinea-Bissau	Male	80-84	8.78	7.00	10.84	Guinea-Bissau	Female	80-84	11.00	8.90	13.50
Guinea-Bissau	Both	45-49 years	0.66	0.51	0.84	Guinea-Bissau	Male	85-89	10.28	8.29	12.47	Guinea-Bissau	Female	85-89	12.69	10.42	15.39
Guinea-Bissau	Both	50-54 years	1.19	0.90	1.53	Guinea-Bissau	Male	90-94	11.60	9.45	14.10	Guinea-Bissau	Female	90-94	14.00	11.53	16.85
Guinea-Bissau	Both	55-59 years	1.92	1.51	2.35	Guinea-Bissau	Male	40-44 years	0.28	0.20	0.37	Guinea-Bissau	Female	40-44 years	0.32	0.23	0.44
Guinea-Bissau	Both	60-64 years	2.84	2.23	3.60	Guinea-Bissau	Male	45-49 years	0.60	0.46	0.77	Guinea-Bissau	Female	45-49 years	0.70	0.54	0.91
Guinea-Bissau	Both	65-69 years	4.26	3.42	5.26	Guinea-Bissau	Male	50-54 years	1.09	0.82	1.40	Guinea-Bissau	Female	50-54 years	1.28	0.96	1.65
Guinea-Bissau	Both	70-74 years	6.14	4.75	7.66	Guinea-Bissau	Male	55-59 years	1.74	1.36	2.14	Guinea-Bissau	Female	55-59 years	2.08	1.62	2.58
Guinea-Bissau	Both	75-79 years	8.16	6.55	9.94	Guinea-Bissau	Male	60-64 years	2.55	2.01	3.20	Guinea-Bissau	Female	60-64 years	3.10	2.40	3.98
Guinea-Bissau	Both	All ages	0.31	0.27	0.36	Guinea-Bissau	Male	65-69 years	3.79	3.01	4.66	Guinea-Bissau	Female	65-69 years	4.69	3.74	5.80
Guinea-Bissau	Both	80-84	10.18	8.26	12.36	Guinea-Bissau	Male	70-74 years	5.38	4.13	6.76	Guinea-Bissau	Female	70-74 years	6.75	5.25	8.52
Guinea-Bissau	Both	85-89	11.88	9.68	14.40	Guinea-Bissau	Male	75-79 years	7.08	5.64	8.74	Guinea-Bissau	Female	75-79 years	8.91	7.18	10.91
Guinea-Bissau	Both	90-94	13.29	10.94	16.02	Guinea-Bissau	Male	All ages	0.26	0.22	0.30	Guinea-Bissau	Female	All ages	0.36	0.31	0.42
Guyana	Both	40-44 years	0.32	0.23	0.44	Guyana	Male	40-44 years	0.27	0.20	0.37	Guyana	Female	40-44 years	0.37	0.27	0.51
Guyana	Both	45-49 years	0.68	0.51	0.86	Guyana	Male	45-49 years	0.57	0.43	0.74	Guyana	Female	45-49 years	0.78	0.60	1.00
Guyana	Both	50-54 years	1.19	0.91	1.52	Guyana	Male	50-54 years	0.99	0.75	1.29	Guyana	Female	50-54 years	1.38	1.04	1.77
Guyana	Both	55-59 years	1.90	1.48	2.33	Guyana	Male	55-59 years	1.58	1.23	1.95	Guyana	Female	55-59 years	2.20	1.70	2.72
Guyana	Both	60-64 years	2.84	2.21	3.55	Guyana	Male	60-64 years	2.36	1.84	2.95	Guyana	Female	60-64 years	3.27	2.53	4.13
Guyana	Both	65-69 years	4.43	3.56	5.44	Guyana	Male	65-69 years	3.66	2.95	4.49	Guyana	Female	65-69 years	5.12	4.07	6.30
Guyana	Both	70-74 years	6.58	5.08	8.37	Guyana	Male	70-74 years	5.40	4.18	6.84	Guyana	Female	70-74 years	7.59	5.76	9.73
Guyana	Both	75-79 years	8.81	7.08	10.79	Guyana	Male	75-79 years	7.22	5.81	8.82	Guyana	Female	75-79 years	10.07	8.00	12.45
Guyana	Both	All ages	0.75	0.64	0.87	Guyana	Male	All ages	0.58	0.50	0.67	Guyana	Female	All ages	0.91	0.78	1.06
Guyana	Both	80-84	10.96	8.95	13.51	Guyana	Male	80-84	8.98	7.24	11.11	Guyana	Female	80-84	12.37	10.07	15.22

Guyana	Both	85-89	12.72	10.40	15.33	Guyana	Male	85-89	10.47	8.52	12.78	Guyana	Female	85-89	14.10	11.50	17.03
Guyana	Both	90-94	14.04	11.50	16.77	Guyana	Male	90-94	11.75	9.60	14.21	Guyana	Female	90-94	15.33	12.62	18.36
Haiti	Both	75-79 years	7.90	6.30	9.69	Haiti	Male	40-44 years	0.26	0.18	0.35	Haiti	Female	40-44 years	0.34	0.25	0.46
Haiti	Both	All ages	0.45	0.39	0.52	Haiti	Male	45-49 years	0.53	0.40	0.67	Haiti	Female	45-49 years	0.72	0.55	0.91
Haiti	Both	80-84	9.73	7.84	12.02	Haiti	Male	50-54 years	0.92	0.69	1.17	Haiti	Female	50-54 years	1.26	0.95	1.59
Haiti	Both	85-89	11.10	9.09	13.42	Haiti	Male	55-59 years	1.45	1.13	1.77	Haiti	Female	55-59 years	1.99	1.55	2.46
Haiti	Both	90-94	12.22	10.10	14.63	Haiti	Male	60-64 years	2.15	1.68	2.68	Haiti	Female	60-64 years	2.96	2.31	3.68
Haiti	Both	40-44 years	0.30	0.22	0.41	Haiti	Male	65-69 years	3.34	2.69	4.10	Haiti	Female	65-69 years	4.64	3.67	5.74
Haiti	Both	45-49 years	0.63	0.49	0.79	Haiti	Male	70-74 years	4.93	3.81	6.26	Haiti	Female	70-74 years	6.86	5.22	8.83
Haiti	Both	50-54 years	1.09	0.83	1.38	Haiti	Male	75-79 years	6.56	5.19	8.14	Haiti	Female	75-79 years	9.09	7.20	11.15
Haiti	Both	55-59 years	1.73	1.35	2.12	Haiti	Male	All ages	0.37	0.31	0.42	Haiti	Female	All ages	0.53	0.45	0.61
Haiti	Both	60-64 years	2.58	2.03	3.22	Haiti	Male	80-84	8.12	6.52	10.06	Haiti	Female	80-84	11.13	8.89	13.73
Haiti	Both	65-69 years	4.03	3.19	4.95	Haiti	Male	85-89	9.43	7.69	11.50	Haiti	Female	85-89	12.65	10.26	15.29
Haiti	Both	70-74 years	5.95	4.56	7.58	Haiti	Male	90-94	10.55	8.66	12.74	Haiti	Female	90-94	13.72	11.25	16.58
Honduras	Both	40-44 years	0.33	0.24	0.45	Honduras	Male	40-44 years	0.28	0.20	0.38	Honduras	Female	40-44 years	0.38	0.27	0.52
Honduras	Both	45-49 years	0.69	0.52	0.88	Honduras	Male	45-49 years	0.57	0.43	0.73	Honduras	Female	45-49 years	0.79	0.60	1.00
Honduras	Both	50-54 years	1.20	0.91	1.54	Honduras	Male	50-54 years	1.00	0.75	1.29	Honduras	Female	50-54 years	1.39	1.05	1.80
Honduras	Both	55-59 years	1.93	1.50	2.35	Honduras	Male	55-59 years	1.60	1.23	1.95	Honduras	Female	55-59 years	2.23	1.74	2.74
Honduras	Both	60-64 years	2.94	2.29	3.69	Honduras	Male	60-64 years	2.43	1.89	3.06	Honduras	Female	60-64 years	3.40	2.67	4.25
Honduras	Both	65-69 years	4.76	3.79	5.81	Honduras	Male	65-69 years	3.94	3.12	4.85	Honduras	Female	65-69 years	5.48	4.39	6.73
Honduras	Both	70-74 years	7.19	5.48	8.96	Honduras	Male	70-74 years	5.95	4.60	7.47	Honduras	Female	70-74 years	8.27	6.29	10.43
Honduras	Both	75-79 years	9.56	7.69	11.70	Honduras	Male	75-79 years	7.93	6.40	9.80	Honduras	Female	75-79 years	10.99	8.80	13.44
Honduras	Both	All ages	0.62	0.53	0.71	Honduras	Male	All ages	0.51	0.43	0.58	Honduras	Female	All ages	0.72	0.62	0.84
Honduras	Both	80-84	11.68	9.55	14.37	Honduras	Male	80-84	9.72	7.94	11.92	Honduras	Female	80-84	13.38	10.87	16.43

Honduras	Both	85-89	13.17	10.79	15.90	Honduras	Male	85-89	11.11	9.13	13.48	Honduras	Female	85-89	14.99	12.21	18.24
Honduras	Both	90-94	14.13	11.72	17.06	Honduras	Male	90-94	12.19	10.09	14.74	Honduras	Female	90-94	15.95	13.06	19.21
Hungary	Both	40-44 years	0.49	0.36	0.66	Hungary	Male	40-44 years	0.46	0.33	0.61	Hungary	Female	40-44 years	0.53	0.39	0.71
Hungary	Both	45-49 years	1.05	0.81	1.32	Hungary	Male	45-49 years	0.95	0.73	1.20	Hungary	Female	45-49 years	1.15	0.89	1.46
Hungary	Both	50-54 years	1.86	1.42	2.37	Hungary	Male	50-54 years	1.66	1.28	2.12	Hungary	Female	50-54 years	2.05	1.56	2.67
Hungary	Both	55-59 years	2.95	2.30	3.59	Hungary	Male	55-59 years	2.61	2.05	3.20	Hungary	Female	55-59 years	3.26	2.52	4.05
Hungary	Both	60-64 years	4.36	3.40	5.39	Hungary	Male	60-64 years	3.81	2.98	4.72	Hungary	Female	60-64 years	4.82	3.72	6.01
Hungary	Both	65-69 years	6.60	5.34	8.11	Hungary	Male	65-69 years	5.67	4.60	7.00	Hungary	Female	65-69 years	7.30	5.88	8.96
Hungary	Both	70-74 years	9.50	7.27	11.87	Hungary	Male	70-74 years	8.06	6.20	10.13	Hungary	Female	70-74 years	10.47	8.02	13.26
Hungary	Both	75-79 years	12.47	9.81	15.31	Hungary	Male	75-79 years	10.56	8.33	12.96	Hungary	Female	75-79 years	13.56	10.66	16.74
Hungary	Both	All ages	3.01	2.58	3.46	Hungary	Male	All ages	2.16	1.86	2.48	Hungary	Female	All ages	3.76	3.19	4.36
Hungary	Both	80-84	15.29	12.42	18.64	Hungary	Male	80-84	13.00	10.44	15.93	Hungary	Female	80-84	16.34	13.24	19.87
Hungary	Both	85-89	17.48	14.42	21.14	Hungary	Male	85-89	15.07	12.39	18.25	Hungary	Female	85-89	18.44	15.10	22.40
Hungary	Both	90-94	19.13	15.97	22.64	Hungary	Male	90-94	16.82	13.89	20.13	Hungary	Female	90-94	19.91	16.67	23.53
Iceland	Both	40-44 years	0.98	0.73	1.29	Iceland	Male	40-44 years	0.54	0.39	0.72	Iceland	Female	40-44 years	1.43	1.06	1.89
Iceland	Both	45-49 years	1.71	1.34	2.13	Iceland	Male	45-49 years	1.11	0.86	1.41	Iceland	Female	45-49 years	2.32	1.81	2.91
Iceland	Both	50-54 years	2.60	2.02	3.23	Iceland	Male	50-54 years	1.98	1.51	2.53	Iceland	Female	50-54 years	3.20	2.47	4.02
Iceland	Both	55-59 years	3.82	3.05	4.64	Iceland	Male	55-59 years	3.33	2.61	4.08	Iceland	Female	55-59 years	4.31	3.44	5.24
Iceland	Both	60-64 years	5.56	4.46	6.82	Iceland	Male	60-64 years	5.16	4.06	6.49	Iceland	Female	60-64 years	5.97	4.76	7.25
Iceland	Both	65-69 years	8.58	7.01	10.39	Iceland	Male	65-69 years	7.84	6.33	9.60	Iceland	Female	65-69 years	9.33	7.60	11.25
Iceland	Both	70-74 years	12.48	9.70	15.62	Iceland	Male	70-74 years	11.07	8.52	13.70	Iceland	Female	70-74 years	13.88	10.70	17.48
Iceland	Both	75-79 years	16.31	13.03	19.85	Iceland	Male	75-79 years	14.06	11.14	17.15	Iceland	Female	75-79 years	18.29	14.63	22.16
Iceland	Both	All ages	3.25	2.82	3.69	Iceland	Male	All ages	2.63	2.25	3.02	Iceland	Female	All ages	3.85	3.34	4.37
Iceland	Both	80-84	19.72	16.01	23.65	Iceland	Male	80-84	16.64	13.47	20.05	Iceland	Female	80-84	22.25	18.10	26.85

Iceland	Both	85-89	22.46	18.73	26.74	Iceland	Male	85-89	18.54	15.26	22.18	Iceland	Female	85-89	25.20	20.89	30.06
Iceland	Both	90-94	24.59	20.71	28.88	Iceland	Male	90-94	19.74	16.50	23.24	Iceland	Female	90-94	27.08	22.77	31.86
India	Both	40-44 years	0.30	0.22	0.40	India	Male	65-69 years	3.26	2.64	4.00	India	Female	40-44 years	0.36	0.26	0.47
India	Both	45-49 years	0.67	0.52	0.85	India	Male	70-74 years	4.57	3.54	5.69	India	Female	45-49 years	0.80	0.62	1.01
India	Both	50-54 years	1.22	0.93	1.57	India	Male	75-79 years	6.01	4.83	7.29	India	Female	50-54 years	1.48	1.13	1.92
India	Both	55-59 years	1.96	1.54	2.38	India	Male	All ages	0.53	0.46	0.61	India	Female	55-59 years	2.38	1.87	2.89
India	Both	60-64 years	2.87	2.26	3.54	India	Male	80-84	7.49	6.11	9.22	India	Female	60-64 years	3.48	2.74	4.31
India	Both	65-69 years	4.23	3.42	5.17	India	Male	85-89	8.94	7.35	10.72	India	Female	65-69 years	5.15	4.18	6.30
India	Both	70-74 years	5.96	4.62	7.42	India	Male	90-94	10.39	8.55	12.49	India	Female	70-74 years	7.28	5.65	9.05
India	Both	75-79 years	7.87	6.33	9.56	India	Male	40-44 years	0.25	0.18	0.33	India	Female	75-79 years	9.50	7.59	11.58
India	Both	All ages	0.72	0.62	0.83	India	Male	45-49 years	0.54	0.42	0.68	India	Female	All ages	0.92	0.79	1.05
India	Both	80-84	9.84	8.05	12.06	India	Male	50-54 years	0.97	0.74	1.24	India	Female	80-84	11.64	9.52	14.18
India	Both	85-89	11.47	9.49	13.74	India	Male	55-59 years	1.54	1.21	1.86	India	Female	85-89	13.40	11.08	16.01
India	Both	90-94	12.88	10.66	15.34	India	Male	60-64 years	2.23	1.76	2.75	India	Female	90-94	14.81	12.28	17.61
Indonesia	Both	85-89	18.14	15.04	21.63	Indonesia	Male	85-89	11.54	9.59	13.78	Indonesia	Female	40-44 years	0.70	0.52	0.91
Indonesia	Both	90-94	19.40	16.30	22.76	Indonesia	Male	90-94	12.70	10.53	15.06	Indonesia	Female	45-49 years	1.66	1.29	2.09
Indonesia	Both	40-44 years	0.56	0.41	0.72	Indonesia	Male	40-44 years	0.42	0.31	0.55	Indonesia	Female	50-54 years	3.13	2.36	4.05
Indonesia	Both	45-49 years	1.29	1.01	1.62	Indonesia	Male	45-49 years	0.93	0.72	1.17	Indonesia	Female	55-59 years	4.95	3.86	6.05
Indonesia	Both	50-54 years	2.41	1.83	3.10	Indonesia	Male	50-54 years	1.69	1.30	2.16	Indonesia	Female	60-64 years	7.09	5.56	8.68
Indonesia	Both	55-59 years	3.80	2.97	4.62	Indonesia	Male	55-59 years	2.64	2.09	3.20	Indonesia	Female	65-69 years	10.17	8.21	12.35
Indonesia	Both	60-64 years	5.42	4.26	6.64	Indonesia	Male	60-64 years	3.73	2.96	4.57	Indonesia	Female	70-74 years	13.92	10.87	17.24
Indonesia	Both	65-69 years	7.73	6.29	9.41	Indonesia	Male	65-69 years	5.18	4.24	6.30	Indonesia	Female	75-79 years	17.43	14.04	21.05
Indonesia	Both	70-74 years	10.70	8.39	13.23	Indonesia	Male	70-74 years	6.90	5.41	8.56	Indonesia	Female	All ages	1.81	1.55	2.07
Indonesia	Both	75-79 years	13.66	11.01	16.47	Indonesia	Male	75-79 years	8.61	6.93	10.39	Indonesia	Female	80-84	20.42	16.90	24.67

Indonesia	Both	All ages	1.33	1.14	1.52	Indonesia	Male	All ages	0.85	0.73	0.98	Indonesia	Female	85-89	22.36	18.51	26.77
Indonesia	Both	80-84	16.27	13.49	19.67	Indonesia	Male	80-84	10.20	8.37	12.38	Indonesia	Female	90-94	23.42	19.65	27.61
Iran (Islamic Republic of)	Both	All ages	0.97	0.84	1.11	Iran (Islamic Republic of)	Male	40-44 years	0.30	0.22	0.40	Iran (Islamic Republic of)	Female	40-44 years	0.44	0.32	0.58
Iran (Islamic Republic of)	Both	80-84	11.83	9.69	14.40	Iran (Islamic Republic of)	Male	45-49 years	0.66	0.51	0.82	Iran (Islamic Republic of)	Female	45-49 years	0.99	0.76	1.24
Iran (Islamic Republic of)	Both	85-89	13.52	11.16	16.18	Iran (Islamic Republic of)	Male	50-54 years	1.19	0.91	1.51	Iran (Islamic Republic of)	Female	50-54 years	1.84	1.39	2.35
Iran (Islamic Republic of)	Both	90-94	14.96	12.44	17.82	Iran (Islamic Republic of)	Male	55-59 years	1.91	1.51	2.32	Iran (Islamic Republic of)	Female	55-59 years	2.99	2.33	3.66
Iran (Islamic Republic of)	Both	40-44 years	0.37	0.27	0.49	Iran (Islamic Republic of)	Male	60-64 years	2.80	2.22	3.43	Iran (Islamic Republic of)	Female	60-64 years	4.45	3.50	5.45
Iran (Islamic Republic of)	Both	45-49 years	0.82	0.63	1.03	Iran (Islamic Republic of)	Male	65-69 years	4.11	3.33	5.00	Iran (Islamic Republic of)	Female	65-69 years	6.61	5.35	8.03
Iran (Islamic Republic of)	Both	50-54 years	1.51	1.16	1.93	Iran (Islamic Republic of)	Male	70-74 years	5.77	4.51	7.21	Iran (Islamic Republic of)	Female	70-74 years	9.30	7.18	11.57
Iran (Islamic Republic of)	Both	55-59 years	2.45	1.92	2.98	Iran (Islamic Republic of)	Male	75-79 years	7.54	6.07	9.19	Iran (Islamic Republic of)	Female	75-79 years	11.98	9.60	14.62
Iran (Islamic Republic of)	Both	60-64 years	3.63	2.88	4.44	Iran (Islamic Republic of)	Male	All ages	0.76	0.66	0.86	Iran (Islamic Republic of)	Female	All ages	1.19	1.03	1.36
Iran (Islamic Republic of)	Both	65-69 years	5.40	4.37	6.57	Iran (Islamic Republic of)	Male	80-84	9.32	7.58	11.39	Iran (Islamic Republic of)	Female	80-84	14.46	11.87	17.50
Iran (Islamic Republic of)	Both	70-74 years	7.61	5.90	9.50	Iran (Islamic Republic of)	Male	85-89	10.97	9.01	13.12	Iran (Islamic Republic of)	Female	85-89	16.38	13.54	19.60
Iran (Islamic Republic of)	Both	75-79 years	9.78	7.84	11.93	Iran (Islamic Republic of)	Male	90-94	12.54	10.34	14.91	Iran (Islamic Republic of)	Female	90-94	17.86	14.87	21.20

Iraq	Both	40-44 years	0.43	0.32	0.58	Iraq	Male	40-44 years	0.38	0.28	0.53	Iraq	Female	40-44 years	0.49	0.36	0.66
Iraq	Both	45-49 years	0.93	0.72	1.18	Iraq	Male	45-49 years	0.81	0.62	1.03	Iraq	Female	45-49 years	1.05	0.81	1.33
Iraq	Both	50-54 years	1.65	1.25	2.10	Iraq	Male	50-54 years	1.43	1.08	1.86	Iraq	Female	50-54 years	1.87	1.42	2.40
Iraq	Both	55-59 years	2.60	2.03	3.20	Iraq	Male	55-59 years	2.26	1.75	2.81	Iraq	Female	55-59 years	2.95	2.30	3.61
Iraq	Both	60-64 years	3.80	2.99	4.72	Iraq	Male	60-64 years	3.29	2.57	4.11	Iraq	Female	60-64 years	4.31	3.36	5.35
Iraq	Both	65-69 years	5.71	4.58	6.98	Iraq	Male	65-69 years	4.89	3.91	6.01	Iraq	Female	65-69 years	6.50	5.15	7.96
Iraq	Both	70-74 years	8.17	6.24	10.32	Iraq	Male	70-74 years	6.96	5.33	8.78	Iraq	Female	70-74 years	9.31	7.08	11.85
Iraq	Both	75-79 years	10.68	8.45	13.11	Iraq	Male	75-79 years	9.10	7.19	11.21	Iraq	Female	75-79 years	12.09	9.60	14.90
Iraq	Both	All ages	0.65	0.56	0.74	Iraq	Male	All ages	0.54	0.46	0.62	Iraq	Female	All ages	0.76	0.65	0.87
Iraq	Both	80-84	13.09	10.62	15.95	Iraq	Male	80-84	11.19	8.92	13.81	Iraq	Female	80-84	14.63	11.83	17.87
Iraq	Both	85-89	15.04	12.35	18.06	Iraq	Male	85-89	12.96	10.51	15.64	Iraq	Female	85-89	16.57	13.58	19.95
Iraq	Both	90-94	16.58	13.81	19.89	Iraq	Male	90-94	14.47	11.86	17.34	Iraq	Female	90-94	17.96	14.87	21.65
Ireland	Both	40-44 years	1.14	0.86	1.47	Ireland	Male	40-44 years	0.60	0.44	0.80	Ireland	Female	40-44 years	1.65	1.23	2.16
Ireland	Both	45-49 years	1.96	1.55	2.40	Ireland	Male	45-49 years	1.25	0.97	1.58	Ireland	Female	45-49 years	2.64	2.06	3.26
Ireland	Both	50-54 years	2.92	2.28	3.64	Ireland	Male	50-54 years	2.24	1.70	2.84	Ireland	Female	50-54 years	3.58	2.80	4.49
Ireland	Both	55-59 years	4.25	3.35	5.15	Ireland	Male	55-59 years	3.73	2.91	4.63	Ireland	Female	55-59 years	4.76	3.74	5.82
Ireland	Both	60-64 years	6.15	4.88	7.62	Ireland	Male	60-64 years	5.74	4.48	7.22	Ireland	Female	60-64 years	6.55	5.20	8.02
Ireland	Both	65-69 years	9.55	7.83	11.60	Ireland	Male	65-69 years	8.75	7.10	10.73	Ireland	Female	65-69 years	10.34	8.43	12.55
Ireland	Both	70-74 years	13.99	10.86	17.46	Ireland	Male	70-74 years	12.42	9.55	15.71	Ireland	Female	70-74 years	15.52	11.98	19.38
Ireland	Both	75-79 years	18.26	14.64	22.38	Ireland	Male	75-79 years	15.83	12.51	19.67	Ireland	Female	75-79 years	20.46	16.48	24.76
Ireland	Both	All ages	3.47	2.99	3.94	Ireland	Male	All ages	2.86	2.46	3.31	Ireland	Female	All ages	4.03	3.48	4.57
Ireland	Both	80-84	22.17	18.12	26.51	Ireland	Male	80-84	18.80	15.17	22.74	Ireland	Female	80-84	24.84	20.17	29.44
Ireland	Both	85-89	25.24	21.00	29.88	Ireland	Male	85-89	20.96	17.25	25.24	Ireland	Female	85-89	28.05	23.44	33.14
Ireland	Both	90-94	27.58	23.25	32.33	Ireland	Male	90-94	22.29	18.58	26.44	Ireland	Female	90-94	30.04	25.39	35.19

Israel	Both	40-44 years	1.08	0.82	1.41	Israel	Male	80-84	17.99	14.69	21.81	Israel	Female	40-44 years	1.58	1.19	2.05
Israel	Both	45-49 years	1.88	1.48	2.31	Israel	Male	85-89	19.86	16.37	23.80	Israel	Female	45-49 years	2.54	2.01	3.18
Israel	Both	50-54 years	2.82	2.20	3.54	Israel	Male	90-94	20.90	17.39	24.81	Israel	Female	50-54 years	3.47	2.69	4.36
Israel	Both	55-59 years	4.13	3.30	5.00	Israel	Male	40-44 years	0.57	0.42	0.77	Israel	Female	55-59 years	4.63	3.70	5.66
Israel	Both	60-64 years	6.01	4.84	7.31	Israel	Male	45-49 years	1.20	0.93	1.50	Israel	Female	60-64 years	6.40	5.14	7.70
Israel	Both	65-69 years	9.33	7.53	11.18	Israel	Male	50-54 years	2.15	1.65	2.74	Israel	Female	65-69 years	10.07	8.15	12.14
Israel	Both	70-74 years	13.64	10.56	17.04	Israel	Male	55-59 years	3.61	2.83	4.46	Israel	Female	70-74 years	15.02	11.61	18.90
Israel	Both	75-79 years	17.70	14.14	21.61	Israel	Male	60-64 years	5.57	4.43	6.86	Israel	Female	75-79 years	19.70	15.67	24.05
Israel	Both	All ages	2.75	2.39	3.12	Israel	Male	65-69 years	8.50	6.83	10.22	Israel	Female	All ages	3.30	2.86	3.77
Israel	Both	80-84	21.33	17.57	25.73	Israel	Male	70-74 years	12.04	9.30	15.04	Israel	Female	80-84	23.79	19.33	28.67
Israel	Both	85-89	24.00	19.98	28.58	Israel	Male	75-79 years	15.27	12.18	18.65	Israel	Female	85-89	26.73	22.06	31.89
Israel	Both	90-94	25.71	21.60	30.38	Israel	Male	All ages	2.17	1.86	2.49	Israel	Female	90-94	28.50	24.01	33.76
Italy	Both	45-49 years	1.71	1.37	2.11	Italy	Male	40-44 years	0.45	0.33	0.58	Italy	Female	40-44 years	1.41	1.07	1.83
Italy	Both	50-54 years	2.69	2.09	3.39	Italy	Male	45-49 years	0.98	0.76	1.24	Italy	Female	45-49 years	2.42	1.93	2.98
Italy	Both	55-59 years	4.02	3.18	4.83	Italy	Male	50-54 years	1.81	1.39	2.29	Italy	Female	50-54 years	3.54	2.76	4.44
Italy	Both	60-64 years	5.82	4.69	7.06	Italy	Male	55-59 years	3.05	2.40	3.67	Italy	Female	55-59 years	4.93	3.90	5.95
Italy	Both	65-69 years	8.87	7.23	10.67	Italy	Male	60-64 years	4.68	3.70	5.75	Italy	Female	60-64 years	6.87	5.48	8.33
Italy	Both	70-74 years	12.83	10.09	15.75	Italy	Male	65-69 years	7.03	5.71	8.49	Italy	Female	65-69 years	10.55	8.66	12.63
Italy	Both	75-79 years	16.78	13.54	20.30	Italy	Male	70-74 years	9.87	7.68	12.17	Italy	Female	70-74 years	15.43	12.16	19.02
Italy	Both	All ages	4.83	4.18	5.48	Italy	Male	75-79 years	12.57	10.09	15.23	Italy	Female	75-79 years	20.15	16.27	24.37
Italy	Both	80-84	20.46	16.99	24.46	Italy	Male	All ages	3.24	2.77	3.70	Italy	Female	All ages	6.29	5.45	7.11
Italy	Both	85-89	23.47	19.61	27.90	Italy	Male	80-84	14.97	12.26	18.06	Italy	Female	80-84	24.33	20.23	29.02
Italy	Both	90-94	25.80	21.73	30.14	Italy	Male	85-89	16.74	13.82	20.07	Italy	Female	85-89	27.23	22.84	32.26
Italy	Both	40-44 years	0.94	0.71	1.21	Italy	Male	90-94	17.94	15.00	21.21	Italy	Female	90-94	28.96	24.46	33.83

Jamaica	Both	40-44 years	0.35	0.25	0.47	Jamaica	Male	40-44 years	0.29	0.21	0.39	Jamaica	Female	40-44 years	0.39	0.28	0.54
Jamaica	Both	45-49 years	0.73	0.56	0.92	Jamaica	Male	45-49 years	0.61	0.47	0.78	Jamaica	Female	45-49 years	0.84	0.64	1.07
Jamaica	Both	50-54 years	1.28	0.97	1.63	Jamaica	Male	50-54 years	1.08	0.81	1.36	Jamaica	Female	50-54 years	1.49	1.13	1.93
Jamaica	Both	55-59 years	2.07	1.62	2.55	Jamaica	Male	55-59 years	1.74	1.36	2.12	Jamaica	Female	55-59 years	2.42	1.87	3.01
Jamaica	Both	60-64 years	3.16	2.47	3.99	Jamaica	Male	60-64 years	2.63	2.07	3.29	Jamaica	Female	60-64 years	3.67	2.84	4.65
Jamaica	Both	65-69 years	4.94	3.95	6.12	Jamaica	Male	65-69 years	4.11	3.27	5.12	Jamaica	Female	65-69 years	5.76	4.60	7.16
Jamaica	Both	70-74 years	7.27	5.61	9.19	Jamaica	Male	70-74 years	6.04	4.65	7.71	Jamaica	Female	70-74 years	8.49	6.55	10.69
Jamaica	Both	75-79 years	9.68	7.71	11.91	Jamaica	Male	75-79 years	8.00	6.29	9.94	Jamaica	Female	75-79 years	11.19	8.94	13.78
Jamaica	Both	All ages	1.19	1.03	1.35	Jamaica	Male	All ages	0.94	0.81	1.07	Jamaica	Female	All ages	1.43	1.24	1.63
Jamaica	Both	80-84	11.98	9.75	14.71	Jamaica	Male	80-84	9.83	7.98	12.22	Jamaica	Female	80-84	13.61	11.02	16.68
Jamaica	Both	85-89	13.76	11.28	16.65	Jamaica	Male	85-89	11.35	9.24	13.84	Jamaica	Female	85-89	15.34	12.59	18.62
Jamaica	Both	90-94	15.04	12.48	18.03	Jamaica	Male	90-94	12.60	10.39	15.26	Jamaica	Female	90-94	16.46	13.63	19.73
Japan	Both	85-89	17.52	14.60	20.91	Japan	Male	40-44 years	0.24	0.17	0.32	Japan	Female	85-89	22.29	18.57	26.56
Japan	Both	90-94	19.98	16.84	23.52	Japan	Male	45-49 years	0.51	0.40	0.65	Japan	Female	90-94	24.09	20.19	28.26
Japan	Both	40-44 years	0.59	0.43	0.79	Japan	Male	50-54 years	0.93	0.71	1.19	Japan	Female	40-44 years	0.94	0.69	1.25
Japan	Both	45-49 years	1.10	0.86	1.37	Japan	Male	55-59 years	1.56	1.24	1.89	Japan	Female	45-49 years	1.69	1.32	2.09
Japan	Both	50-54 years	1.76	1.37	2.22	Japan	Male	60-64 years	2.37	1.88	2.93	Japan	Female	50-54 years	2.60	2.02	3.27
Japan	Both	55-59 years	2.66	2.09	3.23	Japan	Male	65-69 years	3.50	2.83	4.26	Japan	Female	55-59 years	3.76	2.95	4.55
Japan	Both	60-64 years	3.89	3.10	4.76	Japan	Male	70-74 years	4.86	3.79	6.03	Japan	Female	60-64 years	5.37	4.26	6.57
Japan	Both	65-69 years	5.93	4.82	7.19	Japan	Male	75-79 years	6.26	5.05	7.60	Japan	Female	65-69 years	8.21	6.68	9.90
Japan	Both	70-74 years	8.61	6.71	10.66	Japan	Male	All ages	1.94	1.66	2.24	Japan	Female	70-74 years	11.99	9.36	14.85
Japan	Both	75-79 years	11.55	9.29	14.04	Japan	Male	80-84	7.65	6.20	9.37	Japan	Female	75-79 years	15.87	12.76	19.27
Japan	Both	All ages	3.97	3.42	4.54	Japan	Male	85-89	8.97	7.42	10.75	Japan	Female	All ages	5.85	5.04	6.69
Japan	Both	80-84	14.60	12.04	17.62	Japan	Male	90-94	10.23	8.47	12.23	Japan	Female	80-84	19.54	16.18	23.45

Jordan	Both	40-44 years	0.42	0.31	0.56	Jordan	Male	40-44 years	0.38	0.27	0.51	Jordan	Female	40-44 years	0.48	0.34	0.64
Jordan	Both	45-49 years	0.90	0.69	1.14	Jordan	Male	45-49 years	0.79	0.61	1.00	Jordan	Female	45-49 years	1.02	0.79	1.31
Jordan	Both	50-54 years	1.60	1.21	2.06	Jordan	Male	50-54 years	1.40	1.06	1.79	Jordan	Female	50-54 years	1.83	1.38	2.38
Jordan	Both	55-59 years	2.53	1.97	3.10	Jordan	Male	55-59 years	2.22	1.73	2.74	Jordan	Female	55-59 years	2.89	2.23	3.56
Jordan	Both	60-64 years	3.72	2.88	4.63	Jordan	Male	60-64 years	3.24	2.50	4.04	Jordan	Female	60-64 years	4.25	3.30	5.30
Jordan	Both	65-69 years	5.57	4.48	6.81	Jordan	Male	65-69 years	4.82	3.88	5.90	Jordan	Female	65-69 years	6.37	5.10	7.83
Jordan	Both	70-74 years	7.94	6.14	10.00	Jordan	Male	70-74 years	6.82	5.27	8.70	Jordan	Female	70-74 years	9.06	6.95	11.47
Jordan	Both	75-79 years	10.27	8.23	12.50	Jordan	Male	75-79 years	8.89	7.12	10.95	Jordan	Female	75-79 years	11.74	9.32	14.35
Jordan	Both	All ages	0.66	0.57	0.76	Jordan	Male	All ages	0.58	0.50	0.67	Jordan	Female	All ages	0.75	0.64	0.86
Jordan	Both	80-84	12.52	10.12	15.23	Jordan	Male	80-84	10.89	8.76	13.56	Jordan	Female	80-84	14.21	11.43	17.26
Jordan	Both	85-89	14.30	11.73	17.22	Jordan	Male	85-89	12.62	10.30	15.37	Jordan	Female	85-89	16.08	13.12	19.49
Jordan	Both	90-94	15.40	12.77	18.32	Jordan	Male	90-94	14.09	11.45	16.94	Jordan	Female	90-94	17.40	14.38	20.69
Kazakhstan	Both	40-44 years	0.43	0.32	0.58	Kazakhstan	Male	40-44 years	0.38	0.28	0.52	Kazakhstan	Female	40-44 years	0.48	0.35	0.64
Kazakhstan	Both	45-49 years	0.91	0.70	1.15	Kazakhstan	Male	45-49 years	0.79	0.61	0.99	Kazakhstan	Female	45-49 years	1.03	0.78	1.32
Kazakhstan	Both	50-54 years	1.62	1.22	2.07	Kazakhstan	Male	50-54 years	1.36	1.04	1.75	Kazakhstan	Female	50-54 years	1.84	1.38	2.38
Kazakhstan	Both	55-59 years	2.56	2.01	3.16	Kazakhstan	Male	55-59 years	2.14	1.67	2.63	Kazakhstan	Female	55-59 years	2.92	2.25	3.64
Kazakhstan	Both	60-64 years	3.78	2.97	4.67	Kazakhstan	Male	60-64 years	3.11	2.47	3.84	Kazakhstan	Female	60-64 years	4.30	3.37	5.38
Kazakhstan	Both	65-69 years	5.72	4.65	6.94	Kazakhstan	Male	65-69 years	4.61	3.71	5.56	Kazakhstan	Female	65-69 years	6.47	5.19	7.90
Kazakhstan	Both	70-74 years	8.25	6.45	10.47	Kazakhstan	Male	70-74 years	6.54	5.10	8.15	Kazakhstan	Female	70-74 years	9.26	7.24	11.79
Kazakhstan	Both	75-79 years	10.94	8.82	13.41	Kazakhstan	Male	75-79 years	8.59	6.84	10.48	Kazakhstan	Female	75-79 years	12.07	9.67	14.90
Kazakhstan	Both	All ages	1.16	1.00	1.33	Kazakhstan	Male	All ages	0.78	0.67	0.89	Kazakhstan	Female	All ages	1.50	1.30	1.73
Kazakhstan	Both	80-84	13.50	10.86	16.40	Kazakhstan	Male	80-84	10.62	8.48	13.11	Kazakhstan	Female	80-84	14.70	11.81	17.93
Kazakhstan	Both	85-89	15.69	12.89	18.92	Kazakhstan	Male	85-89	12.46	10.22	15.12	Kazakhstan	Female	85-89	16.79	13.74	20.22
Kazakhstan	Both	90-94	17.36	14.41	20.65	Kazakhstan	Male	90-94	14.16	11.67	16.93	Kazakhstan	Female	90-94	18.37	15.16	21.93

Kenya	Both	40-44 years	0.28	0.21	0.38	Kenya	Male	80-84	8.25	6.71	10.14	Kenya	Female	80-84	12.75	10.43	15.55
Kenya	Both	45-49 years	0.66	0.51	0.83	Kenya	Male	85-89	9.80	8.03	11.72	Kenya	Female	85-89	14.70	12.16	17.62
Kenya	Both	50-54 years	1.23	0.94	1.58	Kenya	Male	90-94	11.30	9.29	13.55	Kenya	Female	90-94	16.25	13.41	19.49
Kenya	Both	55-59 years	2.03	1.59	2.47	Kenya	Male	40-44 years	0.24	0.17	0.32	Kenya	Female	40-44 years	0.33	0.24	0.44
Kenya	Both	60-64 years	3.05	2.39	3.79	Kenya	Male	45-49 years	0.54	0.42	0.69	Kenya	Female	45-49 years	0.77	0.59	0.98
Kenya	Both	65-69 years	4.56	3.68	5.58	Kenya	Male	50-54 years	1.01	0.77	1.29	Kenya	Female	50-54 years	1.46	1.11	1.88
Kenya	Both	70-74 years	6.54	5.07	8.17	Kenya	Male	55-59 years	1.64	1.28	2.00	Kenya	Female	55-59 years	2.42	1.89	2.94
Kenya	Both	75-79 years	8.74	7.03	10.66	Kenya	Male	60-64 years	2.43	1.92	3.02	Kenya	Female	60-64 years	3.64	2.85	4.54
Kenya	Both	All ages	0.40	0.34	0.45	Kenya	Male	65-69 years	3.58	2.89	4.40	Kenya	Female	65-69 years	5.49	4.44	6.71
Kenya	Both	80-84	11.06	9.04	13.56	Kenya	Male	70-74 years	5.03	3.91	6.26	Kenya	Female	70-74 years	7.86	6.06	9.81
Kenya	Both	85-89	13.08	10.78	15.69	Kenya	Male	75-79 years	6.62	5.32	8.04	Kenya	Female	75-79 years	10.35	8.30	12.65
Kenya	Both	90-94	14.89	12.25	17.86	Kenya	Male	All ages	0.29	0.25	0.34	Kenya	Female	All ages	0.50	0.43	0.57
Kiribati	Both	55-59 years	4.64	3.60	5.72	Kiribati	Male	55-59 years	4.01	3.09	4.93	Kiribati	Female	55-59 years	5.23	4.07	6.44
Kiribati	Both	60-64 years	6.54	5.09	8.11	Kiribati	Male	60-64 years	5.58	4.27	6.94	Kiribati	Female	60-64 years	7.36	5.70	9.18
Kiribati	Both	65-69 years	9.61	7.68	11.61	Kiribati	Male	65-69 years	7.83	6.21	9.53	Kiribati	Female	65-69 years	10.75	8.56	13.08
Kiribati	Both	70-74 years	13.41	10.43	16.76	Kiribati	Male	70-74 years	10.57	8.14	13.25	Kiribati	Female	70-74 years	15.00	11.60	18.83
Kiribati	Both	75-79 years	16.66	13.28	20.45	Kiribati	Male	75-79 years	13.06	10.33	16.05	Kiribati	Female	75-79 years	18.76	14.78	23.16
Kiribati	Both	All ages	1.06	0.91	1.22	Kiribati	Male	All ages	0.78	0.66	0.91	Kiribati	Female	All ages	1.33	1.13	1.52
Kiribati	Both	80-84	19.39	15.87	23.59	Kiribati	Male	80-84	15.10	12.27	18.37	Kiribati	Female	80-84	21.68	17.70	26.47
Kiribati	Both	85-89	21.00	17.35	25.43	Kiribati	Male	85-89	16.38	13.55	19.68	Kiribati	Female	85-89	23.24	19.25	28.16
Kiribati	Both	90-94	21.65	17.88	25.88	Kiribati	Male	90-94	17.05	13.99	20.44	Kiribati	Female	90-94	23.66	19.63	28.35
Kiribati	Both	40-44 years	0.76	0.56	1.01	Kiribati	Male	40-44 years	0.67	0.49	0.90	Kiribati	Female	40-44 years	0.84	0.62	1.13
Kiribati	Both	45-49 years	1.69	1.29	2.15	Kiribati	Male	45-49 years	1.47	1.13	1.86	Kiribati	Female	45-49 years	1.89	1.44	2.39
Kiribati	Both	50-54 years	3.03	2.28	3.93	Kiribati	Male	50-54 years	2.62	1.97	3.39	Kiribati	Female	50-54 years	3.41	2.54	4.44

Kuwait	Both	80-84	11.79	9.68	14.43	Kuwait	Male	40-44 years	0.35	0.25	0.48	Kuwait	Female	80-84	13.87	11.30	16.85
Kuwait	Both	85-89	14.00	11.62	16.82	Kuwait	Male	45-49 years	0.74	0.57	0.94	Kuwait	Female	85-89	16.05	13.33	19.23
Kuwait	Both	90-94	15.77	13.11	18.76	Kuwait	Male	50-54 years	1.30	0.99	1.68	Kuwait	Female	90-94	17.85	14.89	21.33
Kuwait	Both	40-44 years	0.39	0.28	0.52	Kuwait	Male	55-59 years	2.08	1.62	2.57	Kuwait	Female	40-44 years	0.44	0.32	0.59
Kuwait	Both	45-49 years	0.83	0.64	1.05	Kuwait	Male	60-64 years	3.09	2.41	3.80	Kuwait	Female	45-49 years	0.94	0.72	1.19
Kuwait	Both	50-54 years	1.46	1.11	1.87	Kuwait	Male	65-69 years	4.59	3.70	5.62	Kuwait	Female	50-54 years	1.68	1.27	2.16
Kuwait	Both	55-59 years	2.35	1.81	2.90	Kuwait	Male	70-74 years	6.51	5.09	8.15	Kuwait	Female	55-59 years	2.72	2.11	3.35
Kuwait	Both	60-64 years	3.48	2.73	4.28	Kuwait	Male	75-79 years	8.57	6.89	10.47	Kuwait	Female	60-64 years	4.07	3.17	5.07
Kuwait	Both	65-69 years	5.24	4.22	6.41	Kuwait	Male	All ages	0.65	0.56	0.74	Kuwait	Female	65-69 years	6.11	4.86	7.48
Kuwait	Both	70-74 years	7.28	5.66	9.04	Kuwait	Male	80-84	10.63	8.66	13.26	Kuwait	Female	70-74 years	8.67	6.67	10.82
Kuwait	Both	75-79 years	9.66	7.77	11.72	Kuwait	Male	85-89	12.55	10.31	15.31	Kuwait	Female	75-79 years	11.31	9.00	13.96
Kuwait	Both	All ages	0.65	0.56	0.74	Kuwait	Male	90-94	14.35	11.85	17.17	Kuwait	Female	All ages	0.66	0.56	0.75
Kyrgyzstan	Both	40-44 years	0.40	0.29	0.53	Kyrgyzstan	Male	45-49 years	0.72	0.56	0.91	Kyrgyzstan	Female	45-49 years	0.93	0.72	1.19
Kyrgyzstan	Both	45-49 years	0.83	0.65	1.05	Kyrgyzstan	Male	50-54 years	1.23	0.93	1.57	Kyrgyzstan	Female	50-54 years	1.66	1.25	2.14
Kyrgyzstan	Both	50-54 years	1.46	1.10	1.87	Kyrgyzstan	Male	55-59 years	1.91	1.50	2.34	Kyrgyzstan	Female	55-59 years	2.62	2.03	3.23
Kyrgyzstan	Both	55-59 years	2.29	1.79	2.78	Kyrgyzstan	Male	60-64 years	2.76	2.19	3.44	Kyrgyzstan	Female	60-64 years	3.83	2.95	4.74
Kyrgyzstan	Both	60-64 years	3.35	2.62	4.14	Kyrgyzstan	Male	65-69 years	4.06	3.24	4.96	Kyrgyzstan	Female	65-69 years	5.73	4.60	7.09
Kyrgyzstan	Both	65-69 years	5.02	4.03	6.19	Kyrgyzstan	Male	70-74 years	5.73	4.49	7.13	Kyrgyzstan	Female	70-74 years	8.18	6.30	10.38
Kyrgyzstan	Both	70-74 years	7.20	5.55	9.07	Kyrgyzstan	Male	75-79 years	7.49	6.05	9.12	Kyrgyzstan	Female	75-79 years	10.60	8.37	13.08
Kyrgyzstan	Both	75-79 years	9.38	7.52	11.48	Kyrgyzstan	Male	All ages	0.54	0.46	0.62	Kyrgyzstan	Female	All ages	0.95	0.82	1.09
Kyrgyzstan	Both	All ages	0.75	0.65	0.86	Kyrgyzstan	Male	80-84	9.22	7.39	11.23	Kyrgyzstan	Female	80-84	12.80	10.20	15.77
Kyrgyzstan	Both	80-84	11.54	9.30	14.22	Kyrgyzstan	Male	85-89	10.71	8.72	12.98	Kyrgyzstan	Female	85-89	14.42	11.71	17.66
Kyrgyzstan	Both	85-89	13.20	10.77	16.05	Kyrgyzstan	Male	90-94	12.04	9.96	14.49	Kyrgyzstan	Female	90-94	15.54	12.85	18.62
Kyrgyzstan	Both	90-94	14.38	11.92	17.17	Kyrgyzstan	Male	40-44 years	0.35	0.25	0.48	Kyrgyzstan	Female	40-44 years	0.44	0.32	0.59

Lao People's Democratic Republic	Both	All ages	0.87	0.74	0.99	Lao People's Democratic Republic	Male	40-44 years	0.50	0.36	0.67	Lao People's Democratic Republic	Female	40-44 years	0.64	0.47	0.85
Lao People's Democratic Republic	Both	80-84	14.27	11.63	17.18	Lao People's Democratic Republic	Male	45-49 years	1.07	0.82	1.35	Lao People's Democratic Republic	Female	45-49 years	1.42	1.08	1.82
Lao People's Democratic Republic	Both	85-89	16.00	13.17	19.28	Lao People's Democratic Republic	Male	50-54 years	1.88	1.40	2.40	Lao People's Democratic Republic	Female	50-54 years	2.58	1.92	3.35
Lao People's Democratic Republic	Both	90-94	17.28	14.16	20.52	Lao People's Democratic Republic	Male	55-59 years	2.88	2.27	3.51	Lao People's Democratic Republic	Female	55-59 years	3.99	3.09	4.91
Lao People's Democratic Republic	Both	40-44 years	0.57	0.42	0.75	Lao People's Democratic Republic	Male	60-64 years	4.01	3.17	5.01	Lao People's Democratic Republic	Female	60-64 years	5.63	4.36	6.94
Lao People's Democratic Republic	Both	45-49 years	1.25	0.96	1.58	Lao People's Democratic Republic	Male	65-69 years	5.55	4.49	6.89	Lao People's Democratic Republic	Female	65-69 years	8.08	6.43	9.78
Lao People's Democratic Republic	Both	50-54 years	2.24	1.67	2.87	Lao People's Democratic Republic	Male	70-74 years	7.43	5.75	9.33	Lao People's Democratic Republic	Female	70-74 years	11.16	8.67	13.89
Lao People's Democratic Republic	Both	55-59 years	3.44	2.68	4.19	Lao People's Democratic Republic	Male	75-79 years	9.29	7.37	11.29	Lao People's Democratic Republic	Female	75-79 years	14.15	11.21	17.47
Lao People's Democratic Republic	Both	60-64 years	4.82	3.79	5.92	Lao People's Democratic Republic	Male	All ages	0.68	0.58	0.78	Lao People's Democratic Republic	Female	All ages	1.05	0.90	1.20
Lao People's Democratic Republic	Both	65-69 years	6.87	5.54	8.35	Lao People's Democratic Republic	Male	80-84	10.99	8.87	13.41	Lao People's Democratic Republic	Female	80-84	16.80	13.56	20.30
Lao People's Democra	Both	70-74 years	9.33	7.28	11.68	Lao People's Democra	Male	85-89	12.31	10.04	14.95	Lao People's Democra	Female	85-89	18.56	15.27	22.43

tic Republic						tic Republic						tic Republic					
Lao People's Democratic Republic	Both	75-79 years	11.94	9.50	14.57	Lao People's Democratic Republic	Male	90-94	13.33	10.98	16.06	Lao People's Democratic Republic	Female	90-94	19.57	16.03	23.29
Latvia	Both	40-44 years	0.49	0.36	0.65	Latvia	Male	40-44 years	0.38	0.28	0.51	Latvia	Female	40-44 years	0.58	0.42	0.79
Latvia	Both	45-49 years	1.03	0.80	1.31	Latvia	Male	45-49 years	0.79	0.61	0.99	Latvia	Female	45-49 years	1.26	0.96	1.60
Latvia	Both	50-54 years	1.84	1.41	2.35	Latvia	Male	50-54 years	1.37	1.05	1.74	Latvia	Female	50-54 years	2.26	1.69	2.93
Latvia	Both	55-59 years	2.95	2.27	3.61	Latvia	Male	55-59 years	2.16	1.70	2.64	Latvia	Female	55-59 years	3.61	2.77	4.48
Latvia	Both	60-64 years	4.39	3.44	5.43	Latvia	Male	60-64 years	3.15	2.48	3.85	Latvia	Female	60-64 years	5.34	4.15	6.67
Latvia	Both	65-69 years	6.73	5.43	8.15	Latvia	Male	65-69 years	4.67	3.78	5.65	Latvia	Female	65-69 years	8.09	6.48	9.87
Latvia	Both	70-74 years	9.78	7.56	12.36	Latvia	Male	70-74 years	6.61	5.12	8.32	Latvia	Female	70-74 years	11.58	8.96	14.68
Latvia	Both	75-79 years	12.94	10.30	16.10	Latvia	Male	75-79 years	8.66	6.98	10.68	Latvia	Female	75-79 years	14.96	11.91	18.75
Latvia	Both	All ages	3.28	2.80	3.77	Latvia	Male	All ages	1.74	1.49	2.00	Latvia	Female	All ages	4.55	3.88	5.25
Latvia	Both	80-84	15.92	13.06	19.29	Latvia	Male	80-84	10.71	8.60	13.10	Latvia	Female	80-84	17.95	14.69	21.71
Latvia	Both	85-89	18.28	15.10	21.82	Latvia	Male	85-89	12.55	10.21	15.27	Latvia	Female	85-89	20.10	16.51	24.06
Latvia	Both	90-94	20.15	16.74	23.90	Latvia	Male	90-94	14.23	11.54	17.19	Latvia	Female	90-94	21.49	17.80	25.37
Lebanon	Both	60-64 years	4.26	3.32	5.24	Lebanon	Male	60-64 years	3.61	2.82	4.50	Lebanon	Female	40-44 years	0.52	0.38	0.71
Lebanon	Both	65-69 years	6.38	5.14	7.84	Lebanon	Male	65-69 years	5.37	4.31	6.63	Lebanon	Female	45-49 years	1.14	0.86	1.44
Lebanon	Both	70-74 years	9.08	6.99	11.49	Lebanon	Male	70-74 years	7.63	5.90	9.64	Lebanon	Female	50-54 years	2.04	1.52	2.64
Lebanon	Both	75-79 years	11.79	9.43	14.47	Lebanon	Male	75-79 years	9.98	7.90	12.26	Lebanon	Female	55-59 years	3.25	2.51	4.00
Lebanon	Both	All ages	1.44	1.24	1.67	Lebanon	Male	All ages	1.15	0.99	1.32	Lebanon	Female	60-64 years	4.79	3.69	5.90
Lebanon	Both	80-84	14.33	11.67	17.42	Lebanon	Male	80-84	12.24	9.85	15.14	Lebanon	Female	65-69 years	7.22	5.79	8.85
Lebanon	Both	85-89	16.29	13.38	19.60	Lebanon	Male	85-89	14.12	11.50	17.21	Lebanon	Female	70-74 years	10.30	7.82	13.12
Lebanon	Both	90-94	17.86	14.83	21.32	Lebanon	Male	90-94	15.65	12.92	18.78	Lebanon	Female	75-79 years	13.29	10.63	16.40
Lebanon	Both	40-44 years	0.47	0.35	0.63	Lebanon	Male	40-44 years	0.41	0.30	0.55	Lebanon	Female	All ages	1.72	1.47	1.98

Lebanon	Both	45-49 years	1.02	0.79	1.29	Lebanon	Male	45-49 years	0.88	0.68	1.12	Lebanon	Female	80-84	15.97	12.93	19.41
Lebanon	Both	50-54 years	1.83	1.37	2.34	Lebanon	Male	50-54 years	1.57	1.18	2.00	Lebanon	Female	85-89	17.99	14.76	21.64
Lebanon	Both	55-59 years	2.90	2.24	3.55	Lebanon	Male	55-59 years	2.48	1.92	3.03	Lebanon	Female	90-94	19.41	16.13	23.28
Lesotho	Both	40-44 years	0.39	0.29	0.52	Lesotho	Male	40-44 years	0.33	0.24	0.45	Lesotho	Female	40-44 years	0.45	0.33	0.60
Lesotho	Both	45-49 years	0.88	0.69	1.12	Lesotho	Male	45-49 years	0.74	0.57	0.93	Lesotho	Female	45-49 years	1.02	0.79	1.31
Lesotho	Both	50-54 years	1.64	1.25	2.12	Lesotho	Male	50-54 years	1.35	1.03	1.73	Lesotho	Female	50-54 years	1.89	1.41	2.49
Lesotho	Both	55-59 years	2.68	2.08	3.30	Lesotho	Male	55-59 years	2.16	1.70	2.61	Lesotho	Female	55-59 years	3.07	2.35	3.78
Lesotho	Both	60-64 years	3.99	3.14	4.95	Lesotho	Male	60-64 years	3.17	2.49	3.94	Lesotho	Female	60-64 years	4.58	3.57	5.70
Lesotho	Both	65-69 years	6.11	4.89	7.49	Lesotho	Male	65-69 years	4.74	3.81	5.81	Lesotho	Female	65-69 years	7.03	5.59	8.63
Lesotho	Both	70-74 years	8.95	6.88	11.24	Lesotho	Male	70-74 years	6.76	5.16	8.47	Lesotho	Female	70-74 years	10.22	7.84	12.93
Lesotho	Both	75-79 years	11.92	9.43	14.76	Lesotho	Male	75-79 years	8.88	7.08	10.83	Lesotho	Female	75-79 years	13.41	10.58	16.56
Lesotho	Both	All ages	0.74	0.63	0.85	Lesotho	Male	All ages	0.46	0.39	0.53	Lesotho	Female	All ages	1.01	0.86	1.16
Lesotho	Both	80-84	14.81	12.09	18.21	Lesotho	Male	80-84	10.92	8.79	13.35	Lesotho	Female	80-84	16.30	13.30	20.12
Lesotho	Both	85-89	16.99	13.90	20.65	Lesotho	Male	85-89	12.61	10.30	15.33	Lesotho	Female	85-89	18.32	15.00	22.40
Lesotho	Both	90-94	18.39	15.25	21.98	Lesotho	Male	90-94	13.98	11.37	16.77	Lesotho	Female	90-94	19.53	16.19	23.43
Liberia	Both	45-49 years	0.69	0.52	0.88	Liberia	Male	40-44 years	0.29	0.21	0.39	Liberia	Female	45-49 years	0.74	0.56	0.96
Liberia	Both	50-54 years	1.25	0.94	1.61	Liberia	Male	45-49 years	0.64	0.48	0.81	Liberia	Female	50-54 years	1.36	1.01	1.77
Liberia	Both	55-59 years	2.02	1.58	2.48	Liberia	Male	50-54 years	1.16	0.86	1.49	Liberia	Female	55-59 years	2.21	1.71	2.71
Liberia	Both	60-64 years	2.99	2.36	3.76	Liberia	Male	55-59 years	1.86	1.44	2.29	Liberia	Female	60-64 years	3.29	2.59	4.12
Liberia	Both	65-69 years	4.50	3.57	5.55	Liberia	Male	60-64 years	2.73	2.12	3.46	Liberia	Female	65-69 years	4.99	3.94	6.15
Liberia	Both	70-74 years	6.47	4.92	8.24	Liberia	Male	65-69 years	4.05	3.22	5.01	Liberia	Female	70-74 years	7.19	5.49	9.19
Liberia	Both	75-79 years	8.54	6.90	10.40	Liberia	Male	70-74 years	5.73	4.41	7.26	Liberia	Female	75-79 years	9.48	7.60	11.78
Liberia	Both	All ages	0.38	0.32	0.43	Liberia	Male	75-79 years	7.54	5.98	9.18	Liberia	Female	All ages	0.41	0.35	0.47
Liberia	Both	80-84	10.51	8.45	12.90	Liberia	Male	All ages	0.35	0.30	0.40	Liberia	Female	80-84	11.65	9.33	14.29

Liberia	Both	85-89	12.07	9.78	14.59	Liberia	Male	80-84	9.32	7.43	11.39	Liberia	Female	85-89	13.32	10.79	16.18
Liberia	Both	90-94	13.36	11.06	16.13	Liberia	Male	85-89	10.83	8.78	13.13	Liberia	Female	90-94	14.55	11.97	17.62
Liberia	Both	40-44 years	0.31	0.22	0.42	Liberia	Male	90-94	12.08	9.83	14.70	Liberia	Female	40-44 years	0.34	0.24	0.46
Libya	Both	40-44 years	0.40	0.28	0.54	Libya	Male	40-44 years	0.35	0.25	0.48	Libya	Female	40-44 years	0.44	0.32	0.61
Libya	Both	45-49 years	0.84	0.64	1.05	Libya	Male	45-49 years	0.74	0.56	0.93	Libya	Female	45-49 years	0.95	0.73	1.21
Libya	Both	50-54 years	1.49	1.13	1.89	Libya	Male	50-54 years	1.30	0.99	1.65	Libya	Female	50-54 years	1.68	1.27	2.17
Libya	Both	55-59 years	2.37	1.86	2.88	Libya	Male	55-59 years	2.06	1.62	2.52	Libya	Female	55-59 years	2.68	2.10	3.32
Libya	Both	60-64 years	3.48	2.74	4.31	Libya	Male	60-64 years	3.02	2.37	3.80	Libya	Female	60-64 years	3.96	3.11	4.89
Libya	Both	65-69 years	5.20	4.20	6.37	Libya	Male	65-69 years	4.47	3.62	5.49	Libya	Female	65-69 years	5.93	4.76	7.28
Libya	Both	70-74 years	7.34	5.63	9.20	Libya	Male	70-74 years	6.31	4.87	7.97	Libya	Female	70-74 years	8.41	6.48	10.56
Libya	Both	75-79 years	9.50	7.62	11.58	Libya	Male	75-79 years	8.23	6.52	10.01	Libya	Female	75-79 years	10.92	8.66	13.39
Libya	Both	All ages	0.82	0.71	0.93	Libya	Male	All ages	0.71	0.62	0.82	Libya	Female	All ages	0.93	0.81	1.06
Libya	Both	80-84	11.62	9.39	14.32	Libya	Male	80-84	10.11	8.10	12.45	Libya	Female	80-84	13.26	10.62	16.36
Libya	Both	85-89	13.48	11.11	16.21	Libya	Male	85-89	11.76	9.65	14.24	Libya	Female	85-89	15.07	12.34	18.16
Libya	Both	90-94	15.01	12.39	18.00	Libya	Male	90-94	13.22	10.90	15.95	Libya	Female	90-94	16.42	13.52	19.73
Lithuania	Both	80-84	15.37	12.52	18.80	Lithuania	Male	80-84	10.37	8.26	12.65	Lithuania	Female	40-44 years	0.56	0.41	0.74
Lithuania	Both	85-89	17.67	14.51	21.25	Lithuania	Male	85-89	12.19	9.95	14.70	Lithuania	Female	45-49 years	1.21	0.93	1.54
Lithuania	Both	90-94	19.51	16.23	23.19	Lithuania	Male	90-94	13.90	11.42	16.63	Lithuania	Female	50-54 years	2.17	1.63	2.79
Lithuania	Both	40-44 years	0.47	0.34	0.63	Lithuania	Male	40-44 years	0.37	0.27	0.50	Lithuania	Female	55-59 years	3.48	2.69	4.34
Lithuania	Both	45-49 years	1.00	0.78	1.25	Lithuania	Male	45-49 years	0.77	0.59	0.95	Lithuania	Female	60-64 years	5.19	4.04	6.47
Lithuania	Both	50-54 years	1.78	1.35	2.25	Lithuania	Male	50-54 years	1.33	1.02	1.67	Lithuania	Female	65-69 years	7.85	6.29	9.59
Lithuania	Both	55-59 years	2.85	2.22	3.50	Lithuania	Male	55-59 years	2.09	1.65	2.57	Lithuania	Female	70-74 years	11.21	8.62	14.29
Lithuania	Both	60-64 years	4.26	3.34	5.28	Lithuania	Male	60-64 years	3.05	2.41	3.80	Lithuania	Female	75-79 years	14.49	11.40	17.95
Lithuania	Both	65-69 years	6.51	5.24	7.95	Lithuania	Male	65-69 years	4.51	3.64	5.53	Lithuania	Female	All ages	4.31	3.66	5.00

Lithuania	Both	70-74 years	9.46	7.29	11.99	Lithuania	Male	70-74 years	6.39	4.96	7.97	Lithuania	Female	80-84	17.43	14.15	21.27
Lithuania	Both	75-79 years	12.48	9.93	15.38	Lithuania	Male	75-79 years	8.37	6.70	10.12	Lithuania	Female	85-89	19.58	16.10	23.66
Lithuania	Both	All ages	3.13	2.67	3.61	Lithuania	Male	All ages	1.70	1.46	1.94	Lithuania	Female	90-94	21.02	17.48	24.98
Luxembourg	Both	40-44 years	1.14	0.84	1.47	Luxembourg	Male	40-44 years	0.60	0.44	0.81	Luxembourg	Female	40-44 years	1.67	1.23	2.15
Luxembourg	Both	45-49 years	1.96	1.57	2.41	Luxembourg	Male	45-49 years	1.26	0.97	1.59	Luxembourg	Female	45-49 years	2.68	2.10	3.35
Luxembourg	Both	50-54 years	2.92	2.27	3.67	Luxembourg	Male	50-54 years	2.24	1.70	2.90	Luxembourg	Female	50-54 years	3.65	2.84	4.57
Luxembourg	Both	55-59 years	4.28	3.39	5.23	Luxembourg	Male	55-59 years	3.74	2.93	4.61	Luxembourg	Female	55-59 years	4.85	3.88	5.94
Luxembourg	Both	60-64 years	6.23	5.01	7.54	Luxembourg	Male	60-64 years	5.78	4.51	7.21	Luxembourg	Female	60-64 years	6.69	5.39	8.08
Luxembourg	Both	65-69 years	9.68	7.88	11.56	Luxembourg	Male	65-69 years	8.83	7.10	10.62	Luxembourg	Female	65-69 years	10.53	8.55	12.57
Luxembourg	Both	70-74 years	14.21	11.15	17.85	Luxembourg	Male	70-74 years	12.58	9.76	15.80	Luxembourg	Female	70-74 years	15.78	12.33	19.82
Luxembourg	Both	75-79 years	18.68	15.05	22.85	Luxembourg	Male	75-79 years	16.06	12.77	19.74	Luxembourg	Female	75-79 years	20.81	16.85	25.43
Luxembourg	Both	All ages	3.72	3.24	4.21	Luxembourg	Male	All ages	2.91	2.50	3.34	Luxembourg	Female	All ages	4.50	3.90	5.11
Luxembourg	Both	80-84	22.74	18.78	27.35	Luxembourg	Male	80-84	19.10	15.57	22.97	Luxembourg	Female	80-84	25.28	20.96	30.49
Luxembourg	Both	85-89	25.97	21.74	31.05	Luxembourg	Male	85-89	21.34	17.64	25.51	Luxembourg	Female	85-89	28.60	23.84	34.45
Luxembourg	Both	90-94	28.45	24.10	33.27	Luxembourg	Male	90-94	22.75	19.07	26.87	Luxembourg	Female	90-94	30.70	25.93	35.98
Madagascar	Both	75-79 years	8.02	6.43	9.76	Madagascar	Male	40-44 years	0.26	0.19	0.35	Madagascar	Female	75-79 years	9.13	7.37	11.27
Madagascar	Both	All ages	0.33	0.28	0.38	Madagascar	Male	45-49 years	0.57	0.43	0.73	Madagascar	Female	All ages	0.39	0.33	0.44
Madagascar	Both	80-84	9.96	8.13	12.31	Madagascar	Male	50-54 years	1.03	0.77	1.32	Madagascar	Female	80-84	11.29	9.18	13.92
Madagascar	Both	85-89	11.60	9.44	13.95	Madagascar	Male	55-59 years	1.65	1.29	2.02	Madagascar	Female	85-89	13.02	10.62	15.78
Madagascar	Both	90-94	13.02	10.69	15.49	Madagascar	Male	60-64 years	2.43	1.91	3.06	Madagascar	Female	90-94	14.37	11.87	17.19
Madagascar	Both	40-44 years	0.30	0.21	0.39	Madagascar	Male	65-69 years	3.59	2.86	4.44	Madagascar	Female	40-44 years	0.33	0.24	0.45
Madagascar	Both	45-49 years	0.65	0.49	0.82	Madagascar	Male	70-74 years	5.07	3.87	6.35	Madagascar	Female	45-49 years	0.72	0.55	0.92
Madagascar	Both	50-54 years	1.17	0.89	1.50	Madagascar	Male	75-79 years	6.67	5.31	8.13	Madagascar	Female	50-54 years	1.31	0.99	1.67
Madagascar	Both	55-59 years	1.89	1.48	2.32	Madagascar	Male	All ages	0.28	0.24	0.32	Madagascar	Female	55-59 years	2.13	1.65	2.62

Madagas car	Both	60-64 years	2.81	2.20	3.53	Madagas car	Male	80-84	8.28	6.71	10.29	Madagas car	Female	60-64 years	3.18	2.49	4.03
Madagas car	Both	65-69 years	4.22	3.36	5.18	Madagas car	Male	85-89	9.75	7.93	11.73	Madagas car	Female	65-69 years	4.81	3.83	5.95
Madagas car	Both	70-74 years	6.04	4.65	7.58	Madagas car	Male	90-94	11.09	9.09	13.24	Madagas car	Female	70-74 years	6.92	5.32	8.74
Malawi	Both	40-44 years	0.32	0.23	0.43	Malawi	Male	40-44 years	0.29	0.21	0.39	Malawi	Female	40-44 years	0.36	0.26	0.48
Malawi	Both	45-49 years	0.71	0.54	0.90	Malawi	Male	45-49 years	0.63	0.48	0.81	Malawi	Female	45-49 years	0.79	0.61	1.01
Malawi	Both	50-54 years	1.30	0.98	1.66	Malawi	Male	50-54 years	1.13	0.86	1.48	Malawi	Female	50-54 years	1.45	1.08	1.86
Malawi	Both	55-59 years	2.10	1.63	2.58	Malawi	Male	55-59 years	1.82	1.41	2.22	Malawi	Female	55-59 years	2.36	1.83	2.91
Malawi	Both	60-64 years	3.13	2.46	3.91	Malawi	Male	60-64 years	2.67	2.11	3.35	Malawi	Female	60-64 years	3.53	2.77	4.43
Malawi	Both	65-69 years	4.73	3.75	5.79	Malawi	Male	65-69 years	3.96	3.14	4.90	Malawi	Female	65-69 years	5.36	4.24	6.60
Malawi	Both	70-74 years	6.84	5.21	8.50	Malawi	Male	70-74 years	5.63	4.26	7.06	Malawi	Female	70-74 years	7.73	5.92	9.67
Malawi	Both	75-79 years	9.09	7.17	11.07	Malawi	Male	75-79 years	7.40	5.81	8.95	Malawi	Female	75-79 years	10.19	8.09	12.39
Malawi	Both	All ages	0.37	0.32	0.43	Malawi	Male	All ages	0.28	0.24	0.33	Malawi	Female	All ages	0.46	0.39	0.53
Malawi	Both	80-84	11.35	9.16	13.96	Malawi	Male	80-84	9.16	7.43	11.21	Malawi	Female	80-84	12.52	10.02	15.46
Malawi	Both	85-89	13.24	10.73	16.06	Malawi	Male	85-89	10.68	8.70	12.91	Malawi	Female	85-89	14.28	11.56	17.41
Malawi	Both	90-94	14.78	12.17	17.75	Malawi	Male	90-94	11.99	9.81	14.40	Malawi	Female	90-94	15.54	12.82	18.70
Malaysia	Both	40-44 years	0.54	0.40	0.72	Malaysia	Male	40-44 years	0.48	0.35	0.64	Malaysia	Female	40-44 years	0.61	0.44	0.81
Malaysia	Both	45-49 years	1.18	0.91	1.49	Malaysia	Male	45-49 years	1.02	0.79	1.28	Malaysia	Female	45-49 years	1.36	1.04	1.72
Malaysia	Both	50-54 years	2.13	1.61	2.72	Malaysia	Male	50-54 years	1.80	1.38	2.29	Malaysia	Female	50-54 years	2.47	1.85	3.20
Malaysia	Both	55-59 years	3.33	2.60	4.05	Malaysia	Male	55-59 years	2.78	2.17	3.37	Malaysia	Female	55-59 years	3.89	3.03	4.78
Malaysia	Both	60-64 years	4.75	3.75	5.78	Malaysia	Male	60-64 years	3.92	3.08	4.79	Malaysia	Female	60-64 years	5.57	4.41	6.81
Malaysia	Both	65-69 years	6.77	5.50	8.18	Malaysia	Male	65-69 years	5.46	4.40	6.60	Malaysia	Female	65-69 years	8.01	6.47	9.72
Malaysia	Both	70-74 years	9.13	7.22	11.31	Malaysia	Male	70-74 years	7.31	5.77	9.13	Malaysia	Female	70-74 years	11.00	8.69	13.74
Malaysia	Both	75-79 years	11.62	9.39	14.22	Malaysia	Male	75-79 years	9.19	7.39	11.22	Malaysia	Female	75-79 years	13.98	11.26	17.21
Malaysia	Both	All ages	1.23	1.06	1.40	Malaysia	Male	All ages	0.98	0.83	1.12	Malaysia	Female	All ages	1.49	1.29	1.71

Malaysia	Both	80-84	14.01	11.53	16.92	Malaysia	Male	80-84	11.00	9.00	13.36	Malaysia	Female	80-84	16.74	13.66	20.31
Malaysia	Both	85-89	15.88	13.17	19.04	Malaysia	Male	85-89	12.53	10.36	14.98	Malaysia	Female	85-89	18.84	15.56	22.62
Malaysia	Both	90-94	17.07	14.21	20.18	Malaysia	Male	90-94	13.84	11.56	16.42	Malaysia	Female	90-94	20.36	16.90	24.26
Maldives	Both	40-44 years	0.53	0.39	0.70	Maldives	Male	40-44 years	0.48	0.35	0.64	Maldives	Female	40-44 years	0.61	0.45	0.80
Maldives	Both	45-49 years	1.16	0.90	1.45	Maldives	Male	45-49 years	1.03	0.80	1.29	Maldives	Female	45-49 years	1.36	1.05	1.72
Maldives	Both	50-54 years	2.10	1.60	2.68	Maldives	Male	50-54 years	1.82	1.38	2.33	Maldives	Female	50-54 years	2.48	1.89	3.18
Maldives	Both	55-59 years	3.31	2.59	4.02	Maldives	Male	55-59 years	2.82	2.22	3.43	Maldives	Female	55-59 years	3.90	3.02	4.77
Maldives	Both	60-64 years	4.74	3.72	5.82	Maldives	Male	60-64 years	3.99	3.17	4.92	Maldives	Female	60-64 years	5.59	4.31	6.94
Maldives	Both	65-69 years	6.69	5.43	8.12	Maldives	Male	65-69 years	5.54	4.48	6.78	Maldives	Female	65-69 years	8.02	6.45	9.78
Maldives	Both	70-74 years	9.14	7.16	11.42	Maldives	Male	70-74 years	7.37	5.75	9.22	Maldives	Female	70-74 years	11.00	8.61	13.84
Maldives	Both	75-79 years	11.56	9.24	14.13	Maldives	Male	75-79 years	9.20	7.34	11.28	Maldives	Female	75-79 years	13.95	11.10	17.04
Maldives	Both	All ages	0.85	0.73	0.96	Maldives	Male	All ages	0.65	0.56	0.75	Maldives	Female	All ages	1.14	0.98	1.30
Maldives	Both	80-84	13.62	11.04	16.46	Maldives	Male	80-84	10.92	8.75	13.25	Maldives	Female	80-84	16.64	13.45	20.21
Maldives	Both	85-89	15.16	12.47	18.06	Maldives	Male	85-89	12.33	10.05	14.84	Maldives	Female	85-89	18.59	15.28	22.38
Maldives	Both	90-94	16.36	13.59	19.55	Maldives	Male	90-94	13.50	11.07	16.31	Maldives	Female	90-94	19.89	16.59	23.56
Mali	Both	40-44 years	0.29	0.21	0.38	Mali	Male	40-44 years	0.27	0.19	0.36	Mali	Female	40-44 years	0.31	0.22	0.41
Mali	Both	45-49 years	0.63	0.48	0.80	Mali	Male	45-49 years	0.58	0.45	0.74	Mali	Female	45-49 years	0.68	0.52	0.87
Mali	Both	50-54 years	1.14	0.87	1.47	Mali	Male	50-54 years	1.05	0.80	1.36	Mali	Female	50-54 years	1.23	0.93	1.58
Mali	Both	55-59 years	1.84	1.43	2.25	Mali	Male	55-59 years	1.68	1.29	2.08	Mali	Female	55-59 years	2.00	1.56	2.46
Mali	Both	60-64 years	2.72	2.12	3.40	Mali	Male	60-64 years	2.47	1.92	3.10	Mali	Female	60-64 years	2.98	2.32	3.75
Mali	Both	65-69 years	4.05	3.23	4.96	Mali	Male	65-69 years	3.65	2.91	4.51	Mali	Female	65-69 years	4.49	3.61	5.49
Mali	Both	70-74 years	5.78	4.44	7.23	Mali	Male	70-74 years	5.17	3.95	6.47	Mali	Female	70-74 years	6.44	4.96	8.15
Mali	Both	75-79 years	7.62	6.10	9.32	Mali	Male	75-79 years	6.81	5.46	8.28	Mali	Female	75-79 years	8.51	6.84	10.38
Mali	Both	All ages	0.32	0.27	0.37	Mali	Male	All ages	0.30	0.26	0.35	Mali	Female	All ages	0.33	0.29	0.38

Mali	Both	80-84	9.46	7.69	11.55	Mali	Male	80-84	8.46	6.83	10.38	Mali	Female	80-84	10.54	8.49	12.95
Mali	Both	85-89	11.04	9.04	13.35	Mali	Male	85-89	9.94	8.08	12.00	Mali	Female	85-89	12.21	9.98	14.87
Mali	Both	90-94	12.42	10.27	14.94	Mali	Male	90-94	11.27	9.33	13.70	Mali	Female	90-94	13.57	11.18	16.29
Malta	Both	40-44 years	1.19	0.89	1.54	Malta	Male	40-44 years	0.63	0.46	0.85	Malta	Female	40-44 years	1.77	1.33	2.29
Malta	Both	45-49 years	2.07	1.63	2.60	Malta	Male	45-49 years	1.33	1.02	1.71	Malta	Female	45-49 years	2.83	2.21	3.55
Malta	Both	50-54 years	3.10	2.41	3.95	Malta	Male	50-54 years	2.38	1.80	3.09	Malta	Female	50-54 years	3.83	2.95	4.85
Malta	Both	55-59 years	4.53	3.56	5.50	Malta	Male	55-59 years	3.98	3.06	4.89	Malta	Female	55-59 years	5.08	4.04	6.23
Malta	Both	60-64 years	6.57	5.25	8.09	Malta	Male	60-64 years	6.13	4.78	7.67	Malta	Female	60-64 years	7.00	5.59	8.60
Malta	Both	65-69 years	10.22	8.35	12.32	Malta	Male	65-69 years	9.35	7.55	11.49	Malta	Female	65-69 years	11.06	8.92	13.26
Malta	Both	70-74 years	14.95	11.56	18.64	Malta	Male	70-74 years	13.23	10.21	16.52	Malta	Female	70-74 years	16.52	12.72	20.75
Malta	Both	75-79 years	19.37	15.43	23.68	Malta	Male	75-79 years	16.71	13.26	20.33	Malta	Female	75-79 years	21.59	17.23	26.46
Malta	Both	All ages	4.97	4.24	5.68	Malta	Male	All ages	3.95	3.36	4.59	Malta	Female	All ages	5.94	5.09	6.82
Malta	Both	80-84	23.29	19.00	28.20	Malta	Male	80-84	19.60	15.93	23.61	Malta	Female	80-84	25.94	21.17	31.16
Malta	Both	85-89	26.36	21.88	31.41	Malta	Male	85-89	21.55	17.88	25.84	Malta	Female	85-89	28.95	23.98	34.62
Malta	Both	90-94	28.20	23.76	33.35	Malta	Male	90-94	22.58	18.82	26.79	Malta	Female	90-94	30.61	25.85	36.15
Marshall Islands	Both	40-44 years	0.58	0.44	0.78	Marshall Islands	Male	40-44 years	0.53	0.39	0.71	Marshall Islands	Female	40-44 years	0.65	0.48	0.86
Marshall Islands	Both	45-49 years	1.27	0.99	1.60	Marshall Islands	Male	45-49 years	1.13	0.88	1.42	Marshall Islands	Female	45-49 years	1.42	1.11	1.81
Marshall Islands	Both	50-54 years	2.27	1.74	2.92	Marshall Islands	Male	50-54 years	1.99	1.50	2.56	Marshall Islands	Female	50-54 years	2.56	1.96	3.27
Marshall Islands	Both	55-59 years	3.50	2.77	4.28	Marshall Islands	Male	55-59 years	3.05	2.42	3.76	Marshall Islands	Female	55-59 years	3.95	3.07	4.84
Marshall Islands	Both	60-64 years	4.91	3.88	6.06	Marshall Islands	Male	60-64 years	4.25	3.34	5.25	Marshall Islands	Female	60-64 years	5.58	4.36	6.88
Marshall Islands	Both	65-69 years	6.94	5.64	8.45	Marshall Islands	Male	65-69 years	5.93	4.76	7.25	Marshall Islands	Female	65-69 years	8.03	6.47	9.75
Marshall Islands	Both	70-74 years	9.44	7.40	11.79	Marshall Islands	Male	70-74 years	7.96	6.22	9.96	Marshall Islands	Female	70-74 years	11.11	8.66	13.96
Marshall Islands	Both	75-79 years	11.85	9.51	14.52	Marshall Islands	Male	75-79 years	9.90	7.97	12.07	Marshall Islands	Female	75-79 years	14.07	11.30	17.14
Marshall Islands	Both	All ages	0.83	0.71	0.95	Marshall Islands	Male	All ages	0.72	0.62	0.84	Marshall Islands	Female	All ages	0.94	0.80	1.08

Marshall Islands	Both	80-84	13.95	11.48	16.87	Marshall Islands	Male	80-84	11.60	9.46	14.08	Marshall Islands	Female	80-84	16.63	13.65	20.26
Marshall Islands	Both	85-89	15.34	12.62	18.41	Marshall Islands	Male	85-89	12.87	10.57	15.49	Marshall Islands	Female	85-89	18.29	15.08	22.05
Marshall Islands	Both	90-94	16.23	13.56	19.35	Marshall Islands	Male	90-94	13.79	11.52	16.54	Marshall Islands	Female	90-94	19.18	15.88	22.81
Mauritan ia	Both	40-44 years	0.30	0.22	0.40	Mauritan ia	Male	85-89	10.39	8.45	12.42	Mauritan ia	Female	40-44 years	0.32	0.23	0.43
Mauritan ia	Both	45-49 years	0.65	0.50	0.83	Mauritan ia	Male	90-94	11.79	9.64	14.20	Mauritan ia	Female	45-49 years	0.70	0.54	0.90
Mauritan ia	Both	50-54 years	1.18	0.90	1.52	Mauritan ia	Male	40-44 years	0.27	0.20	0.37	Mauritan ia	Female	50-54 years	1.27	0.97	1.64
Mauritan ia	Both	55-59 years	1.91	1.49	2.33	Mauritan ia	Male	45-49 years	0.60	0.46	0.77	Mauritan ia	Female	55-59 years	2.07	1.63	2.60
Mauritan ia	Both	60-64 years	2.84	2.24	3.53	Mauritan ia	Male	50-54 years	1.08	0.82	1.39	Mauritan ia	Female	60-64 years	3.11	2.43	3.91
Mauritan ia	Both	65-69 years	4.26	3.41	5.21	Mauritan ia	Male	55-59 years	1.75	1.36	2.14	Mauritan ia	Female	65-69 years	4.70	3.75	5.78
Mauritan ia	Both	70-74 years	6.08	4.63	7.66	Mauritan ia	Male	60-64 years	2.58	2.02	3.24	Mauritan ia	Female	70-74 years	6.76	5.17	8.48
Mauritan ia	Both	75-79 years	8.00	6.42	9.75	Mauritan ia	Male	65-69 years	3.83	3.10	4.69	Mauritan ia	Female	75-79 years	8.93	7.16	10.91
Mauritan ia	Both	All ages	0.46	0.40	0.53	Mauritan ia	Male	70-74 years	5.42	4.12	6.87	Mauritan ia	Female	All ages	0.49	0.42	0.56
Mauritan ia	Both	80-84	9.91	7.93	12.12	Mauritan ia	Male	75-79 years	7.13	5.66	8.71	Mauritan ia	Female	80-84	11.05	8.86	13.65
Mauritan ia	Both	85-89	11.56	9.51	13.90	Mauritan ia	Male	All ages	0.43	0.37	0.49	Mauritan ia	Female	85-89	12.83	10.64	15.60
Mauritan ia	Both	90-94	13.03	10.76	15.63	Mauritan ia	Male	80-84	8.84	7.11	10.77	Mauritan ia	Female	90-94	14.28	11.81	17.21
Mauritius	Both	40-44 years	0.62	0.45	0.84	Mauritius	Male	40-44 years	0.54	0.39	0.74	Mauritius	Female	40-44 years	0.70	0.52	0.94
Mauritius	Both	45-49 years	1.37	1.06	1.72	Mauritius	Male	45-49 years	1.16	0.90	1.46	Mauritius	Female	45-49 years	1.57	1.22	1.98
Mauritius	Both	50-54 years	2.47	1.87	3.18	Mauritius	Male	50-54 years	2.07	1.58	2.67	Mauritius	Female	50-54 years	2.86	2.16	3.66
Mauritius	Both	55-59 years	3.86	3.04	4.72	Mauritius	Male	55-59 years	3.20	2.53	3.91	Mauritius	Female	55-59 years	4.49	3.52	5.52
Mauritius	Both	60-64 years	5.50	4.33	6.79	Mauritius	Male	60-64 years	4.50	3.55	5.54	Mauritius	Female	60-64 years	6.44	4.98	7.97
Mauritius	Both	65-69 years	7.89	6.39	9.67	Mauritius	Male	65-69 years	6.27	5.00	7.61	Mauritius	Female	65-69 years	9.30	7.50	11.50
Mauritius	Both	70-74 years	10.88	8.52	13.54	Mauritius	Male	70-74 years	8.41	6.62	10.47	Mauritius	Female	70-74 years	12.82	9.99	16.04
Mauritius	Both	75-79 years	13.84	11.09	16.92	Mauritius	Male	75-79 years	10.55	8.43	12.97	Mauritius	Female	75-79 years	16.20	12.97	19.96
Mauritius	Both	All ages	2.33	2.01	2.68	Mauritius	Male	All ages	1.71	1.46	1.96	Mauritius	Female	All ages	2.93	2.53	3.40

Mauritius	Both	80-84	16.67	13.58	20.07	Mauritius	Male	80-84	12.54	10.07	15.33	Mauritius	Female	80-84	19.19	15.62	23.06
Mauritius	Both	85-89	18.70	15.49	22.27	Mauritius	Male	85-89	14.15	11.56	16.91	Mauritius	Female	85-89	21.28	17.60	25.52
Mauritius	Both	90-94	20.14	16.80	23.85	Mauritius	Male	90-94	15.42	12.76	18.42	Mauritius	Female	90-94	22.56	18.79	26.70
Mexico	Both	40-44 years	0.31	0.23	0.42	Mexico	Male	40-44 years	0.22	0.15	0.29	Mexico	Female	40-44 years	0.40	0.29	0.53
Mexico	Both	45-49 years	0.69	0.53	0.87	Mexico	Male	45-49 years	0.46	0.36	0.58	Mexico	Female	45-49 years	0.89	0.69	1.13
Mexico	Both	50-54 years	1.26	0.95	1.62	Mexico	Male	50-54 years	0.82	0.62	1.04	Mexico	Female	50-54 years	1.66	1.25	2.13
Mexico	Both	55-59 years	2.08	1.62	2.53	Mexico	Male	55-59 years	1.33	1.05	1.62	Mexico	Female	55-59 years	2.74	2.14	3.33
Mexico	Both	60-64 years	3.19	2.52	3.94	Mexico	Male	60-64 years	2.03	1.60	2.51	Mexico	Female	60-64 years	4.21	3.33	5.21
Mexico	Both	65-69 years	5.05	4.06	6.18	Mexico	Male	65-69 years	3.18	2.54	3.90	Mexico	Female	65-69 years	6.71	5.40	8.20
Mexico	Both	70-74 years	7.54	5.83	9.45	Mexico	Male	70-74 years	4.71	3.60	5.86	Mexico	Female	70-74 years	9.96	7.74	12.46
Mexico	Both	75-79 years	10.02	7.98	12.20	Mexico	Male	75-79 years	6.30	5.02	7.71	Mexico	Female	75-79 years	13.14	10.50	16.05
Mexico	Both	All ages	1.05	0.91	1.19	Mexico	Male	All ages	0.64	0.56	0.74	Mexico	Female	All ages	1.41	1.23	1.62
Mexico	Both	80-84	12.38	10.19	15.05	Mexico	Male	80-84	7.86	6.39	9.67	Mexico	Female	80-84	15.99	13.19	19.41
Mexico	Both	85-89	14.20	11.74	17.05	Mexico	Male	85-89	9.28	7.63	11.18	Mexico	Female	85-89	17.99	14.84	21.60
Mexico	Both	90-94	15.46	12.90	18.38	Mexico	Male	90-94	10.64	8.73	12.81	Mexico	Female	90-94	19.27	16.18	22.84
Micronesia (Federated States of)	Both	40-44 years	0.58	0.43	0.77	Micronesia (Federated States of)	Male	40-44 years	0.52	0.39	0.69	Micronesia (Federated States of)	Female	90-94	19.04	15.81	22.49
Micronesia (Federated States of)	Both	45-49 years	1.27	1.00	1.59	Micronesia (Federated States of)	Male	45-49 years	1.12	0.87	1.41	Micronesia (Federated States of)	Female	40-44 years	0.64	0.48	0.85
Micronesia (Federated States of)	Both	50-54 years	2.27	1.74	2.92	Micronesia (Federated States of)	Male	50-54 years	1.97	1.50	2.53	Micronesia (Federated States of)	Female	45-49 years	1.43	1.12	1.79
Micronesia (Federated States of)	Both	55-59 years	3.49	2.72	4.25	Micronesia (Federated States of)	Male	55-59 years	3.02	2.36	3.71	Micronesia (Federated States of)	Female	50-54 years	2.57	1.96	3.29

Micronesia (Federated States of)	Both	60-64 years	4.89	3.78	6.03	Micronesia (Federated States of)	Male	60-64 years	4.21	3.27	5.21	Micronesia (Federated States of)	Female	55-59 years	3.95	3.08	4.81
Micronesia (Federated States of)	Both	65-69 years	7.00	5.60	8.51	Micronesia (Federated States of)	Male	65-69 years	5.88	4.68	7.22	Micronesia (Federated States of)	Female	60-64 years	5.57	4.27	6.91
Micronesia (Federated States of)	Both	70-74 years	9.70	7.59	12.09	Micronesia (Federated States of)	Male	70-74 years	7.90	6.14	9.71	Micronesia (Federated States of)	Female	65-69 years	8.03	6.39	9.75
Micronesia (Federated States of)	Both	75-79 years	12.40	9.96	15.11	Micronesia (Federated States of)	Male	75-79 years	9.82	7.85	11.85	Micronesia (Federated States of)	Female	70-74 years	11.12	8.65	14.00
Micronesia (Federated States of)	Both	All ages	0.97	0.83	1.12	Micronesia (Federated States of)	Male	All ages	0.77	0.65	0.89	Micronesia (Federated States of)	Female	75-79 years	14.04	11.24	17.28
Micronesia (Federated States of)	Both	80-84	14.75	12.11	17.78	Micronesia (Federated States of)	Male	80-84	11.51	9.39	13.98	Micronesia (Federated States of)	Female	All ages	1.17	1.00	1.36
Micronesia (Federated States of)	Both	85-89	16.18	13.44	19.36	Micronesia (Federated States of)	Male	85-89	12.76	10.52	15.30	Micronesia (Federated States of)	Female	80-84	16.55	13.53	20.03
Micronesia (Federated States of)	Both	90-94	16.97	14.10	20.01	Micronesia (Federated States of)	Male	90-94	13.66	11.31	16.48	Micronesia (Federated States of)	Female	85-89	18.17	15.06	21.70
Monaco	Both	40-44 years	1.06	0.80	1.36	Monaco	Male	40-44 years	0.56	0.41	0.74	Monaco	Female	40-44 years	1.51	1.14	1.95
Monaco	Both	45-49 years	1.82	1.44	2.25	Monaco	Male	45-49 years	1.16	0.90	1.48	Monaco	Female	45-49 years	2.42	1.89	3.01
Monaco	Both	50-54 years	2.70	2.10	3.41	Monaco	Male	50-54 years	2.06	1.58	2.65	Monaco	Female	50-54 years	3.32	2.58	4.15
Monaco	Both	55-59 years	3.95	3.15	4.76	Monaco	Male	55-59 years	3.47	2.74	4.21	Monaco	Female	55-59 years	4.47	3.55	5.43
Monaco	Both	60-64 years	5.79	4.61	7.05	Monaco	Male	60-64 years	5.39	4.24	6.69	Monaco	Female	60-64 years	6.20	4.95	7.57
Monaco	Both	65-69 years	8.98	7.32	10.83	Monaco	Male	65-69 years	8.20	6.58	10.00	Monaco	Female	65-69 years	9.70	7.88	11.67

Monaco	Both	70-74 years	13.18	10.47	16.18	Monaco	Male	70-74 years	11.68	9.12	14.56	Monaco	Female	70-74 years	14.51	11.52	18.04
Monaco	Both	75-79 years	17.44	14.07	21.07	Monaco	Male	75-79 years	15.08	12.13	18.30	Monaco	Female	75-79 years	19.33	15.50	23.40
Monaco	Both	All ages	5.37	4.63	6.12	Monaco	Male	All ages	4.37	3.74	5.04	Monaco	Female	All ages	6.27	5.41	7.17
Monaco	Both	80-84	21.33	17.61	25.42	Monaco	Male	80-84	18.23	15.11	21.68	Monaco	Female	80-84	23.87	19.51	28.80
Monaco	Both	85-89	24.89	20.97	29.40	Monaco	Male	85-89	20.80	17.54	24.48	Monaco	Female	85-89	27.56	23.06	32.90
Monaco	Both	90-94	27.55	23.55	31.83	Monaco	Male	90-94	22.72	19.40	26.33	Monaco	Female	90-94	30.31	25.72	35.33
Mongolia	Both	40-44 years	0.42	0.31	0.56	Mongolia	Male	40-44 years	0.37	0.27	0.50	Mongolia	Female	40-44 years	0.46	0.34	0.63
Mongolia	Both	45-49 years	0.88	0.67	1.11	Mongolia	Male	45-49 years	0.76	0.58	0.96	Mongolia	Female	45-49 years	0.99	0.76	1.27
Mongolia	Both	50-54 years	1.55	1.18	2.01	Mongolia	Male	50-54 years	1.31	1.01	1.68	Mongolia	Female	50-54 years	1.77	1.33	2.30
Mongolia	Both	55-59 years	2.44	1.91	2.99	Mongolia	Male	55-59 years	2.05	1.65	2.53	Mongolia	Female	55-59 years	2.78	2.13	3.42
Mongolia	Both	60-64 years	3.57	2.77	4.43	Mongolia	Male	60-64 years	2.97	2.37	3.69	Mongolia	Female	60-64 years	4.04	3.10	5.02
Mongolia	Both	65-69 years	5.38	4.34	6.59	Mongolia	Male	65-69 years	4.39	3.58	5.36	Mongolia	Female	65-69 years	6.08	4.89	7.42
Mongolia	Both	70-74 years	7.73	6.02	9.75	Mongolia	Male	70-74 years	6.23	4.88	7.76	Mongolia	Female	70-74 years	8.73	6.75	11.08
Mongolia	Both	75-79 years	10.07	7.99	12.25	Mongolia	Male	75-79 years	8.15	6.58	9.87	Mongolia	Female	75-79 years	11.40	9.01	13.90
Mongolia	Both	All ages	0.75	0.65	0.86	Mongolia	Male	All ages	0.56	0.47	0.64	Mongolia	Female	All ages	0.94	0.81	1.08
Mongolia	Both	80-84	12.41	10.05	15.15	Mongolia	Male	80-84	10.05	8.14	12.32	Mongolia	Female	80-84	13.89	11.08	16.99
Mongolia	Both	85-89	14.56	11.95	17.60	Mongolia	Male	85-89	11.74	9.55	14.22	Mongolia	Female	85-89	15.83	12.87	19.34
Mongolia	Both	90-94	16.57	13.73	19.84	Mongolia	Male	90-94	13.29	10.92	16.05	Mongolia	Female	90-94	17.25	14.22	20.74
Montenegro	Both	All ages	2.55	2.20	2.93	Montenegro	Male	40-44 years	0.50	0.36	0.67	Montenegro	Female	40-44 years	0.58	0.42	0.79
Montenegro	Both	80-84	16.27	13.26	19.82	Montenegro	Male	45-49 years	1.03	0.80	1.29	Montenegro	Female	45-49 years	1.26	0.96	1.61
Montenegro	Both	85-89	18.37	15.18	22.11	Montenegro	Male	50-54 years	1.81	1.37	2.32	Montenegro	Female	50-54 years	2.26	1.70	2.99
Montenegro	Both	90-94	19.87	16.60	23.65	Montenegro	Male	55-59 years	2.87	2.24	3.56	Montenegro	Female	55-59 years	3.59	2.75	4.46
Montenegro	Both	40-44 years	0.54	0.39	0.73	Montenegro	Male	60-64 years	4.21	3.28	5.33	Montenegro	Female	60-64 years	5.31	4.12	6.61
Montenegro	Both	45-49 years	1.15	0.88	1.45	Montenegro	Male	65-69 years	6.28	4.99	7.81	Montenegro	Female	65-69 years	8.02	6.45	9.78

Montenegro	Both	50-54 years	2.04	1.53	2.65	Montenegro	Male	70-74 years	8.92	6.94	11.17	Montenegro	Female	70-74 years	11.44	8.81	14.33
Montenegro	Both	55-59 years	3.24	2.51	4.00	Montenegro	Male	75-79 years	11.61	9.27	14.18	Montenegro	Female	75-79 years	14.72	11.74	18.12
Montenegro	Both	60-64 years	4.77	3.72	5.99	Montenegro	Male	All ages	2.02	1.71	2.34	Montenegro	Female	All ages	3.07	2.63	3.55
Montenegro	Both	65-69 years	7.21	5.82	8.85	Montenegro	Male	80-84	14.15	11.38	17.28	Montenegro	Female	80-84	17.61	14.28	21.54
Montenegro	Both	70-74 years	10.36	8.04	12.87	Montenegro	Male	85-89	16.17	13.21	19.68	Montenegro	Female	85-89	19.66	16.29	23.74
Montenegro	Both	75-79 years	13.46	10.76	16.45	Montenegro	Male	90-94	17.74	14.66	21.16	Montenegro	Female	90-94	20.95	17.50	25.02
Morocco	Both	40-44 years	0.39	0.29	0.52	Morocco	Male	40-44 years	0.35	0.25	0.48	Morocco	Female	40-44 years	0.43	0.32	0.59
Morocco	Both	45-49 years	0.83	0.64	1.04	Morocco	Male	45-49 years	0.73	0.56	0.92	Morocco	Female	45-49 years	0.92	0.71	1.17
Morocco	Both	50-54 years	1.46	1.12	1.86	Morocco	Male	50-54 years	1.27	0.97	1.63	Morocco	Female	50-54 years	1.64	1.25	2.11
Morocco	Both	55-59 years	2.30	1.81	2.81	Morocco	Male	55-59 years	2.00	1.58	2.45	Morocco	Female	55-59 years	2.59	2.02	3.19
Morocco	Both	60-64 years	3.34	2.64	4.13	Morocco	Male	60-64 years	2.90	2.28	3.62	Morocco	Female	60-64 years	3.80	2.94	4.70
Morocco	Both	65-69 years	4.97	4.00	6.08	Morocco	Male	65-69 years	4.30	3.42	5.29	Morocco	Female	65-69 years	5.69	4.61	6.95
Morocco	Both	70-74 years	7.11	5.49	9.00	Morocco	Male	70-74 years	6.09	4.64	7.65	Morocco	Female	70-74 years	8.12	6.28	10.39
Morocco	Both	75-79 years	9.37	7.53	11.41	Morocco	Male	75-79 years	7.97	6.32	9.71	Morocco	Female	75-79 years	10.57	8.47	12.94
Morocco	Both	All ages	0.92	0.79	1.05	Morocco	Male	All ages	0.79	0.68	0.91	Morocco	Female	All ages	1.04	0.90	1.20
Morocco	Both	80-84	11.48	9.37	14.03	Morocco	Male	80-84	9.80	7.94	12.15	Morocco	Female	80-84	12.86	10.47	15.79
Morocco	Both	85-89	13.13	10.82	15.83	Morocco	Male	85-89	11.42	9.33	13.84	Morocco	Female	85-89	14.63	12.03	17.64
Morocco	Both	90-94	14.31	11.91	17.06	Morocco	Male	90-94	12.84	10.59	15.45	Morocco	Female	90-94	15.92	13.17	19.13
Mozambique	Both	80-84	11.76	9.53	14.52	Mozambique	Male	40-44 years	0.29	0.21	0.39	Mozambique	Female	80-84	12.88	10.39	15.92
Mozambique	Both	85-89	13.67	11.19	16.49	Mozambique	Male	45-49 years	0.64	0.49	0.81	Mozambique	Female	85-89	14.69	12.00	17.74
Mozambique	Both	90-94	15.11	12.55	18.00	Mozambique	Male	50-54 years	1.16	0.88	1.48	Mozambique	Female	90-94	15.96	13.17	18.98
Mozambique	Both	40-44 years	0.33	0.24	0.44	Mozambique	Male	55-59 years	1.85	1.45	2.28	Mozambique	Female	40-44 years	0.37	0.26	0.50
Mozambique	Both	45-49 years	0.73	0.55	0.93	Mozambique	Male	60-64 years	2.73	2.12	3.40	Mozambique	Female	45-49 years	0.81	0.61	1.04
Mozambique	Both	50-54 years	1.34	1.00	1.70	Mozambique	Male	65-69 years	4.07	3.22	5.03	Mozambique	Female	50-54 years	1.49	1.10	1.93

Mozambique	Both	55-59 years	2.15	1.67	2.64	Mozambique	Male	70-74 years	5.79	4.42	7.38	Mozambique	Female	55-59 years	2.42	1.88	3.01
Mozambique	Both	60-64 years	3.19	2.48	3.98	Mozambique	Male	75-79 years	7.64	6.13	9.39	Mozambique	Female	60-64 years	3.61	2.81	4.56
Mozambique	Both	65-69 years	4.83	3.85	5.93	Mozambique	Male	All ages	0.27	0.23	0.31	Mozambique	Female	65-69 years	5.49	4.33	6.75
Mozambique	Both	70-74 years	7.02	5.36	8.86	Mozambique	Male	80-84	9.46	7.62	11.72	Mozambique	Female	70-74 years	7.93	6.03	10.09
Mozambique	Both	75-79 years	9.38	7.54	11.50	Mozambique	Male	85-89	11.01	9.00	13.29	Mozambique	Female	75-79 years	10.47	8.34	12.82
Mozambique	Both	All ages	0.35	0.30	0.41	Mozambique	Male	90-94	12.32	10.14	14.81	Mozambique	Female	All ages	0.43	0.37	0.50
Myanmar	Both	40-44 years	0.64	0.47	0.85	Myanmar	Male	80-84	12.33	10.03	15.12	Myanmar	Female	40-44 years	0.71	0.53	0.95
Myanmar	Both	45-49 years	1.41	1.09	1.77	Myanmar	Male	85-89	13.70	11.17	16.63	Myanmar	Female	45-49 years	1.60	1.23	2.00
Myanmar	Both	50-54 years	2.54	1.93	3.30	Myanmar	Male	90-94	14.66	12.08	17.56	Myanmar	Female	50-54 years	2.91	2.18	3.79
Myanmar	Both	55-59 years	3.92	3.09	4.83	Myanmar	Male	40-44 years	0.55	0.40	0.74	Myanmar	Female	55-59 years	4.49	3.48	5.59
Myanmar	Both	60-64 years	5.51	4.28	6.84	Myanmar	Male	45-49 years	1.19	0.93	1.51	Myanmar	Female	60-64 years	6.33	4.85	7.87
Myanmar	Both	65-69 years	7.89	6.45	9.66	Myanmar	Male	50-54 years	2.11	1.62	2.73	Myanmar	Female	65-69 years	9.16	7.36	11.28
Myanmar	Both	70-74 years	10.90	8.70	13.68	Myanmar	Male	55-59 years	3.23	2.55	3.95	Myanmar	Female	70-74 years	12.72	10.08	16.21
Myanmar	Both	75-79 years	13.79	11.04	16.90	Myanmar	Male	60-64 years	4.49	3.52	5.53	Myanmar	Female	75-79 years	16.04	12.87	19.72
Myanmar	Both	All ages	1.39	1.19	1.59	Myanmar	Male	65-69 years	6.25	5.08	7.62	Myanmar	Female	All ages	1.75	1.50	2.00
Myanmar	Both	80-84	16.33	13.39	19.74	Myanmar	Male	70-74 years	8.40	6.61	10.53	Myanmar	Female	80-84	18.86	15.38	22.76
Myanmar	Both	85-89	18.14	14.92	21.69	Myanmar	Male	75-79 years	10.48	8.34	12.80	Myanmar	Female	85-89	20.64	16.95	24.81
Myanmar	Both	90-94	19.34	15.98	22.94	Myanmar	Male	All ages	1.00	0.86	1.15	Myanmar	Female	90-94	21.53	17.78	25.76
Namibia	Both	40-44 years	0.37	0.27	0.50	Namibia	Male	80-84	10.31	8.27	12.67	Namibia	Female	80-84	15.37	12.50	18.90
Namibia	Both	45-49 years	0.82	0.63	1.03	Namibia	Male	85-89	12.13	9.92	14.59	Namibia	Female	85-89	17.63	14.50	21.36
Namibia	Both	50-54 years	1.51	1.15	1.93	Namibia	Male	90-94	13.75	11.22	16.56	Namibia	Female	90-94	19.28	16.05	22.91
Namibia	Both	55-59 years	2.48	1.93	3.04	Namibia	Male	40-44 years	0.31	0.22	0.41	Namibia	Female	40-44 years	0.42	0.30	0.57
Namibia	Both	60-64 years	3.72	2.92	4.66	Namibia	Male	45-49 years	0.68	0.52	0.86	Namibia	Female	45-49 years	0.94	0.72	1.20
Namibia	Both	65-69 years	5.64	4.57	6.87	Namibia	Male	50-54 years	1.24	0.94	1.59	Namibia	Female	50-54 years	1.75	1.33	2.24

Namibia	Both	70-74 years	8.13	6.28	10.23	Namibia	Male	55-59 years	2.01	1.57	2.45	Namibia	Female	55-59 years	2.86	2.22	3.53
Namibia	Both	75-79 years	10.83	8.74	13.20	Namibia	Male	60-64 years	2.97	2.32	3.70	Namibia	Female	60-64 years	4.30	3.34	5.41
Namibia	Both	All ages	0.69	0.59	0.79	Namibia	Male	65-69 years	4.41	3.54	5.39	Namibia	Female	65-69 years	6.54	5.24	8.03
Namibia	Both	80-84	13.58	11.06	16.67	Namibia	Male	70-74 years	6.26	4.83	7.80	Namibia	Female	70-74 years	9.44	7.28	11.97
Namibia	Both	85-89	15.95	13.18	19.27	Namibia	Male	75-79 years	8.26	6.60	10.01	Namibia	Female	75-79 years	12.46	10.04	15.26
Namibia	Both	90-94	17.89	14.81	21.23	Namibia	Male	All ages	0.47	0.40	0.54	Namibia	Female	All ages	0.89	0.77	1.02
Nauru	Both	40-44 years	0.61	0.45	0.80	Nauru	Male	40-44 years	0.54	0.40	0.72	Nauru	Female	40-44 years	0.67	0.49	0.89
Nauru	Both	45-49 years	1.33	1.03	1.66	Nauru	Male	45-49 years	1.17	0.91	1.47	Nauru	Female	45-49 years	1.48	1.14	1.85
Nauru	Both	50-54 years	2.37	1.81	3.03	Nauru	Male	50-54 years	2.08	1.57	2.67	Nauru	Female	50-54 years	2.66	2.01	3.42
Nauru	Both	55-59 years	3.68	2.87	4.44	Nauru	Male	55-59 years	3.18	2.50	3.89	Nauru	Female	55-59 years	4.13	3.14	5.05
Nauru	Both	60-64 years	5.23	4.14	6.45	Nauru	Male	60-64 years	4.42	3.51	5.44	Nauru	Female	60-64 years	5.86	4.55	7.27
Nauru	Both	65-69 years	7.62	6.16	9.30	Nauru	Male	65-69 years	6.16	4.98	7.48	Nauru	Female	65-69 years	8.46	6.82	10.33
Nauru	Both	70-74 years	10.50	8.23	12.98	Nauru	Male	70-74 years	8.30	6.52	10.32	Nauru	Female	70-74 years	11.73	9.18	14.48
Nauru	Both	75-79 years	13.19	10.61	16.11	Nauru	Male	75-79 years	10.39	8.26	12.65	Nauru	Female	75-79 years	14.91	11.97	18.23
Nauru	Both	All ages	0.55	0.46	0.64	Nauru	Male	All ages	0.43	0.36	0.50	Nauru	Female	All ages	0.68	0.56	0.79
Nauru	Both	80-84	13.87	11.26	16.82	Nauru	Male	80-84	12.29	9.88	14.98	Nauru	Female	80-84	17.73	14.44	21.52
Nauru	Both	85-89	15.60	12.94	18.63	Nauru	Male	85-89	13.74	11.33	16.60	Nauru	Female	85-89	19.64	16.11	23.50
Nauru	Both	90-94	18.14	15.03	21.70	Nauru	Male	90-94	14.84	12.29	17.92	Nauru	Female	90-94	20.78	17.15	24.81
Nepal	Both	40-44 years	0.33	0.24	0.45	Nepal	Male	40-44 years	0.29	0.21	0.39	Nepal	Female	40-44 years	0.37	0.27	0.49
Nepal	Both	45-49 years	0.70	0.55	0.88	Nepal	Male	45-49 years	0.61	0.47	0.78	Nepal	Female	45-49 years	0.78	0.60	0.98
Nepal	Both	50-54 years	1.23	0.93	1.56	Nepal	Male	50-54 years	1.06	0.81	1.36	Nepal	Female	50-54 years	1.38	1.04	1.76
Nepal	Both	55-59 years	1.92	1.48	2.33	Nepal	Male	55-59 years	1.66	1.30	2.03	Nepal	Female	55-59 years	2.17	1.66	2.65
Nepal	Both	60-64 years	2.79	2.18	3.47	Nepal	Male	60-64 years	2.40	1.89	3.00	Nepal	Female	60-64 years	3.16	2.41	3.92
Nepal	Both	65-69 years	4.14	3.32	5.10	Nepal	Male	65-69 years	3.53	2.81	4.38	Nepal	Female	65-69 years	4.69	3.78	5.78

Nepal	Both	70-74 years	5.85	4.48	7.29	Nepal	Male	70-74 years	4.99	3.79	6.33	Nepal	Female	70-74 years	6.67	5.11	8.33
Nepal	Both	75-79 years	7.65	6.16	9.35	Nepal	Male	75-79 years	6.55	5.23	7.99	Nepal	Female	75-79 years	8.69	6.86	10.66
Nepal	Both	All ages	0.64	0.55	0.74	Nepal	Male	All ages	0.55	0.47	0.64	Nepal	Female	All ages	0.71	0.61	0.83
Nepal	Both	80-84	9.41	7.56	11.55	Nepal	Male	80-84	8.11	6.49	9.92	Nepal	Female	80-84	10.60	8.50	13.06
Nepal	Both	85-89	10.93	8.91	13.13	Nepal	Male	85-89	9.49	7.72	11.47	Nepal	Female	85-89	12.13	9.92	14.70
Nepal	Both	90-94	12.23	10.04	14.64	Nepal	Male	90-94	10.73	8.70	12.90	Nepal	Female	90-94	13.33	10.98	16.06
Netherlands	Both	All ages	4.05	3.50	4.63	Netherlands	Male	40-44 years	0.56	0.40	0.74	Netherlands	Female	All ages	4.75	4.10	5.39
Netherlands	Both	80-84	20.11	16.64	24.07	Netherlands	Male	45-49 years	1.16	0.90	1.45	Netherlands	Female	80-84	22.33	18.41	26.70
Netherlands	Both	85-89	23.05	19.24	27.53	Netherlands	Male	50-54 years	2.06	1.58	2.61	Netherlands	Female	85-89	25.33	21.10	30.15
Netherlands	Both	90-94	25.26	21.24	29.60	Netherlands	Male	55-59 years	3.44	2.70	4.22	Netherlands	Female	90-94	27.30	22.94	32.13
Netherlands	Both	40-44 years	1.02	0.76	1.33	Netherlands	Male	60-64 years	5.29	4.16	6.64	Netherlands	Female	40-44 years	1.46	1.09	1.92
Netherlands	Both	45-49 years	1.76	1.38	2.18	Netherlands	Male	65-69 years	8.02	6.49	10.01	Netherlands	Female	45-49 years	2.35	1.85	2.93
Netherlands	Both	50-54 years	2.65	2.06	3.31	Netherlands	Male	70-74 years	11.33	8.80	14.48	Netherlands	Female	50-54 years	3.23	2.51	4.06
Netherlands	Both	55-59 years	3.89	3.11	4.75	Netherlands	Male	75-79 years	14.42	11.54	18.02	Netherlands	Female	55-59 years	4.34	3.45	5.33
Netherlands	Both	60-64 years	5.66	4.51	6.97	Netherlands	Male	All ages	3.30	2.82	3.89	Netherlands	Female	60-64 years	6.01	4.83	7.36
Netherlands	Both	65-69 years	8.72	7.11	10.54	Netherlands	Male	80-84	17.13	14.03	20.73	Netherlands	Female	65-69 years	9.40	7.68	11.34
Netherlands	Both	70-74 years	12.70	9.93	15.99	Netherlands	Male	85-89	19.12	15.88	22.90	Netherlands	Female	70-74 years	14.00	10.94	17.51
Netherlands	Both	75-79 years	16.54	13.39	20.33	Netherlands	Male	90-94	20.37	16.95	24.20	Netherlands	Female	75-79 years	18.40	14.84	22.35
New Zealand	Both	55-59 years	2.56	2.02	3.10	New Zealand	Male	40-44 years	0.27	0.19	0.37	New Zealand	Female	55-59 years	3.19	2.51	3.86
New Zealand	Both	60-64 years	3.72	2.97	4.56	New Zealand	Male	45-49 years	0.60	0.46	0.77	New Zealand	Female	60-64 years	4.52	3.59	5.51
New Zealand	Both	65-69 years	5.58	4.52	6.76	New Zealand	Male	50-54 years	1.11	0.85	1.44	New Zealand	Female	65-69 years	6.84	5.53	8.26
New Zealand	Both	70-74 years	7.98	6.21	9.96	New Zealand	Male	55-59 years	1.87	1.47	2.29	New Zealand	Female	70-74 years	9.90	7.75	12.37
New Zealand	Both	75-79 years	10.50	8.46	12.66	New Zealand	Male	60-64 years	2.86	2.24	3.60	New Zealand	Female	75-79 years	13.12	10.51	15.92
New Zealand	Both	All ages	2.32	2.00	2.64	New Zealand	Male	65-69 years	4.25	3.40	5.25	New Zealand	Female	All ages	2.99	2.57	3.41

New Zealand	Both	80-84	13.02	10.59	15.79	New Zealand	Male	70-74 years	5.92	4.56	7.46	New Zealand	Female	80-84	16.25	13.27	19.61
New Zealand	Both	85-89	15.27	12.71	18.23	New Zealand	Male	75-79 years	7.56	5.97	9.30	New Zealand	Female	85-89	18.73	15.56	22.33
New Zealand	Both	90-94	17.28	14.37	20.52	New Zealand	Male	All ages	1.60	1.37	1.85	New Zealand	Female	90-94	20.54	17.10	24.46
New Zealand	Both	40-44 years	0.55	0.41	0.73	New Zealand	Male	80-84	9.08	7.31	11.24	New Zealand	Female	40-44 years	0.80	0.60	1.05
New Zealand	Both	45-49 years	1.05	0.82	1.32	New Zealand	Male	85-89	10.43	8.54	12.68	New Zealand	Female	45-49 years	1.44	1.12	1.79
New Zealand	Both	50-54 years	1.69	1.31	2.13	New Zealand	Male	90-94	11.63	9.53	13.90	New Zealand	Female	50-54 years	2.21	1.71	2.77
Nicaragua	Both	80-84	11.72	9.59	14.34	Nicaragua	Male	40-44 years	0.28	0.19	0.38	Nicaragua	Female	80-84	13.26	10.86	16.21
Nicaragua	Both	85-89	13.27	10.87	16.03	Nicaragua	Male	45-49 years	0.56	0.43	0.72	Nicaragua	Female	85-89	14.80	12.09	17.98
Nicaragua	Both	90-94	14.34	11.94	17.11	Nicaragua	Male	50-54 years	0.98	0.73	1.26	Nicaragua	Female	90-94	15.71	13.06	18.76
Nicaragua	Both	40-44 years	0.32	0.23	0.44	Nicaragua	Male	55-59 years	1.57	1.22	1.93	Nicaragua	Female	40-44 years	0.37	0.26	0.50
Nicaragua	Both	45-49 years	0.68	0.52	0.86	Nicaragua	Male	60-64 years	2.39	1.86	3.00	Nicaragua	Female	45-49 years	0.78	0.60	0.99
Nicaragua	Both	50-54 years	1.20	0.91	1.52	Nicaragua	Male	65-69 years	3.87	3.08	4.80	Nicaragua	Female	50-54 years	1.38	1.05	1.78
Nicaragua	Both	55-59 years	1.93	1.50	2.35	Nicaragua	Male	70-74 years	5.84	4.48	7.41	Nicaragua	Female	55-59 years	2.23	1.73	2.77
Nicaragua	Both	60-64 years	2.95	2.32	3.66	Nicaragua	Male	75-79 years	7.79	6.27	9.60	Nicaragua	Female	60-64 years	3.42	2.67	4.26
Nicaragua	Both	65-69 years	4.77	3.80	5.84	Nicaragua	Male	All ages	0.52	0.44	0.60	Nicaragua	Female	65-69 years	5.52	4.36	6.78
Nicaragua	Both	70-74 years	7.19	5.61	9.03	Nicaragua	Male	80-84	9.54	7.71	11.61	Nicaragua	Female	70-74 years	8.28	6.39	10.45
Nicaragua	Both	75-79 years	9.58	7.72	11.68	Nicaragua	Male	85-89	10.90	8.85	13.17	Nicaragua	Female	75-79 years	10.95	8.76	13.49
Nicaragua	Both	All ages	0.69	0.59	0.79	Nicaragua	Male	90-94	11.97	9.85	14.46	Nicaragua	Female	All ages	0.85	0.73	0.98
Niger	Both	70-74 years	5.64	4.32	7.05	Niger	Male	40-44 years	0.26	0.19	0.35	Niger	Female	70-74 years	6.27	4.77	7.81
Niger	Both	75-79 years	7.46	6.04	9.02	Niger	Male	45-49 years	0.57	0.44	0.72	Niger	Female	75-79 years	8.29	6.68	10.06
Niger	Both	All ages	0.26	0.22	0.30	Niger	Male	50-54 years	1.02	0.78	1.31	Niger	Female	All ages	0.29	0.25	0.33
Niger	Both	80-84	9.28	7.50	11.37	Niger	Male	55-59 years	1.64	1.29	2.01	Niger	Female	80-84	10.27	8.26	12.66
Niger	Both	85-89	10.81	8.83	12.96	Niger	Male	60-64 years	2.41	1.88	3.00	Niger	Female	85-89	11.89	9.63	14.38
Niger	Both	90-94	12.11	9.88	14.64	Niger	Male	65-69 years	3.54	2.82	4.35	Niger	Female	90-94	13.16	10.61	15.96

Niger	Both	40-44 years	0.28	0.20	0.38	Niger	Male	70-74 years	4.99	3.86	6.28	Niger	Female	40-44 years	0.30	0.22	0.41
Niger	Both	45-49 years	0.62	0.48	0.79	Niger	Male	75-79 years	6.58	5.32	7.98	Niger	Female	45-49 years	0.66	0.50	0.85
Niger	Both	50-54 years	1.12	0.84	1.43	Niger	Male	All ages	0.23	0.20	0.26	Niger	Female	50-54 years	1.21	0.90	1.57
Niger	Both	55-59 years	1.80	1.40	2.19	Niger	Male	80-84	8.20	6.62	10.03	Niger	Female	55-59 years	1.95	1.51	2.42
Niger	Both	60-64 years	2.65	2.07	3.31	Niger	Male	85-89	9.62	7.87	11.61	Niger	Female	60-64 years	2.90	2.26	3.64
Niger	Both	65-69 years	3.97	3.17	4.88	Niger	Male	90-94	10.87	8.86	13.23	Niger	Female	65-69 years	4.37	3.49	5.38
Nigeria	Both	40-44 years	0.26	0.19	0.35	Nigeria	Male	40-44 years	0.24	0.17	0.32	Nigeria	Female	40-44 years	0.28	0.20	0.37
Nigeria	Both	45-49 years	0.60	0.46	0.76	Nigeria	Male	45-49 years	0.55	0.42	0.70	Nigeria	Female	45-49 years	0.64	0.49	0.82
Nigeria	Both	50-54 years	1.13	0.86	1.45	Nigeria	Male	50-54 years	1.03	0.78	1.32	Nigeria	Female	50-54 years	1.21	0.92	1.55
Nigeria	Both	55-59 years	1.85	1.45	2.25	Nigeria	Male	55-59 years	1.67	1.31	2.04	Nigeria	Female	55-59 years	1.99	1.56	2.42
Nigeria	Both	60-64 years	2.77	2.18	3.41	Nigeria	Male	60-64 years	2.48	1.95	3.07	Nigeria	Female	60-64 years	3.00	2.35	3.70
Nigeria	Both	65-69 years	4.11	3.31	5.02	Nigeria	Male	65-69 years	3.65	2.95	4.47	Nigeria	Female	65-69 years	4.50	3.64	5.50
Nigeria	Both	70-74 years	5.77	4.46	7.23	Nigeria	Male	70-74 years	5.14	3.98	6.46	Nigeria	Female	70-74 years	6.43	4.97	8.02
Nigeria	Both	75-79 years	7.64	6.15	9.28	Nigeria	Male	75-79 years	6.77	5.45	8.27	Nigeria	Female	75-79 years	8.47	6.81	10.35
Nigeria	Both	All ages	0.33	0.28	0.37	Nigeria	Male	All ages	0.29	0.25	0.33	Nigeria	Female	All ages	0.36	0.31	0.41
Nigeria	Both	80-84	9.45	7.69	11.60	Nigeria	Male	80-84	8.44	6.86	10.36	Nigeria	Female	80-84	10.51	8.56	12.89
Nigeria	Both	85-89	11.13	9.18	13.36	Nigeria	Male	85-89	10.03	8.28	12.02	Nigeria	Female	85-89	12.32	10.14	14.84
Nigeria	Both	90-94	12.83	10.56	15.35	Nigeria	Male	90-94	11.57	9.54	13.86	Nigeria	Female	90-94	13.92	11.49	16.61
Niue	Both	80-84	15.59	12.72	18.73	Niue	Male	80-84	12.27	10.07	14.77	Niue	Female	40-44 years	0.66	0.49	0.88
Niue	Both	85-89	17.50	14.55	20.87	Niue	Male	85-89	13.74	11.45	16.54	Niue	Female	45-49 years	1.47	1.13	1.87
Niue	Both	90-94	18.88	15.74	22.42	Niue	Male	90-94	14.87	12.34	17.74	Niue	Female	50-54 years	2.67	2.05	3.45
Niue	Both	40-44 years	0.60	0.44	0.80	Niue	Male	40-44 years	0.54	0.40	0.73	Niue	Female	55-59 years	4.17	3.23	5.12
Niue	Both	45-49 years	1.32	1.02	1.67	Niue	Male	45-49 years	1.16	0.89	1.47	Niue	Female	60-64 years	5.94	4.61	7.37
Niue	Both	50-54 years	2.36	1.80	3.03	Niue	Male	50-54 years	2.06	1.57	2.65	Niue	Female	65-69 years	8.54	6.93	10.47

Niue	Both	55-59 years	3.64	2.85	4.48	Niue	Male	55-59 years	3.18	2.49	3.87	Niue	Female	70-74 years	11.77	9.04	14.67
Niue	Both	60-64 years	5.17	4.06	6.37	Niue	Male	60-64 years	4.46	3.49	5.50	Niue	Female	75-79 years	14.91	11.80	18.12
Niue	Both	65-69 years	7.47	6.07	9.10	Niue	Male	65-69 years	6.21	5.07	7.58	Niue	Female	All ages	2.70	2.32	3.09
Niue	Both	70-74 years	10.39	8.05	12.93	Niue	Male	70-74 years	8.32	6.58	10.41	Niue	Female	80-84	17.72	14.30	21.39
Niue	Both	75-79 years	13.20	10.55	15.97	Niue	Male	75-79 years	10.38	8.37	12.64	Niue	Female	85-89	19.66	16.22	23.65
Niue	Both	All ages	2.16	1.87	2.46	Niue	Male	All ages	1.61	1.39	1.84	Niue	Female	90-94	20.84	17.28	24.74
North Macedonia	Both	40-44 years	0.48	0.35	0.65	North Macedonia	Male	40-44 years	0.45	0.33	0.61	North Macedonia	Female	40-44 years	0.52	0.38	0.69
North Macedonia	Both	45-49 years	1.03	0.79	1.28	North Macedonia	Male	45-49 years	0.93	0.72	1.18	North Macedonia	Female	45-49 years	1.12	0.85	1.42
North Macedonia	Both	50-54 years	1.81	1.38	2.31	North Macedonia	Male	50-54 years	1.63	1.24	2.09	North Macedonia	Female	50-54 years	2.01	1.51	2.57
North Macedonia	Both	55-59 years	2.87	2.27	3.47	North Macedonia	Male	55-59 years	2.56	2.02	3.15	North Macedonia	Female	55-59 years	3.19	2.48	3.92
North Macedonia	Both	60-64 years	4.23	3.34	5.23	North Macedonia	Male	60-64 years	3.75	2.95	4.64	North Macedonia	Female	60-64 years	4.71	3.69	5.84
North Macedonia	Both	65-69 years	6.35	5.13	7.75	North Macedonia	Male	65-69 years	5.58	4.47	6.81	North Macedonia	Female	65-69 years	7.08	5.70	8.63
North Macedonia	Both	70-74 years	9.07	7.06	11.37	North Macedonia	Male	70-74 years	7.90	6.14	9.87	North Macedonia	Female	70-74 years	10.06	7.82	12.74
North Macedonia	Both	75-79 years	11.82	9.44	14.56	North Macedonia	Male	75-79 years	10.31	8.18	12.60	North Macedonia	Female	75-79 years	13.02	10.39	16.09
North Macedonia	Both	All ages	2.07	1.77	2.37	North Macedonia	Male	All ages	1.71	1.46	1.96	North Macedonia	Female	All ages	2.43	2.07	2.79
North Macedonia	Both	80-84	14.43	11.74	17.71	North Macedonia	Male	80-84	12.62	10.16	15.54	North Macedonia	Female	80-84	15.72	12.64	19.12
North Macedonia	Both	85-89	16.43	13.49	19.90	North Macedonia	Male	85-89	14.53	11.85	17.64	North Macedonia	Female	85-89	17.68	14.53	21.52
North Macedonia	Both	90-94	17.84	14.85	21.35	North Macedonia	Male	90-94	16.11	13.18	19.42	North Macedonia	Female	90-94	18.96	15.67	22.71
Northern Mariana Islands	Both	40-44 years	0.55	0.41	0.73	Northern Mariana Islands	Male	40-44 years	0.49	0.36	0.66	Northern Mariana Islands	Female	40-44 years	0.59	0.44	0.79

Northern Mariana Islands	Both	45-49 years	1.13	0.88	1.40	Northern Mariana Islands	Male	45-49 years	1.04	0.81	1.30	Northern Mariana Islands	Female	45-49 years	1.30	1.01	1.64
Northern Mariana Islands	Both	50-54 years	2.14	1.64	2.74	Northern Mariana Islands	Male	50-54 years	1.83	1.40	2.33	Northern Mariana Islands	Female	50-54 years	2.36	1.79	3.04
Northern Mariana Islands	Both	55-59 years	3.18	2.51	3.84	Northern Mariana Islands	Male	55-59 years	2.84	2.25	3.46	Northern Mariana Islands	Female	55-59 years	3.70	2.92	4.53
Northern Mariana Islands	Both	60-64 years	4.72	3.74	5.82	Northern Mariana Islands	Male	60-64 years	4.02	3.18	4.97	Northern Mariana Islands	Female	60-64 years	5.33	4.19	6.59
Northern Mariana Islands	Both	65-69 years	6.53	5.30	7.94	Northern Mariana Islands	Male	65-69 years	5.60	4.51	6.91	Northern Mariana Islands	Female	65-69 years	7.66	6.17	9.28
Northern Mariana Islands	Both	70-74 years	9.10	7.15	11.19	Northern Mariana Islands	Male	70-74 years	7.50	5.92	9.31	Northern Mariana Islands	Female	70-74 years	10.56	8.31	12.97
Northern Mariana Islands	Both	75-79 years	11.32	9.15	13.70	Northern Mariana Islands	Male	75-79 years	9.41	7.56	11.48	Northern Mariana Islands	Female	75-79 years	13.47	10.86	16.31
Northern Mariana Islands	Both	All ages	1.72	1.47	2.00	Northern Mariana Islands	Male	All ages	1.48	1.25	1.72	Northern Mariana Islands	Female	All ages	1.98	1.68	2.28
Northern Mariana Islands	Both	80-84	14.18	11.58	17.15	Northern Mariana Islands	Male	80-84	11.24	9.19	13.65	Northern Mariana Islands	Female	80-84	16.21	13.18	19.58
Northern Mariana Islands	Both	85-89	15.97	13.31	19.26	Northern Mariana Islands	Male	85-89	12.80	10.58	15.45	Northern Mariana Islands	Female	85-89	18.31	15.24	22.15
Northern Mariana Islands	Both	90-94	17.40	14.45	20.57	Northern Mariana Islands	Male	90-94	14.17	11.71	16.84	Northern Mariana Islands	Female	90-94	19.86	16.51	23.54
Norway	Both	40-44 years	0.96	0.72	1.25	Norway	Male	40-44 years	0.48	0.35	0.63	Norway	Female	40-44 years	1.46	1.10	1.87
Norway	Both	45-49 years	1.76	1.39	2.17	Norway	Male	45-49 years	1.06	0.82	1.33	Norway	Female	45-49 years	2.49	1.98	3.06
Norway	Both	50-54 years	2.79	2.16	3.48	Norway	Male	50-54 years	1.97	1.51	2.51	Norway	Female	50-54 years	3.64	2.84	4.58
Norway	Both	55-59 years	4.18	3.32	5.03	Norway	Male	55-59 years	3.34	2.64	4.02	Norway	Female	55-59 years	5.05	4.01	6.10
Norway	Both	60-64 years	6.10	4.89	7.44	Norway	Male	60-64 years	5.17	4.10	6.38	Norway	Female	60-64 years	7.04	5.67	8.57
Norway	Both	65-69 years	9.32	7.62	11.25	Norway	Male	65-69 years	7.78	6.30	9.44	Norway	Female	65-69 years	10.85	8.90	13.01
Norway	Both	70-74 years	13.47	10.58	16.53	Norway	Male	70-74 years	10.91	8.49	13.54	Norway	Female	70-74 years	15.93	12.52	19.72
Norway	Both	75-79 years	17.62	14.25	21.35	Norway	Male	75-79 years	13.91	11.26	16.92	Norway	Female	75-79 years	20.89	16.95	25.24
Norway	Both	All ages	3.87	3.36	4.40	Norway	Male	All ages	2.82	2.42	3.24	Norway	Female	All ages	4.90	4.25	5.55

Norway	Both	80-84	21.57	17.94	25.77	Norway	Male	80-84	16.61	13.68	20.05	Norway	Female	80-84	25.31	20.97	30.07
Norway	Both	85-89	24.69	20.57	29.30	Norway	Male	85-89	18.60	15.50	22.18	Norway	Female	85-89	28.47	23.86	33.66
Norway	Both	90-94	27.08	22.95	31.58	Norway	Male	90-94	19.96	16.71	23.57	Norway	Female	90-94	30.42	25.86	35.43
Oman	Both	40-44 years	0.40	0.29	0.53	Oman	Male	40-44 years	0.37	0.26	0.50	Oman	Female	40-44 years	0.46	0.33	0.62
Oman	Both	45-49 years	0.84	0.66	1.06	Oman	Male	45-49 years	0.77	0.60	0.98	Oman	Female	45-49 years	0.99	0.76	1.24
Oman	Both	50-54 years	1.50	1.15	1.93	Oman	Male	50-54 years	1.37	1.03	1.77	Oman	Female	50-54 years	1.78	1.34	2.28
Oman	Both	55-59 years	2.40	1.87	2.92	Oman	Male	55-59 years	2.16	1.68	2.67	Oman	Female	55-59 years	2.81	2.21	3.47
Oman	Both	60-64 years	3.57	2.79	4.44	Oman	Male	60-64 years	3.15	2.45	3.95	Oman	Female	60-64 years	4.14	3.20	5.16
Oman	Both	65-69 years	5.45	4.41	6.70	Oman	Male	65-69 years	4.66	3.73	5.72	Oman	Female	65-69 years	6.20	5.00	7.56
Oman	Both	70-74 years	7.68	5.97	9.58	Oman	Male	70-74 years	6.64	5.14	8.30	Oman	Female	70-74 years	8.86	6.83	11.11
Oman	Both	75-79 years	10.09	8.04	12.34	Oman	Male	75-79 years	8.77	7.06	10.68	Oman	Female	75-79 years	11.61	9.25	14.20
Oman	Both	All ages	0.41	0.35	0.47	Oman	Male	All ages	0.33	0.28	0.39	Oman	Female	All ages	0.54	0.46	0.62
Oman	Both	80-84	12.60	10.24	15.23	Oman	Male	80-84	10.96	8.82	13.34	Oman	Female	80-84	14.29	11.61	17.24
Oman	Both	85-89	15.15	12.67	18.04	Oman	Male	85-89	12.96	10.68	15.62	Oman	Female	85-89	16.51	13.72	19.67
Oman	Both	90-94	16.83	14.04	19.91	Oman	Male	90-94	14.77	12.22	17.53	Oman	Female	90-94	18.27	15.20	21.63
Pakistan	Both	40-44 years	0.34	0.25	0.45	Pakistan	Male	40-44 years	0.27	0.19	0.36	Pakistan	Female	40-44 years	0.42	0.30	0.55
Pakistan	Both	45-49 years	0.77	0.59	0.97	Pakistan	Male	45-49 years	0.58	0.45	0.74	Pakistan	Female	45-49 years	0.95	0.73	1.20
Pakistan	Both	50-54 years	1.39	1.05	1.77	Pakistan	Male	50-54 years	1.05	0.79	1.34	Pakistan	Female	50-54 years	1.76	1.33	2.27
Pakistan	Both	55-59 years	2.19	1.71	2.68	Pakistan	Male	55-59 years	1.66	1.30	2.04	Pakistan	Female	55-59 years	2.81	2.19	3.45
Pakistan	Both	60-64 years	3.21	2.53	3.98	Pakistan	Male	60-64 years	2.41	1.91	2.99	Pakistan	Female	60-64 years	4.10	3.21	5.09
Pakistan	Both	65-69 years	4.78	3.87	5.84	Pakistan	Male	65-69 years	3.53	2.85	4.36	Pakistan	Female	65-69 years	6.10	4.95	7.51
Pakistan	Both	70-74 years	6.76	5.23	8.49	Pakistan	Male	70-74 years	4.97	3.85	6.23	Pakistan	Female	70-74 years	8.68	6.69	10.93
Pakistan	Both	75-79 years	8.89	7.11	10.80	Pakistan	Male	75-79 years	6.52	5.18	7.95	Pakistan	Female	75-79 years	11.32	8.99	13.81
Pakistan	Both	All ages	0.48	0.41	0.55	Pakistan	Male	All ages	0.36	0.31	0.42	Pakistan	Female	All ages	0.60	0.51	0.68

Pakistan	Both	80-84	10.96	8.96	13.42	Pakistan	Male	80-84	8.10	6.58	9.95	Pakistan	Female	80-84	13.79	11.24	16.86
Pakistan	Both	85-89	12.71	10.50	15.35	Pakistan	Male	85-89	9.57	7.81	11.46	Pakistan	Female	85-89	15.66	12.88	19.10
Pakistan	Both	90-94	14.19	11.80	16.84	Pakistan	Male	90-94	10.99	9.04	13.20	Pakistan	Female	90-94	17.02	14.14	20.20
Palau	Both	40-44 years	0.59	0.45	0.77	Palau	Male	40-44 years	0.54	0.40	0.72	Palau	Female	40-44 years	0.66	0.50	0.87
Palau	Both	45-49 years	1.30	1.01	1.64	Palau	Male	45-49 years	1.16	0.89	1.47	Palau	Female	45-49 years	1.47	1.15	1.86
Palau	Both	50-54 years	2.34	1.78	3.00	Palau	Male	50-54 years	2.06	1.54	2.67	Palau	Female	50-54 years	2.66	2.05	3.45
Palau	Both	55-59 years	3.64	2.88	4.42	Palau	Male	55-59 years	3.17	2.50	3.85	Palau	Female	55-59 years	4.14	3.25	5.13
Palau	Both	60-64 years	5.15	4.06	6.33	Palau	Male	60-64 years	4.44	3.50	5.43	Palau	Female	60-64 years	5.88	4.60	7.31
Palau	Both	65-69 years	7.36	5.97	8.92	Palau	Male	65-69 years	6.18	4.99	7.43	Palau	Female	65-69 years	8.48	6.80	10.42
Palau	Both	70-74 years	10.08	7.83	12.52	Palau	Male	70-74 years	8.30	6.49	10.28	Palau	Female	70-74 years	11.72	9.02	14.54
Palau	Both	75-79 years	12.93	10.34	15.77	Palau	Male	75-79 years	10.40	8.32	12.81	Palau	Female	75-79 years	14.89	11.91	18.17
Palau	Both	All ages	1.81	1.54	2.08	Palau	Male	All ages	1.41	1.20	1.63	Palau	Female	All ages	2.27	1.94	2.62
Palau	Both	80-84	15.49	12.73	18.83	Palau	Male	80-84	12.33	9.97	15.18	Palau	Female	80-84	17.73	14.53	21.54
Palau	Both	85-89	17.11	14.12	20.56	Palau	Male	85-89	13.84	11.30	16.78	Palau	Female	85-89	19.75	16.32	23.82
Palau	Both	90-94	18.35	15.21	21.74	Palau	Male	90-94	15.02	12.31	17.93	Palau	Female	90-94	21.03	17.55	24.88
Palestine	Both	40-44 years	0.39	0.28	0.52	Palestine	Male	All ages	0.42	0.35	0.48	Palestine	Female	40-44 years	0.43	0.31	0.58
Palestine	Both	45-49 years	0.82	0.64	1.03	Palestine	Male	80-84	9.69	7.78	11.88	Palestine	Female	45-49 years	0.92	0.71	1.18
Palestine	Both	50-54 years	1.45	1.11	1.87	Palestine	Male	85-89	11.24	9.17	13.65	Palestine	Female	50-54 years	1.65	1.26	2.13
Palestine	Both	55-59 years	2.29	1.79	2.80	Palestine	Male	90-94	12.60	10.30	15.14	Palestine	Female	55-59 years	2.61	2.02	3.23
Palestine	Both	60-64 years	3.36	2.62	4.15	Palestine	Male	40-44 years	0.34	0.25	0.46	Palestine	Female	60-64 years	3.82	2.98	4.73
Palestine	Both	65-69 years	4.98	3.99	6.08	Palestine	Male	45-49 years	0.72	0.55	0.92	Palestine	Female	65-69 years	5.70	4.56	6.99
Palestine	Both	70-74 years	7.10	5.48	8.98	Palestine	Male	50-54 years	1.27	0.96	1.61	Palestine	Female	70-74 years	8.07	6.26	10.18
Palestine	Both	75-79 years	9.35	7.51	11.34	Palestine	Male	55-59 years	2.00	1.55	2.47	Palestine	Female	75-79 years	10.45	8.42	12.78
Palestine	Both	All ages	0.50	0.43	0.58	Palestine	Male	60-64 years	2.90	2.25	3.63	Palestine	Female	All ages	0.59	0.51	0.68

Palestine	Both	80-84	11.48	9.33	14.04	Palestine	Male	65-69 years	4.27	3.37	5.22	Palestine	Female	80-84	12.64	10.26	15.34
Palestine	Both	85-89	13.17	10.73	15.70	Palestine	Male	70-74 years	6.04	4.67	7.61	Palestine	Female	85-89	14.31	11.70	17.10
Palestine	Both	90-94	14.71	12.11	17.49	Palestine	Male	75-79 years	7.88	6.28	9.56	Palestine	Female	90-94	15.52	12.88	18.50
Panama	Both	45-49 years	0.63	0.49	0.79	Panama	Male	45-49 years	0.53	0.41	0.67	Panama	Female	40-44 years	0.35	0.25	0.48
Panama	Both	50-54 years	1.11	0.84	1.42	Panama	Male	50-54 years	0.92	0.70	1.18	Panama	Female	45-49 years	0.73	0.56	0.93
Panama	Both	55-59 years	1.80	1.42	2.21	Panama	Male	55-59 years	1.50	1.18	1.83	Panama	Female	50-54 years	1.29	0.98	1.66
Panama	Both	60-64 years	2.79	2.21	3.45	Panama	Male	60-64 years	2.31	1.82	2.87	Panama	Female	55-59 years	2.11	1.63	2.60
Panama	Both	65-69 years	4.47	3.59	5.45	Panama	Male	65-69 years	3.69	2.95	4.52	Panama	Female	60-64 years	3.26	2.57	4.06
Panama	Both	70-74 years	6.68	5.16	8.42	Panama	Male	70-74 years	5.53	4.28	6.97	Panama	Female	65-69 years	5.23	4.21	6.42
Panama	Both	75-79 years	8.95	7.15	11.01	Panama	Male	75-79 years	7.38	5.91	9.05	Panama	Female	70-74 years	7.81	6.02	9.85
Panama	Both	All ages	1.02	0.88	1.17	Panama	Male	All ages	0.82	0.70	0.95	Panama	Female	75-79 years	10.39	8.19	12.83
Panama	Both	80-84	11.07	8.98	13.55	Panama	Male	80-84	9.13	7.30	11.30	Panama	Female	All ages	1.22	1.05	1.40
Panama	Both	85-89	12.79	10.44	15.40	Panama	Male	85-89	10.61	8.65	12.83	Panama	Female	80-84	12.74	10.29	15.59
Panama	Both	90-94	14.16	11.66	16.85	Panama	Male	90-94	11.91	9.82	14.23	Panama	Female	85-89	14.50	11.78	17.68
Panama	Both	40-44 years	0.31	0.22	0.42	Panama	Male	40-44 years	0.26	0.19	0.36	Panama	Female	90-94	15.75	12.90	18.90
Papua New Guinea	Both	40-44 years	0.55	0.40	0.72	Papua New Guinea	Male	40-44 years	0.49	0.36	0.66	Papua New Guinea	Female	40-44 years	0.60	0.45	0.80
Papua New Guinea	Both	45-49 years	1.18	0.91	1.48	Papua New Guinea	Male	45-49 years	1.05	0.80	1.33	Papua New Guinea	Female	45-49 years	1.32	1.03	1.65
Papua New Guinea	Both	50-54 years	2.09	1.60	2.67	Papua New Guinea	Male	50-54 years	1.84	1.39	2.35	Papua New Guinea	Female	50-54 years	2.36	1.79	3.00
Papua New Guinea	Both	55-59 years	3.20	2.50	3.88	Papua New Guinea	Male	55-59 years	2.81	2.21	3.44	Papua New Guinea	Female	55-59 years	3.65	2.82	4.44
Papua New Guinea	Both	60-64 years	4.47	3.51	5.46	Papua New Guinea	Male	60-64 years	3.91	3.12	4.81	Papua New Guinea	Female	60-64 years	5.15	3.97	6.36
Papua New Guinea	Both	65-69 years	6.30	5.11	7.62	Papua New Guinea	Male	65-69 years	5.44	4.42	6.57	Papua New Guinea	Female	65-69 years	7.41	5.95	9.12
Papua New Guinea	Both	70-74 years	8.68	6.84	10.81	Papua New Guinea	Male	70-74 years	7.28	5.74	8.98	Papua New Guinea	Female	70-74 years	10.24	8.06	12.99

Papua New Guinea	Both	75-79 years	10.95	8.85	13.29	Papua New Guinea	Male	75-79 years	9.07	7.28	10.96	Papua New Guinea	Female	75-79 years	12.98	10.51	15.87
Papua New Guinea	Both	All ages	0.60	0.51	0.69	Papua New Guinea	Male	All ages	0.53	0.45	0.61	Papua New Guinea	Female	All ages	0.67	0.57	0.77
Papua New Guinea	Both	80-84	13.06	10.69	15.77	Papua New Guinea	Male	80-84	10.68	8.68	13.03	Papua New Guinea	Female	80-84	15.38	12.65	18.54
Papua New Guinea	Both	85-89	14.67	12.11	17.59	Papua New Guinea	Male	85-89	11.90	9.72	14.50	Papua New Guinea	Female	85-89	16.98	14.01	20.34
Papua New Guinea	Both	90-94	15.95	13.27	19.01	Papua New Guinea	Male	90-94	12.83	10.52	15.43	Papua New Guinea	Female	90-94	17.90	14.83	21.41
Paraguay	Both	40-44 years	0.34	0.25	0.46	Paraguay	Male	40-44 years	0.30	0.22	0.41	Paraguay	Female	40-44 years	0.38	0.27	0.52
Paraguay	Both	45-49 years	0.73	0.55	0.92	Paraguay	Male	45-49 years	0.64	0.49	0.81	Paraguay	Female	45-49 years	0.81	0.62	1.05
Paraguay	Both	50-54 years	1.29	0.97	1.66	Paraguay	Male	50-54 years	1.13	0.85	1.43	Paraguay	Female	50-54 years	1.46	1.10	1.90
Paraguay	Both	55-59 years	2.09	1.62	2.55	Paraguay	Male	55-59 years	1.81	1.41	2.23	Paraguay	Female	55-59 years	2.36	1.82	2.91
Paraguay	Both	60-64 years	3.16	2.47	3.94	Paraguay	Male	60-64 years	2.73	2.13	3.40	Paraguay	Female	60-64 years	3.58	2.78	4.47
Paraguay	Both	65-69 years	4.94	3.91	6.09	Paraguay	Male	65-69 years	4.28	3.41	5.29	Paraguay	Female	65-69 years	5.57	4.41	6.85
Paraguay	Both	70-74 years	7.29	5.60	9.29	Paraguay	Male	70-74 years	6.34	4.85	8.10	Paraguay	Female	70-74 years	8.15	6.20	10.37
Paraguay	Both	75-79 years	9.70	7.71	11.97	Paraguay	Male	75-79 years	8.41	6.73	10.41	Paraguay	Female	75-79 years	10.75	8.48	13.24
Paraguay	Both	All ages	0.87	0.75	1.00	Paraguay	Male	All ages	0.71	0.61	0.83	Paraguay	Female	All ages	1.02	0.88	1.18
Paraguay	Both	80-84	11.93	9.75	14.62	Paraguay	Male	80-84	10.34	8.42	12.75	Paraguay	Female	80-84	13.14	10.73	16.11
Paraguay	Both	85-89	13.70	11.21	16.57	Paraguay	Male	85-89	11.92	9.72	14.60	Paraguay	Female	85-89	14.88	12.12	18.12
Paraguay	Both	90-94	15.03	12.35	18.09	Paraguay	Male	90-94	13.20	10.70	15.94	Paraguay	Female	90-94	16.06	13.17	19.43
Peru	Both	40-44 years	0.27	0.19	0.36	Peru	Male	40-44 years	0.24	0.17	0.32	Peru	Female	40-44 years	0.30	0.21	0.41
Peru	Both	45-49 years	0.56	0.43	0.70	Peru	Male	45-49 years	0.48	0.37	0.61	Peru	Female	45-49 years	0.63	0.48	0.80
Peru	Both	50-54 years	0.98	0.74	1.24	Peru	Male	50-54 years	0.84	0.64	1.08	Peru	Female	50-54 years	1.11	0.84	1.43
Peru	Both	55-59 years	1.58	1.24	1.93	Peru	Male	55-59 years	1.34	1.05	1.64	Peru	Female	55-59 years	1.80	1.40	2.23
Peru	Both	60-64 years	2.39	1.88	2.95	Peru	Male	60-64 years	2.02	1.59	2.49	Peru	Female	60-64 years	2.75	2.13	3.41

Peru	Both	65-69 years	3.70	2.96	4.53	Peru	Male	65-69 years	3.12	2.49	3.82	Peru	Female	65-69 years	4.25	3.40	5.28
Peru	Both	70-74 years	5.39	4.14	6.82	Peru	Male	70-74 years	4.55	3.51	5.71	Peru	Female	70-74 years	6.19	4.72	7.81
Peru	Both	75-79 years	7.14	5.71	8.74	Peru	Male	75-79 years	6.04	4.83	7.49	Peru	Female	75-79 years	8.17	6.54	9.96
Peru	Both	All ages	0.79	0.68	0.90	Peru	Male	All ages	0.65	0.56	0.74	Peru	Female	All ages	0.93	0.80	1.06
Peru	Both	80-84	8.86	7.12	10.90	Peru	Male	80-84	7.51	5.99	9.28	Peru	Female	80-84	10.07	8.05	12.44
Peru	Both	85-89	10.38	8.47	12.61	Peru	Male	85-89	8.87	7.22	10.80	Peru	Female	85-89	11.66	9.49	14.26
Peru	Both	90-94	11.73	9.66	14.11	Peru	Male	90-94	10.18	8.32	12.41	Peru	Female	90-94	12.98	10.63	15.82
Philippines	Both	45-49 years	1.19	0.92	1.49	Philippines	Male	40-44 years	0.41	0.31	0.54	Philippines	Female	45-49 years	1.47	1.13	1.85
Philippines	Both	50-54 years	2.21	1.69	2.83	Philippines	Male	45-49 years	0.92	0.71	1.15	Philippines	Female	50-54 years	2.76	2.10	3.55
Philippines	Both	55-59 years	3.50	2.76	4.25	Philippines	Male	50-54 years	1.66	1.27	2.12	Philippines	Female	55-59 years	4.41	3.46	5.36
Philippines	Both	60-64 years	5.04	3.98	6.16	Philippines	Male	55-59 years	2.58	2.04	3.12	Philippines	Female	60-64 years	6.34	5.00	7.76
Philippines	Both	65-69 years	7.18	5.84	8.67	Philippines	Male	60-64 years	3.64	2.88	4.46	Philippines	Female	65-69 years	9.04	7.31	10.97
Philippines	Both	70-74 years	9.89	7.75	12.22	Philippines	Male	65-69 years	5.04	4.12	6.14	Philippines	Female	70-74 years	12.28	9.63	15.18
Philippines	Both	75-79 years	12.62	10.19	15.29	Philippines	Male	70-74 years	6.71	5.28	8.29	Philippines	Female	75-79 years	15.37	12.43	18.64
Philippines	Both	All ages	1.03	0.89	1.17	Philippines	Male	75-79 years	8.37	6.76	10.12	Philippines	Female	All ages	1.40	1.21	1.59
Philippines	Both	80-84	15.12	12.46	18.29	Philippines	Male	All ages	0.66	0.57	0.76	Philippines	Female	80-84	18.07	14.91	21.81
Philippines	Both	85-89	16.83	13.93	20.05	Philippines	Male	80-84	9.92	8.15	12.09	Philippines	Female	85-89	19.93	16.53	23.74
Philippines	Both	90-94	17.23	14.39	20.43	Philippines	Male	85-89	11.24	9.29	13.41	Philippines	Female	90-94	21.08	17.72	24.92
Philippines	Both	40-44 years	0.51	0.38	0.67	Philippines	Male	90-94	12.42	10.30	14.79	Philippines	Female	40-44 years	0.62	0.46	0.80
Poland	Both	40-44 years	0.41	0.30	0.55	Poland	Male	40-44 years	0.33	0.24	0.44	Poland	Female	40-44 years	0.50	0.37	0.66
Poland	Both	45-49 years	0.92	0.71	1.16	Poland	Male	45-49 years	0.71	0.55	0.89	Poland	Female	45-49 years	1.13	0.87	1.43
Poland	Both	50-54 years	1.69	1.28	2.17	Poland	Male	50-54 years	1.27	0.97	1.61	Poland	Female	50-54 years	2.11	1.59	2.71
Poland	Both	55-59 years	2.75	2.16	3.35	Poland	Male	55-59 years	2.03	1.60	2.47	Poland	Female	55-59 years	3.43	2.67	4.20
Poland	Both	60-64 years	4.11	3.25	5.05	Poland	Male	60-64 years	2.97	2.35	3.67	Poland	Female	60-64 years	5.12	4.04	6.30

Poland	Both	65-69 years	6.18	5.02	7.54	Poland	Male	65-69 years	4.36	3.55	5.33	Poland	Female	65-69 years	7.65	6.21	9.33
Poland	Both	70-74 years	8.81	6.83	10.95	Poland	Male	70-74 years	6.12	4.78	7.65	Poland	Female	70-74 years	10.78	8.35	13.43
Poland	Both	75-79 years	11.61	9.27	14.09	Poland	Male	75-79 years	8.00	6.42	9.71	Poland	Female	75-79 years	13.84	11.04	16.88
Poland	Both	All ages	2.56	2.21	2.94	Poland	Male	All ages	1.55	1.34	1.78	Poland	Female	All ages	3.49	3.00	4.00
Poland	Both	80-84	14.36	11.79	17.46	Poland	Male	80-84	9.90	8.06	12.11	Poland	Female	80-84	16.62	13.66	20.16
Poland	Both	85-89	16.62	13.76	19.85	Poland	Male	85-89	11.68	9.65	14.01	Poland	Female	85-89	18.74	15.55	22.34
Poland	Both	90-94	18.56	15.50	21.98	Poland	Male	90-94	13.41	11.06	15.99	Poland	Female	90-94	20.26	16.98	23.98
Portugal	Both	40-44 years	1.03	0.77	1.34	Portugal	Male	40-44 years	0.55	0.40	0.74	Portugal	Female	40-44 years	1.47	1.08	1.92
Portugal	Both	45-49 years	1.79	1.41	2.22	Portugal	Male	45-49 years	1.14	0.87	1.43	Portugal	Female	45-49 years	2.38	1.83	3.00
Portugal	Both	50-54 years	2.68	2.09	3.37	Portugal	Male	50-54 years	2.03	1.54	2.62	Portugal	Female	50-54 years	3.27	2.52	4.12
Portugal	Both	55-59 years	3.92	3.14	4.75	Portugal	Male	55-59 years	3.38	2.64	4.18	Portugal	Female	55-59 years	4.40	3.46	5.36
Portugal	Both	60-64 years	5.67	4.55	6.98	Portugal	Male	60-64 years	5.19	4.04	6.50	Portugal	Female	60-64 years	6.10	4.86	7.42
Portugal	Both	65-69 years	8.80	7.17	10.63	Portugal	Male	65-69 years	7.90	6.40	9.66	Portugal	Female	65-69 years	9.57	7.80	11.57
Portugal	Both	70-74 years	12.90	10.08	16.16	Portugal	Male	70-74 years	11.22	8.62	14.05	Portugal	Female	70-74 years	14.26	11.12	18.12
Portugal	Both	75-79 years	16.77	13.46	20.35	Portugal	Male	75-79 years	14.25	11.29	17.57	Portugal	Female	75-79 years	18.67	15.10	22.73
Portugal	Both	All ages	4.60	3.94	5.26	Portugal	Male	All ages	3.52	2.99	4.05	Portugal	Female	All ages	5.54	4.76	6.36
Portugal	Both	80-84	20.25	16.53	24.29	Portugal	Male	80-84	16.82	13.58	20.44	Portugal	Female	80-84	22.53	18.36	27.06
Portugal	Both	85-89	22.99	19.04	27.19	Portugal	Male	85-89	18.65	15.38	22.28	Portugal	Female	85-89	25.36	21.02	30.09
Portugal	Both	90-94	24.92	20.98	29.23	Portugal	Male	90-94	19.76	16.43	23.36	Portugal	Female	90-94	27.11	22.81	31.97
Puerto Rico	Both	40-44 years	0.31	0.22	0.42	Puerto Rico	Male	80-84	8.91	7.18	11.04	Puerto Rico	Female	80-84	12.39	10.01	15.27
Puerto Rico	Both	45-49 years	0.65	0.50	0.82	Puerto Rico	Male	85-89	10.55	8.62	12.92	Puerto Rico	Female	85-89	14.30	11.71	17.38
Puerto Rico	Both	50-54 years	1.15	0.89	1.47	Puerto Rico	Male	90-94	12.07	9.81	14.66	Puerto Rico	Female	90-94	15.83	13.11	18.82
Puerto Rico	Both	55-59 years	1.88	1.48	2.31	Puerto Rico	Male	40-44 years	0.27	0.19	0.37	Puerto Rico	Female	40-44 years	0.35	0.25	0.47
Puerto Rico	Both	60-64 years	2.87	2.28	3.55	Puerto Rico	Male	45-49 years	0.55	0.43	0.69	Puerto Rico	Female	45-49 years	0.74	0.57	0.93

Puerto Rico	Both	65-69 years	4.46	3.59	5.42	Puerto Rico	Male	50-54 years	0.96	0.73	1.24	Puerto Rico	Female	50-54 years	1.33	1.02	1.69
Puerto Rico	Both	70-74 years	6.56	5.10	8.19	Puerto Rico	Male	55-59 years	1.54	1.23	1.92	Puerto Rico	Female	55-59 years	2.17	1.71	2.66
Puerto Rico	Both	75-79 years	8.75	6.98	10.71	Puerto Rico	Male	60-64 years	2.34	1.85	2.90	Puerto Rico	Female	60-64 years	3.32	2.62	4.14
Puerto Rico	Both	All ages	2.11	1.80	2.43	Puerto Rico	Male	65-69 years	3.61	2.88	4.41	Puerto Rico	Female	65-69 years	5.17	4.14	6.32
Puerto Rico	Both	80-84	10.91	8.87	13.42	Puerto Rico	Male	70-74 years	5.30	4.13	6.65	Puerto Rico	Female	70-74 years	7.57	5.79	9.47
Puerto Rico	Both	85-89	12.83	10.56	15.63	Puerto Rico	Male	75-79 years	7.10	5.72	8.68	Puerto Rico	Female	75-79 years	10.04	8.00	12.37
Puerto Rico	Both	90-94	14.49	11.98	17.28	Puerto Rico	Male	All ages	1.58	1.36	1.84	Puerto Rico	Female	All ages	2.57	2.19	2.97
Qatar	Both	40-44 years	0.37	0.27	0.51	Qatar	Male	40-44 years	0.35	0.25	0.48	Qatar	Female	40-44 years	0.44	0.32	0.60
Qatar	Both	45-49 years	0.78	0.60	0.99	Qatar	Male	45-49 years	0.73	0.56	0.93	Qatar	Female	45-49 years	0.94	0.72	1.18
Qatar	Both	50-54 years	1.38	1.04	1.76	Qatar	Male	50-54 years	1.30	0.98	1.66	Qatar	Female	50-54 years	1.68	1.26	2.13
Qatar	Both	55-59 years	2.19	1.73	2.66	Qatar	Male	55-59 years	2.07	1.63	2.54	Qatar	Female	55-59 years	2.69	2.08	3.31
Qatar	Both	60-64 years	3.26	2.57	4.01	Qatar	Male	60-64 years	3.06	2.41	3.77	Qatar	Female	60-64 years	3.99	3.07	5.02
Qatar	Both	65-69 years	4.90	3.96	5.98	Qatar	Male	65-69 years	4.54	3.65	5.53	Qatar	Female	65-69 years	5.98	4.80	7.35
Qatar	Both	70-74 years	7.02	5.40	8.72	Qatar	Male	70-74 years	6.46	4.97	8.07	Qatar	Female	70-74 years	8.54	6.57	10.59
Qatar	Both	75-79 years	9.49	7.63	11.60	Qatar	Male	75-79 years	8.59	6.91	10.55	Qatar	Female	75-79 years	11.28	9.01	13.81
Qatar	Both	All ages	0.36	0.30	0.42	Qatar	Male	All ages	0.35	0.29	0.41	Qatar	Female	All ages	0.40	0.33	0.46
Qatar	Both	80-84	11.67	9.42	14.15	Qatar	Male	80-84	10.84	8.70	13.31	Qatar	Female	80-84	14.08	11.49	17.02
Qatar	Both	85-89	13.50	11.20	16.36	Qatar	Male	85-89	12.99	10.76	15.76	Qatar	Female	85-89	16.54	13.77	19.73
Qatar	Both	90-94	15.59	12.90	18.54	Qatar	Male	90-94	15.05	12.36	17.95	Qatar	Female	90-94	18.65	15.68	21.96
Republic of Korea	Both	All ages	2.12	1.83	2.42	Republic of Korea	Male	All ages	1.30	1.11	1.49	Republic of Korea	Female	All ages	2.93	2.53	3.35
Republic of Korea	Both	80-84	13.43	11.02	16.29	Republic of Korea	Male	80-84	8.67	6.92	10.72	Republic of Korea	Female	80-84	16.17	13.17	19.57
Republic of Korea	Both	85-89	16.26	13.45	19.48	Republic of Korea	Male	85-89	10.14	8.24	12.30	Republic of Korea	Female	85-89	18.80	15.56	22.48
Republic of Korea	Both	90-94	18.60	15.42	21.92	Republic of Korea	Male	90-94	11.44	9.35	13.77	Republic of Korea	Female	90-94	20.74	17.23	24.52
Republic of Korea	Both	40-44 years	0.57	0.43	0.76	Republic of Korea	Male	40-44 years	0.29	0.21	0.38	Republic of Korea	Female	40-44 years	0.88	0.65	1.17

Republic of Korea	Both	45-49 years	1.03	0.79	1.28	Republic of Korea	Male	45-49 years	0.59	0.46	0.75	Republic of Korea	Female	45-49 years	1.48	1.13	1.87
Republic of Korea	Both	50-54 years	1.59	1.22	1.99	Republic of Korea	Male	50-54 years	1.05	0.81	1.33	Republic of Korea	Female	50-54 years	2.14	1.62	2.72
Republic of Korea	Both	55-59 years	2.37	1.87	2.91	Republic of Korea	Male	55-59 years	1.74	1.37	2.13	Republic of Korea	Female	55-59 years	3.01	2.36	3.74
Republic of Korea	Both	60-64 years	3.46	2.78	4.25	Republic of Korea	Male	60-64 years	2.63	2.06	3.28	Republic of Korea	Female	60-64 years	4.27	3.38	5.25
Republic of Korea	Both	65-69 years	5.29	4.29	6.44	Republic of Korea	Male	65-69 years	3.91	3.16	4.76	Republic of Korea	Female	65-69 years	6.60	5.33	8.04
Republic of Korea	Both	70-74 years	7.74	6.05	9.72	Republic of Korea	Male	70-74 years	5.49	4.24	6.91	Republic of Korea	Female	70-74 years	9.72	7.56	12.29
Republic of Korea	Both	75-79 years	10.48	8.44	12.74	Republic of Korea	Male	75-79 years	7.09	5.63	8.70	Republic of Korea	Female	75-79 years	12.98	10.43	16.02
Republic of Moldova	Both	55-59 years	2.89	2.27	3.53	Republic of Moldova	Male	40-44 years	0.39	0.28	0.52	Republic of Moldova	Female	40-44 years	0.58	0.42	0.77
Republic of Moldova	Both	60-64 years	4.26	3.33	5.28	Republic of Moldova	Male	45-49 years	0.79	0.62	1.01	Republic of Moldova	Female	45-49 years	1.25	0.95	1.59
Republic of Moldova	Both	65-69 years	6.48	5.21	7.93	Republic of Moldova	Male	50-54 years	1.37	1.04	1.75	Republic of Moldova	Female	50-54 years	2.22	1.67	2.87
Republic of Moldova	Both	70-74 years	9.36	7.25	11.90	Republic of Moldova	Male	55-59 years	2.14	1.69	2.63	Republic of Moldova	Female	55-59 years	3.50	2.70	4.28
Republic of Moldova	Both	75-79 years	12.23	9.70	15.01	Republic of Moldova	Male	60-64 years	3.11	2.48	3.82	Republic of Moldova	Female	60-64 years	5.12	3.94	6.40
Republic of Moldova	Both	All ages	2.27	1.97	2.62	Republic of Moldova	Male	65-69 years	4.58	3.68	5.57	Republic of Moldova	Female	65-69 years	7.79	6.19	9.60
Republic of Moldova	Both	80-84	14.81	12.16	18.07	Republic of Moldova	Male	70-74 years	6.46	5.03	8.07	Republic of Moldova	Female	70-74 years	11.20	8.63	14.51
Republic of Moldova	Both	85-89	16.71	13.65	20.19	Republic of Moldova	Male	75-79 years	8.41	6.74	10.22	Republic of Moldova	Female	75-79 years	14.44	11.33	18.07
Republic of Moldova	Both	90-94	18.09	14.87	21.46	Republic of Moldova	Male	All ages	1.36	1.18	1.56	Republic of Moldova	Female	All ages	3.08	2.65	3.57
Republic of Moldova	Both	40-44 years	0.48	0.36	0.64	Republic of Moldova	Male	80-84	10.29	8.38	12.56	Republic of Moldova	Female	80-84	17.22	14.04	20.89
Republic of Moldova	Both	45-49 years	1.02	0.79	1.30	Republic of Moldova	Male	85-89	11.91	9.76	14.44	Republic of Moldova	Female	85-89	19.02	15.68	22.97
Republic of Moldova	Both	50-54 years	1.83	1.39	2.32	Republic of Moldova	Male	90-94	13.31	10.91	16.10	Republic of Moldova	Female	90-94	19.95	16.39	23.68

Romania	Both	40-44 years	0.43	0.31	0.57	Romania	Male	40-44 years	0.40	0.29	0.53	Romania	Female	40-44 years	0.46	0.34	0.62
Romania	Both	45-49 years	0.90	0.69	1.13	Romania	Male	45-49 years	0.82	0.64	1.03	Romania	Female	45-49 years	0.98	0.75	1.24
Romania	Both	50-54 years	1.59	1.20	2.03	Romania	Male	50-54 years	1.42	1.09	1.81	Romania	Female	50-54 years	1.75	1.32	2.26
Romania	Both	55-59 years	2.53	1.98	3.07	Romania	Male	55-59 years	2.24	1.77	2.74	Romania	Female	55-59 years	2.80	2.16	3.42
Romania	Both	60-64 years	3.75	2.96	4.68	Romania	Male	60-64 years	3.28	2.61	4.14	Romania	Female	60-64 years	4.16	3.24	5.21
Romania	Both	65-69 years	5.63	4.55	6.90	Romania	Male	65-69 years	4.87	3.92	6.02	Romania	Female	65-69 years	6.24	5.00	7.76
Romania	Both	70-74 years	8.04	6.18	10.09	Romania	Male	70-74 years	6.90	5.34	8.69	Romania	Female	70-74 years	8.86	6.81	11.23
Romania	Both	75-79 years	10.54	8.45	12.98	Romania	Male	75-79 years	9.03	7.21	11.06	Romania	Female	75-79 years	11.49	9.19	14.18
Romania	Both	All ages	2.49	2.13	2.85	Romania	Male	All ages	1.87	1.61	2.14	Romania	Female	All ages	3.06	2.60	3.52
Romania	Both	80-84	12.95	10.39	15.95	Romania	Male	80-84	11.13	8.94	13.64	Romania	Female	80-84	13.94	11.13	17.16
Romania	Both	85-89	14.91	12.20	18.16	Romania	Male	85-89	12.99	10.57	15.79	Romania	Female	85-89	15.89	13.01	19.37
Romania	Both	90-94	16.47	13.67	19.93	Romania	Male	90-94	14.64	12.02	17.72	Romania	Female	90-94	17.37	14.38	20.97
Russian Federation	Both	40-44 years	0.50	0.37	0.65	Russian Federation	Male	40-44 years	0.28	0.21	0.38	Russian Federation	Female	40-44 years	0.70	0.52	0.90
Russian Federation	Both	45-49 years	1.13	0.87	1.43	Russian Federation	Male	45-49 years	0.60	0.46	0.75	Russian Federation	Female	45-49 years	1.62	1.25	2.04
Russian Federation	Both	50-54 years	2.11	1.60	2.71	Russian Federation	Male	50-54 years	1.05	0.81	1.32	Russian Federation	Female	50-54 years	3.03	2.29	3.93
Russian Federation	Both	55-59 years	3.45	2.69	4.20	Russian Federation	Male	55-59 years	1.66	1.31	2.01	Russian Federation	Female	55-59 years	4.89	3.80	5.98
Russian Federation	Both	60-64 years	5.21	4.07	6.40	Russian Federation	Male	60-64 years	2.42	1.91	2.97	Russian Federation	Female	60-64 years	7.23	5.62	8.88
Russian Federation	Both	65-69 years	8.03	6.53	9.73	Russian Federation	Male	65-69 years	3.52	2.86	4.30	Russian Federation	Female	65-69 years	10.93	8.88	13.24
Russian Federation	Both	70-74 years	11.68	9.08	14.53	Russian Federation	Male	70-74 years	4.93	3.82	6.11	Russian Federation	Female	70-74 years	15.53	12.10	19.35
Russian Federation	Both	75-79 years	15.81	12.68	19.14	Russian Federation	Male	75-79 years	6.45	5.19	7.81	Russian Federation	Female	75-79 years	19.81	15.93	24.01
Russian Federation	Both	All ages	2.93	2.54	3.35	Russian Federation	Male	All ages	1.02	0.88	1.17	Russian Federation	Female	All ages	4.54	3.92	5.18

Russian Federation	Both	80-84	19.32	15.93	23.35	Russian Federation	Male	80-84	8.03	6.56	9.87	Russian Federation	Female	80-84	23.39	19.23	28.25
Russian Federation	Both	85-89	21.77	18.08	25.92	Russian Federation	Male	85-89	9.61	7.91	11.50	Russian Federation	Female	85-89	25.73	21.31	30.73
Russian Federation	Both	90-94	23.43	19.82	27.50	Russian Federation	Male	90-94	11.30	9.32	13.61	Russian Federation	Female	90-94	27.01	22.74	31.65
Rwanda	Both	40-44 years	0.32	0.23	0.43	Rwanda	Male	40-44 years	0.28	0.20	0.37	Rwanda	Female	40-44 years	0.35	0.26	0.48
Rwanda	Both	45-49 years	0.70	0.54	0.90	Rwanda	Male	45-49 years	0.61	0.46	0.79	Rwanda	Female	45-49 years	0.78	0.60	0.99
Rwanda	Both	50-54 years	1.28	0.97	1.64	Rwanda	Male	50-54 years	1.11	0.84	1.46	Rwanda	Female	50-54 years	1.42	1.07	1.84
Rwanda	Both	55-59 years	2.07	1.61	2.54	Rwanda	Male	55-59 years	1.79	1.39	2.19	Rwanda	Female	55-59 years	2.31	1.79	2.83
Rwanda	Both	60-64 years	3.09	2.42	3.85	Rwanda	Male	60-64 years	2.63	2.06	3.35	Rwanda	Female	60-64 years	3.44	2.68	4.30
Rwanda	Both	65-69 years	4.67	3.70	5.79	Rwanda	Male	65-69 years	3.90	3.11	4.85	Rwanda	Female	65-69 years	5.24	4.17	6.49
Rwanda	Both	70-74 years	6.78	5.19	8.62	Rwanda	Male	70-74 years	5.53	4.27	6.98	Rwanda	Female	70-74 years	7.58	5.75	9.75
Rwanda	Both	75-79 years	9.00	7.18	11.06	Rwanda	Male	75-79 years	7.27	5.81	8.90	Rwanda	Female	75-79 years	10.01	7.93	12.50
Rwanda	Both	All ages	0.44	0.38	0.51	Rwanda	Male	All ages	0.32	0.27	0.37	Rwanda	Female	All ages	0.55	0.47	0.64
Rwanda	Both	80-84	11.16	9.02	13.68	Rwanda	Male	80-84	9.01	7.29	11.10	Rwanda	Female	80-84	12.32	9.94	15.18
Rwanda	Both	85-89	12.98	10.68	15.70	Rwanda	Male	85-89	10.57	8.59	12.91	Rwanda	Female	85-89	14.13	11.62	17.13
Rwanda	Both	90-94	14.50	11.90	17.27	Rwanda	Male	90-94	11.94	9.79	14.44	Rwanda	Female	90-94	15.47	12.64	18.50
Saint Kitts and Nevis	Both	55-59 years	1.88	1.48	2.29	Saint Kitts and Nevis	Male	40-44 years	0.27	0.19	0.37	Saint Kitts and Nevis	Female	55-59 years	2.19	1.71	2.68
Saint Kitts and Nevis	Both	60-64 years	2.83	2.23	3.52	Saint Kitts and Nevis	Male	45-49 years	0.57	0.44	0.72	Saint Kitts and Nevis	Female	60-64 years	3.29	2.58	4.14
Saint Kitts and Nevis	Both	65-69 years	4.40	3.55	5.41	Saint Kitts and Nevis	Male	50-54 years	0.99	0.75	1.28	Saint Kitts and Nevis	Female	65-69 years	5.13	4.06	6.28
Saint Kitts and Nevis	Both	70-74 years	6.50	5.04	8.20	Saint Kitts and Nevis	Male	55-59 years	1.58	1.24	1.93	Saint Kitts and Nevis	Female	70-74 years	7.57	5.79	9.57
Saint Kitts and Nevis	Both	75-79 years	8.71	6.92	10.70	Saint Kitts and Nevis	Male	60-64 years	2.35	1.84	2.95	Saint Kitts and Nevis	Female	75-79 years	10.08	7.93	12.42
Saint Kitts and Nevis	Both	All ages	1.03	0.89	1.18	Saint Kitts and Nevis	Male	65-69 years	3.66	2.92	4.52	Saint Kitts and Nevis	Female	All ages	1.25	1.07	1.43

Saint Kitts and Nevis	Both	80-84	11.06	8.91	13.55	Saint Kitts and Nevis	Male	70-74 years	5.40	4.17	6.88	Saint Kitts and Nevis	Female	80-84	12.49	9.96	15.43
Saint Kitts and Nevis	Both	85-89	13.26	10.89	16.07	Saint Kitts and Nevis	Male	75-79 years	7.23	5.82	8.87	Saint Kitts and Nevis	Female	85-89	14.41	11.78	17.58
Saint Kitts and Nevis	Both	90-94	15.23	12.55	18.33	Saint Kitts and Nevis	Male	All ages	0.82	0.70	0.95	Saint Kitts and Nevis	Female	90-94	15.90	13.08	19.09
Saint Kitts and Nevis	Both	40-44 years	0.32	0.23	0.42	Saint Kitts and Nevis	Male	80-84	9.04	7.24	11.07	Saint Kitts and Nevis	Female	40-44 years	0.36	0.26	0.49
Saint Kitts and Nevis	Both	45-49 years	0.66	0.51	0.85	Saint Kitts and Nevis	Male	85-89	10.67	8.76	12.92	Saint Kitts and Nevis	Female	45-49 years	0.77	0.59	0.98
Saint Kitts and Nevis	Both	50-54 years	1.17	0.89	1.52	Saint Kitts and Nevis	Male	90-94	12.17	9.94	14.62	Saint Kitts and Nevis	Female	50-54 years	1.36	1.03	1.78
Saint Lucia	Both	40-44 years	0.33	0.23	0.44	Saint Lucia	Male	40-44 years	0.28	0.20	0.38	Saint Lucia	Female	40-44 years	0.37	0.27	0.51
Saint Lucia	Both	45-49 years	0.69	0.53	0.87	Saint Lucia	Male	45-49 years	0.58	0.45	0.74	Saint Lucia	Female	45-49 years	0.79	0.60	1.00
Saint Lucia	Both	50-54 years	1.21	0.92	1.54	Saint Lucia	Male	50-54 years	1.01	0.77	1.31	Saint Lucia	Female	50-54 years	1.40	1.05	1.77
Saint Lucia	Both	55-59 years	1.95	1.52	2.36	Saint Lucia	Male	55-59 years	1.62	1.26	2.01	Saint Lucia	Female	55-59 years	2.26	1.77	2.77
Saint Lucia	Both	60-64 years	2.94	2.30	3.65	Saint Lucia	Male	60-64 years	2.43	1.91	3.06	Saint Lucia	Female	60-64 years	3.43	2.69	4.26
Saint Lucia	Both	65-69 years	4.60	3.65	5.62	Saint Lucia	Male	65-69 years	3.79	2.99	4.70	Saint Lucia	Female	65-69 years	5.36	4.23	6.55
Saint Lucia	Both	70-74 years	6.74	5.17	8.53	Saint Lucia	Male	70-74 years	5.58	4.25	7.10	Saint Lucia	Female	70-74 years	7.88	6.06	9.98
Saint Lucia	Both	75-79 years	9.06	7.13	11.10	Saint Lucia	Male	75-79 years	7.42	5.87	9.11	Saint Lucia	Female	75-79 years	10.41	8.18	12.73
Saint Lucia	Both	All ages	1.26	1.08	1.43	Saint Lucia	Male	All ages	0.97	0.84	1.12	Saint Lucia	Female	All ages	1.54	1.33	1.75
Saint Lucia	Both	80-84	11.25	9.11	13.76	Saint Lucia	Male	80-84	9.19	7.47	11.29	Saint Lucia	Female	80-84	12.74	10.23	15.64
Saint Lucia	Both	85-89	13.12	10.76	15.84	Saint Lucia	Male	85-89	10.71	8.83	12.94	Saint Lucia	Female	85-89	14.53	11.83	17.56
Saint Lucia	Both	90-94	14.62	12.15	17.49	Saint Lucia	Male	90-94	12.06	9.88	14.47	Saint Lucia	Female	90-94	15.84	13.12	18.91
Saint Vincent and the Grenadin es	Both	80-84	11.22	9.09	13.68	Saint Vincent and the Grenadin es	Male	40-44 years	0.28	0.20	0.39	Saint Vincent and the Grenadin es	Female	80-84	12.98	10.44	15.61
Saint Vincent and the Grenadin	Both	85-89	12.98	10.59	15.58	Saint Vincent and the Grenadin	Male	45-49 years	0.59	0.45	0.74	Saint Vincent and the Grenadin	Female	85-89	14.76	12.04	17.85

es						es						es					
Saint Vincent and the Grenadines	Both	90-94	14.62	12.10	17.57	Saint Vincent and the Grenadines	Male	50-54 years	1.03	0.78	1.30	Saint Vincent and the Grenadines	Female	90-94	16.03	13.17	19.36
Saint Vincent and the Grenadines	Both	40-44 years	0.33	0.24	0.45	Saint Vincent and the Grenadines	Male	55-59 years	1.65	1.29	2.04	Saint Vincent and the Grenadines	Female	40-44 years	0.38	0.27	0.51
Saint Vincent and the Grenadines	Both	45-49 years	0.69	0.54	0.87	Saint Vincent and the Grenadines	Male	60-64 years	2.48	1.93	3.13	Saint Vincent and the Grenadines	Female	45-49 years	0.80	0.62	1.02
Saint Vincent and the Grenadines	Both	50-54 years	1.22	0.93	1.55	Saint Vincent and the Grenadines	Male	65-69 years	3.86	3.05	4.76	Saint Vincent and the Grenadines	Female	50-54 years	1.43	1.09	1.82
Saint Vincent and the Grenadines	Both	55-59 years	1.96	1.53	2.40	Saint Vincent and the Grenadines	Male	70-74 years	5.68	4.31	7.23	Saint Vincent and the Grenadines	Female	55-59 years	2.30	1.79	2.84
Saint Vincent and the Grenadines	Both	60-64 years	2.95	2.31	3.68	Saint Vincent and the Grenadines	Male	75-79 years	7.55	6.07	9.34	Saint Vincent and the Grenadines	Female	60-64 years	3.48	2.73	4.35
Saint Vincent and the Grenadines	Both	65-69 years	4.62	3.69	5.64	Saint Vincent and the Grenadines	Male	All ages	1.02	0.87	1.18	Saint Vincent and the Grenadines	Female	65-69 years	5.45	4.33	6.64
Saint Vincent and the Grenadines	Both	70-74 years	6.78	5.19	8.61	Saint Vincent and the Grenadines	Male	80-84	9.35	7.52	11.69	Saint Vincent and the Grenadines	Female	70-74 years	8.02	6.09	10.19
Saint Vincent and the Grenadines	Both	75-79 years	9.06	7.21	11.22	Saint Vincent and the Grenadines	Male	85-89	10.90	8.86	13.26	Saint Vincent and the Grenadines	Female	75-79 years	10.60	8.32	13.09
Saint Vincent and the Grenadines	Both	All ages	1.22	1.05	1.39	Saint Vincent and the Grenadines	Male	90-94	12.25	10.06	14.86	Saint Vincent and the Grenadines	Female	All ages	1.42	1.22	1.63

Samoa	Both	40-44 years	0.69	0.52	0.90	Samoa	Male	40-44 years	0.61	0.46	0.82	Samoa	Female	40-44 years	0.76	0.56	1.00
Samoa	Both	45-49 years	1.51	1.17	1.88	Samoa	Male	45-49 years	1.33	1.04	1.67	Samoa	Female	45-49 years	1.71	1.31	2.16
Samoa	Both	50-54 years	2.70	2.04	3.46	Samoa	Male	50-54 years	2.37	1.81	3.03	Samoa	Female	50-54 years	3.08	2.31	4.00
Samoa	Both	55-59 years	4.16	3.24	5.07	Samoa	Male	55-59 years	3.63	2.84	4.42	Samoa	Female	55-59 years	4.74	3.67	5.87
Samoa	Both	60-64 years	5.86	4.52	7.23	Samoa	Male	60-64 years	5.07	3.90	6.33	Samoa	Female	60-64 years	6.68	5.12	8.22
Samoa	Both	65-69 years	8.43	6.83	10.25	Samoa	Male	65-69 years	7.10	5.69	8.69	Samoa	Female	65-69 years	9.69	7.80	11.73
Samoa	Both	70-74 years	11.64	9.10	14.63	Samoa	Male	70-74 years	9.56	7.49	12.05	Samoa	Female	70-74 years	13.47	10.53	16.92
Samoa	Both	75-79 years	14.73	11.86	18.01	Samoa	Male	75-79 years	11.88	9.39	14.59	Samoa	Female	75-79 years	17.00	13.70	20.93
Samoa	Both	All ages	1.23	1.06	1.41	Samoa	Male	All ages	1.00	0.86	1.15	Samoa	Female	All ages	1.47	1.28	1.69
Samoa	Both	80-84	17.33	14.31	20.91	Samoa	Male	80-84	13.87	11.15	16.97	Samoa	Female	80-84	19.97	16.37	24.18
Samoa	Both	85-89	19.02	15.71	22.76	Samoa	Male	85-89	15.26	12.47	18.45	Samoa	Female	85-89	21.78	17.98	26.00
Samoa	Both	90-94	19.94	16.67	23.59	Samoa	Male	90-94	16.16	13.31	19.15	Samoa	Female	90-94	22.60	18.94	26.88
San Marino	Both	40-44 years	1.10	0.83	1.44	San Marino	Male	40-44 years	0.56	0.41	0.75	San Marino	Female	40-44 years	1.52	1.14	1.99
San Marino	Both	45-49 years	1.88	1.46	2.33	San Marino	Male	45-49 years	1.17	0.90	1.48	San Marino	Female	45-49 years	2.46	1.90	3.08
San Marino	Both	50-54 years	2.77	2.13	3.47	San Marino	Male	50-54 years	2.09	1.61	2.69	San Marino	Female	50-54 years	3.38	2.56	4.26
San Marino	Both	55-59 years	4.04	3.19	4.91	San Marino	Male	55-59 years	3.51	2.75	4.32	San Marino	Female	55-59 years	4.55	3.58	5.58
San Marino	Both	60-64 years	5.87	4.66	7.23	San Marino	Male	60-64 years	5.42	4.21	6.79	San Marino	Female	60-64 years	6.33	5.06	7.78
San Marino	Both	65-69 years	9.04	7.33	10.87	San Marino	Male	65-69 years	8.22	6.59	10.02	San Marino	Female	65-69 years	9.91	7.99	11.96
San Marino	Both	70-74 years	13.17	10.27	16.45	San Marino	Male	70-74 years	11.59	9.00	14.43	San Marino	Female	70-74 years	14.76	11.46	18.50
San Marino	Both	75-79 years	17.22	13.83	21.10	San Marino	Male	75-79 years	14.78	11.79	17.89	San Marino	Female	75-79 years	19.36	15.63	23.48
San Marino	Both	All ages	4.13	3.57	4.68	San Marino	Male	All ages	3.41	2.91	3.92	San Marino	Female	All ages	4.76	4.13	5.41
San Marino	Both	80-84	20.91	17.34	24.94	San Marino	Male	80-84	17.59	14.40	21.18	San Marino	Female	80-84	23.42	19.32	28.05
San Marino	Both	85-89	23.78	19.91	28.48	San Marino	Male	85-89	19.64	16.19	23.38	San Marino	Female	85-89	26.47	22.26	31.79
San Marino	Both	90-94	25.87	21.82	30.37	San Marino	Male	90-94	20.91	17.47	24.73	San Marino	Female	90-94	28.45	24.04	33.41

Sao Tome and Principe	Both	40-44 years	0.31	0.22	0.41	Sao Tome and Principe	Male	40-44 years	0.28	0.20	0.38	Sao Tome and Principe	Female	40-44 years	0.33	0.24	0.45
Sao Tome and Principe	Both	45-49 years	0.67	0.52	0.85	Sao Tome and Principe	Male	45-49 years	0.62	0.48	0.79	Sao Tome and Principe	Female	45-49 years	0.72	0.55	0.93
Sao Tome and Principe	Both	50-54 years	1.23	0.92	1.56	Sao Tome and Principe	Male	50-54 years	1.13	0.85	1.44	Sao Tome and Principe	Female	50-54 years	1.32	0.99	1.70
Sao Tome and Principe	Both	55-59 years	1.99	1.55	2.45	Sao Tome and Principe	Male	55-59 years	1.82	1.41	2.24	Sao Tome and Principe	Female	55-59 years	2.16	1.67	2.67
Sao Tome and Principe	Both	60-64 years	2.96	2.33	3.67	Sao Tome and Principe	Male	60-64 years	2.68	2.08	3.36	Sao Tome and Principe	Female	60-64 years	3.23	2.53	4.05
Sao Tome and Principe	Both	65-69 years	4.43	3.52	5.50	Sao Tome and Principe	Male	65-69 years	3.96	3.14	4.92	Sao Tome and Principe	Female	65-69 years	4.88	3.90	6.05
Sao Tome and Principe	Both	70-74 years	6.34	4.86	8.01	Sao Tome and Principe	Male	70-74 years	5.58	4.29	7.02	Sao Tome and Principe	Female	70-74 years	7.02	5.36	8.95
Sao Tome and Principe	Both	75-79 years	8.40	6.70	10.40	Sao Tome and Principe	Male	75-79 years	7.34	5.87	9.04	Sao Tome and Principe	Female	75-79 years	9.26	7.33	11.53
Sao Tome and Principe	Both	All ages	0.46	0.40	0.53	Sao Tome and Principe	Male	All ages	0.40	0.34	0.46	Sao Tome and Principe	Female	All ages	0.53	0.45	0.61
Sao Tome and Principe	Both	80-84	10.40	8.43	12.87	Sao Tome and Principe	Male	80-84	9.12	7.42	11.30	Sao Tome and Principe	Female	80-84	11.45	9.25	14.19
Sao Tome and Principe	Both	85-89	12.14	10.03	14.66	Sao Tome and Principe	Male	85-89	10.71	8.80	13.05	Sao Tome and Principe	Female	85-89	13.24	10.86	16.06
Sao Tome and Principe	Both	90-94	13.58	11.26	16.18	Sao Tome and Principe	Male	90-94	12.13	9.98	14.69	Sao Tome and Principe	Female	90-94	14.67	12.11	17.49
Saudi Arabia	Both	80-84	10.48	8.50	12.76	Saudi Arabia	Male	80-84	9.25	7.47	11.39	Saudi Arabia	Female	40-44 years	0.40	0.28	0.54
Saudi Arabia	Both	85-89	12.64	10.42	15.32	Saudi Arabia	Male	85-89	11.08	9.07	13.46	Saudi Arabia	Female	45-49 years	0.84	0.64	1.05

Saudi Arabia	Both	90-94	14.51	11.99	17.26	Saudi Arabia	Male	90-94	12.86	10.50	15.41	Saudi Arabia	Female	50-54 years	1.49	1.12	1.87
Saudi Arabia	Both	40-44 years	0.35	0.25	0.47	Saudi Arabia	Male	40-44 years	0.32	0.23	0.43	Saudi Arabia	Female	55-59 years	2.37	1.87	2.90
Saudi Arabia	Both	45-49 years	0.73	0.57	0.92	Saudi Arabia	Male	45-49 years	0.66	0.51	0.85	Saudi Arabia	Female	60-64 years	3.51	2.78	4.34
Saudi Arabia	Both	50-54 years	1.29	0.98	1.64	Saudi Arabia	Male	50-54 years	1.16	0.88	1.48	Saudi Arabia	Female	65-69 years	5.23	4.25	6.41
Saudi Arabia	Both	55-59 years	2.06	1.62	2.50	Saudi Arabia	Male	55-59 years	1.84	1.43	2.26	Saudi Arabia	Female	70-74 years	7.45	5.83	9.31
Saudi Arabia	Both	60-64 years	3.02	2.39	3.74	Saudi Arabia	Male	60-64 years	2.69	2.13	3.36	Saudi Arabia	Female	75-79 years	9.78	7.88	11.87
Saudi Arabia	Both	65-69 years	4.45	3.62	5.46	Saudi Arabia	Male	65-69 years	3.96	3.21	4.91	Saudi Arabia	Female	All ages	0.49	0.42	0.56
Saudi Arabia	Both	70-74 years	6.27	4.90	7.77	Saudi Arabia	Male	70-74 years	5.58	4.36	6.99	Saudi Arabia	Female	80-84	12.11	9.81	14.72
Saudi Arabia	Both	75-79 years	8.28	6.71	10.05	Saudi Arabia	Male	75-79 years	7.37	5.97	8.96	Saudi Arabia	Female	85-89	14.17	11.71	17.13
Saudi Arabia	Both	All ages	0.45	0.38	0.51	Saudi Arabia	Male	All ages	0.42	0.35	0.48	Saudi Arabia	Female	90-94	15.97	13.24	19.21
Senegal	Both	80-84	10.29	8.35	12.63	Senegal	Male	40-44 years	0.28	0.20	0.39	Senegal	Female	80-84	11.34	9.14	13.81
Senegal	Both	85-89	12.01	9.78	14.48	Senegal	Male	45-49 years	0.62	0.47	0.79	Senegal	Female	85-89	13.10	10.65	15.75
Senegal	Both	90-94	13.50	11.05	16.18	Senegal	Male	50-54 years	1.12	0.84	1.43	Senegal	Female	90-94	14.49	11.90	17.36
Senegal	Both	40-44 years	0.30	0.22	0.41	Senegal	Male	55-59 years	1.79	1.38	2.19	Senegal	Female	40-44 years	0.32	0.24	0.44
Senegal	Both	45-49 years	0.67	0.51	0.85	Senegal	Male	60-64 years	2.63	2.05	3.29	Senegal	Female	45-49 years	0.72	0.55	0.91
Senegal	Both	50-54 years	1.22	0.92	1.56	Senegal	Male	65-69 years	3.91	3.12	4.85	Senegal	Female	50-54 years	1.32	0.99	1.69
Senegal	Both	55-59 years	1.97	1.53	2.41	Senegal	Male	70-74 years	5.55	4.31	6.99	Senegal	Female	55-59 years	2.15	1.67	2.62
Senegal	Both	60-64 years	2.92	2.28	3.62	Senegal	Male	75-79 years	7.31	5.86	9.01	Senegal	Female	60-64 years	3.21	2.51	4.03
Senegal	Both	65-69 years	4.38	3.51	5.41	Senegal	Male	All ages	0.38	0.33	0.44	Senegal	Female	65-69 years	4.84	3.86	5.99
Senegal	Both	70-74 years	6.26	4.86	7.84	Senegal	Male	80-84	9.07	7.30	11.33	Senegal	Female	70-74 years	6.94	5.30	8.64
Senegal	Both	75-79 years	8.29	6.66	10.05	Senegal	Male	85-89	10.63	8.63	12.99	Senegal	Female	75-79 years	9.16	7.30	11.09
Senegal	Both	All ages	0.44	0.38	0.51	Senegal	Male	90-94	12.03	9.84	14.50	Senegal	Female	All ages	0.50	0.43	0.58
Serbia	Both	40-44 years	0.49	0.36	0.66	Serbia	Male	80-84	12.66	10.36	15.66	Serbia	Female	80-84	15.80	12.82	19.14
Serbia	Both	45-49 years	1.03	0.81	1.30	Serbia	Male	85-89	14.56	11.88	17.80	Serbia	Female	85-89	17.73	14.44	21.26

Serbia	Both	50-54 years	1.83	1.38	2.33	Serbia	Male	90-94	16.15	13.20	19.54	Serbia	Female	90-94	19.01	15.65	22.45
Serbia	Both	55-59 years	2.91	2.30	3.54	Serbia	Male	40-44 years	0.45	0.33	0.62	Serbia	Female	40-44 years	0.53	0.38	0.71
Serbia	Both	60-64 years	4.29	3.37	5.43	Serbia	Male	45-49 years	0.94	0.73	1.17	Serbia	Female	45-49 years	1.13	0.88	1.43
Serbia	Both	65-69 years	6.45	5.18	7.90	Serbia	Male	50-54 years	1.63	1.25	2.07	Serbia	Female	50-54 years	2.02	1.51	2.60
Serbia	Both	70-74 years	9.20	7.21	11.61	Serbia	Male	55-59 years	2.57	2.05	3.15	Serbia	Female	55-59 years	3.22	2.52	3.98
Serbia	Both	75-79 years	11.95	9.60	14.69	Serbia	Male	60-64 years	3.77	2.98	4.69	Serbia	Female	60-64 years	4.76	3.68	6.06
Serbia	Both	All ages	2.63	2.25	3.04	Serbia	Male	65-69 years	5.62	4.55	6.89	Serbia	Female	65-69 years	7.17	5.76	8.83
Serbia	Both	80-84	14.55	11.89	17.66	Serbia	Male	70-74 years	7.98	6.22	10.02	Serbia	Female	70-74 years	10.21	7.96	13.04
Serbia	Both	85-89	16.63	13.64	20.02	Serbia	Male	75-79 years	10.38	8.39	12.72	Serbia	Female	75-79 years	13.16	10.38	16.26
Serbia	Both	90-94	17.71	14.63	21.16	Serbia	Male	All ages	2.10	1.80	2.42	Serbia	Female	All ages	3.13	2.68	3.64
Seychelles	Both	40-44 years	0.56	0.41	0.74	Seychelles	Male	40-44 years	0.49	0.37	0.66	Seychelles	Female	40-44 years	0.63	0.46	0.84
Seychelles	Both	45-49 years	1.22	0.95	1.53	Seychelles	Male	45-49 years	1.05	0.82	1.33	Seychelles	Female	45-49 years	1.41	1.09	1.78
Seychelles	Both	50-54 years	2.20	1.69	2.81	Seychelles	Male	50-54 years	1.86	1.44	2.37	Seychelles	Female	50-54 years	2.57	1.95	3.31
Seychelles	Both	55-59 years	3.45	2.72	4.21	Seychelles	Male	55-59 years	2.87	2.28	3.48	Seychelles	Female	55-59 years	4.05	3.17	4.98
Seychelles	Both	60-64 years	4.93	3.90	6.00	Seychelles	Male	60-64 years	4.05	3.19	4.97	Seychelles	Female	60-64 years	5.84	4.62	7.13
Seychelles	Both	65-69 years	7.02	5.68	8.49	Seychelles	Male	65-69 years	5.64	4.52	6.86	Seychelles	Female	65-69 years	8.41	6.86	10.23
Seychelles	Both	70-74 years	9.64	7.59	11.79	Seychelles	Male	70-74 years	7.57	5.97	9.28	Seychelles	Female	70-74 years	11.57	9.07	14.27
Seychelles	Both	75-79 years	12.54	10.05	15.23	Seychelles	Male	75-79 years	9.54	7.76	11.64	Seychelles	Female	75-79 years	14.68	11.76	17.78
Seychelles	Both	All ages	1.65	1.42	1.89	Seychelles	Male	All ages	1.19	1.01	1.36	Seychelles	Female	All ages	2.15	1.86	2.46
Seychelles	Both	80-84	15.39	12.53	18.64	Seychelles	Male	80-84	11.44	9.30	14.04	Seychelles	Female	80-84	17.52	14.18	21.17
Seychelles	Both	85-89	17.59	14.53	21.00	Seychelles	Male	85-89	13.05	10.80	15.77	Seychelles	Female	85-89	19.65	16.18	23.43
Seychelles	Both	90-94	19.27	16.11	22.64	Seychelles	Male	90-94	14.43	11.93	17.20	Seychelles	Female	90-94	21.16	17.70	24.91
Sierra Leone	Both	45-49 years	0.70	0.54	0.89	Sierra Leone	Male	45-49 years	0.65	0.50	0.83	Sierra Leone	Female	45-49 years	0.76	0.57	0.97
Sierra Leone	Both	50-54 years	1.28	0.96	1.65	Sierra Leone	Male	50-54 years	1.18	0.87	1.54	Sierra Leone	Female	50-54 years	1.39	1.04	1.80

Sierra Leone	Both	55-59 years	2.07	1.60	2.54	Sierra Leone	Male	55-59 years	1.90	1.47	2.37	Sierra Leone	Female	55-59 years	2.26	1.73	2.77
Sierra Leone	Both	60-64 years	3.07	2.41	3.84	Sierra Leone	Male	60-64 years	2.80	2.18	3.54	Sierra Leone	Female	60-64 years	3.37	2.64	4.23
Sierra Leone	Both	65-69 years	4.62	3.71	5.70	Sierra Leone	Male	65-69 years	4.15	3.32	5.16	Sierra Leone	Female	65-69 years	5.10	4.07	6.27
Sierra Leone	Both	70-74 years	6.62	5.15	8.31	Sierra Leone	Male	70-74 years	5.89	4.52	7.44	Sierra Leone	Female	70-74 years	7.34	5.64	9.32
Sierra Leone	Both	75-79 years	8.76	7.08	10.73	Sierra Leone	Male	75-79 years	7.74	6.28	9.46	Sierra Leone	Female	75-79 years	9.67	7.70	11.93
Sierra Leone	Both	All ages	0.40	0.34	0.46	Sierra Leone	Male	All ages	0.36	0.31	0.42	Sierra Leone	Female	All ages	0.43	0.37	0.50
Sierra Leone	Both	80-84	10.83	8.73	13.30	Sierra Leone	Male	80-84	9.57	7.70	11.74	Sierra Leone	Female	80-84	11.90	9.51	14.69
Sierra Leone	Both	85-89	12.52	10.17	15.10	Sierra Leone	Male	85-89	11.14	9.02	13.50	Sierra Leone	Female	85-89	13.64	11.03	16.51
Sierra Leone	Both	90-94	13.90	11.46	16.79	Sierra Leone	Male	90-94	12.46	10.12	15.07	Sierra Leone	Female	90-94	14.95	12.24	18.09
Sierra Leone	Both	40-44 years	0.32	0.23	0.43	Sierra Leone	Male	40-44 years	0.30	0.21	0.40	Sierra Leone	Female	40-44 years	0.34	0.24	0.46
Singapore	Both	40-44 years	0.54	0.41	0.72	Singapore	Male	40-44 years	0.26	0.19	0.35	Singapore	Female	40-44 years	0.78	0.59	1.05
Singapore	Both	45-49 years	0.87	0.68	1.09	Singapore	Male	45-49 years	0.54	0.42	0.69	Singapore	Female	45-49 years	1.33	1.06	1.68
Singapore	Both	50-54 years	1.45	1.12	1.83	Singapore	Male	50-54 years	0.96	0.73	1.21	Singapore	Female	50-54 years	1.95	1.51	2.50
Singapore	Both	55-59 years	2.13	1.69	2.59	Singapore	Male	55-59 years	1.60	1.27	1.95	Singapore	Female	55-59 years	2.75	2.18	3.35
Singapore	Both	60-64 years	3.14	2.52	3.85	Singapore	Male	60-64 years	2.43	1.91	2.98	Singapore	Female	60-64 years	3.89	3.11	4.77
Singapore	Both	65-69 years	4.75	3.85	5.72	Singapore	Male	65-69 years	3.60	2.87	4.36	Singapore	Female	65-69 years	5.97	4.85	7.23
Singapore	Both	70-74 years	6.92	5.42	8.58	Singapore	Male	70-74 years	5.02	3.94	6.23	Singapore	Female	70-74 years	8.78	6.84	10.94
Singapore	Both	75-79 years	9.48	7.67	11.47	Singapore	Male	75-79 years	6.51	5.21	7.94	Singapore	Female	75-79 years	11.79	9.48	14.29
Singapore	Both	All ages	1.57	1.36	1.77	Singapore	Male	All ages	1.05	0.89	1.20	Singapore	Female	All ages	2.09	1.81	2.37
Singapore	Both	80-84	12.01	9.75	14.60	Singapore	Male	80-84	8.03	6.45	9.91	Singapore	Female	80-84	14.87	12.05	18.07
Singapore	Both	85-89	14.56	12.04	17.43	Singapore	Male	85-89	9.51	7.81	11.57	Singapore	Female	85-89	17.59	14.57	21.11
Singapore	Both	90-94	16.95	14.17	19.94	Singapore	Male	90-94	10.91	8.97	13.22	Singapore	Female	90-94	19.78	16.50	23.33
Slovakia	Both	40-44 years	0.42	0.31	0.56	Slovakia	Male	60-64 years	3.19	2.50	3.96	Slovakia	Female	40-44 years	0.45	0.33	0.61
Slovakia	Both	45-49 years	0.88	0.68	1.10	Slovakia	Male	65-69 years	4.72	3.80	5.74	Slovakia	Female	45-49 years	0.96	0.74	1.21

Slovakia	Both	50-54 years	1.55	1.19	1.98	Slovakia	Male	70-74 years	6.68	5.19	8.36	Slovakia	Female	50-54 years	1.70	1.30	2.21
Slovakia	Both	55-59 years	2.46	1.93	2.99	Slovakia	Male	75-79 years	8.76	6.97	10.72	Slovakia	Female	55-59 years	2.72	2.12	3.34
Slovakia	Both	60-64 years	3.64	2.86	4.50	Slovakia	Male	All ages	1.55	1.33	1.77	Slovakia	Female	60-64 years	4.04	3.15	5.00
Slovakia	Both	65-69 years	5.47	4.43	6.66	Slovakia	Male	80-84	10.84	8.62	13.22	Slovakia	Female	65-69 years	6.07	4.92	7.41
Slovakia	Both	70-74 years	7.83	6.09	9.74	Slovakia	Male	85-89	12.71	10.36	15.25	Slovakia	Female	70-74 years	8.64	6.73	10.77
Slovakia	Both	75-79 years	10.31	8.16	12.61	Slovakia	Male	90-94	14.41	11.89	17.20	Slovakia	Female	75-79 years	11.21	8.85	13.63
Slovakia	Both	All ages	2.10	1.79	2.40	Slovakia	Male	40-44 years	0.39	0.28	0.53	Slovakia	Female	All ages	2.61	2.23	2.98
Slovakia	Both	80-84	12.72	10.36	15.49	Slovakia	Male	45-49 years	0.80	0.62	1.00	Slovakia	Female	80-84	13.63	11.00	16.67
Slovakia	Both	85-89	14.76	12.21	17.68	Slovakia	Male	50-54 years	1.39	1.06	1.77	Slovakia	Female	85-89	15.61	12.91	18.71
Slovakia	Both	90-94	16.48	13.62	19.59	Slovakia	Male	55-59 years	2.18	1.72	2.68	Slovakia	Female	90-94	17.17	14.17	20.44
Slovenia	Both	40-44 years	0.43	0.32	0.57	Slovenia	Male	40-44 years	0.40	0.30	0.54	Slovenia	Female	40-44 years	0.46	0.33	0.62
Slovenia	Both	45-49 years	0.90	0.70	1.14	Slovenia	Male	45-49 years	0.82	0.64	1.03	Slovenia	Female	45-49 years	0.98	0.76	1.26
Slovenia	Both	50-54 years	1.59	1.22	2.03	Slovenia	Male	50-54 years	1.42	1.08	1.80	Slovenia	Female	50-54 years	1.76	1.33	2.28
Slovenia	Both	55-59 years	2.55	2.02	3.10	Slovenia	Male	55-59 years	2.26	1.79	2.75	Slovenia	Female	55-59 years	2.84	2.20	3.50
Slovenia	Both	60-64 years	3.80	3.02	4.71	Slovenia	Male	60-64 years	3.34	2.66	4.16	Slovenia	Female	60-64 years	4.26	3.33	5.29
Slovenia	Both	65-69 years	5.69	4.62	6.94	Slovenia	Male	65-69 years	4.95	3.99	6.05	Slovenia	Female	65-69 years	6.41	5.17	7.84
Slovenia	Both	70-74 years	8.13	6.36	10.14	Slovenia	Male	70-74 years	6.98	5.39	8.72	Slovenia	Female	70-74 years	9.10	7.06	11.39
Slovenia	Both	75-79 years	10.66	8.61	13.08	Slovenia	Male	75-79 years	9.13	7.25	11.08	Slovenia	Female	75-79 years	11.79	9.46	14.48
Slovenia	Both	All ages	2.73	2.36	3.12	Slovenia	Male	All ages	2.04	1.76	2.34	Slovenia	Female	All ages	3.38	2.93	3.89
Slovenia	Both	80-84	13.15	10.72	15.87	Slovenia	Male	80-84	11.27	9.08	13.82	Slovenia	Female	80-84	14.29	11.59	17.15
Slovenia	Both	85-89	15.36	12.66	18.42	Slovenia	Male	85-89	13.18	10.79	15.93	Slovenia	Female	85-89	16.28	13.42	19.56
Slovenia	Both	90-94	17.20	14.27	20.47	Slovenia	Male	90-94	14.93	12.24	17.86	Slovenia	Female	90-94	17.80	14.81	21.29
Solomon Islands	Both	40-44 years	0.58	0.44	0.77	Solomon Islands	Male	40-44 years	0.52	0.39	0.71	Solomon Islands	Female	40-44 years	0.64	0.48	0.84
Solomon Islands	Both	45-49 years	1.27	0.99	1.59	Solomon Islands	Male	45-49 years	1.12	0.87	1.42	Solomon Islands	Female	45-49 years	1.42	1.11	1.79

Solomon Islands	Both	50-54 years	2.26	1.74	2.88	Solomon Islands	Male	50-54 years	1.97	1.50	2.53	Solomon Islands	Female	50-54 years	2.56	1.97	3.27
Solomon Islands	Both	55-59 years	3.46	2.71	4.22	Solomon Islands	Male	55-59 years	3.02	2.38	3.69	Solomon Islands	Female	55-59 years	3.94	3.08	4.87
Solomon Islands	Both	60-64 years	4.85	3.82	6.03	Solomon Islands	Male	60-64 years	4.20	3.31	5.21	Solomon Islands	Female	60-64 years	5.55	4.34	6.91
Solomon Islands	Both	65-69 years	6.91	5.56	8.45	Solomon Islands	Male	65-69 years	5.85	4.71	7.21	Solomon Islands	Female	65-69 years	8.02	6.41	9.86
Solomon Islands	Both	70-74 years	9.51	7.43	11.83	Solomon Islands	Male	70-74 years	7.87	6.12	9.79	Solomon Islands	Female	70-74 years	11.14	8.68	13.97
Solomon Islands	Both	75-79 years	11.95	9.64	14.48	Solomon Islands	Male	75-79 years	9.80	7.88	11.90	Solomon Islands	Female	75-79 years	14.07	11.22	17.08
Solomon Islands	Both	All ages	0.63	0.53	0.72	Solomon Islands	Male	All ages	0.54	0.46	0.62	Solomon Islands	Female	All ages	0.72	0.61	0.83
Solomon Islands	Both	80-84	13.92	11.46	16.71	Solomon Islands	Male	80-84	11.50	9.43	13.93	Solomon Islands	Female	80-84	16.56	13.56	20.11
Solomon Islands	Both	85-89	15.20	12.55	18.26	Solomon Islands	Male	85-89	12.73	10.36	15.26	Solomon Islands	Female	85-89	18.11	14.96	21.89
Solomon Islands	Both	90-94	15.89	13.12	19.20	Solomon Islands	Male	90-94	13.59	11.12	16.33	Solomon Islands	Female	90-94	18.89	15.64	22.70
Somalia	Both	80-84	10.74	8.73	13.10	Somalia	Male	40-44 years	0.27	0.20	0.36	Somalia	Female	80-84	11.74	9.52	14.39
Somalia	Both	85-89	12.40	10.18	15.04	Somalia	Male	45-49 years	0.59	0.46	0.75	Somalia	Female	85-89	13.36	11.01	16.19
Somalia	Both	90-94	13.66	11.35	16.33	Somalia	Male	50-54 years	1.06	0.80	1.36	Somalia	Female	90-94	14.49	12.04	17.35
Somalia	Both	40-44 years	0.31	0.22	0.41	Somalia	Male	55-59 years	1.70	1.32	2.09	Somalia	Female	40-44 years	0.34	0.25	0.46
Somalia	Both	45-49 years	0.67	0.52	0.86	Somalia	Male	60-64 years	2.51	1.96	3.15	Somalia	Female	45-49 years	0.75	0.57	0.97
Somalia	Both	50-54 years	1.22	0.92	1.59	Somalia	Male	65-69 years	3.73	2.97	4.65	Somalia	Female	50-54 years	1.37	1.02	1.79
Somalia	Both	55-59 years	1.99	1.55	2.44	Somalia	Male	70-74 years	5.30	4.03	6.72	Somalia	Female	55-59 years	2.21	1.73	2.74
Somalia	Both	60-64 years	2.96	2.32	3.71	Somalia	Male	75-79 years	6.97	5.60	8.48	Somalia	Female	60-64 years	3.29	2.57	4.15
Somalia	Both	65-69 years	4.49	3.57	5.56	Somalia	Male	All ages	0.19	0.16	0.22	Somalia	Female	65-69 years	5.02	3.98	6.25
Somalia	Both	70-74 years	6.48	4.96	8.14	Somalia	Male	80-84	8.62	6.88	10.45	Somalia	Female	70-74 years	7.26	5.55	9.17
Somalia	Both	75-79 years	8.65	6.93	10.53	Somalia	Male	85-89	10.03	8.20	12.20	Somalia	Female	75-79 years	9.57	7.69	11.69
Somalia	Both	All ages	0.27	0.23	0.31	Somalia	Male	90-94	11.24	9.28	13.59	Somalia	Female	All ages	0.36	0.30	0.41
South Africa	Both	50-54 years	1.58	1.20	2.02	South Africa	Male	40-44 years	0.27	0.20	0.35	South Africa	Female	40-44 years	0.43	0.32	0.57
South Africa	Both	55-59 years	2.64	2.07	3.22	South Africa	Male	45-49 years	0.61	0.47	0.77	South Africa	Female	45-49 years	1.02	0.78	1.28

South Africa	Both	60-64 years	4.01	3.14	4.96	South Africa	Male	50-54 years	1.14	0.87	1.46	South Africa	Female	50-54 years	1.95	1.48	2.50
South Africa	Both	65-69 years	6.08	4.92	7.41	South Africa	Male	55-59 years	1.87	1.46	2.29	South Africa	Female	55-59 years	3.26	2.56	3.99
South Africa	Both	70-74 years	8.72	6.78	10.87	South Africa	Male	60-64 years	2.78	2.19	3.45	South Africa	Female	60-64 years	4.96	3.88	6.16
South Africa	Both	75-79 years	11.68	9.39	14.28	South Africa	Male	65-69 years	4.11	3.33	5.02	South Africa	Female	65-69 years	7.54	6.10	9.20
South Africa	Both	All ages	1.01	0.88	1.16	South Africa	Male	70-74 years	5.77	4.48	7.19	South Africa	Female	70-74 years	10.78	8.40	13.44
South Africa	Both	80-84	14.80	12.12	18.03	South Africa	Male	75-79 years	7.60	6.10	9.32	South Africa	Female	75-79 years	14.13	11.37	17.33
South Africa	Both	85-89	17.29	14.28	20.79	South Africa	Male	All ages	0.58	0.50	0.66	South Africa	Female	All ages	1.42	1.24	1.62
South Africa	Both	90-94	19.21	15.98	22.73	South Africa	Male	80-84	9.51	7.69	11.71	South Africa	Female	80-84	17.34	14.20	21.06
South Africa	Both	40-44 years	0.35	0.26	0.46	South Africa	Male	85-89	11.28	9.30	13.59	South Africa	Female	85-89	19.75	16.35	23.76
South Africa	Both	45-49 years	0.82	0.63	1.04	South Africa	Male	90-94	12.93	10.65	15.43	South Africa	Female	90-94	21.39	17.79	25.34
South Sudan	Both	45-49 years	0.67	0.51	0.85	South Sudan	Male	45-49 years	0.59	0.45	0.75	South Sudan	Female	40-44 years	0.34	0.24	0.45
South Sudan	Both	50-54 years	1.22	0.92	1.55	South Sudan	Male	50-54 years	1.07	0.80	1.39	South Sudan	Female	45-49 years	0.74	0.57	0.95
South Sudan	Both	55-59 years	1.98	1.55	2.42	South Sudan	Male	55-59 years	1.74	1.36	2.16	South Sudan	Female	50-54 years	1.37	1.03	1.74
South Sudan	Both	60-64 years	2.96	2.34	3.69	South Sudan	Male	60-64 years	2.59	2.03	3.26	South Sudan	Female	55-59 years	2.25	1.77	2.75
South Sudan	Both	65-69 years	4.43	3.58	5.45	South Sudan	Male	65-69 years	3.84	3.03	4.73	South Sudan	Female	60-64 years	3.40	2.69	4.24
South Sudan	Both	70-74 years	6.32	4.91	7.85	South Sudan	Male	70-74 years	5.43	4.18	6.84	South Sudan	Female	65-69 years	5.13	4.15	6.32
South Sudan	Both	75-79 years	8.36	6.73	10.24	South Sudan	Male	75-79 years	7.20	5.73	8.80	South Sudan	Female	70-74 years	7.34	5.68	9.16
South Sudan	Both	All ages	0.37	0.31	0.42	South Sudan	Male	All ages	0.34	0.29	0.39	South Sudan	Female	75-79 years	9.75	7.88	11.93
South Sudan	Both	80-84	10.48	8.40	12.82	South Sudan	Male	80-84	9.03	7.22	11.13	South Sudan	Female	All ages	0.39	0.34	0.45
South Sudan	Both	85-89	12.40	10.19	14.91	South Sudan	Male	85-89	10.75	8.77	13.04	South Sudan	Female	80-84	12.20	9.79	14.81
South Sudan	Both	90-94	14.35	11.93	17.13	South Sudan	Male	90-94	12.38	10.24	15.00	South Sudan	Female	85-89	14.28	11.67	17.23
South Sudan	Both	40-44 years	0.31	0.22	0.41	South Sudan	Male	40-44 years	0.27	0.19	0.36	South Sudan	Female	90-94	16.02	13.34	19.02
Spain	Both	80-84	19.22	15.77	23.24	Spain	Male	40-44 years	0.50	0.38	0.66	Spain	Female	80-84	21.96	17.98	26.62
Spain	Both	85-89	21.96	18.22	26.16	Spain	Male	45-49 years	1.04	0.81	1.32	Spain	Female	85-89	24.78	20.61	29.46

Spain	Both	90-94	24.01	20.15	28.35	Spain	Male	50-54 years	1.84	1.41	2.34	Spain	Female	90-94	26.56	22.24	31.30
Spain	Both	40-44 years	0.96	0.73	1.26	Spain	Male	55-59 years	3.05	2.39	3.74	Spain	Female	40-44 years	1.42	1.07	1.83
Spain	Both	45-49 years	1.67	1.32	2.06	Spain	Male	60-64 years	4.67	3.66	5.78	Spain	Female	45-49 years	2.29	1.81	2.86
Spain	Both	50-54 years	2.52	1.98	3.13	Spain	Male	65-69 years	7.07	5.67	8.61	Spain	Female	50-54 years	3.17	2.49	3.98
Spain	Both	55-59 years	3.69	2.92	4.48	Spain	Male	70-74 years	10.00	7.85	12.36	Spain	Female	55-59 years	4.30	3.44	5.28
Spain	Both	60-64 years	5.35	4.27	6.56	Spain	Male	75-79 years	12.76	10.29	15.43	Spain	Female	60-64 years	5.99	4.81	7.34
Spain	Both	65-69 years	8.26	6.71	9.93	Spain	Male	All ages	2.93	2.50	3.36	Spain	Female	65-69 years	9.34	7.58	11.38
Spain	Both	70-74 years	12.07	9.45	15.01	Spain	Male	80-84	15.18	12.34	18.39	Spain	Female	70-74 years	13.85	10.82	17.56
Spain	Both	75-79 years	15.78	12.89	19.32	Spain	Male	85-89	16.98	13.93	20.62	Spain	Female	75-79 years	18.16	14.79	22.26
Spain	Both	All ages	4.06	3.51	4.63	Spain	Male	90-94	18.15	15.01	21.76	Spain	Female	All ages	5.10	4.42	5.84
Sri Lanka	Both	40-44 years	0.55	0.41	0.73	Sri Lanka	Male	40-44 years	0.48	0.35	0.64	Sri Lanka	Female	40-44 years	0.62	0.45	0.83
Sri Lanka	Both	45-49 years	1.20	0.93	1.52	Sri Lanka	Male	45-49 years	1.02	0.80	1.29	Sri Lanka	Female	45-49 years	1.37	1.06	1.73
Sri Lanka	Both	50-54 years	2.17	1.65	2.77	Sri Lanka	Male	50-54 years	1.81	1.37	2.31	Sri Lanka	Female	50-54 years	2.50	1.89	3.20
Sri Lanka	Both	55-59 years	3.41	2.67	4.12	Sri Lanka	Male	55-59 years	2.79	2.20	3.42	Sri Lanka	Female	55-59 years	3.95	3.07	4.85
Sri Lanka	Both	60-64 years	4.88	3.85	5.96	Sri Lanka	Male	60-64 years	3.92	3.13	4.79	Sri Lanka	Female	60-64 years	5.70	4.46	7.04
Sri Lanka	Both	65-69 years	6.94	5.65	8.41	Sri Lanka	Male	65-69 years	5.44	4.44	6.55	Sri Lanka	Female	65-69 years	8.17	6.60	9.89
Sri Lanka	Both	70-74 years	9.46	7.46	11.76	Sri Lanka	Male	70-74 years	7.26	5.76	9.02	Sri Lanka	Female	70-74 years	11.13	8.75	13.92
Sri Lanka	Both	75-79 years	11.94	9.68	14.60	Sri Lanka	Male	75-79 years	9.07	7.41	11.09	Sri Lanka	Female	75-79 years	13.99	11.25	17.27
Sri Lanka	Both	All ages	1.75	1.51	2.01	Sri Lanka	Male	All ages	1.27	1.09	1.47	Sri Lanka	Female	All ages	2.19	1.88	2.51
Sri Lanka	Both	80-84	14.26	11.79	17.31	Sri Lanka	Male	80-84	10.75	8.74	13.06	Sri Lanka	Female	80-84	16.52	13.57	20.16
Sri Lanka	Both	85-89	16.09	13.40	19.35	Sri Lanka	Male	85-89	12.11	9.94	14.55	Sri Lanka	Female	85-89	18.34	15.28	22.21
Sri Lanka	Both	90-94	17.43	14.50	20.63	Sri Lanka	Male	90-94	13.23	11.00	15.63	Sri Lanka	Female	90-94	19.53	16.24	23.29
Sudan	Both	40-44 years	0.35	0.26	0.47	Sudan	Male	80-84	8.43	6.77	10.29	Sudan	Female	80-84	10.94	8.86	13.42
Sudan	Both	45-49 years	0.73	0.56	0.91	Sudan	Male	85-89	9.85	7.97	11.83	Sudan	Female	85-89	12.50	10.13	15.14

Sudan	Both	50-54 years	1.27	0.96	1.60	Sudan	Male	90-94	11.14	9.14	13.37	Sudan	Female	90-94	13.74	11.24	16.55
Sudan	Both	55-59 years	1.97	1.56	2.39	Sudan	Male	40-44 years	0.31	0.23	0.42	Sudan	Female	40-44 years	0.38	0.28	0.52
Sudan	Both	60-64 years	2.85	2.27	3.52	Sudan	Male	45-49 years	0.64	0.50	0.80	Sudan	Female	45-49 years	0.81	0.63	1.02
Sudan	Both	65-69 years	4.21	3.39	5.12	Sudan	Male	50-54 years	1.12	0.85	1.41	Sudan	Female	50-54 years	1.43	1.08	1.82
Sudan	Both	70-74 years	5.97	4.59	7.42	Sudan	Male	55-59 years	1.74	1.36	2.14	Sudan	Female	55-59 years	2.25	1.76	2.73
Sudan	Both	75-79 years	7.79	6.21	9.46	Sudan	Male	60-64 years	2.51	1.99	3.12	Sudan	Female	60-64 years	3.27	2.58	4.07
Sudan	Both	All ages	0.41	0.35	0.46	Sudan	Male	65-69 years	3.70	2.95	4.49	Sudan	Female	65-69 years	4.87	3.88	5.99
Sudan	Both	80-84	9.57	7.75	11.72	Sudan	Male	70-74 years	5.23	4.05	6.50	Sudan	Female	70-74 years	6.93	5.32	8.67
Sudan	Both	85-89	11.10	9.12	13.29	Sudan	Male	75-79 years	6.84	5.45	8.27	Sudan	Female	75-79 years	9.01	7.06	10.98
Sudan	Both	90-94	12.48	10.26	14.85	Sudan	Male	All ages	0.39	0.33	0.44	Sudan	Female	All ages	0.42	0.36	0.49
Suriname	Both	45-49 years	0.72	0.56	0.91	Suriname	Male	45-49 years	0.60	0.46	0.77	Suriname	Female	40-44 years	0.39	0.28	0.52
Suriname	Both	50-54 years	1.27	0.96	1.64	Suriname	Male	50-54 years	1.06	0.80	1.36	Suriname	Female	45-49 years	0.83	0.64	1.06
Suriname	Both	55-59 years	2.04	1.59	2.55	Suriname	Male	55-59 years	1.70	1.31	2.11	Suriname	Female	50-54 years	1.48	1.12	1.93
Suriname	Both	60-64 years	3.09	2.43	3.86	Suriname	Male	60-64 years	2.55	1.98	3.22	Suriname	Female	55-59 years	2.38	1.85	2.96
Suriname	Both	65-69 years	4.87	3.92	6.00	Suriname	Male	65-69 years	3.99	3.14	4.93	Suriname	Female	60-64 years	3.60	2.79	4.48
Suriname	Both	70-74 years	7.24	5.60	9.12	Suriname	Male	70-74 years	5.90	4.49	7.47	Suriname	Female	65-69 years	5.63	4.52	6.90
Suriname	Both	75-79 years	9.65	7.63	11.85	Suriname	Male	75-79 years	7.88	6.26	9.69	Suriname	Female	70-74 years	8.30	6.39	10.41
Suriname	Both	All ages	1.11	0.95	1.27	Suriname	Male	All ages	0.84	0.71	0.95	Suriname	Female	75-79 years	11.00	8.66	13.61
Suriname	Both	80-84	11.95	9.67	14.67	Suriname	Male	80-84	9.77	7.90	12.09	Suriname	Female	All ages	1.37	1.17	1.57
Suriname	Both	85-89	13.83	11.23	16.82	Suriname	Male	85-89	11.36	9.26	13.78	Suriname	Female	80-84	13.50	10.84	16.55
Suriname	Both	90-94	15.28	12.60	18.31	Suriname	Male	90-94	12.71	10.31	15.32	Suriname	Female	85-89	15.34	12.47	18.80
Suriname	Both	40-44 years	0.34	0.25	0.45	Suriname	Male	40-44 years	0.29	0.21	0.39	Suriname	Female	90-94	16.61	13.59	19.84
Sweden	Both	40-44 years	0.94	0.71	1.23	Sweden	Male	50-54 years	1.98	1.53	2.52	Sweden	Female	40-44 years	1.42	1.07	1.86
Sweden	Both	45-49 years	1.74	1.36	2.15	Sweden	Male	55-59 years	3.36	2.67	4.09	Sweden	Female	45-49 years	2.42	1.89	3.01

Sweden	Both	50-54 years	2.75	2.14	3.43	Sweden	Male	60-64 years	5.20	4.10	6.49	Sweden	Female	50-54 years	3.54	2.71	4.42
Sweden	Both	55-59 years	4.13	3.26	4.98	Sweden	Male	65-69 years	7.80	6.33	9.50	Sweden	Female	55-59 years	4.91	3.88	5.95
Sweden	Both	60-64 years	6.01	4.84	7.41	Sweden	Male	70-74 years	10.91	8.44	13.59	Sweden	Female	60-64 years	6.83	5.49	8.29
Sweden	Both	65-69 years	9.17	7.47	11.03	Sweden	Male	75-79 years	13.85	11.18	16.99	Sweden	Female	65-69 years	10.50	8.56	12.55
Sweden	Both	70-74 years	13.21	10.33	16.46	Sweden	Male	All ages	3.25	2.75	3.73	Sweden	Female	70-74 years	15.40	12.04	19.17
Sweden	Both	75-79 years	17.16	13.78	20.95	Sweden	Male	80-84	16.47	13.47	20.04	Sweden	Female	75-79 years	20.20	16.21	24.50
Sweden	Both	All ages	4.31	3.72	4.92	Sweden	Male	85-89	18.35	15.23	22.20	Sweden	Female	All ages	5.35	4.60	6.11
Sweden	Both	80-84	20.95	17.29	25.24	Sweden	Male	90-94	19.54	16.33	23.37	Sweden	Female	80-84	24.54	20.25	29.39
Sweden	Both	85-89	23.92	20.05	28.56	Sweden	Male	40-44 years	0.48	0.35	0.64	Sweden	Female	85-89	27.54	23.16	32.72
Sweden	Both	90-94	25.93	21.77	30.60	Sweden	Male	45-49 years	1.07	0.82	1.35	Sweden	Female	90-94	29.28	24.58	34.51
Switzerland	Both	40-44 years	1.02	0.77	1.35	Switzerland	Male	40-44 years	0.55	0.41	0.75	Switzerland	Female	40-44 years	1.49	1.11	1.94
Switzerland	Both	45-49 years	1.78	1.40	2.20	Switzerland	Male	45-49 years	1.15	0.89	1.46	Switzerland	Female	45-49 years	2.40	1.88	2.99
Switzerland	Both	50-54 years	2.67	2.07	3.34	Switzerland	Male	50-54 years	2.05	1.56	2.62	Switzerland	Female	50-54 years	3.30	2.54	4.12
Switzerland	Both	55-59 years	3.94	3.17	4.73	Switzerland	Male	55-59 years	3.43	2.73	4.19	Switzerland	Female	55-59 years	4.45	3.54	5.48
Switzerland	Both	60-64 years	5.75	4.64	7.03	Switzerland	Male	60-64 years	5.31	4.19	6.58	Switzerland	Female	60-64 years	6.19	4.99	7.59
Switzerland	Both	65-69 years	8.90	7.19	10.70	Switzerland	Male	65-69 years	8.06	6.49	9.76	Switzerland	Female	65-69 years	9.69	7.91	11.69
Switzerland	Both	70-74 years	13.00	10.06	16.18	Switzerland	Male	70-74 years	11.40	8.86	14.12	Switzerland	Female	70-74 years	14.44	11.09	18.07
Switzerland	Both	75-79 years	16.98	13.70	20.63	Switzerland	Male	75-79 years	14.55	11.69	17.59	Switzerland	Female	75-79 years	19.02	15.19	23.20
Switzerland	Both	All ages	4.13	3.58	4.70	Switzerland	Male	All ages	3.23	2.78	3.69	Switzerland	Female	All ages	4.99	4.33	5.69
Switzerland	Both	80-84	20.66	17.10	24.69	Switzerland	Male	80-84	17.32	14.23	20.87	Switzerland	Female	80-84	23.11	18.97	27.67
Switzerland	Both	85-89	23.62	19.67	28.11	Switzerland	Male	85-89	19.36	15.97	23.20	Switzerland	Female	85-89	26.17	21.84	31.22
Switzerland	Both	90-94	25.81	21.75	30.21	Switzerland	Male	90-94	20.66	17.13	24.34	Switzerland	Female	90-94	28.15	23.67	33.08
Syrian Arab Republic	Both	45-49 years	0.81	0.63	1.03	Syrian Arab Republic	Male	40-44 years	0.34	0.24	0.46	Syrian Arab Republic	Female	45-49 years	0.91	0.70	1.15
Syrian Arab Republic	Both	50-54 years	1.43	1.08	1.84	Syrian Arab Republic	Male	45-49 years	0.71	0.55	0.91	Syrian Arab Republic	Female	50-54 years	1.62	1.22	2.07

Syrian Arab Republic	Both	55-59 years	2.25	1.76	2.75	Syrian Arab Republic	Male	50-54 years	1.25	0.95	1.61	Syrian Arab Republic	Female	55-59 years	2.56	1.98	3.17
Syrian Arab Republic	Both	60-64 years	3.28	2.56	4.02	Syrian Arab Republic	Male	55-59 years	1.96	1.52	2.42	Syrian Arab Republic	Female	60-64 years	3.74	2.93	4.64
Syrian Arab Republic	Both	65-69 years	4.88	3.89	5.90	Syrian Arab Republic	Male	60-64 years	2.84	2.21	3.53	Syrian Arab Republic	Female	65-69 years	5.58	4.45	6.79
Syrian Arab Republic	Both	70-74 years	6.93	5.30	8.57	Syrian Arab Republic	Male	65-69 years	4.20	3.34	5.13	Syrian Arab Republic	Female	70-74 years	7.91	6.02	9.83
Syrian Arab Republic	Both	75-79 years	9.00	7.26	11.02	Syrian Arab Republic	Male	70-74 years	5.96	4.58	7.52	Syrian Arab Republic	Female	75-79 years	10.24	8.23	12.56
Syrian Arab Republic	Both	All ages	0.88	0.76	1.01	Syrian Arab Republic	Male	75-79 years	7.78	6.22	9.53	Syrian Arab Republic	Female	All ages	0.93	0.79	1.06
Syrian Arab Republic	Both	80-84	10.88	8.81	13.25	Syrian Arab Republic	Male	All ages	0.83	0.71	0.96	Syrian Arab Republic	Female	80-84	12.38	10.04	14.98
Syrian Arab Republic	Both	85-89	12.27	10.02	14.73	Syrian Arab Republic	Male	80-84	9.55	7.70	11.66	Syrian Arab Republic	Female	85-89	14.05	11.47	17.09
Syrian Arab Republic	Both	90-94	12.95	10.68	15.59	Syrian Arab Republic	Male	85-89	11.08	8.99	13.54	Syrian Arab Republic	Female	90-94	15.28	12.59	18.24
Syrian Arab Republic	Both	40-44 years	0.39	0.28	0.53	Syrian Arab Republic	Male	90-94	12.41	10.18	15.02	Syrian Arab Republic	Female	40-44 years	0.43	0.31	0.59
Taiwan (Province of China)	Both	40-44 years	0.69	0.51	0.90	Taiwan (Province of China)	Male	40-44 years	0.56	0.40	0.78	Taiwan (Province of China)	Female	80-84	16.09	13.56	19.34
Taiwan (Province of China)	Both	45-49 years	1.63	1.29	1.97	Taiwan (Province of China)	Male	45-49 years	1.30	0.99	1.63	Taiwan (Province of China)	Female	85-89	17.50	14.72	21.18
Taiwan (Province of China)	Both	50-54 years	3.01	2.32	3.71	Taiwan (Province of China)	Male	50-54 years	2.37	1.77	2.99	Taiwan (Province of China)	Female	90-94	18.52	15.33	22.35
Taiwan (Province of China)	Both	55-59 years	4.48	3.59	5.35	Taiwan (Province of China)	Male	55-59 years	3.52	2.74	4.29	Taiwan (Province of China)	Female	40-44 years	0.81	0.60	1.05
Taiwan (Province of China)	Both	60-64 years	5.95	4.97	7.03	Taiwan (Province of China)	Male	60-64 years	4.65	3.75	5.74	Taiwan (Province of China)	Female	45-49 years	1.95	1.54	2.38
Taiwan (Province of China)	Both	65-69 years	7.81	6.58	9.11	Taiwan (Province of China)	Male	65-69 years	6.06	5.01	7.27	Taiwan (Province of China)	Female	50-54 years	3.62	2.80	4.53
Taiwan (Province of China)	Both	70-74 years	9.93	8.19	11.73	Taiwan (Province of China)	Male	70-74 years	7.68	6.25	9.17	Taiwan (Province of China)	Female	55-59 years	5.40	4.35	6.45

Taiwan (Province of China)	Both	75-79 years	11.92	10.13	13.99	Taiwan (Province of China)	Male	75-79 years	9.10	7.54	10.69	Taiwan (Province of China)	Female	60-64 years	7.17	5.97	8.47
Taiwan (Province of China)	Both	All ages	2.89	2.55	3.20	Taiwan (Province of China)	Male	All ages	2.15	1.84	2.46	Taiwan (Province of China)	Female	65-69 years	9.38	7.92	10.97
Taiwan (Province of China)	Both	80-84	13.65	11.73	16.21	Taiwan (Province of China)	Male	80-84	10.26	8.65	12.06	Taiwan (Province of China)	Female	70-74 years	11.90	9.85	14.14
Taiwan (Province of China)	Both	85-89	14.79	12.75	17.52	Taiwan (Province of China)	Male	85-89	11.17	9.56	13.02	Taiwan (Province of China)	Female	75-79 years	14.18	12.05	16.99
Taiwan (Province of China)	Both	90-94	15.47	13.03	18.41	Taiwan (Province of China)	Male	90-94	11.95	10.28	13.93	Taiwan (Province of China)	Female	All ages	3.60	3.19	4.02
Tajikistan	Both	40-44 years	0.41	0.31	0.55	Tajikistan	Male	40-44 years	0.37	0.27	0.50	Tajikistan	Female	85-89	15.00	12.35	18.11
Tajikistan	Both	45-49 years	0.87	0.67	1.09	Tajikistan	Male	45-49 years	0.75	0.58	0.95	Tajikistan	Female	90-94	16.13	13.32	19.28
Tajikistan	Both	50-54 years	1.53	1.16	1.94	Tajikistan	Male	50-54 years	1.29	1.00	1.65	Tajikistan	Female	40-44 years	0.46	0.34	0.61
Tajikistan	Both	55-59 years	2.39	1.87	2.93	Tajikistan	Male	55-59 years	2.02	1.58	2.46	Tajikistan	Female	45-49 years	0.98	0.76	1.23
Tajikistan	Both	60-64 years	3.49	2.74	4.34	Tajikistan	Male	60-64 years	2.93	2.31	3.63	Tajikistan	Female	50-54 years	1.74	1.31	2.22
Tajikistan	Both	65-69 years	5.20	4.16	6.38	Tajikistan	Male	65-69 years	4.32	3.46	5.30	Tajikistan	Female	55-59 years	2.75	2.13	3.42
Tajikistan	Both	70-74 years	7.36	5.72	9.26	Tajikistan	Male	70-74 years	6.09	4.72	7.65	Tajikistan	Female	60-64 years	4.01	3.11	5.06
Tajikistan	Both	75-79 years	9.53	7.67	11.66	Tajikistan	Male	75-79 years	7.94	6.39	9.68	Tajikistan	Female	65-69 years	6.00	4.76	7.41
Tajikistan	Both	All ages	0.55	0.47	0.64	Tajikistan	Male	All ages	0.44	0.37	0.51	Tajikistan	Female	70-74 years	8.55	6.59	10.87
Tajikistan	Both	80-84	11.81	9.64	14.38	Tajikistan	Male	80-84	9.71	7.82	11.92	Tajikistan	Female	75-79 years	11.06	8.91	13.69
Tajikistan	Both	85-89	13.70	11.31	16.46	Tajikistan	Male	85-89	11.22	9.17	13.47	Tajikistan	Female	All ages	0.66	0.56	0.76
Tajikistan	Both	90-94	15.03	12.49	17.98	Tajikistan	Male	90-94	12.54	10.29	15.14	Tajikistan	Female	80-84	13.34	10.90	16.38
Thailand	Both	40-44 years	0.48	0.36	0.64	Thailand	Male	40-44 years	0.42	0.31	0.56	Thailand	Female	40-44 years	0.54	0.40	0.73
Thailand	Both	45-49 years	1.05	0.81	1.31	Thailand	Male	45-49 years	0.89	0.69	1.13	Thailand	Female	45-49 years	1.19	0.91	1.49
Thailand	Both	50-54 years	1.87	1.44	2.38	Thailand	Male	50-54 years	1.57	1.21	2.01	Thailand	Female	50-54 years	2.15	1.64	2.76
Thailand	Both	55-59 years	2.94	2.30	3.58	Thailand	Male	55-59 years	2.43	1.91	2.97	Thailand	Female	55-59 years	3.40	2.66	4.14
Thailand	Both	60-64 years	4.21	3.33	5.17	Thailand	Male	60-64 years	3.43	2.69	4.24	Thailand	Female	60-64 years	4.91	3.83	6.03

Thailand	Both	65-69 years	5.97	4.87	7.30	Thailand	Male	65-69 years	4.76	3.85	5.83	Thailand	Female	65-69 years	7.03	5.67	8.64
Thailand	Both	70-74 years	8.13	6.39	10.03	Thailand	Male	70-74 years	6.34	4.98	7.86	Thailand	Female	70-74 years	9.61	7.56	11.91
Thailand	Both	75-79 years	10.33	8.36	12.46	Thailand	Male	75-79 years	7.94	6.41	9.59	Thailand	Female	75-79 years	12.19	9.85	14.87
Thailand	Both	All ages	1.92	1.65	2.19	Thailand	Male	All ages	1.43	1.23	1.64	Thailand	Female	All ages	2.37	2.04	2.71
Thailand	Both	80-84	12.46	10.09	15.17	Thailand	Male	80-84	9.47	7.58	11.64	Thailand	Female	80-84	14.57	11.76	17.74
Thailand	Both	85-89	14.19	11.66	17.09	Thailand	Male	85-89	10.78	8.81	13.06	Thailand	Female	85-89	16.35	13.38	19.85
Thailand	Both	90-94	15.47	12.78	18.46	Thailand	Male	90-94	11.93	9.71	14.51	Thailand	Female	90-94	17.64	14.57	21.04
Timor-Leste	Both	All ages	0.86	0.73	0.99	Timor-Leste	Male	All ages	0.69	0.59	0.81	Timor-Leste	Female	40-44 years	0.63	0.46	0.84
Timor-Leste	Both	80-84	13.49	10.98	16.38	Timor-Leste	Male	80-84	10.76	8.76	13.15	Timor-Leste	Female	45-49 years	1.39	1.08	1.76
Timor-Leste	Both	85-89	15.22	12.60	18.28	Timor-Leste	Male	85-89	12.02	9.92	14.52	Timor-Leste	Female	50-54 years	2.53	1.91	3.24
Timor-Leste	Both	90-94	16.01	13.37	19.16	Timor-Leste	Male	90-94	13.00	10.78	15.54	Timor-Leste	Female	55-59 years	3.92	3.06	4.80
Timor-Leste	Both	40-44 years	0.55	0.41	0.74	Timor-Leste	Male	40-44 years	0.49	0.36	0.65	Timor-Leste	Female	60-64 years	5.55	4.30	6.83
Timor-Leste	Both	45-49 years	1.21	0.95	1.52	Timor-Leste	Male	45-49 years	1.04	0.82	1.31	Timor-Leste	Female	65-69 years	7.97	6.43	9.67
Timor-Leste	Both	50-54 years	2.16	1.66	2.77	Timor-Leste	Male	50-54 years	1.84	1.43	2.36	Timor-Leste	Female	70-74 years	10.99	8.55	13.76
Timor-Leste	Both	55-59 years	3.35	2.61	4.05	Timor-Leste	Male	55-59 years	2.84	2.23	3.45	Timor-Leste	Female	75-79 years	13.90	11.13	17.05
Timor-Leste	Both	60-64 years	4.77	3.77	5.86	Timor-Leste	Male	60-64 years	3.98	3.16	4.90	Timor-Leste	Female	All ages	1.02	0.87	1.17
Timor-Leste	Both	65-69 years	6.82	5.53	8.32	Timor-Leste	Male	65-69 years	5.52	4.44	6.77	Timor-Leste	Female	80-84	16.45	13.23	19.94
Timor-Leste	Both	70-74 years	9.30	7.25	11.50	Timor-Leste	Male	70-74 years	7.37	5.76	9.13	Timor-Leste	Female	85-89	18.15	14.85	21.95
Timor-Leste	Both	75-79 years	11.51	9.20	13.85	Timor-Leste	Male	75-79 years	9.16	7.33	11.08	Timor-Leste	Female	90-94	19.13	15.90	22.97
Togo	Both	40-44 years	0.30	0.22	0.40	Togo	Male	40-44 years	0.28	0.20	0.37	Togo	Female	40-44 years	0.33	0.24	0.44
Togo	Both	45-49 years	0.67	0.51	0.86	Togo	Male	45-49 years	0.61	0.47	0.79	Togo	Female	45-49 years	0.72	0.55	0.92
Togo	Both	50-54 years	1.21	0.92	1.55	Togo	Male	50-54 years	1.11	0.84	1.42	Togo	Female	50-54 years	1.31	0.99	1.68
Togo	Both	55-59 years	1.96	1.53	2.42	Togo	Male	55-59 years	1.78	1.39	2.19	Togo	Female	55-59 years	2.13	1.65	2.63
Togo	Both	60-64 years	2.92	2.28	3.66	Togo	Male	60-64 years	2.61	2.03	3.28	Togo	Female	60-64 years	3.18	2.46	4.00

Togo	Both	65-69 years	4.40	3.55	5.44	Togo	Male	65-69 years	3.87	3.10	4.79	Togo	Female	65-69 years	4.80	3.84	5.91
Togo	Both	70-74 years	6.39	4.94	8.08	Togo	Male	70-74 years	5.50	4.24	6.98	Togo	Female	70-74 years	6.90	5.30	8.78
Togo	Both	75-79 years	8.44	6.76	10.44	Togo	Male	75-79 years	7.23	5.79	8.85	Togo	Female	75-79 years	9.11	7.28	11.34
Togo	Both	All ages	0.39	0.34	0.46	Togo	Male	All ages	0.30	0.26	0.35	Togo	Female	All ages	0.48	0.41	0.56
Togo	Both	80-84	10.44	8.48	12.87	Togo	Male	80-84	8.95	7.21	11.03	Togo	Female	80-84	11.24	9.06	13.82
Togo	Both	85-89	12.22	9.96	14.90	Togo	Male	85-89	10.47	8.56	12.74	Togo	Female	85-89	12.95	10.50	15.84
Togo	Both	90-94	13.61	11.22	16.39	Togo	Male	90-94	11.81	9.64	14.24	Togo	Female	90-94	14.28	11.75	17.20
Tokelau	Both	40-44 years	0.62	0.46	0.81	Tokelau	Male	40-44 years	0.54	0.40	0.71	Tokelau	Female	40-44 years	0.67	0.49	0.88
Tokelau	Both	45-49 years	1.31	1.01	1.64	Tokelau	Male	45-49 years	1.17	0.91	1.46	Tokelau	Female	45-49 years	1.48	1.14	1.86
Tokelau	Both	50-54 years	2.38	1.80	3.04	Tokelau	Male	50-54 years	2.08	1.56	2.64	Tokelau	Female	50-54 years	2.68	2.01	3.46
Tokelau	Both	55-59 years	3.71	2.89	4.52	Tokelau	Male	55-59 years	3.21	2.56	3.88	Tokelau	Female	55-59 years	4.15	3.20	5.11
Tokelau	Both	60-64 years	5.08	4.00	6.22	Tokelau	Male	60-64 years	4.50	3.54	5.58	Tokelau	Female	60-64 years	5.87	4.57	7.21
Tokelau	Both	65-69 years	7.41	6.03	9.06	Tokelau	Male	65-69 years	6.25	5.09	7.64	Tokelau	Female	65-69 years	8.47	6.80	10.32
Tokelau	Both	70-74 years	9.79	7.58	12.21	Tokelau	Male	70-74 years	8.36	6.53	10.42	Tokelau	Female	70-74 years	11.73	9.10	14.70
Tokelau	Both	75-79 years	12.95	10.24	15.85	Tokelau	Male	75-79 years	10.39	8.32	12.75	Tokelau	Female	75-79 years	14.84	11.76	18.24
Tokelau	Both	All ages	1.51	1.30	1.74	Tokelau	Male	All ages	1.27	1.09	1.46	Tokelau	Female	All ages	1.76	1.51	2.03
Tokelau	Both	80-84	14.85	12.10	18.01	Tokelau	Male	80-84	12.22	9.78	15.00	Tokelau	Female	80-84	17.55	14.28	21.27
Tokelau	Both	85-89	16.79	13.88	20.23	Tokelau	Male	85-89	13.59	11.16	16.51	Tokelau	Female	85-89	19.37	15.87	23.44
Tokelau	Both	90-94	17.70	14.76	20.98	Tokelau	Male	90-94	14.59	12.10	17.38	Tokelau	Female	90-94	20.43	16.99	24.42
Tonga	Both	80-84	16.38	13.49	19.77	Tonga	Male	40-44 years	0.58	0.42	0.76	Tonga	Female	80-84	18.77	15.34	22.67
Tonga	Both	85-89	18.26	14.98	21.75	Tonga	Male	45-49 years	1.25	0.98	1.56	Tonga	Female	85-89	20.47	16.70	24.63
Tonga	Both	90-94	19.59	16.24	23.26	Tonga	Male	50-54 years	2.23	1.70	2.86	Tonga	Female	90-94	21.31	17.62	25.42
Tonga	Both	40-44 years	0.65	0.48	0.84	Tonga	Male	55-59 years	3.43	2.67	4.16	Tonga	Female	40-44 years	0.72	0.53	0.94
Tonga	Both	45-49 years	1.43	1.10	1.80	Tonga	Male	60-64 years	4.80	3.75	5.98	Tonga	Female	45-49 years	1.60	1.21	2.03

Tonga	Both	50-54 years	2.56	1.94	3.30	Tonga	Male	65-69 years	6.70	5.36	8.30	Tonga	Female	50-54 years	2.90	2.16	3.73
Tonga	Both	55-59 years	3.96	3.06	4.88	Tonga	Male	70-74 years	9.00	6.97	11.30	Tonga	Female	55-59 years	4.49	3.47	5.56
Tonga	Both	60-64 years	5.60	4.38	6.90	Tonga	Male	75-79 years	11.14	8.82	13.73	Tonga	Female	60-64 years	6.36	4.95	7.88
Tonga	Both	65-69 years	8.02	6.44	9.82	Tonga	Male	All ages	1.06	0.90	1.22	Tonga	Female	65-69 years	9.21	7.38	11.22
Tonga	Both	70-74 years	11.01	8.59	13.92	Tonga	Male	80-84	12.99	10.63	15.76	Tonga	Female	70-74 years	12.74	9.83	16.08
Tonga	Both	75-79 years	13.83	11.02	17.09	Tonga	Male	85-89	14.33	11.82	17.19	Tonga	Female	75-79 years	16.02	12.75	19.87
Tonga	Both	All ages	1.36	1.17	1.56	Tonga	Male	90-94	15.27	12.59	18.15	Tonga	Female	All ages	1.65	1.43	1.90
Trinidad and Tobago	Both	40-44 years	0.35	0.25	0.46	Trinidad and Tobago	Male	40-44 years	0.30	0.21	0.40	Trinidad and Tobago	Female	40-44 years	0.40	0.29	0.53
Trinidad and Tobago	Both	45-49 years	0.73	0.56	0.92	Trinidad and Tobago	Male	45-49 years	0.61	0.46	0.79	Trinidad and Tobago	Female	45-49 years	0.84	0.65	1.07
Trinidad and Tobago	Both	50-54 years	1.29	0.98	1.66	Trinidad and Tobago	Male	50-54 years	1.08	0.80	1.41	Trinidad and Tobago	Female	50-54 years	1.51	1.14	1.94
Trinidad and Tobago	Both	55-59 years	2.08	1.61	2.59	Trinidad and Tobago	Male	55-59 years	1.74	1.34	2.16	Trinidad and Tobago	Female	55-59 years	2.43	1.89	3.05
Trinidad and Tobago	Both	60-64 years	3.15	2.45	3.95	Trinidad and Tobago	Male	60-64 years	2.61	2.04	3.29	Trinidad and Tobago	Female	60-64 years	3.67	2.86	4.65
Trinidad and Tobago	Both	65-69 years	4.94	3.98	6.08	Trinidad and Tobago	Male	65-69 years	4.08	3.26	5.02	Trinidad and Tobago	Female	65-69 years	5.77	4.60	7.12
Trinidad and Tobago	Both	70-74 years	7.31	5.62	9.26	Trinidad and Tobago	Male	70-74 years	6.03	4.68	7.60	Trinidad and Tobago	Female	70-74 years	8.52	6.53	10.88
Trinidad and Tobago	Both	75-79 years	9.85	7.87	12.31	Trinidad and Tobago	Male	75-79 years	8.07	6.47	10.10	Trinidad and Tobago	Female	75-79 years	11.32	8.95	14.17
Trinidad and Tobago	Both	All ages	1.48	1.27	1.70	Trinidad and Tobago	Male	All ages	1.15	0.99	1.34	Trinidad and Tobago	Female	All ages	1.81	1.55	2.09
Trinidad and Tobago	Both	80-84	12.30	10.00	15.16	Trinidad and Tobago	Male	80-84	10.08	8.11	12.42	Trinidad and Tobago	Female	80-84	13.94	11.26	17.15
Trinidad and Tobago	Both	85-89	14.34	11.81	17.32	Trinidad and Tobago	Male	85-89	11.83	9.65	14.41	Trinidad and Tobago	Female	85-89	15.97	13.11	19.49
Trinidad and Tobago	Both	90-94	16.02	13.32	19.25	Trinidad and Tobago	Male	90-94	13.37	11.01	16.10	Trinidad and Tobago	Female	90-94	17.46	14.54	21.01

Tunisia	Both	40-44 years	0.41	0.29	0.55	Tunisia	Male	40-44 years	0.36	0.26	0.50	Tunisia	Female	40-44 years	0.46	0.33	0.62
Tunisia	Both	45-49 years	0.88	0.68	1.11	Tunisia	Male	45-49 years	0.76	0.59	0.96	Tunisia	Female	45-49 years	0.98	0.75	1.25
Tunisia	Both	50-54 years	1.56	1.19	1.98	Tunisia	Male	50-54 years	1.35	1.04	1.72	Tunisia	Female	50-54 years	1.76	1.32	2.28
Tunisia	Both	55-59 years	2.48	1.94	3.03	Tunisia	Male	55-59 years	2.14	1.69	2.62	Tunisia	Female	55-59 years	2.81	2.18	3.46
Tunisia	Both	60-64 years	3.64	2.89	4.48	Tunisia	Male	60-64 years	3.13	2.49	3.86	Tunisia	Female	60-64 years	4.16	3.27	5.14
Tunisia	Both	65-69 years	5.42	4.41	6.61	Tunisia	Male	65-69 years	4.63	3.75	5.68	Tunisia	Female	65-69 years	6.21	5.00	7.55
Tunisia	Both	70-74 years	7.69	5.98	9.59	Tunisia	Male	70-74 years	6.53	5.09	8.12	Tunisia	Female	70-74 years	8.80	6.77	11.07
Tunisia	Both	75-79 years	10.00	8.03	12.12	Tunisia	Male	75-79 years	8.51	6.86	10.38	Tunisia	Female	75-79 years	11.37	9.11	13.91
Tunisia	Both	All ages	1.29	1.12	1.47	Tunisia	Male	All ages	1.09	0.94	1.24	Tunisia	Female	All ages	1.48	1.27	1.70
Tunisia	Both	80-84	12.21	10.00	14.80	Tunisia	Male	80-84	10.44	8.50	12.82	Tunisia	Female	80-84	13.74	11.14	16.70
Tunisia	Both	85-89	14.05	11.59	16.80	Tunisia	Male	85-89	12.14	10.00	14.71	Tunisia	Female	85-89	15.57	12.77	18.79
Tunisia	Both	90-94	15.64	12.95	18.76	Tunisia	Male	90-94	13.63	11.19	16.36	Tunisia	Female	90-94	16.93	13.98	20.25
Turkey	Both	40-44 years	0.40	0.29	0.53	Turkey	Male	85-89	12.14	9.87	14.73	Turkey	Female	85-89	15.57	12.71	18.73
Turkey	Both	45-49 years	0.85	0.66	1.07	Turkey	Male	90-94	13.76	11.23	16.59	Turkey	Female	90-94	17.13	14.23	20.32
Turkey	Both	50-54 years	1.51	1.17	1.94	Turkey	Male	40-44 years	0.36	0.26	0.47	Turkey	Female	40-44 years	0.45	0.32	0.60
Turkey	Both	55-59 years	2.41	1.89	2.93	Turkey	Male	45-49 years	0.74	0.58	0.94	Turkey	Female	45-49 years	0.96	0.74	1.21
Turkey	Both	60-64 years	3.56	2.80	4.39	Turkey	Male	50-54 years	1.31	1.00	1.66	Turkey	Female	50-54 years	1.71	1.32	2.22
Turkey	Both	65-69 years	5.35	4.32	6.51	Turkey	Male	55-59 years	2.07	1.63	2.54	Turkey	Female	55-59 years	2.74	2.14	3.38
Turkey	Both	70-74 years	7.53	5.82	9.44	Turkey	Male	60-64 years	3.03	2.39	3.74	Turkey	Female	60-64 years	4.07	3.17	5.05
Turkey	Both	75-79 years	10.01	8.02	12.15	Turkey	Male	65-69 years	4.48	3.62	5.53	Turkey	Female	65-69 years	6.08	4.88	7.42
Turkey	Both	All ages	1.27	1.10	1.45	Turkey	Male	70-74 years	6.37	4.86	8.02	Turkey	Female	70-74 years	8.62	6.64	10.77
Turkey	Both	80-84	12.19	9.87	14.89	Turkey	Male	75-79 years	8.36	6.63	10.18	Turkey	Female	75-79 years	11.18	8.90	13.69
Turkey	Both	85-89	14.09	11.54	17.00	Turkey	Male	All ages	1.01	0.87	1.16	Turkey	Female	All ages	1.52	1.32	1.75
Turkey	Both	90-94	15.71	13.04	18.65	Turkey	Male	80-84	10.35	8.29	12.75	Turkey	Female	80-84	13.60	10.96	16.70

Turkmen istan	Both	65-69 years	5.48	4.42	6.66	Turkmen istan	Male	65-69 years	4.46	3.60	5.51	Turkmen istan	Female	65-69 years	6.24	5.01	7.62
Turkmen istan	Both	70-74 years	7.81	6.03	9.79	Turkmen istan	Male	70-74 years	6.30	4.88	7.89	Turkmen istan	Female	70-74 years	8.91	6.83	11.26
Turkmen istan	Both	75-79 years	10.22	8.16	12.51	Turkmen istan	Male	75-79 years	8.26	6.60	10.09	Turkmen istan	Female	75-79 years	11.60	9.24	14.24
Turkmen istan	Both	All ages	0.89	0.77	1.01	Turkmen istan	Male	All ages	0.63	0.54	0.72	Turkmen istan	Female	All ages	1.15	0.99	1.31
Turkmen istan	Both	80-84	12.64	10.29	15.33	Turkmen istan	Male	80-84	10.23	8.27	12.55	Turkmen istan	Female	80-84	14.13	11.45	17.30
Turkmen istan	Both	85-89	14.84	12.24	17.86	Turkmen istan	Male	85-89	11.99	9.80	14.55	Turkmen istan	Female	85-89	16.15	13.25	19.56
Turkmen istan	Both	90-94	16.78	13.92	20.01	Turkmen istan	Male	90-94	13.61	11.13	16.35	Turkmen istan	Female	90-94	17.69	14.62	21.21
Turkmen istan	Both	40-44 years	0.42	0.31	0.57	Turkmen istan	Male	40-44 years	0.37	0.27	0.51	Turkmen istan	Female	40-44 years	0.47	0.34	0.63
Turkmen istan	Both	45-49 years	0.88	0.68	1.10	Turkmen istan	Male	45-49 years	0.76	0.59	0.96	Turkmen istan	Female	45-49 years	1.00	0.77	1.26
Turkmen istan	Both	50-54 years	1.56	1.19	1.99	Turkmen istan	Male	50-54 years	1.32	1.01	1.68	Turkmen istan	Female	50-54 years	1.78	1.35	2.26
Turkmen istan	Both	55-59 years	2.48	1.93	3.02	Turkmen istan	Male	55-59 years	2.07	1.63	2.54	Turkmen istan	Female	55-59 years	2.83	2.19	3.48
Turkmen istan	Both	60-64 years	3.65	2.88	4.53	Turkmen istan	Male	60-64 years	3.03	2.40	3.75	Turkmen istan	Female	60-64 years	4.16	3.22	5.23
Tuvalu	Both	75-79 years	12.85	10.20	15.57	Tuvalu	Male	40-44 years	0.55	0.41	0.74	Tuvalu	Female	75-79 years	14.76	11.68	17.92
Tuvalu	Both	All ages	1.35	1.16	1.55	Tuvalu	Male	45-49 years	1.18	0.91	1.49	Tuvalu	Female	All ages	1.70	1.45	1.95
Tuvalu	Both	80-84	15.08	12.32	18.36	Tuvalu	Male	50-54 years	2.08	1.57	2.68	Tuvalu	Female	80-84	17.39	14.21	21.32
Tuvalu	Both	85-89	16.52	13.63	19.81	Tuvalu	Male	55-59 years	3.18	2.49	3.90	Tuvalu	Female	85-89	19.05	15.64	22.87
Tuvalu	Both	90-94	17.29	14.42	20.63	Tuvalu	Male	60-64 years	4.43	3.52	5.50	Tuvalu	Female	90-94	19.91	16.54	23.43
Tuvalu	Both	40-44 years	0.61	0.45	0.80	Tuvalu	Male	65-69 years	6.17	4.96	7.55	Tuvalu	Female	40-44 years	0.67	0.50	0.89
Tuvalu	Both	45-49 years	1.33	1.03	1.66	Tuvalu	Male	70-74 years	8.29	6.46	10.38	Tuvalu	Female	45-49 years	1.49	1.15	1.87
Tuvalu	Both	50-54 years	2.38	1.81	3.03	Tuvalu	Male	75-79 years	10.30	8.25	12.49	Tuvalu	Female	50-54 years	2.68	2.04	3.44
Tuvalu	Both	55-59 years	3.67	2.86	4.48	Tuvalu	Male	All ages	1.02	0.88	1.18	Tuvalu	Female	55-59 years	4.13	3.22	5.06
Tuvalu	Both	60-64 years	5.17	4.06	6.38	Tuvalu	Male	80-84	12.07	9.82	14.72	Tuvalu	Female	60-64 years	5.82	4.52	7.26
Tuvalu	Both	65-69 years	7.40	5.95	9.02	Tuvalu	Male	85-89	13.35	10.98	16.20	Tuvalu	Female	65-69 years	8.42	6.69	10.33
Tuvalu	Both	70-74 years	10.22	7.95	12.80	Tuvalu	Male	90-94	14.24	11.84	17.16	Tuvalu	Female	70-74 years	11.68	8.99	14.79

Uganda	Both	40-44 years	0.30	0.21	0.39	Uganda	Male	40-44 years	0.26	0.19	0.36	Uganda	Female	40-44 years	0.33	0.24	0.45
Uganda	Both	45-49 years	0.65	0.50	0.82	Uganda	Male	45-49 years	0.57	0.43	0.73	Uganda	Female	45-49 years	0.72	0.55	0.92
Uganda	Both	50-54 years	1.18	0.90	1.52	Uganda	Male	50-54 years	1.03	0.77	1.33	Uganda	Female	50-54 years	1.31	1.00	1.72
Uganda	Both	55-59 years	1.90	1.50	2.33	Uganda	Male	55-59 years	1.65	1.29	2.00	Uganda	Female	55-59 years	2.13	1.67	2.61
Uganda	Both	60-64 years	2.83	2.22	3.52	Uganda	Male	60-64 years	2.42	1.90	3.02	Uganda	Female	60-64 years	3.18	2.50	3.94
Uganda	Both	65-69 years	4.28	3.43	5.25	Uganda	Male	65-69 years	3.59	2.89	4.43	Uganda	Female	65-69 years	4.82	3.85	5.96
Uganda	Both	70-74 years	6.18	4.81	7.80	Uganda	Male	70-74 years	5.09	3.84	6.39	Uganda	Female	70-74 years	6.96	5.39	8.91
Uganda	Both	75-79 years	8.19	6.61	10.06	Uganda	Male	75-79 years	6.69	5.40	8.19	Uganda	Female	75-79 years	9.21	7.36	11.51
Uganda	Both	All ages	0.29	0.25	0.33	Uganda	Male	All ages	0.22	0.18	0.25	Uganda	Female	All ages	0.36	0.31	0.41
Uganda	Both	80-84	10.22	8.26	12.69	Uganda	Male	80-84	8.31	6.73	10.31	Uganda	Female	80-84	11.37	9.14	14.10
Uganda	Both	85-89	12.06	9.77	14.69	Uganda	Male	85-89	9.77	7.93	11.95	Uganda	Female	85-89	13.11	10.56	15.93
Uganda	Both	90-94	13.66	11.26	16.50	Uganda	Male	90-94	11.12	9.04	13.54	Uganda	Female	90-94	14.45	11.93	17.40
Ukraine	Both	40-44 years	0.50	0.37	0.64	Ukraine	Male	40-44 years	0.28	0.21	0.39	Ukraine	Female	40-44 years	0.69	0.52	0.90
Ukraine	Both	45-49 years	1.14	0.88	1.44	Ukraine	Male	45-49 years	0.60	0.47	0.75	Ukraine	Female	45-49 years	1.61	1.24	2.07
Ukraine	Both	50-54 years	2.12	1.60	2.76	Ukraine	Male	50-54 years	1.06	0.81	1.35	Ukraine	Female	50-54 years	3.00	2.24	3.93
Ukraine	Both	55-59 years	3.45	2.70	4.25	Ukraine	Male	55-59 years	1.67	1.31	2.05	Ukraine	Female	55-59 years	4.83	3.77	5.95
Ukraine	Both	60-64 years	5.18	4.03	6.41	Ukraine	Male	60-64 years	2.42	1.92	3.05	Ukraine	Female	60-64 years	7.12	5.51	8.79
Ukraine	Both	65-69 years	7.97	6.43	9.71	Ukraine	Male	65-69 years	3.53	2.86	4.36	Ukraine	Female	65-69 years	10.75	8.64	13.12
Ukraine	Both	70-74 years	11.60	8.99	14.36	Ukraine	Male	70-74 years	4.95	3.92	6.18	Ukraine	Female	70-74 years	15.27	11.81	19.05
Ukraine	Both	75-79 years	15.37	12.33	18.71	Ukraine	Male	75-79 years	6.46	5.18	7.85	Ukraine	Female	75-79 years	19.41	15.53	23.59
Ukraine	Both	All ages	3.12	2.69	3.59	Ukraine	Male	All ages	1.12	0.96	1.28	Ukraine	Female	All ages	4.78	4.09	5.51
Ukraine	Both	80-84	18.45	15.11	22.17	Ukraine	Male	80-84	8.02	6.48	9.82	Ukraine	Female	80-84	22.83	18.58	27.44
Ukraine	Both	85-89	20.76	17.34	24.83	Ukraine	Male	85-89	9.54	7.76	11.51	Ukraine	Female	85-89	24.95	20.70	29.86
Ukraine	Both	90-94	22.77	19.13	26.97	Ukraine	Male	90-94	11.12	9.05	13.33	Ukraine	Female	90-94	25.95	21.79	30.78

United Arab Emirates	Both	70-74 years	6.99	5.43	8.75	United Arab Emirates	Male	40-44 years	0.35	0.25	0.47	United Arab Emirates	Female	40-44 years	0.44	0.32	0.58
United Arab Emirates	Both	75-79 years	9.40	7.50	11.50	United Arab Emirates	Male	45-49 years	0.73	0.56	0.93	United Arab Emirates	Female	45-49 years	0.93	0.73	1.19
United Arab Emirates	Both	All ages	0.43	0.35	0.50	United Arab Emirates	Male	50-54 years	1.29	0.99	1.66	United Arab Emirates	Female	50-54 years	1.66	1.27	2.13
United Arab Emirates	Both	80-84	11.62	9.49	14.19	United Arab Emirates	Male	55-59 years	2.04	1.60	2.51	United Arab Emirates	Female	55-59 years	2.64	2.05	3.23
United Arab Emirates	Both	85-89	13.43	11.10	16.14	United Arab Emirates	Male	60-64 years	2.99	2.36	3.74	United Arab Emirates	Female	60-64 years	3.90	3.03	4.87
United Arab Emirates	Both	90-94	15.16	12.59	17.95	United Arab Emirates	Male	65-69 years	4.42	3.59	5.46	United Arab Emirates	Female	65-69 years	5.83	4.66	7.18
United Arab Emirates	Both	40-44 years	0.36	0.26	0.49	United Arab Emirates	Male	70-74 years	6.29	4.92	7.90	United Arab Emirates	Female	70-74 years	8.34	6.45	10.46
United Arab Emirates	Both	45-49 years	0.77	0.60	0.98	United Arab Emirates	Male	75-79 years	8.34	6.59	10.22	United Arab Emirates	Female	75-79 years	11.02	8.77	13.56
United Arab Emirates	Both	50-54 years	1.37	1.04	1.75	United Arab Emirates	Male	All ages	0.42	0.35	0.50	United Arab Emirates	Female	All ages	0.43	0.36	0.51
United Arab Emirates	Both	55-59 years	2.19	1.71	2.68	United Arab Emirates	Male	80-84	10.48	8.41	12.86	United Arab Emirates	Female	80-84	13.71	11.09	16.57
United Arab Emirates	Both	60-64 years	3.22	2.54	4.01	United Arab Emirates	Male	85-89	12.53	10.34	15.12	United Arab Emirates	Female	85-89	16.03	13.27	19.32
United Arab Emirates	Both	65-69 years	4.79	3.89	5.90	United Arab Emirates	Male	90-94	14.49	11.93	17.24	United Arab Emirates	Female	90-94	17.97	14.98	21.29
United Kingdom	Both	40-44 years	0.82	0.62	1.08	United Kingdom	Male	40-44 years	0.44	0.32	0.58	United Kingdom	Female	40-44 years	1.20	0.91	1.55
United Kingdom	Both	45-49 years	1.52	1.19	1.87	United Kingdom	Male	45-49 years	0.96	0.74	1.21	United Kingdom	Female	45-49 years	2.06	1.64	2.53
United Kingdom	Both	50-54 years	2.39	1.86	2.99	United Kingdom	Male	50-54 years	1.75	1.35	2.23	United Kingdom	Female	50-54 years	3.01	2.34	3.77
United Kingdom	Both	55-59 years	3.57	2.81	4.30	United Kingdom	Male	55-59 years	2.94	2.32	3.56	United Kingdom	Female	55-59 years	4.17	3.31	5.03
United Kingdom	Both	60-64 years	5.16	4.13	6.30	United Kingdom	Male	60-64 years	4.50	3.56	5.58	United Kingdom	Female	60-64 years	5.79	4.65	7.06
United Kingdom	Both	65-69 years	7.83	6.41	9.46	United Kingdom	Male	65-69 years	6.75	5.50	8.23	United Kingdom	Female	65-69 years	8.85	7.26	10.63
United Kingdom	Both	70-74 years	11.27	8.89	13.93	United Kingdom	Male	70-74 years	9.45	7.36	11.75	United Kingdom	Female	70-74 years	12.94	10.26	16.01

United Kingdom	Both	75-79 years	14.74	11.87	17.84	United Kingdom	Male	75-79 years	12.06	9.66	14.58	United Kingdom	Female	75-79 years	17.05	13.78	20.65
United Kingdom	Both	All ages	3.42	2.96	3.88	United Kingdom	Male	All ages	2.61	2.25	2.98	United Kingdom	Female	All ages	4.17	3.60	4.74
United Kingdom	Both	80-84	18.03	14.90	21.71	United Kingdom	Male	80-84	14.39	11.83	17.43	United Kingdom	Female	80-84	20.87	17.21	25.09
United Kingdom	Both	85-89	20.72	17.25	24.68	United Kingdom	Male	85-89	16.12	13.37	19.28	United Kingdom	Female	85-89	23.71	19.71	28.34
United Kingdom	Both	90-94	22.81	19.17	26.94	United Kingdom	Male	90-94	17.33	14.49	20.54	United Kingdom	Female	90-94	25.57	21.52	30.11
United Republic of Tanzania	Both	60-64 years	3.09	2.43	3.86	United Republic of Tanzania	Male	60-64 years	2.67	2.09	3.35	United Republic of Tanzania	Female	40-44 years	0.35	0.25	0.49
United Republic of Tanzania	Both	65-69 years	4.65	3.72	5.75	United Republic of Tanzania	Male	65-69 years	3.96	3.17	4.89	United Republic of Tanzania	Female	45-49 years	0.79	0.60	1.00
United Republic of Tanzania	Both	70-74 years	6.71	5.18	8.46	United Republic of Tanzania	Male	70-74 years	5.63	4.29	7.18	United Republic of Tanzania	Female	50-54 years	1.45	1.08	1.91
United Republic of Tanzania	Both	75-79 years	8.94	7.15	11.00	United Republic of Tanzania	Male	75-79 years	7.43	5.93	9.16	United Republic of Tanzania	Female	55-59 years	2.35	1.82	2.92
United Republic of Tanzania	Both	All ages	0.41	0.35	0.47	United Republic of Tanzania	Male	All ages	0.34	0.29	0.40	United Republic of Tanzania	Female	60-64 years	3.51	2.75	4.43
United Republic of Tanzania	Both	80-84	11.16	9.03	13.80	United Republic of Tanzania	Male	80-84	9.24	7.48	11.34	United Republic of Tanzania	Female	65-69 years	5.33	4.26	6.62
United Republic of Tanzania	Both	85-89	13.03	10.64	15.83	United Republic of Tanzania	Male	85-89	10.83	8.84	13.08	United Republic of Tanzania	Female	70-74 years	7.70	5.93	9.82
United Republic of Tanzania	Both	90-94	14.64	12.04	17.59	United Republic of Tanzania	Male	90-94	12.23	9.96	14.76	United Republic of Tanzania	Female	75-79 years	10.18	8.11	12.60
United Republic of Tanzania	Both	40-44 years	0.32	0.22	0.44	United Republic of Tanzania	Male	40-44 years	0.28	0.20	0.38	United Republic of Tanzania	Female	All ages	0.48	0.41	0.55
United Republic of Tanzania	Both	45-49 years	0.71	0.54	0.90	United Republic of Tanzania	Male	45-49 years	0.62	0.47	0.78	United Republic of Tanzania	Female	80-84	12.55	10.11	15.55
United Republic	Both	50-54 years	1.29	0.96	1.68	United Republic	Male	50-54 years	1.12	0.84	1.46	United Republic	Female	85-89	14.43	11.76	17.66

of Tanzania						of Tanzania						of Tanzania					
United Republic of Tanzania	Both	55-59 years	2.08	1.62	2.56	United Republic of Tanzania	Male	55-59 years	1.81	1.41	2.25	United Republic of Tanzania	Female	90-94	15.85	12.96	19.00
United States of America	Both	40-44 years	1.17	0.95	1.43	United States of America	Male	40-44 years	0.72	0.58	0.90	United States of America	Female	40-44 years	1.61	1.29	1.94
United States of America	Both	45-49 years	1.96	1.67	2.28	United States of America	Male	45-49 years	1.41	1.17	1.66	United States of America	Female	45-49 years	2.50	2.11	2.91
United States of America	Both	50-54 years	2.86	2.41	3.38	United States of America	Male	50-54 years	2.41	1.98	2.91	United States of America	Female	50-54 years	3.28	2.76	3.83
United States of America	Both	55-59 years	4.19	3.59	4.81	United States of America	Male	55-59 years	4.09	3.48	4.72	United States of America	Female	55-59 years	4.29	3.65	4.94
United States of America	Both	60-64 years	6.23	5.27	7.23	United States of America	Male	60-64 years	6.53	5.45	7.69	United States of America	Female	60-64 years	5.95	5.08	6.85
United States of America	Both	65-69 years	9.99	8.49	11.50	United States of America	Male	65-69 years	10.37	8.83	11.99	United States of America	Female	65-69 years	9.64	8.25	11.10
United States of America	Both	70-74 years	14.92	12.50	17.62	United States of America	Male	70-74 years	15.09	12.61	17.87	United States of America	Female	70-74 years	14.77	12.33	17.51
United States of America	Both	75-79 years	19.62	16.73	22.68	United States of America	Male	75-79 years	19.32	16.39	22.44	United States of America	Female	75-79 years	19.86	17.05	22.96
United States of America	Both	All ages	4.01	3.61	4.41	United States of America	Male	All ages	3.59	3.22	3.99	United States of America	Female	All ages	4.40	3.97	4.83
United States of America	Both	80-84	23.91	20.73	27.60	United States of America	Male	80-84	22.91	19.90	26.31	United States of America	Female	80-84	24.65	21.31	28.58
United States of America	Both	85-89	27.49	24.07	31.54	United States of America	Male	85-89	25.69	22.52	29.43	United States of America	Female	85-89	28.61	25.06	32.80
United States of America	Both	90-94	30.17	26.60	34.14	United States of America	Male	90-94	27.59	24.30	31.16	United States of America	Female	90-94	31.45	27.72	35.66
United States Virgin Islands	Both	40-44 years	0.30	0.22	0.41	United States Virgin Islands	Male	40-44 years	0.26	0.18	0.36	United States Virgin Islands	Female	40-44 years	0.34	0.24	0.48
United States Virgin Islands	Both	45-49 years	0.64	0.49	0.80	United States Virgin Islands	Male	45-49 years	0.53	0.41	0.68	United States Virgin Islands	Female	45-49 years	0.72	0.56	0.90
United States	Both	50-54 years	1.12	0.85	1.43	United States	Male	50-54 years	0.94	0.71	1.20	United States	Female	50-54 years	1.29	0.98	1.64

Virgin Islands						Virgin Islands						Virgin Islands					
United States Virgin Islands	Both	55-59 years	1.82	1.43	2.20	United States Virgin Islands	Male	55-59 years	1.50	1.18	1.82	United States Virgin Islands	Female	55-59 years	2.09	1.64	2.55
United States Virgin Islands	Both	60-64 years	2.76	2.19	3.40	United States Virgin Islands	Male	60-64 years	2.26	1.80	2.84	United States Virgin Islands	Female	60-64 years	3.18	2.52	3.93
United States Virgin Islands	Both	65-69 years	4.28	3.45	5.23	United States Virgin Islands	Male	65-69 years	3.50	2.80	4.30	United States Virgin Islands	Female	65-69 years	4.94	3.98	6.03
United States Virgin Islands	Both	70-74 years	6.29	4.85	7.81	United States Virgin Islands	Male	70-74 years	5.15	3.94	6.48	United States Virgin Islands	Female	70-74 years	7.23	5.53	9.03
United States Virgin Islands	Both	75-79 years	8.49	6.73	10.35	United States Virgin Islands	Male	75-79 years	6.92	5.37	8.50	United States Virgin Islands	Female	75-79 years	9.66	7.70	11.77
United States Virgin Islands	Both	All ages	1.75	1.48	2.02	United States Virgin Islands	Male	All ages	1.33	1.12	1.54	United States Virgin Islands	Female	All ages	2.12	1.80	2.44
United States Virgin Islands	Both	80-84	10.75	8.73	13.12	United States Virgin Islands	Male	80-84	8.71	6.93	10.79	United States Virgin Islands	Female	80-84	12.05	9.89	14.67
United States Virgin Islands	Both	85-89	12.84	10.65	15.45	United States Virgin Islands	Male	85-89	10.40	8.45	12.61	United States Virgin Islands	Female	85-89	14.09	11.68	17.11
United States Virgin Islands	Both	90-94	14.72	12.21	17.50	United States Virgin Islands	Male	90-94	12.04	9.85	14.38	United States Virgin Islands	Female	90-94	15.80	13.14	18.84
Uruguay	Both	40-44 years	0.84	0.63	1.11	Uruguay	Male	85-89	15.88	13.16	19.05	Uruguay	Female	40-44 years	1.22	0.91	1.59
Uruguay	Both	45-49 years	1.50	1.17	1.86	Uruguay	Male	90-94	16.95	14.10	20.04	Uruguay	Female	45-49 years	2.02	1.57	2.52
Uruguay	Both	50-54 years	2.30	1.79	2.92	Uruguay	Male	40-44 years	0.43	0.31	0.59	Uruguay	Female	50-54 years	2.86	2.21	3.66
Uruguay	Both	55-59 years	3.41	2.71	4.15	Uruguay	Male	45-49 years	0.93	0.71	1.19	Uruguay	Female	55-59 years	3.92	3.08	4.86
Uruguay	Both	60-64 years	4.96	3.95	6.15	Uruguay	Male	50-54 years	1.68	1.27	2.14	Uruguay	Female	60-64 years	5.49	4.35	6.82
Uruguay	Both	65-69 years	7.75	6.20	9.41	Uruguay	Male	55-59 years	2.83	2.21	3.45	Uruguay	Female	65-69 years	8.69	7.02	10.60
Uruguay	Both	70-74 years	11.49	8.93	14.33	Uruguay	Male	60-64 years	4.34	3.40	5.45	Uruguay	Female	70-74 years	13.05	10.05	16.23

Uruguay	Both	75-79 years	15.17	12.22	18.41	Uruguay	Male	65-69 years	6.61	5.27	8.12	Uruguay	Female	75-79 years	17.31	13.81	20.98
Uruguay	Both	All ages	2.94	2.54	3.36	Uruguay	Male	70-74 years	9.42	7.23	11.89	Uruguay	Female	All ages	3.76	3.22	4.27
Uruguay	Both	80-84	18.64	15.26	22.44	Uruguay	Male	75-79 years	12.01	9.48	14.79	Uruguay	Female	80-84	21.12	17.15	25.56
Uruguay	Both	85-89	21.31	17.62	25.40	Uruguay	Male	All ages	2.02	1.73	2.34	Uruguay	Female	85-89	23.79	19.54	28.45
Uruguay	Both	90-94	22.95	19.23	27.02	Uruguay	Male	80-84	14.24	11.63	17.27	Uruguay	Female	90-94	25.32	21.16	29.90
Uzbekistan	Both	40-44 years	0.40	0.29	0.53	Uzbekistan	Male	40-44 years	0.36	0.26	0.48	Uzbekistan	Female	40-44 years	0.45	0.32	0.60
Uzbekistan	Both	45-49 years	0.85	0.66	1.07	Uzbekistan	Male	45-49 years	0.74	0.57	0.93	Uzbekistan	Female	45-49 years	0.96	0.75	1.22
Uzbekistan	Both	50-54 years	1.49	1.14	1.92	Uzbekistan	Male	50-54 years	1.27	0.96	1.62	Uzbekistan	Female	50-54 years	1.71	1.29	2.20
Uzbekistan	Both	55-59 years	2.35	1.84	2.87	Uzbekistan	Male	55-59 years	1.98	1.56	2.41	Uzbekistan	Female	55-59 years	2.69	2.06	3.31
Uzbekistan	Both	60-64 years	3.43	2.70	4.30	Uzbekistan	Male	60-64 years	2.86	2.27	3.59	Uzbekistan	Female	60-64 years	3.91	3.05	4.94
Uzbekistan	Both	65-69 years	5.14	4.13	6.31	Uzbekistan	Male	65-69 years	4.21	3.38	5.17	Uzbekistan	Female	65-69 years	5.87	4.67	7.26
Uzbekistan	Both	70-74 years	7.37	5.63	9.29	Uzbekistan	Male	70-74 years	5.95	4.62	7.47	Uzbekistan	Female	70-74 years	8.38	6.37	10.69
Uzbekistan	Both	75-79 years	9.69	7.71	11.84	Uzbekistan	Male	75-79 years	7.75	6.24	9.44	Uzbekistan	Female	75-79 years	10.89	8.60	13.49
Uzbekistan	Both	All ages	0.63	0.53	0.73	Uzbekistan	Male	All ages	0.47	0.40	0.55	Uzbekistan	Female	All ages	0.77	0.65	0.90
Uzbekistan	Both	80-84	12.04	9.76	14.70	Uzbekistan	Male	80-84	9.53	7.78	11.68	Uzbekistan	Female	80-84	13.20	10.57	16.21
Uzbekistan	Both	85-89	13.49	11.02	16.22	Uzbekistan	Male	85-89	11.11	9.16	13.43	Uzbekistan	Female	85-89	15.01	12.11	18.11
Uzbekistan	Both	90-94	14.67	12.10	17.55	Uzbekistan	Male	90-94	12.58	10.35	15.05	Uzbekistan	Female	90-94	16.34	13.44	19.76
Vanuatu	Both	40-44 years	0.61	0.46	0.81	Vanuatu	Male	40-44 years	0.55	0.41	0.73	Vanuatu	Female	40-44 years	0.68	0.51	0.90
Vanuatu	Both	45-49 years	1.34	1.04	1.68	Vanuatu	Male	45-49 years	1.19	0.92	1.50	Vanuatu	Female	45-49 years	1.50	1.17	1.90
Vanuatu	Both	50-54 years	2.40	1.83	3.05	Vanuatu	Male	50-54 years	2.10	1.61	2.70	Vanuatu	Female	50-54 years	2.72	2.08	3.50
Vanuatu	Both	55-59 years	3.68	2.87	4.53	Vanuatu	Male	55-59 years	3.22	2.51	3.95	Vanuatu	Female	55-59 years	4.19	3.25	5.18
Vanuatu	Both	60-64 years	5.15	4.05	6.37	Vanuatu	Male	60-64 years	4.48	3.54	5.58	Vanuatu	Female	60-64 years	5.91	4.59	7.37
Vanuatu	Both	65-69 years	7.32	5.86	8.92	Vanuatu	Male	65-69 years	6.24	5.04	7.62	Vanuatu	Female	65-69 years	8.53	6.78	10.41
Vanuatu	Both	70-74 years	10.01	7.85	12.61	Vanuatu	Male	70-74 years	8.38	6.58	10.61	Vanuatu	Female	70-74 years	11.83	9.22	14.99

Vanuatu	Both	75-79 years	12.53	10.13	15.37	Vanuatu	Male	75-79 years	10.41	8.35	12.79	Vanuatu	Female	75-79 years	14.93	12.06	18.25
Vanuatu	Both	All ages	0.88	0.75	1.00	Vanuatu	Male	All ages	0.78	0.67	0.90	Vanuatu	Female	All ages	0.97	0.82	1.10
Vanuatu	Both	80-84	14.69	12.14	17.81	Vanuatu	Male	80-84	12.19	10.01	14.87	Vanuatu	Female	80-84	17.55	14.45	21.44
Vanuatu	Both	85-89	16.20	13.34	19.65	Vanuatu	Male	85-89	13.46	11.03	16.40	Vanuatu	Female	85-89	19.17	15.69	23.23
Vanuatu	Both	90-94	17.09	14.16	20.46	Vanuatu	Male	90-94	14.31	11.72	17.30	Vanuatu	Female	90-94	19.95	16.41	23.94
Venezuela (Bolivarian Republic of)	Both	40-44 years	0.33	0.23	0.43	Venezuela (Bolivarian Republic of)	Male	40-44 years	0.28	0.20	0.37	Venezuela (Bolivarian Republic of)	Female	40-44 years	0.37	0.26	0.50
Venezuela (Bolivarian Republic of)	Both	45-49 years	0.68	0.52	0.85	Venezuela (Bolivarian Republic of)	Male	45-49 years	0.57	0.44	0.72	Venezuela (Bolivarian Republic of)	Female	45-49 years	0.78	0.60	0.99
Venezuela (Bolivarian Republic of)	Both	50-54 years	1.19	0.92	1.52	Venezuela (Bolivarian Republic of)	Male	50-54 years	0.99	0.76	1.27	Venezuela (Bolivarian Republic of)	Female	50-54 years	1.39	1.05	1.77
Venezuela (Bolivarian Republic of)	Both	55-59 years	1.93	1.51	2.35	Venezuela (Bolivarian Republic of)	Male	55-59 years	1.59	1.24	1.96	Venezuela (Bolivarian Republic of)	Female	55-59 years	2.25	1.73	2.76
Venezuela (Bolivarian Republic of)	Both	60-64 years	2.95	2.31	3.67	Venezuela (Bolivarian Republic of)	Male	60-64 years	2.42	1.91	3.03	Venezuela (Bolivarian Republic of)	Female	60-64 years	3.44	2.68	4.36
Venezuela (Bolivarian Republic of)	Both	65-69 years	4.75	3.77	5.86	Venezuela (Bolivarian Republic of)	Male	65-69 years	3.89	3.08	4.79	Venezuela (Bolivarian Republic of)	Female	65-69 years	5.52	4.35	6.83
Venezuela (Bolivarian Republic of)	Both	70-74 years	7.13	5.51	9.01	Venezuela (Bolivarian Republic of)	Male	70-74 years	5.85	4.47	7.37	Venezuela (Bolivarian Republic of)	Female	70-74 years	8.26	6.30	10.55

Venezuela (Bolivarian Republic of)	Both	75-79 years	9.55	7.61	11.65	Venezuela (Bolivarian Republic of)	Male	75-79 years	7.83	6.16	9.60	Venezuela (Bolivarian Republic of)	Female	75-79 years	10.99	8.71	13.58
Venezuela (Bolivarian Republic of)	Both	All ages	1.08	0.92	1.24	Venezuela (Bolivarian Republic of)	Male	All ages	0.84	0.71	0.97	Venezuela (Bolivarian Republic of)	Female	All ages	1.31	1.12	1.50
Venezuela (Bolivarian Republic of)	Both	80-84	11.83	9.55	14.38	Venezuela (Bolivarian Republic of)	Male	80-84	9.66	7.78	11.87	Venezuela (Bolivarian Republic of)	Female	80-84	13.45	10.82	16.46
Venezuela (Bolivarian Republic of)	Both	85-89	13.56	11.14	16.27	Venezuela (Bolivarian Republic of)	Male	85-89	11.12	9.01	13.38	Venezuela (Bolivarian Republic of)	Female	85-89	15.17	12.49	18.25
Venezuela (Bolivarian Republic of)	Both	90-94	14.81	12.24	17.71	Venezuela (Bolivarian Republic of)	Male	90-94	12.32	10.11	14.72	Venezuela (Bolivarian Republic of)	Female	90-94	16.27	13.44	19.38
Viet Nam	Both	40-44 years	0.56	0.41	0.73	Viet Nam	Male	40-44 years	0.49	0.36	0.65	Viet Nam	Female	40-44 years	0.62	0.46	0.84
Viet Nam	Both	45-49 years	1.22	0.95	1.55	Viet Nam	Male	45-49 years	1.04	0.81	1.33	Viet Nam	Female	45-49 years	1.40	1.09	1.78
Viet Nam	Both	50-54 years	2.21	1.68	2.81	Viet Nam	Male	50-54 years	1.84	1.40	2.37	Viet Nam	Female	50-54 years	2.55	1.93	3.28
Viet Nam	Both	55-59 years	3.44	2.70	4.22	Viet Nam	Male	55-59 years	2.82	2.21	3.49	Viet Nam	Female	55-59 years	4.00	3.11	4.94
Viet Nam	Both	60-64 years	4.90	3.84	6.04	Viet Nam	Male	60-64 years	3.92	3.08	4.89	Viet Nam	Female	60-64 years	5.71	4.43	7.02
Viet Nam	Both	65-69 years	7.01	5.64	8.51	Viet Nam	Male	65-69 years	5.45	4.39	6.74	Viet Nam	Female	65-69 years	8.20	6.58	10.00
Viet Nam	Both	70-74 years	9.62	7.54	12.05	Viet Nam	Male	70-74 years	7.31	5.74	9.05	Viet Nam	Female	70-74 years	11.22	8.77	14.23
Viet Nam	Both	75-79 years	12.26	9.78	15.08	Viet Nam	Male	75-79 years	9.11	7.34	10.99	Viet Nam	Female	75-79 years	14.09	11.22	17.51
Viet Nam	Both	All ages	1.42	1.22	1.63	Viet Nam	Male	All ages	0.97	0.84	1.12	Viet Nam	Female	All ages	1.86	1.59	2.13
Viet Nam	Both	80-84	14.65	11.87	17.76	Viet Nam	Male	80-84	10.74	8.74	13.09	Viet Nam	Female	80-84	16.57	13.43	20.14

Viet Nam	Both	85-89	16.34	13.44	19.68	Viet Nam	Male	85-89	12.00	9.79	14.51	Viet Nam	Female	85-89	18.21	14.85	22.03
Viet Nam	Both	90-94	17.67	14.59	21.09	Viet Nam	Male	90-94	12.97	10.69	15.61	Viet Nam	Female	90-94	19.14	15.83	22.88
Yemen	Both	40-44 years	0.38	0.27	0.52	Yemen	Male	40-44 years	0.33	0.24	0.46	Yemen	Female	40-44 years	0.42	0.30	0.57
Yemen	Both	45-49 years	0.79	0.61	1.00	Yemen	Male	45-49 years	0.70	0.54	0.88	Yemen	Female	45-49 years	0.89	0.69	1.12
Yemen	Both	50-54 years	1.40	1.07	1.77	Yemen	Male	50-54 years	1.22	0.93	1.55	Yemen	Female	50-54 years	1.57	1.19	2.01
Yemen	Both	55-59 years	2.19	1.71	2.67	Yemen	Male	55-59 years	1.91	1.50	2.35	Yemen	Female	55-59 years	2.46	1.92	3.02
Yemen	Both	60-64 years	3.17	2.51	3.95	Yemen	Male	60-64 years	2.75	2.17	3.46	Yemen	Female	60-64 years	3.58	2.77	4.48
Yemen	Both	65-69 years	4.71	3.76	5.79	Yemen	Male	65-69 years	4.08	3.23	5.06	Yemen	Female	65-69 years	5.34	4.24	6.63
Yemen	Both	70-74 years	6.71	5.12	8.46	Yemen	Male	70-74 years	5.78	4.42	7.28	Yemen	Female	70-74 years	7.63	5.79	9.68
Yemen	Both	75-79 years	8.76	7.03	10.65	Yemen	Male	75-79 years	7.55	6.09	9.27	Yemen	Female	75-79 years	9.94	7.93	12.19
Yemen	Both	All ages	0.40	0.34	0.46	Yemen	Male	All ages	0.35	0.29	0.40	Yemen	Female	All ages	0.46	0.39	0.53
Yemen	Both	80-84	10.70	8.66	13.08	Yemen	Male	80-84	9.26	7.42	11.38	Yemen	Female	80-84	12.08	9.80	14.76
Yemen	Both	85-89	12.32	10.04	14.78	Yemen	Male	85-89	10.72	8.72	13.05	Yemen	Female	85-89	13.68	11.13	16.45
Yemen	Both	90-94	13.64	11.19	16.37	Yemen	Male	90-94	11.99	9.83	14.69	Yemen	Female	90-94	14.81	12.16	17.85
Zambia	Both	40-44 years	0.31	0.22	0.41	Zambia	Male	40-44 years	0.27	0.20	0.37	Zambia	Female	40-44 years	0.34	0.25	0.46
Zambia	Both	45-49 years	0.67	0.52	0.85	Zambia	Male	45-49 years	0.60	0.46	0.76	Zambia	Female	45-49 years	0.76	0.58	0.97
Zambia	Both	50-54 years	1.23	0.92	1.58	Zambia	Male	50-54 years	1.08	0.81	1.41	Zambia	Female	50-54 years	1.38	1.03	1.78
Zambia	Both	55-59 years	1.99	1.56	2.46	Zambia	Male	55-59 years	1.74	1.35	2.15	Zambia	Female	55-59 years	2.25	1.76	2.79
Zambia	Both	60-64 years	2.97	2.33	3.71	Zambia	Male	60-64 years	2.55	2.00	3.20	Zambia	Female	60-64 years	3.36	2.62	4.20
Zambia	Both	65-69 years	4.48	3.58	5.51	Zambia	Male	65-69 years	3.78	3.03	4.69	Zambia	Female	65-69 years	5.09	4.06	6.23
Zambia	Both	70-74 years	6.45	4.92	8.10	Zambia	Male	70-74 years	5.36	4.13	6.76	Zambia	Female	70-74 years	7.34	5.56	9.31
Zambia	Both	75-79 years	8.55	6.79	10.50	Zambia	Male	75-79 years	7.10	5.66	8.68	Zambia	Female	75-79 years	9.72	7.66	11.98
Zambia	Both	All ages	0.32	0.27	0.37	Zambia	Male	All ages	0.26	0.23	0.30	Zambia	Female	All ages	0.37	0.32	0.43
Zambia	Both	80-84	10.68	8.58	13.02	Zambia	Male	80-84	8.88	7.07	10.93	Zambia	Female	80-84	12.06	9.71	14.77

Zambia	Both	85-89	12.52	10.21	15.03	Zambia	Male	85-89	10.51	8.57	12.73	Zambia	Female	85-89	13.96	11.34	16.84
Zambia	Both	90-94	14.13	11.55	16.83	Zambia	Male	90-94	12.00	9.81	14.48	Zambia	Female	90-94	15.45	12.64	18.48
Zimbabw e	Both	40-44 years	0.39	0.28	0.53	Zimbabw e	Male	40-44 years	0.33	0.24	0.46	Zimbabw e	Female	40-44 years	0.45	0.33	0.61
Zimbabw e	Both	45-49 years	0.87	0.67	1.10	Zimbabw e	Male	45-49 years	0.73	0.55	0.93	Zimbabw e	Female	45-49 years	1.01	0.77	1.29
Zimbabw e	Both	50-54 years	1.61	1.22	2.09	Zimbabw e	Male	50-54 years	1.32	1.00	1.70	Zimbabw e	Female	50-54 years	1.86	1.42	2.44
Zimbabw e	Both	55-59 years	2.63	2.03	3.28	Zimbabw e	Male	55-59 years	2.12	1.65	2.63	Zimbabw e	Female	55-59 years	3.00	2.31	3.74
Zimbabw e	Both	60-64 years	3.93	3.10	4.91	Zimbabw e	Male	60-64 years	3.12	2.44	3.92	Zimbabw e	Female	60-64 years	4.47	3.48	5.62
Zimbabw e	Both	65-69 years	5.96	4.78	7.32	Zimbabw e	Male	65-69 years	4.65	3.71	5.70	Zimbabw e	Female	65-69 years	6.86	5.47	8.43
Zimbabw e	Both	70-74 years	8.62	6.64	10.83	Zimbabw e	Male	70-74 years	6.61	5.10	8.32	Zimbabw e	Female	70-74 years	9.96	7.60	12.69
Zimbabw e	Both	75-79 years	11.34	9.10	13.89	Zimbabw e	Male	75-79 years	8.65	6.98	10.61	Zimbabw e	Female	75-79 years	13.06	10.33	16.10
Zimbabw e	Both	All ages	0.56	0.48	0.64	Zimbabw e	Male	All ages	0.38	0.32	0.45	Zimbabw e	Female	All ages	0.71	0.60	0.82
Zimbabw e	Both	80-84	13.93	11.38	17.07	Zimbabw e	Male	80-84	10.63	8.68	13.12	Zimbabw e	Female	80-84	15.87	12.91	19.49
Zimbabw e	Both	85-89	15.95	13.05	19.29	Zimbabw e	Male	85-89	12.25	10.03	14.83	Zimbabw e	Female	85-89	17.81	14.55	21.63
Zimbabw e	Both	90-94	17.50	14.44	20.97	Zimbabw e	Male	90-94	13.56	11.13	16.35	Zimbabw e	Female	90-94	18.96	15.58	22.70

Supplementary Methods and Results

Methods

The data, study protocol and GBD 2019 methods that support the findings of this study have been published previously and are available at www.health.data.org. For this study, we obtained estimates of incidence, prevalence, cause-specific mortality, years of life lost (YLLs), years lived with disability (YLDs), and DALYs for PAD from GBD 2019, as described in the original GBD 2019 manuscript.[17-21] Consistent with previous iterations of GBD, PAD burden was estimated only among those aged at least 40 years.

Mortality estimates

Detailed methodology for cause-specific mortality estimation is described in the appendix (pp 51–91) and previous publiations.[17] Cause-specific mortality for PAD was estimated using the Cause of Death Ensemble model (CODEm) software with vital registration (VR) records as input data.[17] International Classification Disease (ICD) codes in VR records were mapped to the GBD cause list (ICD 10: I70.2-I70.8, I73-I73.9 and ICD 9: 440.2, 440.4, 443.0-443.9). Non-specific, intermediate, or implausible causes of death (e.g., "heart disease, unspecified", "heart failure", "senility", "hypertension") were reassigned to correct underlying causes of death, including PAD, via a set of redistribution algorithms developed for GBD 2019.[17] These algorithms utilize 1) proportional information, 2) cause-specific priors, or 3) data sets with complete information on all contributing causes of death in addition to the underlying cause.[17] The garbage code redistribution algorithm has been described in detail in the appendix (pp 63–69). Country-level covariates associated with PAD were included to inform the model. CODEm produces estimates of cause-specific mortality by age, sex, and location for each year by utilizing an ensemble of modeling methods with varying choices of covariates determined by model performance in out-of-sample predictive validity testing.[17] Possible covariates were selected based on

a priori knowledge of the association between the covariate and PAD; this list can be found in the appendix (pp 2–3). Covariates and combinations of covariates were tested for statistical significance and plausibility (the coefficient must be in the expected direction). Covariates meeting these criteria are retained in the final model.[17] Detailed methods describing the covariate selection process used in CODEm can be found in the appendix (pp 80–82) and elsewhere.[22] The results obtained with the Ensemble models were then adjusted by scaling them within the fraction of deaths due to all cardiovascular diseases and all-cause mortality. The 2·5 and 97·5th percentiles of the posterior distribution were used to determine uncertainty intervals.

Morbidity estimates

The studies eligible for assessing PAD prevalence were those that included an ABI measurement and defined PAD as ABI less than or equal to 0·90. We excluded literature with different ABI cut-offs to minimize inconsistency. In addition to published studies, we also included health system administrative data, including outpatient claims data for prevalence assessment. We adjusted administrative health care data using literature data reporting directly measured ABI values as reference data according to the standard adjustment procedure outlined in the appendix (pp 93–116). Details of the search strategy and a full list of the input data sources used in the morbidity analysis can be found in the appendix (pp 5–9). When calculating YLDs, we only accounted burden from PAD with intermittent claudication. Intermittent claudication was defined clinically;[23] as leg pain on exertion in those with an ankle-brachial index (ABI) less than or equal to 0·90. We used DisMod-MR to model the proportion of PAD with intermittent claudication and used the proportion of intermittent claudication to split the overall prevalence of PAD into symptomatic and asymptomatic PAD. This approach has been used in previous GBD papers to split prevalence of disease by stage,[24] symptom,[25] and severity.[26] The list of studies we used to calculate the proportion of claudication and more description are provided in appendix (pp 6–11).

Estimates of overall PAD prevalence and the proportion of PAD cases with intermittent claudication were calculated using two separate DisMod-MR 2.1 models.[17] DisMod-MR is a Bayesian geospatial disease modeling approach that uses different disease parameters (e.g., prevalence, incidence, remission, and mortality), epidemiological relationships between these parameters, and geospatial patterns to generate disease estimates. The model ensures consistency among all disease parameters by employing differential equations with suitable boundary conditions. The tool incorporates an offset log-normal model with fixed effects for location-specific covariates and random effects for locations. The covariates included in the models can be found in the appendix (pp 101–124). Estimates were made for 7 super-regions, 21 world regions, and 204 countries and territories utilizing a geographic cascade as described in the appendix (pp 116–117). Disease distributions from higher geographical levels were used as priors to information for the next levels, and the 2·5 and 97·5th percentiles of the posterior distribution were used to determine uncertainty intervals. The PAD DisMod-MR models were evaluated based on comparisons with estimates from prior iterations of GBD and expert review via the GBD collaborator network.[17]

Summary burden measures

To aid in comparisons of disease burden across locations, the GBD computes three summary measures. YLLs were calculated as the difference between the age of death for PAD and the maximum life expectancy across all locations observed in the GBD. To estimate YLDs caused by PAD, we used the proportion of intermittent claudication to split the overall prevalence of PAD into symptomatic and asymptomatic PAD. YLDs were calculated as the product of the disability weights[27] for symptomatic and asymptomatic PAD and the corresponding prevalence; information on disability weights for these two health states can be found in the appendix (p 10). DALYs are calculated as the sum of YLLs and YLDs to provide a comprehensive picture of the disease burden due to each cause. Age-standardised rates per 100 000 population were computed by the direct method to the GBD population standard.

Risk factors

The GBD comparative risk assessment framework was used to estimate the burden of PAD attributable to six risk factors: smoking, high fasting plasma glucose, high blood pressure, kidney dysfunction, high sodium intake, and lead exposure. These risks were selected based on the following criteria: sufficient evidence for causation for each risk outcome pair using the Bradford Hill; availability of risk exposure data; and potential for risk modification and policy relevance. PAD-related attributable burden was estimated by age, sex, country, and year. Estimating GBD risk factors involves six steps. The first is identifying risk-outcome pairs with convincing or plausible evidence. The second is calculating the relative risk of exposure for each risk-outcome pair. The third is calculating the exposure levels for each risk factor based on age, sex, location, and year. The fourth is establishing a theoretical minimum risk exposure level (TMREL). The fifth is calculating the population attributable fraction and attributable burden, utilizing the relative risk, exposure levels, and TMREL calculated in the ahead steps.[17] The final step is calculating the total disease burden attributable to all risk factors, after accounting for a possible mediation between covariates. Further information about the methodology used and the individual steps is available in the appendix (pp 12–50) and in prior GBD publications.[17]

Sociodemographic index and income

Raw numbers and age-standardised rates of prevalence, incidence, DALY, and mortality of PAD were extracted from GBD 2019 and stratified by geographic region, sex, age, sociodemographic index (SDI), and World Bank income level. We measured the social and developmental status of each country through two measures: sociodemographic index (SDI) and average income. SDI is defined in the GBD study as a composite score of fertility under age 25, average education for individuals over age 15, and lag-distributed income per capita; the index range from 0 to 1.[18] Quintiles of ranked SDI values (i.e., low, low-middle, middle, high-middle, and high SDI) were obtained from the GBD 2019 data and used in the analysis. We also obtained the World Bank income level of each country and classified countries

into high-income (HIC), upper middle-income (UMIC), lower middle-income (LMIC), and low-income (LIC) countries.

Results

Global Burden of PAD

The age-standardised DALYs, mortality, prevalence, and incidence rates of PAD at the country level are shown in Figure 1A-D. In 2019, a global prevalence of PAD is 1·52% (1·33-1·72); the prevalence of PAD was substantially higher in females (2·03% [1·77-2·3]) than males (1·01% [0·88-1·16]) from 1990–2019 (Table 1). The global prevalence of PAD was much higher in the elderly, where the global prevalence of symptomatic PAD was 14·91% (12·41-17·87) in those aged 80-84, and the prevalence diverged by sex (18·03% (15·01-21·63) in females and 10·56% (8·78-12·76) in males). Globally, the total number of patients with PAD almost doubled from 65·8 million [95% uncertainty interval (UI) 57·2–74·5] in 1990 to 113 million [99·2–128·4] in 2019 (appendix pp 140–151, Figure 2A). However, global age-standardised prevalence rates decreased during the study period, from 1,790 [1,564–2,033] per 100 000 population in 1990 to 1,402 [1,229–1,589] per 100 000 population in 2019, a 21·7% [20·5–22·8] decrease (appendix pp 140–151, Figure 2B). Likewise, the total number of DALYs increased twofold from 0·776 million [0·488–1·178] in 1990 to 1·536 million [1·007–2·370] in 2019, while age-standardised DALYs rates decreased from 22·4 [14·1–34·1] to 19.6 [12·9–30·2] per 100 000 population in 1990 and 2019, respectively (appendix pp 152–163, Figure 2).

PAD accounted for a total of 74,063 [41,183–128,164] deaths in 2019, resulting in an age-standardised mortality rate of 1·0 [0·6–1·7] per 100 000 population (appendix pp 164–175, Figure 2A-B), which was fairly constant over the period of 1990-2019. A total number of incidence cases increased

from 6·13 million [5·32–7·00] to 10.50 million [9·16–12·00] in 1990 and 2019, respectively, while age-standardised incident rates decreased 18·9% [18·0–19·8] resulting in incident rates of 127·1 [111·3–145·5] per 100 000 population in 2019 (appendix pp 176–187, Figure 2A-B).

Stratified by 21 GBD-defined regions, High-income North America and Western Europe regions had the highest and second-highest age-standardised prevalence rate of PAD at 2,214 [1,987–2,434] and 1,903 [1,659–2,145] per 100 000 population, respectively, in 2019 (appendix pp 140–151); these were 29·2% [23·7–33·7] and 34·2% [33·3–35·1] decreases from 1990, respectively. The region with the lowest prevalence rate was Andean Latin America, where the age-standardised prevalence rate was 828·7 [715·4–951·2]. Although age-standardised prevalence rates decreased globally, the trend varied widely, with High-income Asia Pacific having experienced a decrease as large as 41·6% [40·8–42·3] and most countries in Oceania (9·3% [6·6–12·9]), East Asia (6·8% [5·5–8·1]), and Southeast Asia (4·2% [2·8–5·7]) reporting increases in PAD prevalence rates from 1990 to 2019.

Eastern Europe had the highest age-standardised DALYs rates of PAD in 2019 at 63·6 [33·5–117·8] per 100 000 population, a 25·9% [-9·8–54·4] increase since 1990. Central and Southern sub-Saharan Africa followed with DALYs rates at 43·1 [24·0–65·5] and 41·8 [33·4– 49·9] per 100 000, respectively. The lowest DALYs rates were reported by Andean Latin America and High-income Asia Pacific regions in 2019 (5·9 [3·7–9·2] and 7·5 [4·5–12·0], respectively). Change in DALYs rates varied widely across countries; many countries, for example, Slovakia (91·4% [22·3–182·8]) and Serbia (69·0% [23·3–132·9]), experienced a steep increase in PAD DALYs rates from 1990 to 2019, while others underwent a precipitous decrease over time, the drop being as large as 47·7% [54·7–40·1] in the Republic of Korea.

Burden of PAD according to demographic factors

Consistently throughout the study period 1990-2019, numbers and age-standardised prevalence and incidence rates of PAD were substantially higher in females than in males (appendix p 138). On the other hand, the total number and age-standardised rate of DALYs and mortality for PAD were

comparable across both sexes throughout the study period (appendix p 138). Stratified by age, in 2019, prevalence, deaths, and DALYs rates increased steeply with increasing age (appendix p 138). In contrast, the age-specific prevalence and incidence cases had a unimodal distribution for both sexes, peaking at age 70-74 and 65-69, respectively (Figure 3A-B). For DALYs, the distribution was similar for males with the highest DALYs occurring at age 70-74, but the distribution was skewed towards higher ages for females, with highest DALYs for PAD at age 80-84 (Figure 3C). Death counts were greatest for females aged 85-89 and males aged 80-84 (Figure 3D).

Burden of PAD according to SDI

The disease burden of PAD had a distinct association with SDI level (appendix p 135). Higher SDI quintiles tended to have higher DALYs and mortality rates, with High and High-Middle quintiles having the highest DALYs and mortality rates across the study period (1·5 [0·7–2·8] and 1·4 [0·7–2·4] PAD deaths per 100 000 population in 2019, respectively) and Middle and Low-middle SDI quintiles with the lowest rates (0·4 [0·3–0·5] PAD deaths per 100 000 population in 2019 for both Middle and Low-middle quintiles); the Low SDI quintile, however, was located in the middle, having DALYs and mortality rates higher than the Low-middle and Middle quintiles and lower than the High and High-middle quintiles (0·7 [0·4–1·0] PAD deaths per 100 000 population in 2019) (appendix pp 164–175). For Middle and Low-middle SDI quintiles, age-standardised mortality rates of PAD increased from 1990 to 2019 (29·3% [4·3–49·8] and 55·4% [21·0–82·4] increase, respectively); the trend was not significant in High, High-middle, and Low SDI quintiles (appendix pp 164–175).

The age-standardised prevalence rates of PAD increased with increasing SDI quintile, with the High SDI quintile having the highest prevalence rates and the Low SDI quintile having the lowest prevalence rates in 2019 (1,794 [1,585–2,006] and 938·6 [815·0–1,074·6] per 100 000 population, respectively)(appendix pp 140–151). Further, higher SDI quintiles underwent a steep decrease in PAD age-standardised prevalence rates, with High and High-middle SDI quintiles having 34·0% [32·0–35·8] and 15·4% [14·2–16·4] drops in age-standardised prevalence rates, respectively, from 1990 to 2019;

however, in lower SDI quintiles, the rates remain stable over time (appendix p 135). The directionality and slope of association between SDI and age-standardised DALYs, mortality, prevalence, and incidence rates of PAD differed according to geographic region (appendix p 136). Meanwhile, there was an overall positive correlation between higher SDI and higher age-standardised DALYs rate (appendix pp 137).

This trend was replicated with the analysis using the World Bank income level; DALYs and mortality rates of PAD had a U-shape with the high DALYs and mortality rates occurring in the highest and lowest income levels (Figure 4A-B). In contrast, prevalence and incidence rates of PAD increased stepwise with increasing income level (Figure 4C-D).

Risk factors

The total number of PAD DALYs globally attributable to all estimated risk factors in 2019 was 1·066 million [0·690–1·646] for both sexes combined, which accounted for 69·4% (64·2–74·3) of all PAD DALYs. Males were estimated to have 0·589 million (0·333–1·072) PAD DALYs attributable to risk factors, or 76·9% (72·7–80·4) of all PAD DALYs in males, whereas females were estimated to have 0·477 million (0·285–0·760) PAD DALYs attributable to risk factors, or 62·0% (57·0–67·0) of all PAD DALYs in females. Figure 5 depicts the contribution of six risk factors to age-standardised DALYs rate due to PAD, for males and females, for global regions in 2019. In males, the age-standardised DALYs rate for PAD attributed to smoking was 9·5 (5·1–17·2), high fasting plasma glucose was 6·4 (3·5–12·0), high blood pressure was 5·9 (3·2–10·8), kidney dysfunction was 3·6 (1·9–6·8), high sodium was 0·9 (0·2–2·4), and lead was 0·3 (0·2–0·6)(Figure 5). In females, the age-standardised DALYs rate for PAD attributed to smoking was 3·1 (1·7–5·4), high fasting plasma glucose was 4·6 (2·7–7·6), high blood pressure was 4·8 (2·7–8·3), kidney dysfunction was 2·7 (1·5–4·4), high sodium was 0·5 (0·1–1·5), and lead was 0·2 (0·1–0·4)(Figure 5).

Authors' Contributions

Providing data or critical feedback on data sources

Mohammadreza Abbasian, Denberu Eshetie Adane Adane, Oladimeji M Adebayo, Fares Alahdab, Sami Almustanyir, Hany Aly, Edward Kwabena Ameyaw, Jason Anderson, Zahra Aryan, Sara Bagherieh, Ovidiu Constantin Baltatu, Maciej Banach, Nebiyou Simegnew Bayileyegn, Sonu Bhaskar, Ajay Nagesh Bhat, Bagas Suryo Bintoro, Vijay Kumar Chattu, Massimo Cirillo, Xiaochen Dai, Thanh Chi Do, Rajkumar Doshi, Michael Ekholuenetale, Islam Y Elgendy, Adeniyi Francis Fagbamigbe, Alireza Feizkhah, Ginenus Fekadu, Mahaveer Golechha, Vivek Kumar Gupta, Jimin Hwang, Catherine Owens Johnson, Jost B Jonas, Charity Ehimwenma Joshua, Jacek Jerzy Jozwiak, Se Yong Jung, Moien AB Khan, Sorour Khateri, Min Seo Kim, Oleksii Korzh, Sindhura Lakshmi Koulmane Laxminarayana, Kewal Krishan, Akshay Kumar, Manoj Kumar, Tri Laksono, Thao Thi Thu Le, Seung Won Lee, Stephen S Lim, Xuefeng Liu, Stefan Lorkowski, Hassan Magdy Abd El Razek, Kashish Malhotra, Andrea Maugeri, Erkin M Mirrakhimov, Awoke Misganaw, Ali H Mokdad, Ahmed Al Montasir, Yousef Moradi, Christopher J L Murray, Mohsen Naghavi, Zuhair S Natto, Dang H Nguyen, Hien Quang Nguyen, Van Thanh Nguyen, Jean Jacques Noubiap, Bogdan Oancea, Mayowa O Owolabi, Seoyeon Park, Pradhum Ram, Sina Rashedi, Ahmed Mustafa Rashid, Salman Rawaf, Christian Razo, Andre M N Renzaho, Leonardo Roever, Aly M A Saad, Seyedmohammad Saadatagah, Mohamed A Saleh, Abdallah M Samy, Milena M Santric-Milicevic, Allen Seylani, Jae Il Shin, Jasvinder A Singh, Michael Spartalis, Johan Sundström, Ker-Kan Tan, Sahel Valadan Tahbaz, Jef Van den Eynde, Ronny Westerman, Suowen Xu, Dereje Y Yada, Kazumasa Yamagishi, Dong Keon Yon, Naohiro Yonemoto, Mikhail Sergeevich Zastrozhin, and Mohammad Zoladl.

Developing methods or computational machinery

Sonu Bhaskar, Kaleb Coberly, Xiaochen Dai, Thanh Chi Do, Alireza Feizkhah, Simon I Hay, Catherine Owens Johnson, Sorour Khateri, Manoj Kumar, Thao Thi Thu Le, Seung Won Lee, Ali H Mokdad, Mohammad Ali Moni, Yousef Moradi, Christopher J L Murray, Mohsen Naghavi, Dang H Nguyen, Hien Quang Nguyen, Van Thanh Nguyen, Seoyeon Park, Christian Razo, Abdallah M Samy, Jae Il Shin, Michael Spartalis, Benjamin A Stark, Ronny Westerman, and Mikhail Sergeevich Zastrozhin.

Providing critical feedback on methods or results

Mohammadreza Abbasian, Hedayat Abbastabar, Amir Parsa Abhari, Victor Aboyans, Denberu Eshetie Adane Adane, Oladimeji M Adebayo, Fares Alahdab, Sami Almustanyir, Hany Aly, Edward Kwabena Ameyaw, Catalina Liliana Andrei, Zahra Aryan, Avinash Aujayeb, Sara Bagherieh, Ovidiu Constantin Baltatu, Maciej Banach, Nebiyou Simegnew Bayileyegn, Lindsay M Bearne, Amir Hossein Behnoush, Isabela M Bensenor, Sonu Bhaskar, Ajay Nagesh Bhat, Vivek Bhat, Boris Bikbov, Luis Alberto Cámera, Alberico L Catapano, Eeshwar K Chandrasekar, Jaykaran Charan, Vijay Kumar Chattu, Gerald Chi, Isaac Sunday Chukwu, Sheng-Chia Chung, Massimo Cirillo, Vera Marisa Costa, Omid Dadras, Xiaochen Dai, Thanh Chi Do, Rajkumar Doshi, Michael Ekholuenetale, Muhammed Elhadi, Adeniyi Francis Fagbamigbe, Alireza Feizkhah, Paramjit Singh Gill, Mohamad Goldust, Mahaveer Golechha, Shi-Yang Guan, Vivek Kumar Gupta, Mostafa Hadei, Najah R Hadi, Ahmad Hammoud, Netanja I Harlianto, Ahmed I Hasaballah, Shoaib Hassan, Mohammed Bheser Hassen, Simon I Hay, Golnaz Heidari, Mihaela Hostiuc, Jimin Hwang, Olayinka Stephen Ilesanmi, Masao Iwagami, Catherine Owens Johnson, Mohammad Jokar, Jost B Jonas, Charity Ehimwenma Joshua, Jacek Jerzy Jozwiak, Se Yong Jung, Sina Kazemian, Mohammad Keykhaei, Amirmohammad Khalaji, Moien AB Khan, Sorour Khateri, Biruk Getahun Kibret, Min Seo Kim, Oleksii

Korzh, Sindhura Lakshmi Koulmane Laxminarayana, Kewal Krishan, Akshay Kumar, Manoj Kumar, Ambily Kuttikkattu, Tri Laksono, Bagher Larijani, Thao Thi Thu Le, Seung Won Lee, Stephen S Lim, Xuefeng Liu, Stefan Lorkowski, Hassan Magdy Abd El Razek, Kashish Malhotra, Yosef Manla, Andrea Maugeri, Alexios-Fotios A Mentis, Tomislav Mestrovic, Andreea Mirica, Erkin M Mirrakhimov, Awoke Misganaw, Manish Mishra, Yousef Mohammad, Ali H Mokdad, Mohammad Ali Moni, Ahmed Al Montasir, Yousef Moradi, Negar Morovatdar, Seyed Ali Mousavi-Aghdas, Christopher J L Murray, Mohsen Naghavi, Tapas Sadasivan Nair, Hasan Nassereldine, Zuhair S Natto, Dang H Nguyen, Hien Quang Nguyen, Van Thanh Nguyen, Jean Jacques Noubiap, Bogdan Oancea, Gláucia Maria Moraes Oliveira, Mayowa O Owolabi, Alicia Padron-Monedero, Seoyeon Park, Ionela-Roxana Petcu, Amir Radfar, Quinn Rafferty, Mosiur Rahman, Muhammad Aziz Rahman, Pradhum Ram, Sina Rashedi, Ahmed Mustafa Rashid, Salman Rawaf, Christian Razo, Andre M N Renzaho, Malihe Rezaee, Leonardo Roever, Gregory A Roth, Aly M A Saad, Seyedmohammad Saadatagah, Mohamed A Saleh, Abdallah M Samy, Milena M Santric-Milicevic, Sadaf G Sepanlou, Sadaf Sharfaei, Jae Il Shin, Seyed Afshin Shorofi, Jasvinder A Singh, Paramdeep Singh, Michael Spartalis, Ker-Kan Tan, Masayuki Teramoto, Samar Tharwat, Stefanos Tyrovolas, Sahel Valadan Tahbaz, Jef Van den Eynde, Priya Vart, Cong Wang, Ronny Westerman, Juan Xia, Kazumasa Yamagishi, Dong Keon Yon, Naohiro Yonemoto, Mazyar Zahir, Moein Zangiabadian, Armin Zarrintan, Mikhail Sergeevich Zastrozhin, and Mohammad Zoladl.

Drafting the work or revising it critically for important intellectual content

Mohammadreza Abbasian, Hedayat Abbastabar, Denberu Eshetie Adane Adane, Oladimeji M Adebayo, Fares Alahdab, Sami Almustanyir, Hany Aly, Catalina Liliana Andrei, Zahra Aryan, Avinash Aujayeb, Sara Bagherieh, Ovidiu Constantin Baltatu, Maciej Banach, Lindsay M Bearne, Amir Hossein Behnoush, Isabela M Bensenor, Sonu Bhaskar, Ajay Nagesh Bhat, Vivek Bhat, Boris Bikbov, Alberico L Catapano, Eeshwar K Chandrasekar, Vijay Kumar Chattu, Gerald Chi, Massimo Cirillo, Vera Marisa Costa, Thanh Chi Do, Rajkumar Doshi, Islam Y Elgendy, Muhammed Elhadi, Adeniyi Francis Fagbamigbe, Alireza Feizkhah, Paramjit Singh Gill, Mohamad Goldust, Shi-Yang Guan, Vivek Kumar Gupta, Mostafa Hadei, Najah R Hadi, Ahmad Hammoud, Graeme J Hankey, Ahmed I Hasaballah, Shoaib Hassan, Simon I Hay, Golnaz Heidari, Jimin Hwang, Olayinka Stephen Ilesanmi, Catherine Owens Johnson, Jost B Jonas, Jacek Jerzy Jozwiak, Se Yong Jung, Amirmohammad Khalaji, Moien AB Khan, Biruk Getahun Kibret, Min Seo Kim, Oleksii Korzh, Sindhura Lakshmi Koulmane Laxminarayana, Kewal Krishan, Akshay Kumar, Manoj Kumar, Bagher Larijani, Thao Thi Thu Le, Seung Won Lee, Stefan Lorkowski, Hassan Magdy Abd El Razek, Andrea Maugeri, Alexios-Fotios A Mentis, Tomislav Mestrovic, Ana Carolina Micheletti Gomide Nogueira de Sá, Awoke Misganaw, Manish Mishra, Yousef Mohammad, Ali H Mokdad, Mohammad Ali Moni, Ahmed Al Montasir, Paula Moraga, Negar Morovatdar, Christopher J L Murray, Mohsen Naghavi, Hasan Nassereldine, Zuhair S Natto, Dang H Nguyen, Hien Quang Nguyen, Van Thanh Nguyen, Jean Jacques Noubiap, Bogdan Oancea, Gláucia Maria Moraes Oliveira, Mayowa O Owolabi, Alicia Padron-Monedero, Seoyeon Park, Norberto Perico, Ionela-Roxana Petcu, Amir Radfar, Pradhum Ram, Salman Rawaf, Christian Razo, Giuseppe Remuzzi, Malihe Rezaee, Leonardo Roever, Gregory A Roth, Aly M A Saad, Seyedmohammad Saadatagah, Masoumeh Sadeghi, Amirhossein Sahebkar, Abdallah M Samy, Milena M Santric-Milicevic, Sadaf G Sepanlou, Allen Seylani, Jae Il Shin, Seyed Afshin Shorofi, Jasvinder A Singh, Paramdeep Singh, Michael Spartalis, Benjamin A Stark, Johan Sundström, Ker-Kan Tan, Masayuki Teramoto, Samar Tharwat, Stefanos Tyrovolas, Sahel Valadan Tahbaz, Jef Van den Eynde, Priya Vart, Fang Wang, Ronny Westerman, Juan Xia, Kazumasa Yamagishi, Dong Keon Yon, Naohiro Yonemoto,

Mazyar Zahir, Armin Zarrintan, Mikhail Sergeevich Zastrozhin, Anasthasia Zastrozhina, and Mohammad Zoladl.

Managing the estimation or publications process

Thanh Chi Do, Simon I Hay, Min Seo Kim, Akshay Kumar, Manoj Kumar, Thao Thi Thu Le, Hassan Magdy Abd El Razek, Ali H Mokdad, Christopher J L Murray, Mohsen Naghavi, Dang H Nguyen, Hien Quang Nguyen, Van Thanh Nguyen, Christian Razo, Aly M A Saad, Abdallah M Samy, Jae Il Shin, Michael Spartalis, Dong Keon Yon, Mikhail Sergeevich Zastrozhin, and Anasthasia Zastrozhina.
